# Vulnerability to snakebite envenoming: a global mapping of hotspots

**DOI:** 10.1016/S0140-6736(18)31224-8

**Published:** 2018-08-25

**Authors:** Joshua Longbottom, Freya M Shearer, Maria Devine, Gabriel Alcoba, Francois Chappuis, Daniel J Weiss, Sarah E Ray, Nicolas Ray, David A Warrell, Rafael Ruiz de Castañeda, David J Williams, Simon I Hay, David M Pigott

**Affiliations:** a Big Data Institute, Li Ka Shing Centre for Health Information and Discovery, University of Oxford, Oxford, UK; b Nuffield Department of Clinical Medicine, University of Oxford, Oxford, UK; c Centre for Health Informatics, Computing and Statistics, Lancaster Medical School, Lancaster University, Lancaster, UK; d Department of Vector Biology, Liverpool School of Tropical Medicine, Liverpool, UK; e Division of Tropical and Humanitarian Medicine, University Hospitals of Geneva, Geneva, Switzerland; f Division of Tropical Medicine and Neglected Tropical Diseases, Médecins Sans Frontières, Geneva, Switzerland; g Institute for Health Metrics and Evaluation, University of Washington, Seattle, WA, USA; h EnviroSPACE Lab, Institute for Environmental Sciences, University of Geneva, Geneva, Switzerland; i Institute of Global Health, Faculty of Medicine, University of Geneva, Geneva, Switzerland; j Australian Venom Research Unit, Department of Pharmacology and Therapeutics, University of Melbourne, Melbourne, VIC, Australia

## Abstract

**Background:**

Snakebite envenoming is a frequently overlooked cause of mortality and morbidity. Data for snake ecology and existing snakebite interventions are scarce, limiting accurate burden estimation initiatives. Low global awareness stunts new interventions, adequate health resources, and available health care. Therefore, we aimed to synthesise currently available data to identify the most vulnerable populations at risk of snakebite, and where additional data to manage this global problem are needed.

**Methods:**

We assembled a list of snake species using WHO guidelines. Where relevant, we obtained expert opinion range (EOR) maps from WHO or the Clinical Toxinology Resources. We also obtained occurrence data for each snake species from a variety of websites, such as VertNet and iNaturalist, using the spocc R package (version 0.7.0). We removed duplicate occurrence data and categorised snakes into three groups: group A (no available EOR map or species occurrence records), group B (EOR map but <5 species occurrence records), and group C (EOR map and ≥5 species occurrence records). For group C species, we did a multivariate environmental similarity analysis using the 2008 WHO EOR maps and newly available evidence. Using these data and the EOR maps, we produced contemporary range maps for medically important venomous snake species at a 5 × 5 km resolution. We subsequently triangulated these data with three health system metrics (antivenom availability, accessibility to urban centres, and the Healthcare Access and Quality [HAQ] Index) to identify the populations most vulnerable to snakebite morbidity and mortality.

**Findings:**

We provide a map showing the ranges of 278 snake species globally. Although about 6·85 billion people worldwide live within range of areas inhabited by snakes, about 146·70 million live within remote areas lacking quality health-care provisioning. Comparing opposite ends of the HAQ Index, 272·91 million individuals (65·25%) of the population within the lowest decile are at risk of exposure to any snake for which no effective therapy exists compared with 519·46 million individuals (27·79%) within the highest HAQ Index decile, showing a disproportionate coverage in reported antivenom availability. Antivenoms were available for 119 (43%) of 278 snake species evaluated by WHO, while globally 750·19 million (10·95%) of those living within snake ranges live more than 1 h from population centres. In total, we identify about 92·66 million people living within these vulnerable geographies, including many sub-Saharan countries, Indonesia, and other parts of southeast Asia.

**Interpretation:**

Identifying exact populations vulnerable to the most severe outcomes of snakebite envenoming at a subnational level is important for prioritising new data collection and collation, reinforcing envenoming treatment, existing health-care systems, and deploying currently available and future interventions. These maps can guide future research efforts on snakebite envenoming from both ecological and public health perspectives and better target future estimates of the burden of this neglected tropical disease.

**Funding:**

Bill & Melinda Gates Foundation.

## Introduction

Snakebite envenoming is a frequently overlooked cause of mortality and morbidity, responsible for 81 000–138 000 deaths annually,^1^, ^2^ and between 421 000 and 1·2 million envenomings.^3^ Contact from venomous snakes, spiders, and scorpions contribute to 1·2 million years of life lived with disability annually.^4^ The burden remains poorly characterised because of under-reporting; as snakebite is rarely notifiable, existing estimates are typically derived from extrapolated hospital records and community surveys.^5^ Snakebite primarily affects the poor rural communities of Asia and sub-Saharan Africa, where socioeconomic status and agricultural and other practices contribute to increased snake–human interaction.^6^ Venomous snakebites can also inflict a heavy burden on livestock, creating economic hardship for already impoverished communities.^7^ Medically important snake species, however, have a cosmopolitan distribution, making snakebite a global challenge.^3^

In June, 2017, snakebite envenoming was classified as a category A neglected tropical disease,^8^, ^9^ and was the subject of a resolution passed by the World Health Assembly in May, 2018. Consequently, there is a renewed impetus to accurately assess the burden and distribution of snakebite to ensure appropriate prevention and control interventions are implemented, and that adequate resources and funding are allocated nationally and subnationally.^10^, ^11^ For other neglected tropical diseases, substantive global targets exist: Sustainable Development Goal target 3·3 aims to “end the epidemics” of these diseases by 2030,^12^, ^13^ with routine reporting, surveillance, and notification architecture in place. As a new neglected tropical disease, snakebite monitoring and evaluation should reflect these objectives.

**Research in context**

**Evidence before this study**

Snakebite envenoming is a category A neglected tropical disease of particular public health importance in tropical areas of Africa, Asia, Latin America, and Papua New Guinea. It is estimated that up to 1·2 million people are envenomed annually, resulting in 81 000–138 000 fatalities. Although effective therapies exist to treat envenoming by some snakes of highest medical importance, there are many species without such treatments. The global distribution of venomous snakes and vulnerable populations remains inadequately characterised; therefore, the lack of knowledge of subnational disease burden might impede production of antivenom supplies and distribution efforts among populations currently at risk. To investigate this further, we searched for articles on PubMed published before March 1, 2017, using the search terms “snakebite”, “distribution”, and “burden”. Contemporary studies have investigated venomous snake distributions and snakebite risk at national levels (several countries in Latin America) or subnational levels (India, Nigeria, and Sri Lanka), but these studies did not encompass all medically important snake species and are limited in both geographical extent and spatial resolution. A more recent analysis mapped the distribution of venomous snakes in Central America and Latin America but was restricted to widely studied species with ample occurrence data. Although an important start, no study has coupled global ecological information about snake distributions with measures relating to public health capabilities to hone in on populations most vulnerable to this cause of mortality and morbidity.

**Added value of this study**

We identified populations most vulnerable to 278 medically important snake species by using expert opinion, species' ranges refined by publicly available occurrence data and multivariate analyses, information about effective therapies, and metrics of health-care quality and accessibility. Although a large proportion of the world's population live in areas where such snakes could be present, proxy metrics such as the Healthcare Access and Quality Index and urban accessibility paired with broad-scale information about market antivenom availability provide a subnationally resolved yet globally comprehensive picture of vulnerability, highlighting populations that could be most affected.

**Implications of all the available evidence**

We highlight locations where the combination of the presence of a variety of venomous snakes, inequalities in health care and accessibility, and possible absence of effective therapy might contribute toward increased vulnerability of snakebite envenoming. Our analyses can be used to inform the positioning of local-scale household surveys to assess the true risk of snakebite in areas where such estimates are currently inadequate. This study highlights the importance of continuing to iterate, improve, and re-evaluate existing geographical assessments of snake distributions, and the need to incorporate spatially heterogeneous risk within future burden estimation efforts. This work is a first step in trying to identify and assist the most neglected populations of this newly designated neglected tropical disease.

Data for the presence of venomous snakes and occurrence of snakebites are sparse and incomplete at the global level, making estimation challenging.^14^, ^15^ Although some countries have done household-level surveys to determine the incidence of snakebites,^14^, ^15^ the global magnitude of this disease remains poorly characterised. Snakebite envenoming represents an interesting One Health challenge requiring clinical, ecological, and public health expertise. Consequently, this issue can be approached by considering vulnerability to snakebite envenoming as a nexus of ecological contexts and public health weaknesses, to provide an evidence base for targeting future quantitative studies.

Clinical challenges involve appropriate case diagnosis and adequate provisioning of care whether supportive (such as ventilators) or direct treatment with antivenom, which might not be available at any given point of care.^2^, ^16^ Ascertainment of the correct antivenom can be challenging,^17^ and current diagnostics can be expensive and slow.^18^, ^19^ Furthermore, nearly half of venomous snakes do not have antivenoms available as tracked by WHO.^2^, ^20^ To comprehensively address snakebites, these clinical challenges need to be considered within an ecological context by understanding snake behaviour and life-history traits that contribute to the frequency and geographical distribution of snakebites. Therefore, by contextualising contemporary knowledge about snake distributions with indicators of the quality of health-care provisioning,^21^ the accessibility of these resources,^22^ and antivenom availability,^20^ we aimed to identify populations vulnerable to the worst health outcomes of an envenoming event.

## Methods

### Study overview

**Figure 1:**
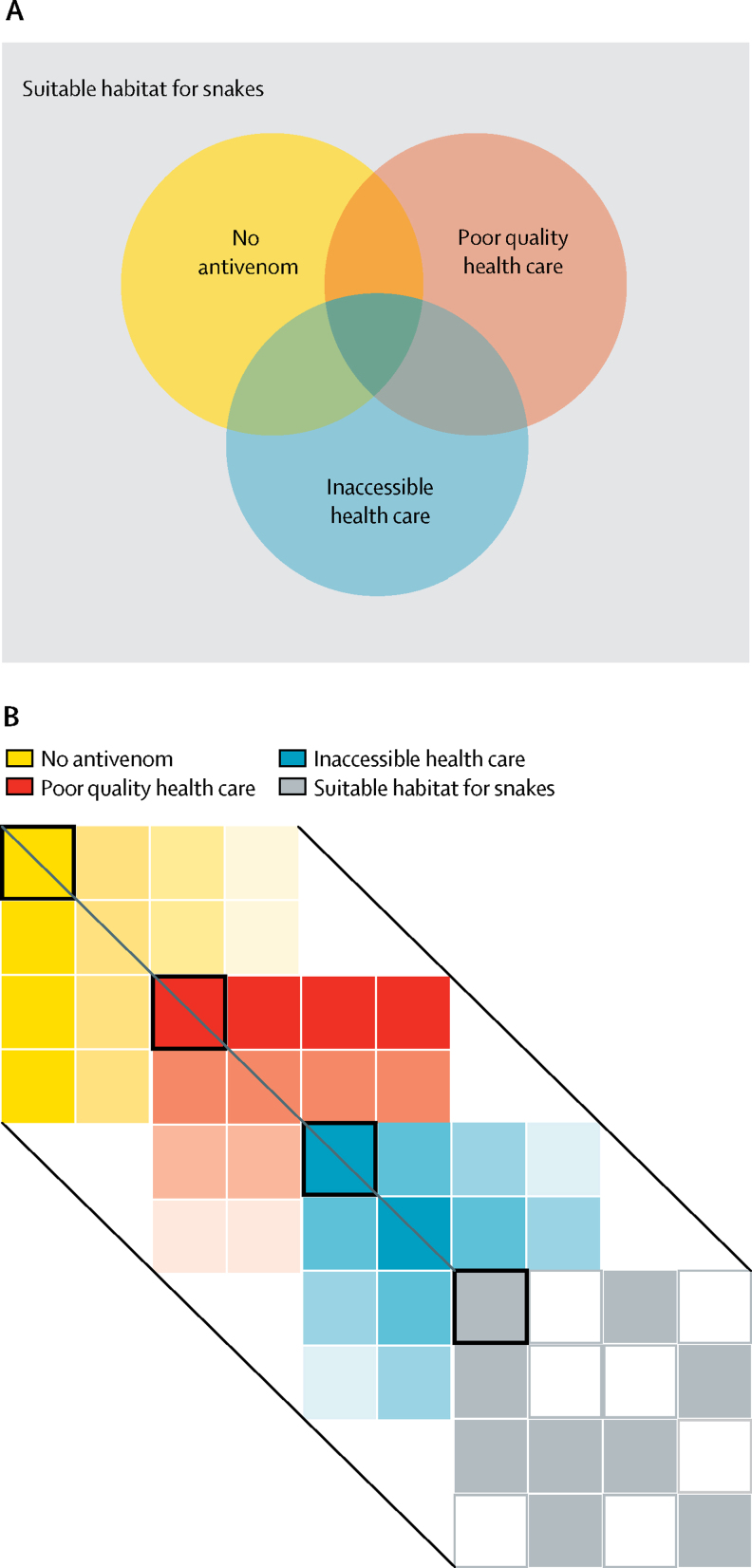
Conceptual overview of vulnerability to snakebite envenoming (A) Vulnerability can be considered as the intersection of populations who live within the range of venomous snakes that have no antivenoms available, cannot easily access health care, and have poor quality health care in delivery of antivenoms or ensuring necessary stocks. The intersection of all three defines the most vulnerable populations. (B) These factors vary in space. By overlaying these features, the most vulnerable populations can be identified spatially (represented here by the boxes outlined in black).

We evaluated range maps for 278 snakes to consider their presence at a 5 × 5 km (grid cell) resolution. To identify the most vulnerable populations, this ecological information was paired with three key metrics: the market availability of antivenom therapies as reported by WHO,^20^ accessibility to urban centres as a proxy for access to health care,^22^ and the Healthcare Access and Quality (HAQ) Index as a proxy for adequacy and efficacy of medical interventions at health-care centres.^21^ Figure 1 shows conceptually how populations lacking in all these measures should be seen as the most vulnerable populations, and how these measures could vary geographically.

### Global list of snake species

We assembled a list of snake species, using WHO guidelines for venomous snake species of medical importance (hereafter referred to as snakes),^23^ which define two tiers of medical importance that reflect both ecological knowledge on propensity to interact with humans and clinical grading of toxicity. Category one species are common or widespread snakes that result in high morbidity, disability, or mortality. Category two species are snakes capable of causing morbidity, disability, or death, or for which epidemiological or clinical data are missing or are less frequently implicated.

Where relevant, expert opinion range (EOR) maps were obtained from WHO blood products online database or the Clinical Toxinology Resources database.^20^, ^24^ Occurrence data for each species were obtained from the Global Biodiversity Information Framework, VertNet (version 2016-09-29), iNaturalist, iDigBio, and Ecoengine, using the spocc R package (version 0.7.0) on May 29, 2017.^25^ Duplicate records based upon shared collection year and latitude or longitude and those missing latitude or longitude were removed.

Given the availability of data, we placed snakes into three groups: group A (no available EOR map or species occurrence records), group B (EOR map but <5 species occurrence records), and group C (EOR map and ≥5 species occurrence records). Group A species (n=9) were excluded from this analysis because of the absence of geographical information, reducing our species inclusion list to 278 (99 group B species and 179 group C species; appendix 1).

### Multivariate environmental similarity surface generation and species' ranges

For group C species with sufficient occurrence records, potential updates to the EOR maps were assessed. EOR maps were updated with data that has become publicly accessible since publication of the WHO EOR maps in 2008. Multivariate environmental similarity method was applied to the occurrence records obtained for group C species, situated within the EOR, allowing for rapid classification of occurrence records outside of the EOR within the environmental range of other records (ie, interpolation) or beyond these limits (ie, extrapolation). Multivariate environmental similarity surfaces (MESS) measure the similarity between new environments (records outside of the EOR) and those in the training sample (records within the EOR), by identifying the maximum and minimum values of environmental data within the training sample, with respect to a set of predictor variables (covariates).^26^ We fitted species-specific MESS using occurrence records within the EOR, and eight bioclimatic covariates thought to influence snake distribution (appendix 2 provides information about the MESS parameters and covariate specifics).

Occurrence records outside of the currently accepted EOR were overlaid on top of each species-specific threshold MESS. Records located within cells of environmental interpolation (termed MESS-positive) were considered valid records of species occurrence. Proposed ranges were developed to encompass all valid MESS-positive records, generated by applying a buffer radius of 0·898° (approximately 100 km at the equator) to each MESS-positive record to address potential movement of species, and possible geopositioning errors.^27^, ^28^ Buffered locations were masked by the threshold MESS to remove areas of environmental extrapolation, and merged with the currently accepted EOR to produce a proposed contemporary range.

### Global distribution of snakes

To reflect the geographical diversity of the snakes studied, we aggregated the ranges of different species. Modified (ie, group C species with MESS-positive records [n=96]) or original EOR surfaces (group B and group C species with no MESS-positive records [n=182]) were converted into 5 × 5 km raster (gridded) files. They were then stacked by summing overlapping cell values, resulting in three composite output layers: a count of the number of unique category one or category two species per cell, or both; a count of the number of unique category one species per cell; and a count of the number of unique category two species per cell.

### Pairing ecological measures with health system metrics

To identify the extent to which snakebites could vary globally as a public health problem, we evaluated three key dimensions: existence of any marketed antivenom therapy, quality of health care and treatment options available, and geographical accessibility to health care. Of the 278 snakes considered, the WHO antivenoms database documents that any form of antivenom (either monospecific or polyvalent) exists for 159 species.^20^ Coupling this availability information with each species' range we identified the geographical distribution of species with no listed antivenoms, stratified by WHO category.

To address differences in health-care quality and therefore identify populations to whom treatment options might not be available or effectively deployed, we categorised countries or regions to identify populations living within each decile of a composite indicator measure of health care (ie, the HAQ Index).^21^ The HAQ Index provides a metric for national levels of personal health-care access and quality, drawing from mortality rates from 32 causes that are amenable to health care. The Index uses risk-standardised cause-specific mortality rates derived from the Global Burden of Disease 2016 study,^29^ scaled to a common 0–100 value, and aggregated using weights derived from a principal component analysis. To construct deciles, countries were ranked on the basis of the HAQ Index score, and threshold values splitting countries into ten equally sized groups were identified. Because of variable numbers of administrative units, subnational locations were not used to construct decile thresholds; subnationals for which HAQ values were estimated were assigned to the corresponding nationally derived decile on the basis of their value. To evaluate the appropriateness of the HAQ Index as a proxy metric for severe snakebite-related outcomes, we analysed the relationship between published estimates of snakebite-specific mortality numbers and the index, mimicking analyses undertaken on other development indices and mortality outcomes.^6^

To reflect relative geographical isolation from health care, we coupled mortality data with a contemporary surface of accessibility to major population centres. Habib and Abubakar^30^ identified that, for a Nigerian cohort of cases, each hour delay between envenomation and antivenom administration was associated with an increased mortality outcome of 1·01% (95% CI 1·00–1·02).^30^ A contemporary surface of accessibility to high-density urban locations (travel time in minutes to locations with a population >50 000) was used to identify remote populations and compared with the mortality statistics above.^22^ To evaluate the suitability of a population–centre-based metric versus a health-care-focused measure, we did a sensitivity analysis using published data for African health-care facilities.^31^

Populations living within these geographical regions of vulnerability were enumerated using the most recent gridded population estimates from WorldPop, producing estimates at the 5 × 5 km pixel level, and aggregated to each country's second-level administrative division aiding government interpretation.^32^

### Data sharing

All codes used throughout this study are available at https://github.com/joshlongbottom/snakebite.

### Role of the funding source

The funder of the study had no role in study design, data collection, data analysis, data interpretation, or writing of the report. The corresponding authors had full access to all the data in the study and had final responsibility for the decision to submit for publication.

## Results

**Figure 2:**
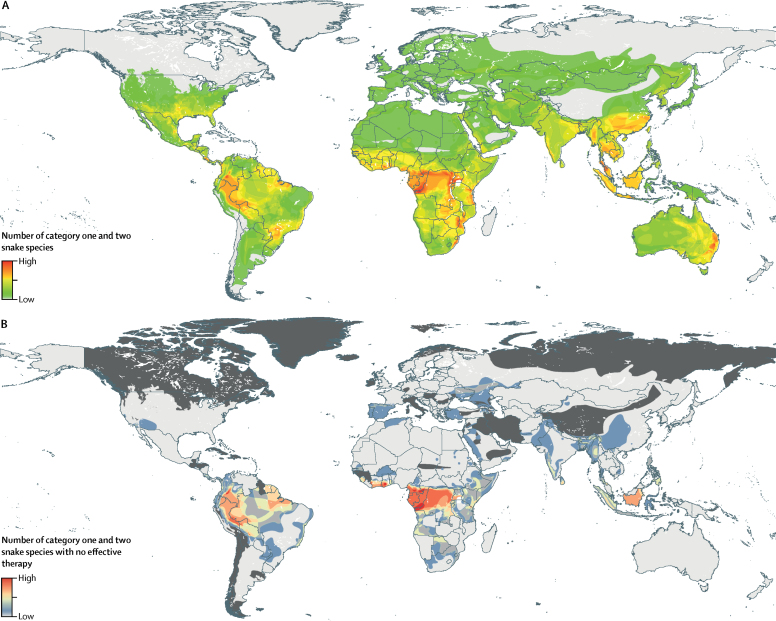
Ranges of venomous snake species and number of medically important venomous snake species per 5 × 5 km location for which no effective therapy is currently listed by WHO (A) Counts range from low (n=1) to high (n=13). The light grey areas represent locations where no medically important venomous snake species are present. (B) Counts range from low (n=1) to high (n=7). The light grey areas represent locations where snake species present have effective therapies listed by WHO, and the dark grey areas represent locations where no medically important venomous snake species are present.

Through the combination of publicly available data, we provide a surface showing the ranges of 278 snake species per 5 × 5 km area globally. Our MESS validation method resulted in range amendments of 96 species (appendix 1). Given the broad distribution of snakes, approximately 6·85 billion people live within the range of one or more of the species considered (figure 2A). When filtered by medical classification, 5·80 billion people live within range of category one species and 5·53 billion people live within range of category two species (appendix 2 pp 13, 14). Hotspots of venomous snake diversity include the Congo Basin, southeast Asia, and Latin America.

Using the only openly available database for antivenom availability, we identified 119 (43%) of the 278 mapped species with no specific therapy. Of these identified species, 24 (20%) were category one importance and 95 (80%) were category two importance. Hotspots of species with no listed antivenom occur throughout west Africa (eg, Ghana has ≤7 species per cell), central Africa (eg, Cameroon has ≤7 species per cell), South America (eg, Colombia has ≤7 species per cell), and south Asia (eg, India has ≤6 species per cell; figure 2B; appendix 2 pp 15, 16). Among category one snake species with no therapy, Myanmar and Bangladesh have the highest number (≤3 species per cell), with areas in west Africa (Mali, Senegal, and Guinea) and Namibia having up to two therapy-naive species per cell.

**Figure 3:**
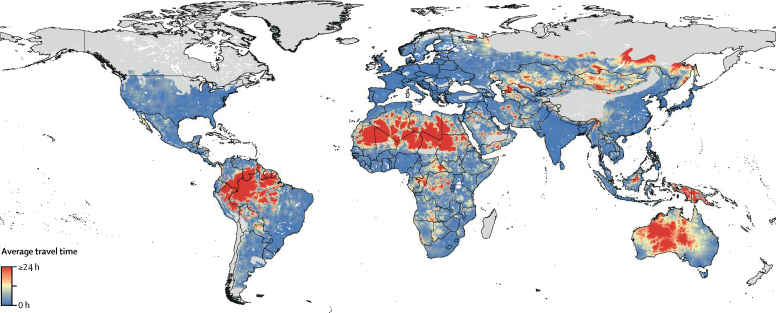
Average travel time to nearest major city for populations living within snake ranges The light grey areas represent locations without the presence of medically important venomous snake species.

Populations living within these ranges of snake species vary greatly in terms of accessibility to population centres and presumed health care. Although antivenoms are deployed in health facilities of some countries with very small communities, this deployment is not universal, and in the absence of exact data for antivenom access, we were required to approximate the influence of travelling time to health-care facilities via a proxy of distance to centres with more than 50 000 inhabitants. Our time-delay surface highlights that should envenoming occur in large areas of Sudan, Algeria, Indonesia, Papua New Guinea, Colombia, and Peru, the time taken to travel to a city in which we might expect to find available treatment could worsen mortality outcomes by more than 25%, assuming linear scaling of the statistic from Habib and Abubakar^30^ (figure 3). For instance, 2 531 665 people (78·71% of the population) live within ranges of any snake species within South Sudan and 624 204 people (89·89% of the population) in Papua New Guinea live more than 1 h from locations with 50 000 people or more (appendix 2 pp 17–26); globally, 750·19 million people (10·95% of the population) potentially at risk from snakebite envenoming live more than 1 h from high-density urban areas, increasing the likelihood of delay-based mortality outcomes after envenoming.

Sensitivity analyses using African hospitals versus cities similarly showed consistent results (appendix 2 pp 34, 35).

**Figure 4:**
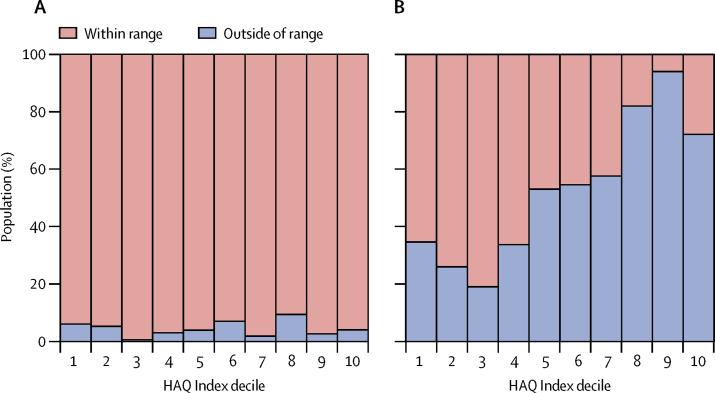
Proportion of populations living within range of snake species by each HAQ Index decile (A) Populations living within the range of one or more medically important venomous snake species (either category one or two). (B) Populations living within the range of one or more medically important venomous snake species (either category one or two), for which no effective therapy is listed. HAQ=Healthcare Access and Quality.

Separating the populations living within ranges of species by HAQ Index deciles reveals large differences across the sociodemographic spectrum (figure 4). Such differences are best highlighted when analysing populations by medical classification: approximately 389·58 million individuals (92·91%) of the population within the lowest HAQ Index decile are at risk of exposure to a category one snake compared with approximately 1·61 billion individuals (86·27%) within the highest decile (appendix 2 p 16). Furthermore, 272·91 million individuals (65·25%) of the population within the lowest decile are at risk of exposure to any snake for which no effective therapy exists compared with 519·46 million individuals (27·79%) within the highest HAQ Index decile (figure 4).

**Figure 5:**
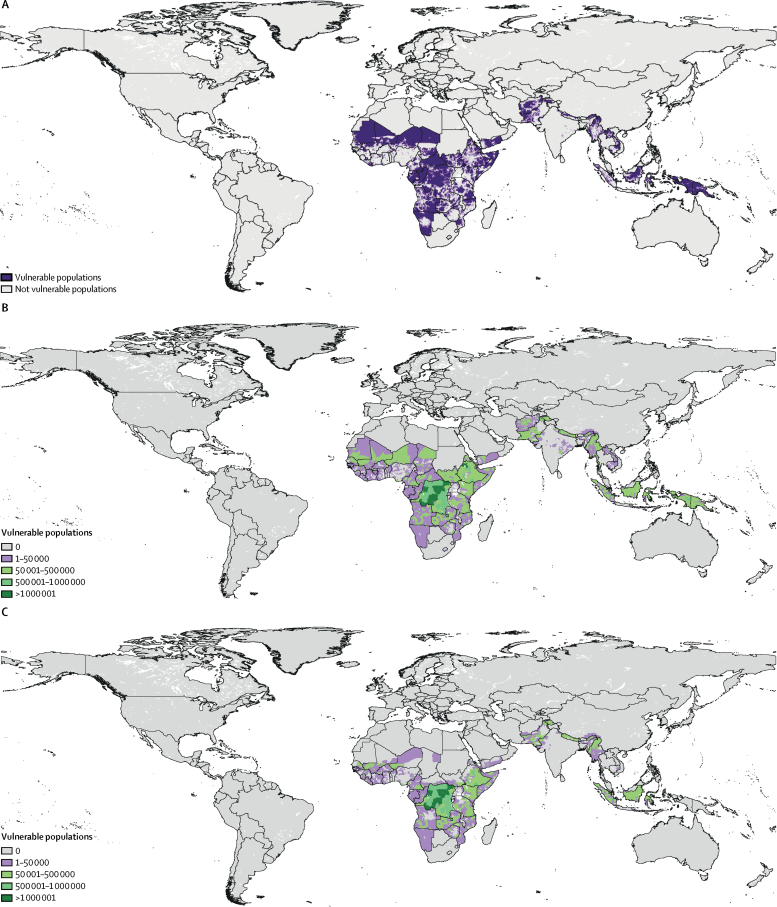
Hotspots of vulnerable populations to medically important venomous snake species Hotspots are defined as people living in areas within the range of one or more medically important venomous snake species, and more than 3 h away from major urban centres with Healthcare Access and Quality Index deciles of 1–3. (A) Pixel-level vulnerability surface (ie, vulnerability to all species of medically important snakes). (B) Aggregated second administrative level vulnerability to all species of medically important venomous snakes, as measured by the absolute number of people. (C) Aggregated second administrative level vulnerability to only those species for which no effective therapy is currently listed by WHO, as measured by the absolute number of people.

Vulnerable populations (ie, people in geographical regions living within the range of any snake species who also lived more than 3 h away from major urban centres, had health systems that scored within the lowest three deciles of the HAQ Index, and were further stratified by the presence or absence of a WHO listed antivenom) are highlighted in figure 5. Vulnerability estimates for HAQ Index deciles of 1–10 are provided in the table. Within the lowest three deciles, we highlighted regions where about 92·66 million vulnerable individuals live (table), with Angola, Pakistan, Indonesia, Ethiopia, and the Democratic Republic of the Congo ranking as the highest locations in absolute numbers. The majority of countries across Africa, many of which have some of the lowest scores on the HAQ Index, have vulnerable populations present. When excluding information about the existence of antivenoms, 146·70 million people live within remote areas lacking quality health care provisioning (deciles 1–3; appendix 2 pp 28–31).

**Table:** Vulnerable population count per HAQ Index decile

	**Decile 1**	**Decile 2**	**Decile 3**	**Decile 4**	**Decile 5**	**Decile 6**	**Decile 7**	**Decile 8**	**Decile 9**	**Decile 10**
Afghanistan	281 586	..	..	..	..	..	..	..	..	..
Algeria	..	..	..	..	74 397	..	..	..	..	..
Angola	..	3 652 123	..	..	..	..	..	..	..	..
Argentina	..	..	..	..	..	78 462	..	..	..	..
Armenia	..	..	..	..	..	..	27 064	..	..	..
Azerbaijan	..	..	..	..	..	116 150	..	..	..	..
Bangladesh	..	..	..	359 780	..	..	..	..	..	..
Belize	..	..	..	..	14 532	..	..	..	..	..
Benin	97 491	..	..	..	..	..	..	..	..	..
Bhutan	..	..	..	114 385	..	..	..	..	..	..
Bolivia	..	..	..	1 307 831	..	..	..	..	..	..
Botswana	..	..	..	323 599	..	..	..	..	..	..
Brazil	..	..	..	..	4 107 300	2296	619	..	..	..
Brunei	..	..	..	..	..	..	..	9630	..	..
Burkina Faso	699 570	..	..	..	..	..	..	..	..	..
Burundi	10 597	..	..	..	..	..	..	..	..	..
Cambodia	..	..	43 972	..	..	..	..	..	..	..
Cameroon	..	1 279 030	..	..	..	..	..	..	..	..
Central African Republic	1 081 841	..	..	..	..	..	..	..	..	..
Chad	5814	..	..	..	..	..	..	..	..	..
China	..	..	..	167 314	6 787 216	6 793 121	11 323 668	8 414 197	14 142	..
Colombia	..	..	..	..	..	6 277 835	..	..	..	..
Congo (Brazzaville)	..	521 800	..	..	..	..	..	..	..	..
Costa Rica	..	..	..	..	..	..	138 011	..	..	..
Côte d'Ivoire	379 448	..	..	..	..	..	..	..	..	..
Democratic Republic of the Congo	22 586 819	..	..	..	..	..	..	..	..	..
Djibouti	..	73 050	..	..	..	..	..	..	..	..
Ecuador	..	..	..	..	552 572	..	..	..	..	..
Egypt	..	..	..	..	37 780	..	..	..	..	..
Equatorial Guinea	..	..	..	242 345	..	..	..	..	..	..
Eritrea	905 464	..	..	..	..	..	..	..	..	..
Ethiopia	10 422 734	..	..	..	..	..	..	..	..	..
Gabon	..	..	499 707	..	..	..	..	..	..	..
Georgia	..	..	..	..	..	111 973	..	..	..	..
Ghana	..	..	354 713	..	..	..	..	..	..	..
Greece	..	..	..	..	..	..	..	..	7327	..
Guatemala	..	..	..	533 186	..	..	..	..	..	..
Guinea	427 253	..	..	..	..	..	..	..	..	..
Guinea-Bissau	120 745	..	..	..	..	..	..	..	..	..
Guyana	..	..	..	138 904	..	..	..	..	..	..
Honduras	..	..	..	58 882	..	..	..	..	..	..
India	..	231 656	1 488 560	4 623 346	..	276 547	..	..	..	..
Indonesia	..	..	10 454 226	..	..	..	..	..	..	..
Iran	..	..	..	..	..	..	341 174	..	..	..
Iraq	..	..	..	49 668	..	..	..	..	..	..
Japan	..	..	..	..	..	..	..	..	38 825	6288
Jordan	..	..	..	..	..	..	91 671	..	..	..
Kazakhstan	..	..	..	..	..	..	2 494 396	..	..	..
Kenya	..	..	1 825 765	..	..	..	..	..	..	..
Kyrgyzstan	..	..	..	..	495 765	..	..	..	..	..
Laos	..	..	18 010	..	..	..	..	..	..	..
Liberia	..	664 940	..	..	..	..	..	..	..	..
Malawi	..	133 687	..	..	..	..	..	..	..	..
Malaysia	..	..	..	..	..	1 790 903	..	..	..	..
Mali	..	2 373 844	..	..	..	..	..	..	..	..
Mauritania	..	..	5129	..	..	..	..	..	..	..
Mexico	..	..	..	..	229 259	384 600	275 250	..	..	..
Morocco	..	..	..	..	93 028	..	..	..	..	..
Mozambique	3 206 555	..	..	..	..	..	..	..	..	..
Myanmar	..	..	2 544 010	..	..	..	..	..	..	..
Namibia	..	..	752 476	..	..	..	..	..	..	..
Nepal	..	..	2 665 443	..	..	..	..	..	..	..
Nicaragua	..	..	..	..	73 046	..	..	..	..	..
Niger	15 45 113	..	..	..	..	..	..	..	..	..
Nigeria	..	..	2 067 928	..	..	..	..	..	..	..
Oman	..	..	..	..	..	..	..	84 388	..	..
Pakistan	..	..	4 425 880	..	..	..	..	..	..	..
Panama	..	..	..	..	..	137 946	..	..	..	..
Paraguay	..	..	..	..	227 331	..	..	..	..	..
Peru	..	..	..	..	..	2 674 949	..	..	..	..
Philippines	..	..	..	1 517 133	..	..	..	..	..	..
Russia	..	..	..	..	..	..	..	1 205 085	..	..
Rwanda	..	..	32 081	..	..	..	..	..	..	..
Saudi Arabia	..	..	..	..	..	..	..	2 392 280	..	..
Senegal	..	467 056	..	..	..	..	..	..	..	..
Sierra Leone	154 596	..	..	..	..	..	..	..	..	..
Somalia	2 140 834	..	..	..	..	..	..	..	..	..
South Africa	..	..	..	289 322	..	..	..	..	..	..
South Sudan	601 410	..	..	..	..	..	..	..	..	..
Sri Lanka	..	..	..	..	..	..	62 951	..	..	..
Sudan	..	..	..	542 183	..	..	..	..	..	..
Suriname	..	..	..	94 635	..	..	..	..	..	..
Swaziland	..	..	226	..	..	..	..	..	..	..
Syria	..	..	..	..	..	48 738	..	..	..	..
Tajikistan	..	..	..	301 870	..	..	..	..	..	..
Tanzania	..	4 950 775	..	..	..	..	..	..	..	..
Thailand	..	..	..	..	..	..	77 295	..	..	..
The Gambia	..	3245	..	..	..	..	..	..	..	..
Togo	..	14 488	..	..	..	..	..	..	..	..
Trinidad and Tobago	..	..	..	..	..	6520	..	..	..	..
Turkey	..	..	..	..	..	..	48 653	..	..	..
Uganda	..	371 059	..	..	..	..	..	..	..	..
Ukraine	..	..	..	..	..	..	..	4956	..	..
USA	..	..	..	..	..	..	..	..	4728	..
Uzbekistan	..	..	..	..	805 059	..	..	..	..	..
Venezuela	..	..	..	..	..	3 3465 88	..	..	..	..
Vietnam	..	..	..	..	175 519	..	..	..	..	..
Yemen	..	..	3 017 147	..	..	..	..	..	..	..
Zambia	2 125 572	..	..	..	..	..	..	..	..	..
Zimbabwe	..	936 801	..	..	..	..	..	..	..	..
Total	46 793 442	15 673 554	30 195 273	10 664 383	13 672 804	22 046 628	14 880 752	12 110 536	65 022	6288

Country-level count of vulnerable people living within the range of one or more medically important venomous snake species, for which no effective therapy exists, and with a travel time of more than 3 h from urban centres with a population of 50 000 people or more provided per HAQ Index decile (ranging from 1 [low] to 10 [high]). Appendix 2 shows the vulnerability estimates not incorporating antivenom availability. HAQ=Healthcare Access and Quality.

## Discussion

Understanding the distribution of venomous snakes and their potential burden on health systems at national, regional, and global levels is important for effective reduction and control of snakebite.^8^ By combining species' range maps, available information about antivenoms, and measures of quality of and distance to health care, this study provides global contemporary maps of vulnerable populations to snakebite and its clinical complications. This analysis, therefore, provides a means of identifying communities in greatest need of support from herpetologists, clinicians, and public health experts, and to prioritise new data-collection activities.

Although this analysis is not a substitute for a full global burden estimation, there is overlap between vulnerable communities and existing burden estimates, with vulnerable countries such as Nigeria, Benin, Congo (Brazzaville), Myanmar, and Papua New Guinea identified as burdensome in country-specific estimates,^1^ and south Asia and sub-Saharan Africa as regions with considerable mortality and morbidity.^3^ A post-hoc analysis of national envenoming and death burden values, shows that, for vulnerable countries, such values were more likely to be estimates as opposed to data-driven numbers (χ^2^ test at 90% significance level, p=0·0476 for envenoming and p=0·0517 for deaths).^3^ Chippaux^33^ similarly shows that where data are available in sub-Saharan countries, they are not necessarily contemporary information. These maps collate ecological and public health metrics, and identify opportunities where substantial improvements and refinements can be undertaken to move from broad vulnerability assessment to a more nuanced and accurate description of the most burdensome populations.

Our study had key limitations, and future efforts can focus on addressing some of these limitations: the relative contribution of different snake species must be quantified, the factors influencing snake–human interactions and subsequent likelihood of envenoming events must be identified, and snakebite-specific measures of local preparedness, effectiveness, and coverage of existing clinical countermeasures must be taken. Paucity of data available at the global scale, despite comprehensive coverage in several high-income countries, remains one of the largest limitations throughout this study. Ultimately, quantifying these additional components will allow for estimates to be based on a bottom-up data synthesis, rather than dependence on global-level datasets and correlations.

Mapping snake species' locations to reflect variations in snake presence is also important. Fine-scale maps, such as those of American venomous snake species,^34^ should be extended globally—this current analysis identifies 216 species requiring updated assessments of current ranges given the quality and quantity of records available. This study also establishes a systematic prioritisation based on medical importance (appendix 2 pp 3–11). Although species occurrence surveys can be formally done by public health initiatives or during ecological assessments, citizen science has a complementary role in facilitating broad-scale data collection.^35^

This assessment considers the presence of any one venomous snake as a prerequisite for vulnerability; however, different species contribute differently to envenoming risk. Species with a very high incidence of envenoming events might be the dominant cause of high snakebite burden in a locality,^36^ regardless of the presence of other species,^37^ as reported for *Echis ocellatus*,^38^ *Daboia russelii,*^39^ and others.^40^ Identifying and quantifying, at a local scale, important species, risky human practices, and ongoing changes to subsequent interactions given climatic and socioeconomic change, are necessary.^41^ Future vulnerability assessments can explicitly leverage interspecies' differences and weigh their relative contribution as a function of species-specific envenoming risk and associated burden. The transition of the WHO resource^20^ into a living database documenting contemporary antivenom availability, species taxonomic changes, higher-resolution distribution data, and other information will substantially aid in this effort.^42^, ^43^

Areas where snakes are present can be further evaluated to determine the true incidence of envenoming events. Local-scale household surveys assessing incidence of snakebite have been done in several countries.^11^, ^14^, ^15^, ^44^ Questions relating to snakebite could also be nested within existing demographic and health surveys,^45^ minimising associated costs and informing current data-poor estimates. By integrating preventive measures with existing management systems for neglected tropical diseases, many logistical obstacles to effective intervention might be overcome.^46^ Corresponding quantification of key risky behaviours will help reflect fine-scale population heterogeneity to exposure. Surveys such as the World Bank Living Standards Measurement Survey series could be used to obtain local-scale information about agricultural practices,^47^ further aiding the identification of communities most at risk and increasing understanding of the public health consequences of different land use. Through these steps, efforts to prevent envenoming events can be tailored to the specifics of any given population.

In many low-income and middle-income countries, a multitude of barriers influence snakebite outcomes including health care, transport, and communications infrastructure, along with adequacy of and access to safe, effective, and affordable antivenom supplies, medical staff proficiency and training, and public health policy. When considering antivenom availability, this method is constrained to listings as reported by WHO.^20^ Since initial compilation, new antivenoms have become available (eg, EchiTAb-Plus-ICP),^48^ while others have ceased production (eg, Fav-Afrique by Sanofi).^42^ Market availability of antivenom products does not translate to in-field availability and efficacy; further information regarding country-specific, contemporary stockpiles, and the positioning of antivenom holding centres is required. Given that some of the countries with the lowest HAQ Index deciles have the largest proportions of the population living in areas with snakes for which no antivenom is currently reported, documented socio-economic differences might amplify inequalities in care.^6^ Although health system indicators and accessibility metrics act as generalised correlates for a location's ability to respond to cases, these measures will possibly underestimate or overstate local vulnerabilities in some settings. Existing analyses of health systems show variation both nationally and subnationally in treatment-seeking behaviours,^49^, ^50^ quality of primary point of care visits and referrals,^51^ and general practitioner knowledge about the condition.^52^ However, the external validity of these existing surveys is unknown. This vulnerability analysis provides a foundation for the identification of locations where further surveys of treatment-seeking behaviours, quality of care, and existing coverage of antivenom stockpiles and supply chains need to be assessed.

The global burden of snakebite can be assessed through an approach that integrates ecological information, human behavioural data, and snakebite-specific health system functioning. The impetus to reduce and control the burden of snakebite envenoming, a thorough cataloguing of snake presence and abundance, species-specific interaction profiles with humans, and detailed understanding of logistical hurdles to intervention delivery should be long-term objectives. Contemporary assessments, such as the resources presented, provide an immediate means of identifying key hotspots and most vulnerable communities where the need for such investigations is greatest.
