# Supplementary material for: Vulnerability to snakebite envenoming: a global mapping of hotspots
Source: Lancet. 2018 Aug 25;392(10148):673–84. doi: 10.1016/S0140-6736(18)31224-8 (PMC6115328; doi:10.1016/S0140-6736(18)31224-8)
Supplement: Supplementary appendix 2 [file mmc2.pdf]

THE LANCET

Supplementary appendix 2

This appendix formed part of the original submission and has been peer reviewed. We post it as supplied by the authors.

Supplement to: Longbottom J, Shearer FM, Devine M, et al. Vulnerability to snakebite envenoming: a global mapping of hotspots. *Lancet* 2018; published online July 12. http://dx.doi.org/10.1016/S0140-6736(18)31224-8.

Supplementary File 1

This supplementary file (1) provides supplementary methods, figures and tables, for "Vulnerability to snakebite envenoming: a global mapping of hotspots. Joshua Longbottom, Freya M. Shearer, Maria Devine, Gabriel Alcoba, Francois Chappuis, Daniel J. Weiss, Sarah E. Ray, Nicolas Ray, Rafael Ruizde Castañeda, David J. Williams, David Warrell, Simon I. Hay & David M. Pigott."

Table of Contents:

Supplementary Figure 1: Schematic overview of the methods 2

Supplementary Table 1: Ranked species requiring range validation 3

Supplementary Figure 2: Venomous snake species ranges and their overlap based upon proposed, amended ranges 12

Supplementary Figure 3: Numbers of species with no listed antivenom split by medical importance 14

Supplementary Figure 4: Proportion of each HAQ Index decile population living within ranges of medically important snake species 15

Supplementary Figure 5: Population time-delay plots, per HAQ Index decile 16

Supplementary Figure 6: Vulnerable population hotspots 26

Supplementary Table 2: Vulnerable population count 28

Methods Supplement

Medically Important Category Descriptions and Inclusion Information 32

Supplementary Table 3: Genus inclusion list 32

Multivariate Environmental Similarity Surfaces 32

Supplementary Table 4: Covariates used to construct each Multivariate Environmental Similarity Surface 33

Supplementary Figure 7: Visualisation of MESS construction 34

Sensitivity Analysis of Travel Time Covariate 34

Health Care Access and Quality Index Sensitivity Analysis 35

Additional references 36

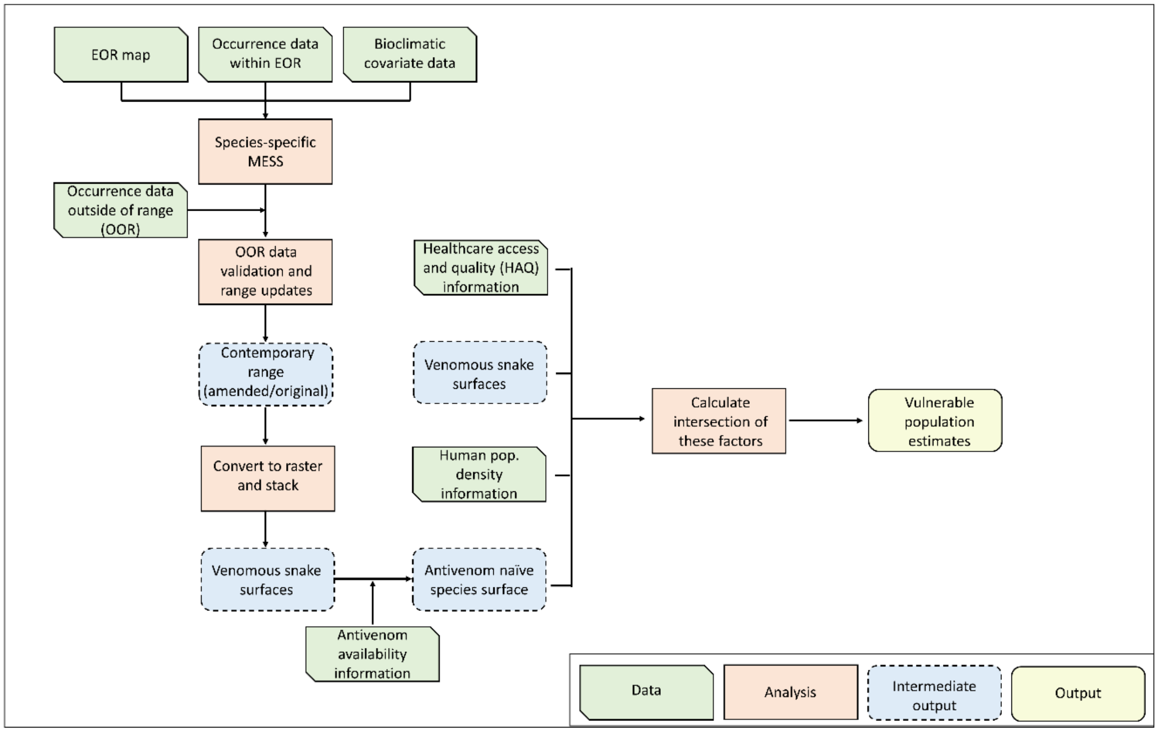

Supplementary Figure 1: Schematic overview of the methods. Overview of the methods representing input data (green, snip diagonal corner rectangle), analyses (orange, rectangle), intermediate outputs (blue, rounded rectangle, dashed) and final outputs (yellow, rounded rectangle).

Supplementary Table 1: Ranked species requiring range validation. Species requiring range validation are prioritized based on; medical category, number of out of range records, and average distance (decimal degrees) of out of range records. Species with an asterisk are species for which we provide a recommended amended range within our analysis (see Supplementary File 2 for range visualizations).

Rank	Species	No. of records	No. within range	No. out of range	Cumulative distance	Average distance	Category
1	*Crotalus viridis**	2013	799	1214	7025.996	5.787	1
2	*Crotalus atrox**	5633	4680	953	1166.113	1.224	1
3	*Pseudonaja nuchalis**	1299	507	792	5643.859	7.126	1
4	*Crotalus durissus**	937	320	617	11532.156	18.691	1
5	*Bothrops cf. atrox*	354	23	331	3997.329	12.077	1
6	*Bothrops asper**	1397	1081	316	1120.957	3.547	1
7	*Notechis scutatus**	1369	1120	249	642.232	2.579	1
8	*Crotalus oreganus**	3684	3436	248	248.010	1.000	1
9	*Agkistrodon contortrix**	4080	3836	244	762.361	3.124	1
10	*Pseudonaja affinis**	477	262	215	554.312	2.578	1
11	*Agkistrodon piscivorus**	2722	2518	204	289.690	1.420	1
12	*Crotalus scutulatus**	2865	2675	190	295.161	1.553	1
13	*Bothrops atrox**	464	278	186	1208.329	6.496	1
14	*Crotalus horridus**	1588	1409	179	1133.830	6.334	1
15	*Vipera aspis**	1595	1462	133	194.315	1.461	1
16	*Pseudonaja textilis**	3488	3371	117	349.519	2.987	1
17	*Pseudechis australis**	1990	1877	113	164.741	1.458	1
18	*Dendroaspis viridis**	153	59	94	101.653	1.081	1
19	*Bitis nasicornis**	154	66	88	189.360	2.152	1
20	*Agkistrodon bilineatus**	235	149	86	479.110	5.571	1
21	*Echis carinatus**	124	41	83	1797.895	21.661	1

Rank	Species	No. of records	No. within range	No. out of range	Cumulative distance	Average distance	Category
22	*Bitis gabonica**	201	119	82	307.339	3.748	1
23	*Dendroaspis jamesoni**	181	99	82	210.929	2.572	1
24	*Naja nigricollis**	356	274	82	208.688	2.545	1
25	*Pseudonaja mengdeni**	543	465	78	96.552	1.238	1
26	*Naja naja*	94	22	72	2399.227	33.323	1
27	*Bitis arietans**	920	858	62	220.748	3.560	1
28	*Crotalus adamanteus**	915	860	55	551.571	10.029	1
29	*Daboia russelii**	64	18	46	293.668	6.384	1
30	*Crotalus simus**	73	28	45	586.513	13.034	1
31	*Echis ocellatus**	100	62	38	42.613	1.121	1
32	*Bothrops moojeni**	81	46	35	37.224	1.064	1
33	*Pseudocerastes persicus*	36	2	34	283.047	8.325	1
34	*Oxyuranus scutellatus**	342	308	34	144.121	4.239	1
35	*Lachesis muta**	85	52	33	191.753	5.811	1
36	*Cerastes cerastes**	125	94	31	266.664	8.602	1
37	*Naja melanoleuca**	385	355	30	75.944	2.531	1
38	*Echis coloratus**	148	123	25	122.661	4.906	1
39	*Echis pyramidum*	29	6	23	145.048	6.306	1
40	*Dendroaspis angusticeps**	98	75	23	81.791	3.556	1
41	*Daboia palaestinae**	252	230	22	4.583	0.208	1
42	*Bothrops alternatus**	105	84	21	145.597	6.933	1
43	*Vipera ammodytes*	51	32	19	276.191	14.536	1
44	*Naja haje*	28	9	19	164.959	8.682	1
45	*Bothrops jararaca**	473	454	19	70.280	3.699	1
46	*Crotalus totonacus*	24	5	19	13.611	0.716	1
47	*Naja mossambica**	196	180	16	65.749	4.109	1
48	*Vipera berus**	189	174	15	203.627	13.575	1

Rank	Species	No. of records	No. within range	No. out of range	Cumulative distance	Average distance	Category
49	*Dendroaspis polylepis**	153	139	14	18.691	1.335	1
50	*Acanthophis laevis**	85	72	13	167.606	12.893	1
51	*Gloydius halys*	27	15	12	117.594	9.799	1
52	*Bothrops jararacussu**	176	164	12	47.823	3.985	1
53	*Naja annulifera*	106	95	11	50.441	4.586	1
54	*Protobothrops mucrosquamatus**	50	39	11	11.351	1.032	1
55	*Bothrops brazili**	39	29	10	0.616	0.062	1
56	*Bitis rhinoceros*	19	10	9	4.686	0.521	1
57	*Bungarus multicinctus*	84	76	8	18.351	2.294	1
58	*Naja sumatrana*	25	18	7	58.700	8.386	1
59	*Bungarus magnimaculatus*	20	13	7	20.687	2.955	1
60	*Naja sputatrix*	12	6	6	255.749	42.625	1
61	*Naja kaouthia*	41	35	6	168.148	28.025	1
62	*Gloydius blomhoffi*	9	4	5	369.991	73.998	1
63	*Bothrops leucurus*	93	89	4	21.150	5.287	1
64	*Naja nivea**	106	102	4	6.880	1.720	1
65	*Bungarus caeruleus*	17	13	4	6.804	1.701	1
66	*Bungarus niger*	4	0	4	5.593	1.398	1
67	*Cryptelytrops albolabris*	8	4	4	1.691	0.423	1
68	*Hypnale hypnale*	12	8	4	0.627	0.157	1
69	*Bothrops lanceolatus*	3	0	3	125.713	41.904	1
70	*Bungarus candidus*	27	24	3	110.796	36.932	1
71	*Calloselasma rhodostoma*	28	25	3	99.803	33.268	1
72	*Daboia siamensis*	5	2	3	96.838	32.279	1
73	*Deinagkistrodon acutus*	14	11	3	12.461	4.154	1
74	*Naja philippinensis*	18	15	3	11.351	3.784	1
75	*Bothrops diporus*	13	10	3	6.737	2.246	1

Rank	Species	No. of records	No. within range	No. out of range	Cumulative distance	Average distance	Category
76	*Bothrops pictus*	3	0	3	1.498	0.499	1
77	*Naja anchietae*	5	2	3	1.388	0.463	1
78	*Cerastes gasperettii*	26	23	3	0.847	0.282	1
79	*Protobothrops flavoviridis*	17	14	3	0.006	0.002	1
80	*Naja ashei*	13	11	2	1.405	0.703	1
81	*Echis leucogaster*	16	14	2	1.068	0.534	1
82	*Macrovipera lebetina*	13	11	2	0.603	0.301	1
83	*Gloydius brevicaudus*	17	15	2	0.388	0.194	1
84	*Montivipera xanthina*	3	2	1	10.181	10.181	1
85	*Bothrops bilineatus*	15	14	1	9.419	9.419	1
86	*Bothrops mattogrossensis*	4	3	1	1.439	1.439	1
87	*Naja katiensis*	1	0	1	1.329	1.329	1
88	*Naja nigricincta*	6	5	1	1.045	1.045	1
89	*Naja oxiana*	4	3	1	0.864	0.864	1
90	*Naja senegalensis*	3	2	1	0.363	0.363	1
91	*Agkistrodon taylori*	1	0	1	0.214	0.214	1
92	*Micrurus fulvius**	1087	746	341	3929.429	11.523	2
93	*Acanthophis antarcticus**	898	592	306	2598.917	8.493	2
94	*Sistrurus catenatus**	1575	1284	291	13524.864	46.477	2
95	*Pseudechis porphyriacus**	3791	3514	277	773.798	2.793	2
96	*Crotalus ruber**	1738	1513	225	190.111	0.845	2
97	*Micrurus nigrocinctus**	423	223	200	139.372	0.697	2
98	*Cerrophidion godmani**	264	66	198	527.707	2.665	2
99	*Crotalus ornatus**	491	311	180	197.131	1.095	2
100	*Thelotornis kirtlandii**	197	43	154	445.913	2.896	2
101	*Atropoides nummifer**	209	56	153	730.048	4.772	2
102	*Crotalus basiliscus**	563	416	147	217.128	1.477	2

Rank	Species	No. of records	No. within range	No. out of range	Cumulative distance	Average distance	Category
103	*Crotalus molossus**	1606	1466	140	963.762	6.884	2
104	*Pseudonaja aspidorhyncha**	240	128	112	320.310	2.860	2
105	*Atractaspis bibronii**	236	125	111	194.708	1.754	2
106	*Austrelaps ramsayi**	526	429	97	34.181	0.352	2
107	*Ophryacus undulatus*	93	2	91	141.350	1.553	2
108	*Acanthophis praelongus**	163	73	90	696.211	7.736	2
109	*Sistrurus miliarius**	1036	951	85	649.916	7.646	2
110	*Austrelaps superbus**	679	597	82	339.842	4.144	2
111	*Porthidium dunni**	162	83	79	95.881	1.214	2
112	*Bothriechis schlegelii**	357	292	65	8.200	0.126	2
113	*Micrurus mipartitus**	211	148	63	246.455	3.912	2
114	*Micrurus lemniscatus**	141	79	62	102.369	1.651	2
115	*Tropidechis carinatus**	338	280	58	44.115	0.761	2
116	*Pseudonaja inframacula**	275	217	58	24.373	0.420	2
117	*Bothrops neuwiedi*	96	45	51	283.124	5.551	2
118	*Thelotornis capensis**	200	151	49	297.657	6.075	2
119	*Vipera latastei**	685	638	47	7.496	0.159	2
120	*Hemachatus haemachatus**	156	112	44	45.985	1.045	2
121	*Hoplocephalus bungaroides**	143	104	39	83.530	2.142	2
122	*Atractaspis irregularis**	77	40	37	33.884	0.916	2
123	*Vipera seoanei**	402	366	36	12.803	0.356	2
124	*Porthidium nasutum**	210	178	32	13.112	0.410	2
125	*Porthidium ophryomegas*	100	71	29	187.242	6.457	2
126	*Acanthophis wellsi**	78	52	26	7.959	0.306	2
127	*Rhabdophis tigrinus**	196	173	23	560.331	24.362	2
128	*Micrurus tener**	577	556	21	423.563	20.170	2
129	*Micrurus spixii*	59	38	21	27.296	1.300	2

Rank	Species	No. of records	No. within range	No. out of range	Cumulative distance	Average distance	Category
130	*Pseudonaja guttata**	214	194	20	29.863	1.493	2
131	*Pseudechis guttatus**	349	329	20	5.114	0.256	2
132	*Pseudonaja ingrami**	56	36	20	2.558	0.128	2
133	*Porthidium lansbergii*	41	22	19	2.401	0.126	2
134	*Bungarus fasciatus**	85	67	18	93.368	5.187	2
135	*Walterinnesia aegyptia**	73	56	17	118.610	6.977	2
136	*Atropoides mexicanus*	34	18	16	40.100	2.506	2
137	*Ophiophagus hannah**	68	53	15	390.090	26.006	2
138	*Hoplocephalus bitorquatus**	349	334	15	151.054	10.070	2
139	*Acanthophis rugosus*	15	1	14	166.523	11.895	2
140	*Hoplocephalus stephensii**	370	356	14	161.122	11.509	2
141	*Austrelaps labialis**	224	210	14	0.049	0.003	2
142	*Oxyuranus microlepidotus*	69	56	13	19.789	1.522	2
143	*Cryptelytrops macrops*	16	3	13	12.219	0.940	2
144	*Acanthophis pyrrhus**	196	183	13	9.946	0.765	2
145	*Bothriechis bicolor*	39	26	13	6.099	0.469	2
146	*Trimeresurus gramineus*	15	3	12	548.605	45.717	2
147	*Pseudechis butleri*	31	19	12	2.572	0.214	2
148	*Micruroides euryxanthus**	260	249	11	256.304	23.300	2
149	*Calliophis intestinalis**	81	70	11	192.840	17.531	2
150	*Porthidium yucatanicum*	90	79	11	8.887	0.808	2
151	*Cerrophidion tzotzilorum*	101	90	11	4.283	0.389	2
152	*Bothrops ammodytoides*	30	20	10	482.480	48.248	2
153	*Bothriechis aurifer*	15	5	10	3.497	0.350	2
154	*Vipera ursinii*	9	0	9	109.176	12.131	2
155	*Micrurus corallinus*	293	284	9	87.932	9.770	2
156	*Dispholidus typus*	83	74	9	10.243	1.138	2

Rank	Species	No. of records	No. within range	No. out of range	Cumulative distance	Average distance	Category
157	*Atropoides olmec**	41	32	9	0.898	0.100	2
158	*Pseudechis colletti*	74	66	8	34.386	4.298	2
159	*Thelotornis usambaricus*	17	9	8	17.844	2.231	2
160	*Tropidolaemus subannulatus**	57	49	8	11.027	1.378	2
161	*Bothrocophias hyoprora*	30	22	8	7.366	0.921	2
162	*Bothrops andianus*	9	1	8	4.017	0.502	2
163	*Bothriechis rowleyi*	10	2	8	0.863	0.108	2
164	*Bothriechis lateralis*	63	56	7	164.995	23.571	2
165	*Lachesis melanocephala*	15	8	7	58.256	8.322	2
166	*Thelotornis mossambicanus*	15	9	6	4.137	0.689	2
167	*Micropechis ikaheka*	101	95	6	0.029	0.005	2
168	*Eristocophis macmahoni*	7	2	5	163.237	32.647	2
169	*Micrurus surinamensis*	41	36	5	3.120	0.624	2
170	*Naja nubiae*	8	4	4	64.174	16.043	2
171	*Bothrops pubescens*	6	2	4	62.980	15.745	2
172	*Atheris squamigera**	46	42	4	19.718	4.929	2
173	*Bothrops barnetti*	6	2	4	8.015	2.004	2
174	*Pseudechis papuanus*	24	20	4	6.232	1.558	2
175	*Crotalus tzabcan*	26	22	4	1.132	0.283	2
176	*Bothriechis nigroviridis*	28	24	4	0.193	0.048	2
177	*Cerrophidion barbouri*	19	15	4	0.140	0.035	2
178	*Trimeresurus malabaricus*	12	8	4	0.118	0.029	2
179	*Pseudonaja tanneri*	3	0	3	8.248	2.749	2
180	*Lachesis stenophrys*	16	13	3	7.657	2.552	2
181	*Vipera renardi*	8	5	3	3.706	1.235	2
182	*Atropoides indomitus*	7	4	3	1.979	0.660	2
183	*Ophryacus melanurus*	3	0	3	1.566	0.522	2

Rank	Species	No. of records	No. within range	No. out of range	Cumulative distance	Average distance	Category
184	*Bothrocophias myersi*	4	1	3	1.500	0.500	2
185	*Vipera kaznakovi*	5	2	3	0.333	0.111	2
186	*Bothriechis marchi*	12	9	3	0.179	0.060	2
187	*Gloydius intermedius*	8	6	2	96.445	48.222	2
188	*Oxyuranus temporalis*	4	2	2	5.423	2.712	2
189	*Protobothrops jerdonii*	44	42	2	4.576	2.288	2
190	*Pseudohaje nigra*	5	3	2	2.799	1.400	2
191	*Atropoides occiduus*	2	0	2	1.953	0.976	2
192	*Bothrocophias microphthalmus*	9	7	2	1.139	0.569	2
193	*Lachesis acrochorda*	5	3	2	0.758	0.379	2
194	*Pseudohaje goldii*	13	11	2	0.687	0.344	2
195	*Bothriechis thalassinus*	5	3	2	0.392	0.196	2
196	*Bitis parviocula*	2	0	2	0.248	0.124	2
197	*Naja annulata*	5	3	2	0.186	0.093	2
198	*Atheris chlorechis*	20	18	2	0.106	0.053	2
199	*Atropoides picadoi*	9	7	2	0.086	0.043	2
200	*Bungarus flaviceps*	6	5	1	95.347	95.347	2
201	*Trimeresurus trigonocephalus*	13	12	1	80.112	80.112	2
202	*Naja pallida*	33	32	1	36.939	36.939	2
203	*Vipera nikolskii*	1	0	1	16.512	16.512	2
204	*Bothrops sanctaecrucis*	5	4	1	6.041	6.041	2
205	*Tropidolaemus philippensis*	4	3	1	2.264	2.264	2
206	*Montivipera raddei*	10	9	1	0.556	0.556	2
207	*Atractaspis engaddensis*	27	26	1	0.438	0.438	2
208	*Bothrops osbornei*	1	0	1	0.388	0.388	2
209	*Bothrocophias colombianus*	1	0	1	0.240	0.240	2
210	*Gloydius halys caucasicus*	2	1	1	0.169	0.169	2

Rank	Species	No. of records	No. within range	No. out of range	Cumulative distance	Average distance	Category
211	*Echis hughesi*	1	0	1	0.136	0.136	2
212	*Pseudechis rossignolii*	1	0	1	0.085	0.085	2
213	*Proatheris superciliaris*	2	1	1	0.076	0.076	2
214	*Pseudechis weigeli*	4	3	1	0.067	0.067	2
215	*Porthidium hespere*	1	0	1	0.023	0.023	2
216	*Trimeresurus hageni*	12	11	1	0.002	0.002	2

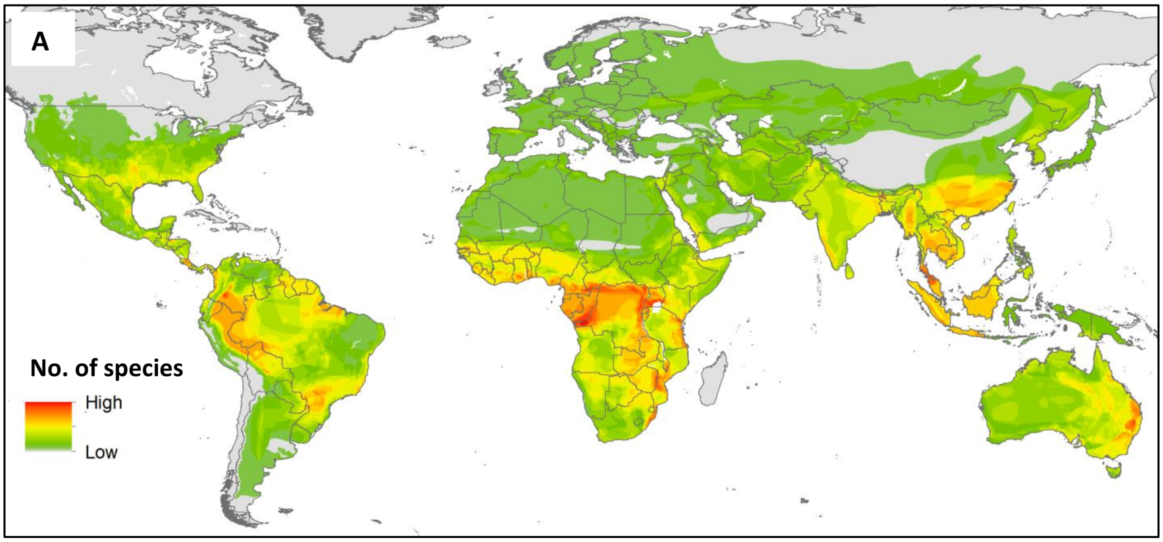
A
No. of species
High
Low

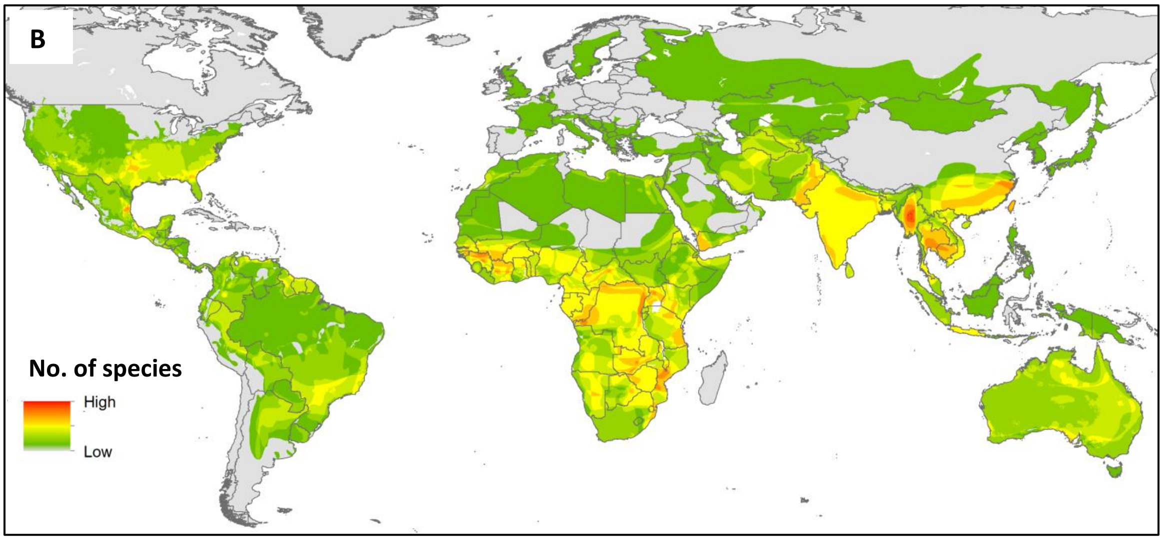
B
No. of species
High
Low

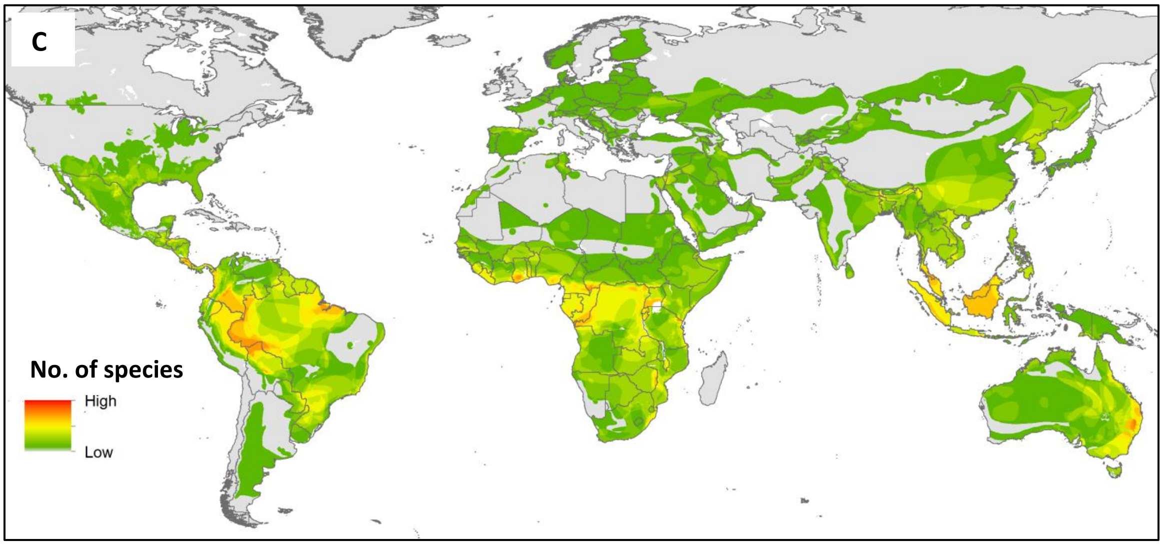
C
No. of species
High
Low

Figure 2. Venomous snake species ranges and their overlap based upon proposed, amended ranges. A: Categories one and two venomous snake species count ranging from low (1) to high (13); B: Category one venomous snake species count ranging from low (1) to high (8); C: Category two venomous snake species count ranging from low (1) to high (11). Grey represents locations where no venomous snakes within the different aggregations are to be found.

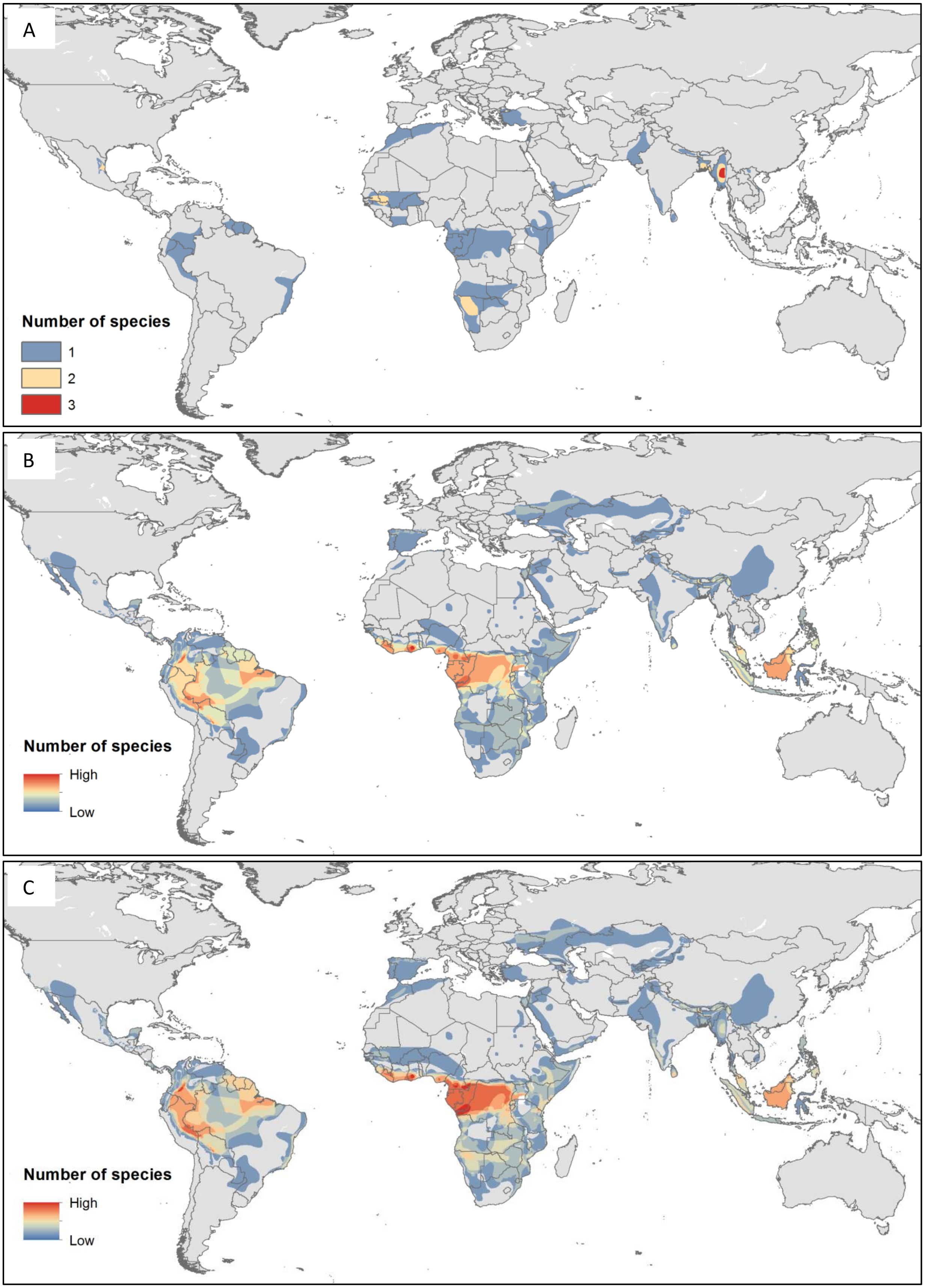
A
Number of species
1
2
3
B
Number of species
High
Low
C
Number of species
High
Low

Supplementary Figure 3: Numbers of species with no listed antivenom split by medical importance. Each panel represents the number of venomous snake species per 5 x 5km cell for which no species-specific antivenom exists. Panel A: Category one medically important species; Panel B: Category two medically important species, ranging from low (blue, 1 species) to high (red, 7 species); Panel C. Both category one and category two medically important species, ranging from low (blue, 1 species), to high (red, 7 species).

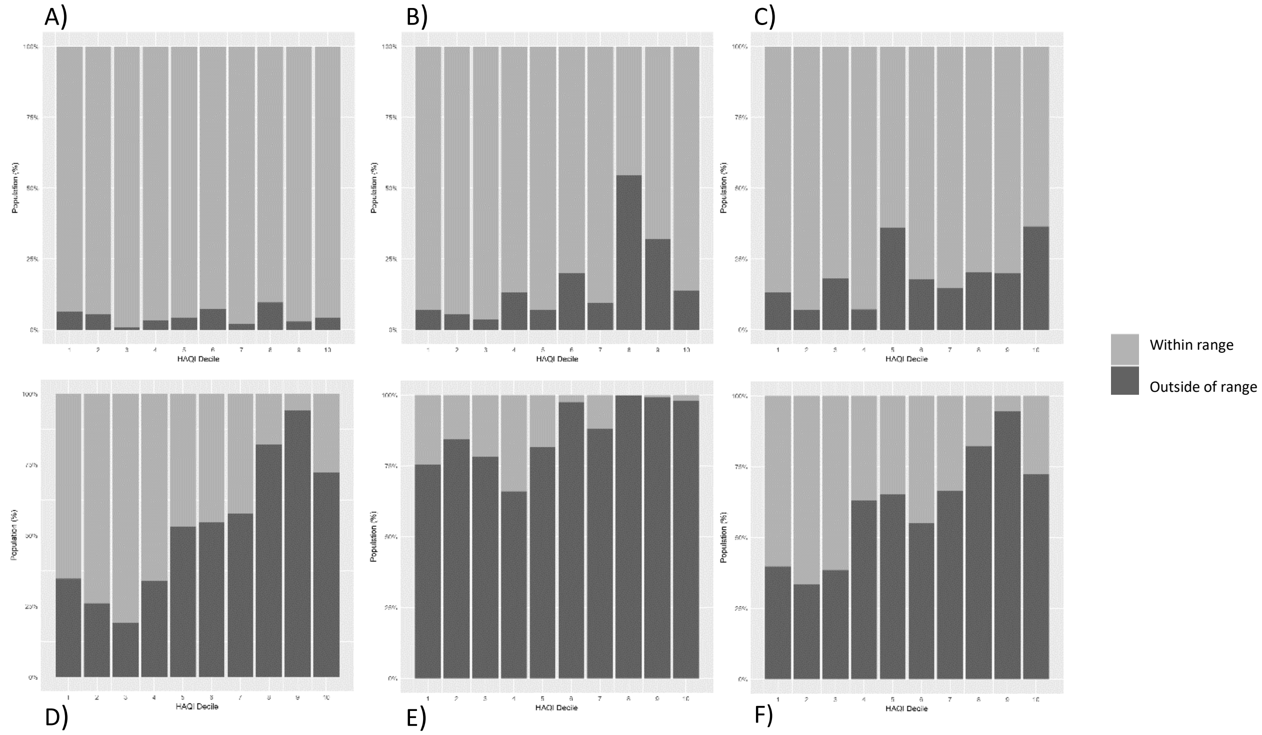

Supplementary Figure 4: Proportion of each HAQ Index decile population living within ranges of medically important snake species. A) One or more VSSMI (either category); B) One or more Category one VSSMI; C) One or more Category two VSSMI; D) One or more VSSMI lacking listed antivenoms (either category); E) One or more Category one VSSMI lacking listed antivenoms; F) One or more Category two VSSMI lacking listed antivenoms.

HAQ Index, Decile 1.

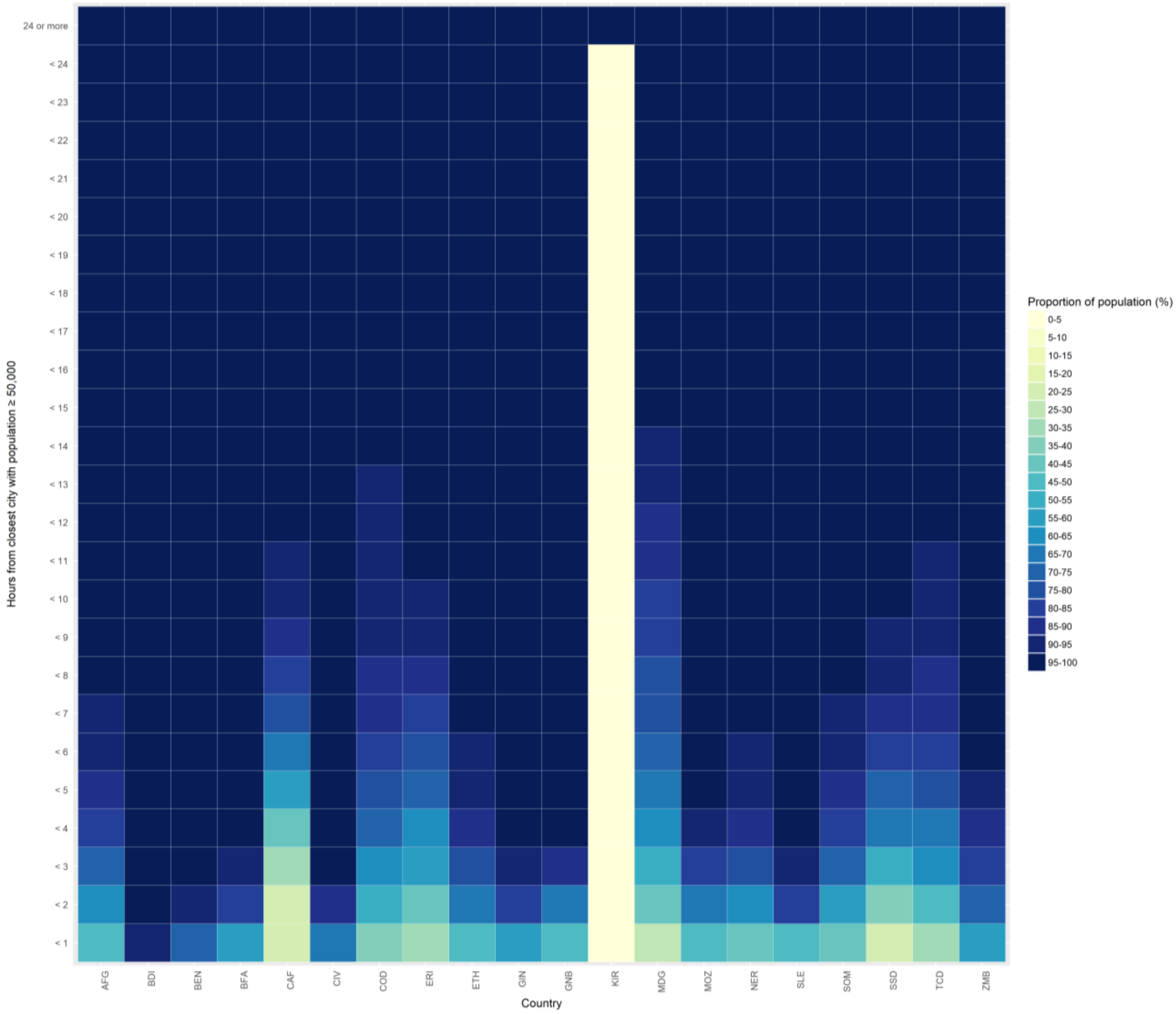

HAQ Index, Decile 2.

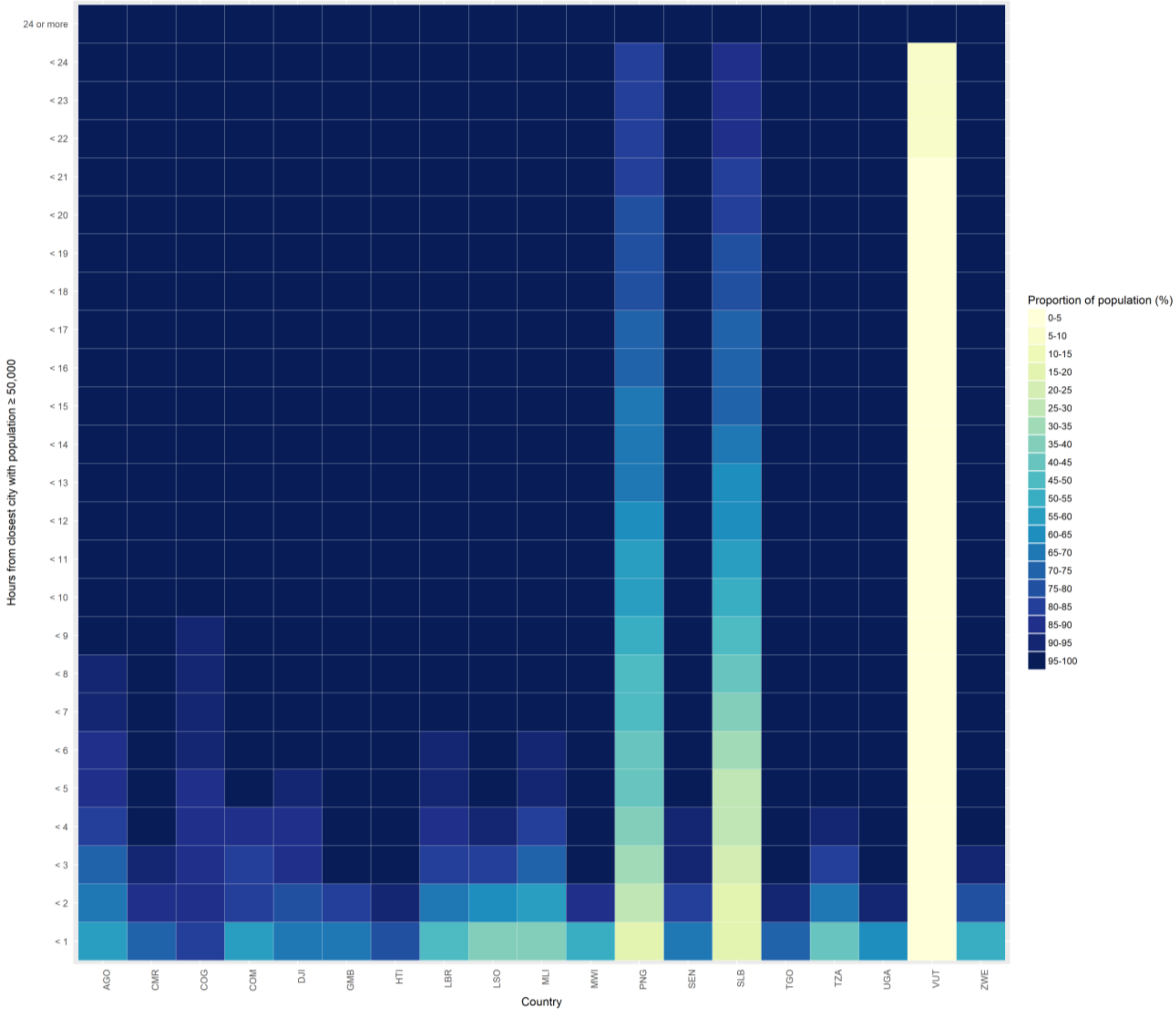

HAQ Index, Decile 3.

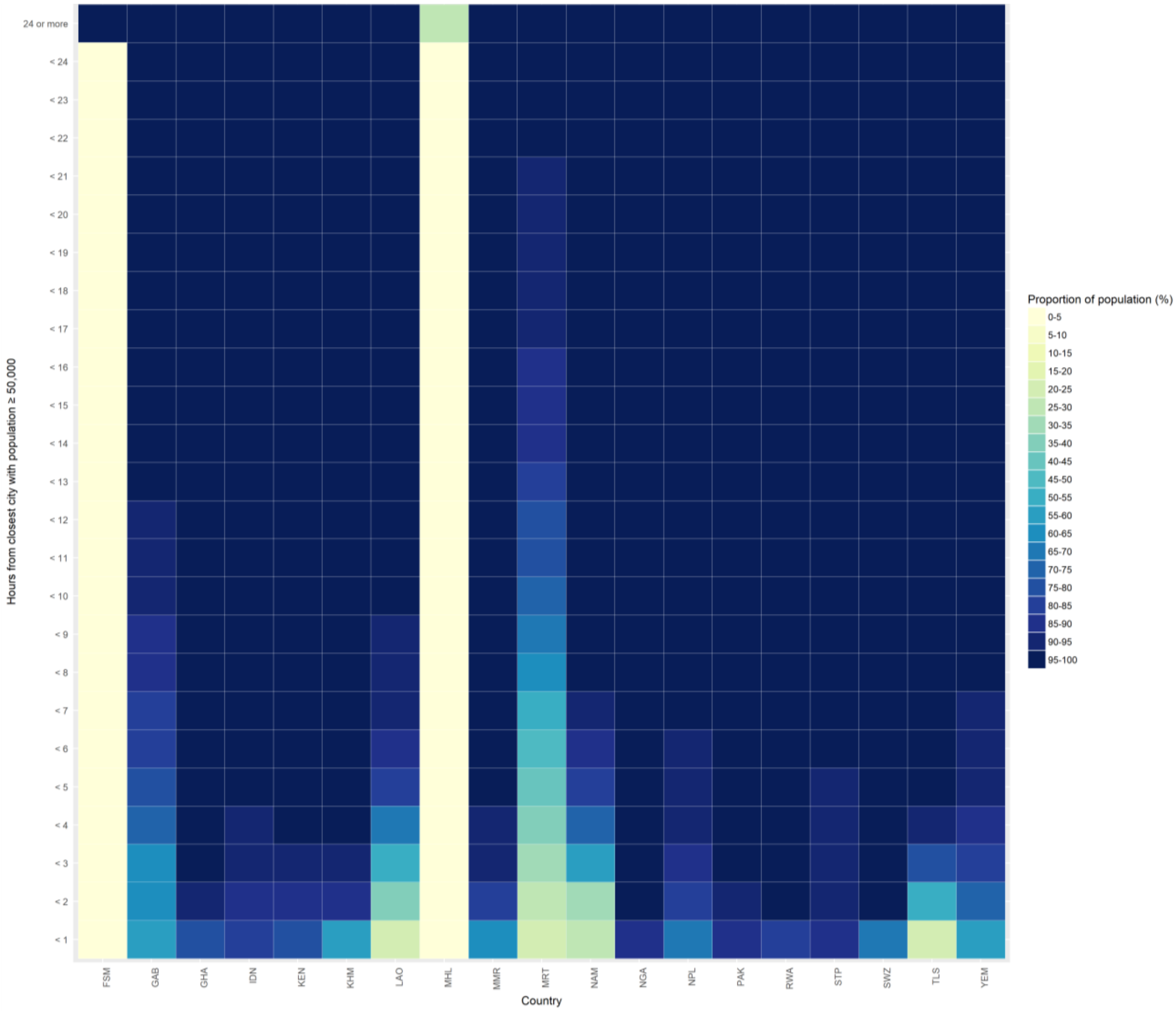

HAQ Index, Decile 4.

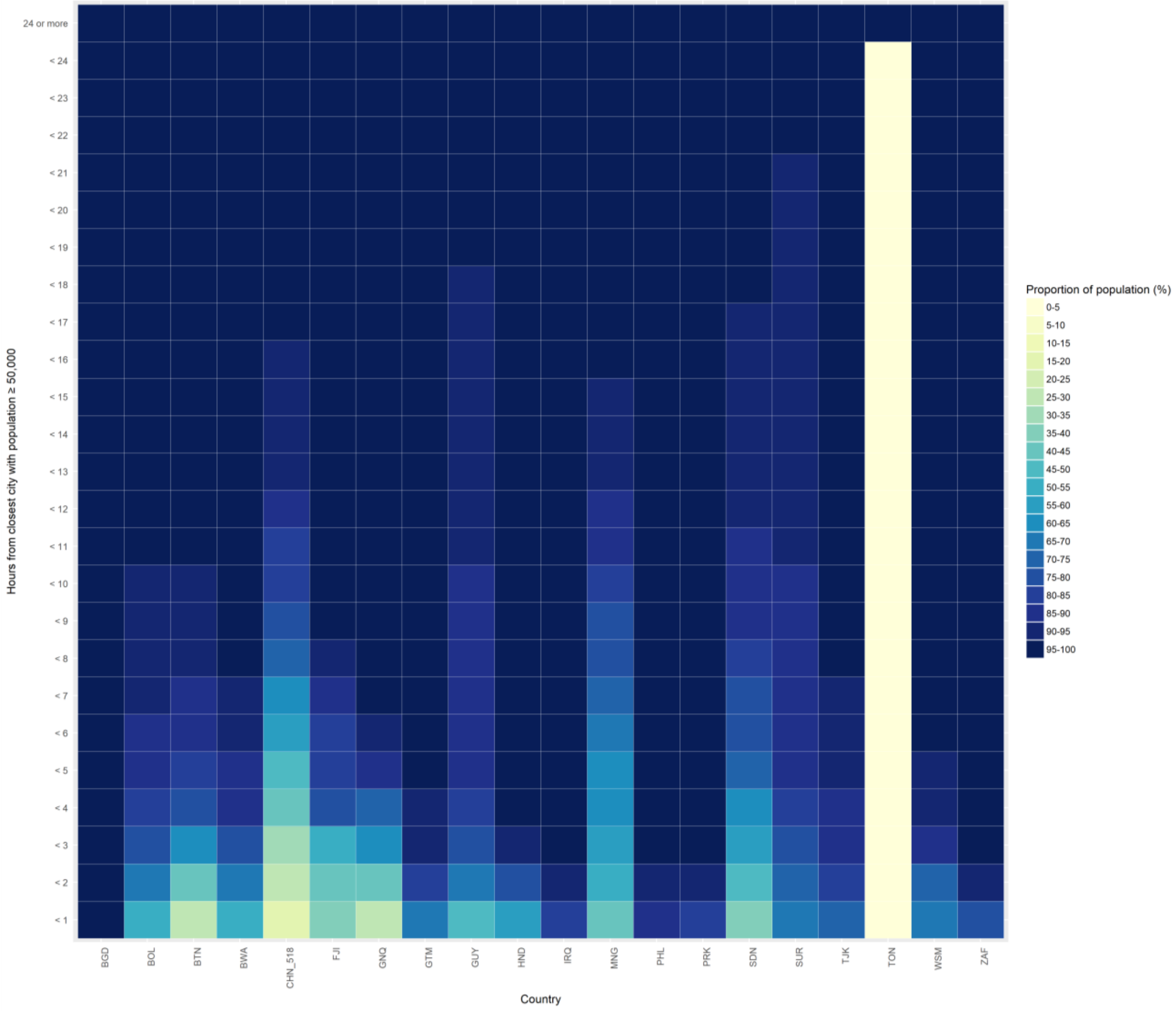

HAQ Index, Decile 5.

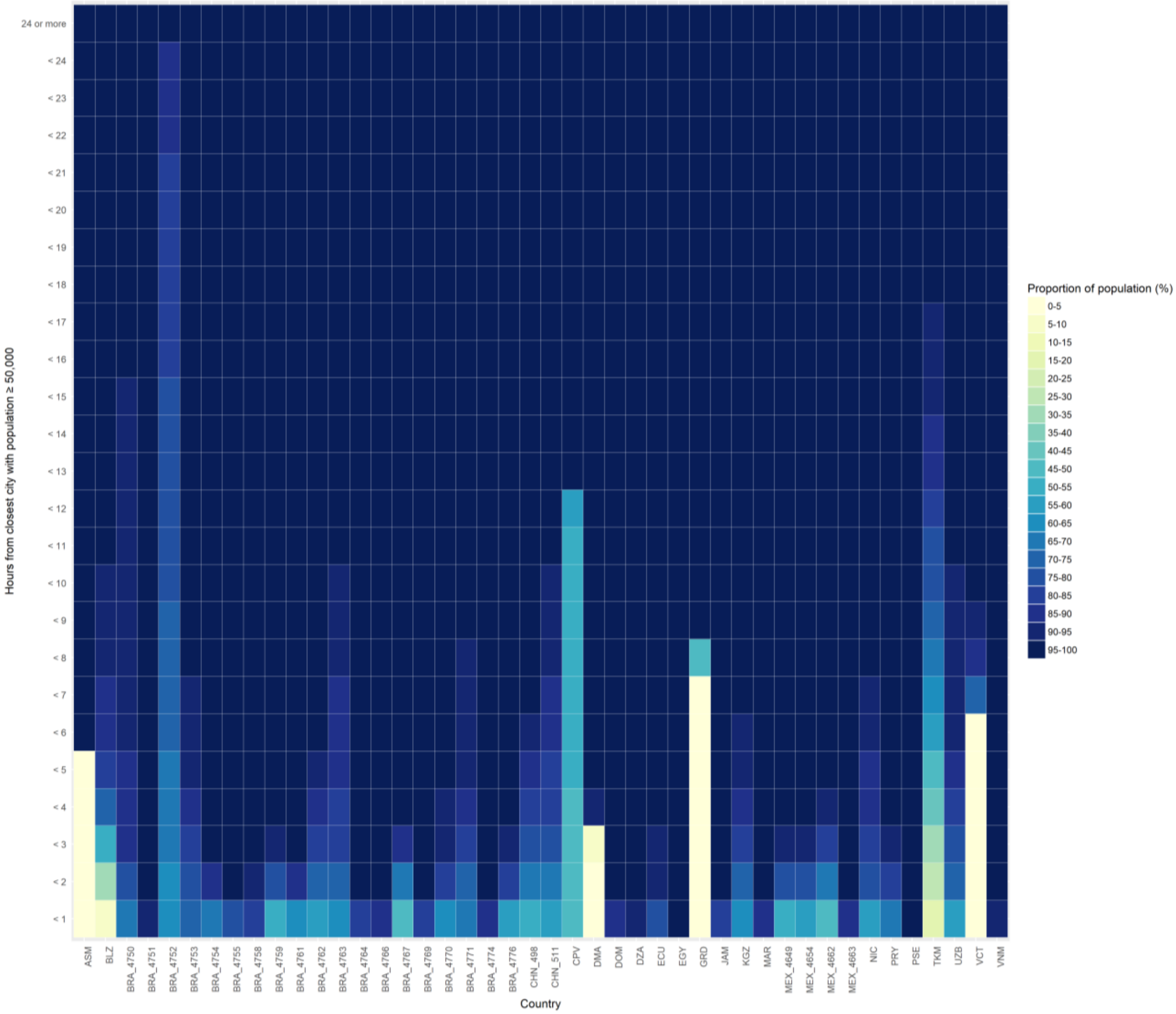

HAQ Index, Decile 6.

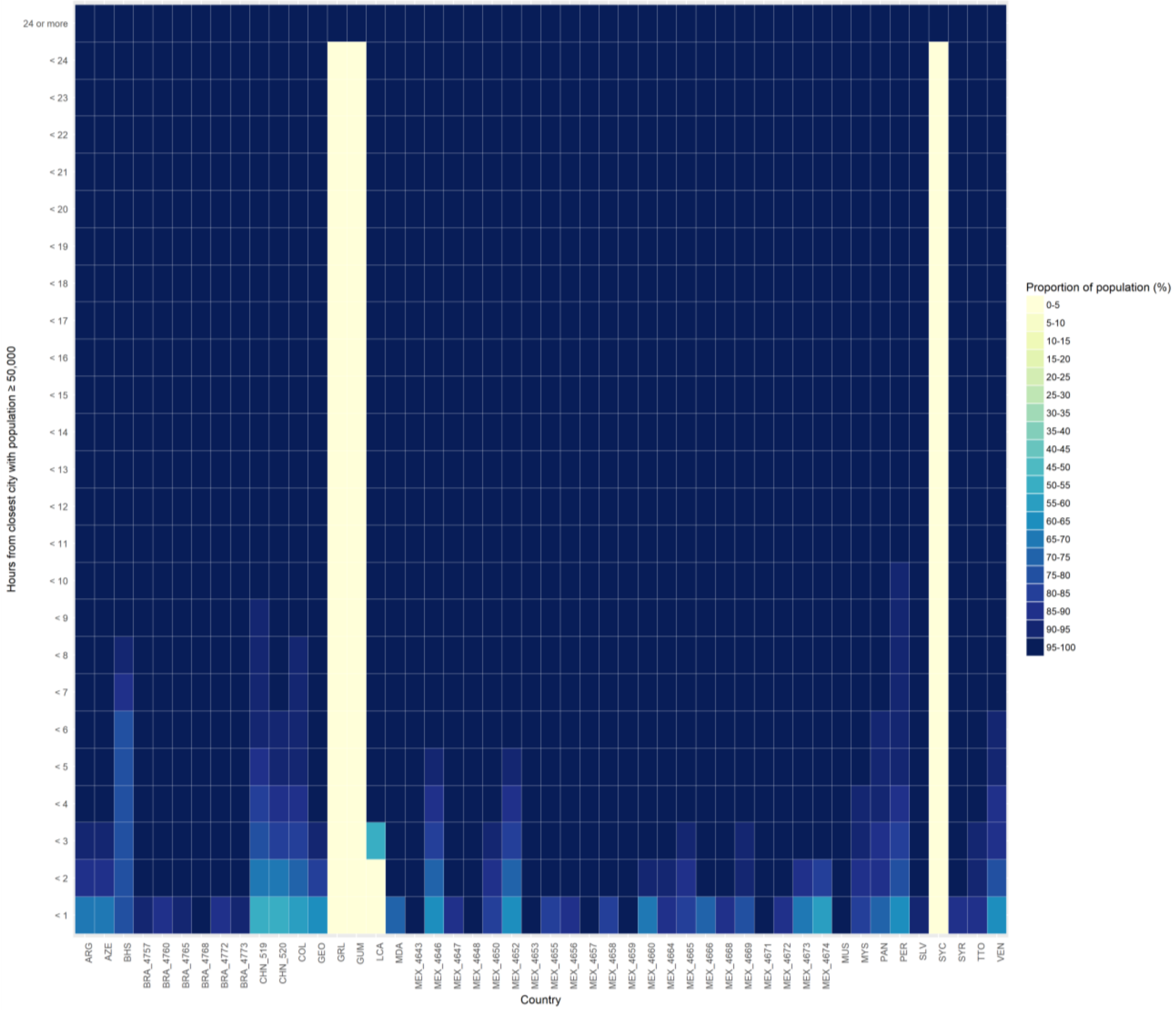

HAQ Index, Decile 7.

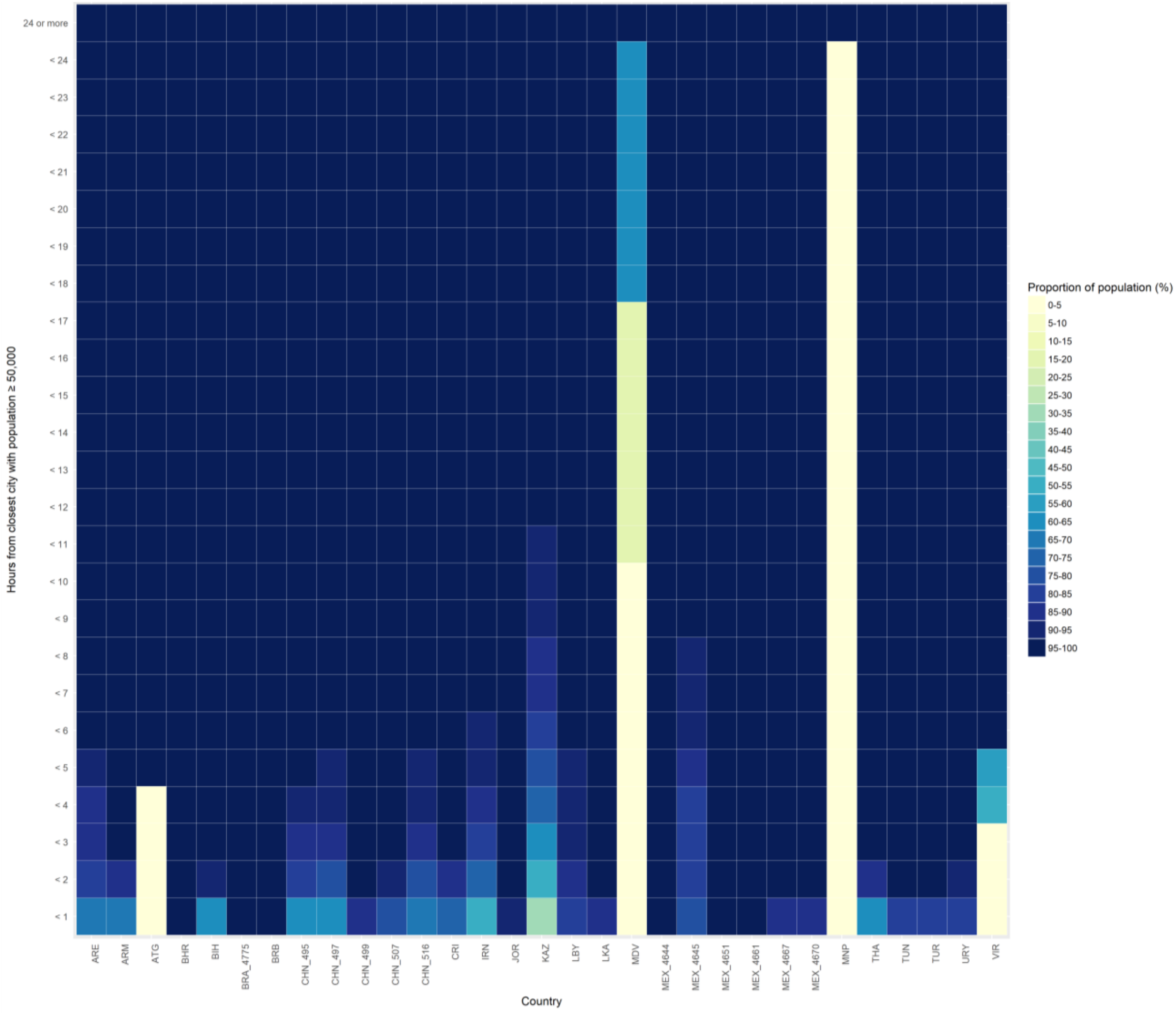

HAQ Index, Decile 8.

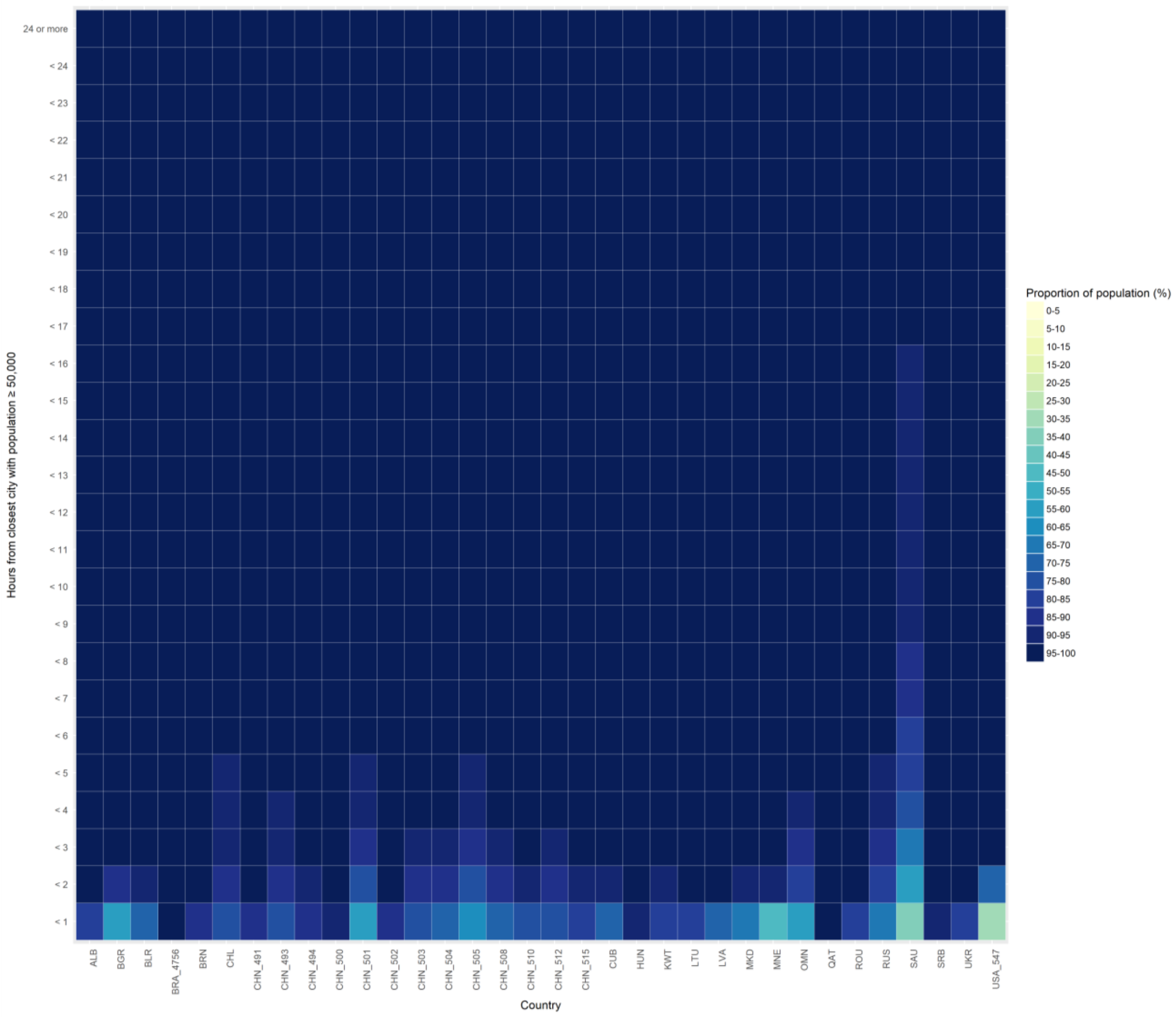

HAQ Index, Decile 9.

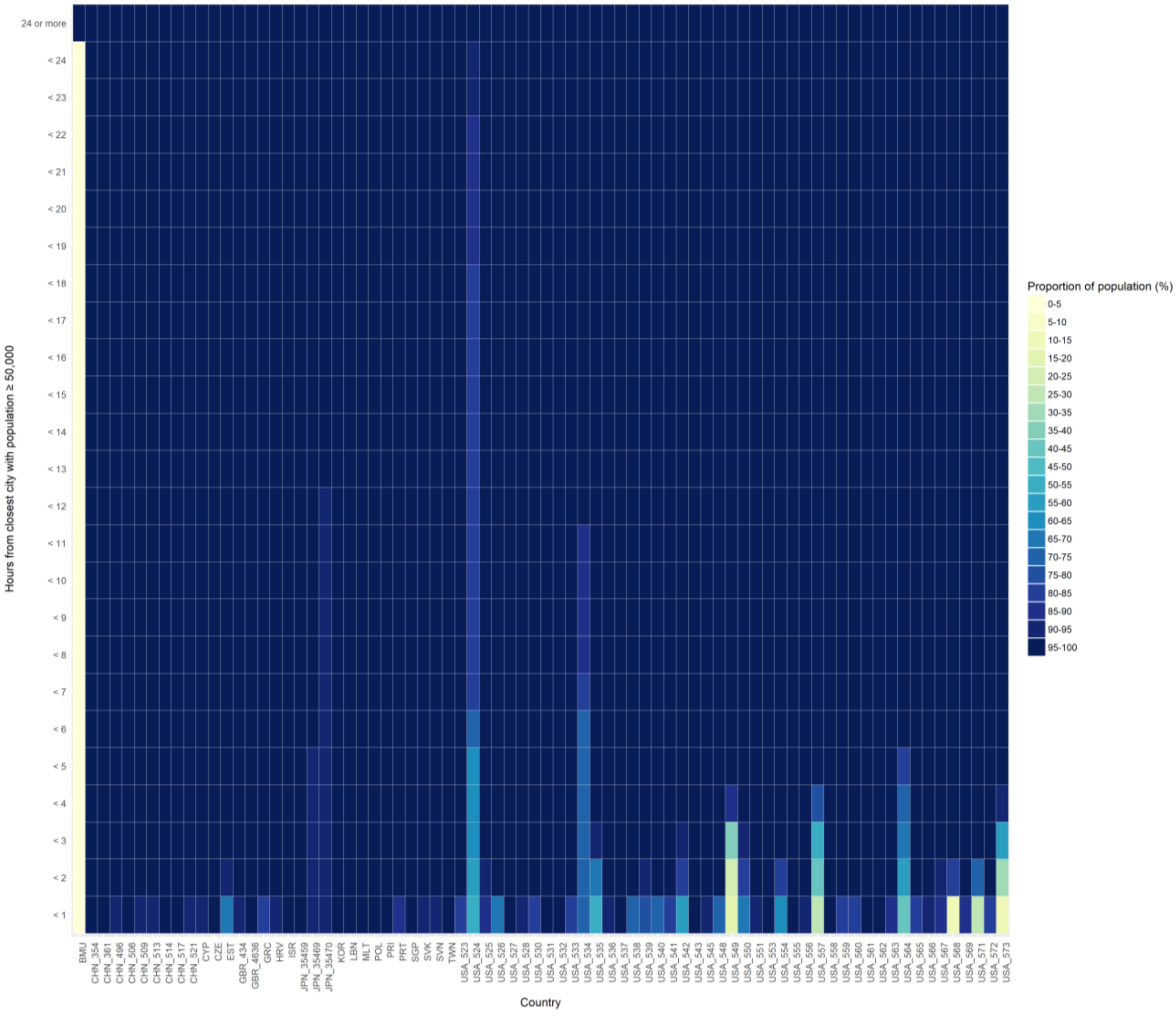

HAQ Index, Decile 10.

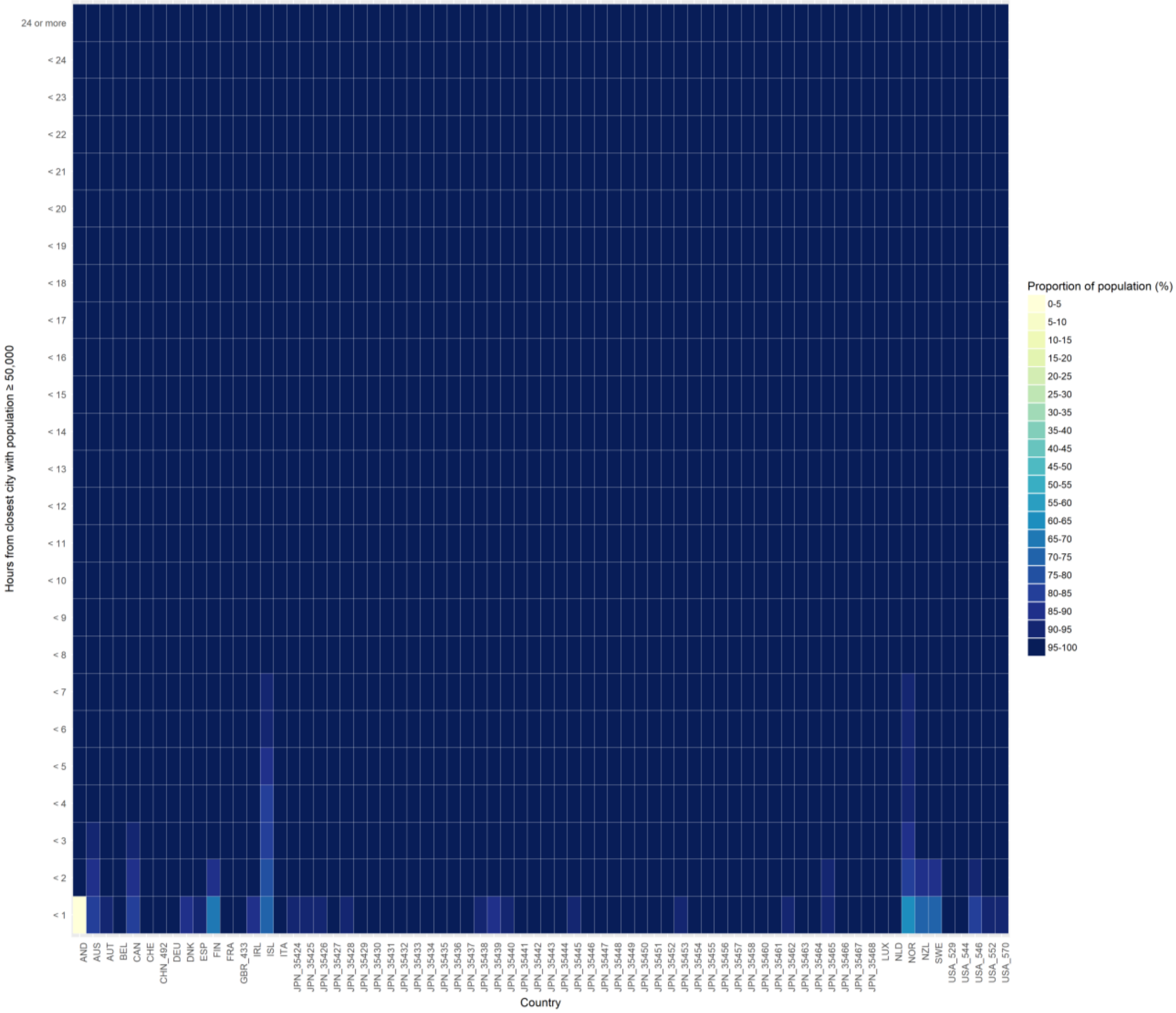

Supplementary Figure 5: Population time-delay plots, per HAQ Index decile. Separate plots per HAQ Index decile (1-10), showing the percentage of the population living within n hours from urban centres with a population ≥50 000. Three letter codes represent each countries ISO3 code; numeric values following ISO3 codes (where applicable), represent the Food and Agriculture Organisation (FAO) Global Administrative Unit Layers (GAUL) code (administrative level one).

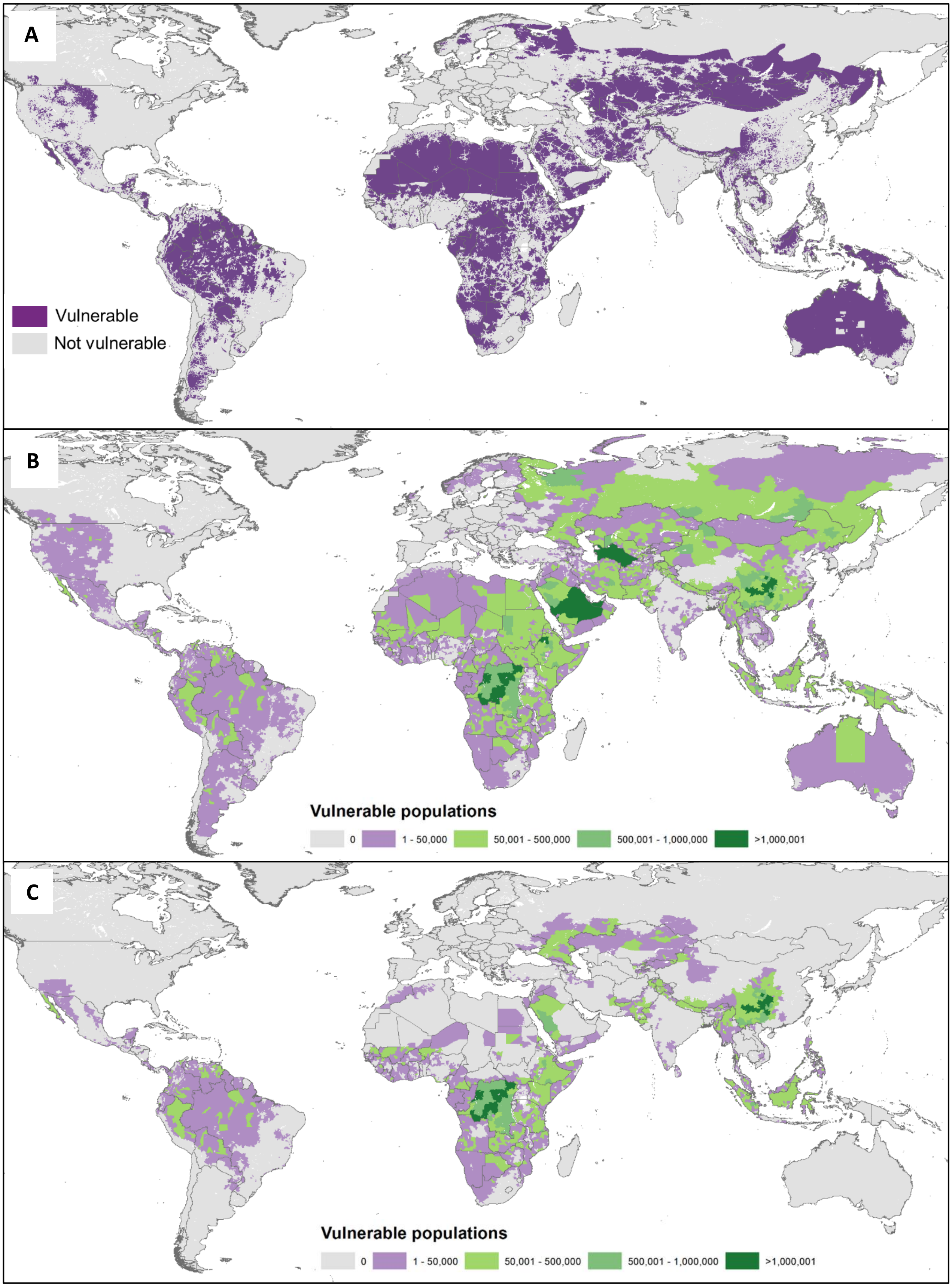
A
Vulnerable
Not vulnerable
B
Vulnerable populations
0
1 - 50,000
50,001 - 500,000
500,001 - 1,000,000
>1,000,001
C
Vulnerable populations
0
1 - 50,000
50,001 - 500,000
500,001 - 1,000,000
>1,000,001

Supplementary Figure 6: Vulnerable population hotspots. This map indicates the absolute numbers of people living in areas within the range of one or more medically important venomous snake species, and more than three hours away from major urban centres, for HAQ Index deciles 1-10. A: pixel-level vulnerability surface (vulnerability to all species of medically important snakes). B: Aggregated administrative level two vulnerability to all species of medically important venomous snakes. C: Aggregated administrative level two vulnerability to only those species for which no effective therapy is currently listed by WHO.

Supplementary Table 2: Vulnerable population count. Country-level count of vulnerable peoples living within the range of one or more medically important venomous snake species and more than three hours from urban centers with a population ≥50 000, provided per HAQ Index decile [ranging from 1 (low) to 10 (high)].

**Country**	**Vulnerable Populations (per HAQ Index decile)**									
	**1**	**2**	**3**	**4**	**5**	**6**	**7**	**8**	**9**	**10**
Afghanistan	7803553									
Albania								13793		
Algeria					1140427					
Angola		4881993								
Argentina						1858578				
Armenia							56694			
Australia										1148363
Austria										195
Azerbaijan						302949				
Bangladesh				359780						
Belarus								26893		
Belize					126937					
Benin	126660									
Bhutan				268986						
Bolivia				1519803						
Botswana				325445						
Brazil					4625526	51488	5173			
Brunei Darussalam								9630		
Bulgaria								3395		
Burkina Faso	709068									
Burundi	10597									
Cote d'Ivoire	486076									
Cambodia			588431							
Cameroon		1422974								
Canada										157780
Central African Republic	2998037									
Chad	3532131									

China				231307	7149839	10009387	17629364	19547364	1940482	2734
Colombia						6807867				
Congo		521800								
Costa Rica							145203			
Dem People's Rep of Korea				341665						
Democratic Republic of the Congo	23640458									
Denmark										15959
Djibouti		103302								
Ecuador					629398					
Egypt					336017					
Equatorial Guinea				242345						
Eritrea	2709721									
Estonia									27716	
Ethiopia	17543888									
Finland										26077
France										339
Gabon			499707							
Gambia		4150								
Georgia						155877				
Ghana			370252							
Greece									52602	
Guatemala				1270558						
Guinea	518883									
Guinea-Bissau	120745									
Guyana				138904						
Honduras				476651						
India		289370	2362089	5499503		282018				
Indonesia			14827781							
Iran (Islamic Republic of)							10464201			
Iraq				887209						
Italy										1396
Japan									104663	48422
Jordan							91863			
Kazakhstan							5150647			
Kenya			1829918							
Korea, Republic of									283	
Kuwait								80573		
Kyrgyzstan					772033					

Lao People's Democratic Republic			2639241							
Lesotho		221546								
Liberia		664940								
Libya							388576			
Malawi		148021								
Malaysia						1791023				
Mali		3326350								
Mauritania			2671588							
Mexico					957599	1049820	284836			
Mongolia				1272586						
Montenegro								255		
Morocco					344538					
Mozambique	3430172									
Myanmar			3740417							
Namibia			765829							
Nepal			3256668							
Nicaragua					882138					
Niger	3335837									
Nigeria			2401524							
Norway										61101
Oman								365802		
Pakistan			6312693							
Panama						345512				
Papua New Guinea		3686437								
Paraguay					406918					
Peru						3548552				
Philippines				1601070						
Qatar								1549		
Romania								8542		
Russian Federation								9582896		
Rwanda			32081							
Saint Lucia						12354				
Sao Tome and Principe			4348							
Saudi Arabia								7354954		
Senegal		935734								
Sierra Leone	169374									
Somalia	2510541									
South Africa				404616						

South Sudan	4819771									
Sri Lanka							62951			
Sudan				14475517						
Suriname				94635						
Swaziland			226							
Sweden										151764
Switzerland										6705
Syrian Arab Republic						267460				
Taiwan									15775	
Tajikistan				657894						
Thailand							885426			
The former Yugoslav Republic of Macedonia								5701		
Timor-Leste			152212							
Togo		16536								
Trinidad and Tobago						32606				
Tunisia							79786			
Turkey							393639			
Turkmenistan					5006267					
U.K. of Great Britain and Northern Ireland									2009	
Uganda		414640								
Ukraine								19122		
United Arab Emirates							1028512			
United Republic of Tanzania		6206331								
United States of America								6	1112329	581
Uruguay							41422			
Uzbekistan					5346025					
Venezuela						3925520				
Viet Nam					576632					
Yemen			3809162							
Zambia	2192019									
Zimbabwe		936801								
**Grand Total**	**76657533**	**23780923**	**46264166**	**30068475**	**28300296**	**30441012**	**36708293**	**37020476**	**3255859**	**1621416**

Medically Important Category Descriptions and Inclusion Information

Screening of the WHOs *"Guidelines for the production, control and regulation of snake antivenom immunoglobulins"*[1] identified 287 venomous snake species of medical importance. These species belonged to four main families: *Atractaspididae* (n = five), *Colubridae* (n = six), *Elapidae* (n = 99), and *Viperidae* (n = 177) [Supplementary Table 3]. The medical importance of each species was defined as:

"**Category 1:** Highest medical importance. Highly venomous snakes which are common or widespread and cause numerous snakebites, resulting in high levels of morbidity, disability or mortality.

Category 2: Secondary medical importance. Highly venomous snakes capable of causing morbidity, disability or death, but for which (i) exact epidemiological or clinical data may be lacking; and/or (ii) are less frequently implicated (due to their activity cycles, behaviour, habitat preferences or occurrence in areas remote to large human populations)."[1]

The medical classification may vary between countries within a species' range, for example, *Bothrops diporus* is of highest medical importance (Category 1) in Argentina, but of secondary medical importance (Category 2) in Brazil and Paraguay. Where the medical classification was absent for a species (n = six), we assigned such species as being of secondary medical importance.

Supplementary Table 3. Genus inclusion list.

Family	Genus (count of individual species)
Atractaspididae	*Atractaspis (5)*
Colubridae	*Dispholidus (1), Rhabdophis (1), Thelotornis (4)*
Elapidae	*Acanthophis (7), Austrelaps (3), Bungarus (14), Calliophis (2), Dendroaspis (4), Hemachatus (1), Hoplocephalus (3), Micropechis (1), Micruroides (1), Micrurus (9), Naja (27), Notechis (1), Ophiophagus (1), Oxyuranus (3), Pseudechis (8), Pseudohaje (2), Pseudonaja (9), Tropidechis (1), Walterinnesia (2)*
Viperidae	*Agkistrodon (6), Atheris (3), Atropoides (6), Bitis (6), Bothriechis (9), Bothrocophias (5), Bothrops (29), Calloselasma (1), Cerastes (2), Cerrophidion (6), Crotalus (14), Cryptelytrops (6), Daboia (5), Deinagkistrodon (1), Echis (11), Eristocophis (1), Gloydius (8), Himalayophis (1), Hypnale (3), Lachesis (4), Macrovipera (2), Montivipera (2), Ophryacus (2), Parias (2), Porthidium (9), Proatheris (1), Protobothrops (6), Pseudocerastes (2), Sistrurus (2), Trimeresurus (6), Tropidolaemus (2), Vipera (13), Viridovipera (1)*

Multivariate Environmental Similarity Surfaces

We constructed each species-specific MESS using occurrence data located within the expert opinion range (EOR) as the reference dataset, and eight bioclimatic covariates thought to influence the distribution of snake species (Supplementary Table 4), through utilization of the 'mess' function in the 'dismo' package in R,[2] identifying areas of environmental interpolation and extrapolation beyond the current EOR.

To reduce the effect of environmental outliers within the training sample, which may heavily influence the MESS, we generated 100 subsampled MESS for each species, each of which was transformed into a binary interpolation/extrapolation surface. The 100 separate binary MESS iterations were aggregated, to provide a relative likelihood of interpolation surface, with values ranging from '0' (strong evidence of environmental dissimilarity) to '100' (strong evidence of environmental similarity). We applied a 95% threshold to the aggregated MESS to return to a single binary surface of interpolation/extrapolation for each species. Cells which were predicted to be areas of interpolation in 95 out of 100 bootstraps were assigned the cell value '1', all other cells were assigned the cell value '0' and were deemed to be cells of environmental extrapolation.

Utilizing this binary bootstrapped surface, occurrence records for each species (which were currently outside of the EOR) were classified. Out-of-range records were overlaid, and if they were located within cells of interpolation they were classified as 'MESS +ve'; if records were located in cells of extrapolation, they were classified as 'MESS –ve'. For a visual example of this process see Supplementary Figure 7.

Supplementary Table 4. Covariates used to construct each Multivariate Environmental Similarity Surface.

Covariate	Source
Daytime land surface temperature (mean) [synoptic]	Gap-filled MODIS LST data.[3,4]
Daytime land surface temperature (standard deviation) [synoptic]	Gap-filled MODIS LST data.[3,4]
Night-time land surface temperature (mean) [synoptic]	Gap-filled MODIS LST data.[3,4]
Night-time land surface temperature (standard deviation) [synoptic]	Gap-filled MODIS LST data.[3,4]
Tasselled cap brightness (standard deviation) [synoptic]	Gap-filled MODIS satellite data.[3,5,6]
Tasselled cap wetness (mean) [synoptic]	Gap-filled MODIS satellite data.[3,5,6]
Tasselled cap wetness (standard deviation) [synoptic]	Gap-filled MODIS satellite data.[3,5,6]
Elevation [synoptic]	Shuttle Radar Topography Mission (SRTM).[6,7]

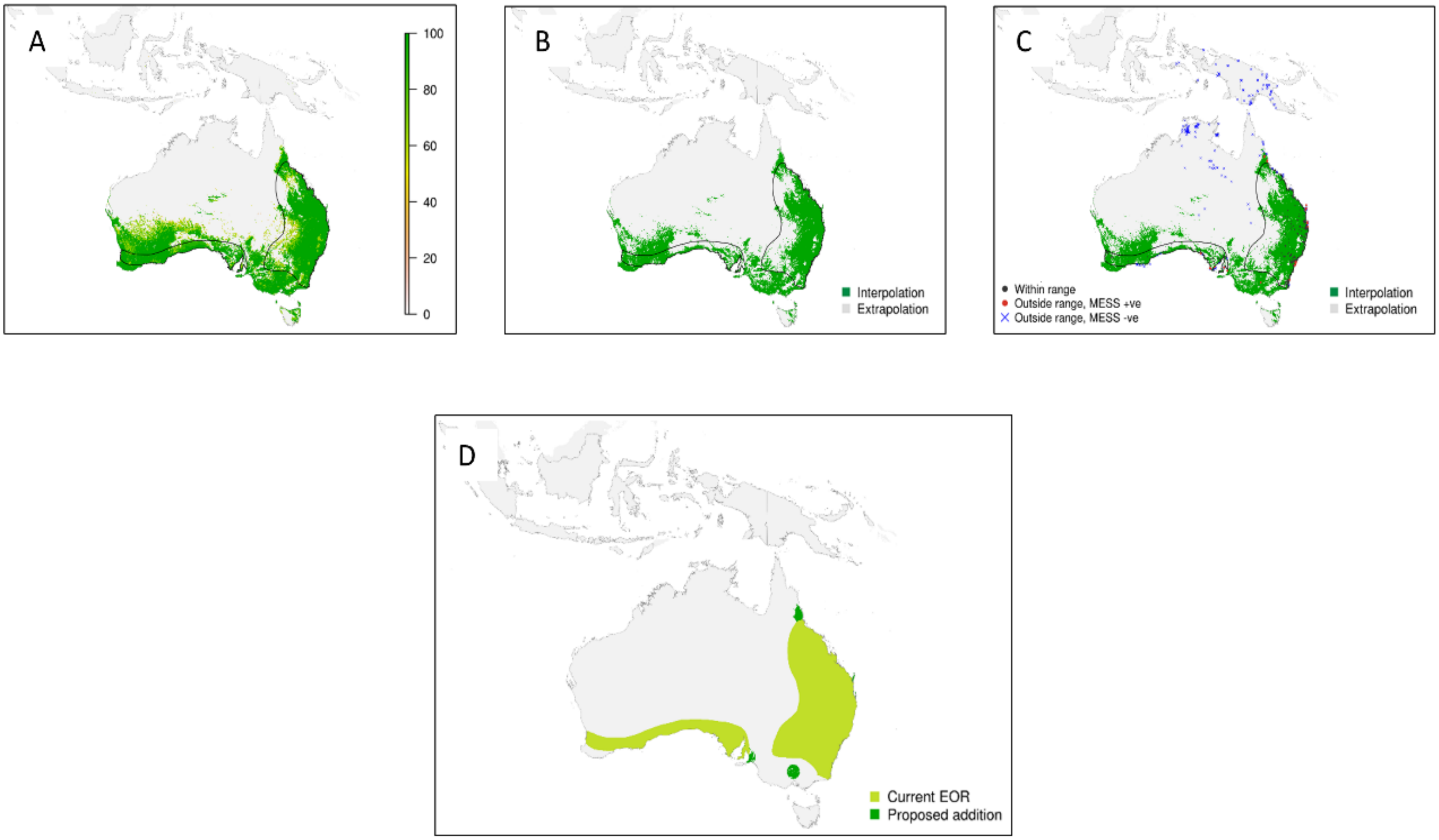

Supplementary Figure 7. Visualisation of MESS construction and record evaluation process. Panel A represents a stacked output of 100 MESS iterations, with cell values ranging from 0 to 100, generated using occurrence records within the currently accepted expert opinion range (black outline). Panel B represents a binary version of the stacked output (A), in which cells with a value ≥95 in (A) are classified as being cells of environmental interpolation, and cells <95 in (A) are classified as being cells of environmental extrapolation. Out-of-range records are then overlaid on top of the new binary surface (Panel C), and are classified as being MESS +ve or MESS –ve. Records which are MESS +ve contribute towards a new range recommendation (Panel D).

Sensitivity Analysis of Travel Time Covariate

We use travel time as a proxy for healthcare accessibility and have performed a sensitivity for its appropriateness by using a recently published dataset of healthcare facilities for the African continent. To our knowledge no global dataset for this information exists, hence the need to rely on travel time to population centres with more than 50,000 individuals as a proxy metric. The African dataset, compiled by Ouma & colleagues,[8] represents the most geographically widespread dataset of this nature, consisting of 4,908 hospitals across the continent. A sensitivity analysis was performed by extracting travel time estimates for each hospital from the surface used within our study,[9] and presenting these estimates as a cumulative frequency plot. To allow comparison, 100 iterations of 4908 random coordinates were sampled from within the African continent, and travel time estimates were extracted

for these. Cumulative frequency plots for each iteration were stacked, and visualized against the hospital travel time data. The result of this analysis is given below:

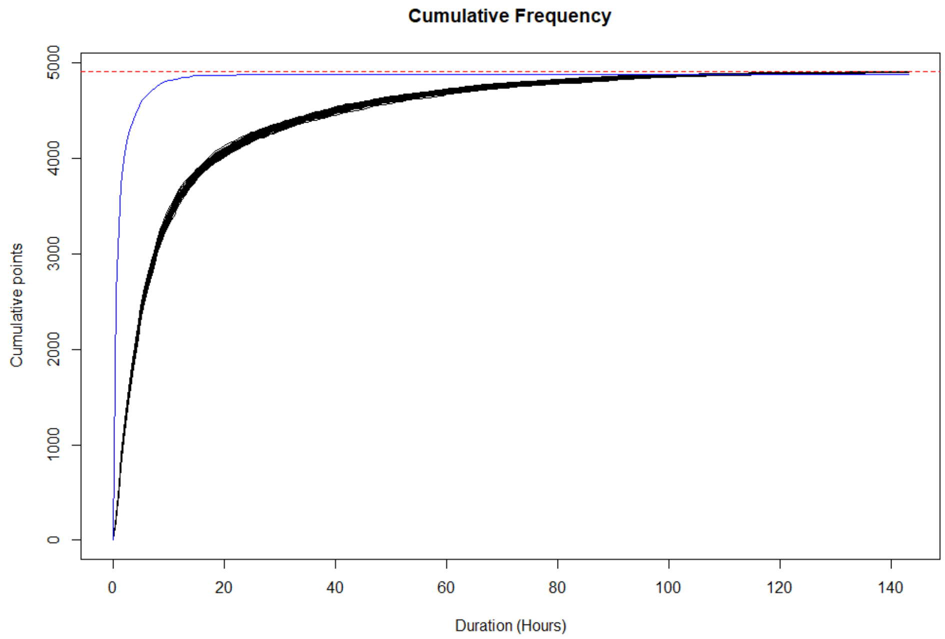

Black lines represent the cumulative frequency of average travel time to urban centers for 4 908 randomly sampled points (100 iterations, one line per iteration); the blue line represents the cumulative frequency of average travel time to urban centers for 4 908 hospitals located within the African continent.

As shown by the above plot, 52.85% (2 594/4 908) of true hospitals are located within 30 minutes travel time from urban centres with a population ≥50 000 and 87.43% (4 291/4 908) hospitals within the African continent are located within 3 hours travel time. Compared with the randomly sampled data, this indicated that the travel time surface serves as a valid proxy for hospital accessibility; with the majority of hospitals being located within 3 hours of an urban centre with a population ≥50 000. This assessment suggests that using travel time to population centres is a suitable proxy for accessibility to healthcare provisioning.

Health Care Access and Quality Index Sensitivity Analysis

To understand how appropriate the HAQ Index measure is as a proxy for poorer health provisioning for snake bites, we regressed the 2005 and 2010 values for the HAQ Index with mean snake mortality estimates for the year 2007 as published in Kasturiratne et al. 2008 (mimicking a similar analysis done by Harrison et al. 2009),[10,11] the results of which are as follows:

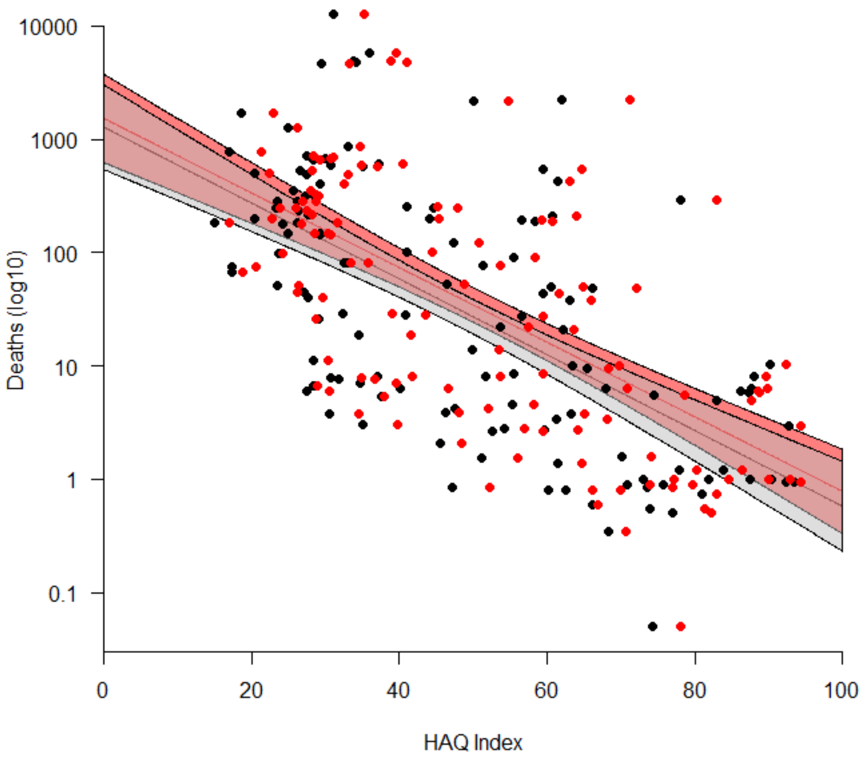

There is a negative association between HAQ Index (where higher scores are indicative of better healthcare access and quality) and deaths (in log space), suggesting that general trends in amenable conditions are associated with snake-bite specific mortality (for HAQ Index 2005 $p < 0.0001$ [black in the figure above], for HAQ Index 2010 $p < 0.0001$ [red in the figure above]).

For snakebites specifically, reporting of treatment variation is focused more on treatment seeking behaviour rather than antivenom stocks (both of which are considered as part of the Healthcare Access and Quality Index). In Kenya, a large proportion (80% [40/50]) of patients sought traditional medicine prior to hospital visits,[12] compared to 100% (114/114) in Lao PDR and 43.3% (301/695) in Sri Lanka.[13,14] Interestingly, in a different study of the effectiveness of primary hospital management in Sri Lanka, 18% (399/2186) admissions had to be referred to another hospital, the majority of which (85% [341/399]) were judged to be unnecessary.[15] Lack of antivenom was the cause for transfer in only two instances; the authors suggested that rural doctors who refer may be concerned about their ability to treat anaphylactic reactions. The general applicability of these findings to the most vulnerable settings is unknown (Sri Lanka is in the 7th decile, whereas Kenya and Lao PDR are in the 3rd decile); Medicin Sans Frontieres materials suggest that in their priority countries (South Sudan, Central African Republic and Chad) antivenom provisioning is low. Finally, a study of Nigerian clinicians found that overall knowledge of snakes and treatment options was low (evaluated over a variety of measures), but knowledge was correlated with years of practitioner experience, and varied geographically.[16]

Additional References

1. World Health Organization. WHO guidelines for the production, control and regulation of snake antivenom immunoglobulins: post ECBS version. Geneva, Switzerland, 2016.

2. Hijmans RJ, Phillips S, Leathwick J, Elith J. dismo: Species Distribution Modeling. Version 1.1-4. 2017. https://cran.r-project.org/web/packages/dismo/index.html (accessed 29th May 2017).
3. Weiss DJ, Atkinson PM, Bhatt S, Mappin B, Hay SI, Gething PW. An effective approach for gap-filling continental scale remotely sensed time-series. *ISPRS J Photogramm Remote Sens* 2014; **98**: 106-18.
4. Wan Z, Zhang Y, Zhang Q, Li Z-l. Validation of the land-surface temperature products retrieved from Terra Moderate Resolution Imaging Spectroradiometer data. *Remote Sens Environ* 2002; **83**: 163–80.
5. Lobser SE, Cohen WB. MODIS tasselled cap: land cover characteristics expressed through transformed MODIS data. *International Journal of Remote Sensing* 2007; **28**(22): 5079-101.
6. Weiss DJ, Mappin B, Dalrymple U, et al. Re-examining environmental correlates of Plasmodium falciparum malaria endemicity: a data-intensive variable selection approach. *Malaria Journal* 2015; **14**(1): 68.
7. Farr TG, Rosen PA, Caro E, et al. The Shuttle Radar Topography Mission. *Reviews of Geophysics* 2007; **45**(2).
8. Ouma PO, Maina J, Thuranira PN, et al. Access to emergency hospital care provided by the public sector in sub-Saharan Africa in 2015: a geocoded inventory and spatial analysis. *Lancet Glob Health*; **6**(3): e342-e50.
9. Weiss DJ, Nelson A, Gibson HS, et al. A global map of travel time to cities to assess inequalities in accessibility in 2015. *Nature* 2018; **553**: 333-6.
10. Kasturiratne A, Wickremasinghe AR, de Silva N, et al. The global burden of snakebite: a literature analysis and modelling based on regional estimates of envenoming and deaths. *PLoS Med* 2008; **5**(11): e218.
11. Harrison RA, Hargreaves A, Wagstaff SC, Faragher B, Lalloo DG. Snake envenoming: a disease of poverty. *PLoS Negl Trop Dis* 2009; **3**(12): e569.
12. Sloan DJ, Dedicoat MJ, Lalloo DG. Healthcare-seeking behaviour and use of traditional healers after snakebite in Hlabisa sub-district, KwaZulu Natal. *Tropical Medicine & International Health* 2007; **12**(11): 1386-90.
13. Vongphoumy I, Phongmany P, Sydala S, Prasith N, Reintjes R, Blessmann J. Snakebites in two rural districts in Lao PDR: Community-based surveys disclose high incidence of an invisible public health problem. *PLoS Negl Trop Dis* 2015; **9**(6): e0003887.
14. Ediriweera DS, Kasturiratne A, Pathmeswaran A, et al. Health seeking behavior following snakebites in Sri Lanka: Results of an island wide community based survey. *PLoS Negl Trop Dis* 2017; **11**(11): e0006073.
15. Shahmy S, Kularatne SAM, Rathnayake SS, Dawson AH. A prospective cohort study of the effectiveness of the primary hospital management of all snakebites in Kurunegala district of Sri Lanka. *PLoS Negl Trop Dis* 2017; **11**(8): e0005847.
16. Michael GC, Grema BA, Aliyu I, et al. Knowledge of venomous snakes, snakebite first aid, treatment, and prevention among clinicians in northern Nigeria: a cross-sectional multicentre study. *Trans R Soc Trop Med Hyg* 2018; **112**(2): 47-56.
