# Supplementary material for: Vulnerability to snakebite envenoming: a global mapping of hotspots
Source: Lancet. 2018 Aug 25;392(10148):673–84. doi: 10.1016/S0140-6736(18)31224-8 (PMC6115328; doi:10.1016/S0140-6736(18)31224-8)

THE LANCET

Supplementary appendix 1

This appendix formed part of the original submission and has been peer reviewed. We post it as supplied by the authors.

Supplement to: Longbottom J, Shearer FM, Devine M, et al. Vulnerability to snakebite envenoming: a global mapping of hotspots. *Lancet* 2018; published online July 12. http://dx.doi.org/10.1016/S0140-6736(18)31224-8.

Acanthophis antarcticus

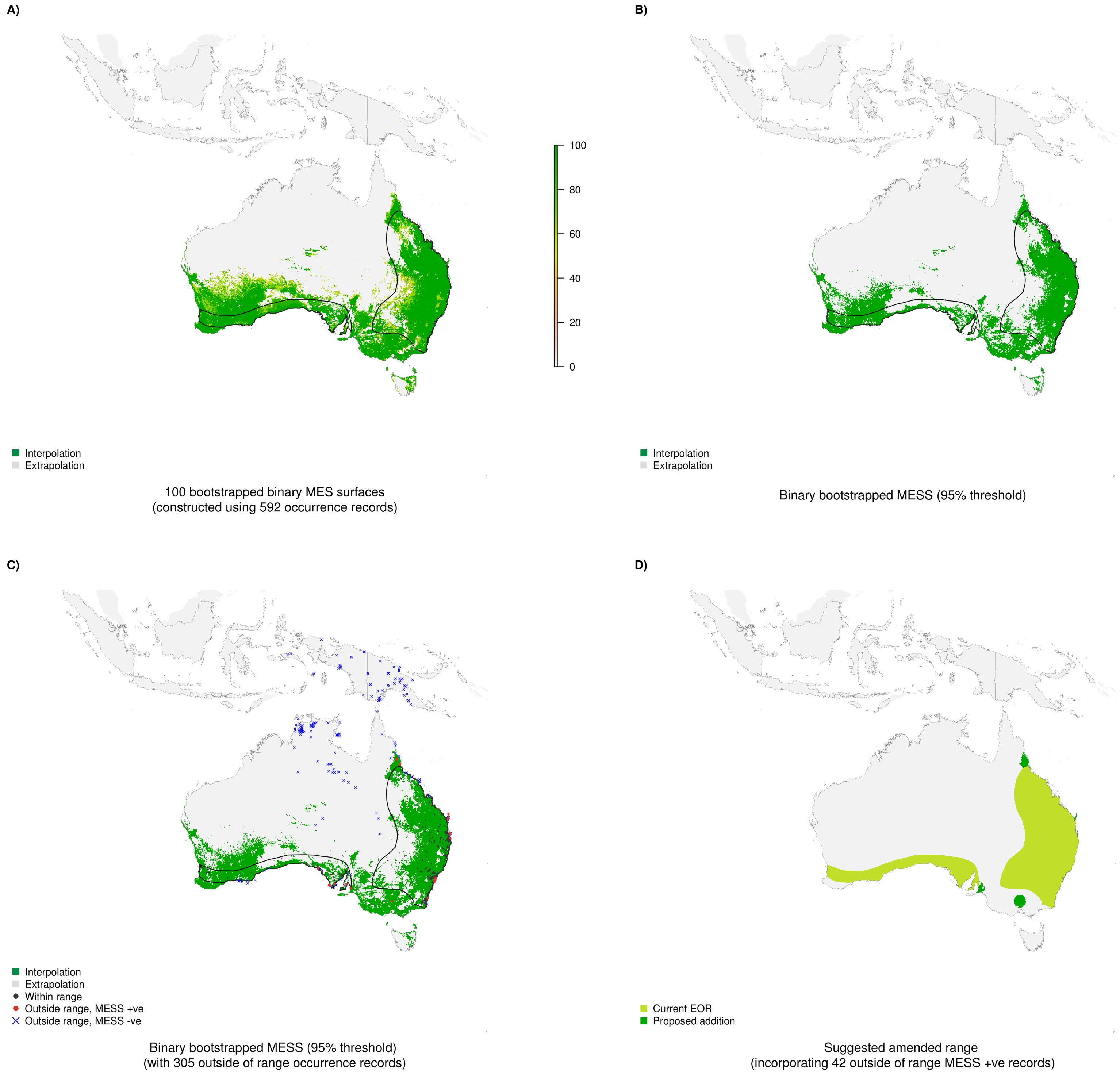

Acanthophis cryptamydros

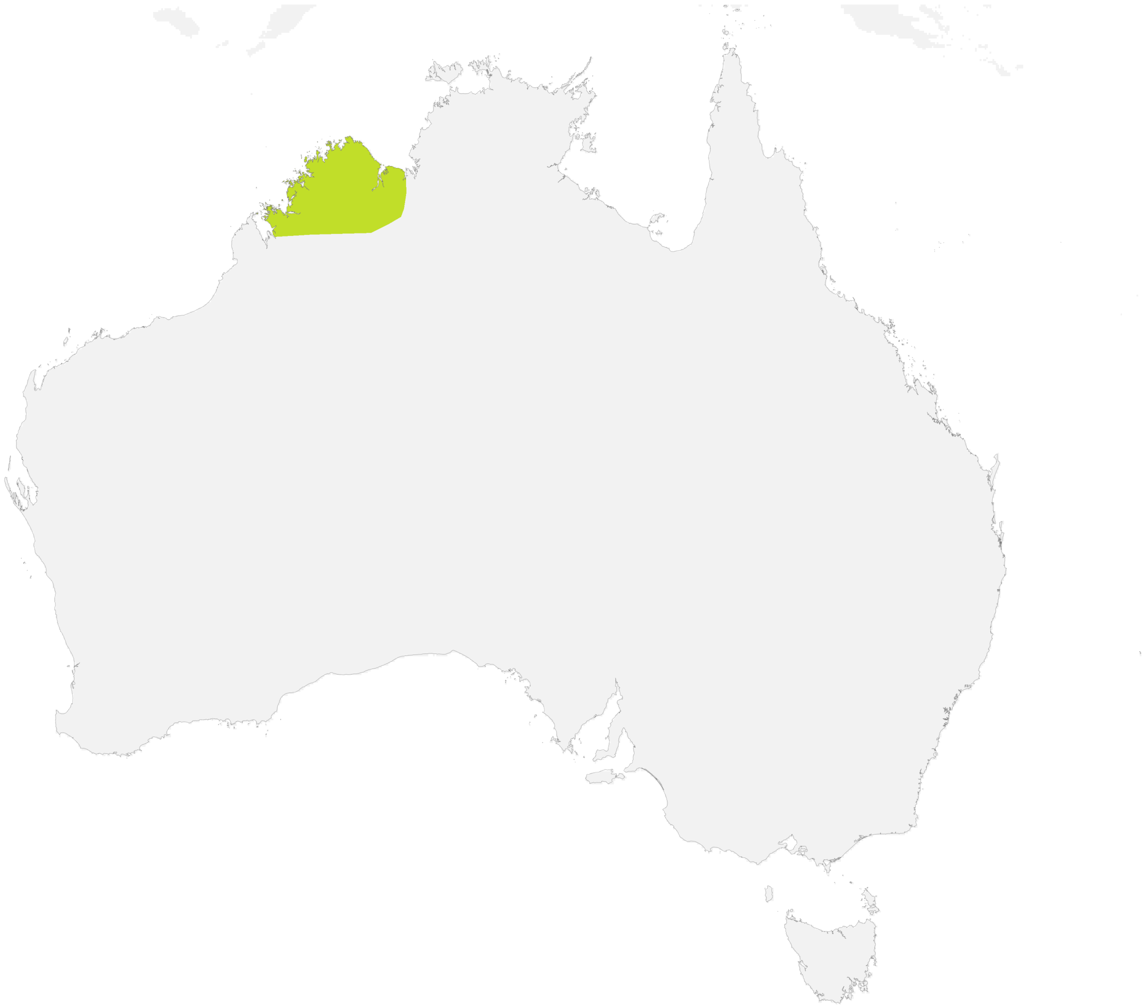

Acanthophis laevis

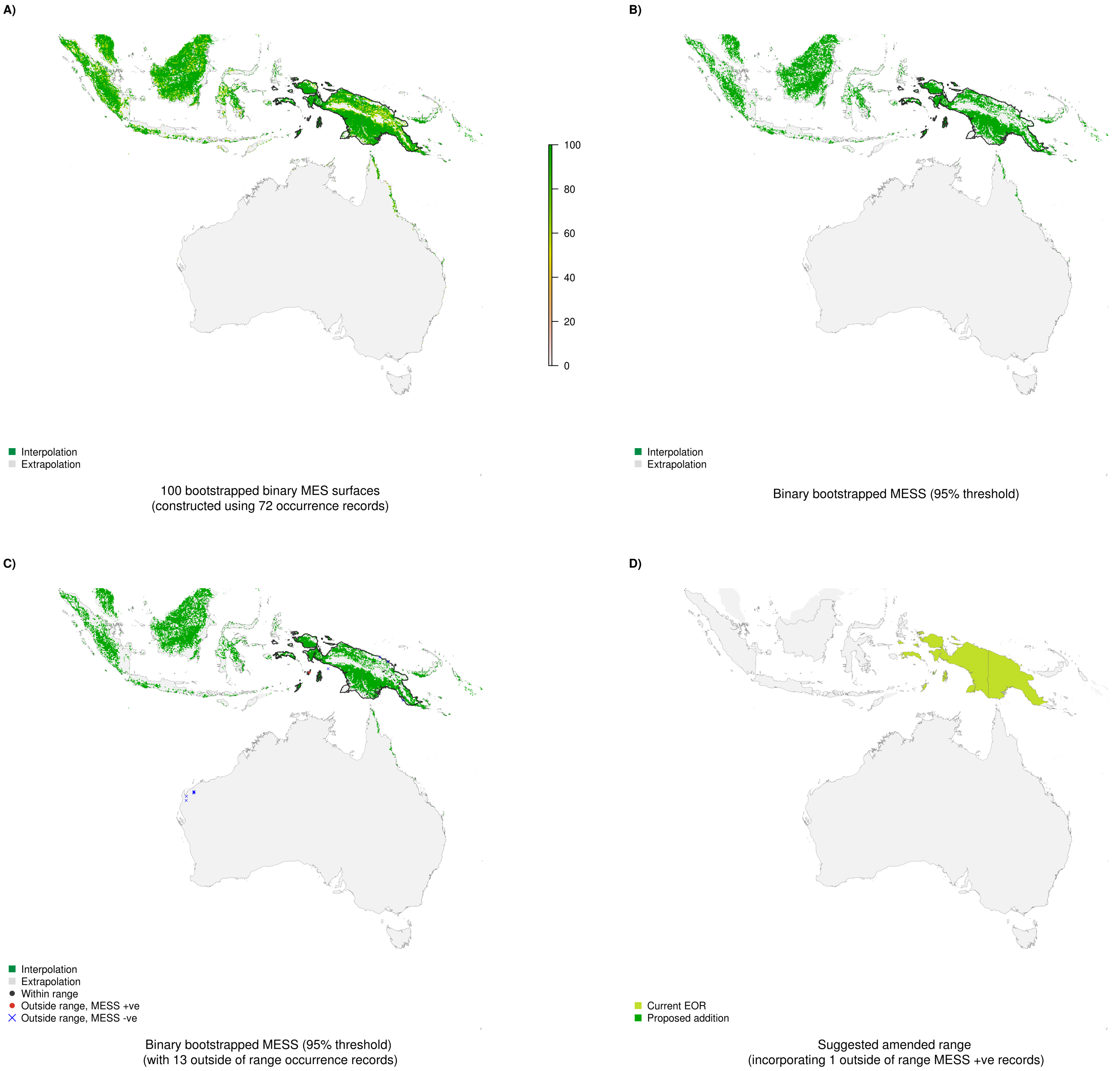

Acanthophis praelongus

A)

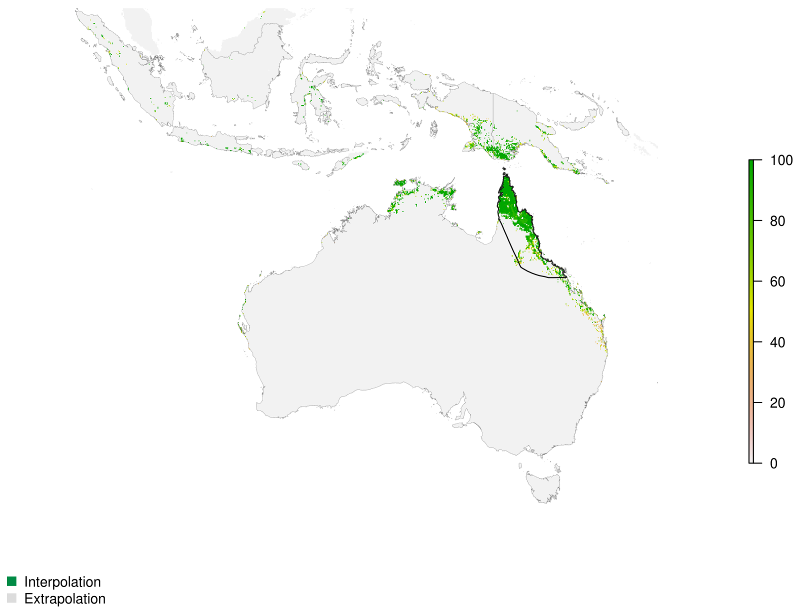

100 bootstrapped binary MES surfaces
(constructed using 73 occurrence records)

B)

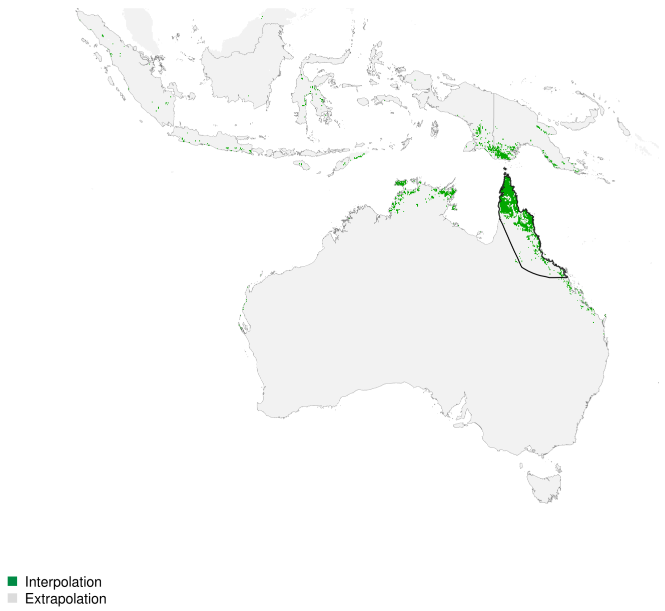

Binary bootstrapped MESS (95% threshold)

C)

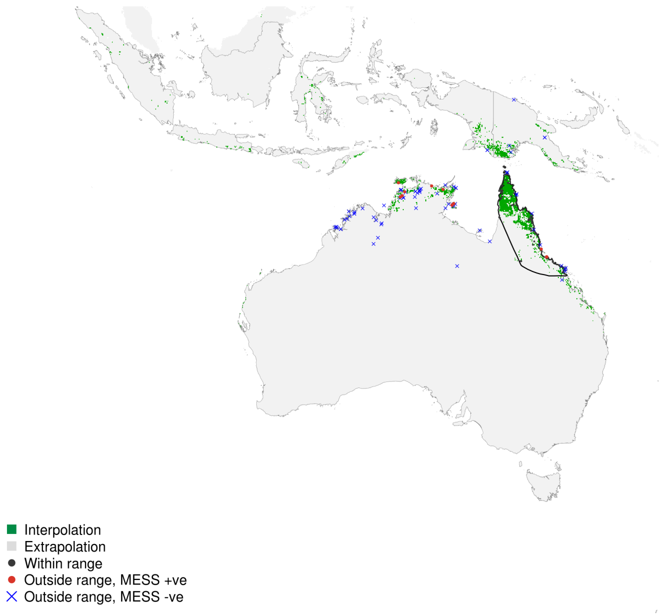

Binary bootstrapped MESS (95% threshold)
(with 90 outside of range occurrence records)

D)

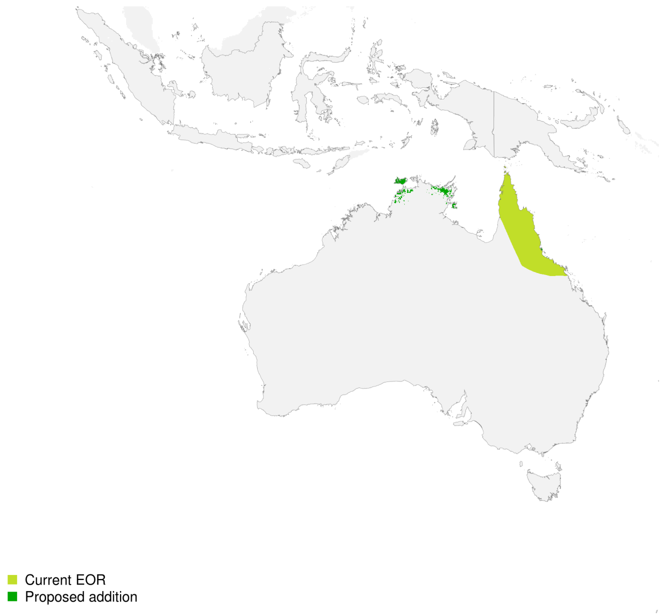

Suggested amended range
(incorporating 17 outside of range MESS +ve records)

Acanthophis pyrrhus

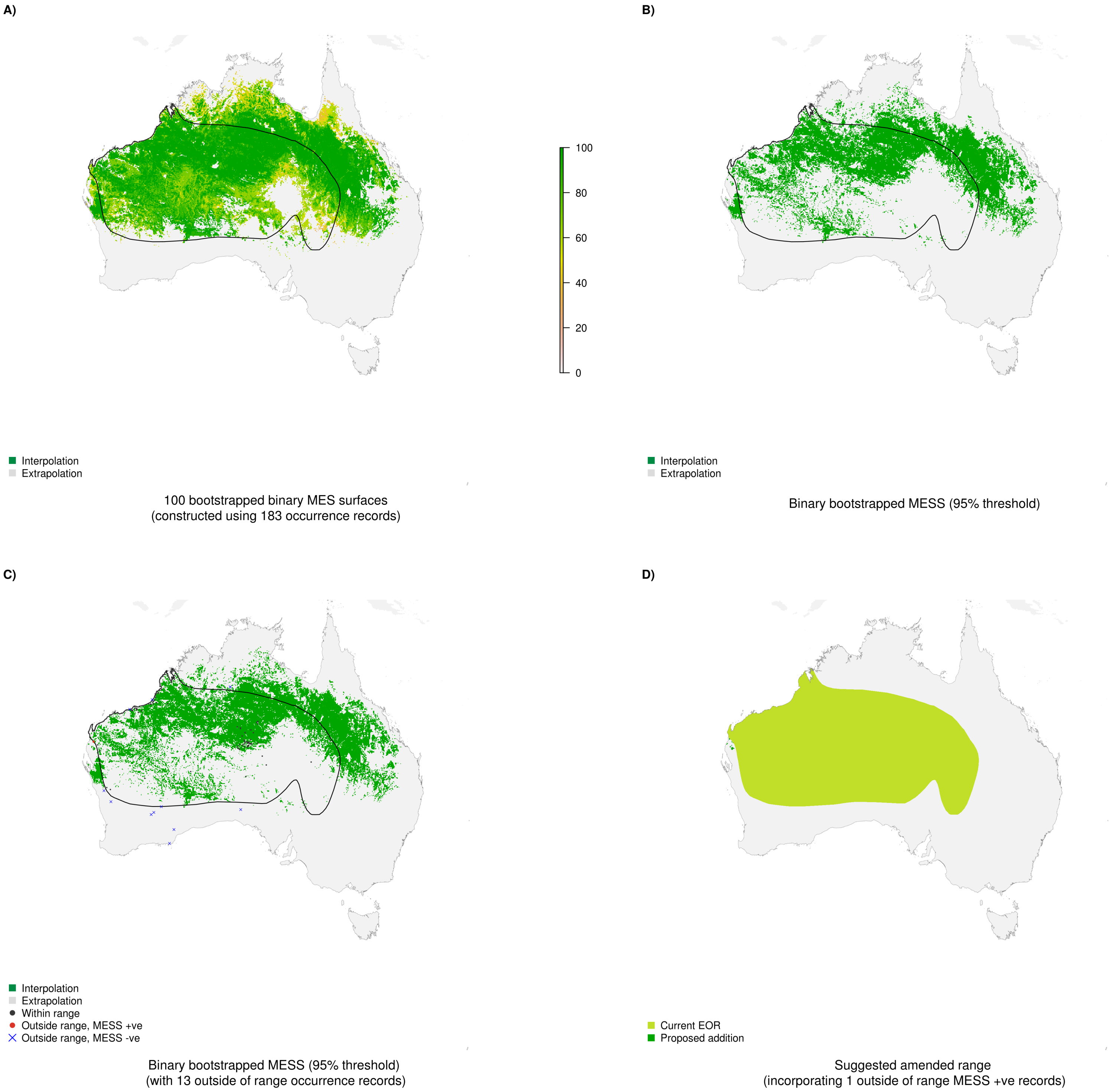

Acanthophis rugosus

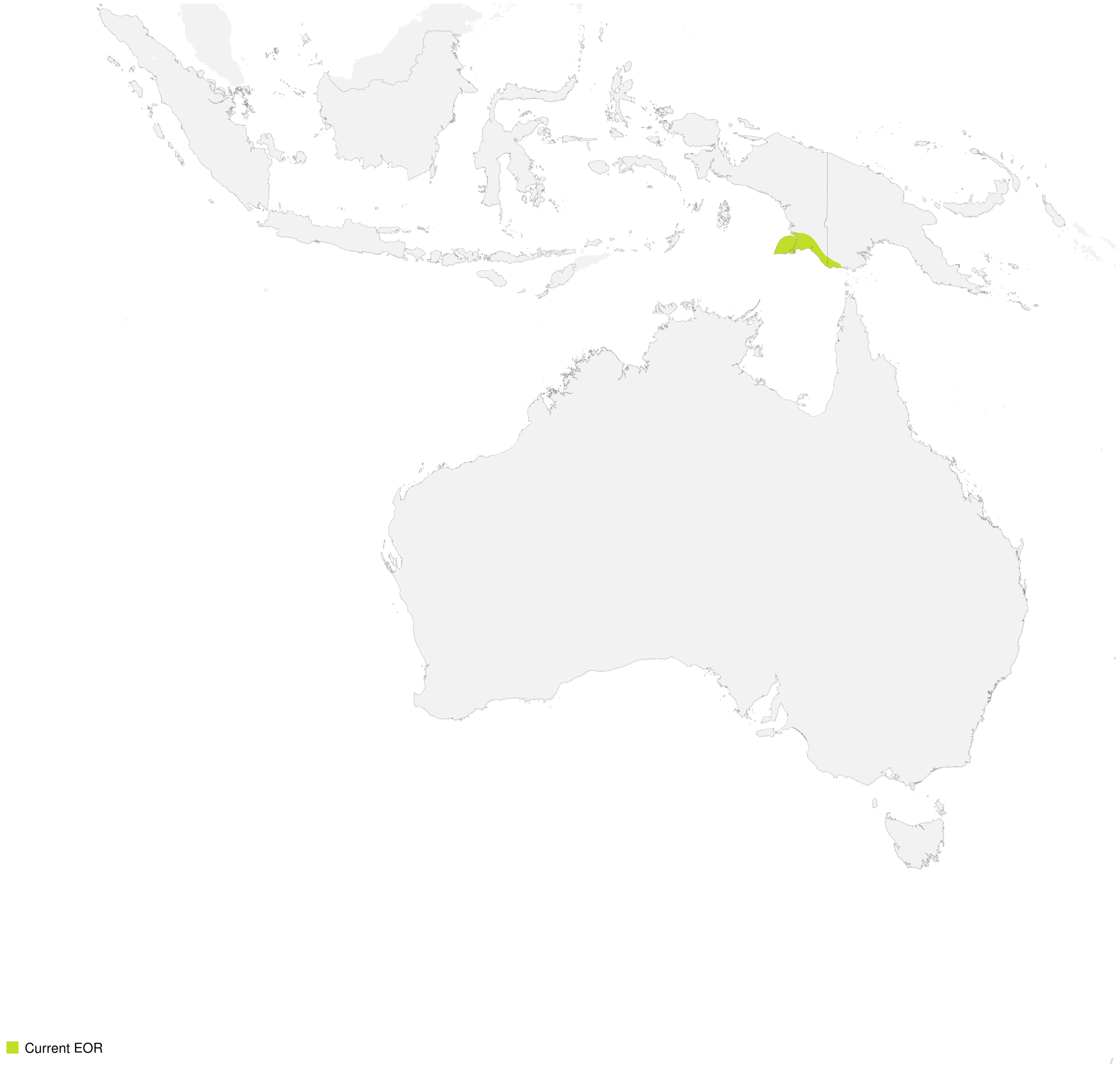

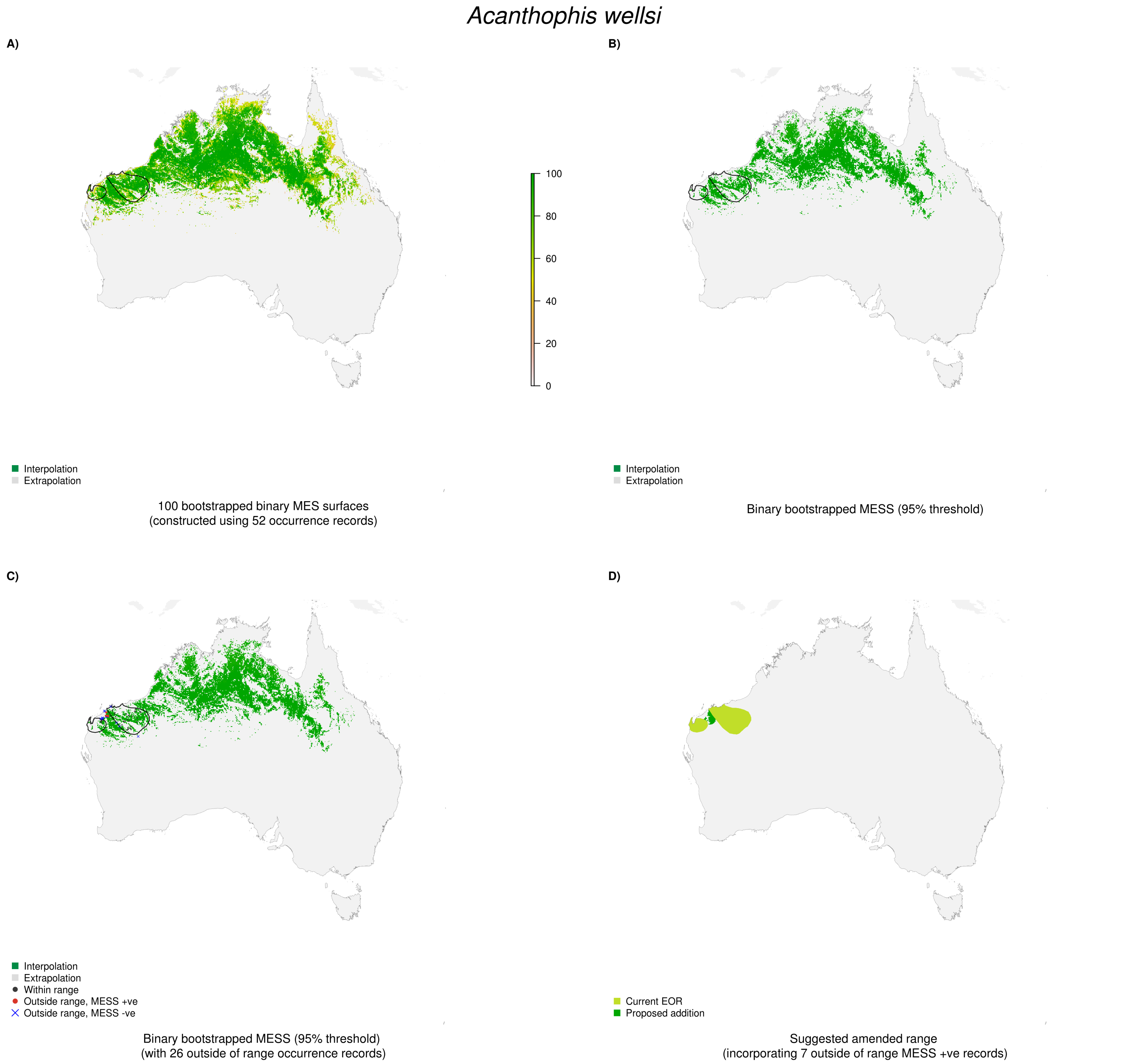

Acanthophis wellsi
A)
100
80
60
40
20
0
Interpolation
Extrapolation
100 bootstrapped binary MES surfaces
(constructed using 52 occurrence records)
B)
Interpolation
Extrapolation
Binary bootstrapped MESS (95% threshold)
C)
Interpolation
Extrapolation
Within range
Outside range, MESS +ve
Outside range, MESS -ve
Binary bootstrapped MESS (95% threshold)
(with 26 outside of range occurrence records)
D)
Current EOR
Proposed addition
Suggested amended range
(incorporating 7 outside of range MESS +ve records)

Agkistrodon bilineatus

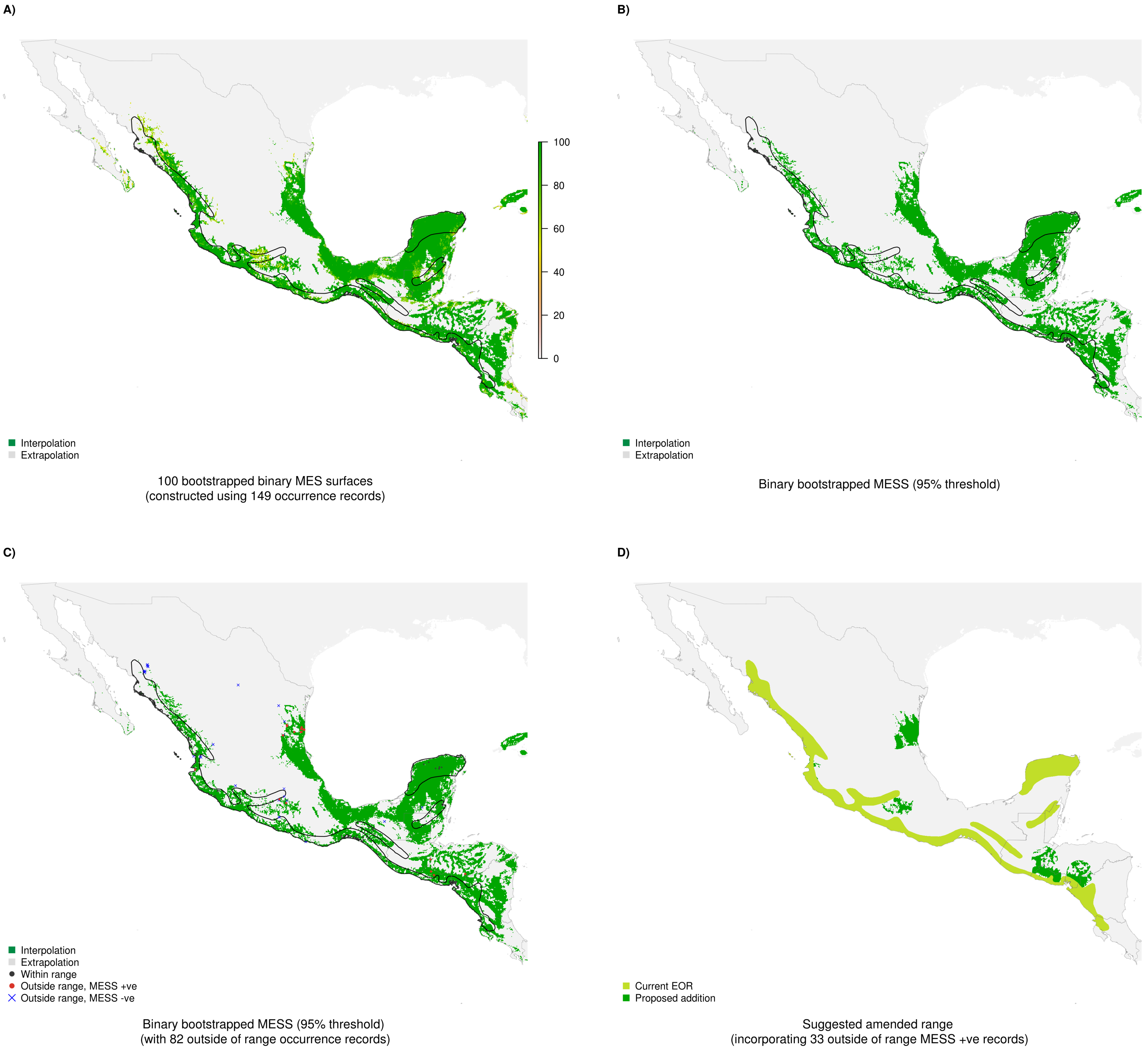

Agkistrodon contortrix

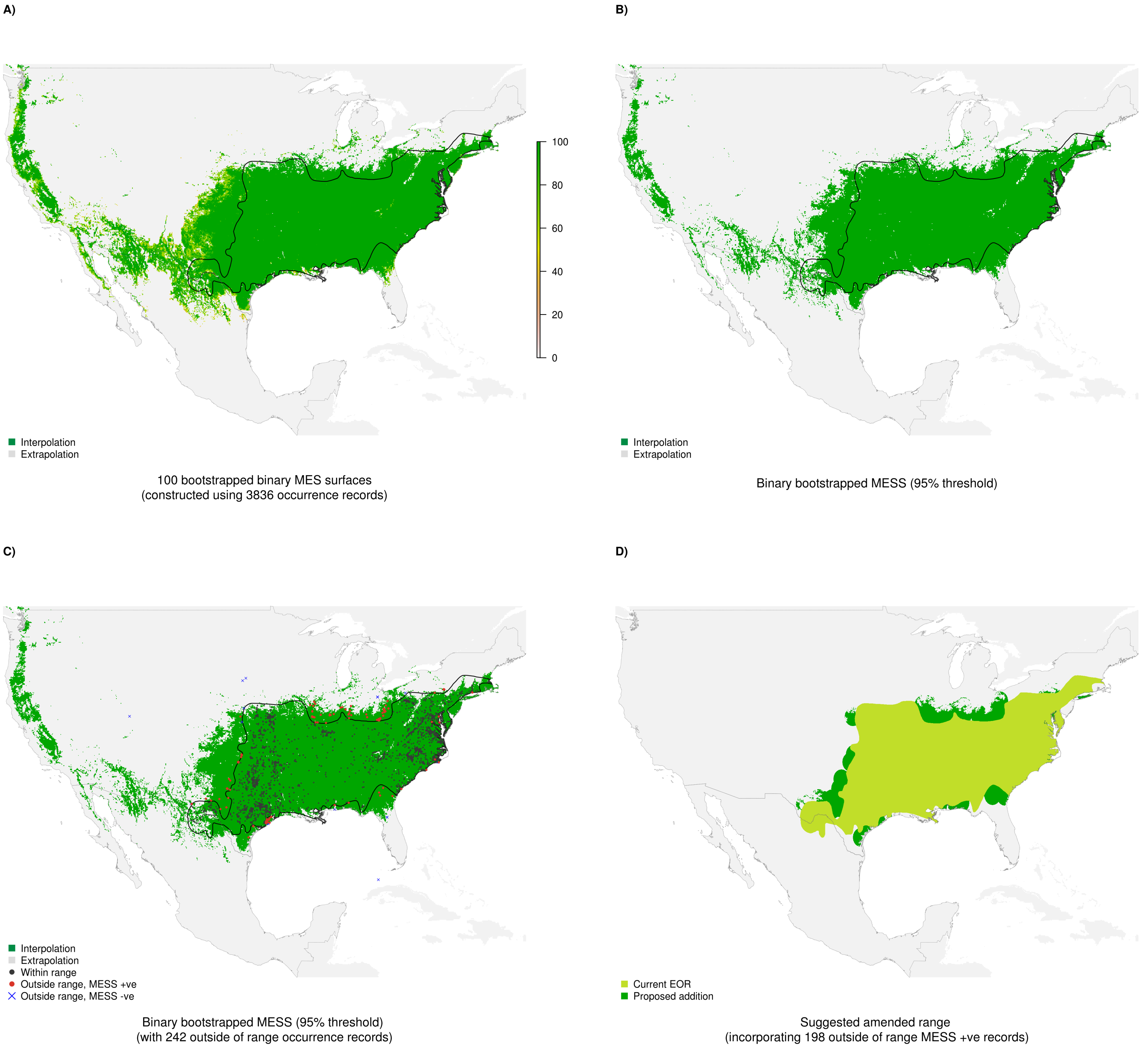

Agkistrodon piscivorus

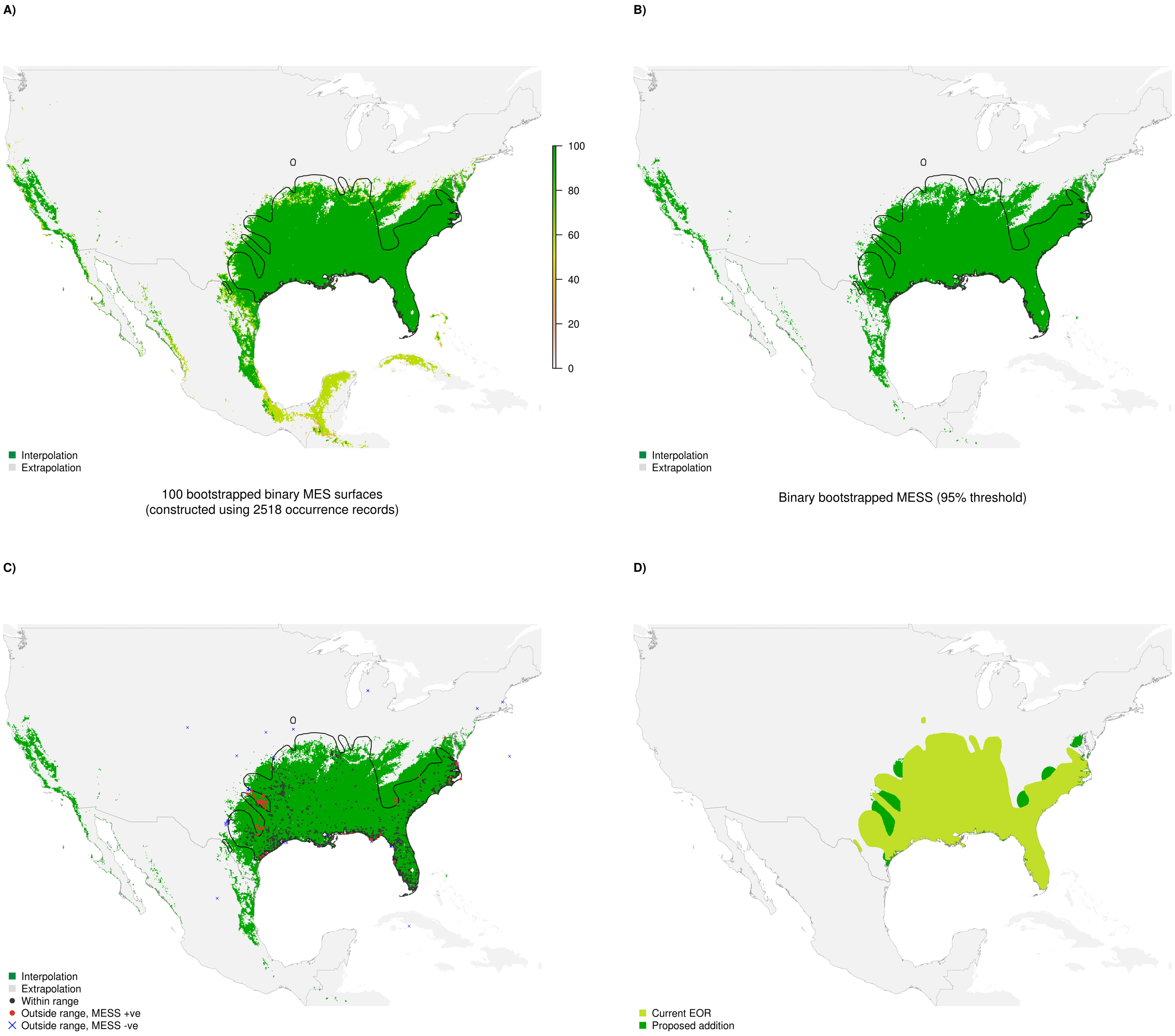

100 bootstrapped binary MES surfaces
(constructed using 2518 occurrence records)

Binary bootstrapped MESS (95% threshold)

Binary bootstrapped MESS (95% threshold)
(with 203 outside of range occurrence records)

Suggested amended range
(incorporating 157 outside of range MESS +ve records)

Agkistrodon taylori

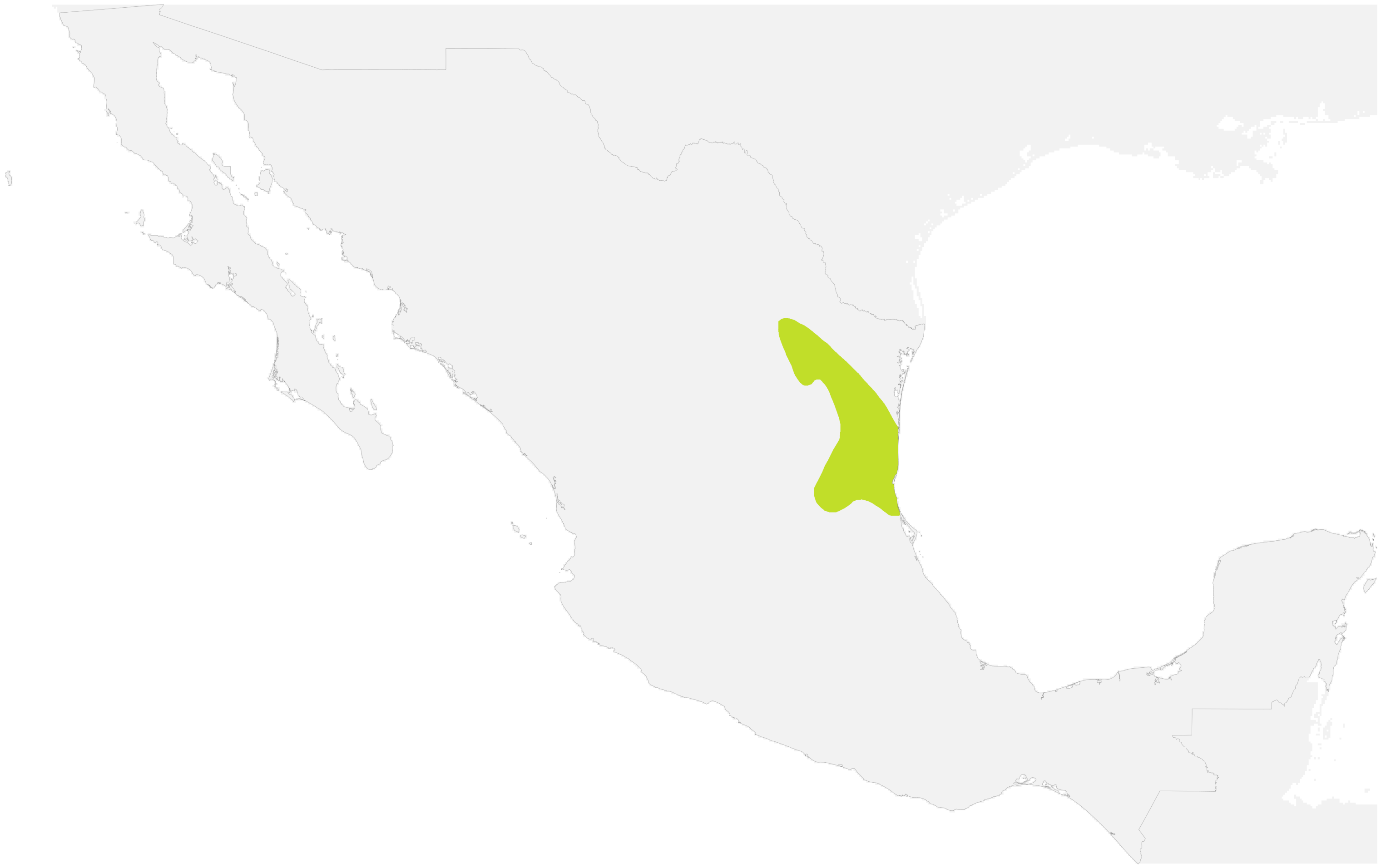

Current EOR

Atheris broadleyi

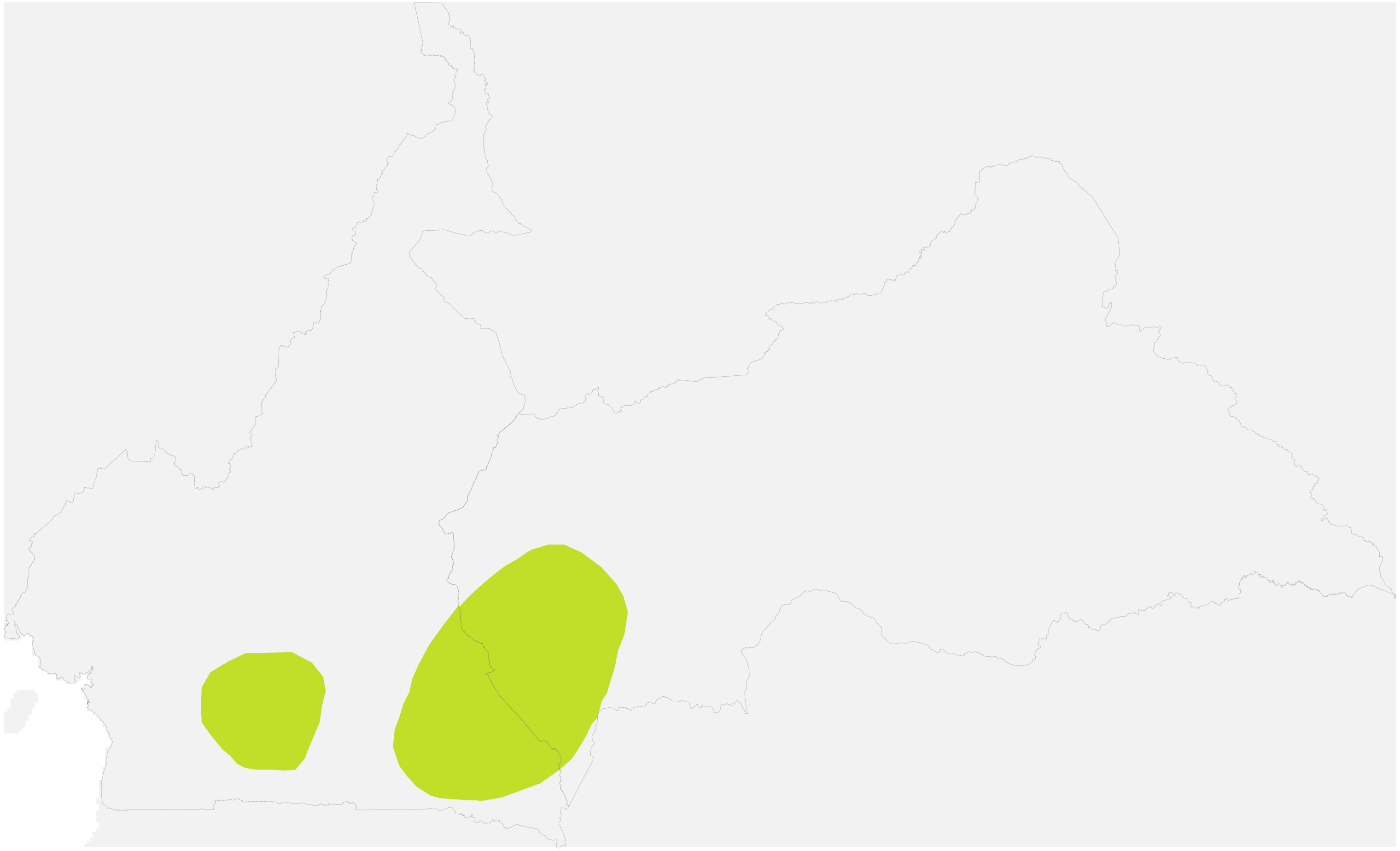

Current EOR

Atheris chlorechis

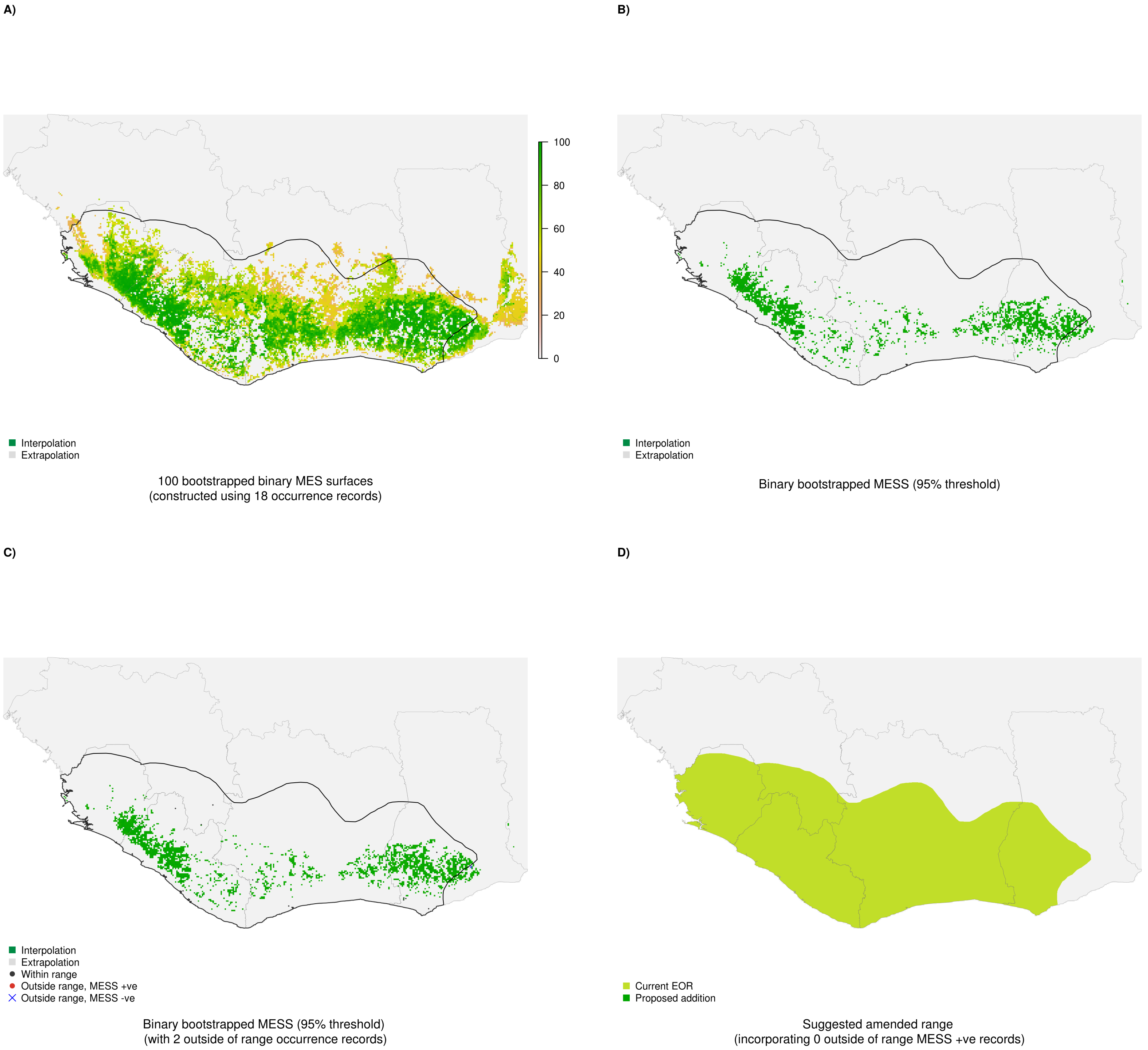

Atheris squamigera

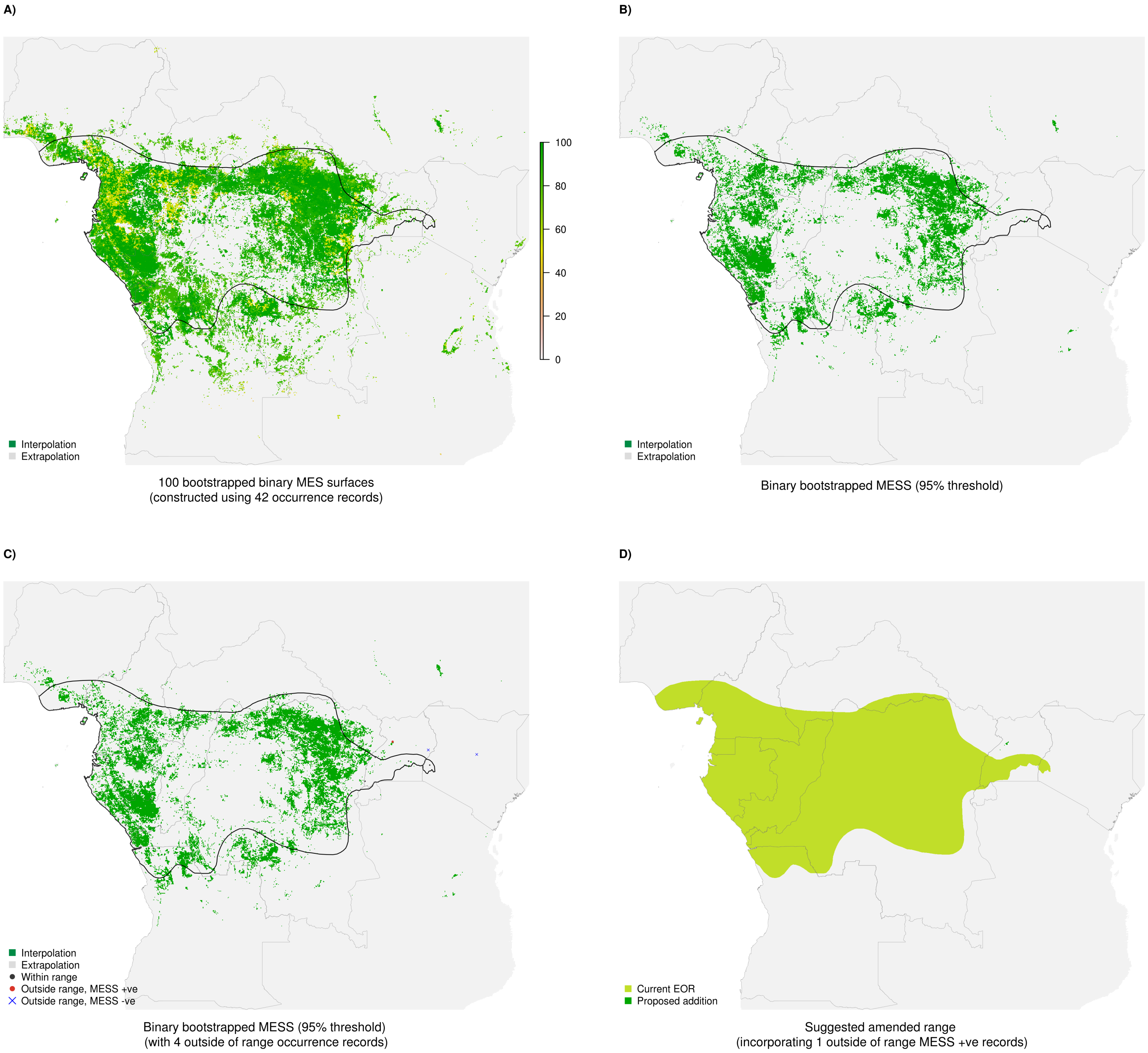

Atractaspis andersonii

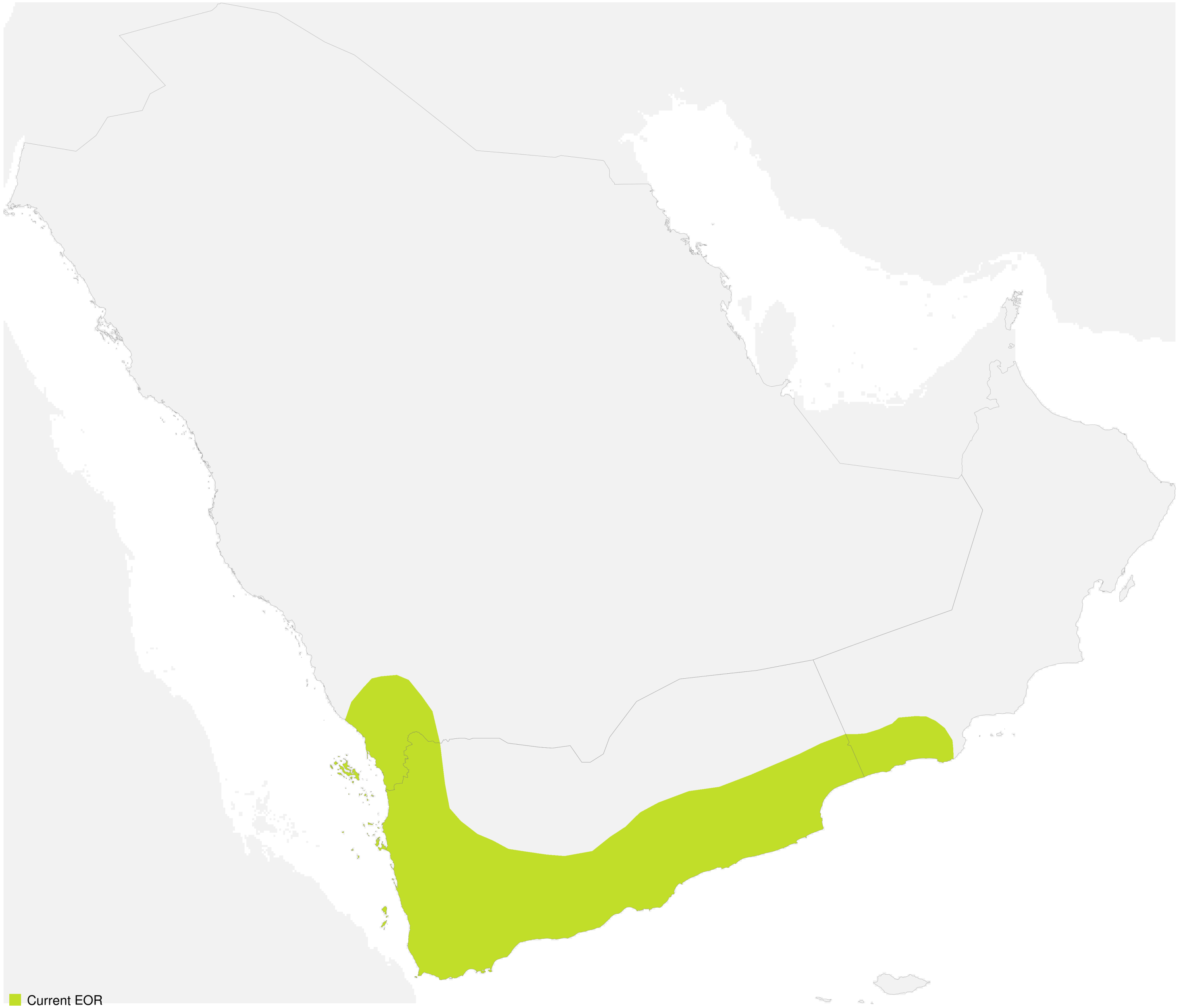

Atractaspis bibronii

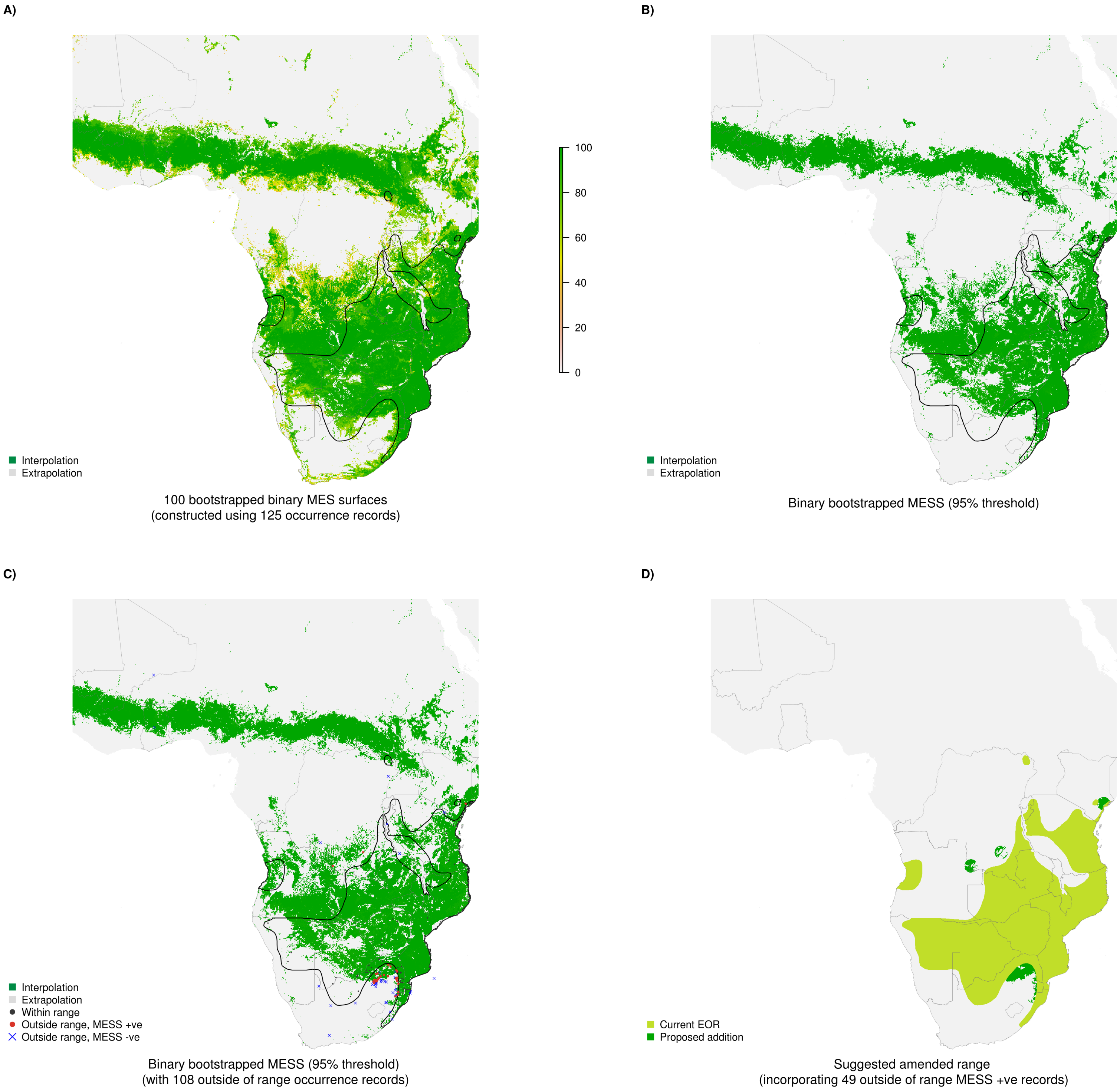

Atractaspis engaddensis

A)

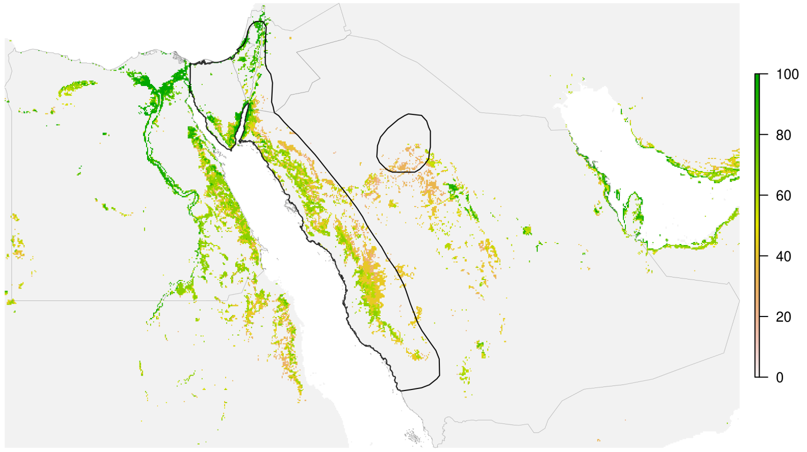

100 bootstrapped binary MES surfaces
(constructed using 26 occurrence records)

B)

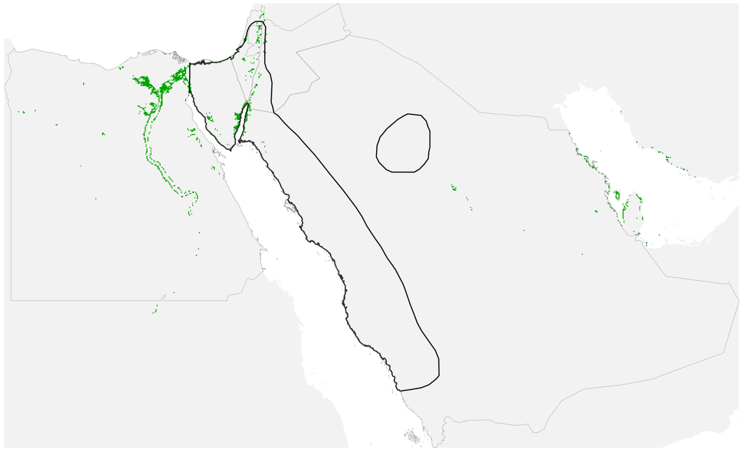

Binary bootstrapped MESS (95% threshold)

C)

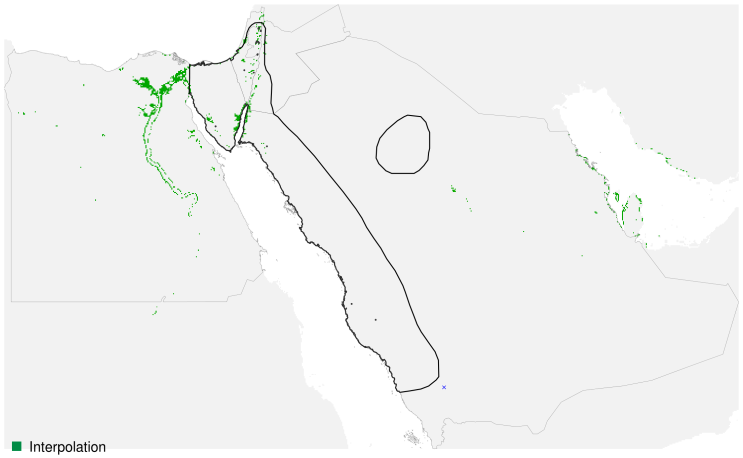

Binary bootstrapped MESS (95% threshold)
(with 1 outside of range occurrence records)

D)

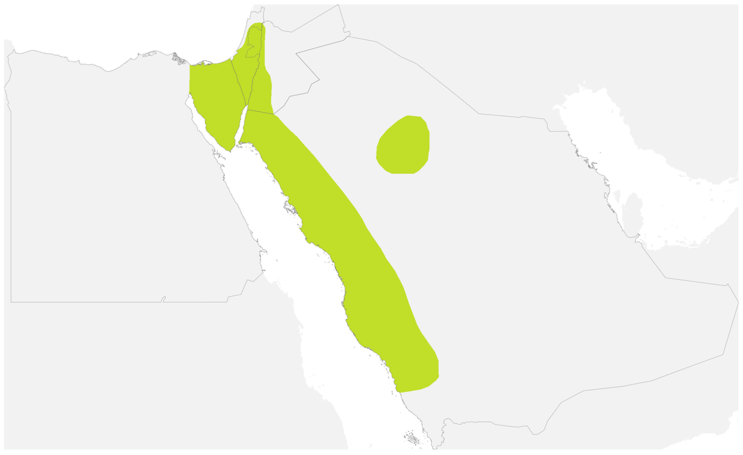

Suggested amended range
(incorporating 0 outside of range MESS +ve records)

Atractaspis fallax

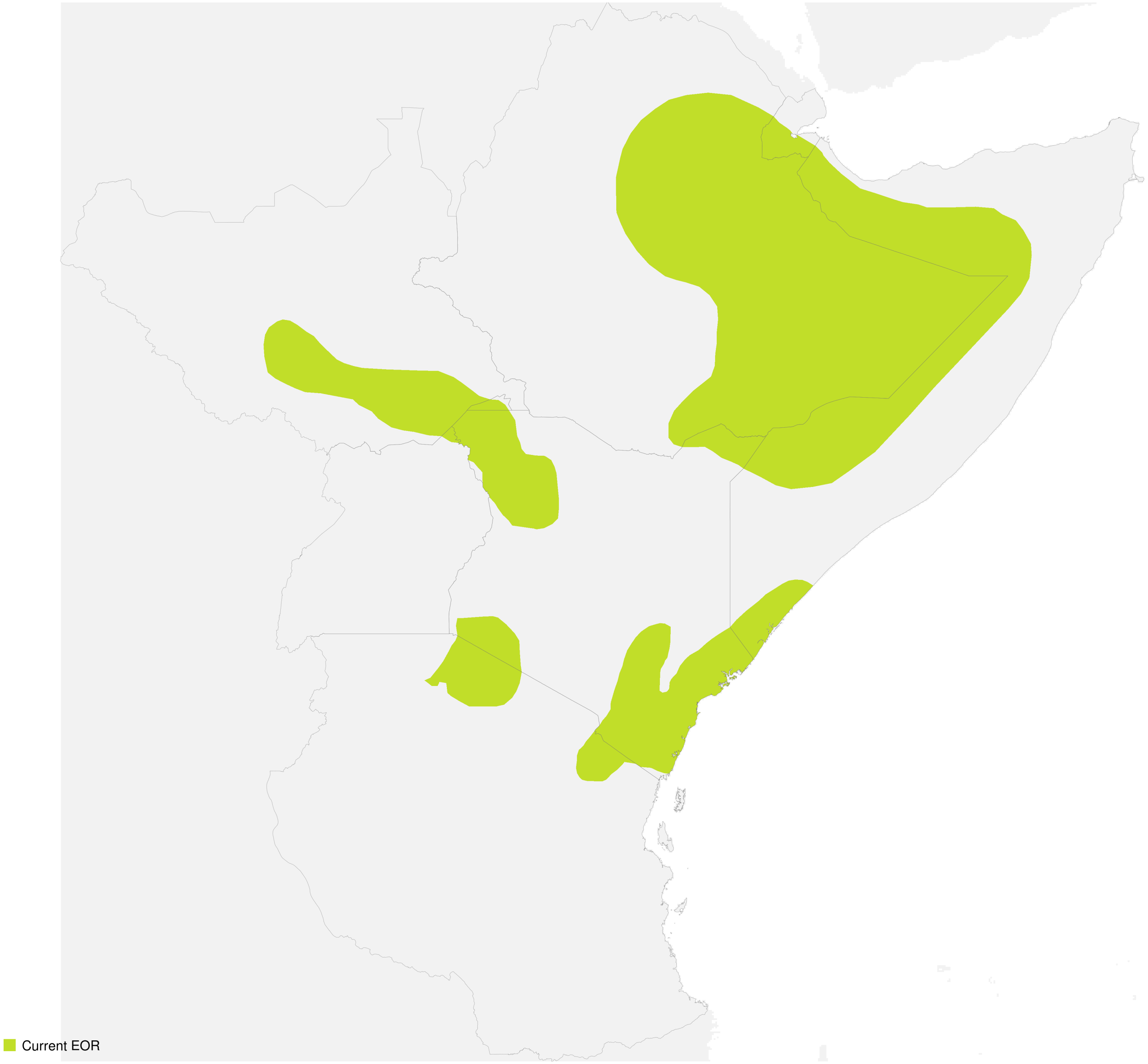

Atractaspis irregularis

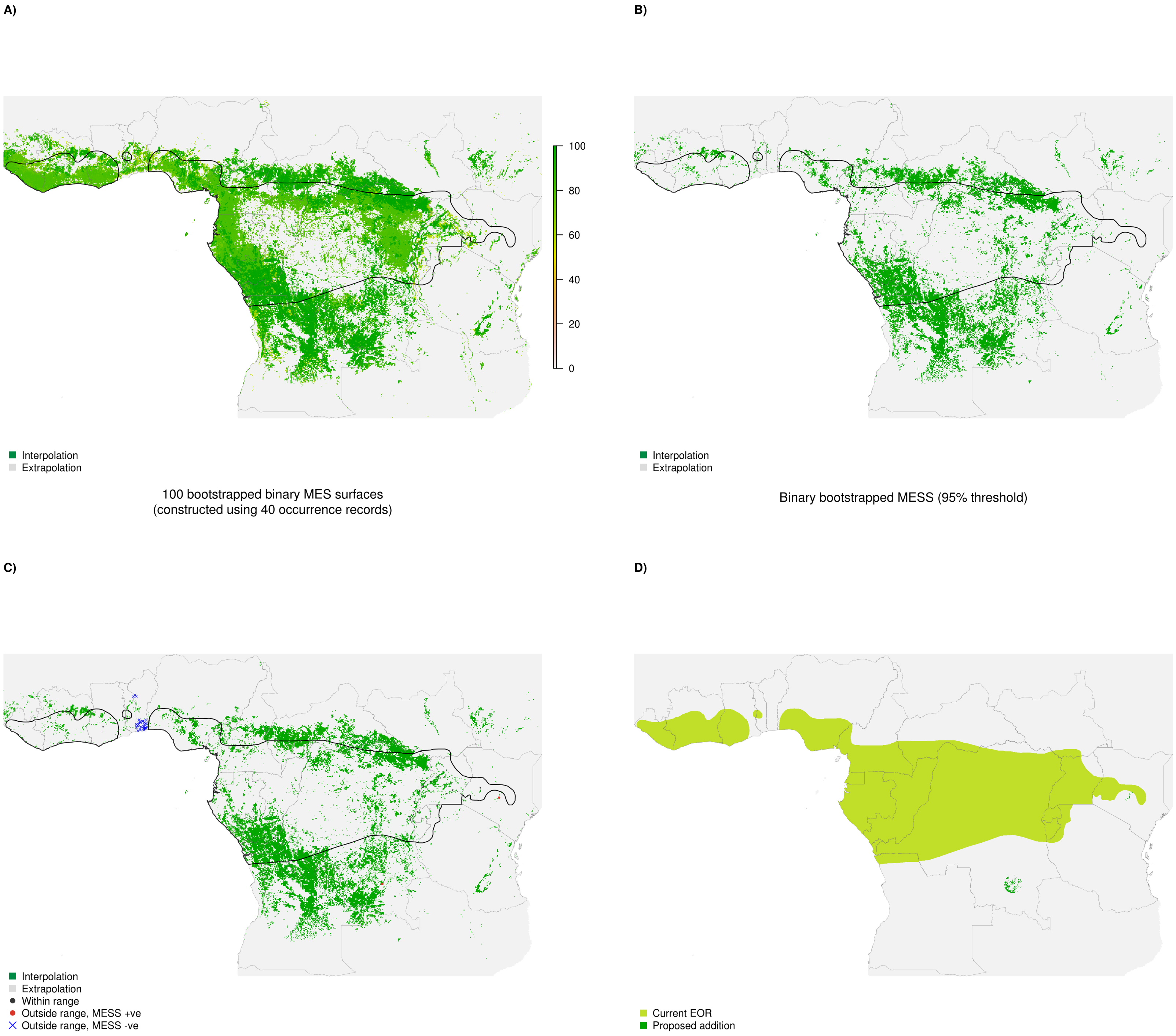

A) 100 bootstrapped binary MES surfaces (constructed using 40 occurrence records)

B) Binary bootstrapped MESS (95% threshold)

C) Binary bootstrapped MESS (95% threshold) (with 37 outside of range occurrence records)

D) Suggested amended range (incorporating 4 outside of range MESS +ve records)

Atropoides indomitus

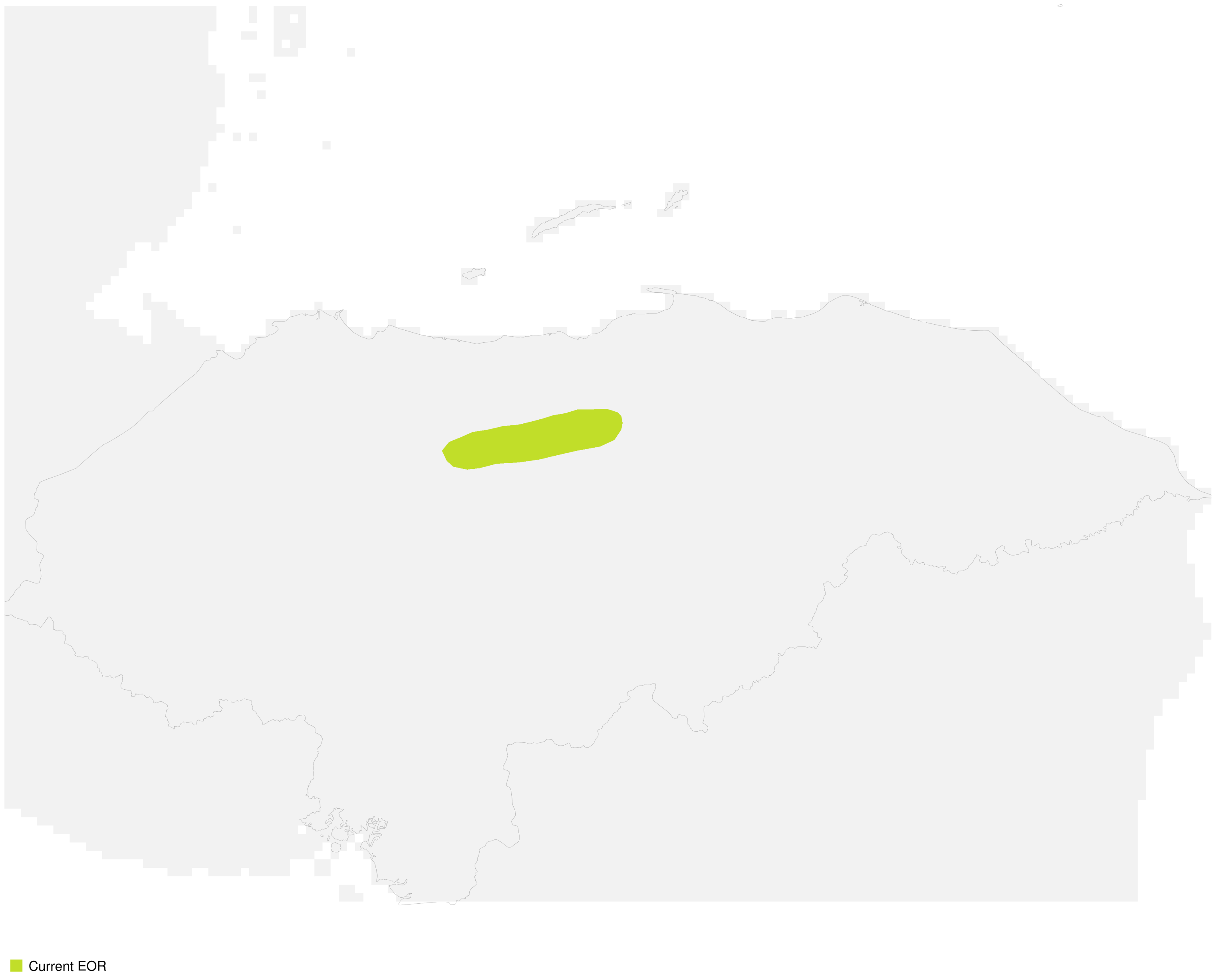

Atropoides mexicanus

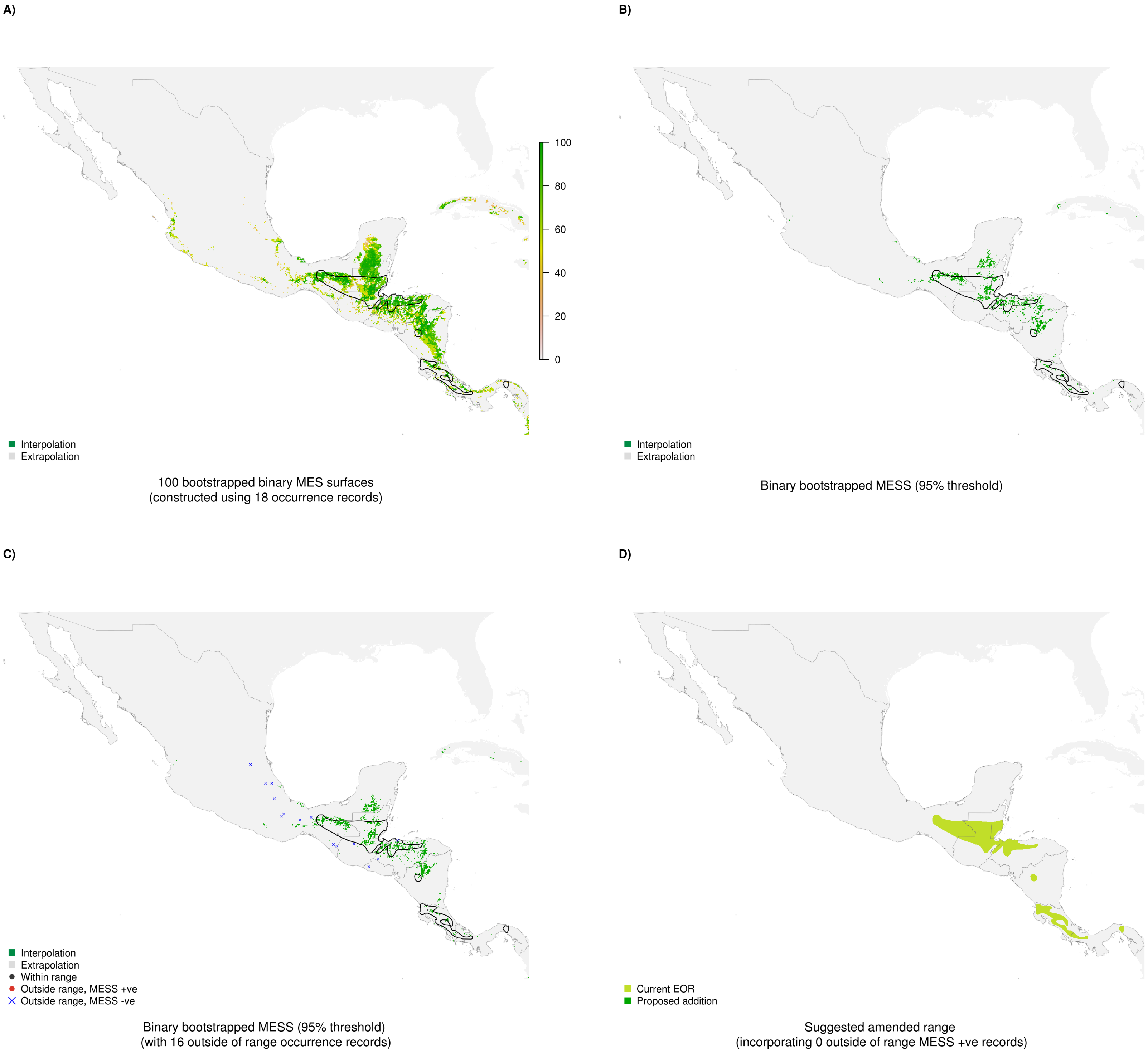

Atropoides nummifer

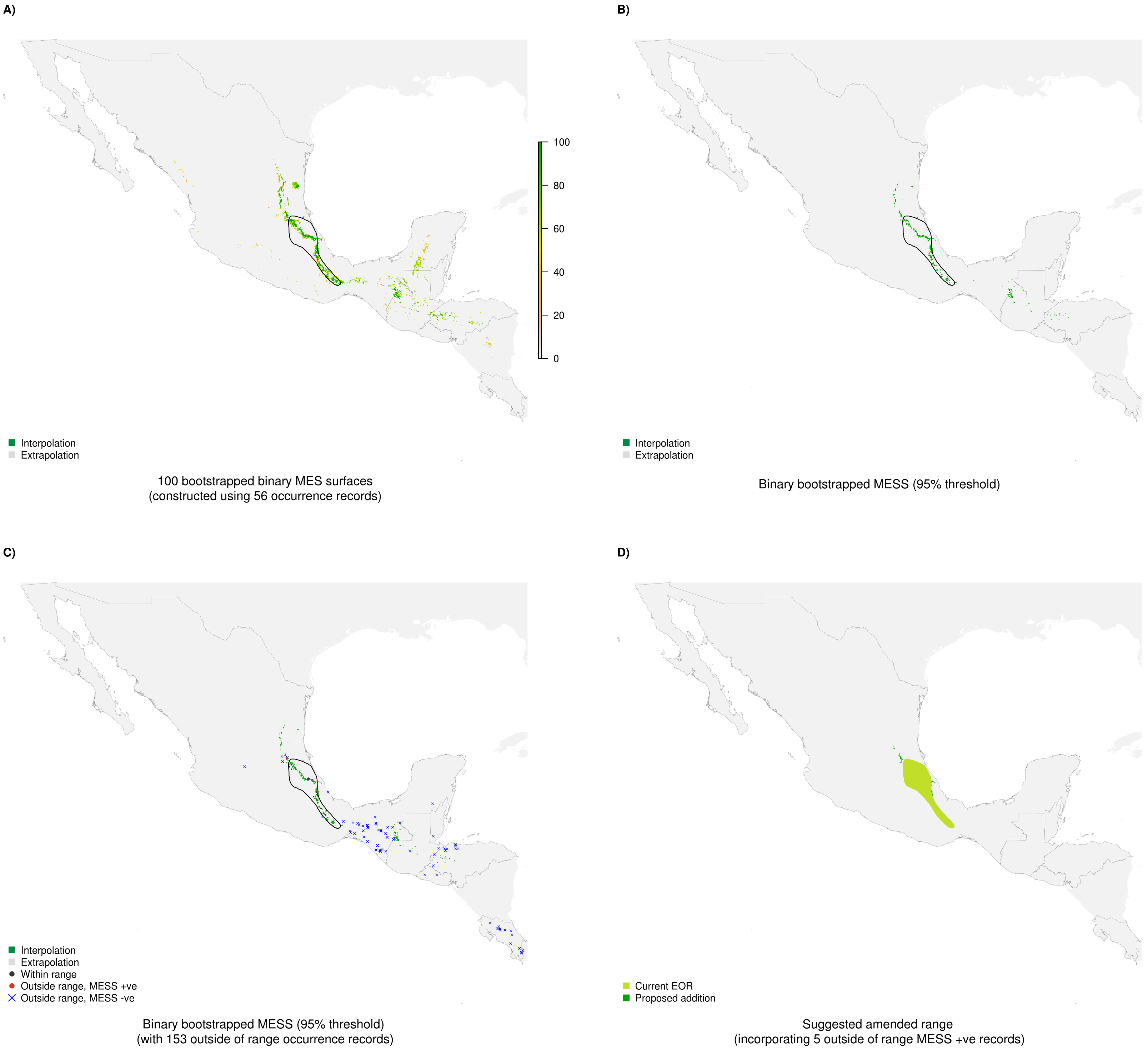

Atropoides occiduus

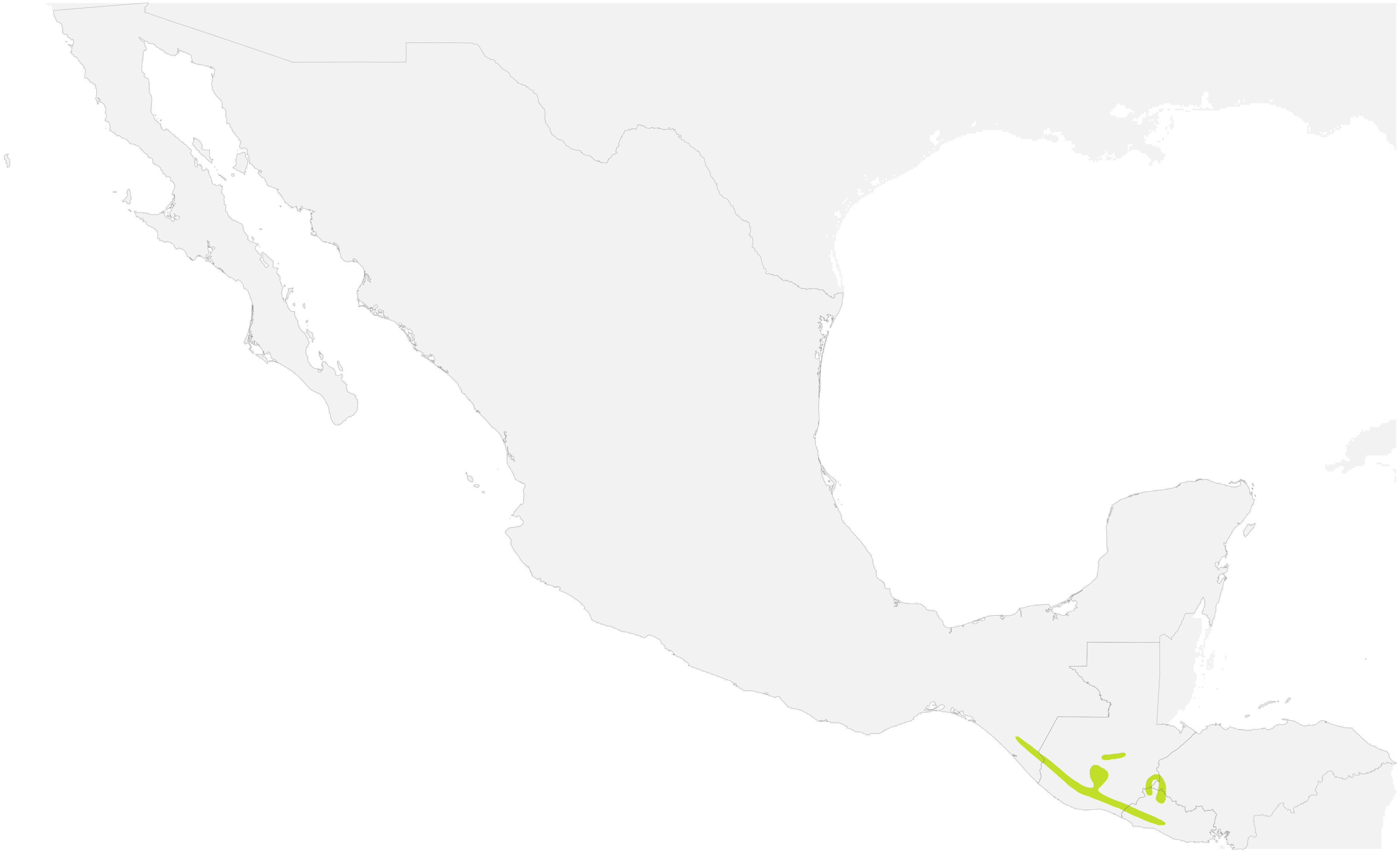

Current EOR

Atropoides olmec

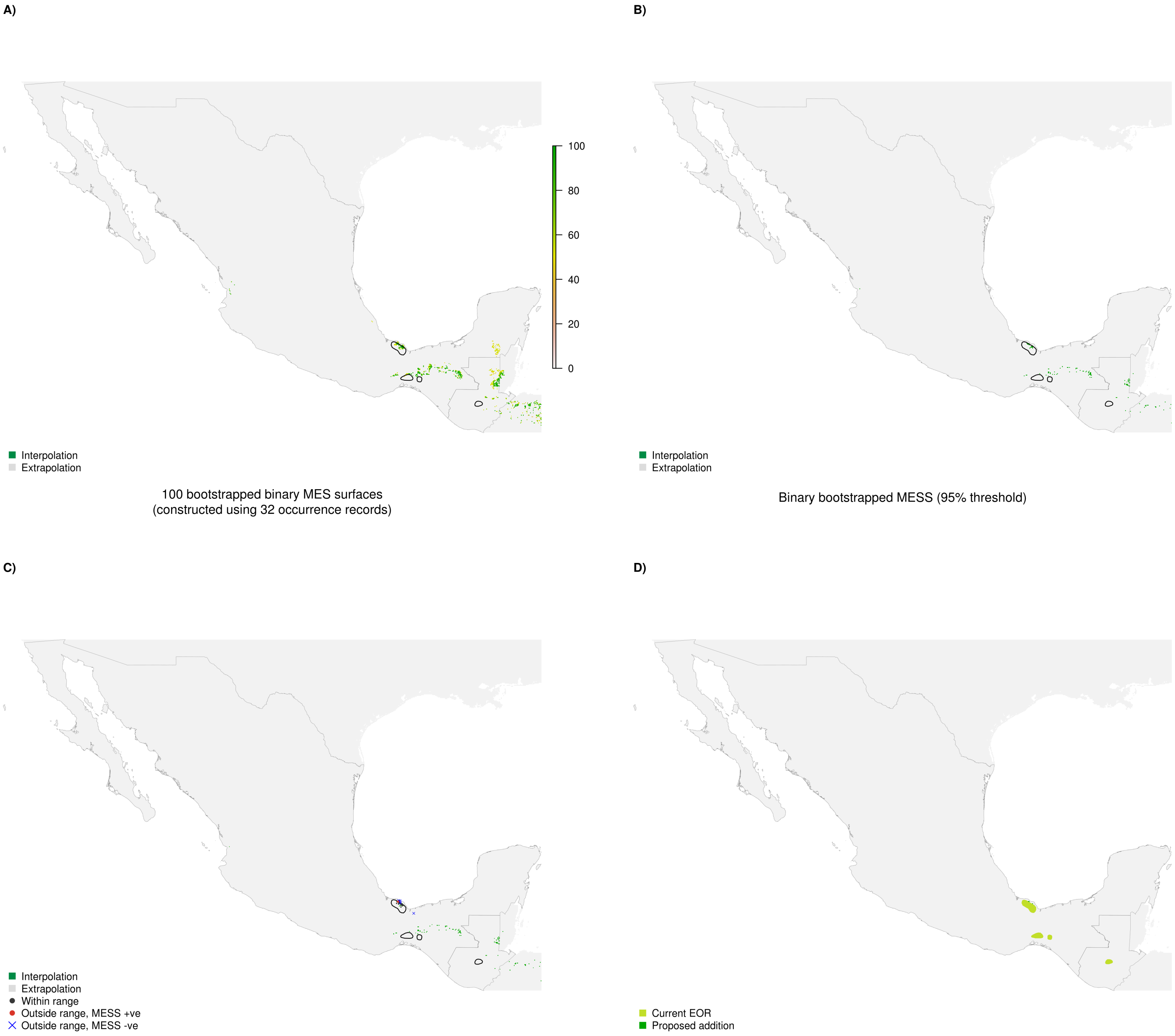

A) 100 bootstrapped binary MES surfaces (constructed using 32 occurrence records)

B) Binary bootstrapped MESS (95% threshold)

C) Binary bootstrapped MESS (95% threshold) (with 9 outside of range occurrence records)

D) Suggested amended range (incorporating 1 outside of range MESS +ve records)

Atropoides picadoi

A)

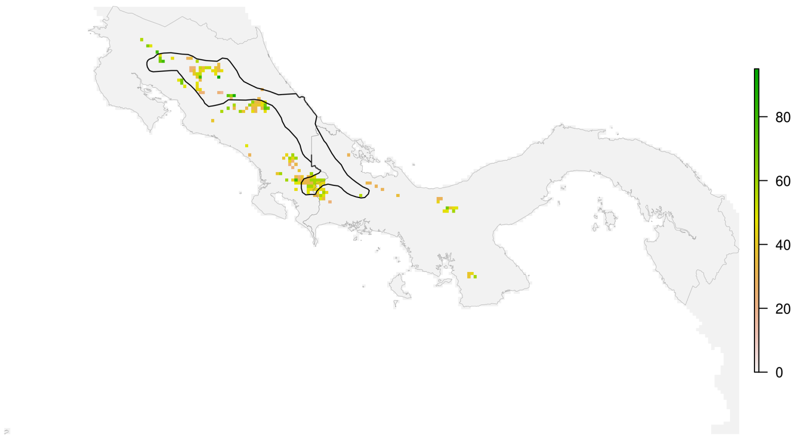

Interpolation
Extrapolation

100 bootstrapped binary MES surfaces
(constructed using 7 occurrence records)

B)

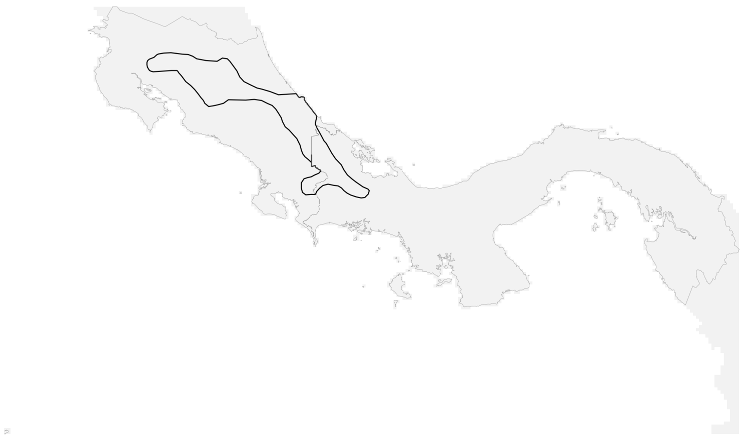

Interpolation
Extrapolation

Binary bootstrapped MESS (95% threshold)
[0 cells met this threshold]

B)

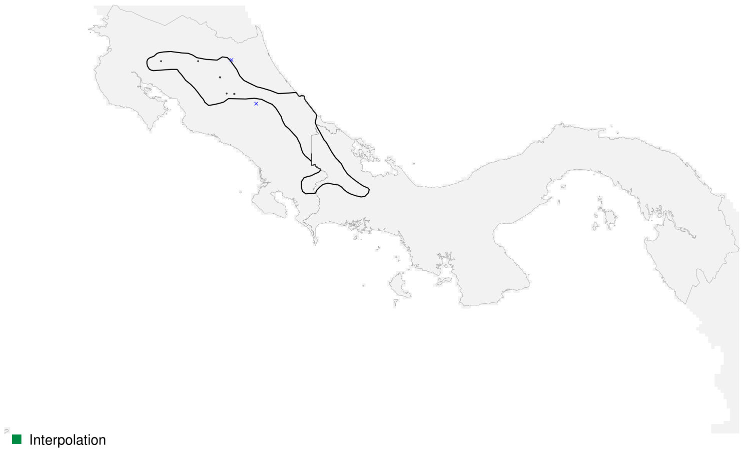

Interpolation
Extrapolation
Within range
Outside range, MESS +ve
Outside range, MESS -ve

Binary bootstrapped MESS (95% threshold)
(with 2 outside of range occurrence records)

D)

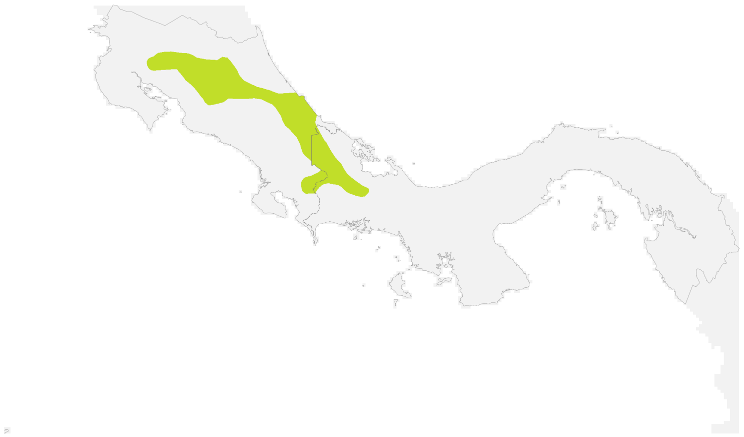

Current EOR
Proposed addition

Suggested amended range
(incorporating 0 outside of range MESS +ve records)

Austrelaps labialis

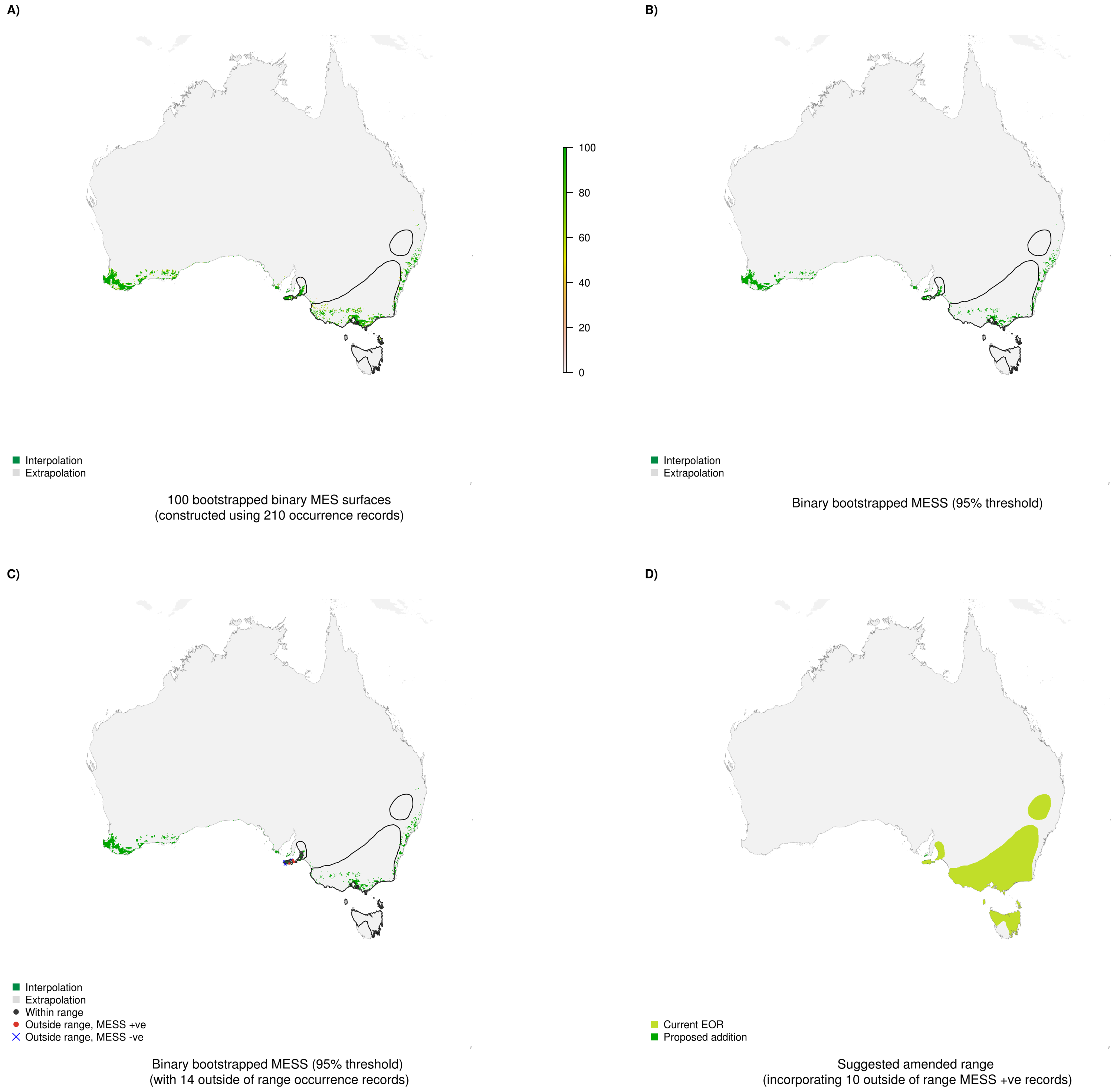

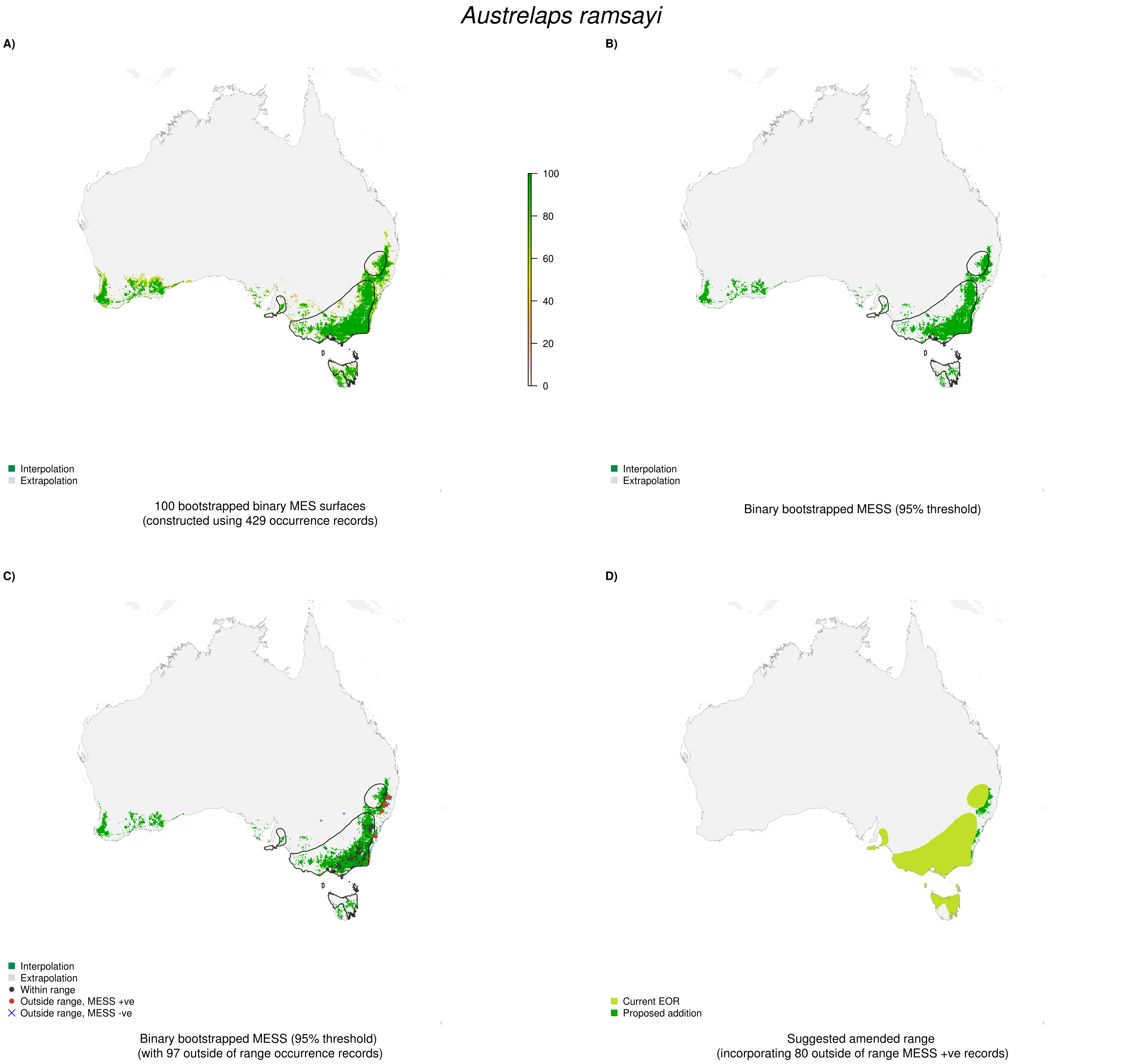
Austrelaps ramsayi
A)
100
80
60
40
20
0
Interpolation
Extrapolation
100 bootstrapped binary MES surfaces
(constructed using 429 occurrence records)
B)
Interpolation
Extrapolation
Binary bootstrapped MESS (95% threshold)
C)
Interpolation
Extrapolation
Within range
Outside range, MESS +ve
Outside range, MESS -ve
Binary bootstrapped MESS (95% threshold)
(with 97 outside of range occurrence records)
D)
Current EOR
Proposed addition
Suggested amended range
(incorporating 80 outside of range MESS +ve records)

Austrelaps superbus

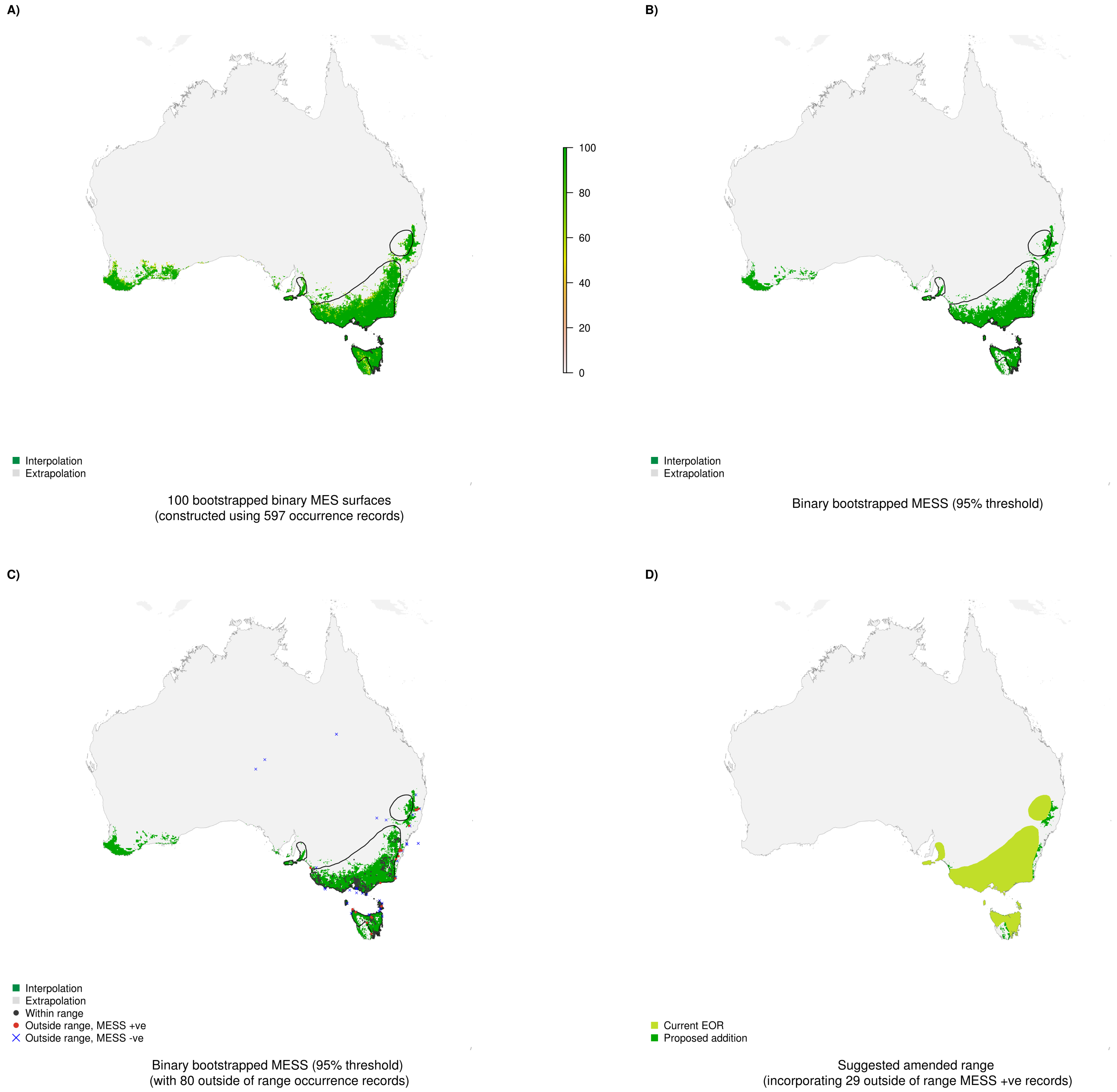

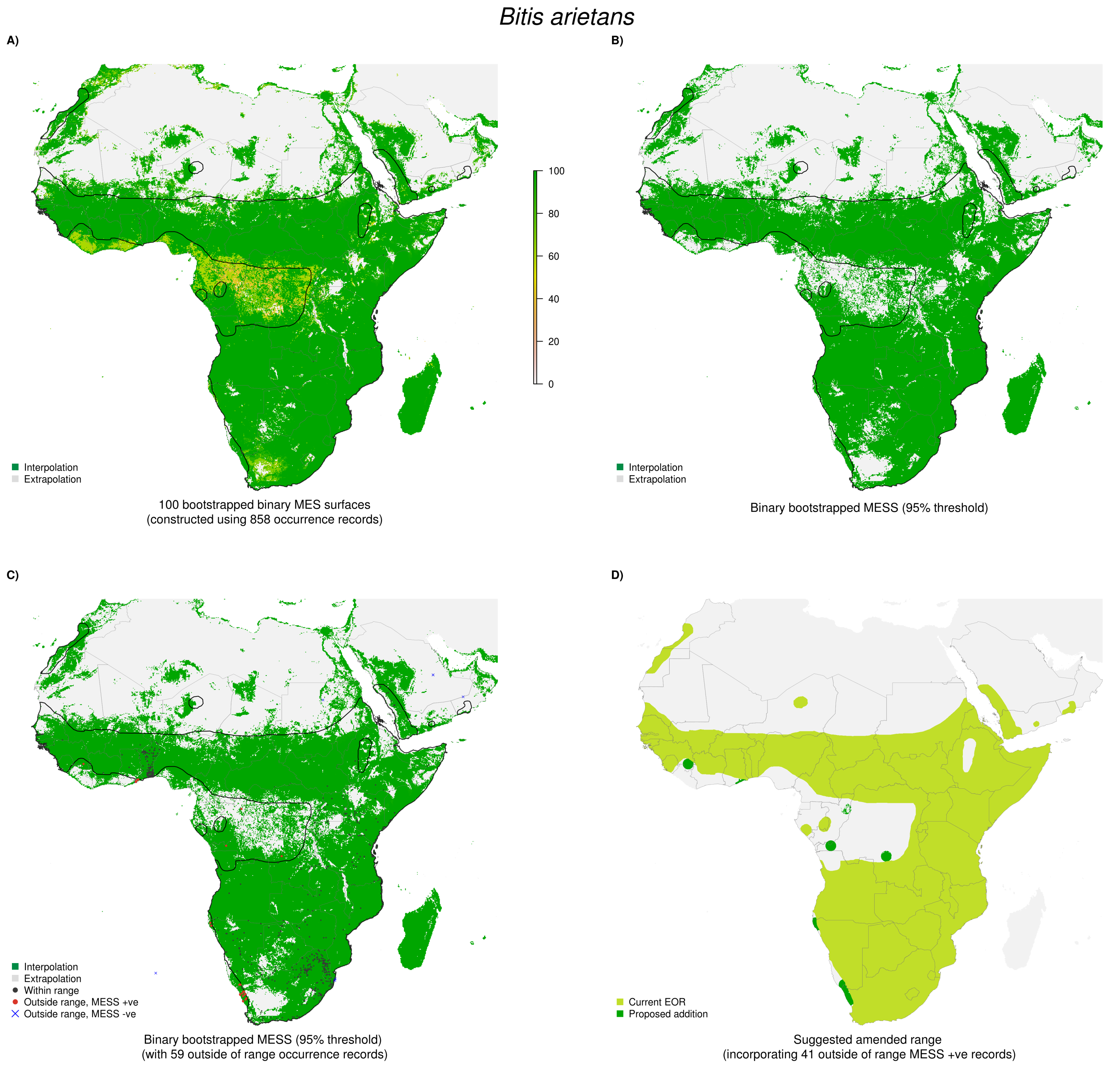
Bitis arietans
A)
B)
100
80
60
40
20
0
Interpolation
Extrapolation
100 bootstrapped binary MES surfaces
(constructed using 858 occurrence records)
Interpolation
Extrapolation
Binary bootstrapped MESS (95% threshold)
C)
D)
Interpolation
Extrapolation
Within range
Outside range, MESS +ve
Outside range, MESS -ve
Binary bootstrapped MESS (95% threshold)
(with 59 outside of range occurrence records)
Current EOR
Proposed addition
Suggested amended range
(incorporating 41 outside of range MESS +ve records)

Bitis gabonica

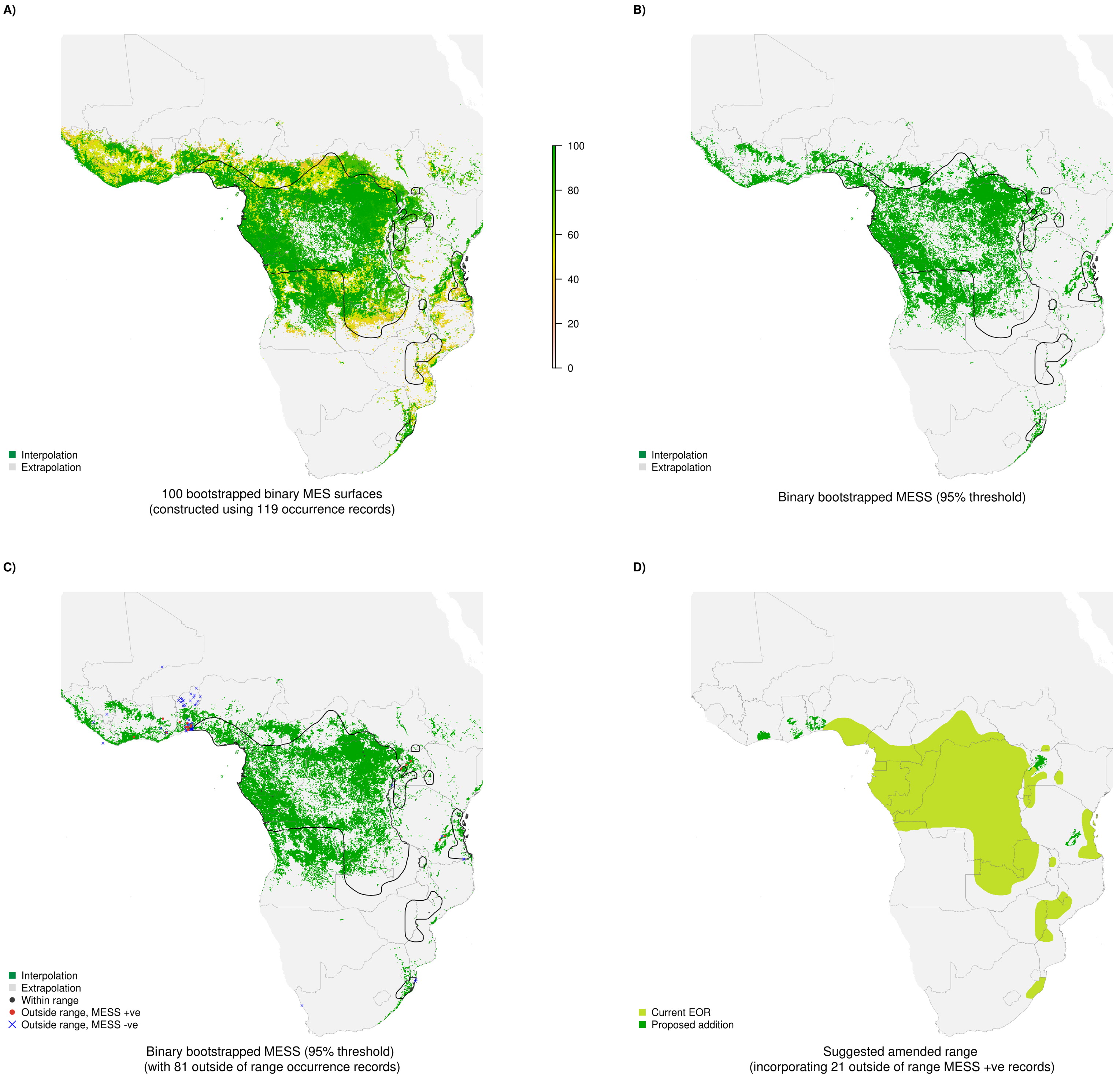

Bitis nasicornis

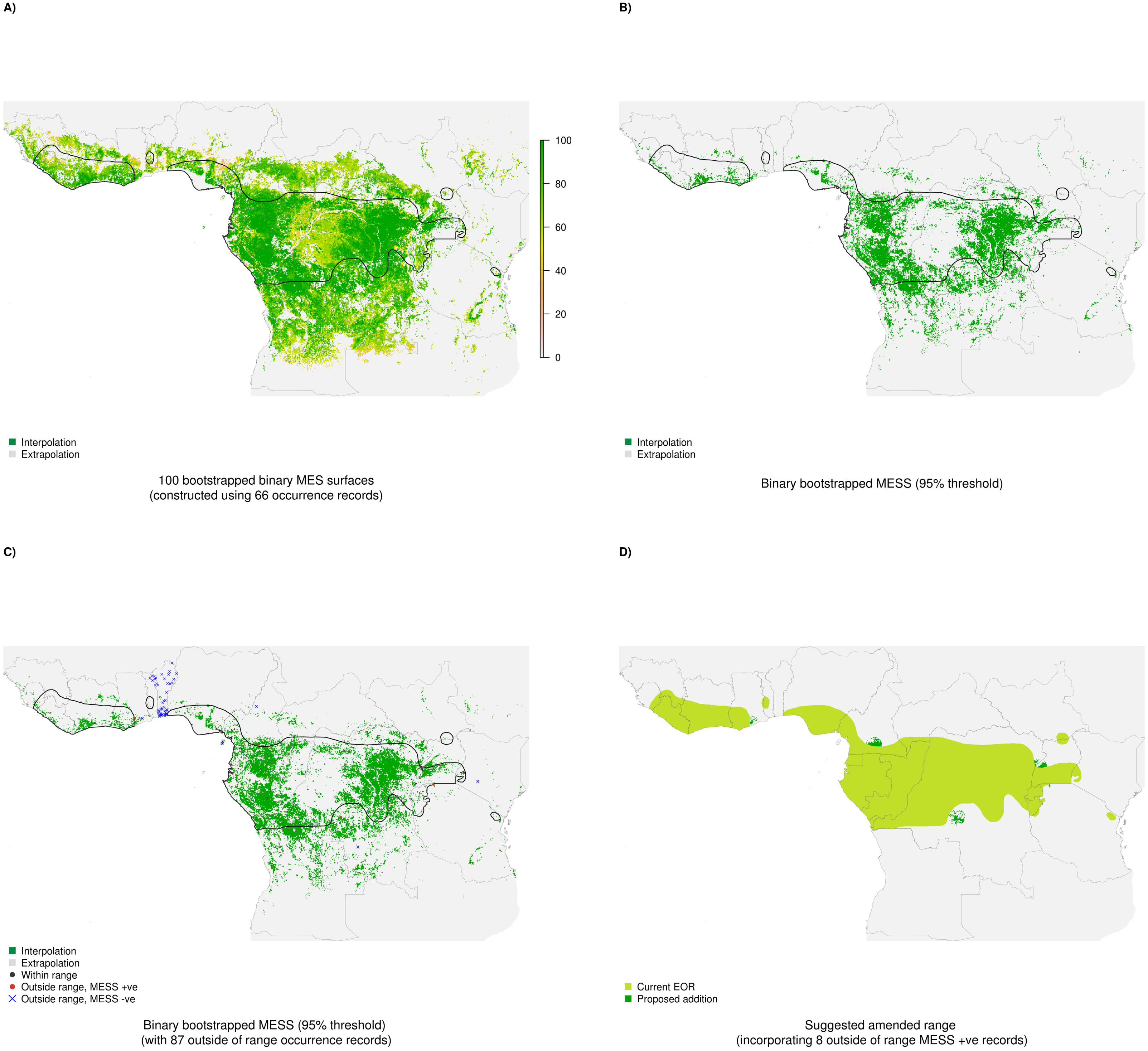

Bitis parviocula

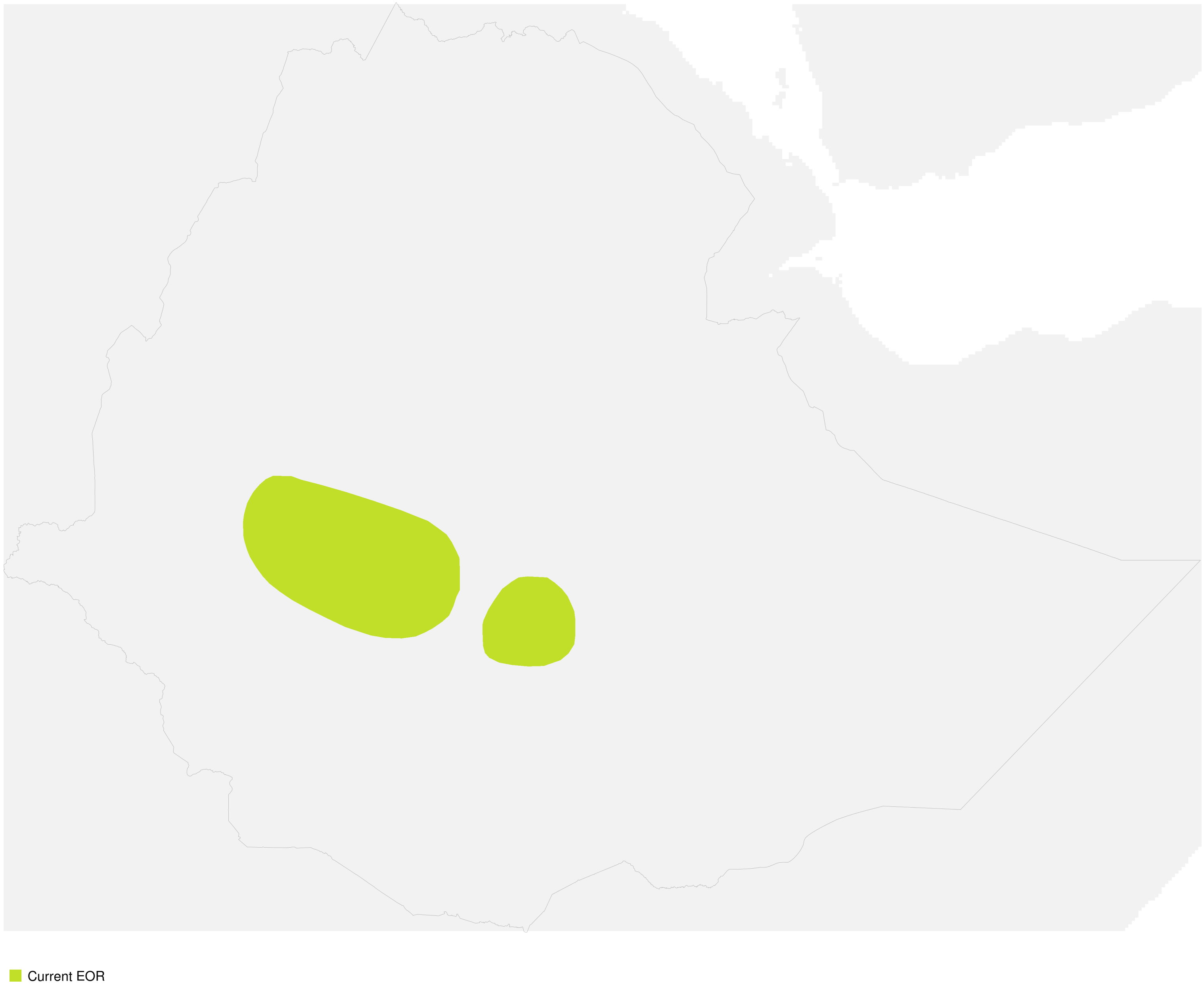

Bitis rhinoceros

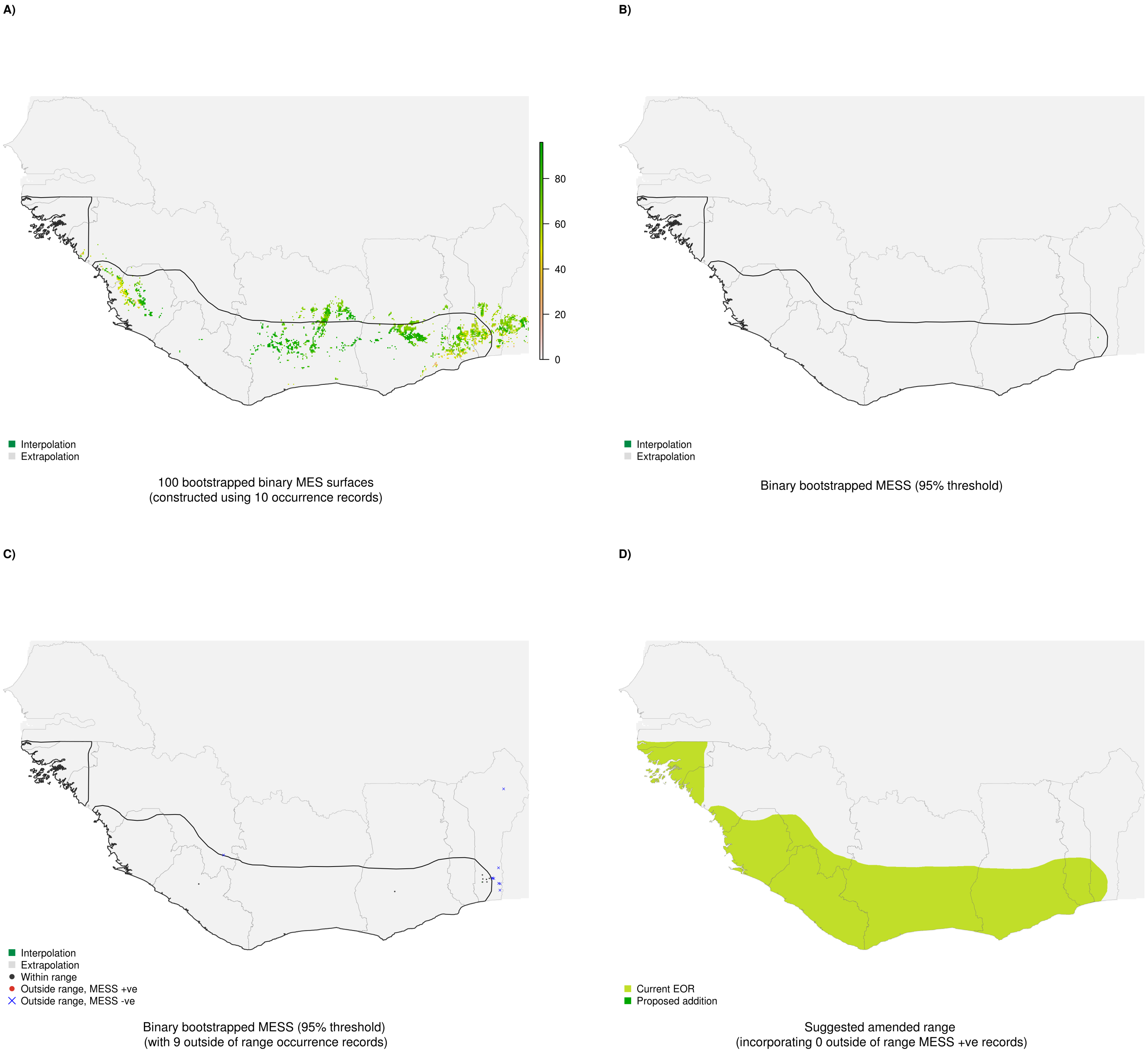

Bothriechis aurifer

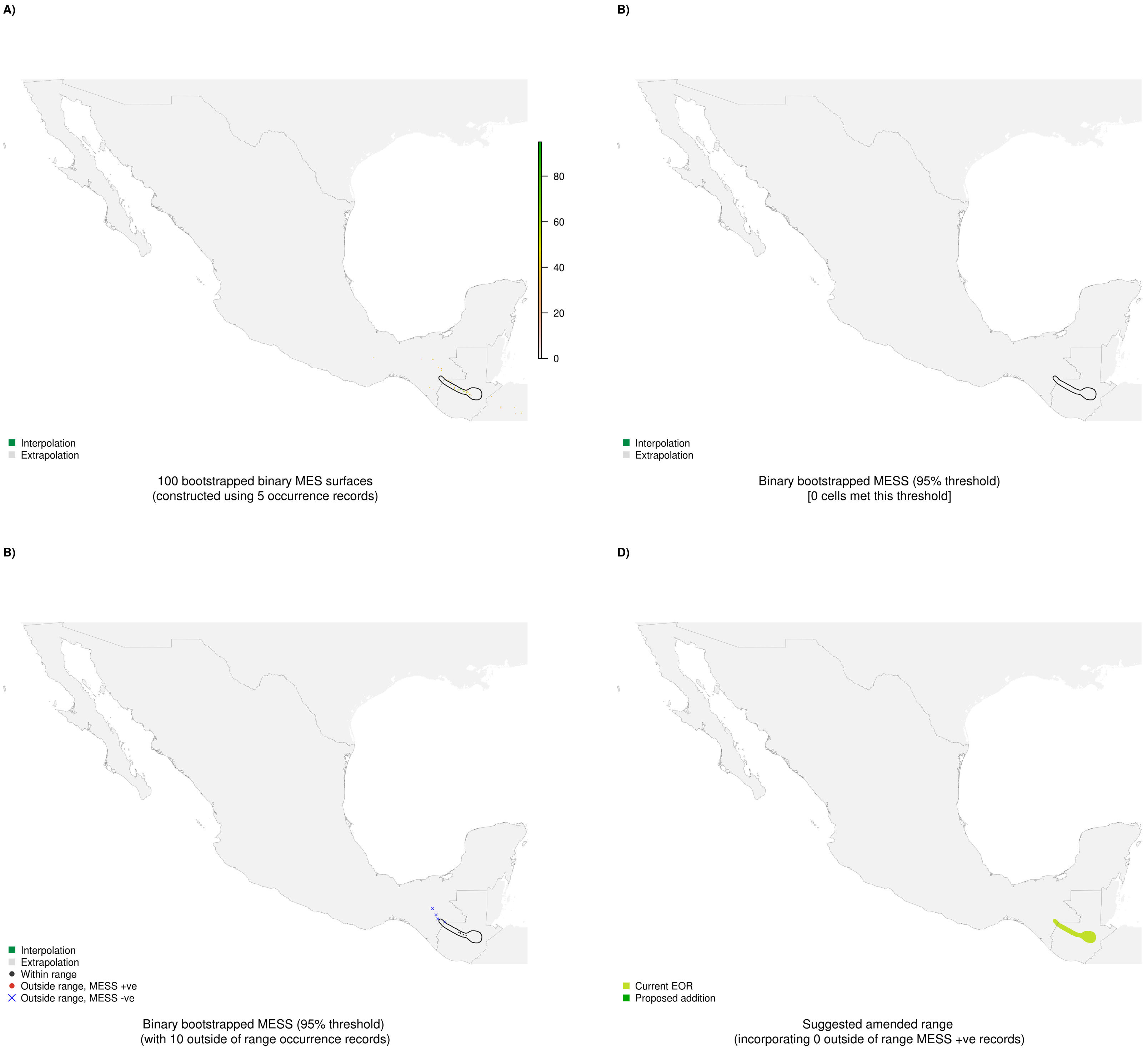

Bothriechis bicolor

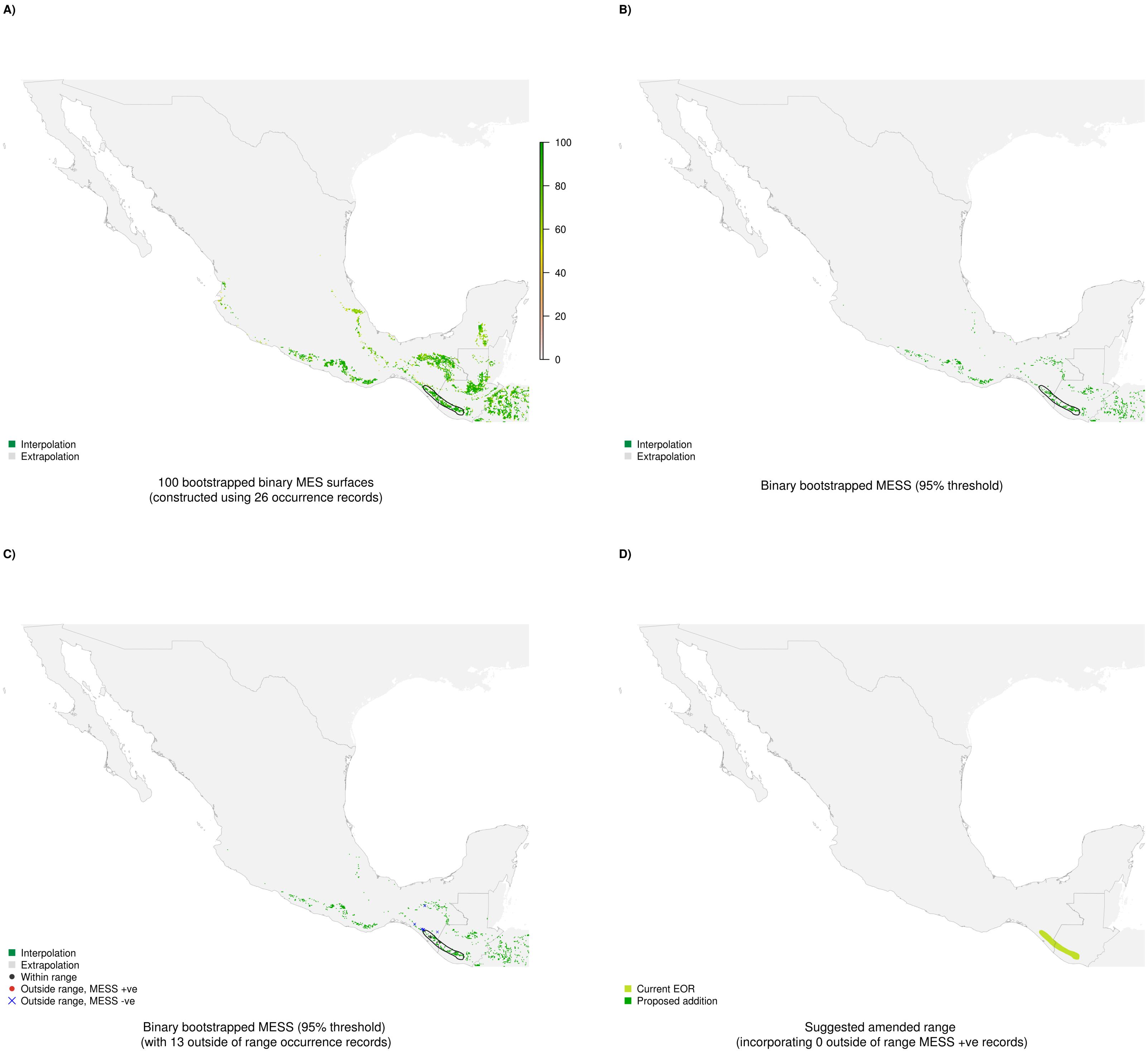

Bothriechis lateralis

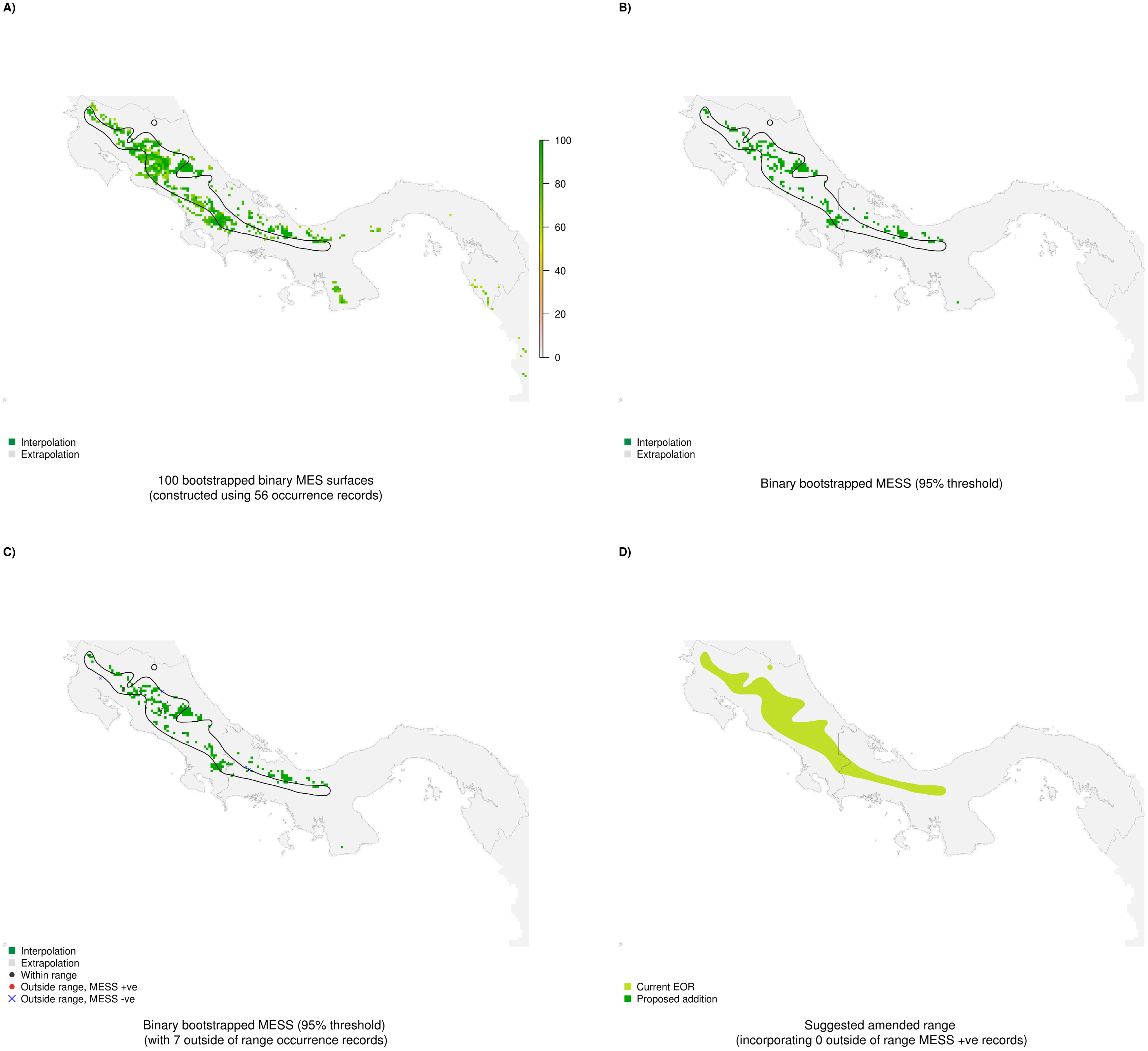

Bothriechis marchi

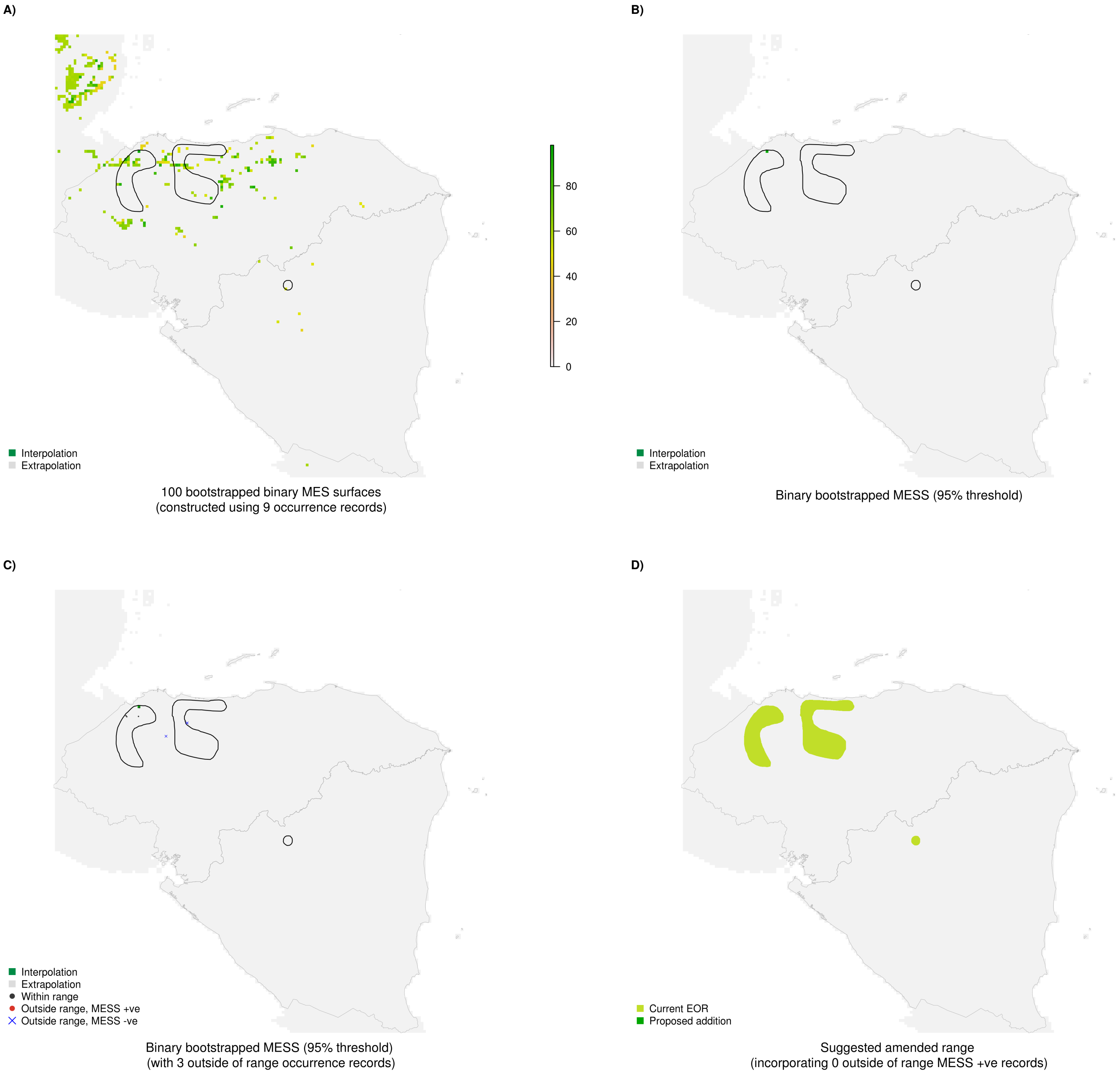

Bothriechis nigroviridis

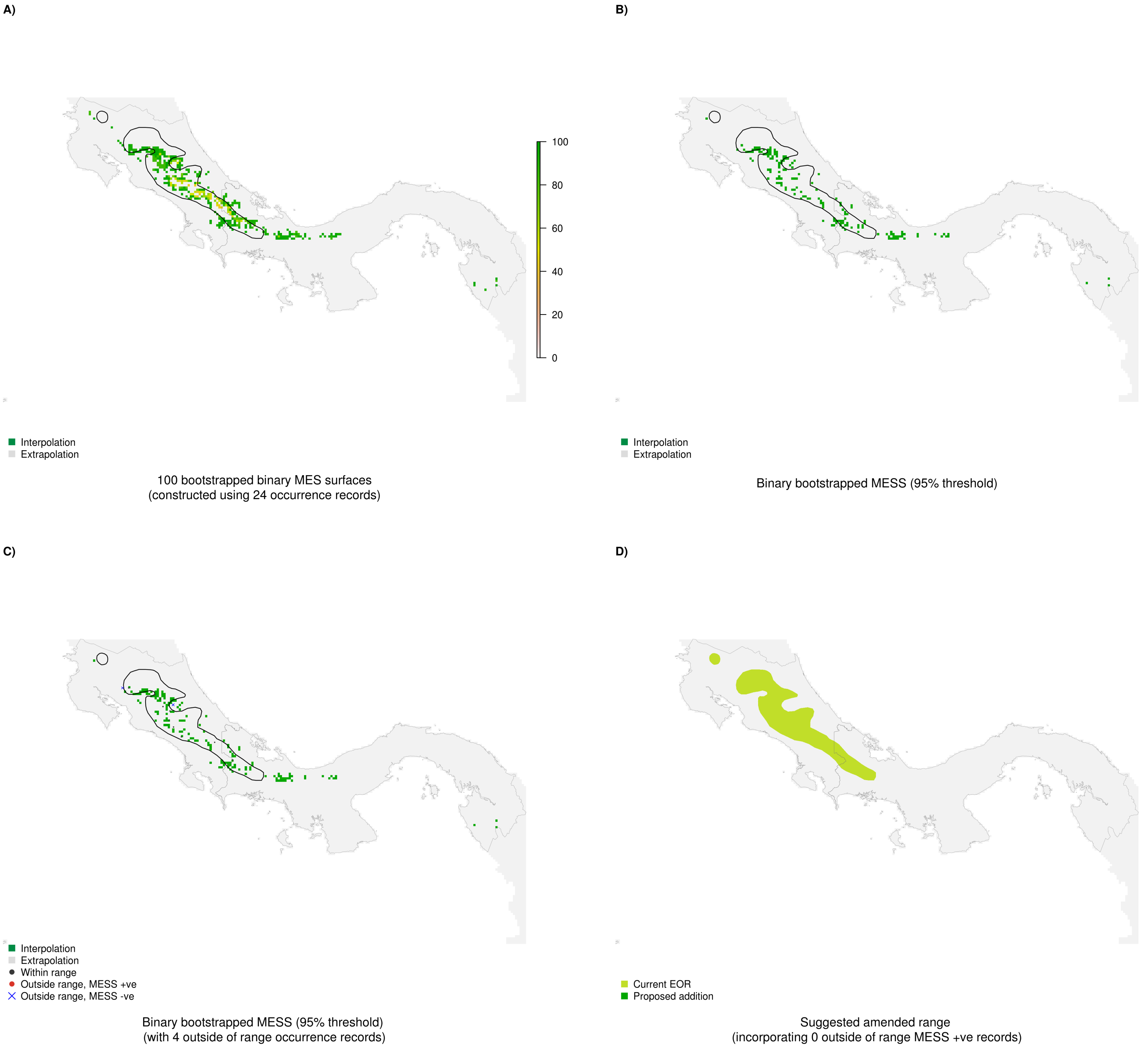

Bothriechis rowleyi

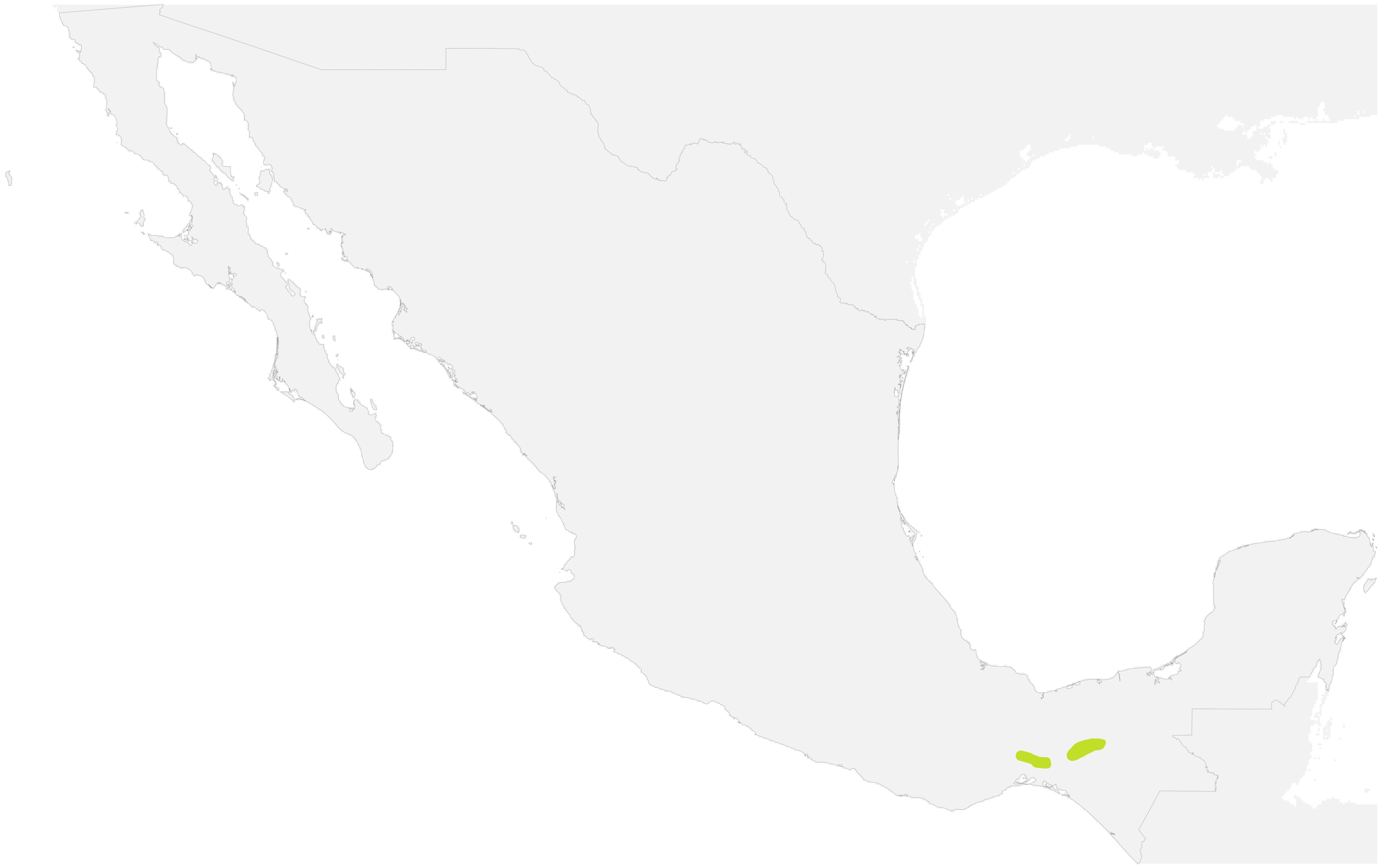

Current EOR

Bothriechis schlegelii

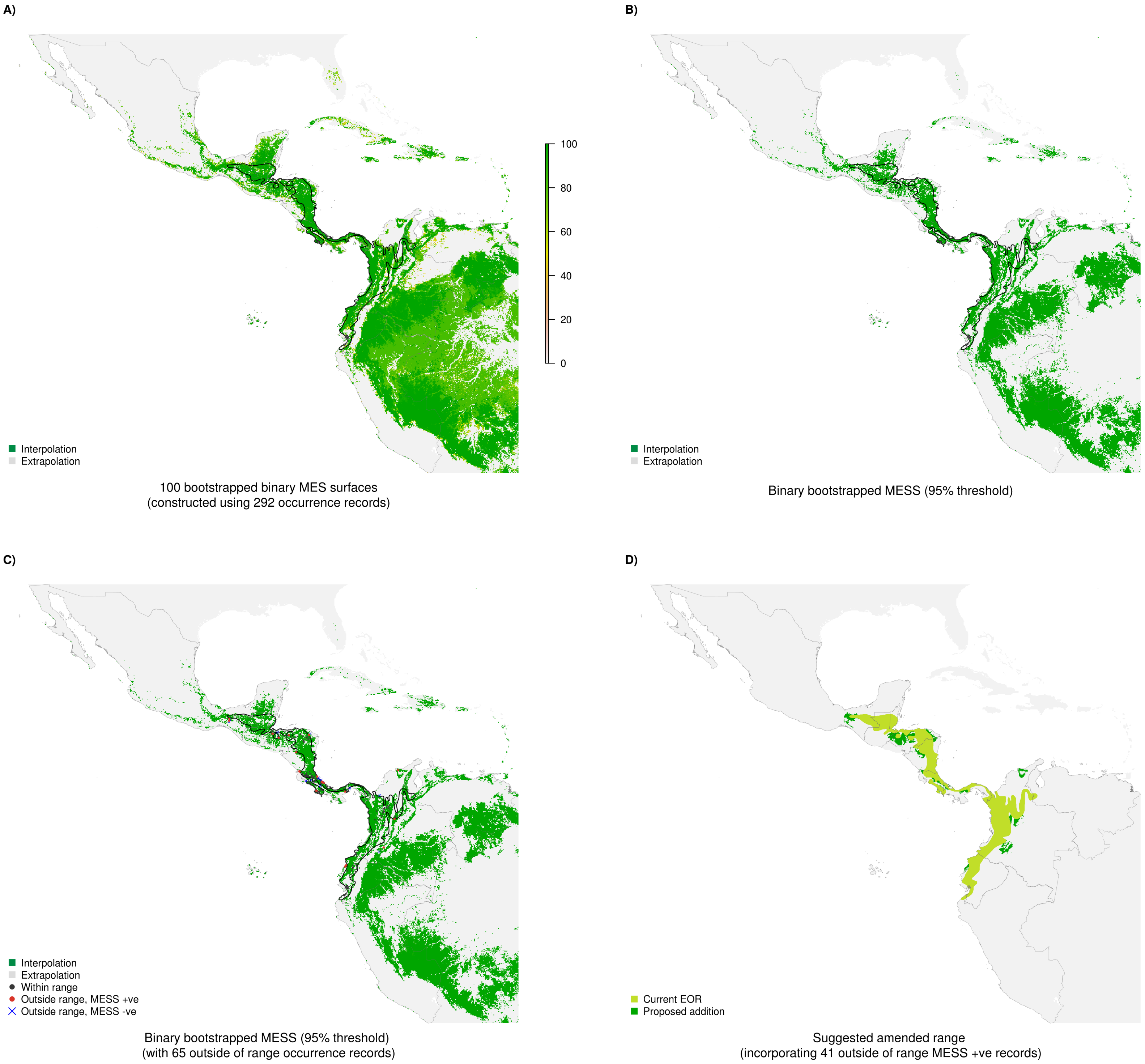

Bothriechis supraciliaris

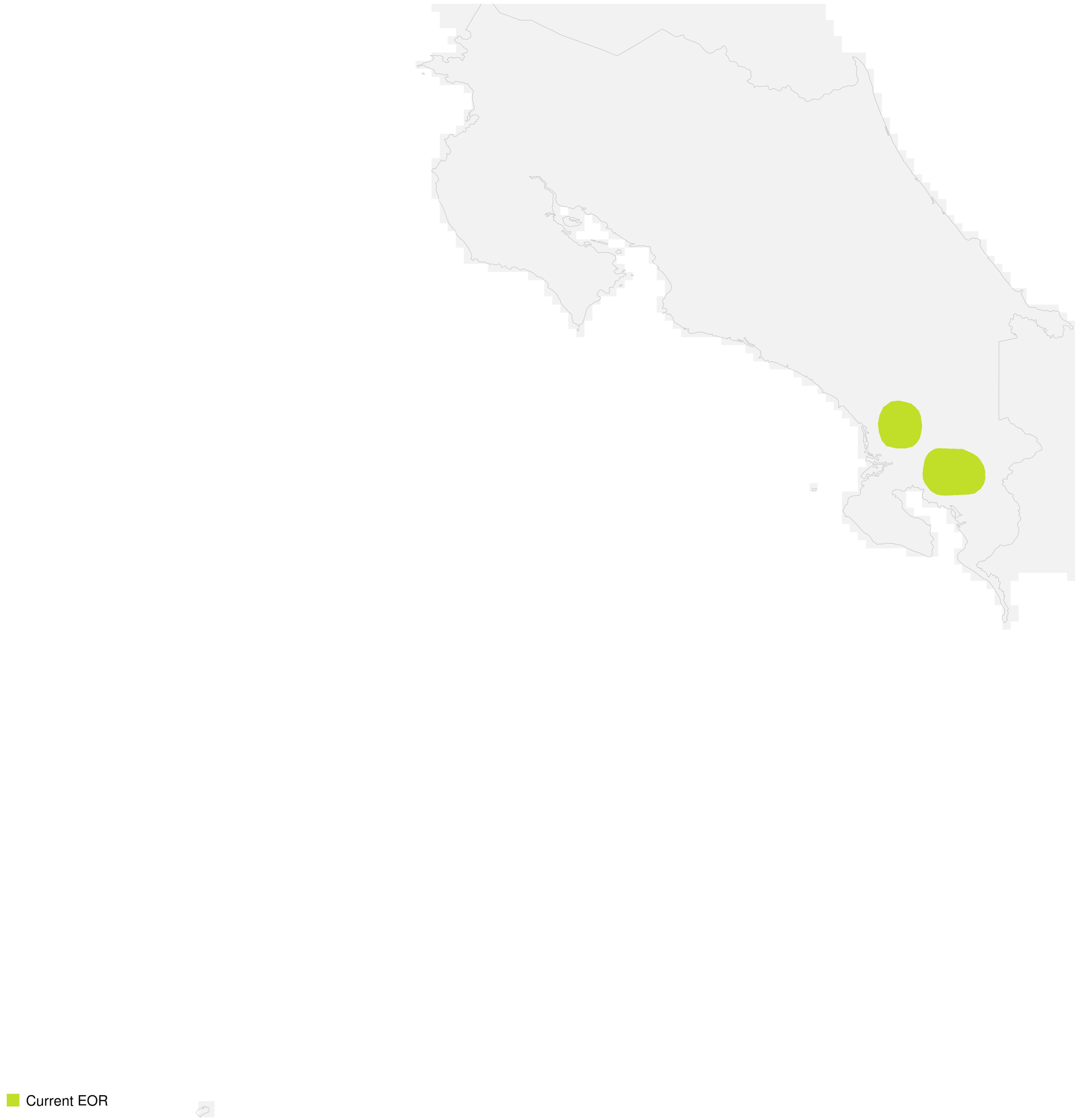

Bothriechis thalassinus

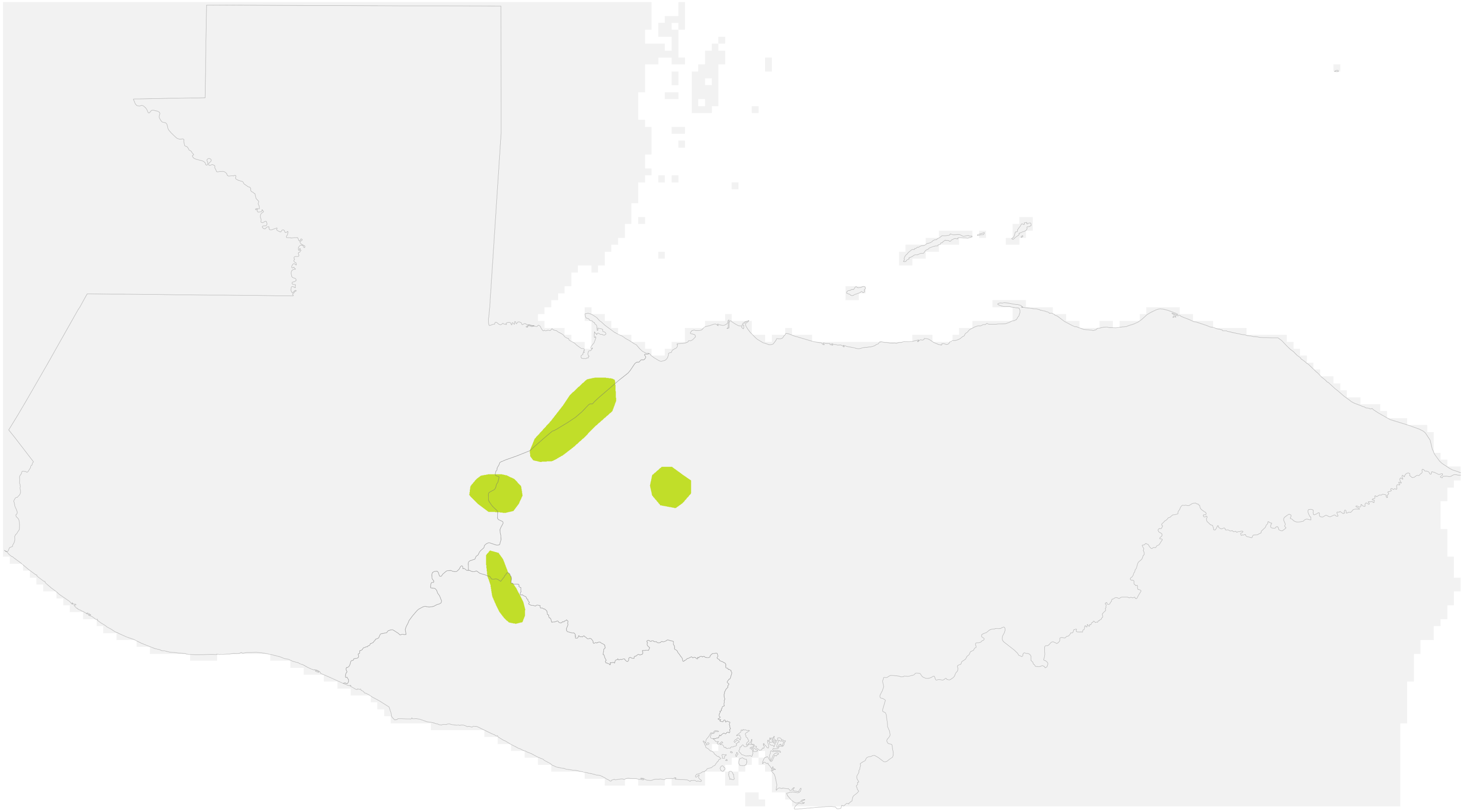

Bothrocophias campbelli

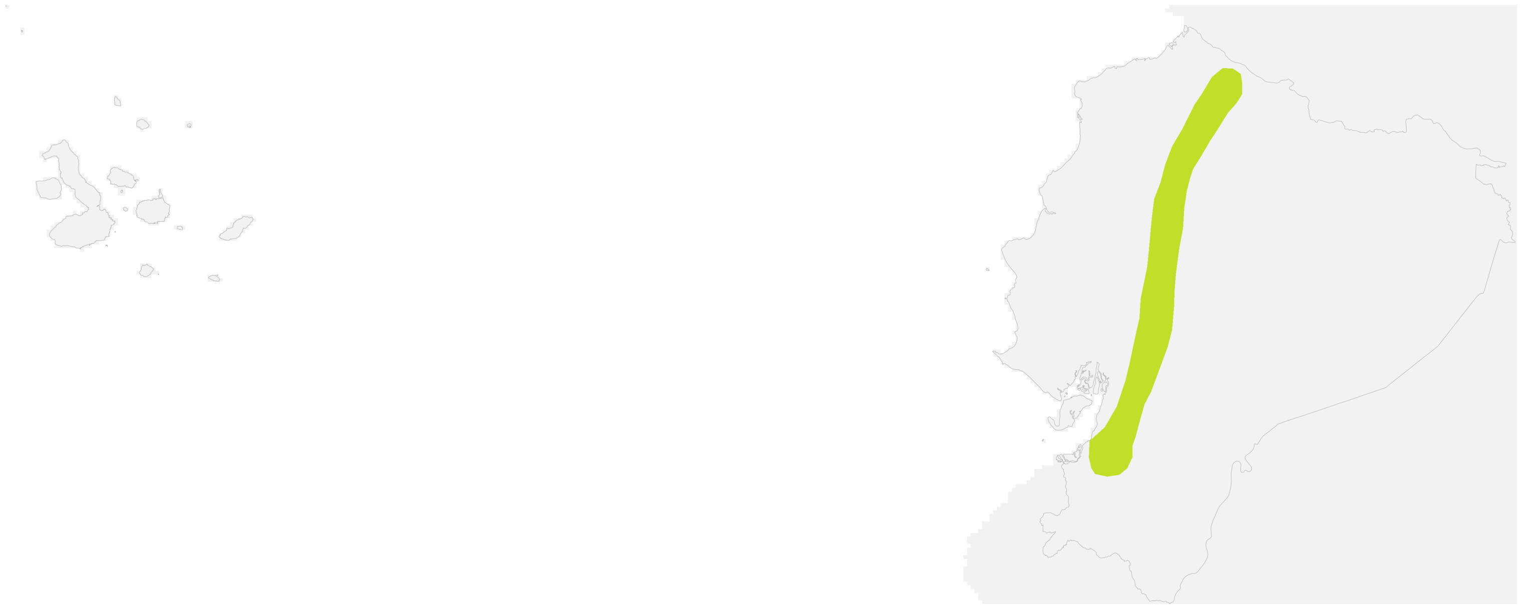

Current EOR

Bothrocophias colombianus

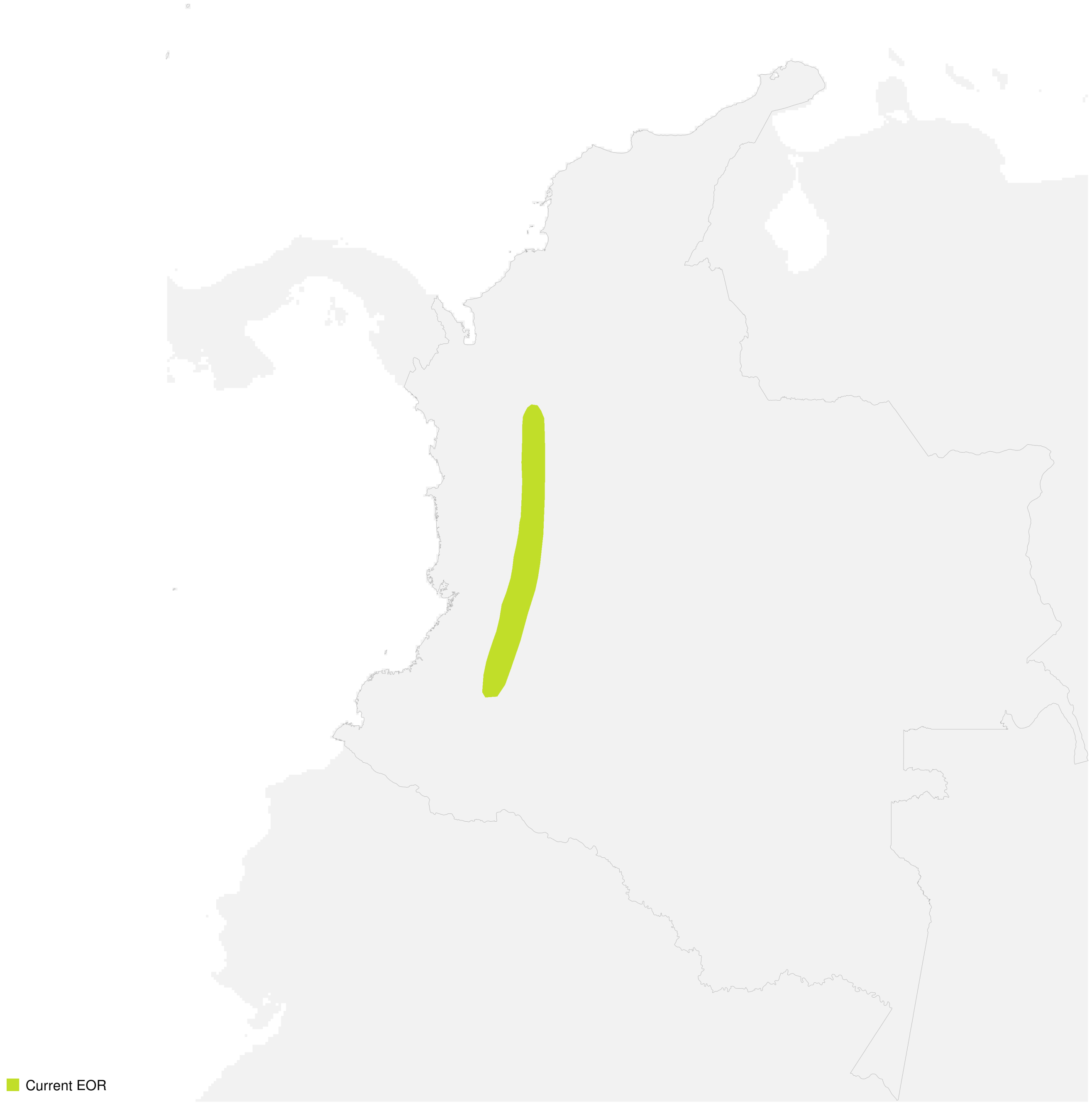

Bothrocophias hyoprora

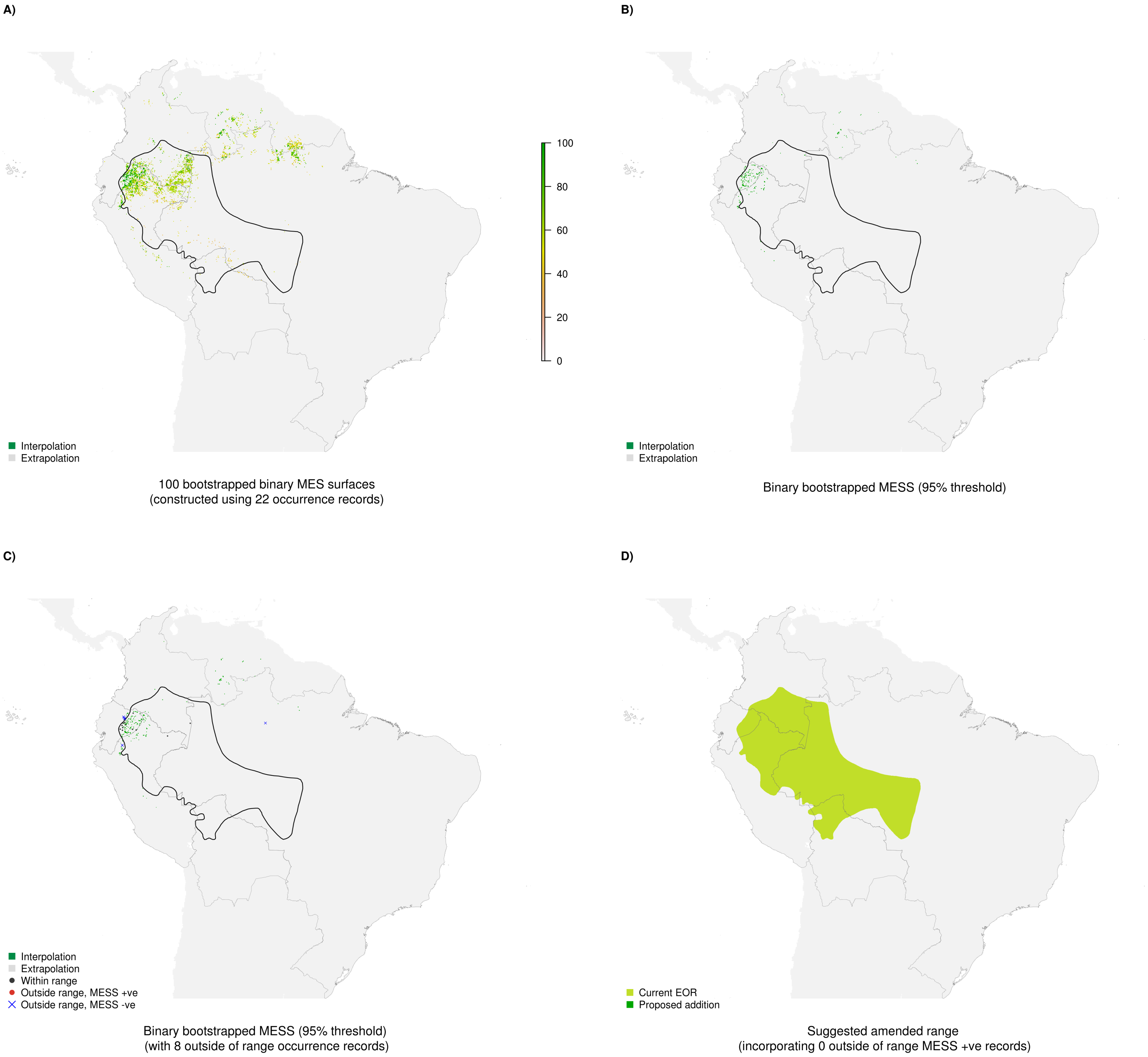

Bothrocophias microphthalmus

A)

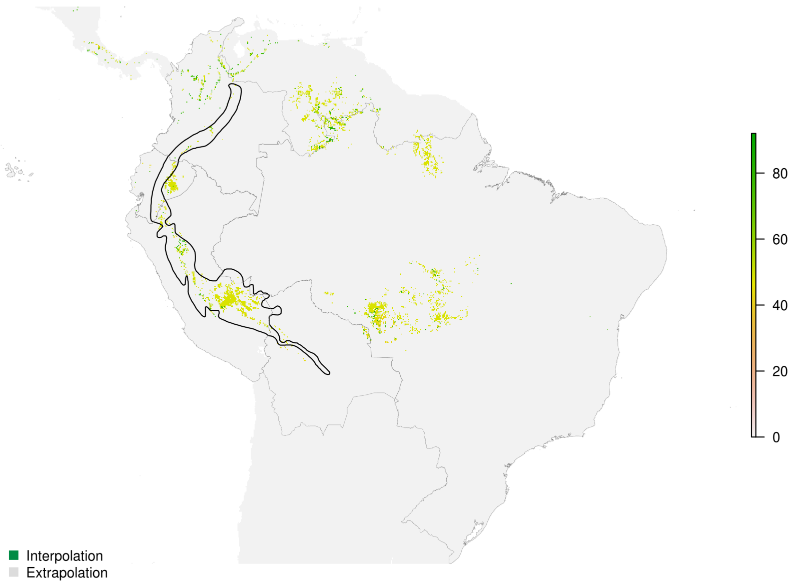

100 bootstrapped binary MES surfaces
(constructed using 7 occurrence records)

B)

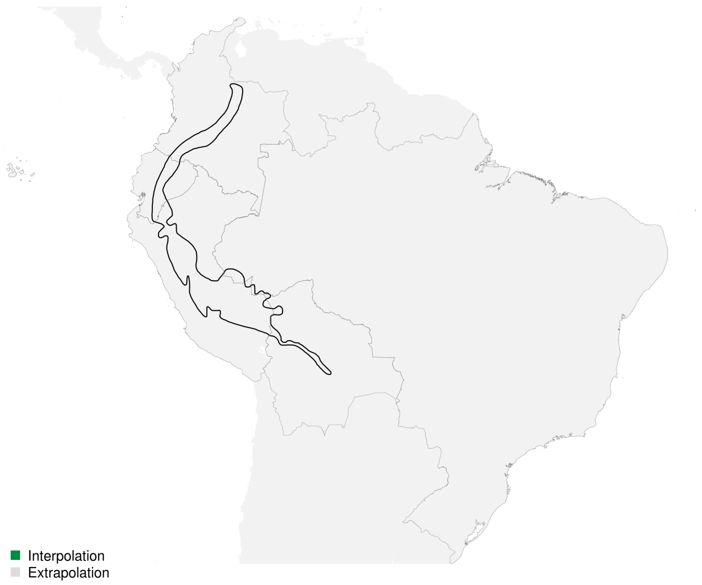

Binary bootstrapped MESS (95% threshold)
[0 cells met this threshold]

B)

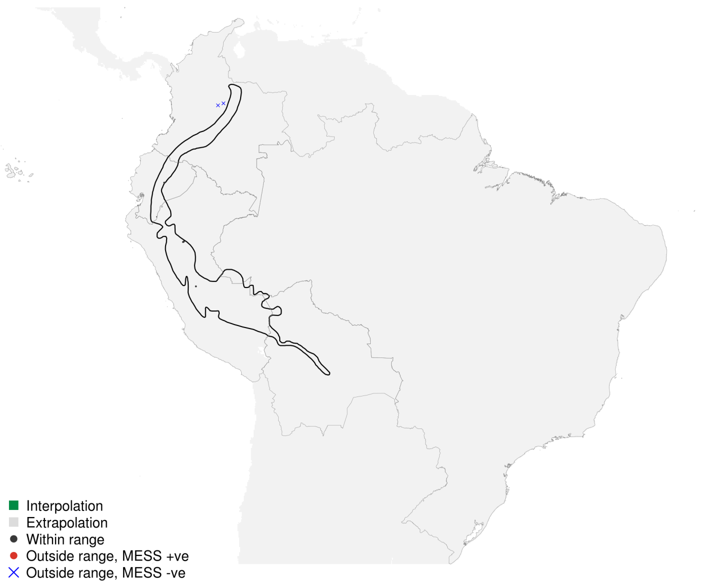

Binary bootstrapped MESS (95% threshold)
(with 2 outside of range occurrence records)

D)

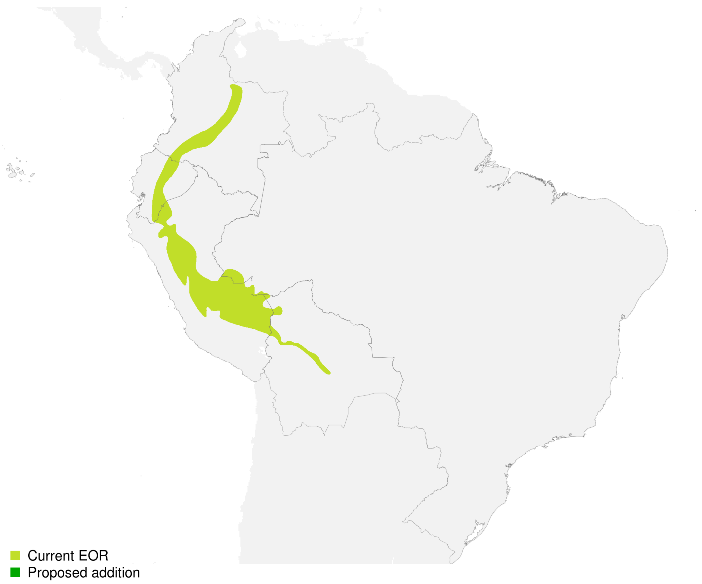

Suggested amended range
(incorporating 0 outside of range MESS +ve records)

Bothrocophias myersi

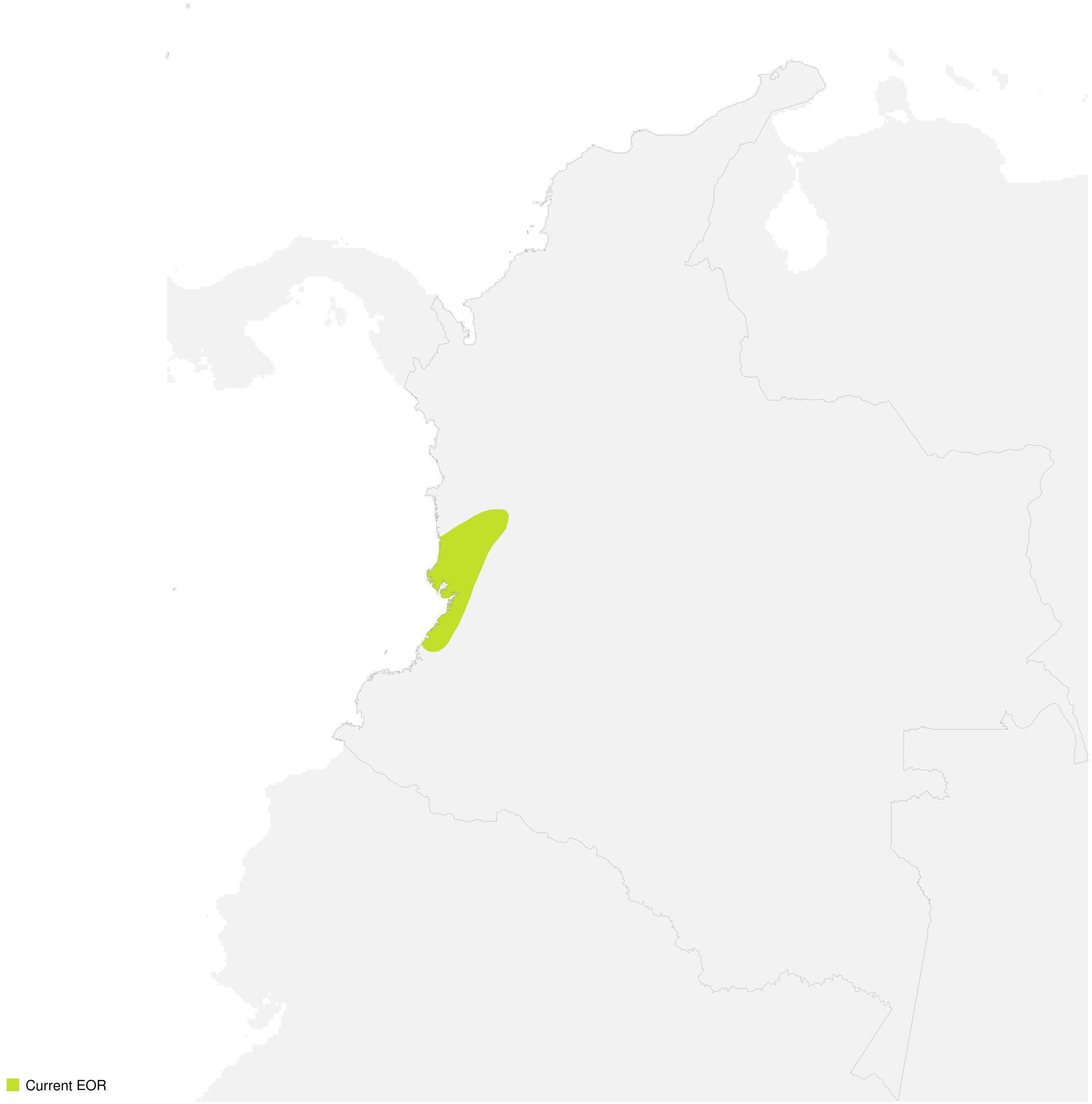

Bothrops alternatus

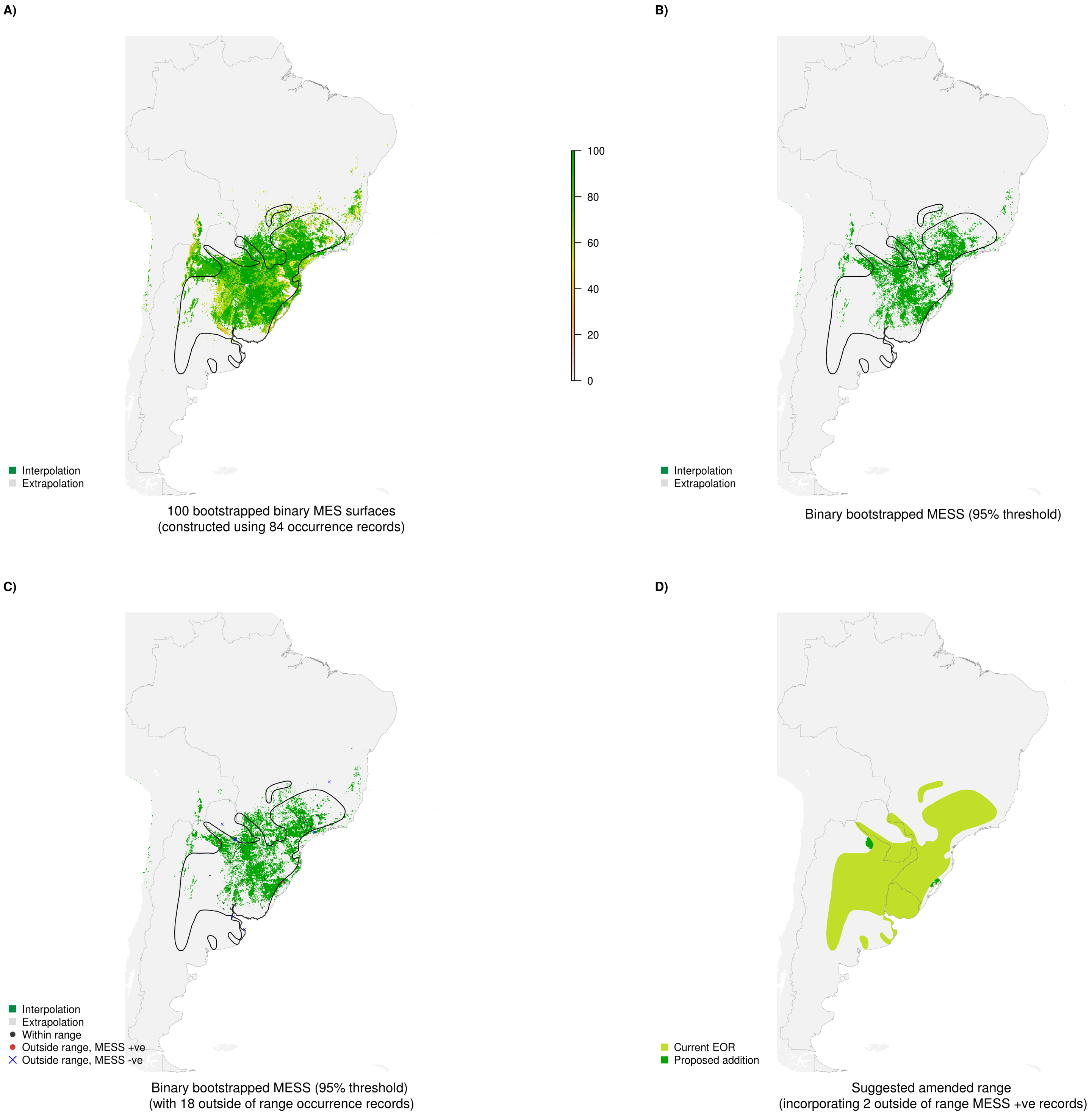

Bothrops ammodytoides

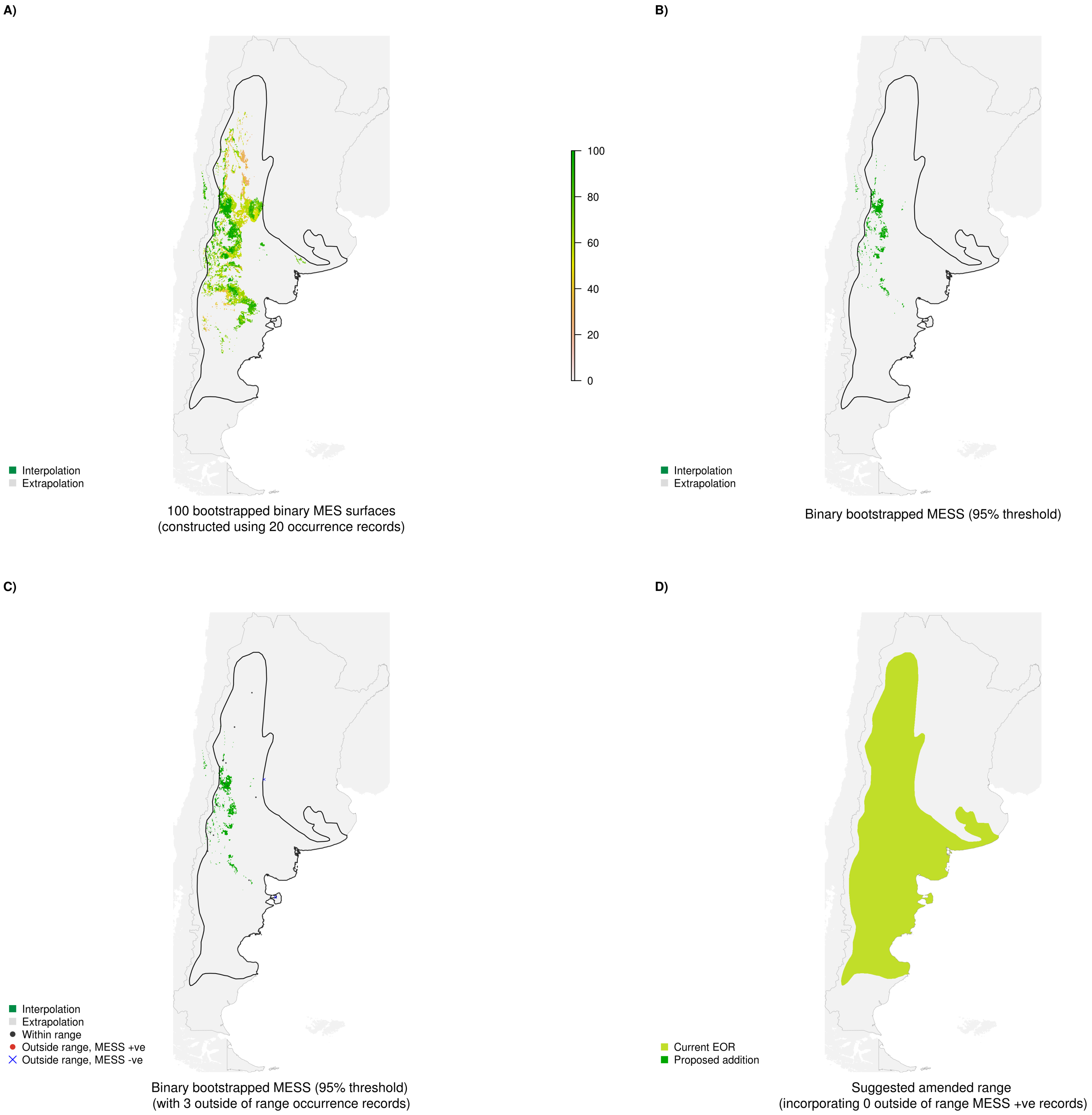

Bothrops andianus

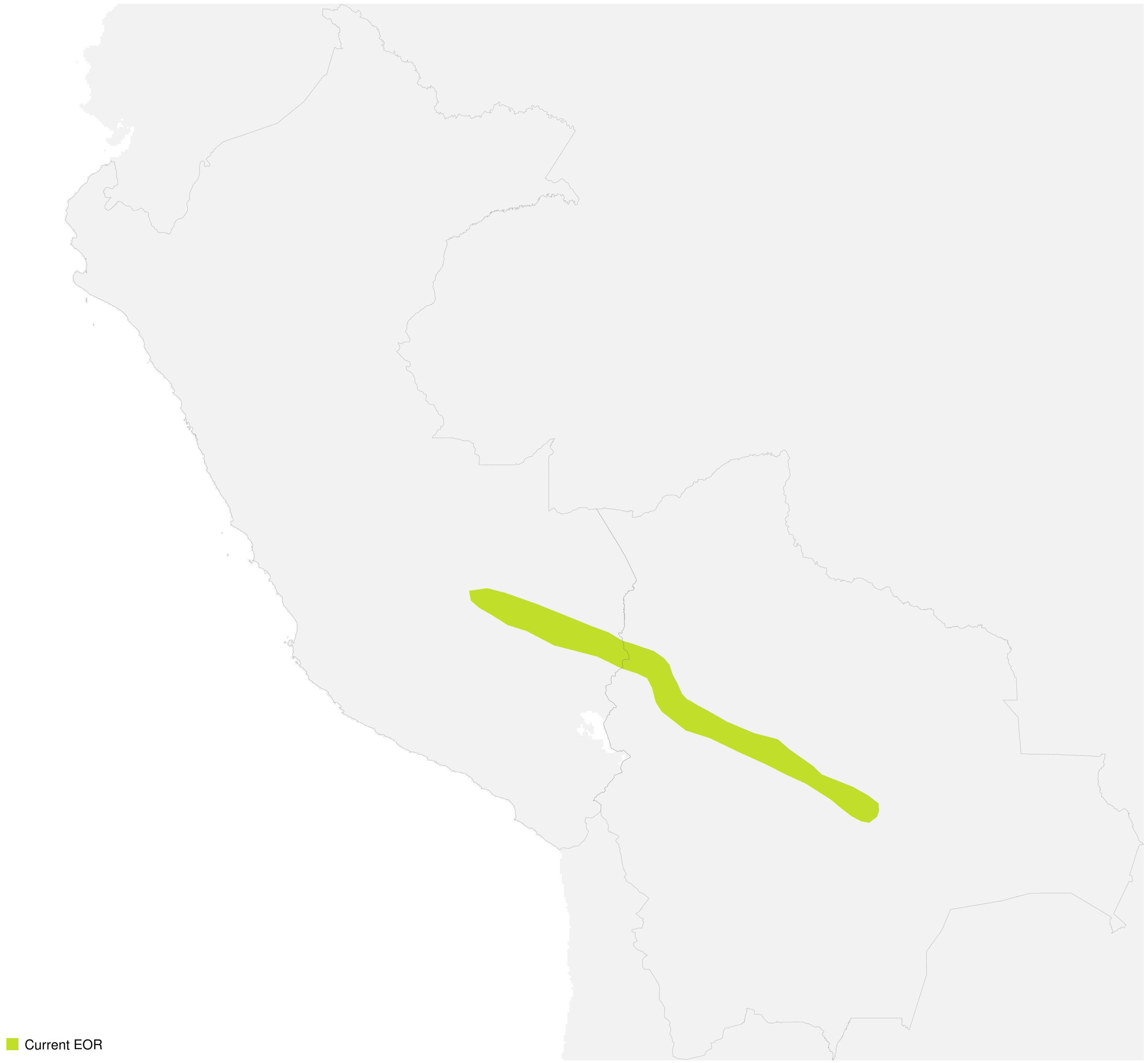

Bothrops asper

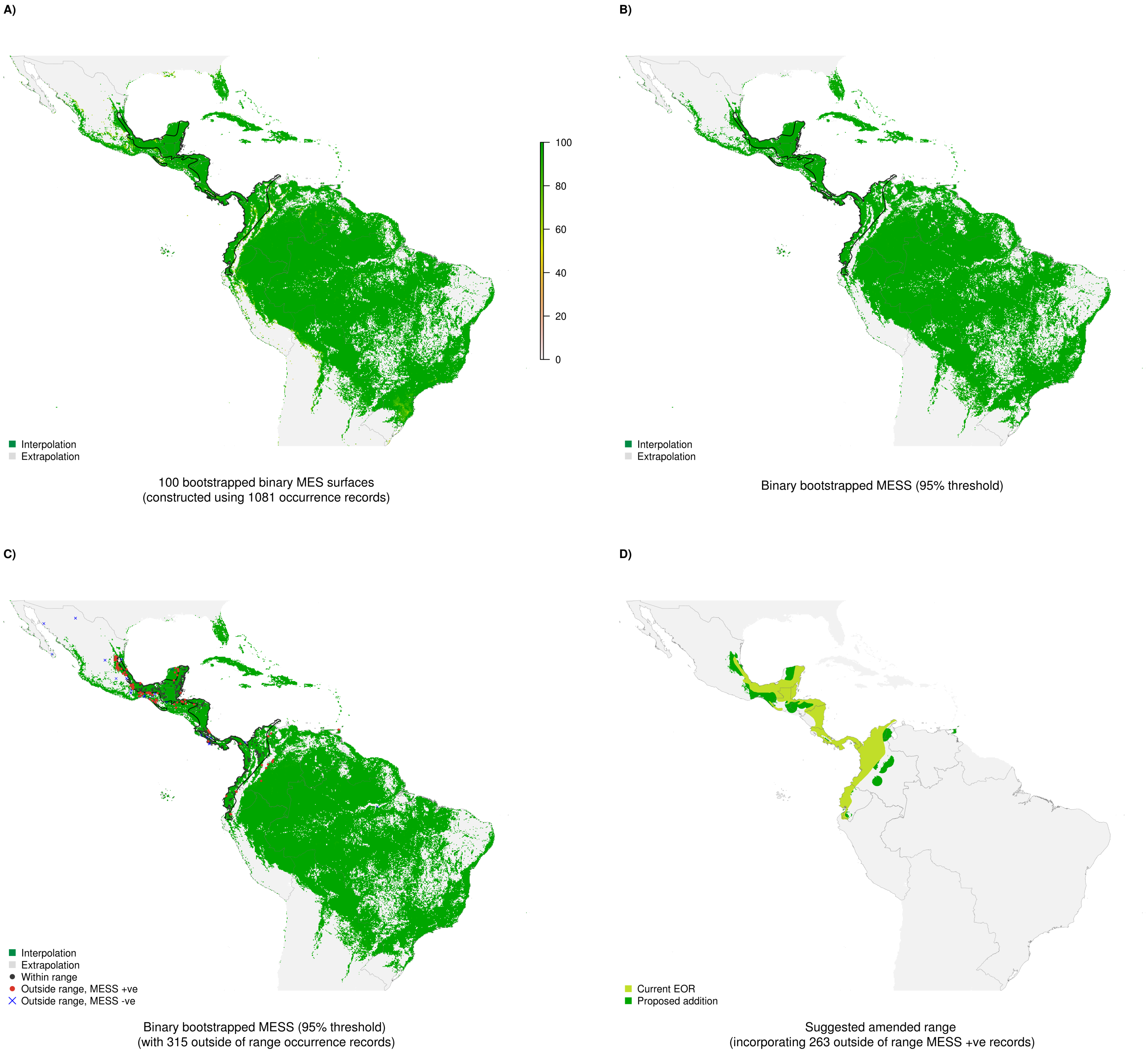

Bothrops atrox

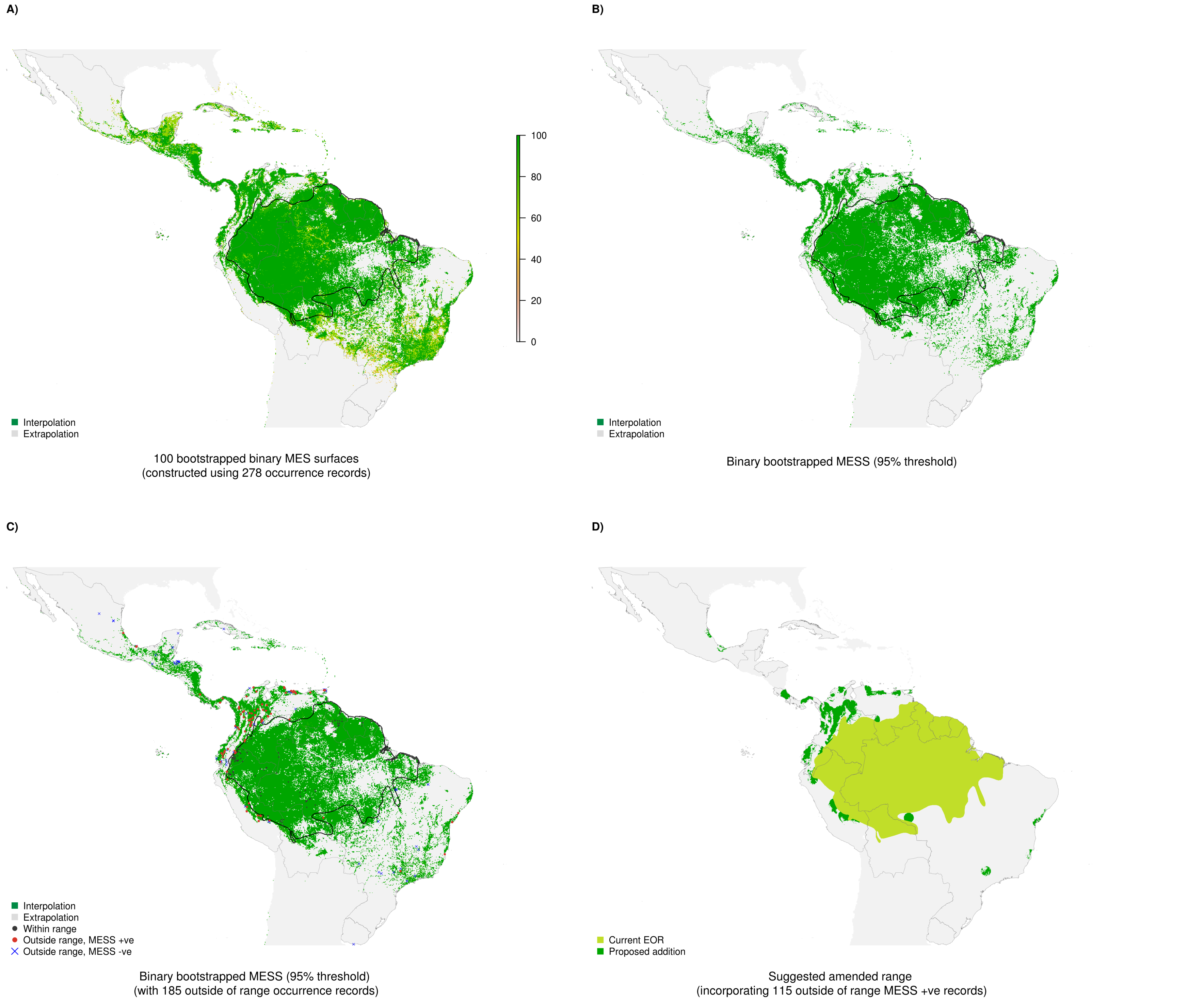

Bothrops barnetti

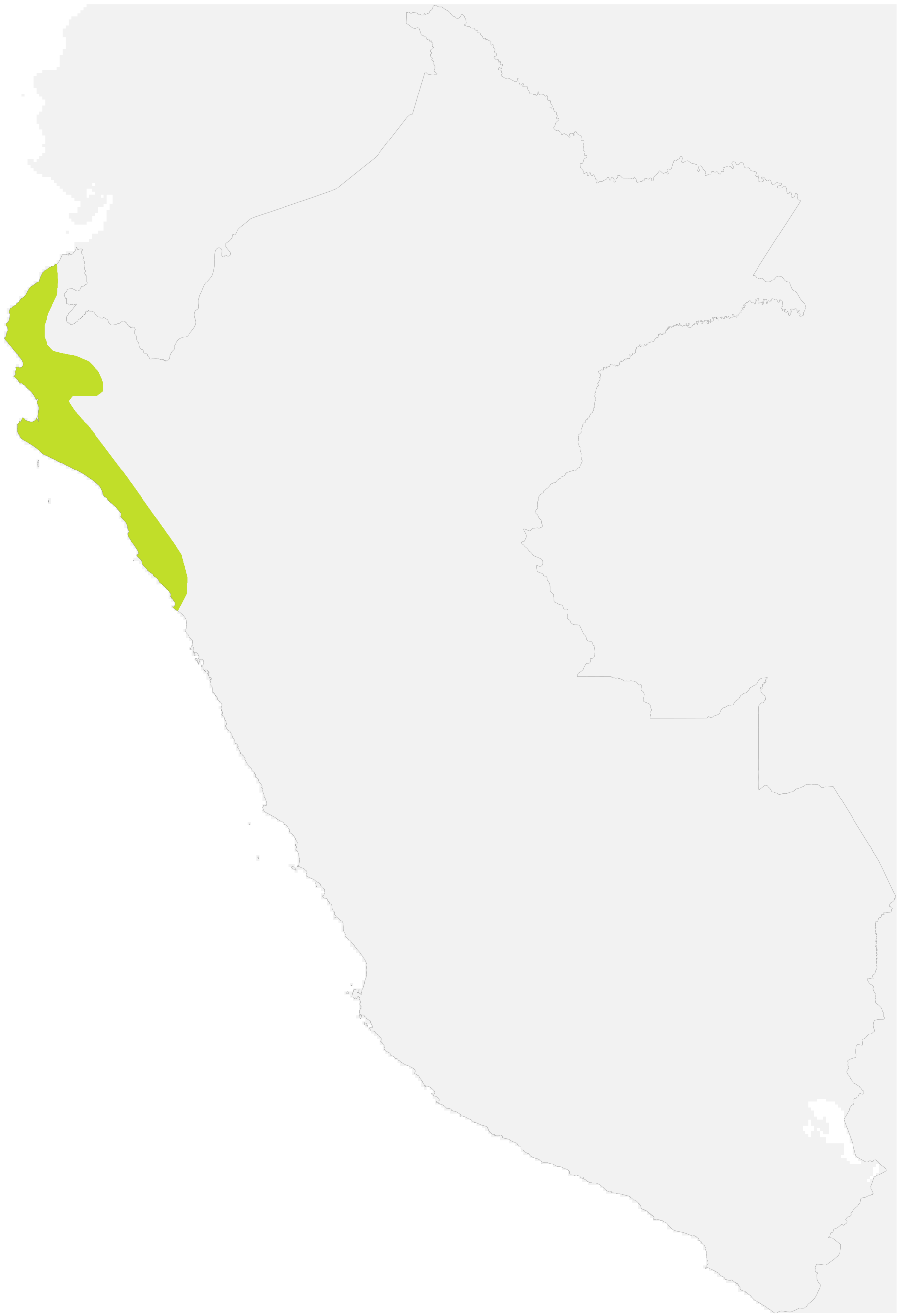

Current EOR

Bothrops bilineatus

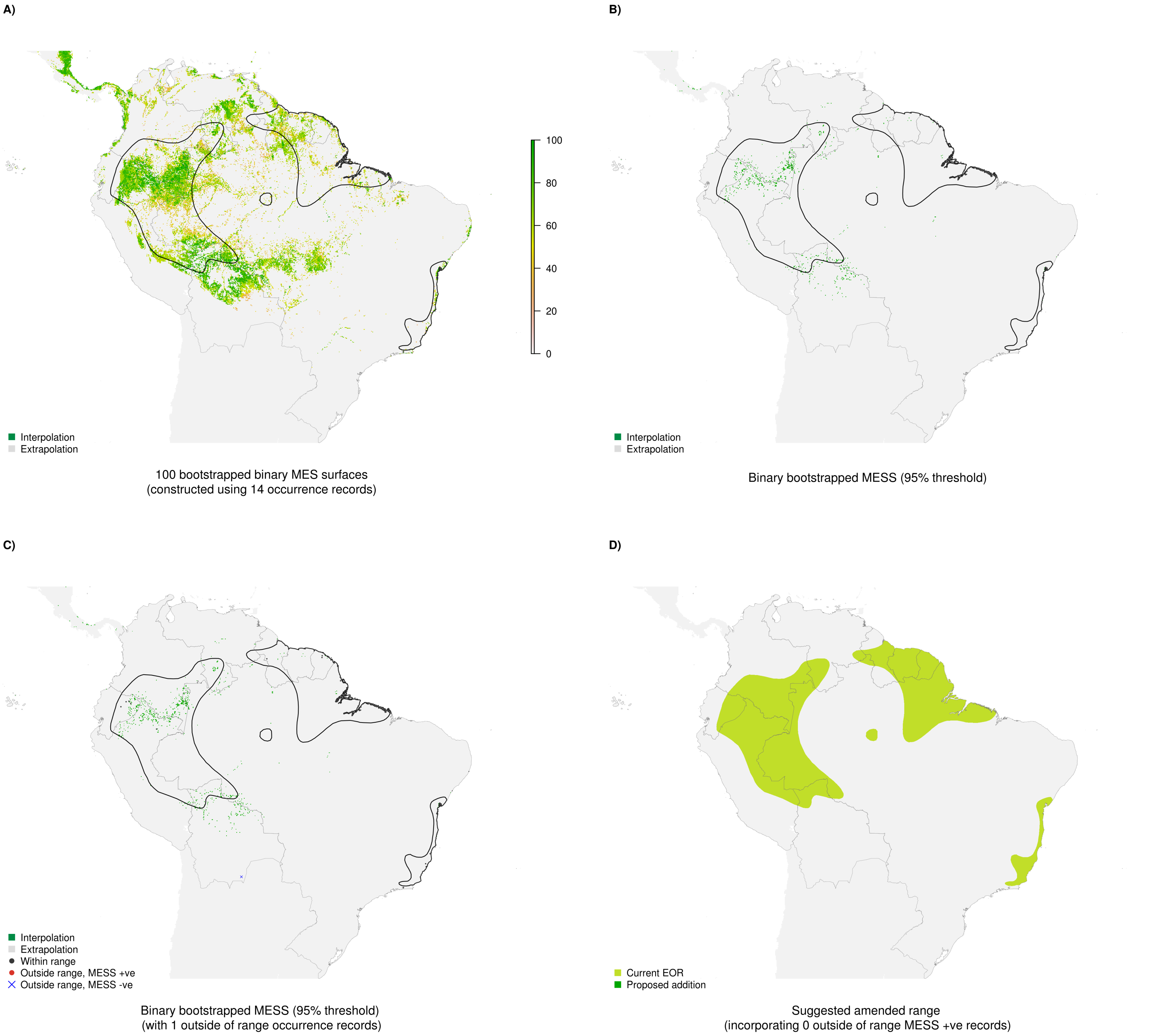

Bothrops brazili

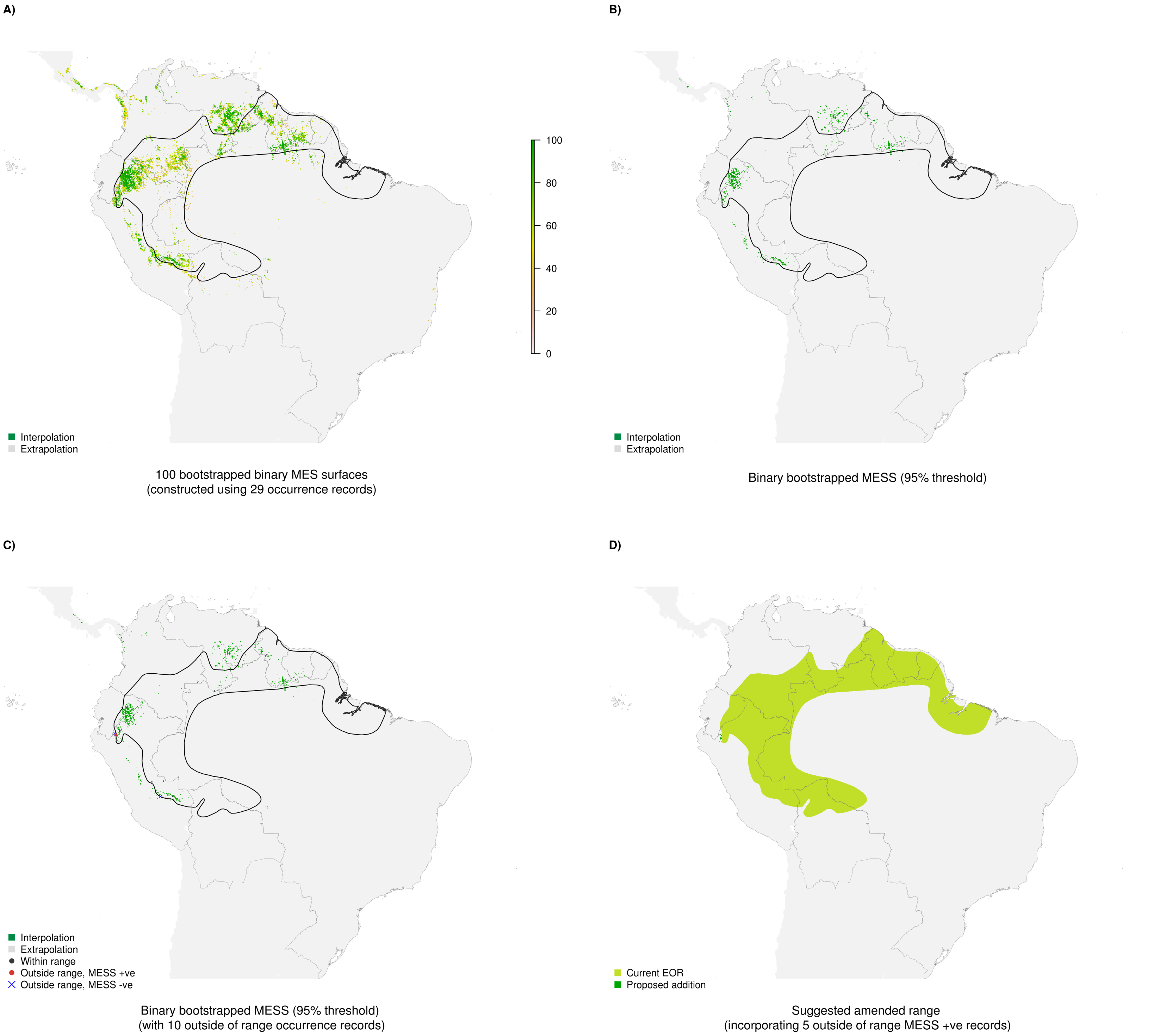

Bothrops caribbaeus

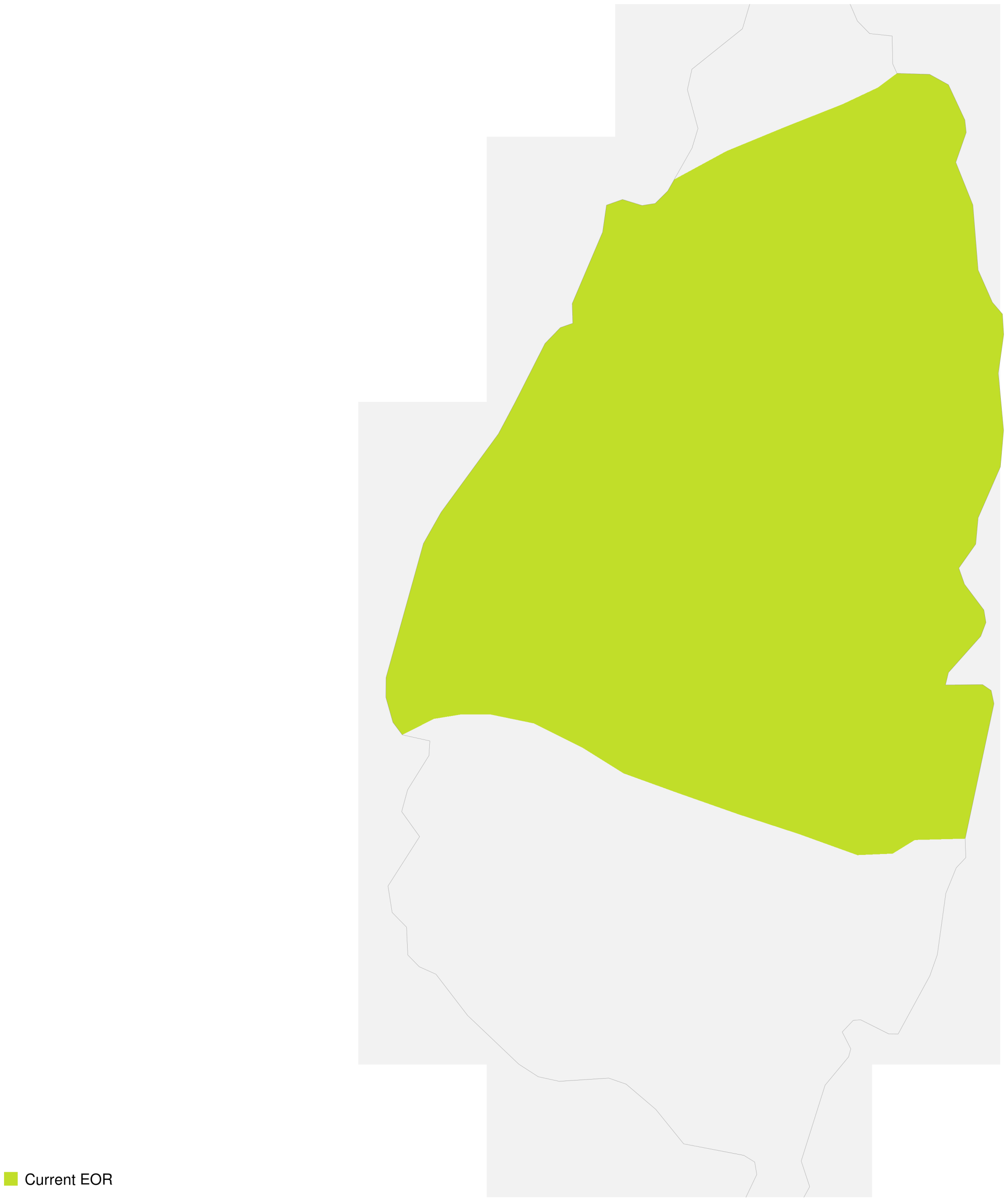

Bothrops cf atrox

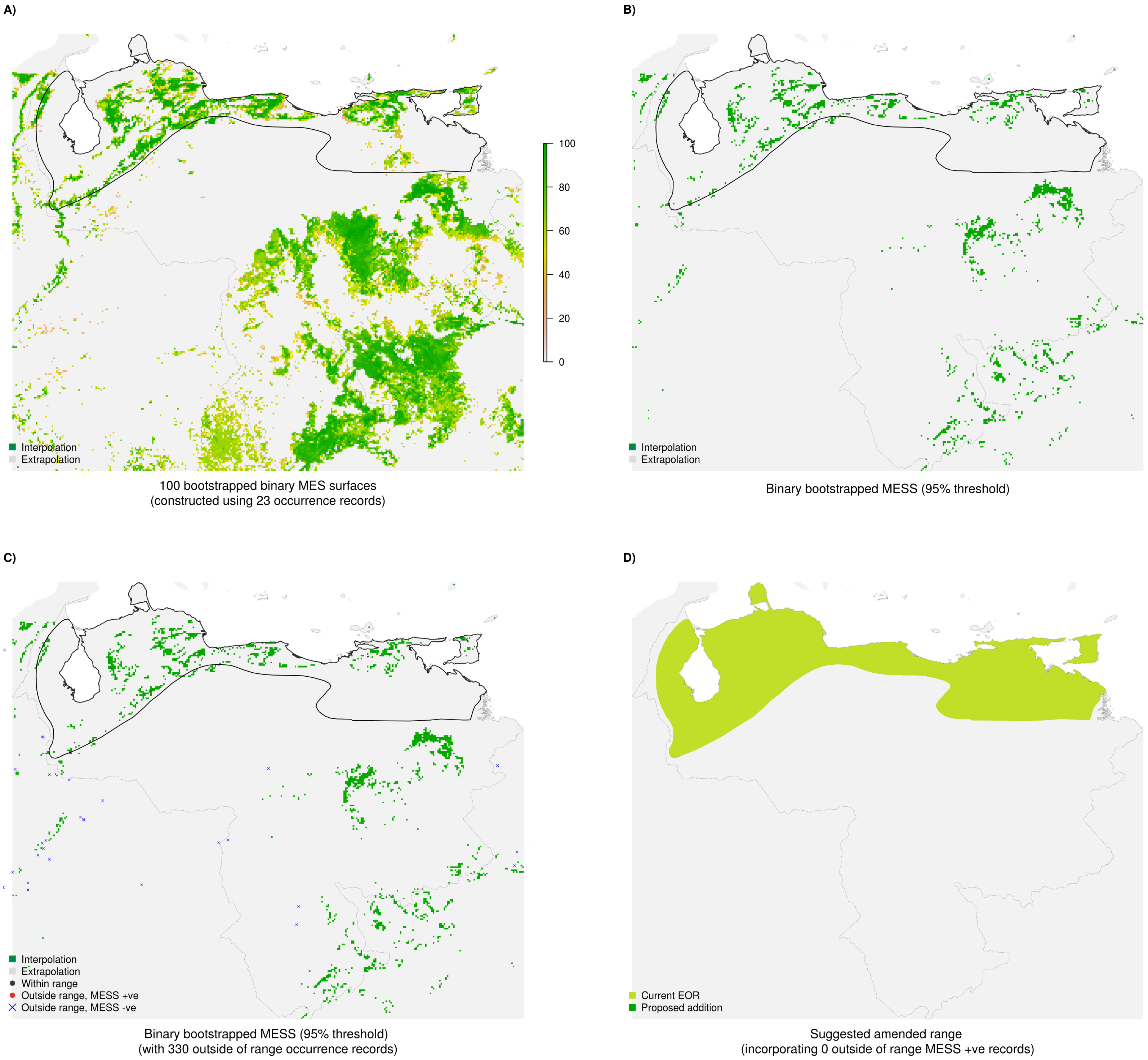

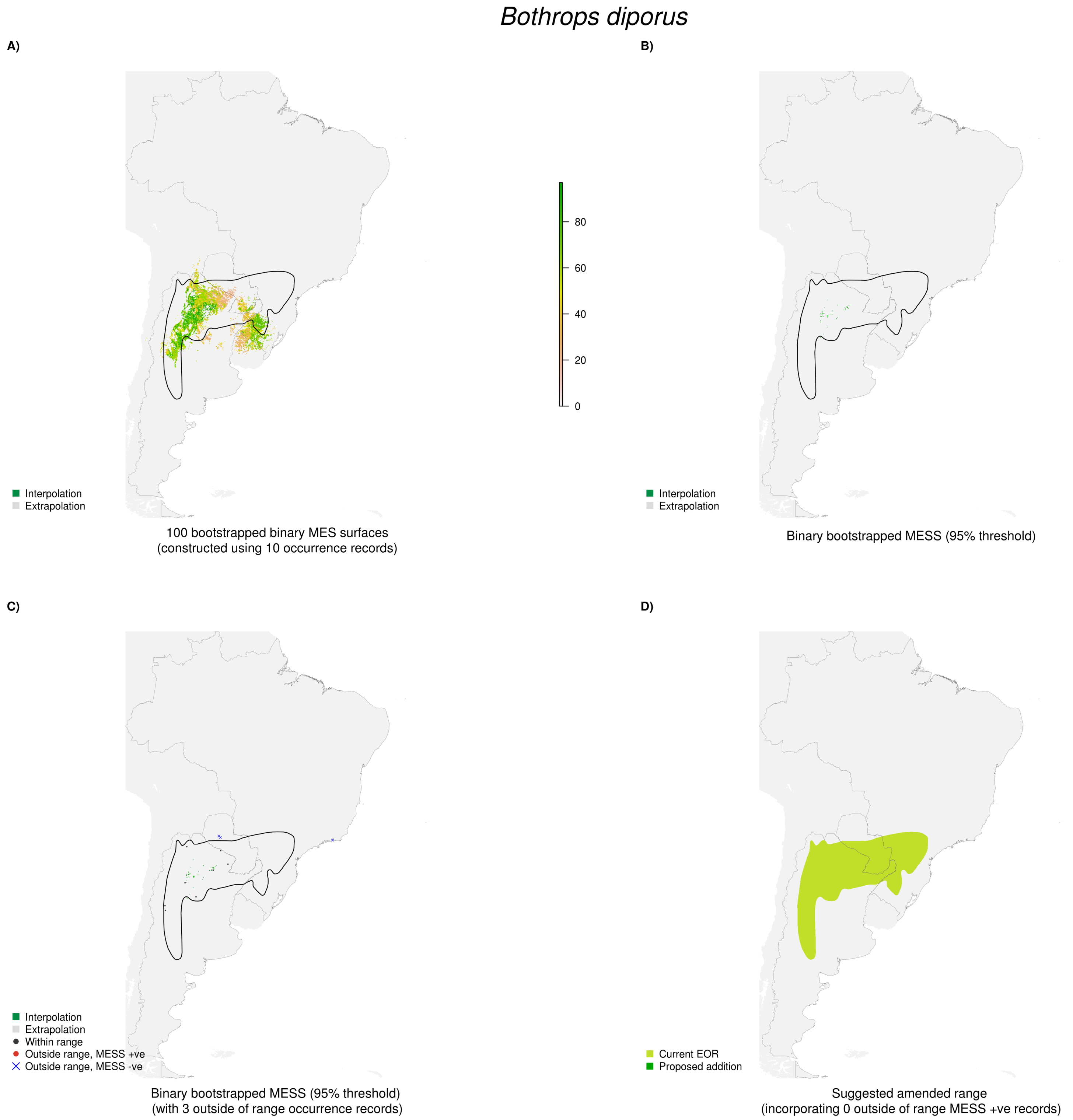

Bothrops diporus
A)
80
60
40
20
0
Interpolation
Extrapolation
100 bootstrapped binary MES surfaces
(constructed using 10 occurrence records)
B)
Interpolation
Extrapolation
Binary bootstrapped MESS (95% threshold)
C)
Interpolation
Extrapolation
Within range
Outside range, MESS +ve
Outside range, MESS -ve
Binary bootstrapped MESS (95% threshold)
(with 3 outside of range occurrence records)
D)
Current EOR
Proposed addition
Suggested amended range
(incorporating 0 outside of range MESS +ve records)

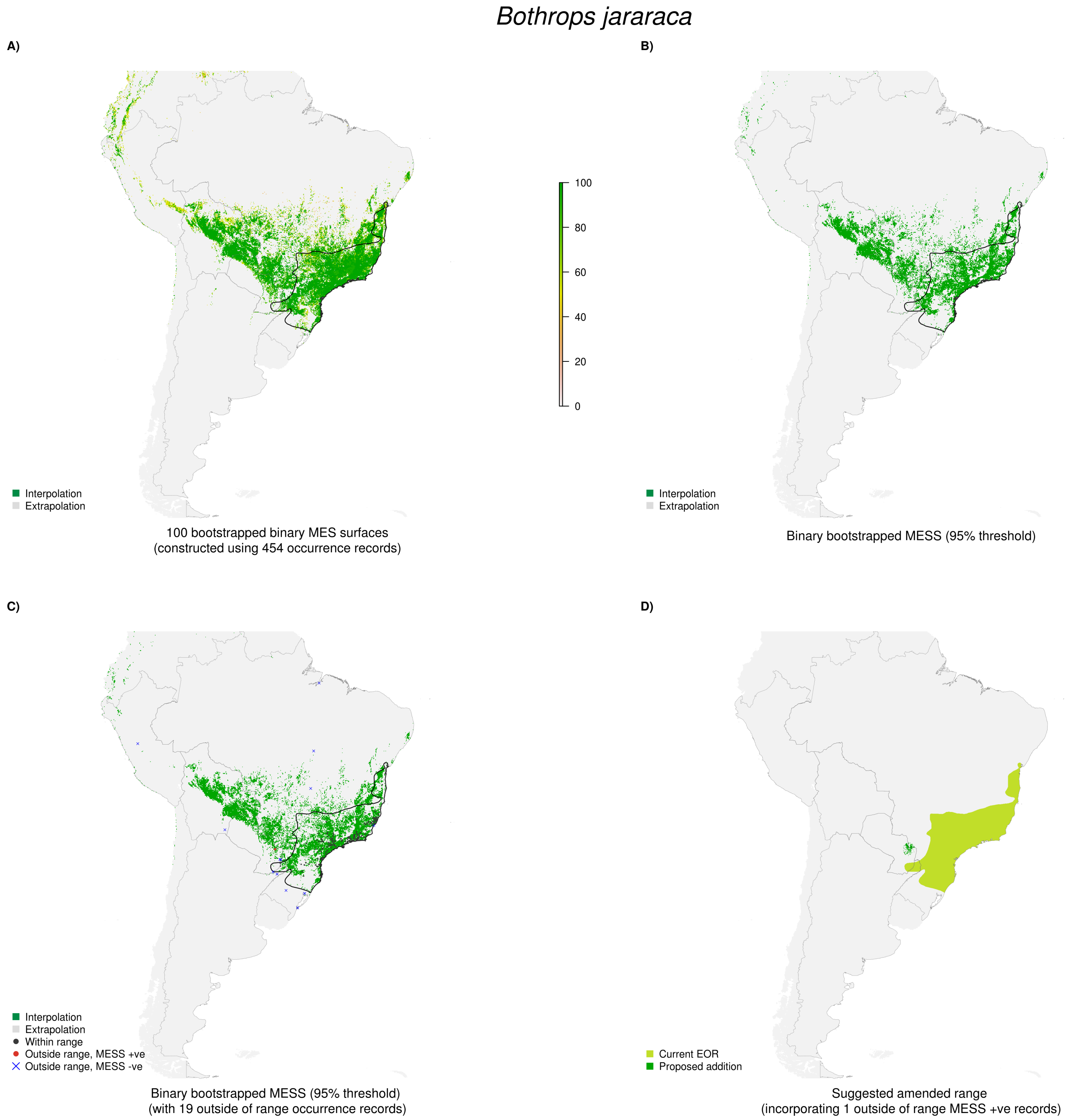

Bothrops jararaca
A)
B)
100
80
60
40
20
0
Interpolation
Extrapolation
100 bootstrapped binary MES surfaces
(constructed using 454 occurrence records)
Interpolation
Extrapolation
Binary bootstrapped MESS (95% threshold)
C)
D)
Interpolation
Extrapolation
Within range
Outside range, MESS +ve
Outside range, MESS -ve
Binary bootstrapped MESS (95% threshold)
(with 19 outside of range occurrence records)
Current EOR
Proposed addition
Suggested amended range
(incorporating 1 outside of range MESS +ve records)

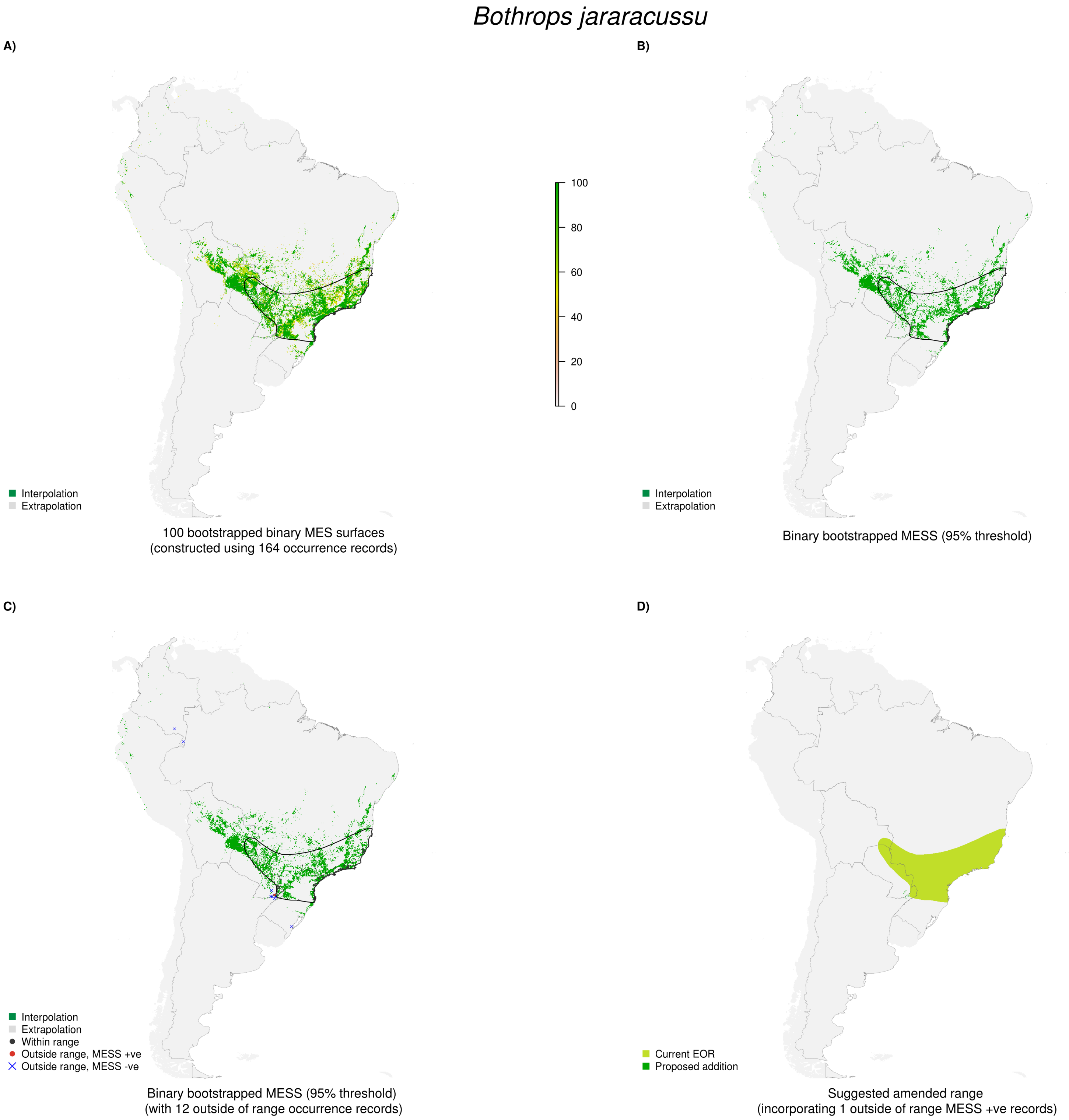
Bothrops jararacussu
A)
B)
100
80
60
40
20
0
Interpolation
Extrapolation
100 bootstrapped binary MES surfaces
(constructed using 164 occurrence records)
Interpolation
Extrapolation
Binary bootstrapped MESS (95% threshold)
C)
D)
Interpolation
Extrapolation
Within range
Outside range, MESS +ve
Outside range, MESS -ve
Binary bootstrapped MESS (95% threshold)
(with 12 outside of range occurrence records)
Current EOR
Proposed addition
Suggested amended range
(incorporating 1 outside of range MESS +ve records)

Bothrops jonathani

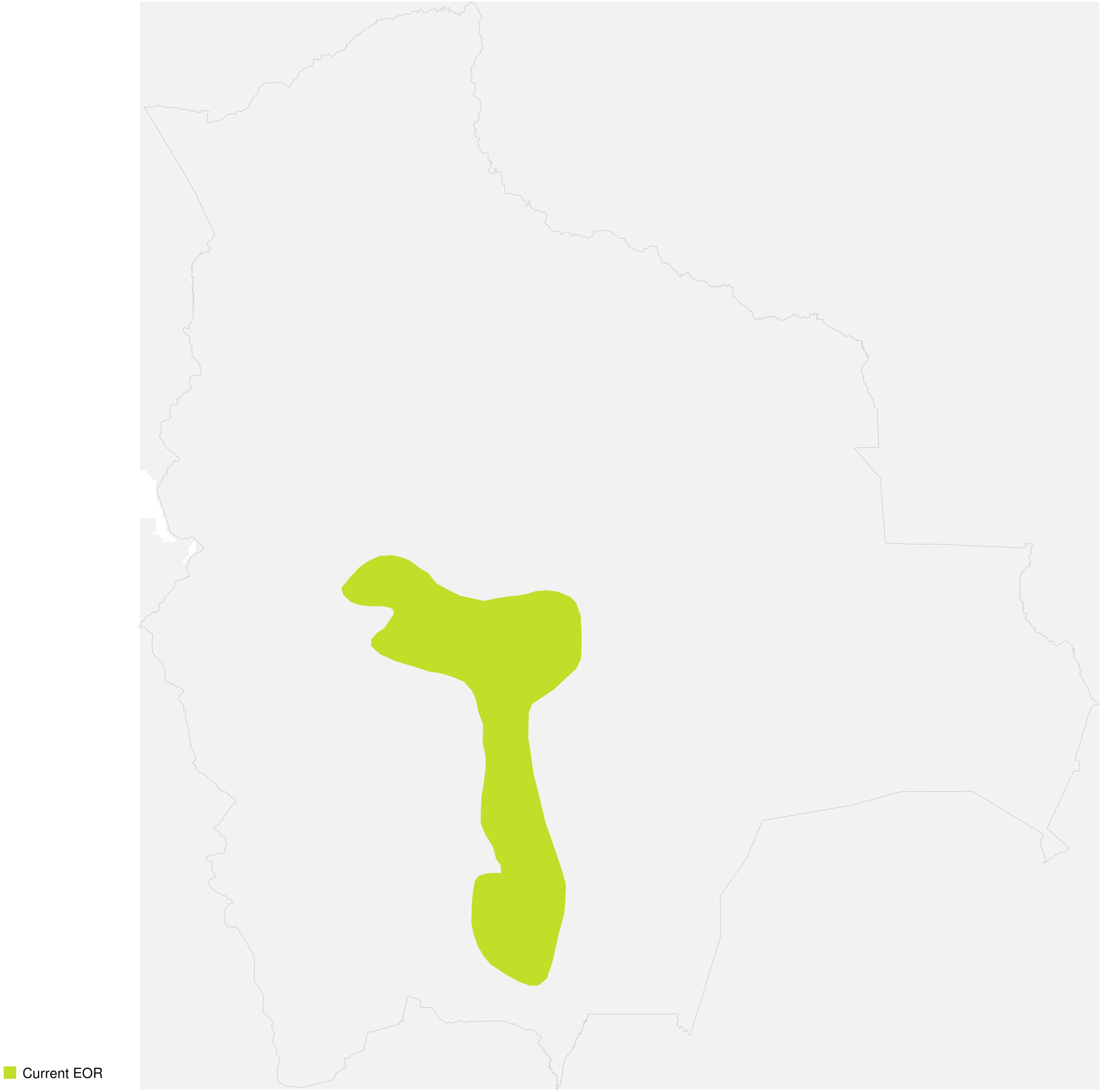

Bothrops lanceolatus

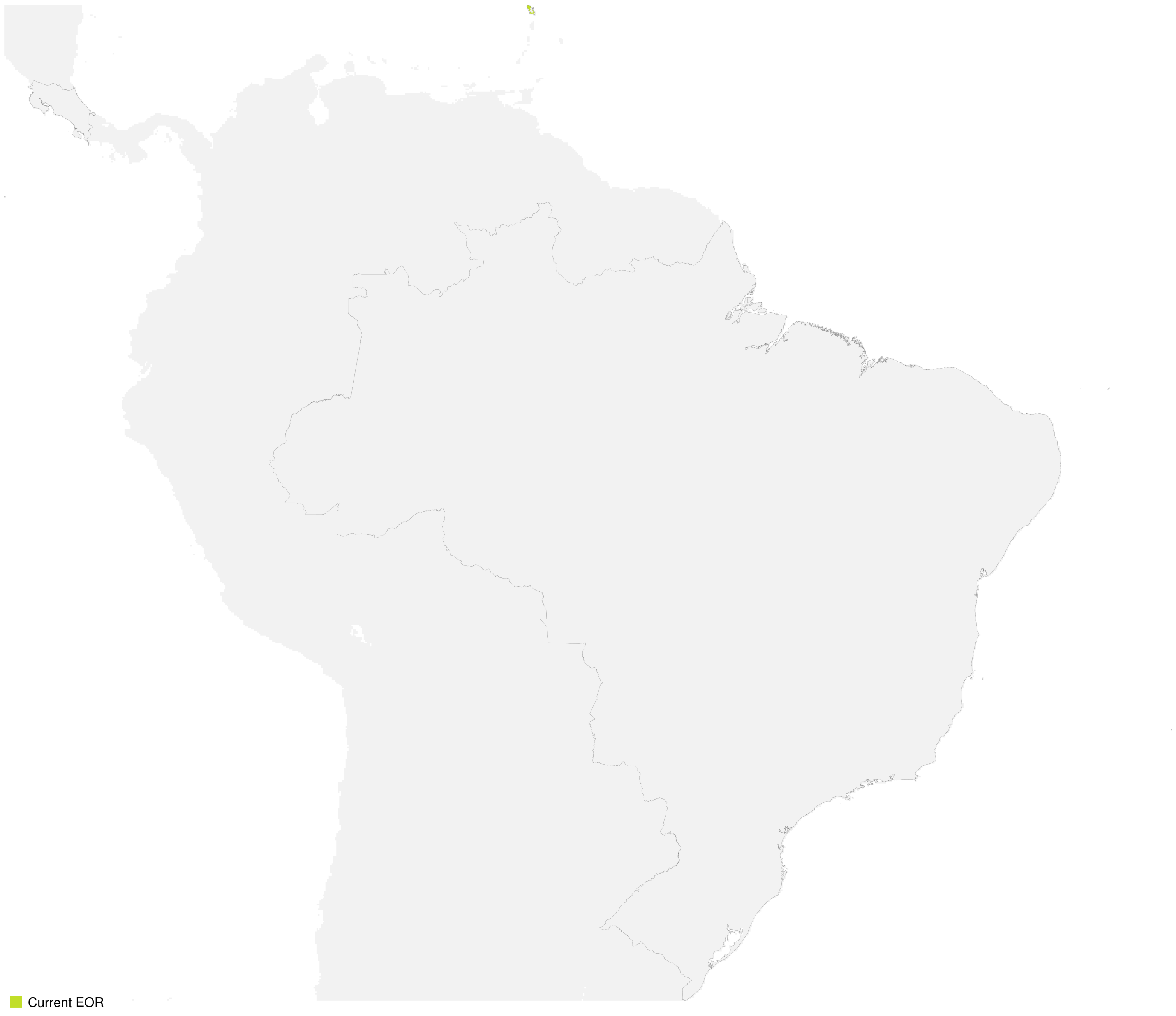

Bothrops leucurus

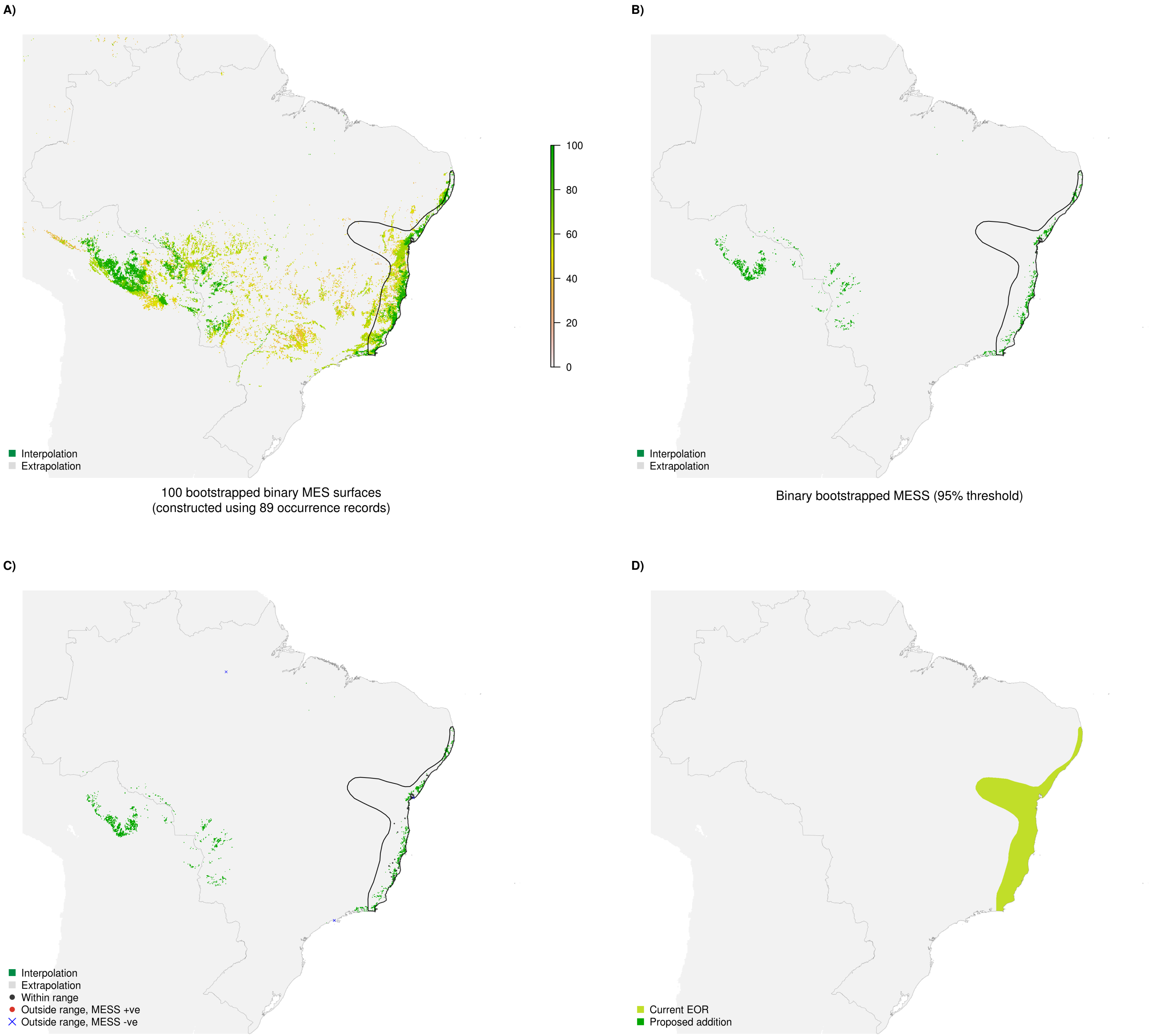

Binary bootstrapped MESS (95% threshold)
(with 4 outside of range occurrence records)

Suggested amended range
(incorporating 0 outside of range MESS +ve records)

Bothrops lojanus

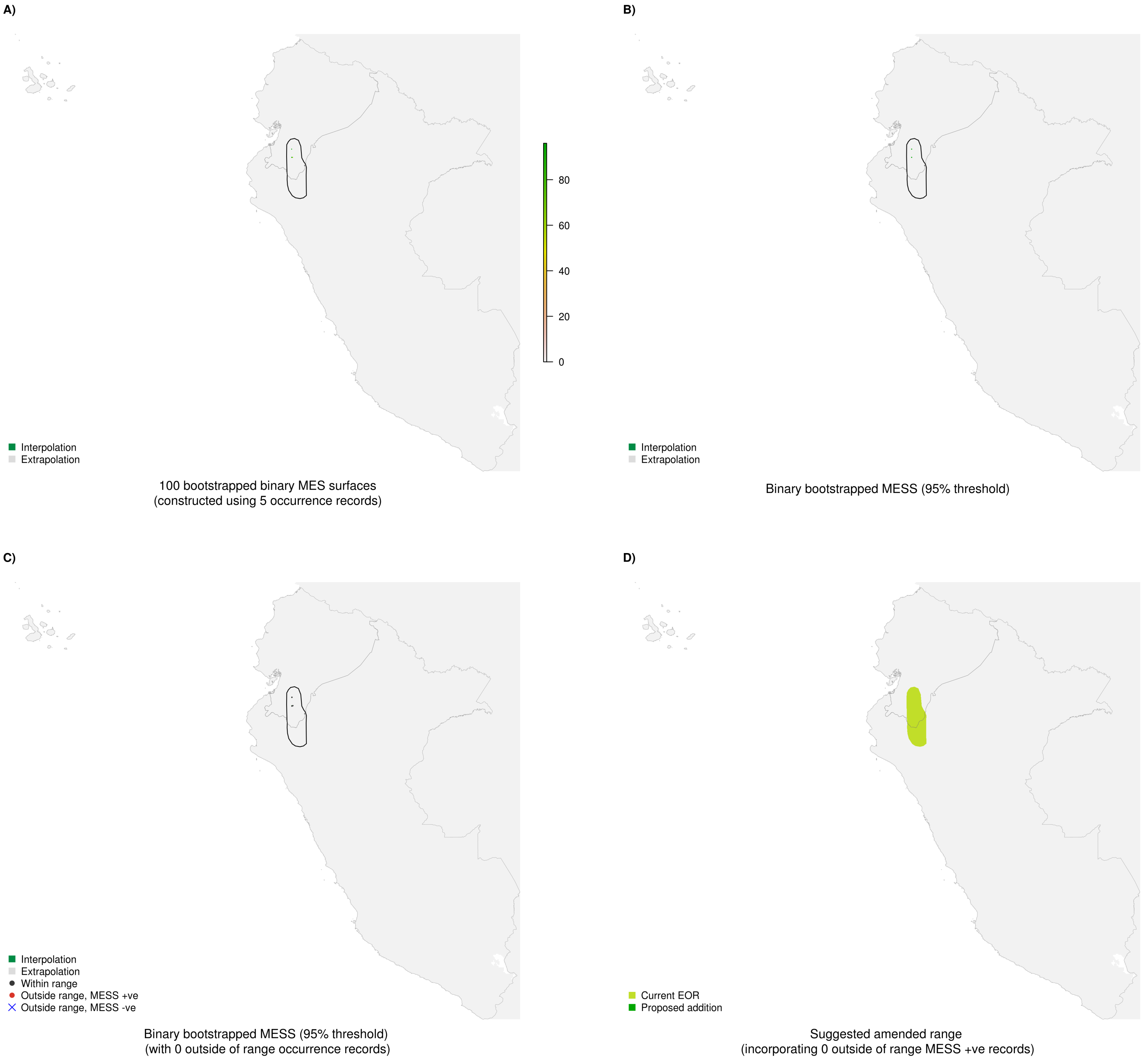

Bothrops marajoensis

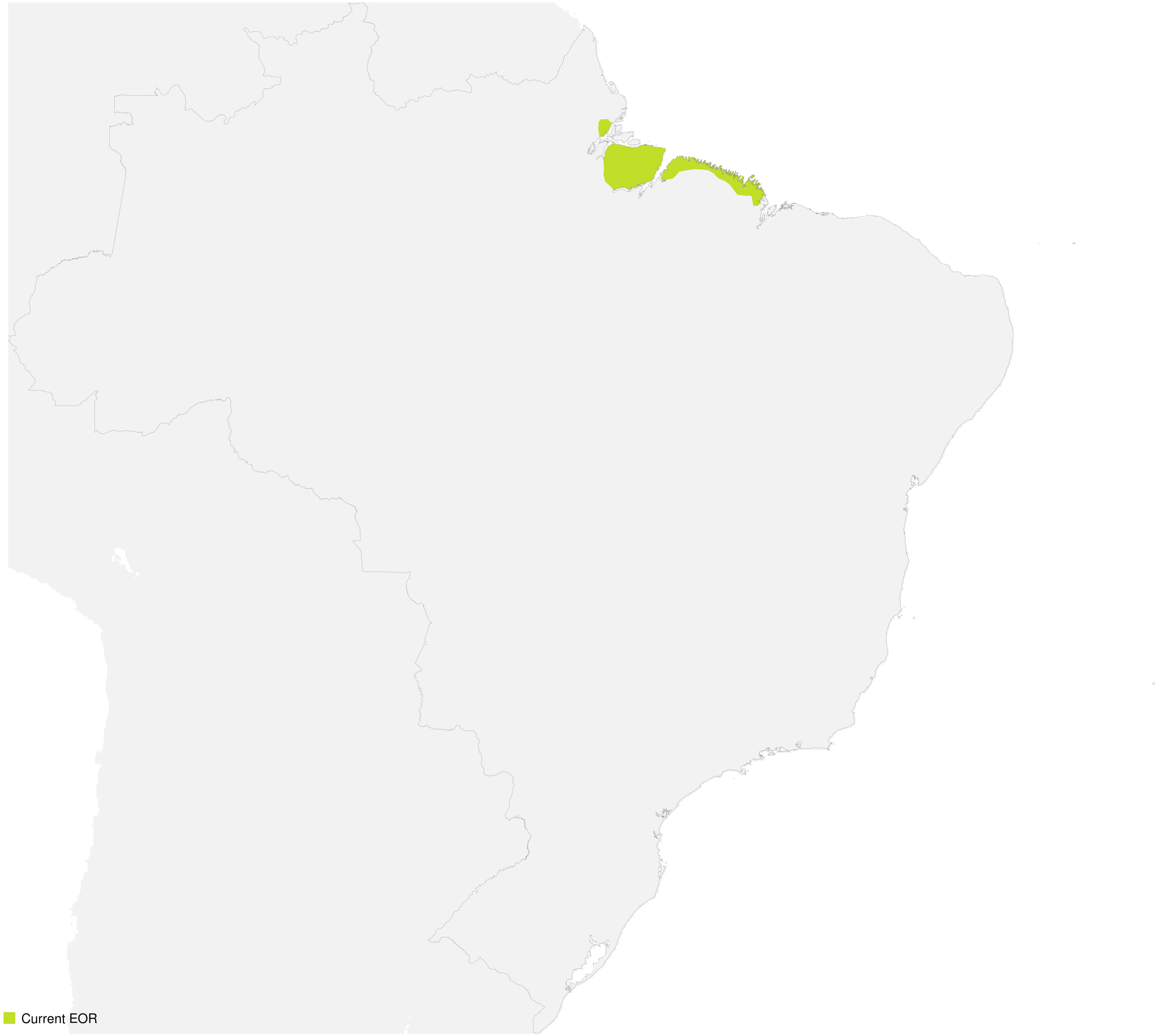

Bothrops mattogrossensis

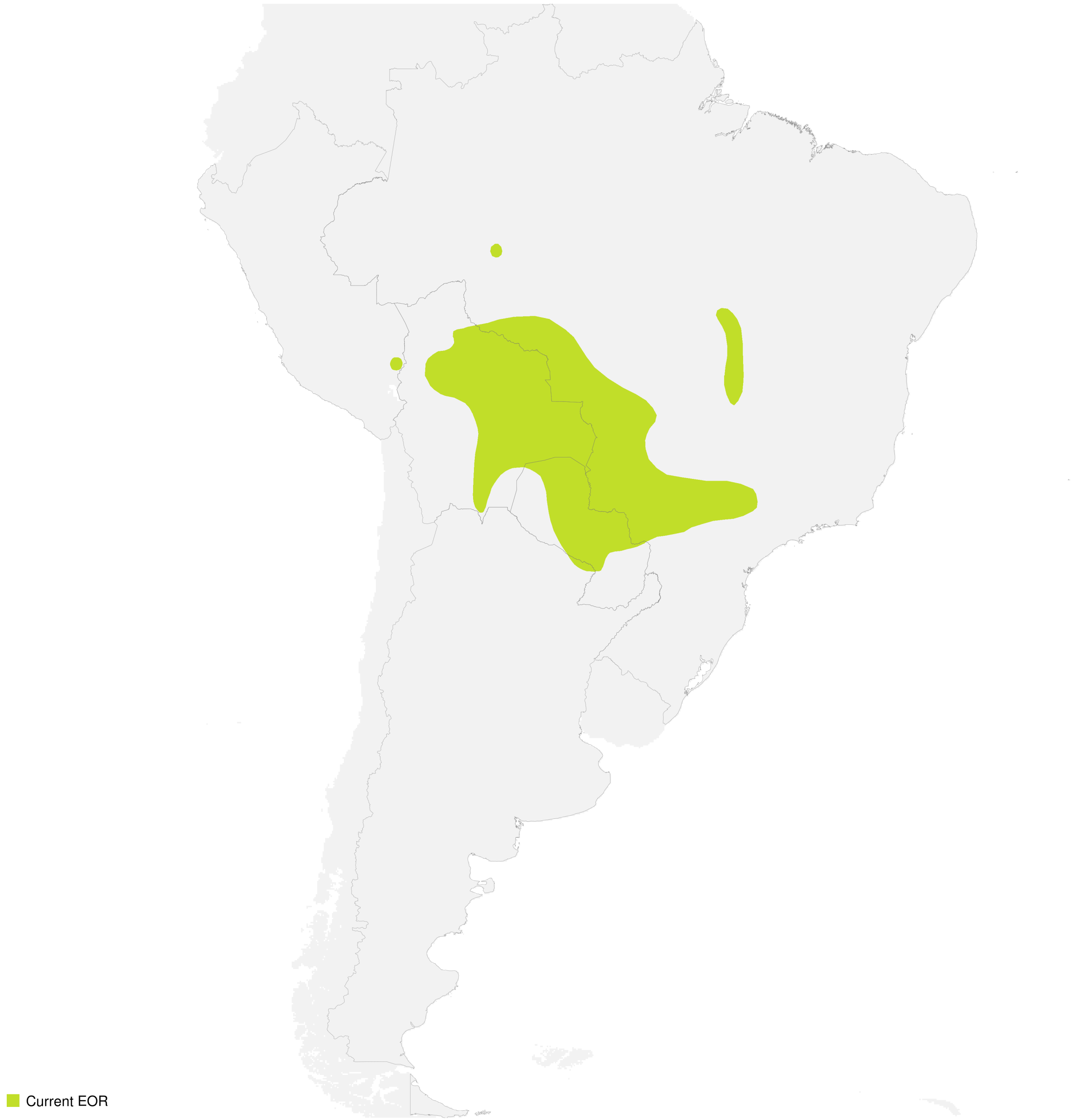

Bothrops moojeni

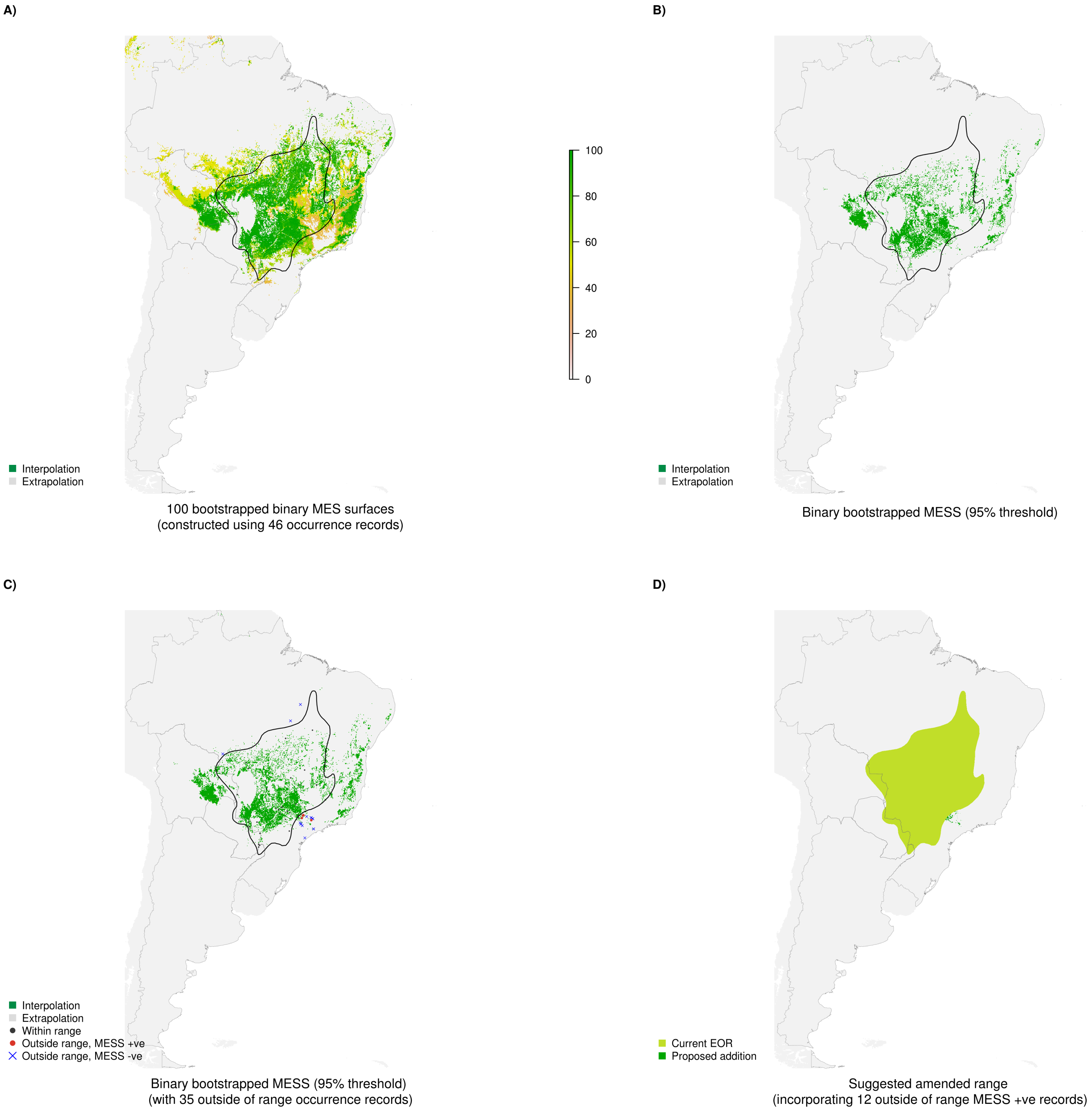

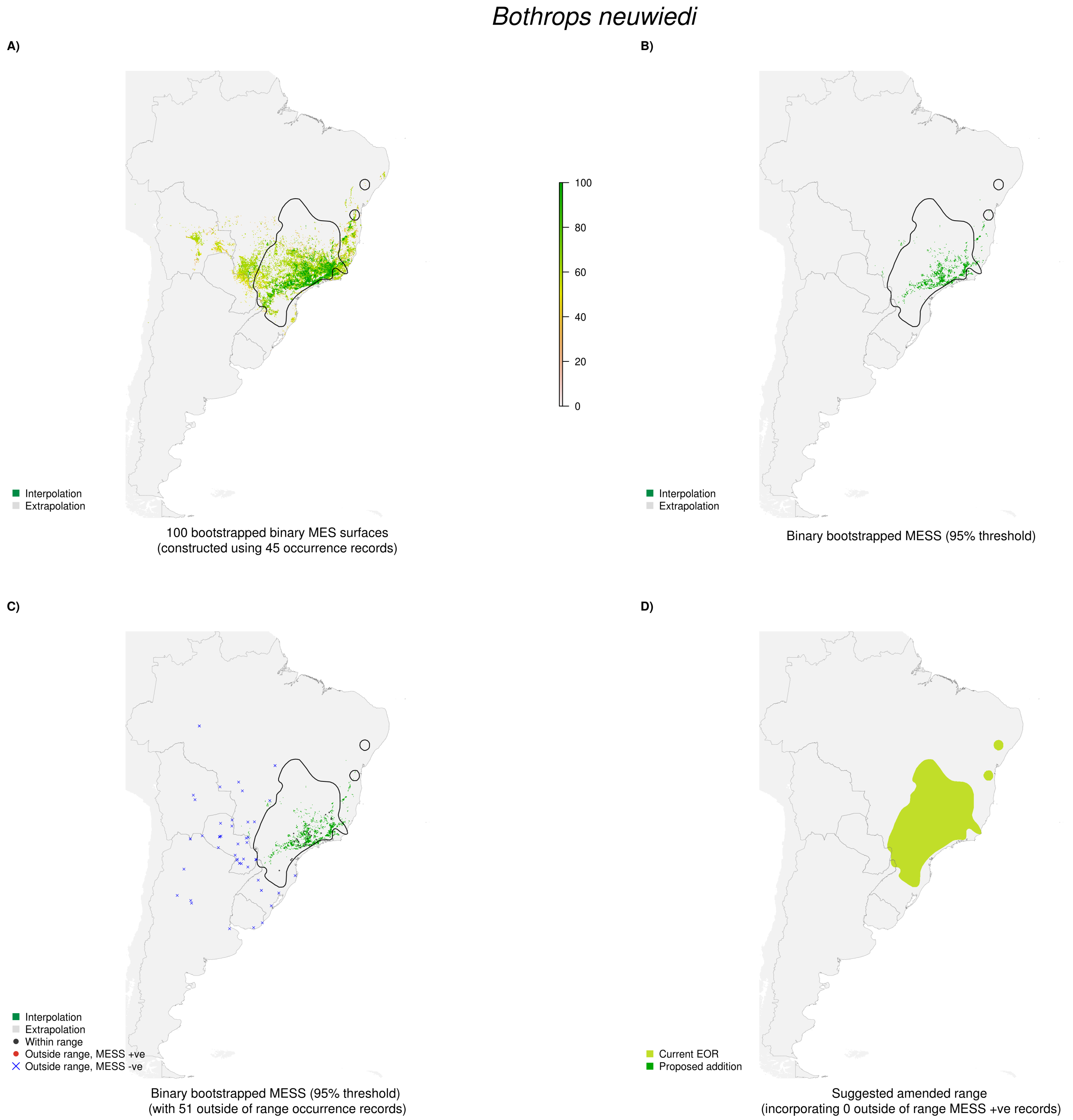

Bothrops neuwiedi
A)
B)
C)
D)
100
80
60
40
20
0
Interpolation
Extrapolation
Within range
Outside range, MESS +ve
Outside range, MESS -ve
Current EOR
Proposed addition
100 bootstrapped binary MES surfaces
(constructed using 45 occurrence records)
Binary bootstrapped MESS (95% threshold)
Binary bootstrapped MESS (95% threshold)
(with 51 outside of range occurrence records)
Suggested amended range
(incorporating 0 outside of range MESS +ve records)

Bothrops osbornei

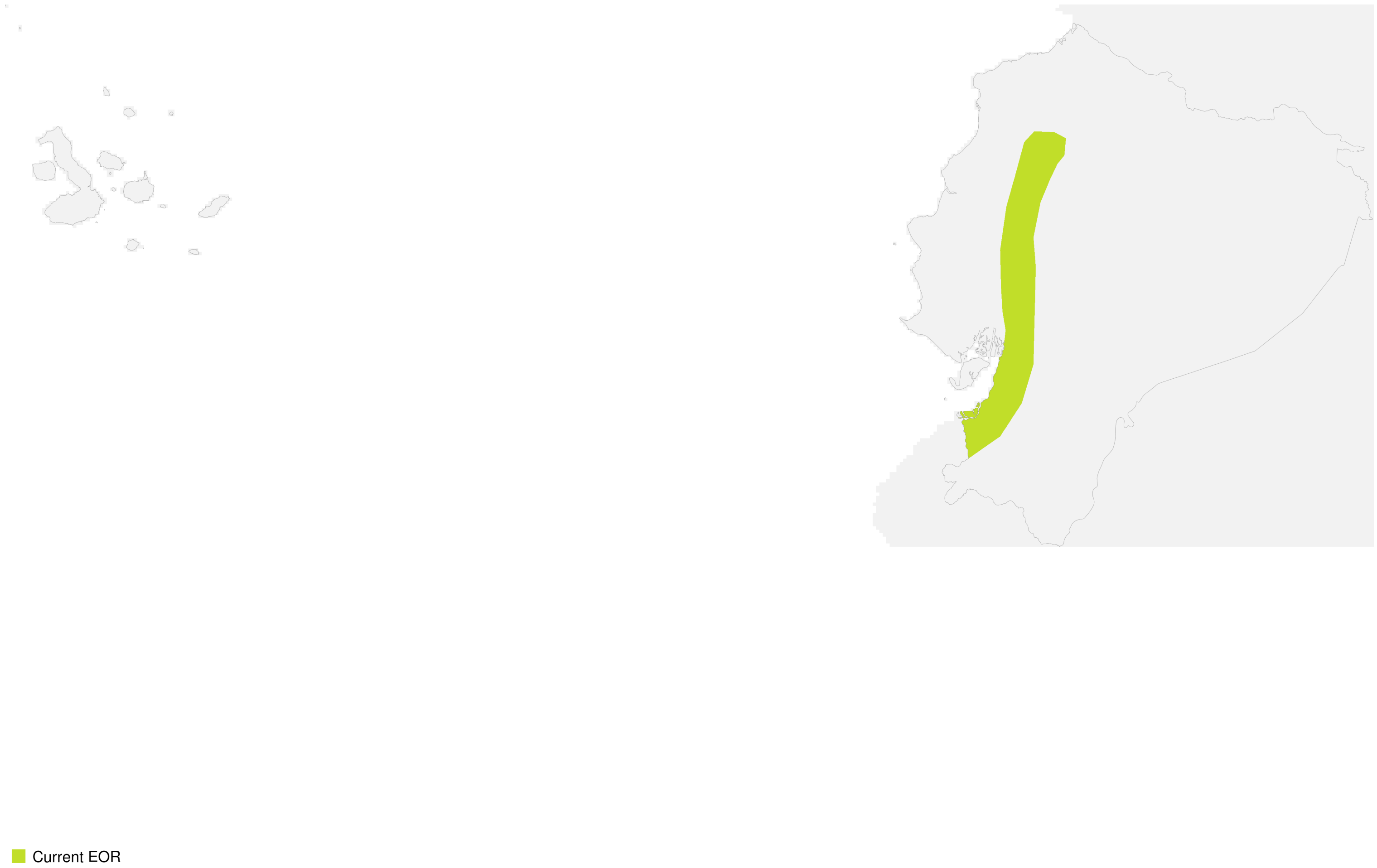

Bothrops pictus

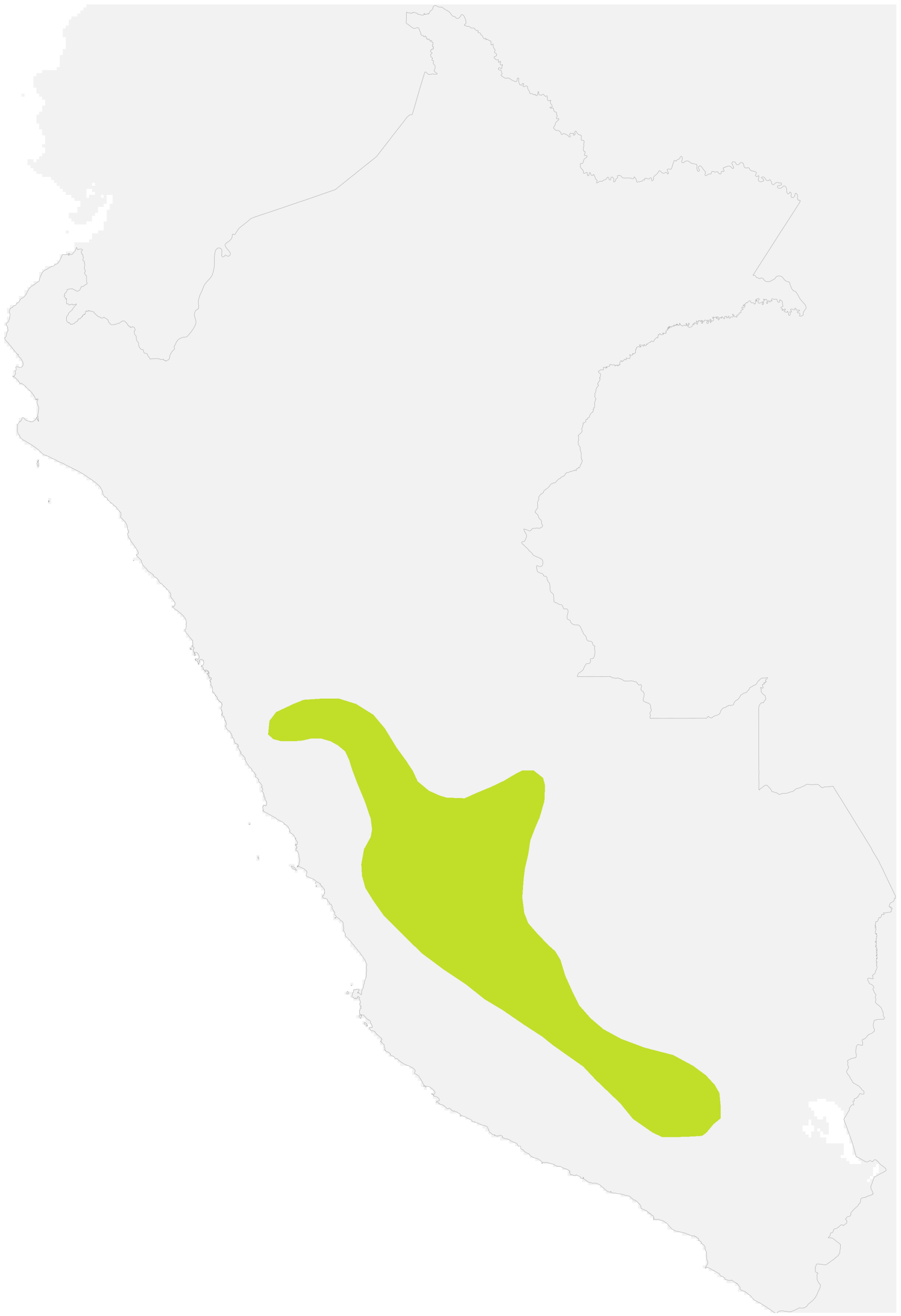

Current EOR

Bothrops pubescens

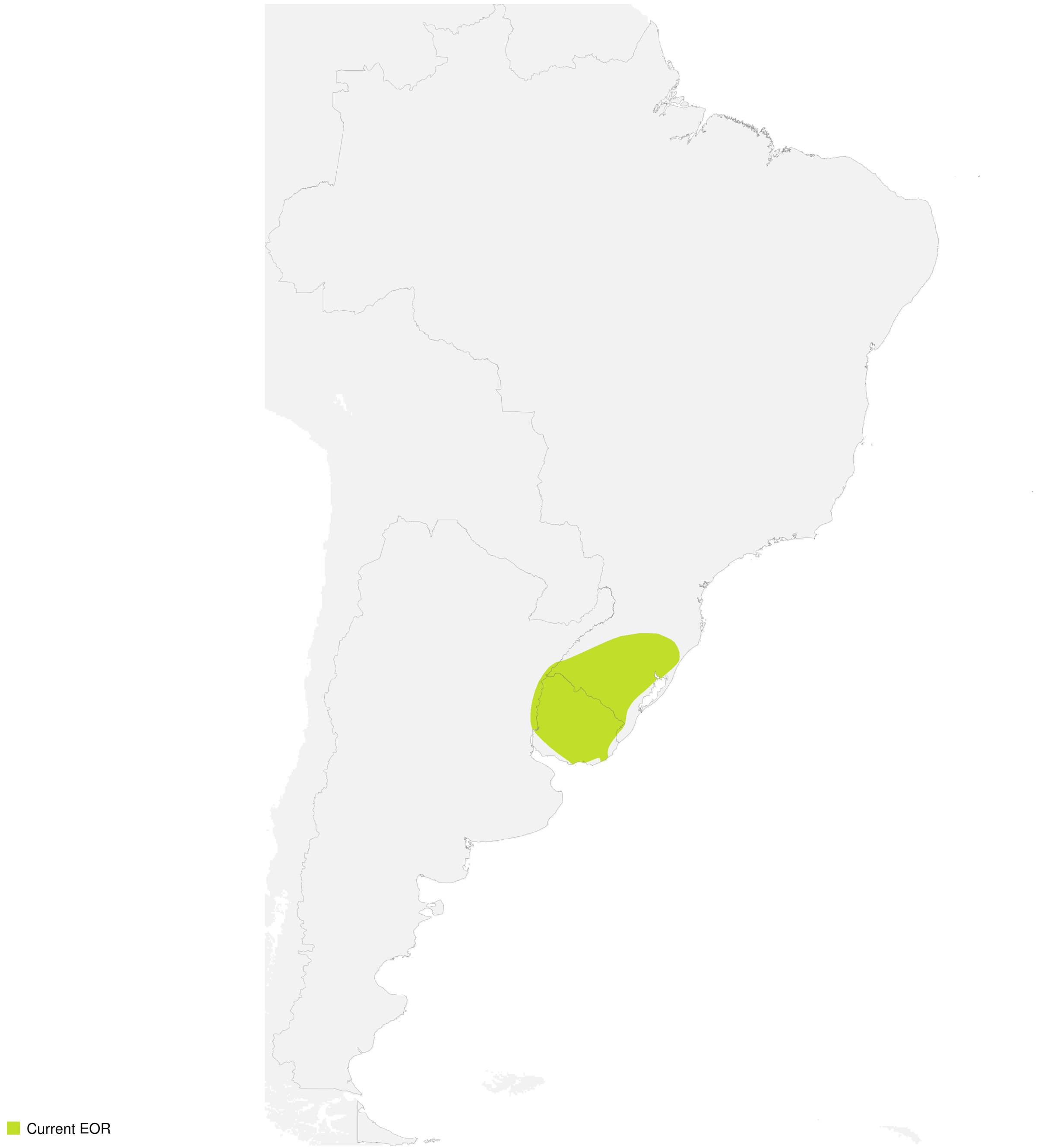

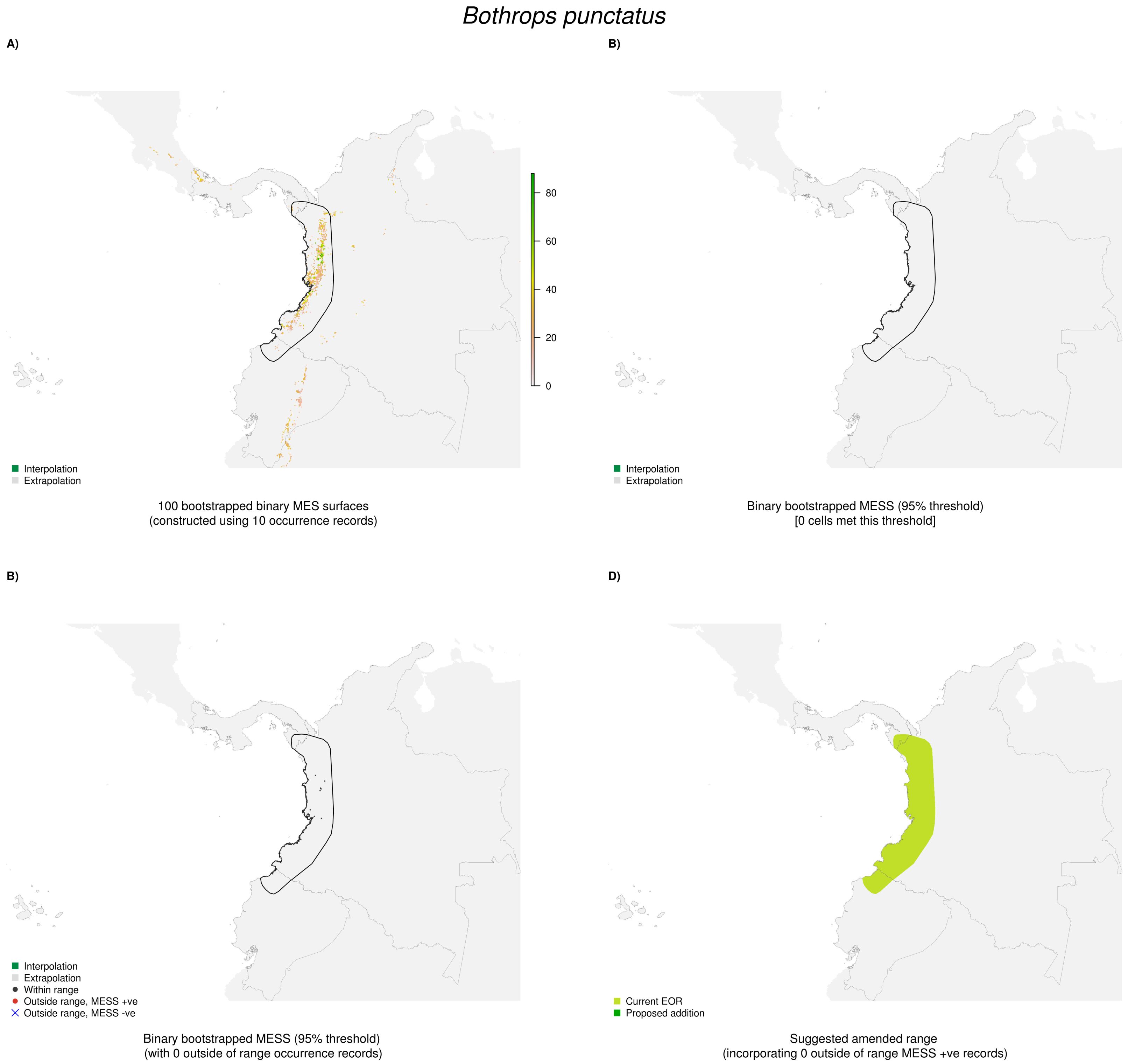

Bothrops punctatus
A)
B)
0
20
40
60
80
Interpolation
Extrapolation
100 bootstrapped binary MES surfaces
(constructed using 10 occurrence records)
Interpolation
Extrapolation
Binary bootstrapped MESS (95% threshold)
[0 cells met this threshold]
B)
D)
Interpolation
Extrapolation
Within range
Outside range, MESS +ve
Outside range, MESS -ve
Binary bootstrapped MESS (95% threshold)
(with 0 outside of range occurrence records)
Current EOR
Proposed addition
Suggested amended range
(incorporating 0 outside of range MESS +ve records)

Bothrops roedingeri

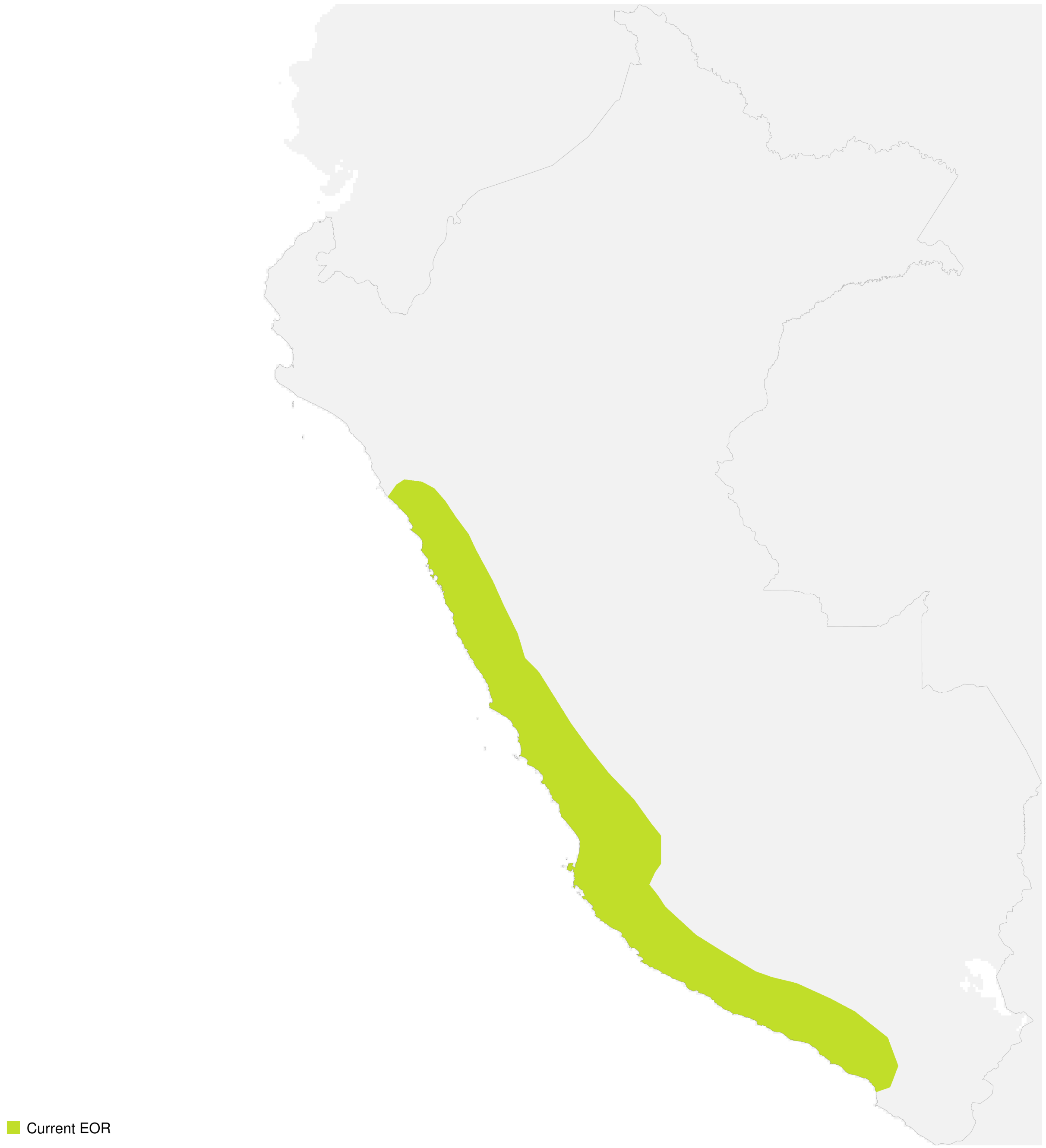

Bothrops sanctaecrucis

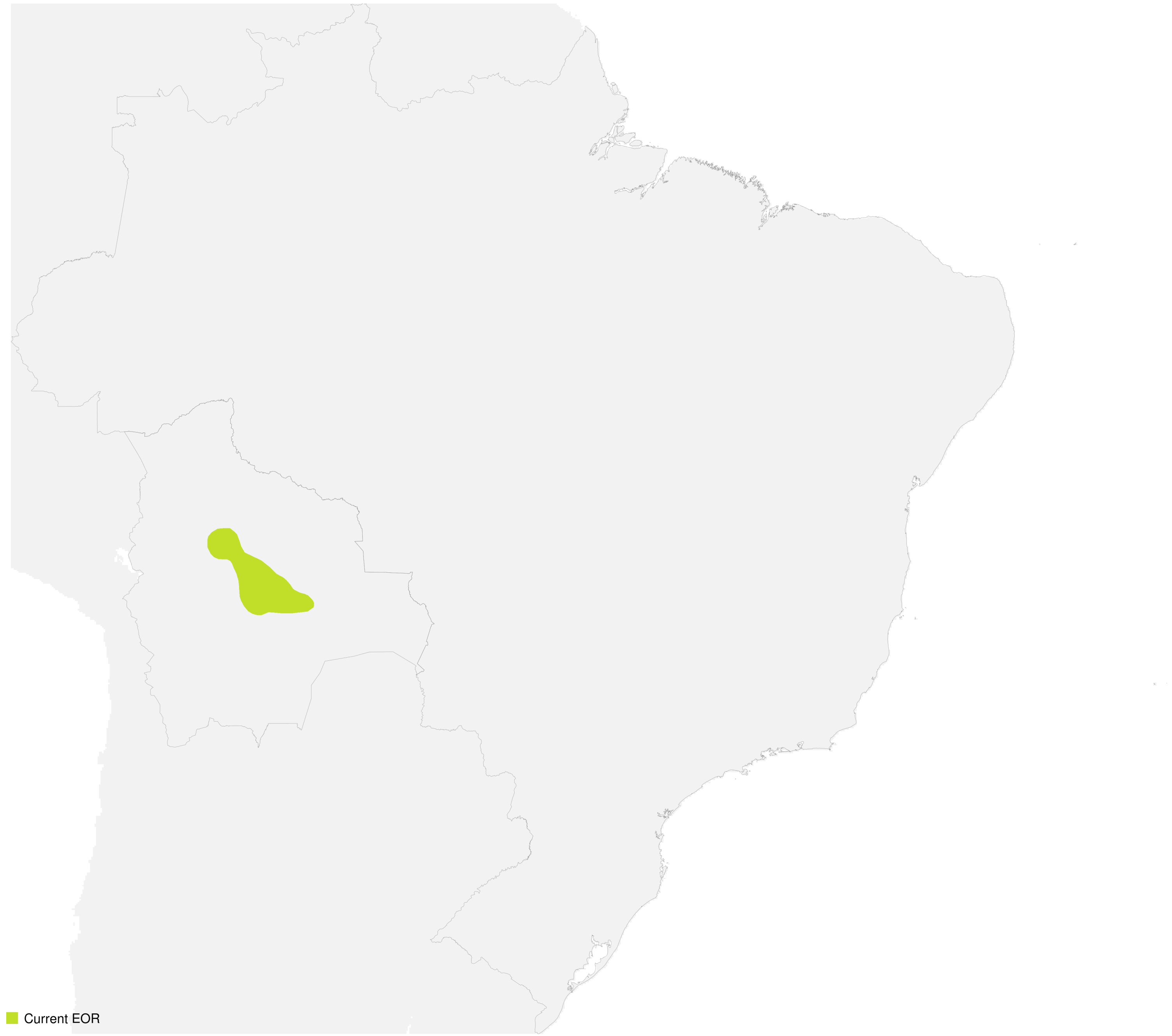

Bothrops taeniatus

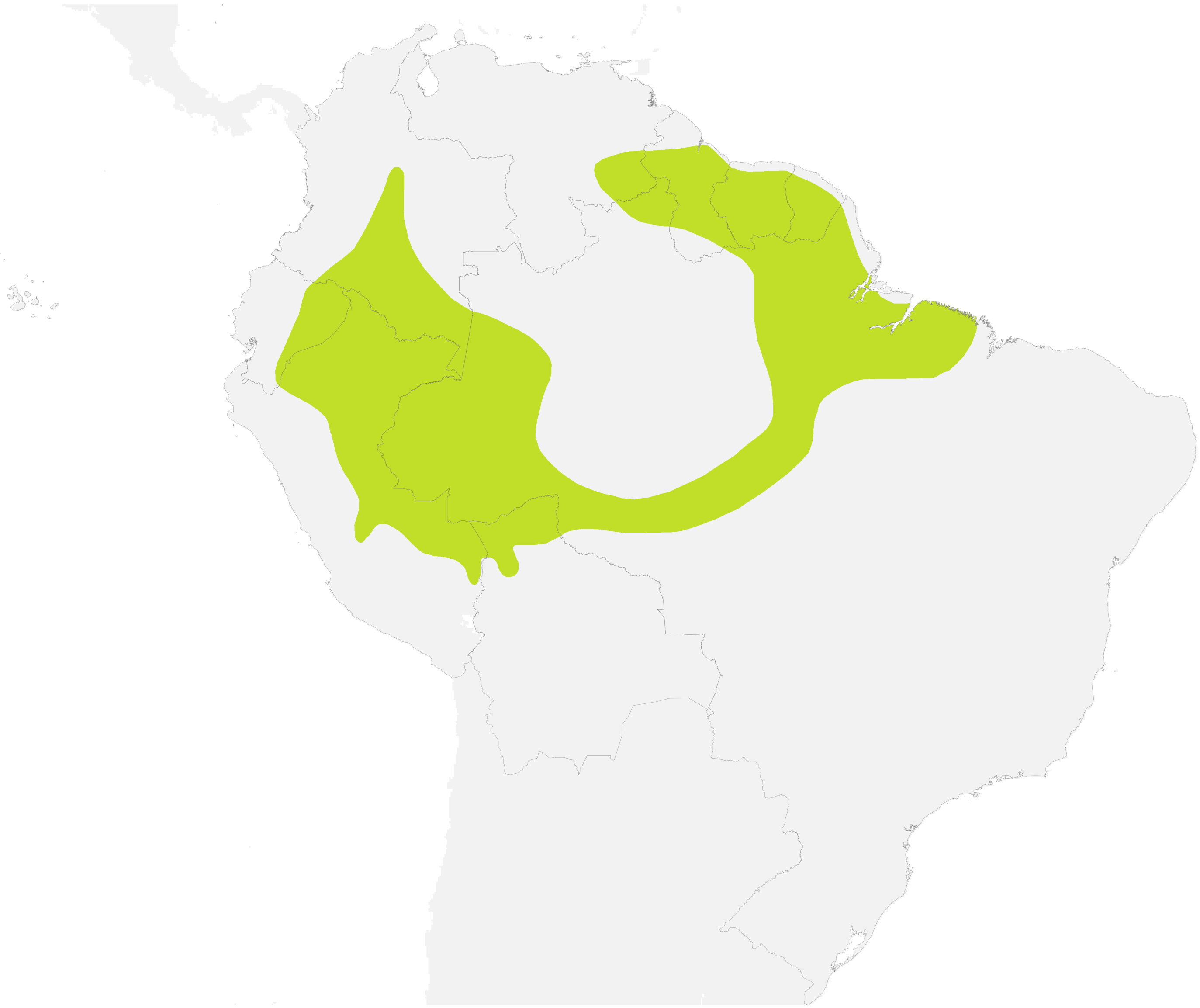

Current EOR

Bothrops venezuelensis

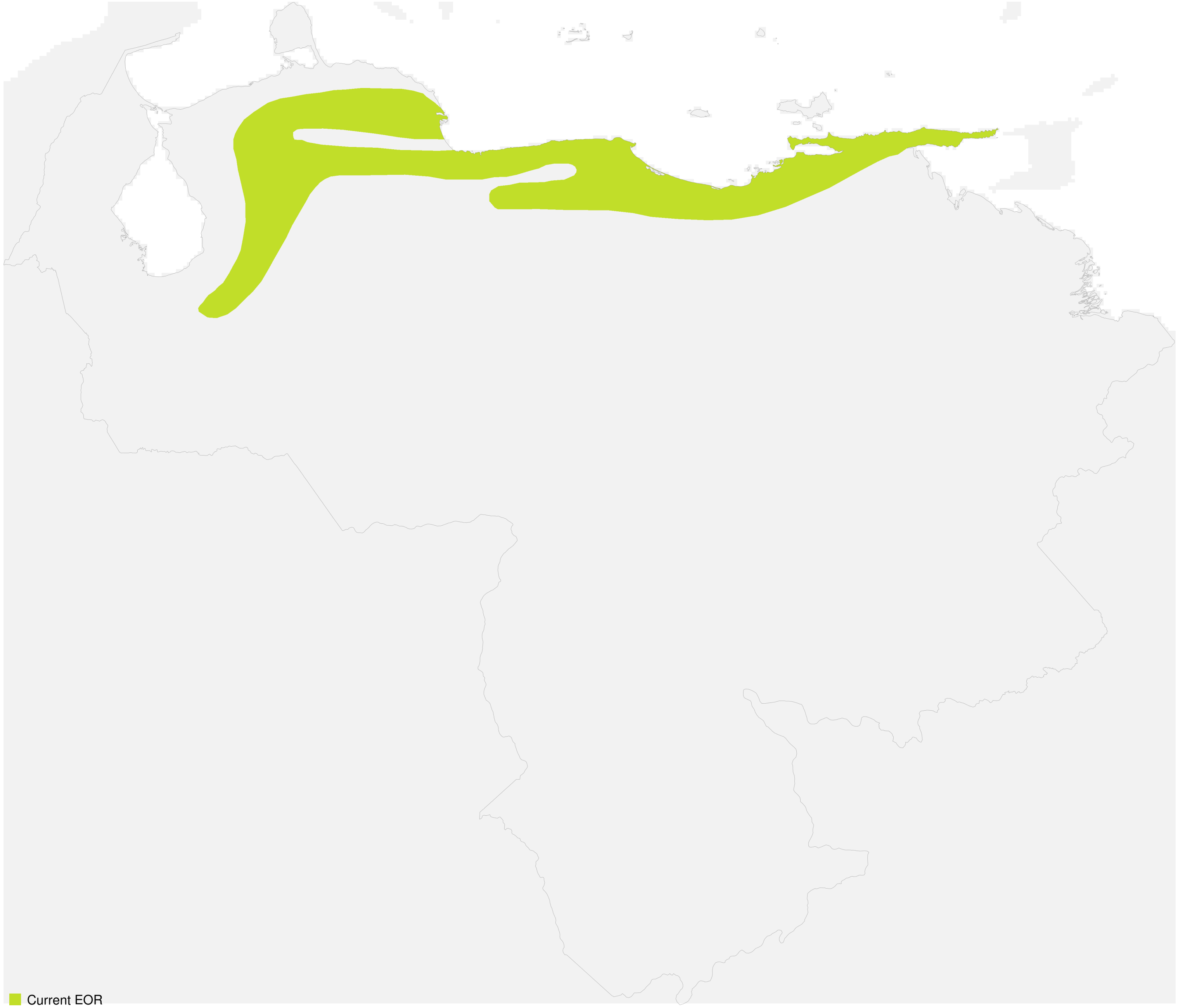

Bungarus bungaroides

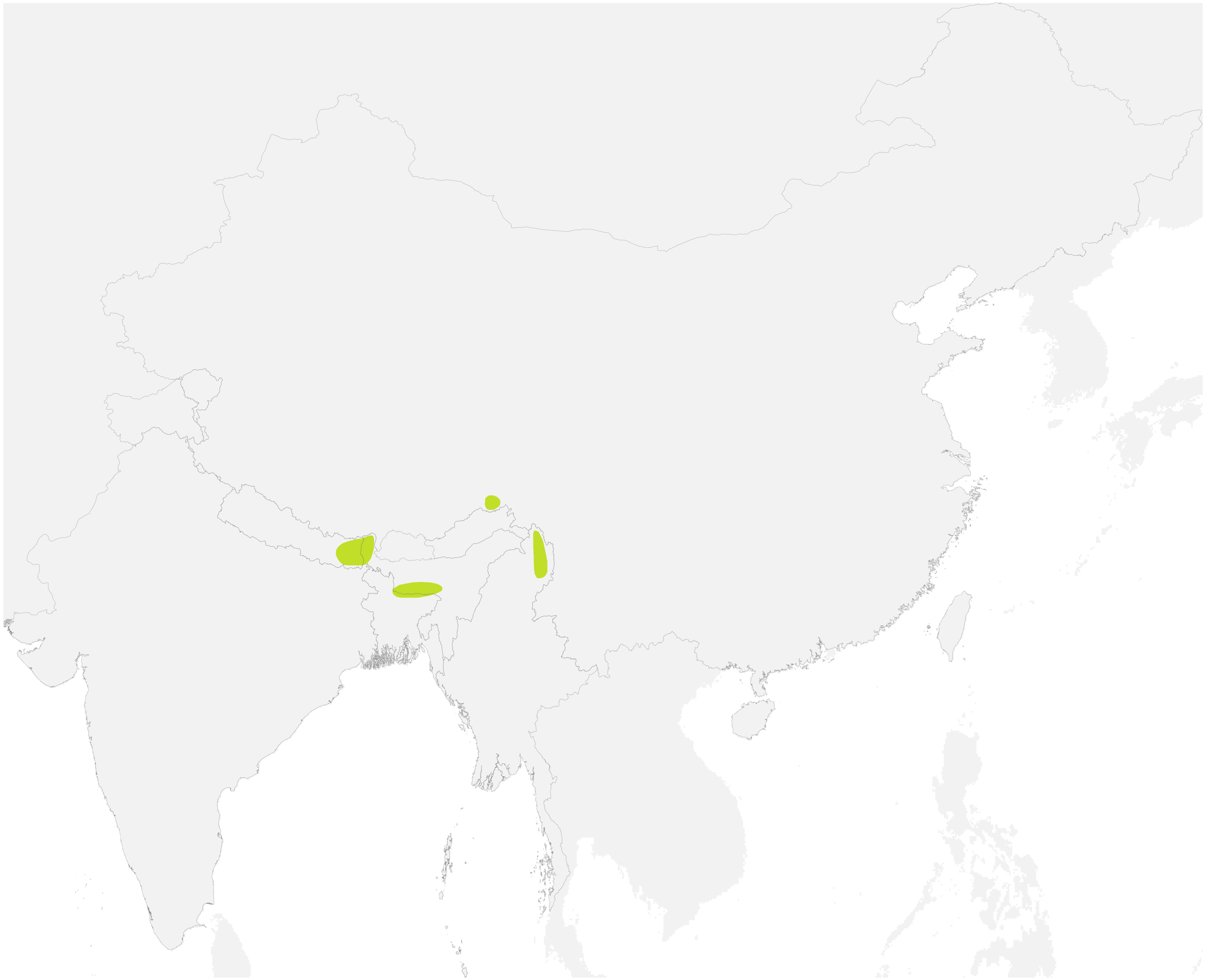

Current EOR

Bungarus caeruleus

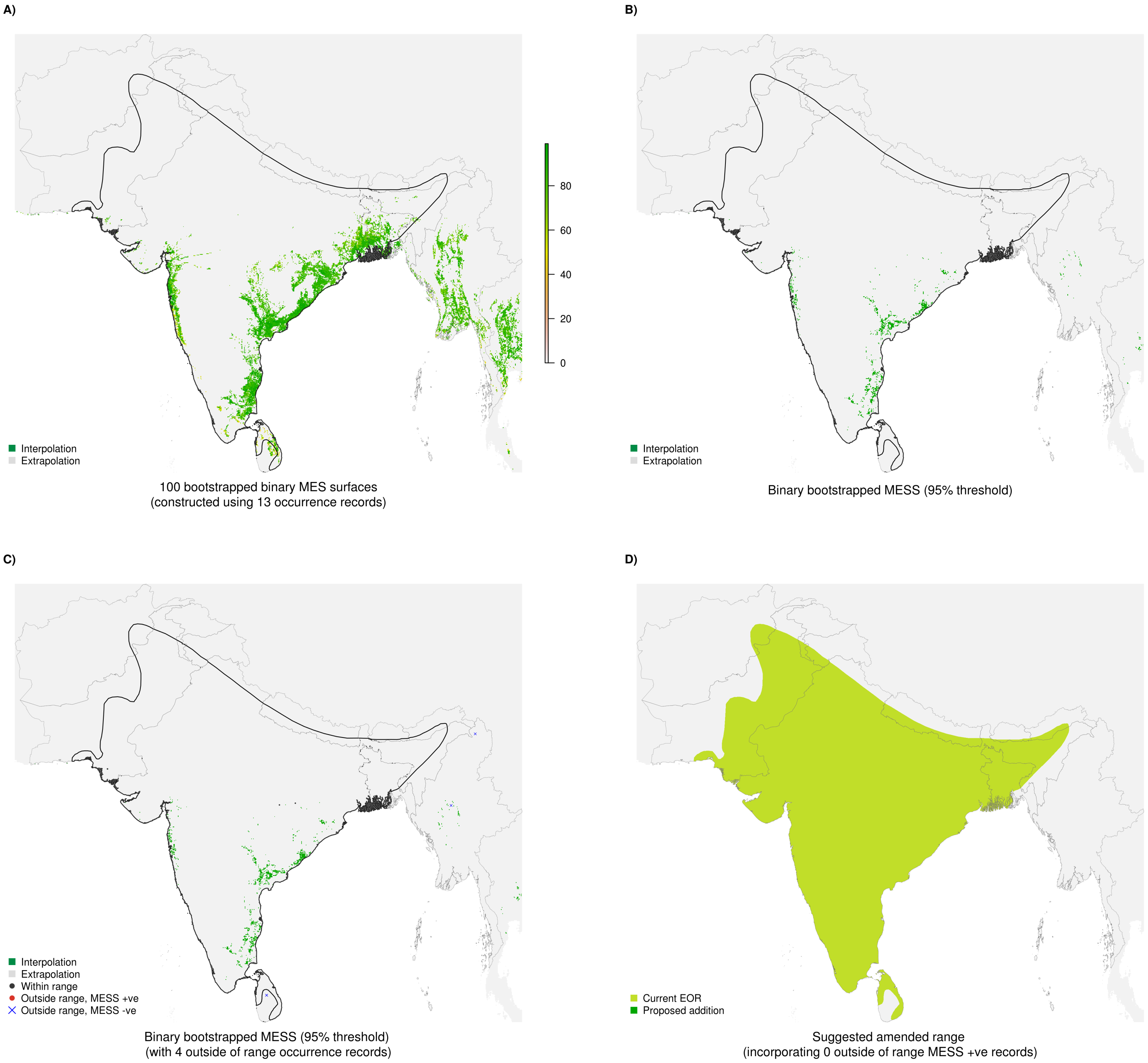

Bungarus candidus

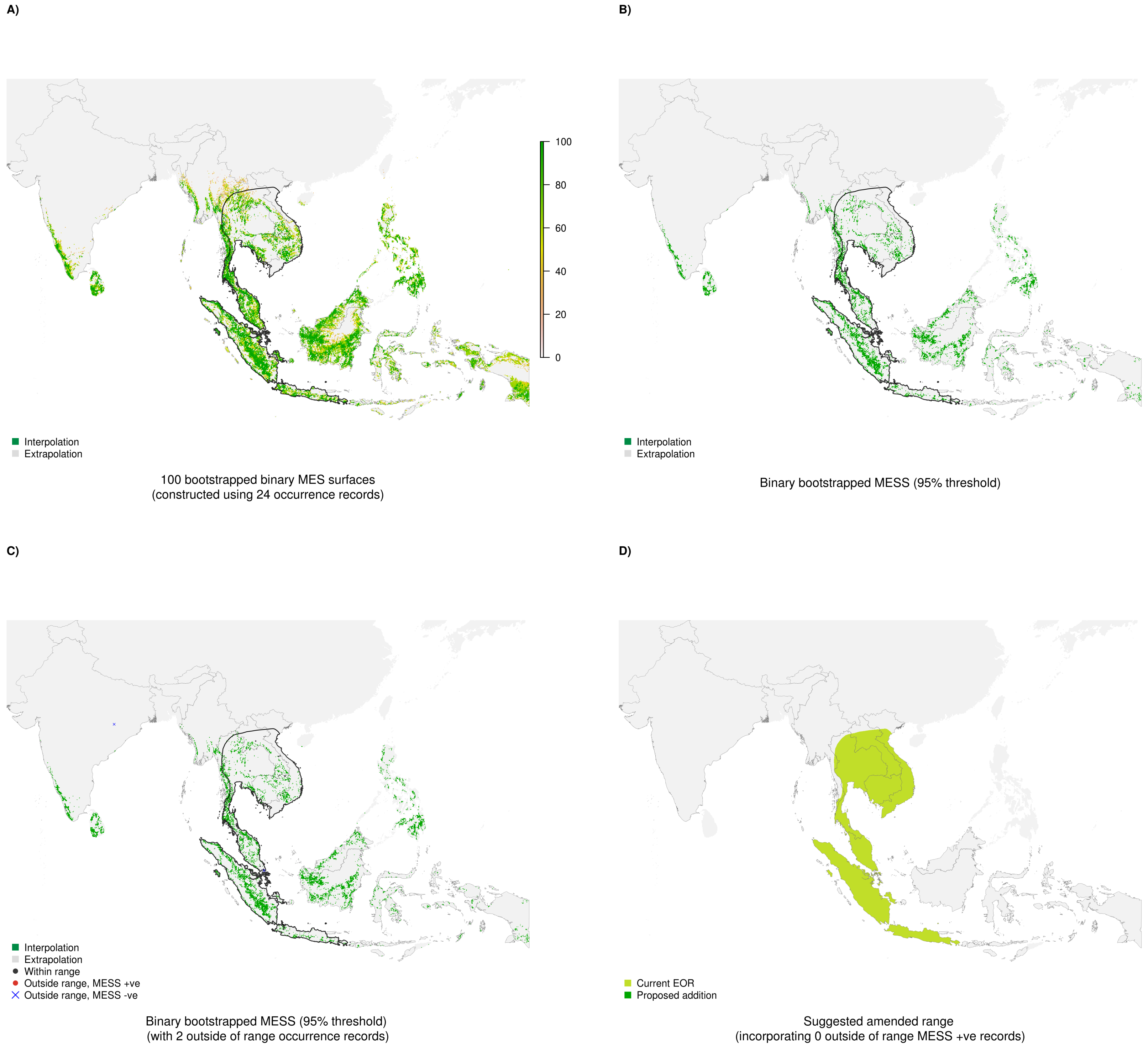

Bungarus ceylonicus

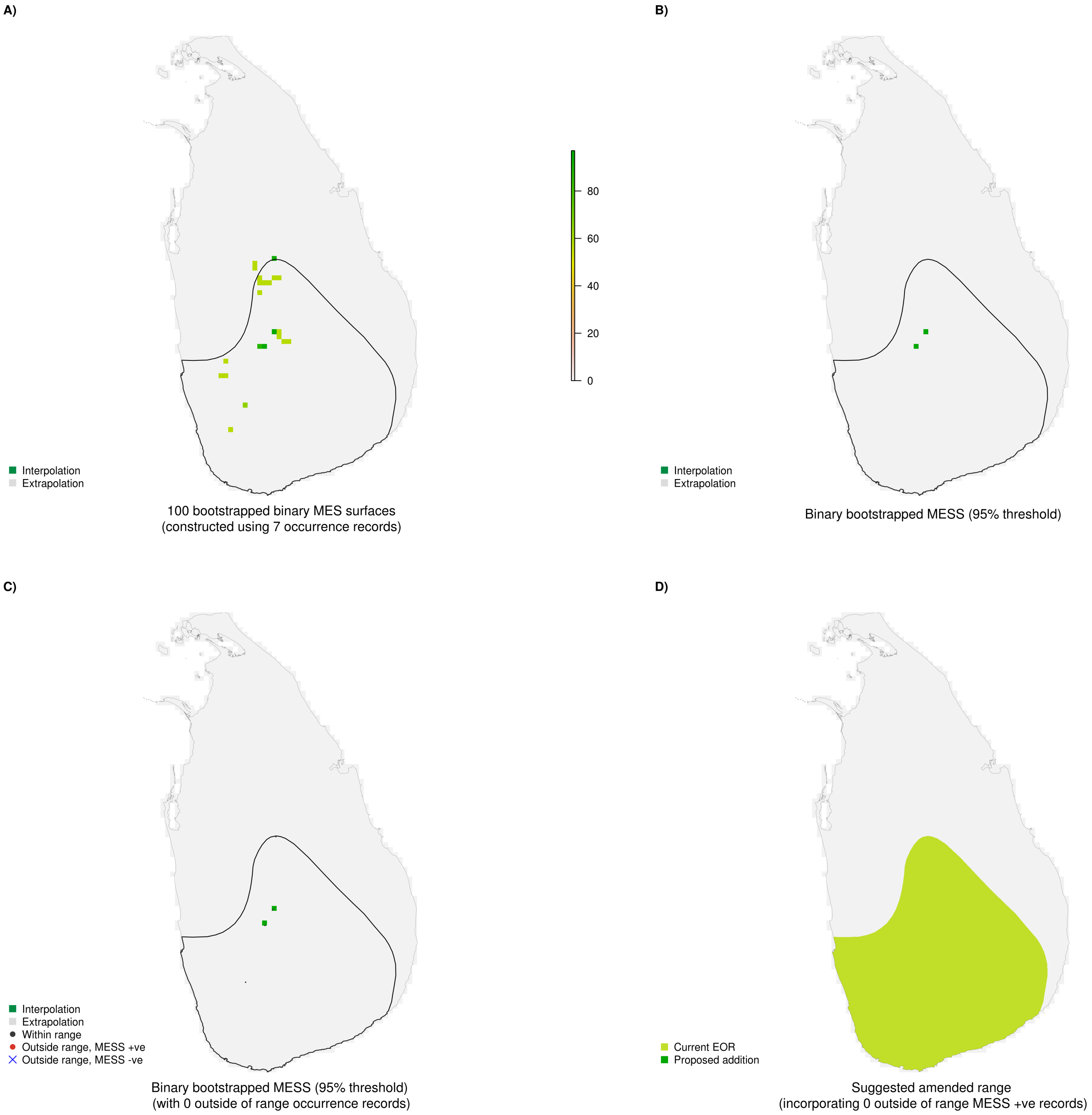

Bungarus fasciatus

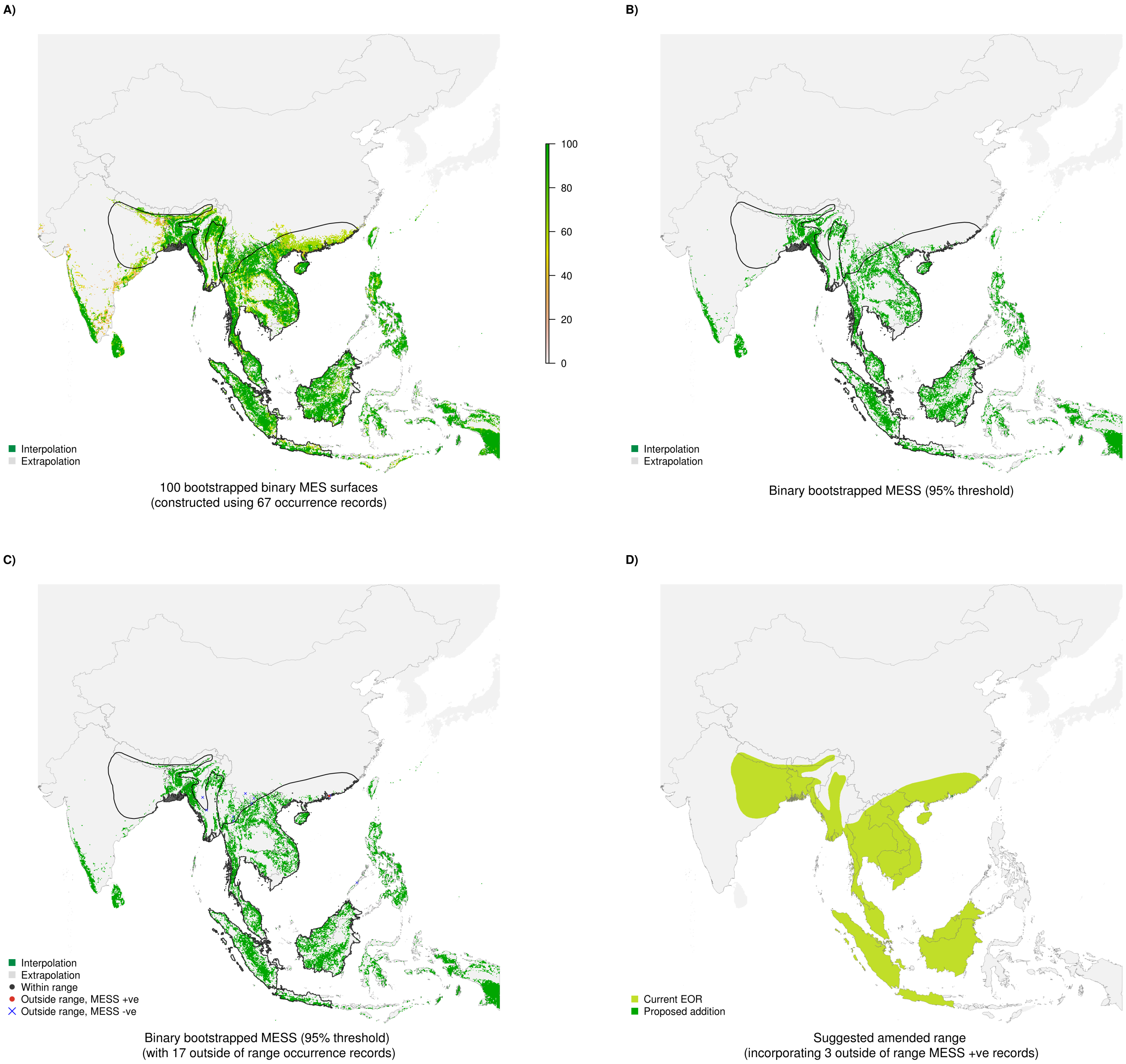

Bungarus flaviceps

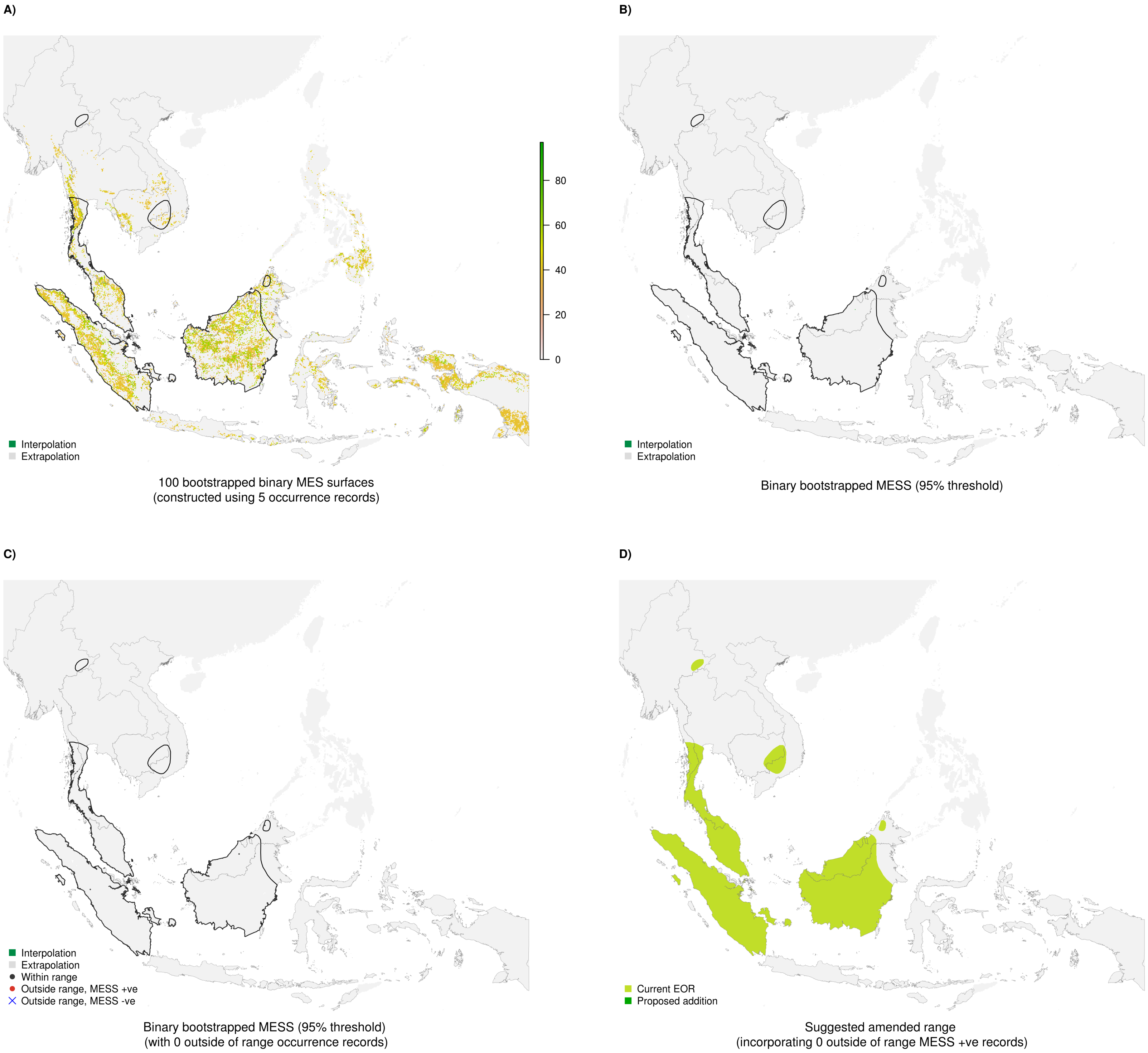

Bungarus lividus

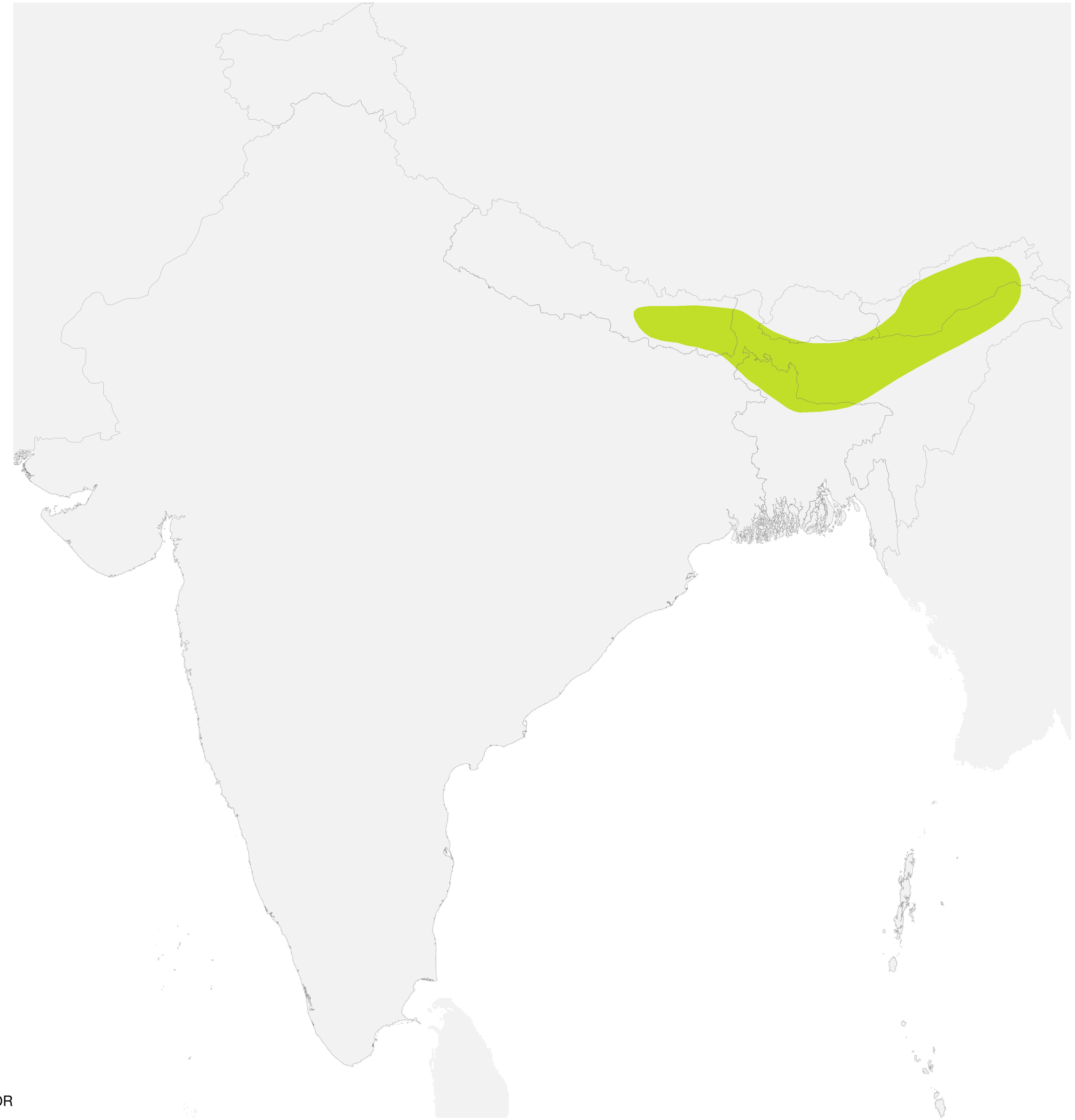

Current EOR

Bungarus magnimaculatus

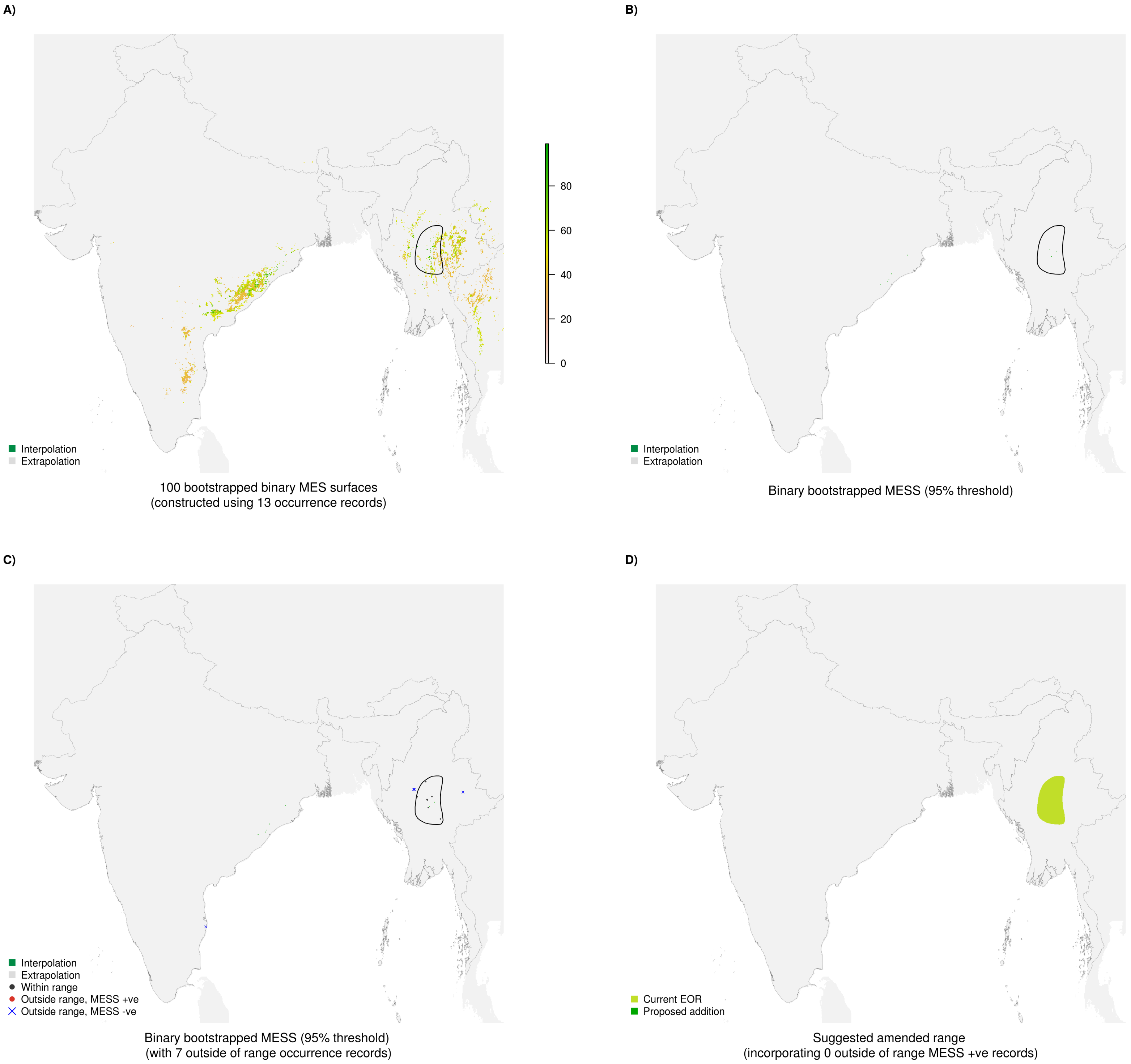

Bungarus multicinctus

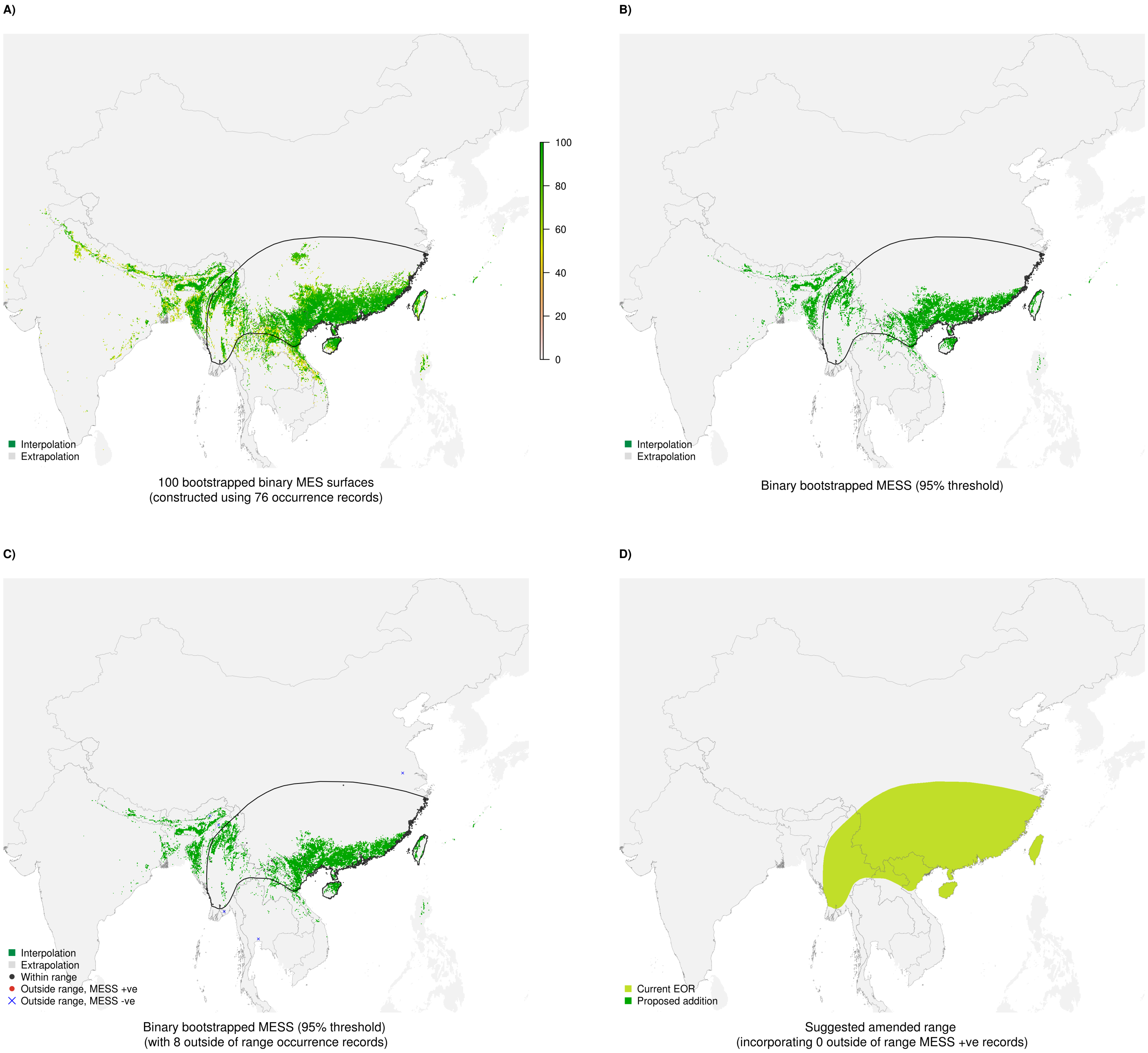

Bungarus niger

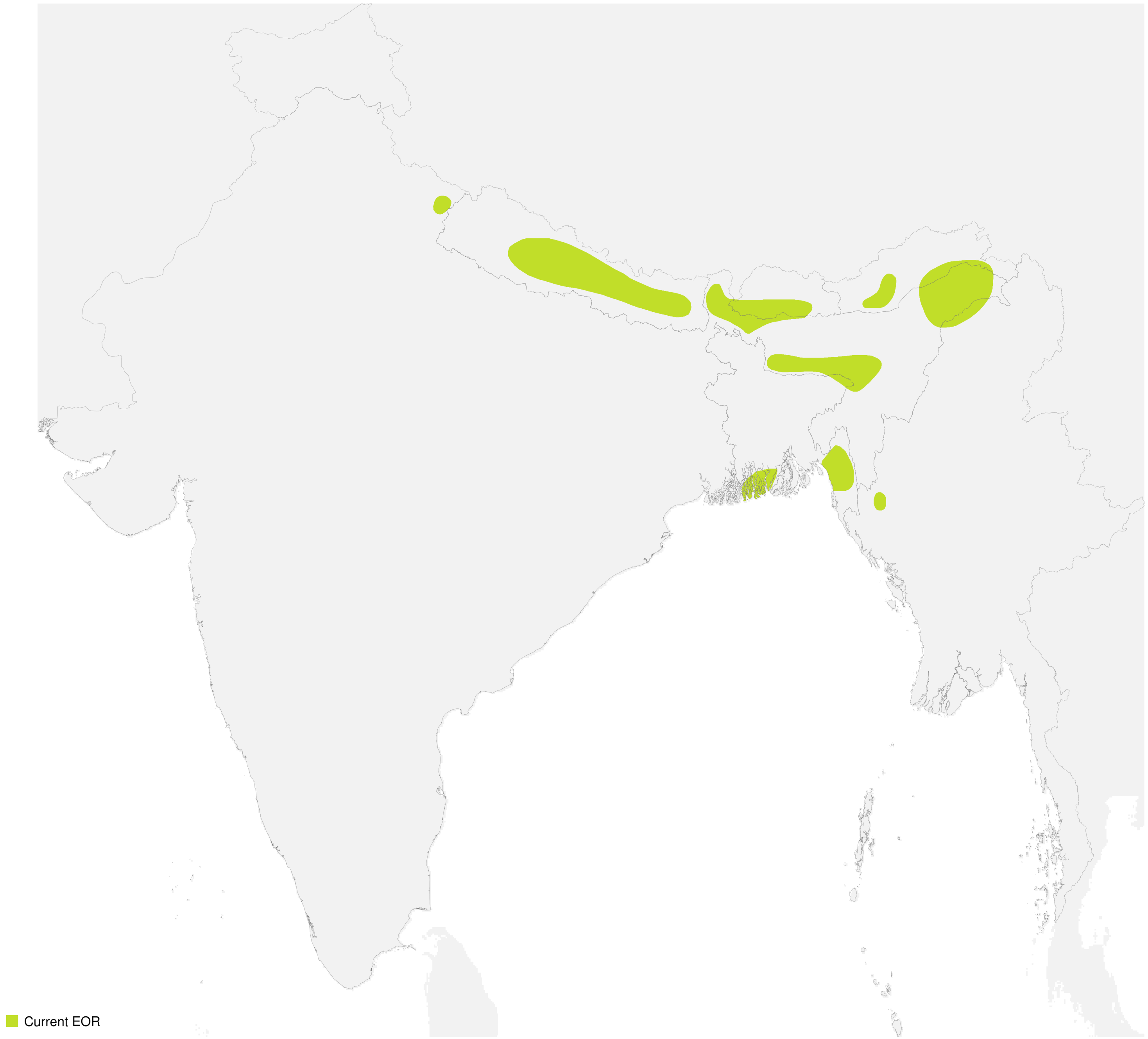

Bungarus sindanus

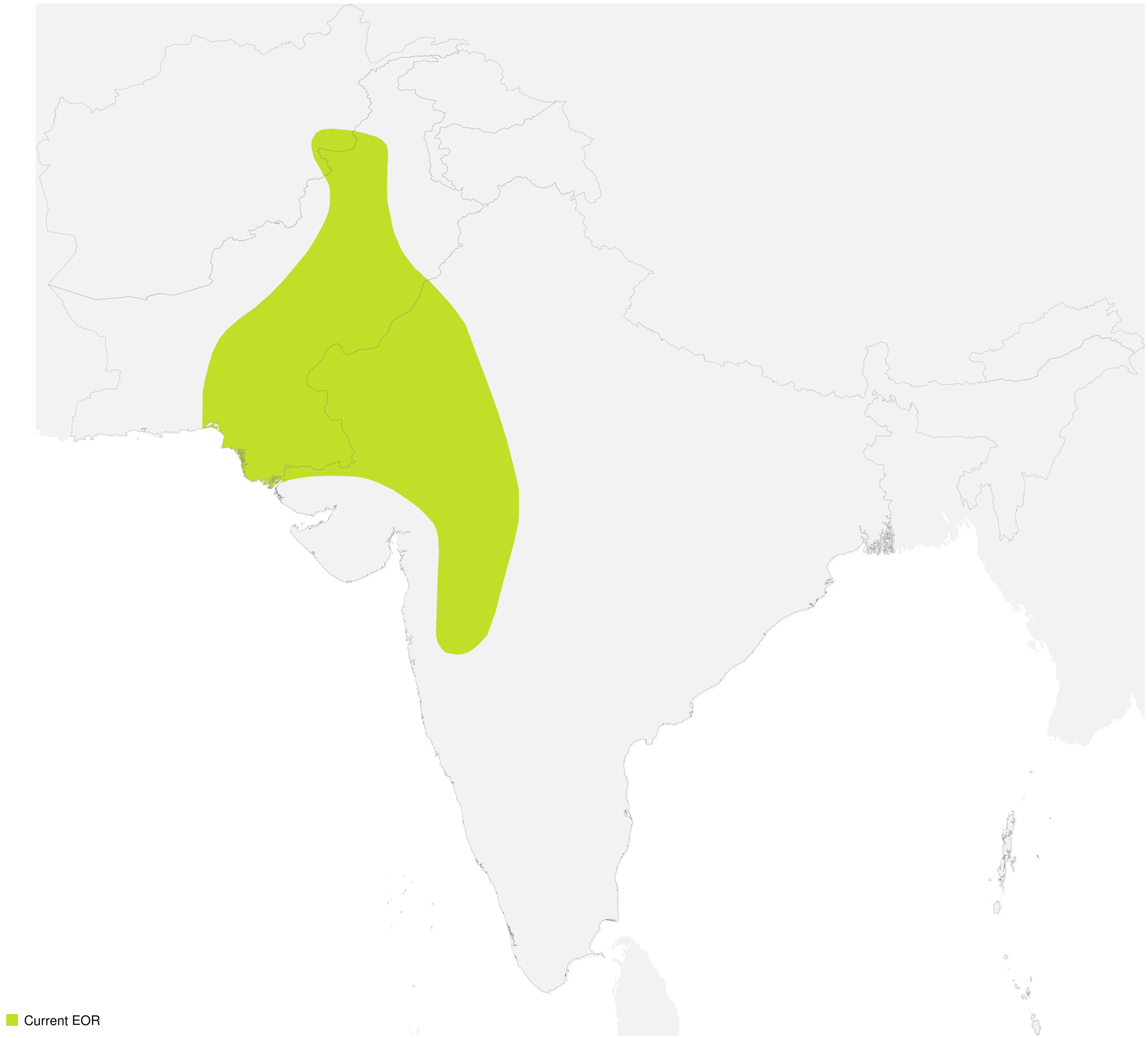

Bungarus slowinskii

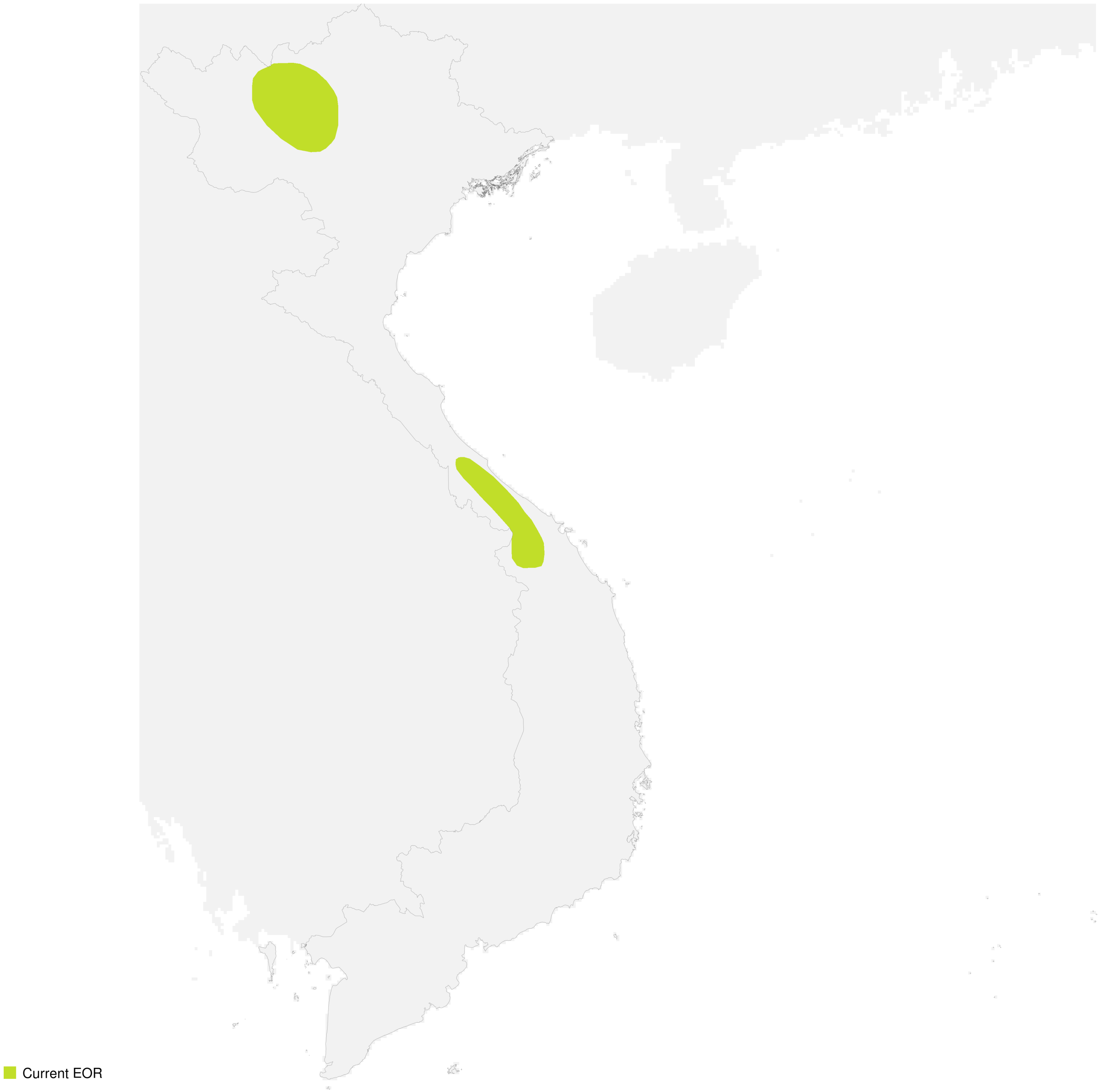

Bungarus walli

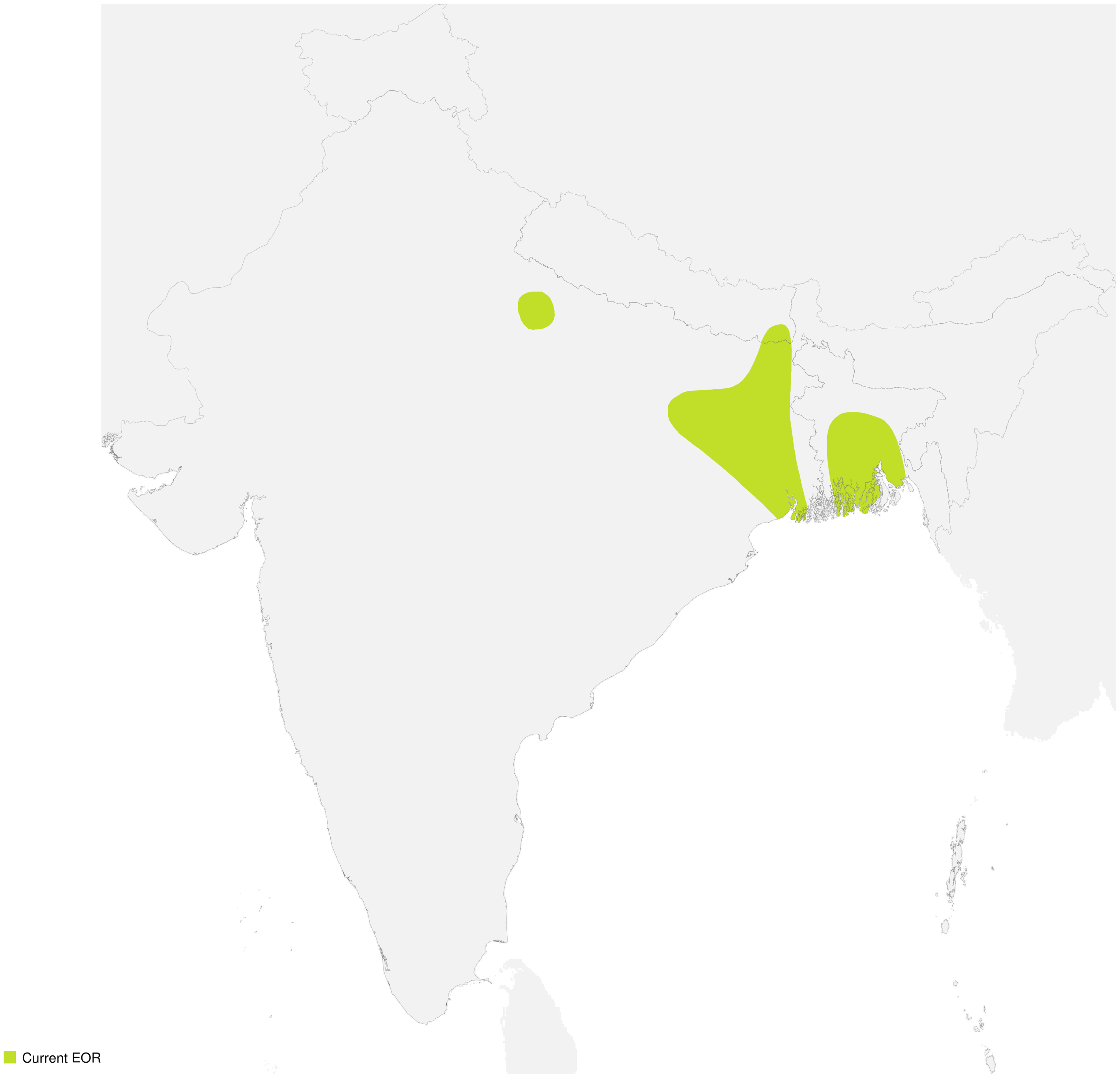

Calliophis bivirgatus

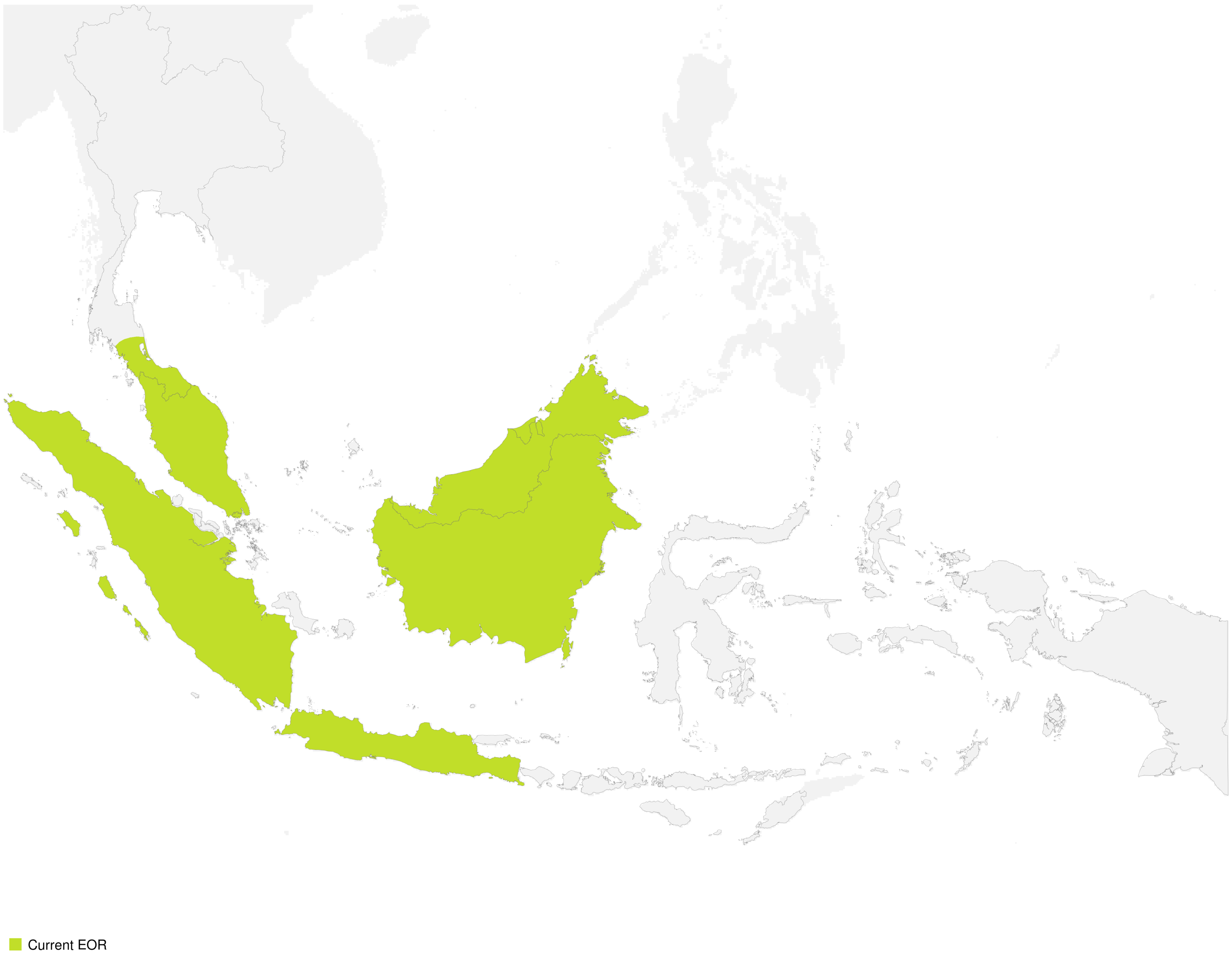

Calliophis intestinalis

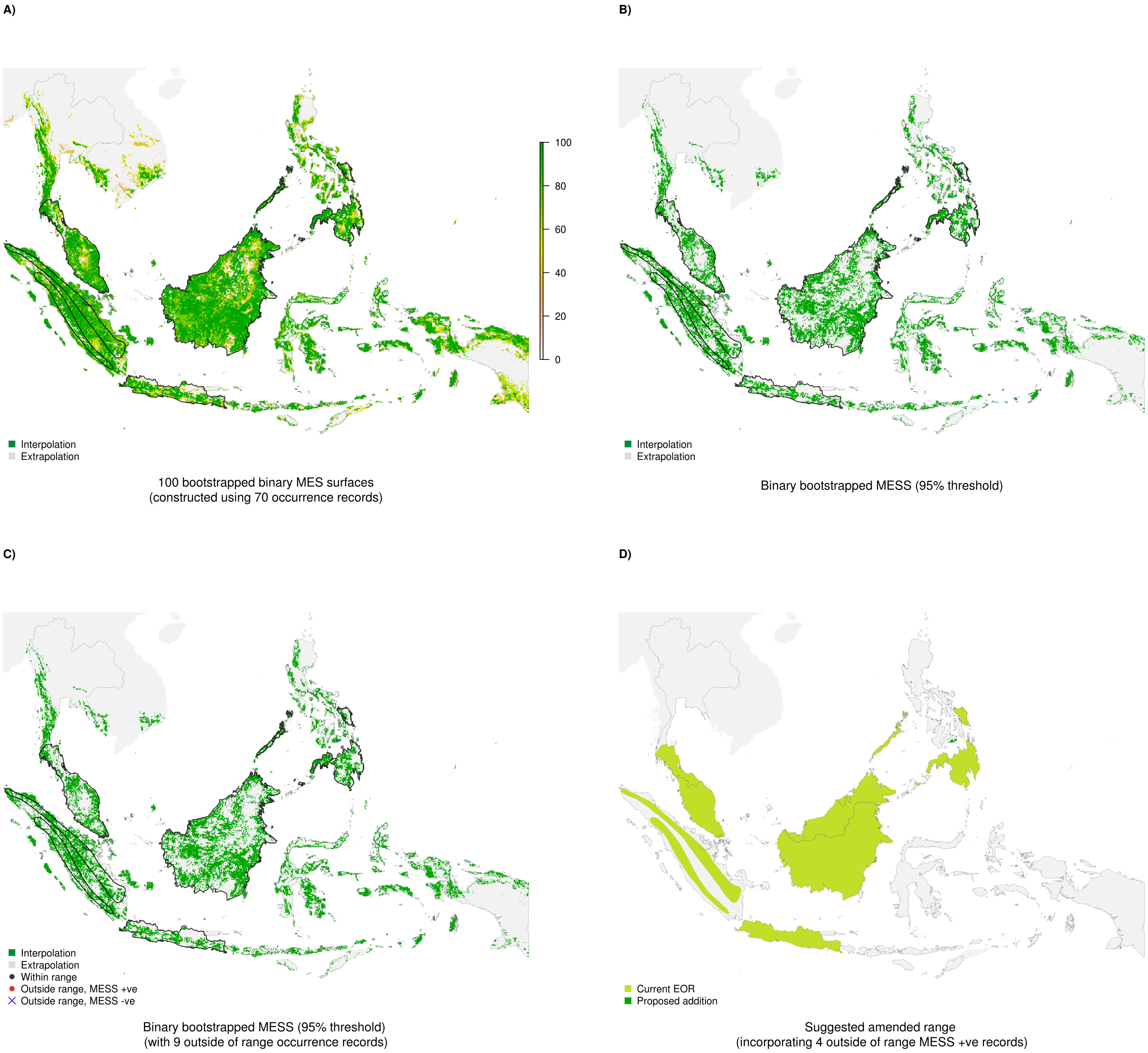

Calloselasma rhodostoma

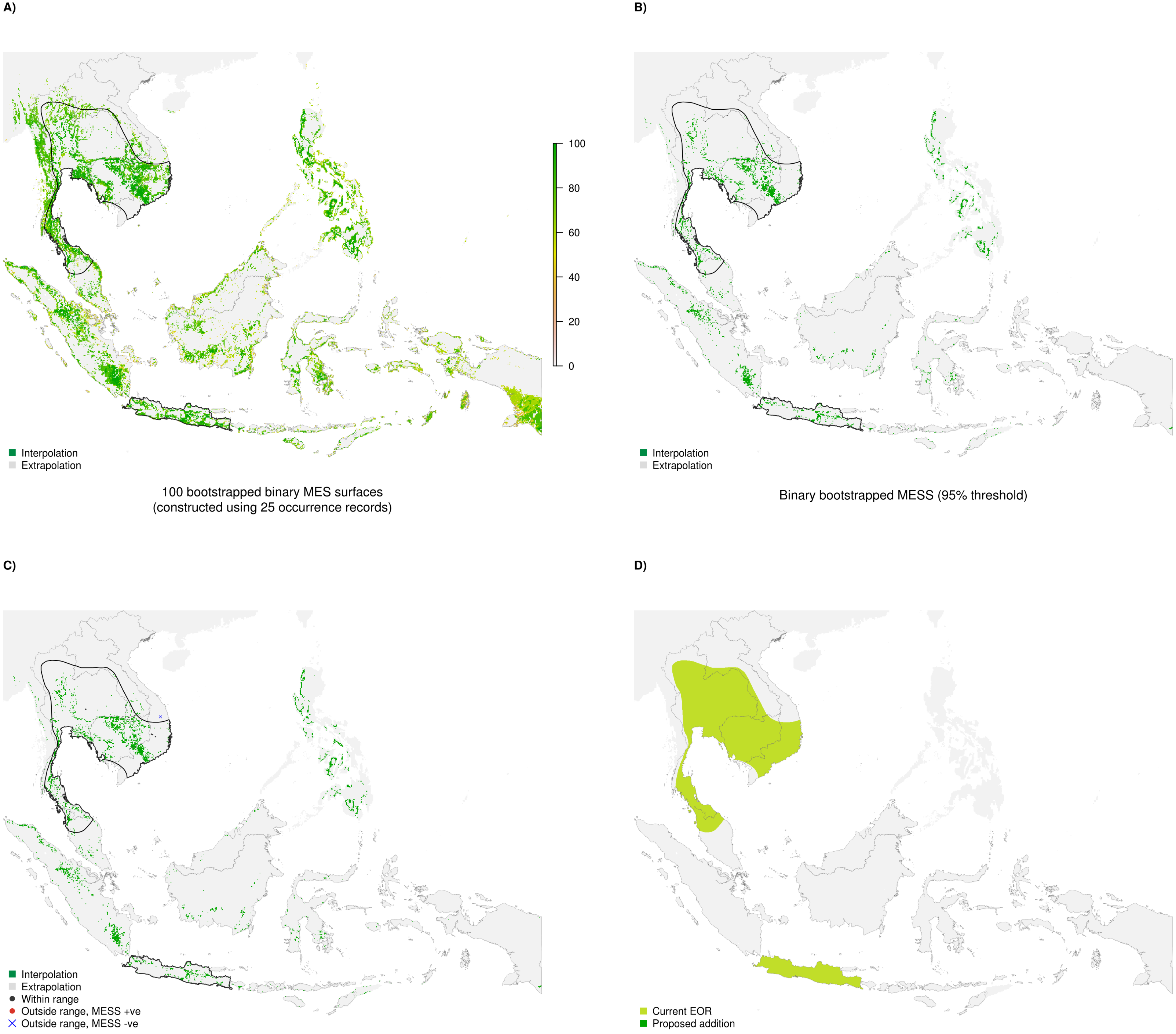

A) 100 bootstrapped binary MES surfaces (constructed using 25 occurrence records)

B) Binary bootstrapped MESS (95% threshold)

C) Binary bootstrapped MESS (95% threshold) (with 2 outside of range occurrence records)

D) Suggested amended range (incorporating 0 outside of range MESS +ve records)

Cerastes cerastes

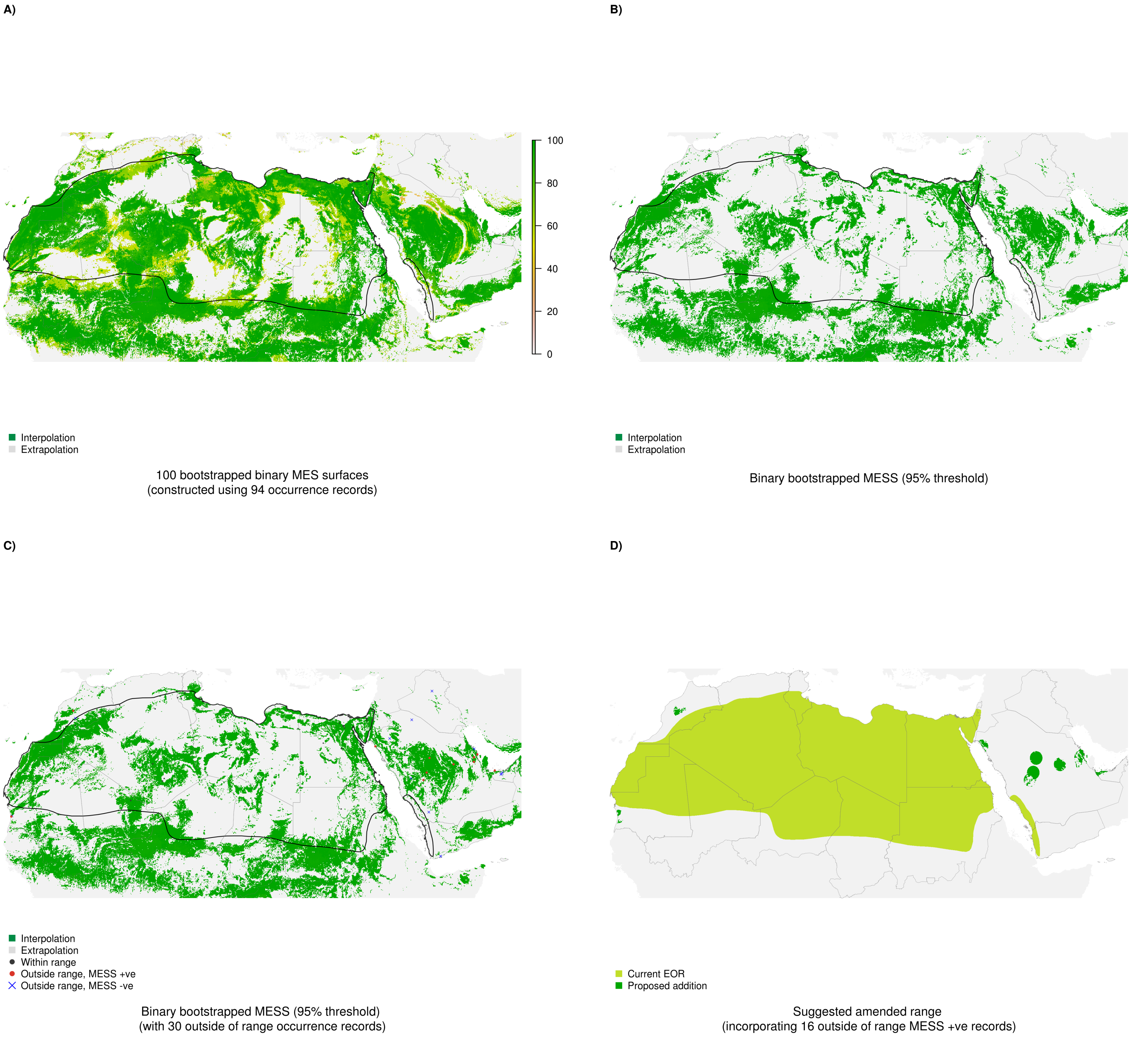

Cerastes gasperettii

A)

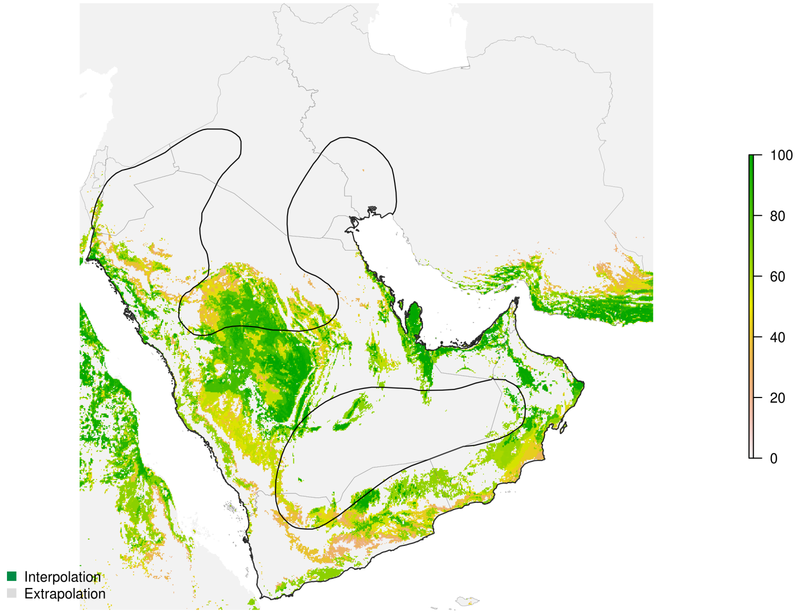

100 bootstrapped binary MES surfaces
(constructed using 23 occurrence records)

B)

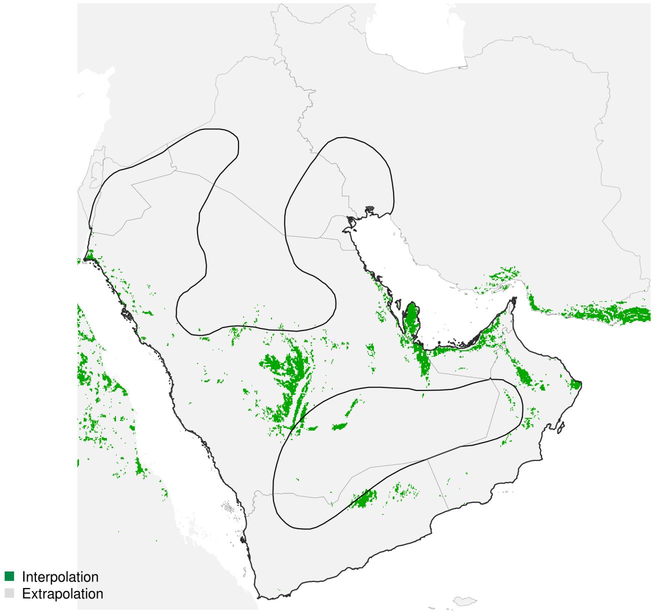

Binary bootstrapped MESS (95% threshold)

C)

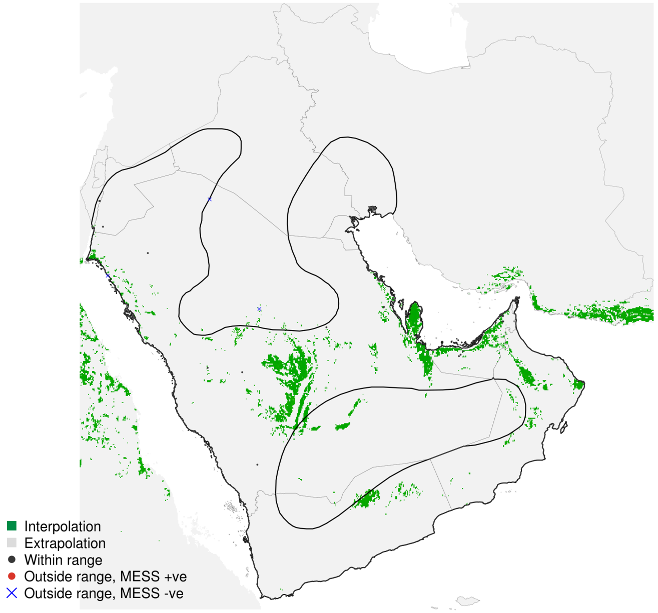

Binary bootstrapped MESS (95% threshold)
(with 3 outside of range occurrence records)

D)

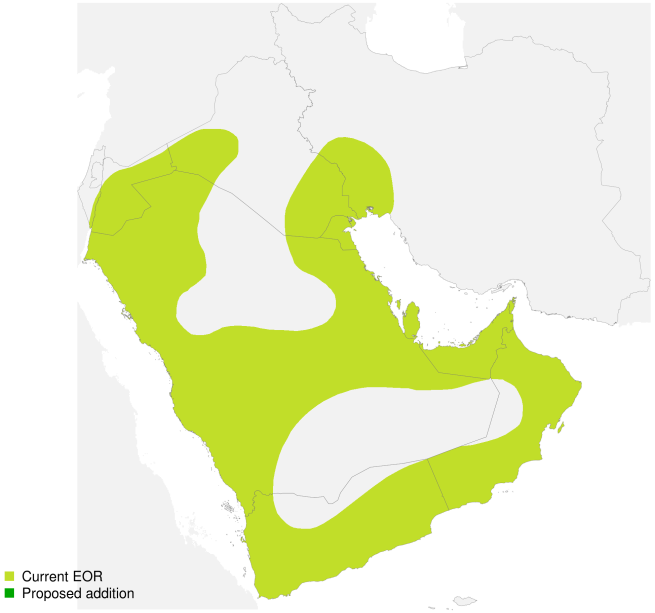

Suggested amended range
(incorporating 0 outside of range MESS +ve records)

Cerrophidion barbouri

A)

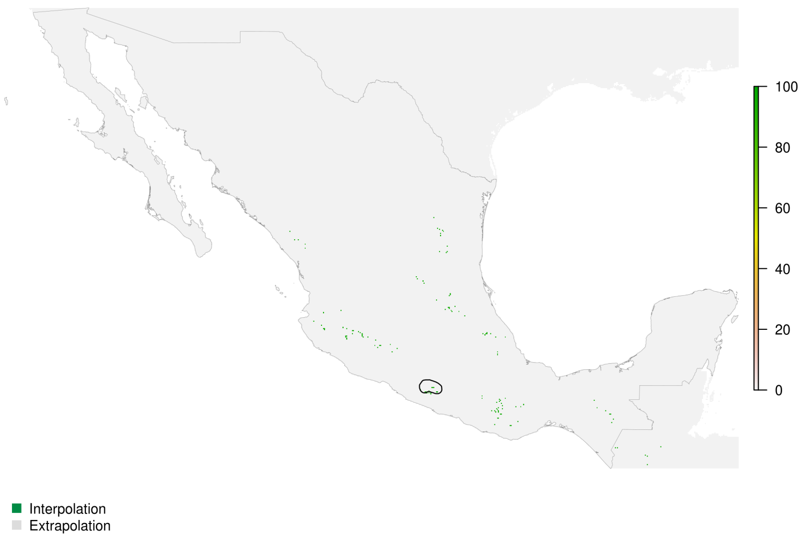

100 bootstrapped binary MES surfaces
(constructed using 15 occurrence records)

B)

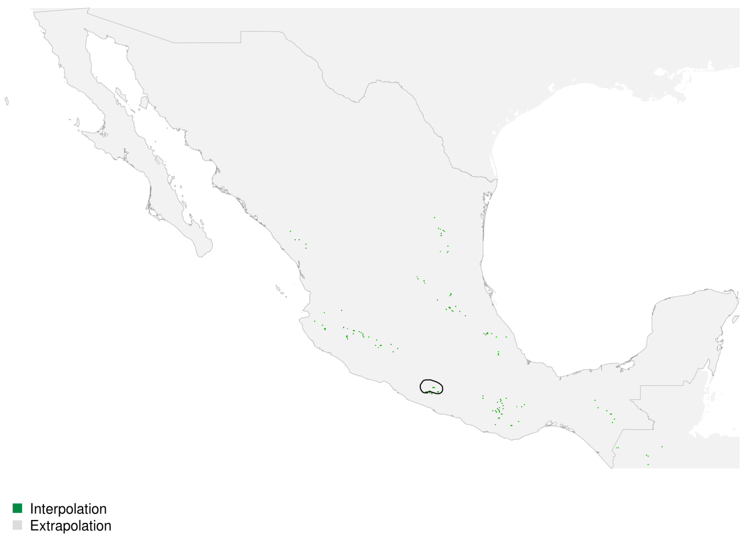

Binary bootstrapped MESS (95% threshold)

C)

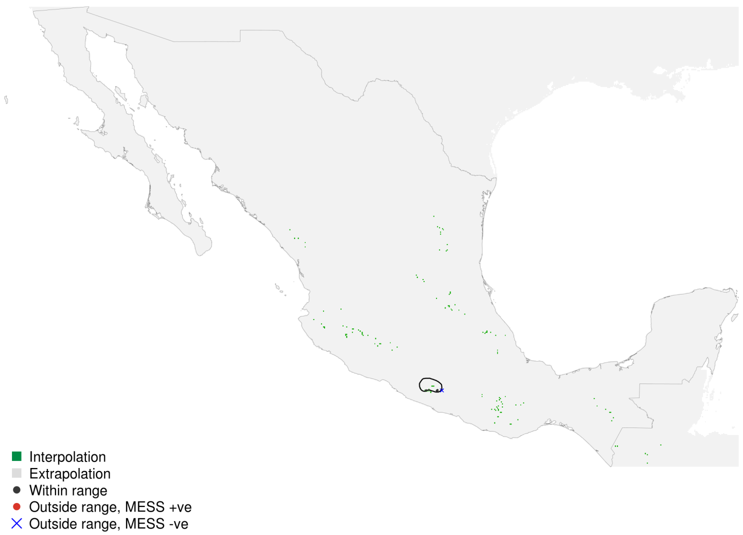

Binary bootstrapped MESS (95% threshold)
(with 4 outside of range occurrence records)

D)

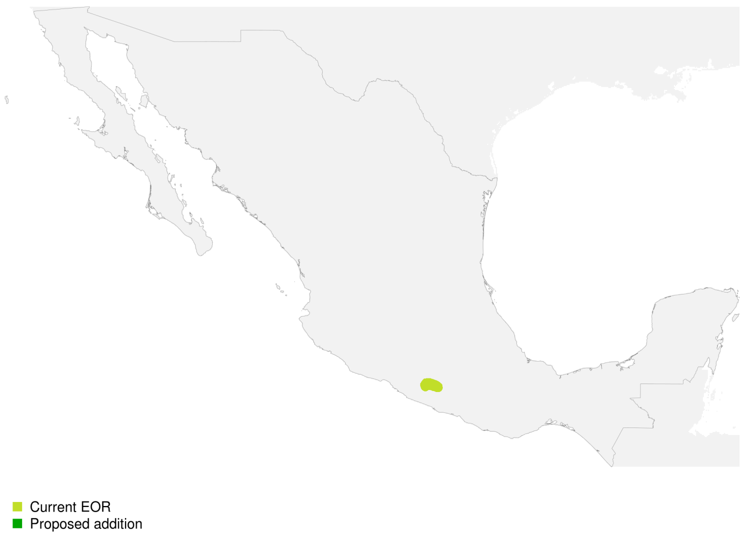

Suggested amended range
(incorporating 0 outside of range MESS +ve records)

Cerrophidion godmani

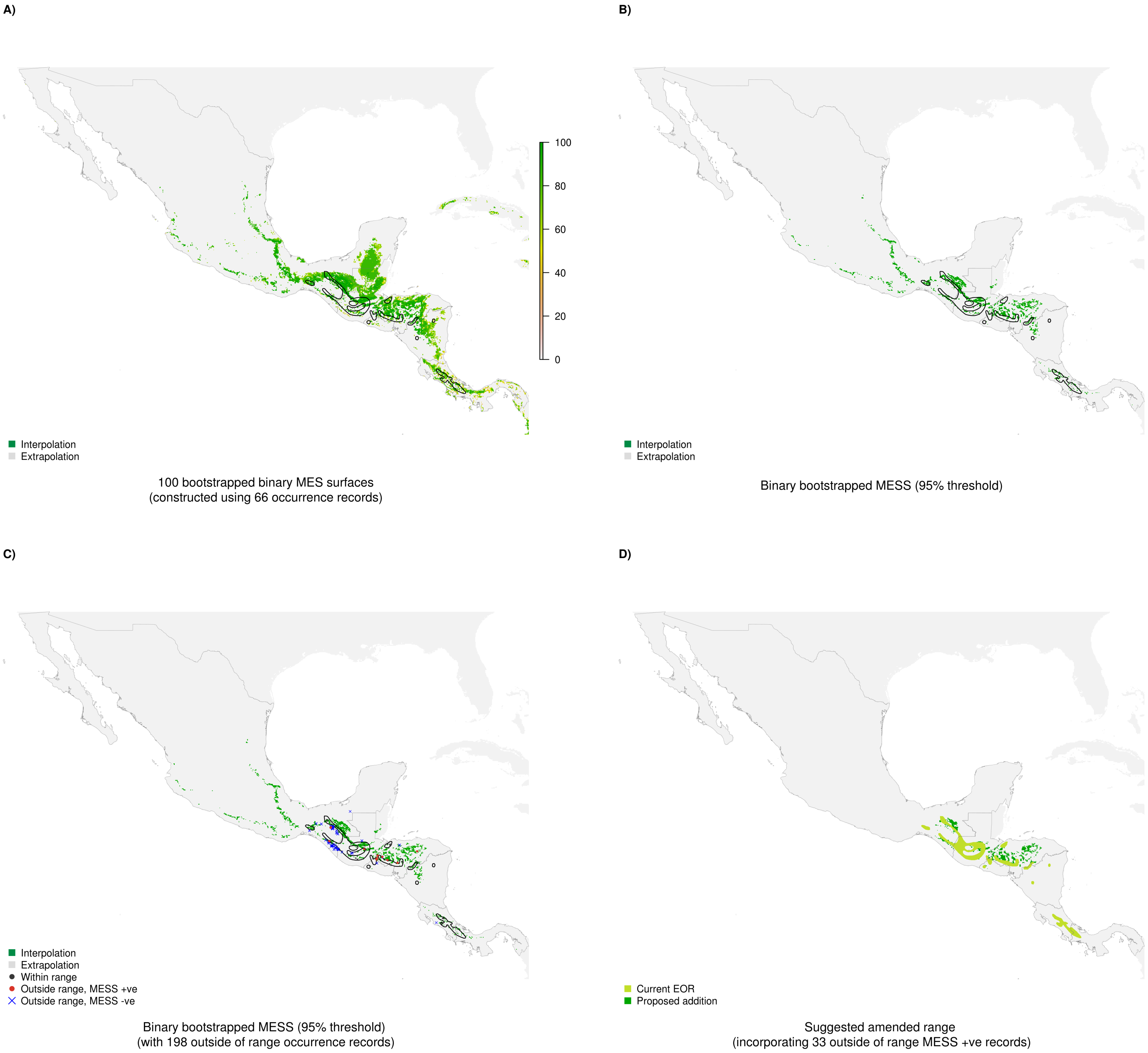

Cerrophidion petlalcalensis

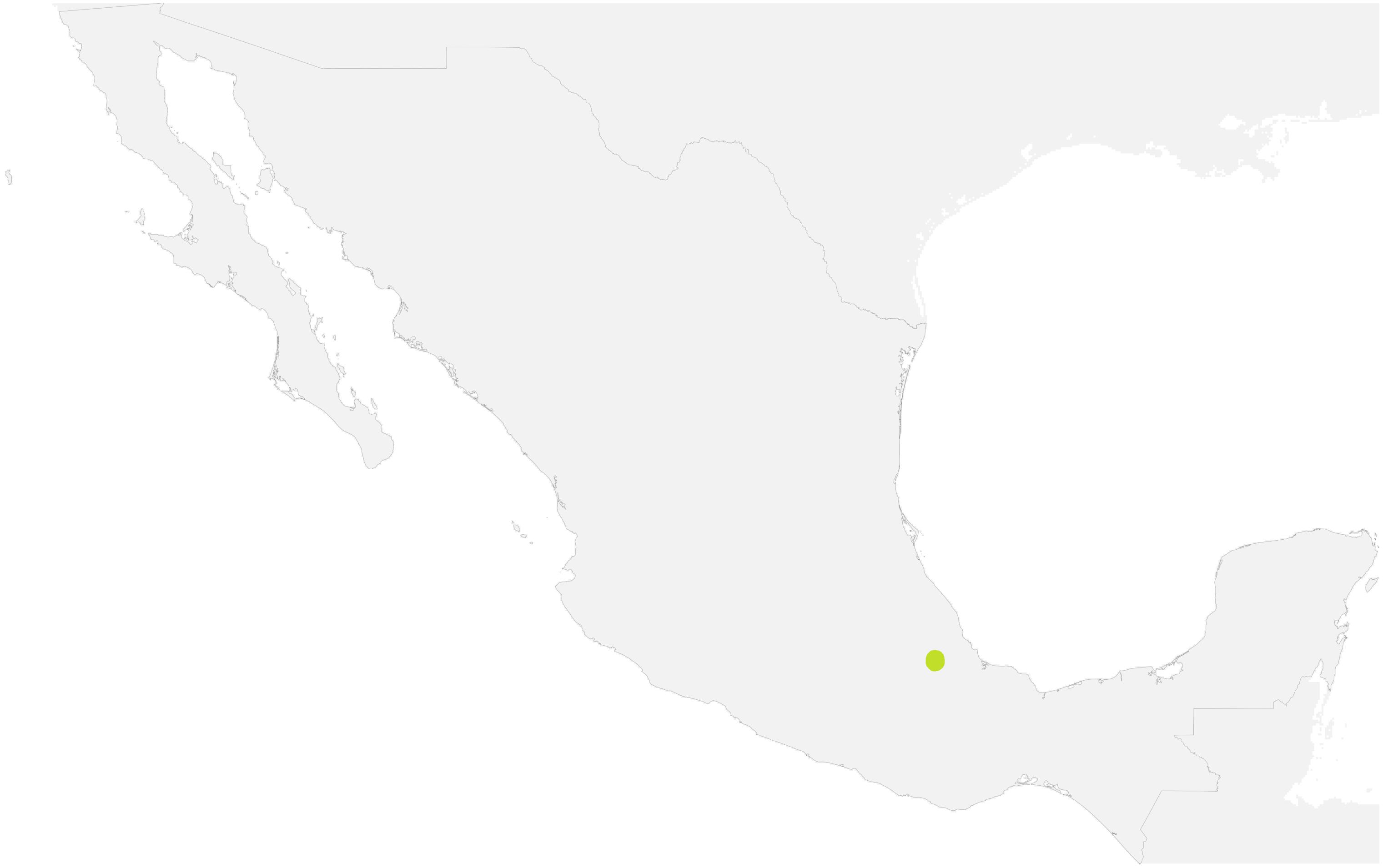

Current EOR

Cerrophidion tzotzilorum

A)

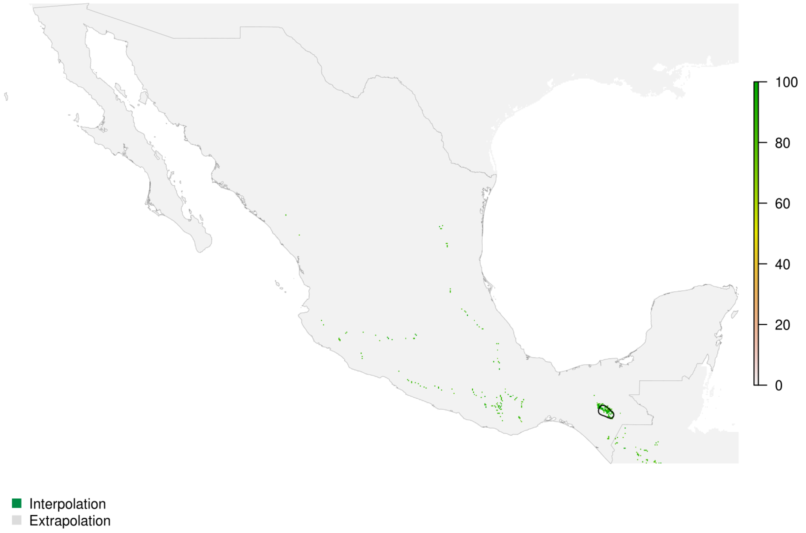

100 bootstrapped binary MES surfaces
(constructed using 90 occurrence records)

B)

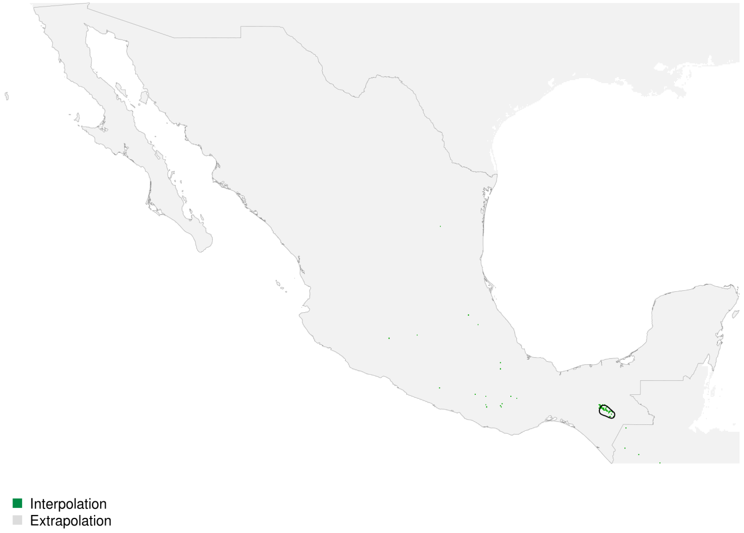

Binary bootstrapped MESS (95% threshold)

C)

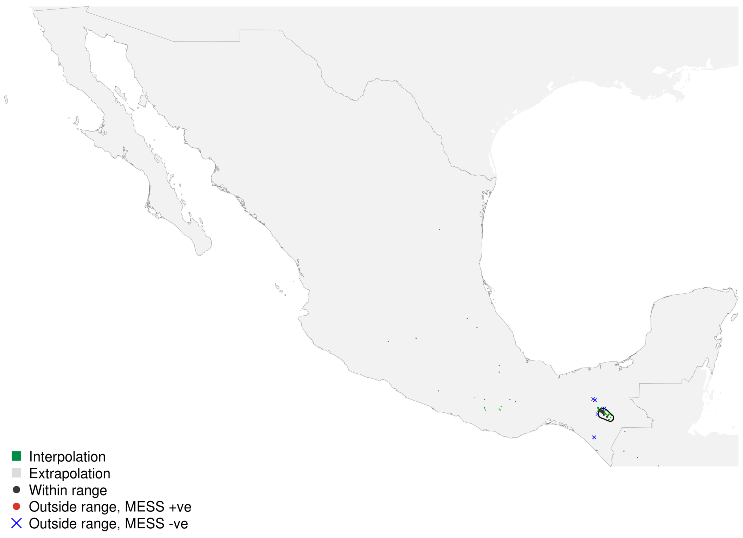

Binary bootstrapped MESS (95% threshold)
(with 11 outside of range occurrence records)

D)

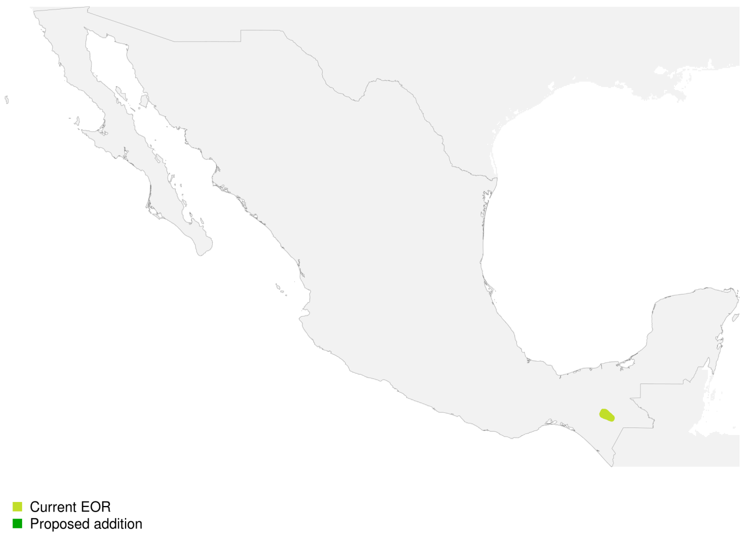

Suggested amended range
(incorporating 0 outside of range MESS +ve records)

Crotalus adamanteus

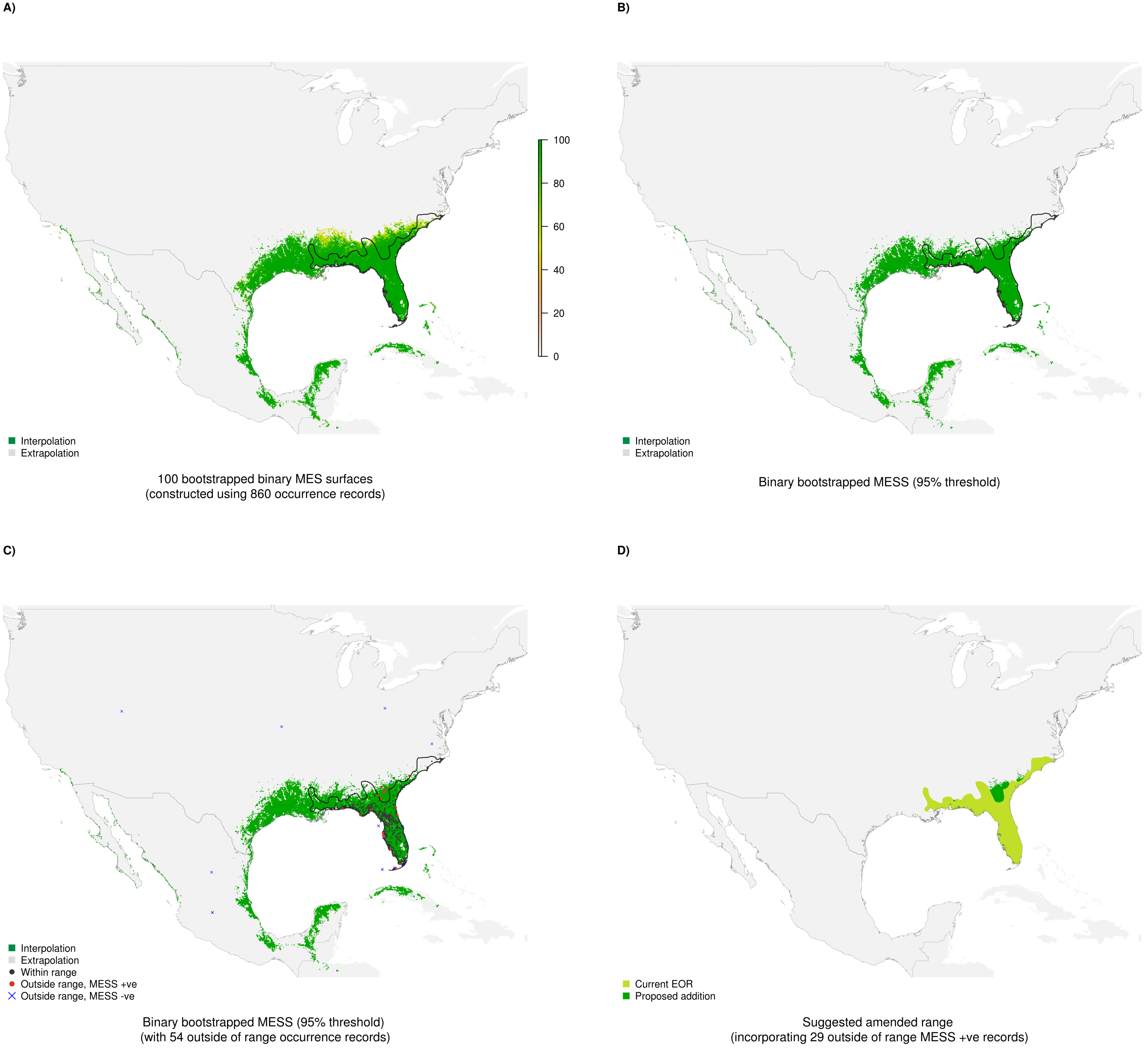

Crotalus atrox

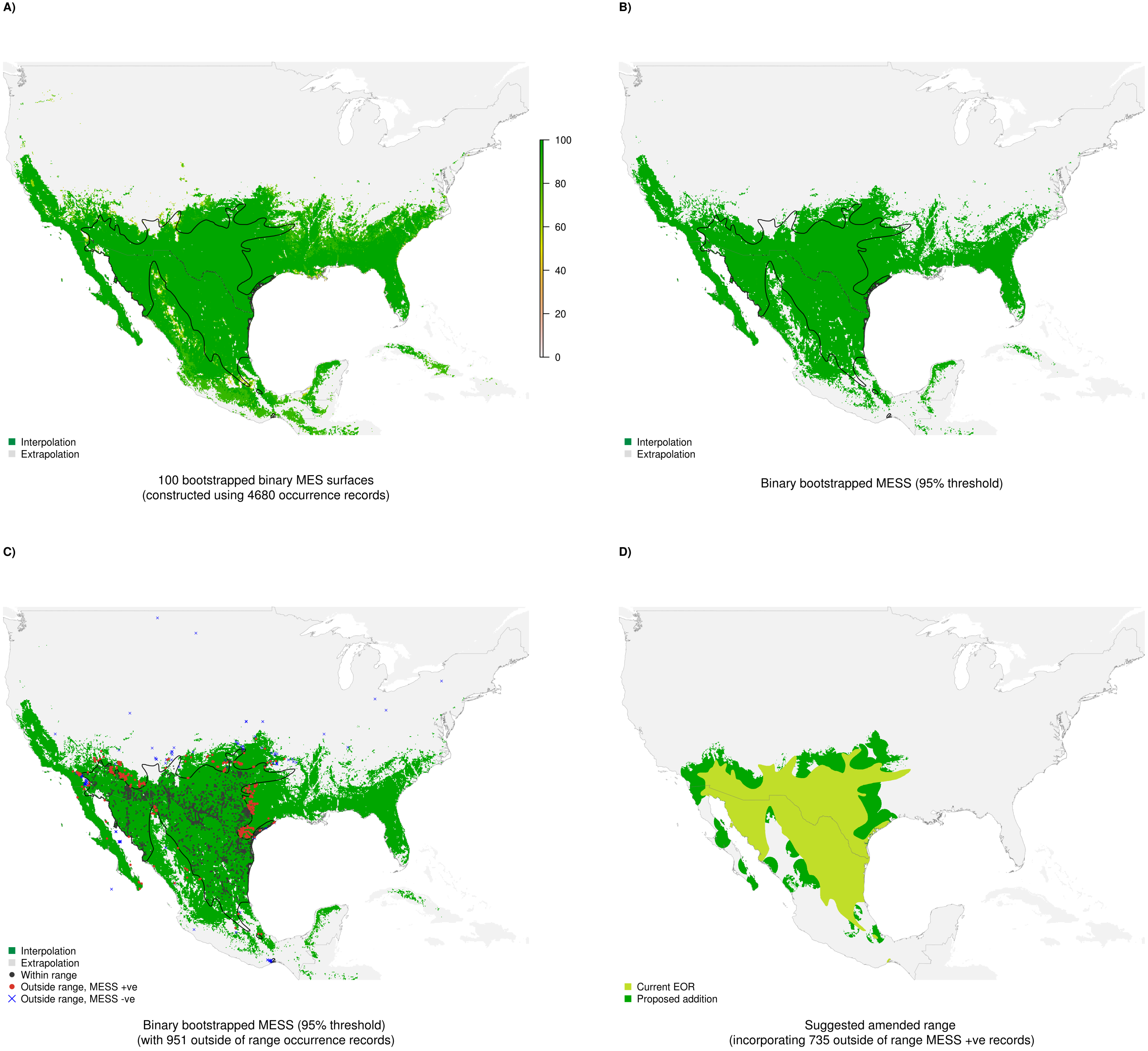

Crotalus basiliscus

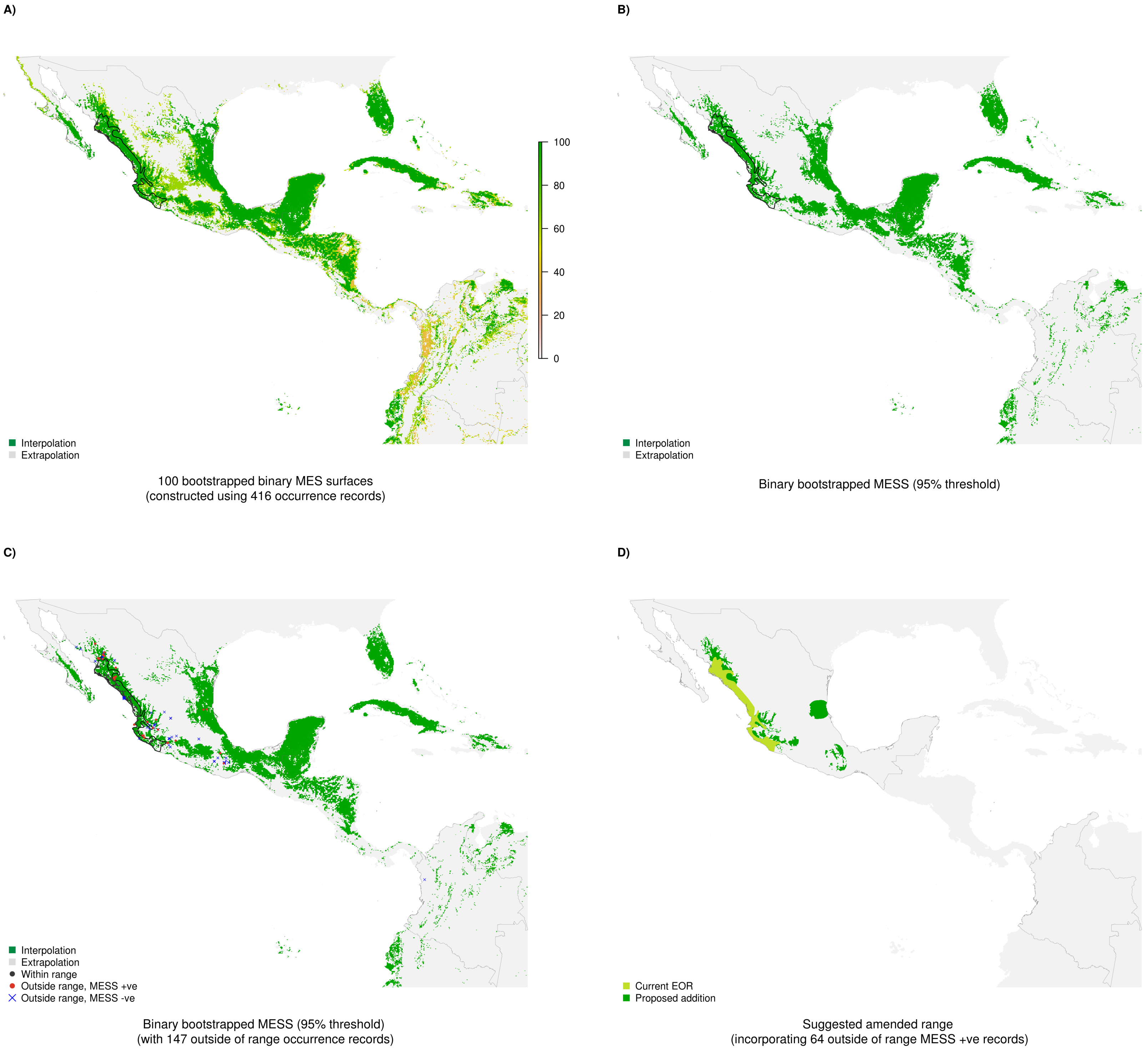

Crotalus durissus

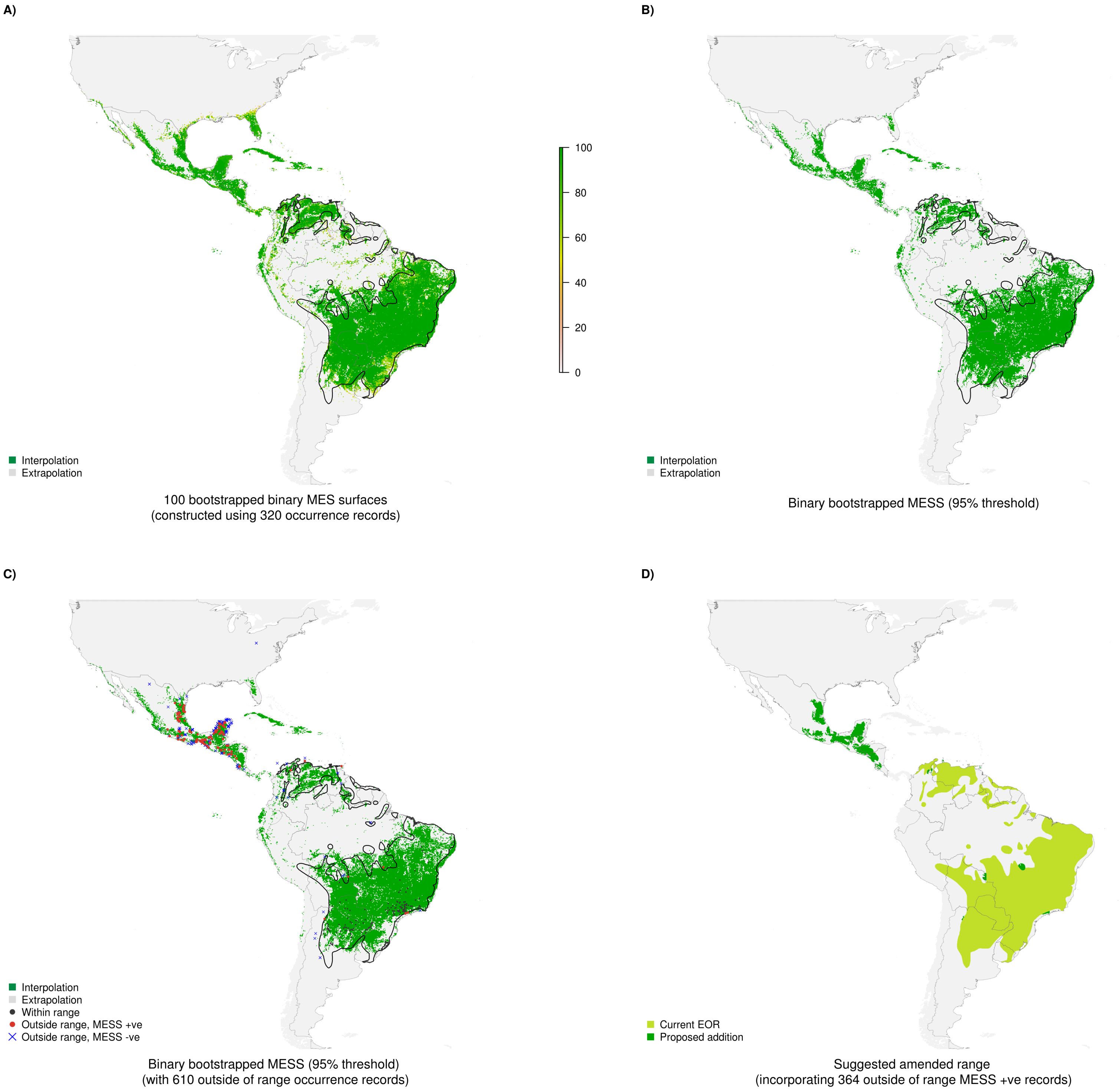

Crotalus horridus

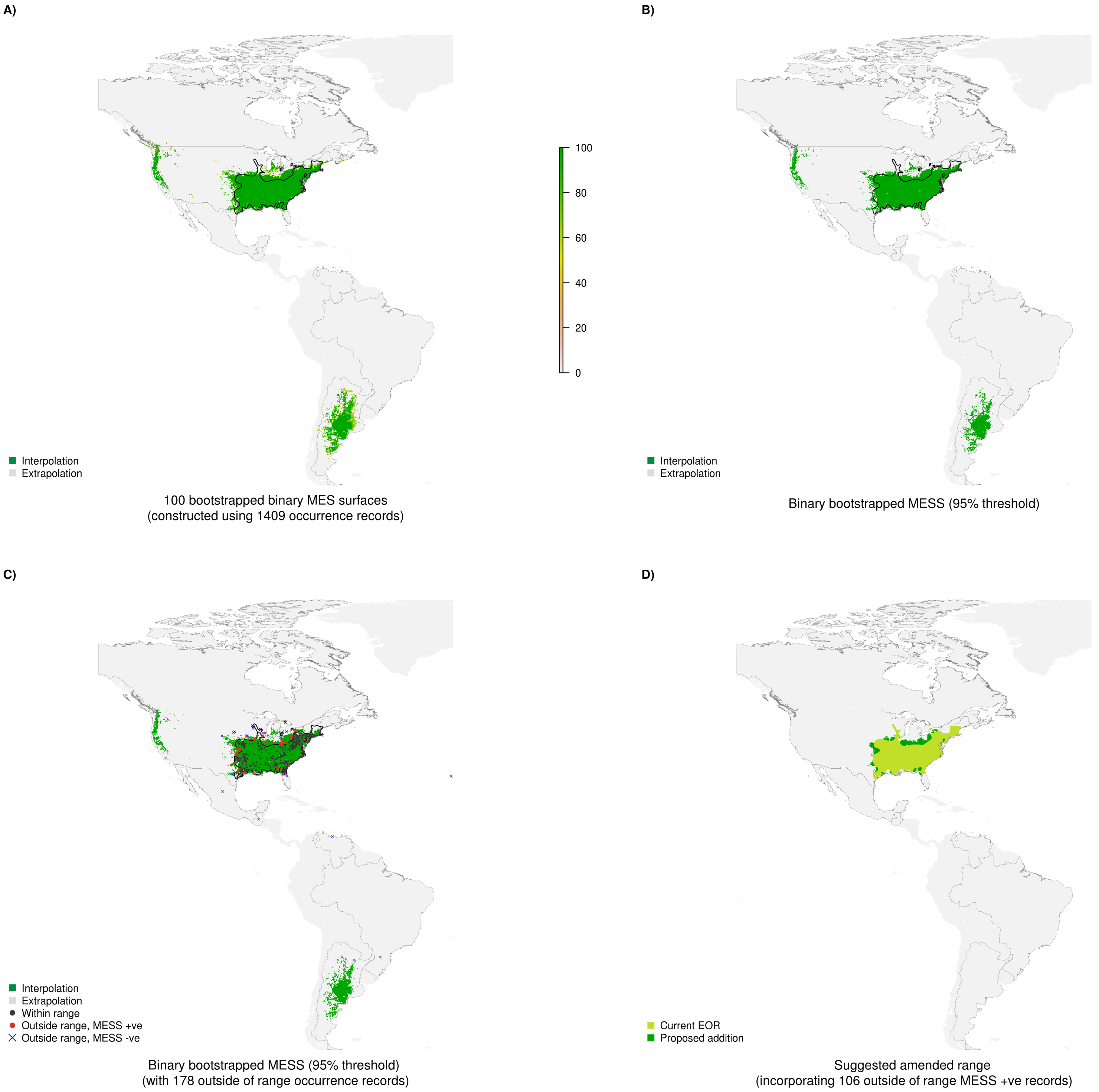

Crotalus molossus

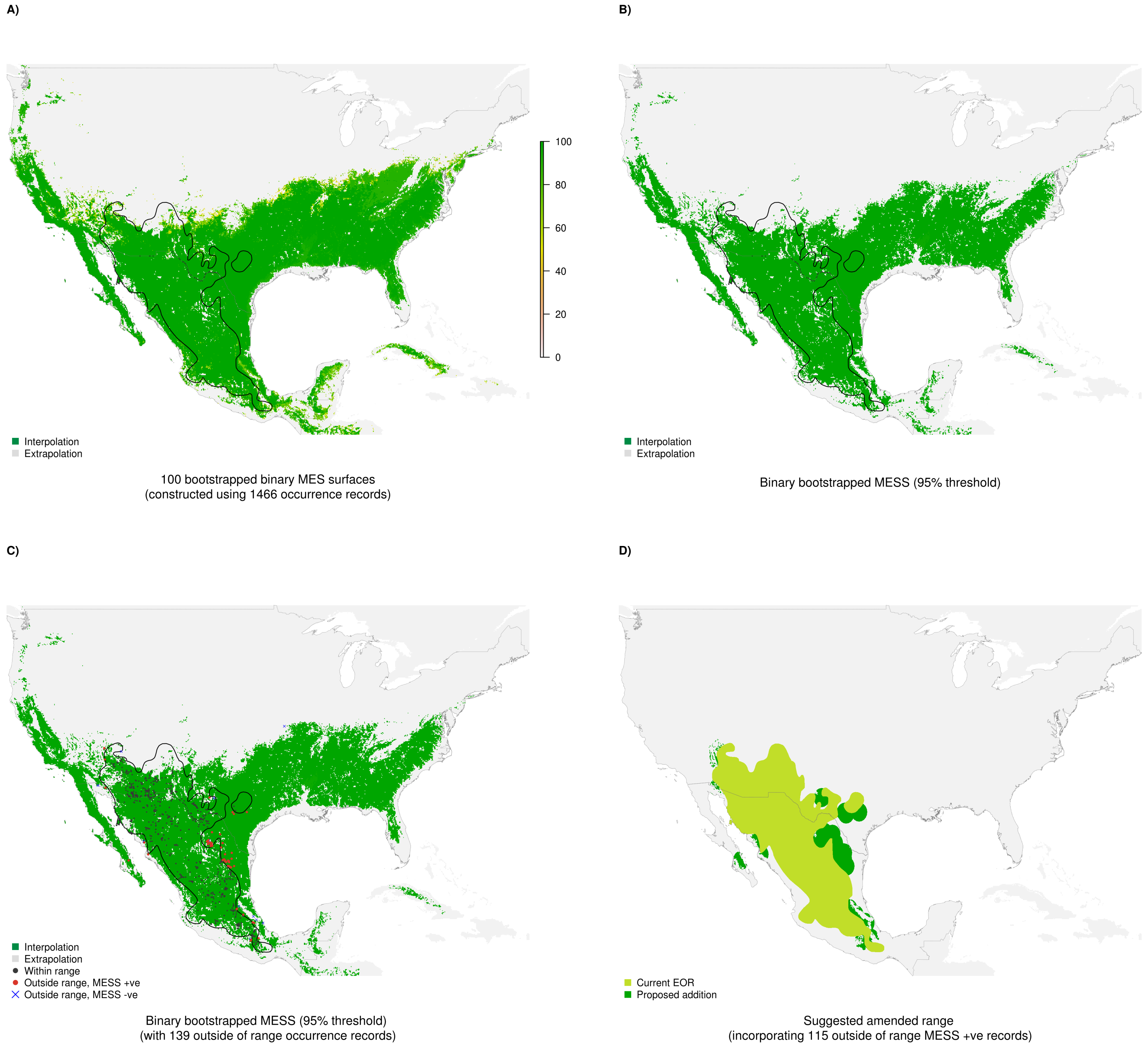

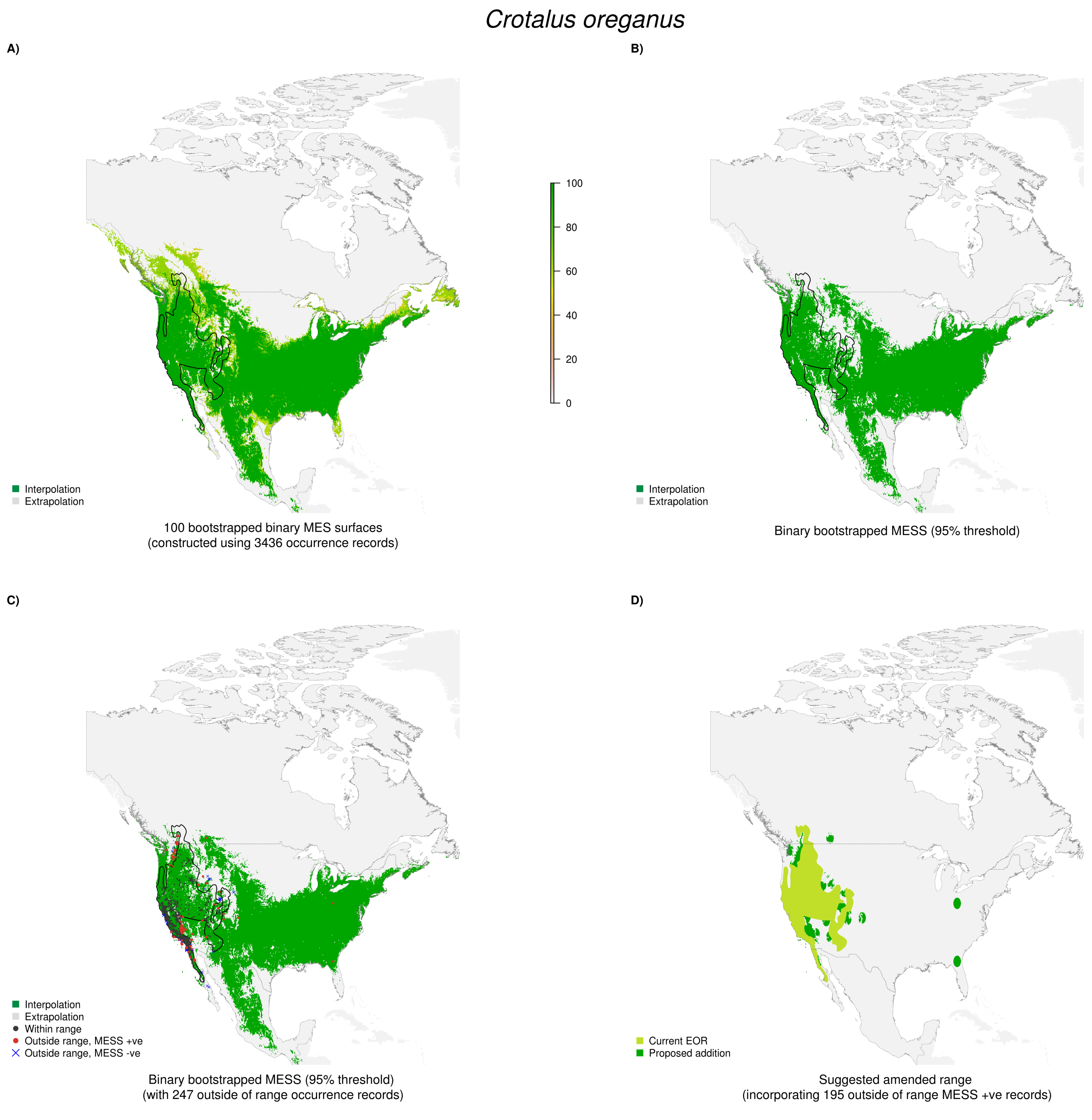

A) 100 bootstrapped binary MES surfaces (constructed using 3436 occurrence records)

B) Binary bootstrapped MESS (95% threshold)

C) Binary bootstrapped MESS (95% threshold) (with 247 outside of range occurrence records)

D) Suggested amended range (incorporating 195 outside of range MESS +ve records)

Crotalus ornatus

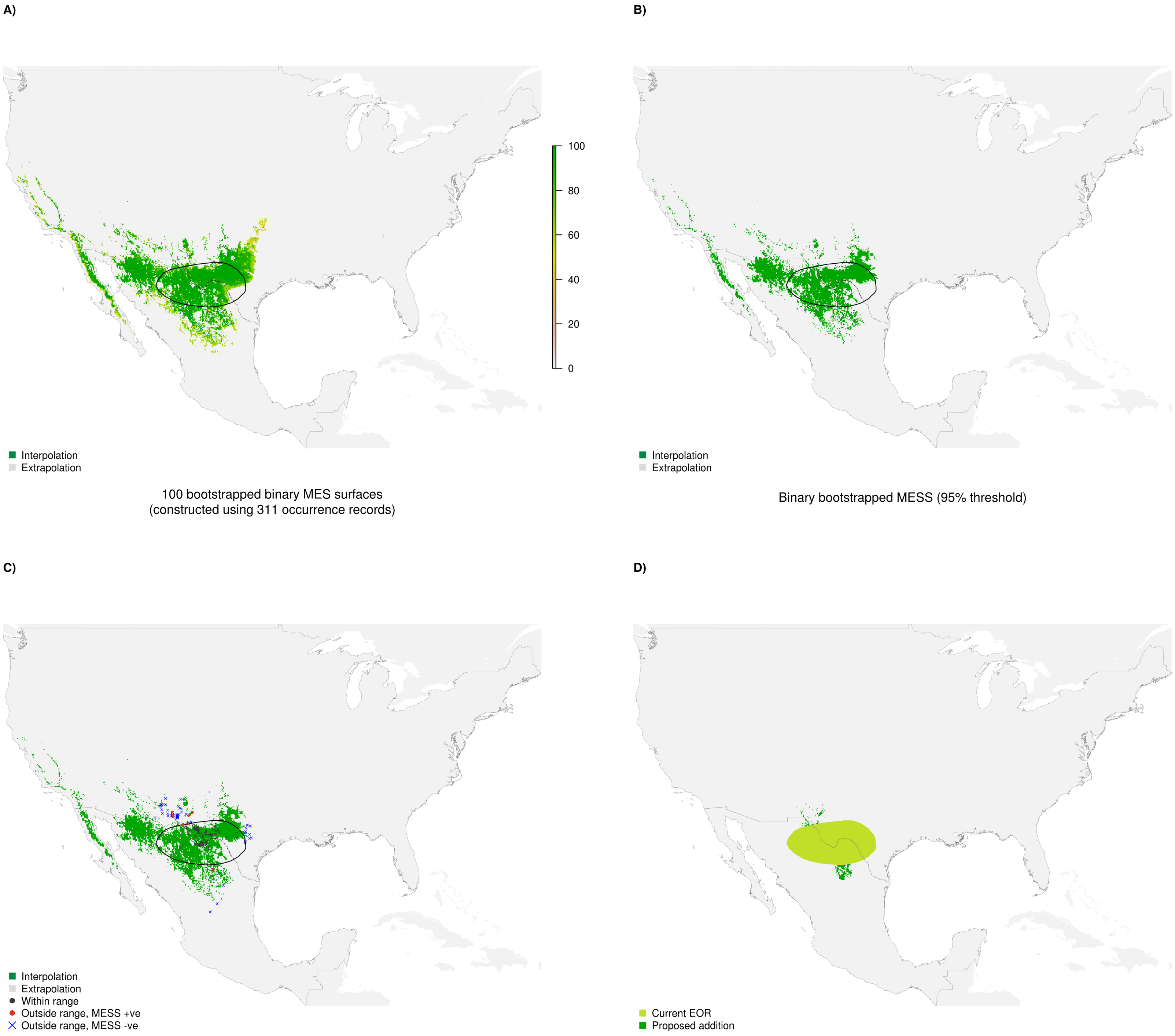

A) 100 bootstrapped binary MES surfaces
(constructed using 311 occurrence records)

B) Binary bootstrapped MESS (95% threshold)

C) Binary bootstrapped MESS (95% threshold)
(with 180 outside of range occurrence records)

D) Suggested amended range
(incorporating 59 outside of range MESS +ve records)

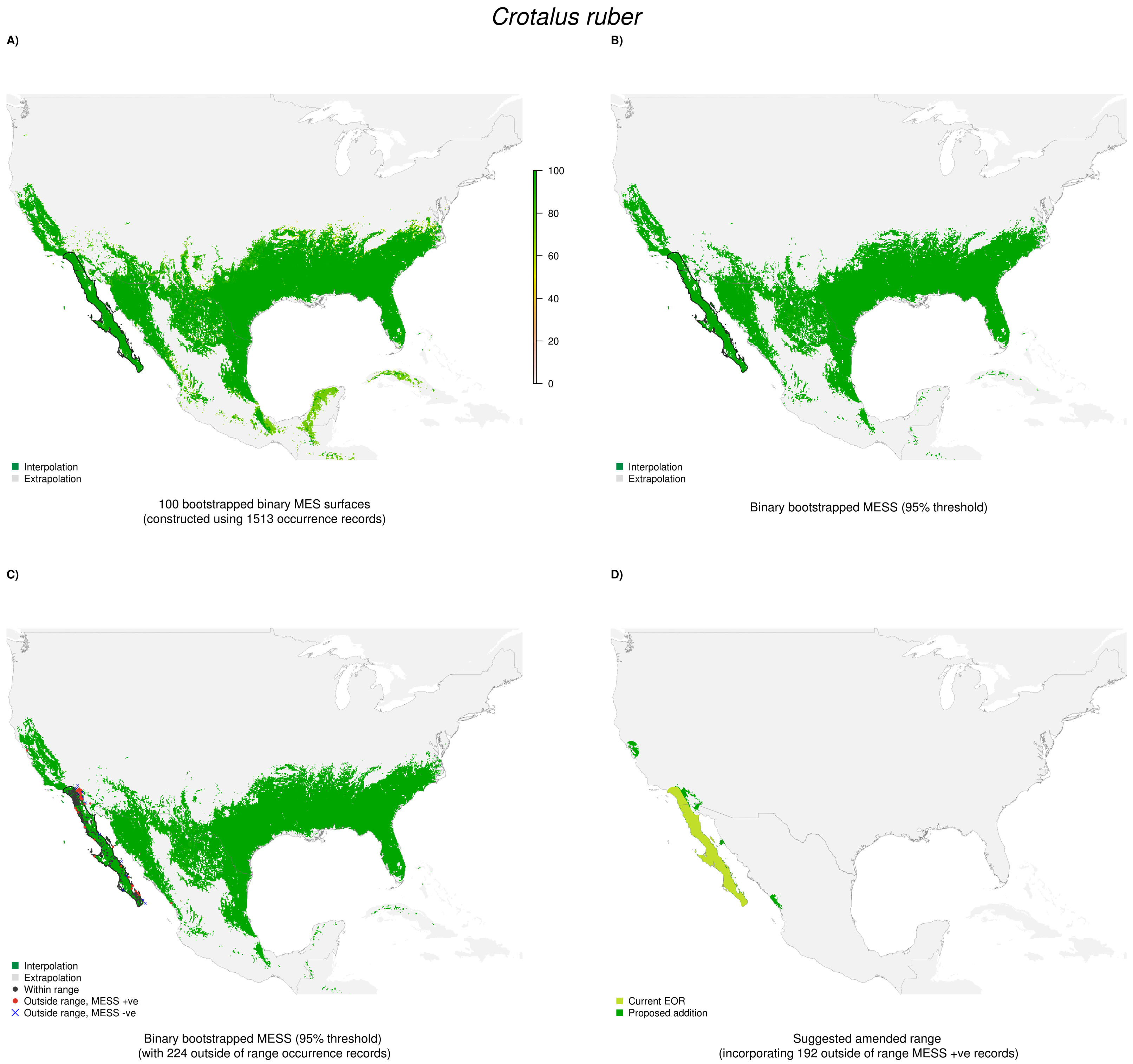
Crotalus ruber
A)
B)
100
80
60
40
20
0
Interpolation
Extrapolation
100 bootstrapped binary MES surfaces
(constructed using 1513 occurrence records)
Interpolation
Extrapolation
Binary bootstrapped MESS (95% threshold)
C)
D)
Interpolation
Extrapolation
Within range
Outside range, MESS +ve
Outside range, MESS -ve
Binary bootstrapped MESS (95% threshold)
(with 224 outside of range occurrence records)
Current EOR
Proposed addition
Suggested amended range
(incorporating 192 outside of range MESS +ve records)

Crotalus scutulatus

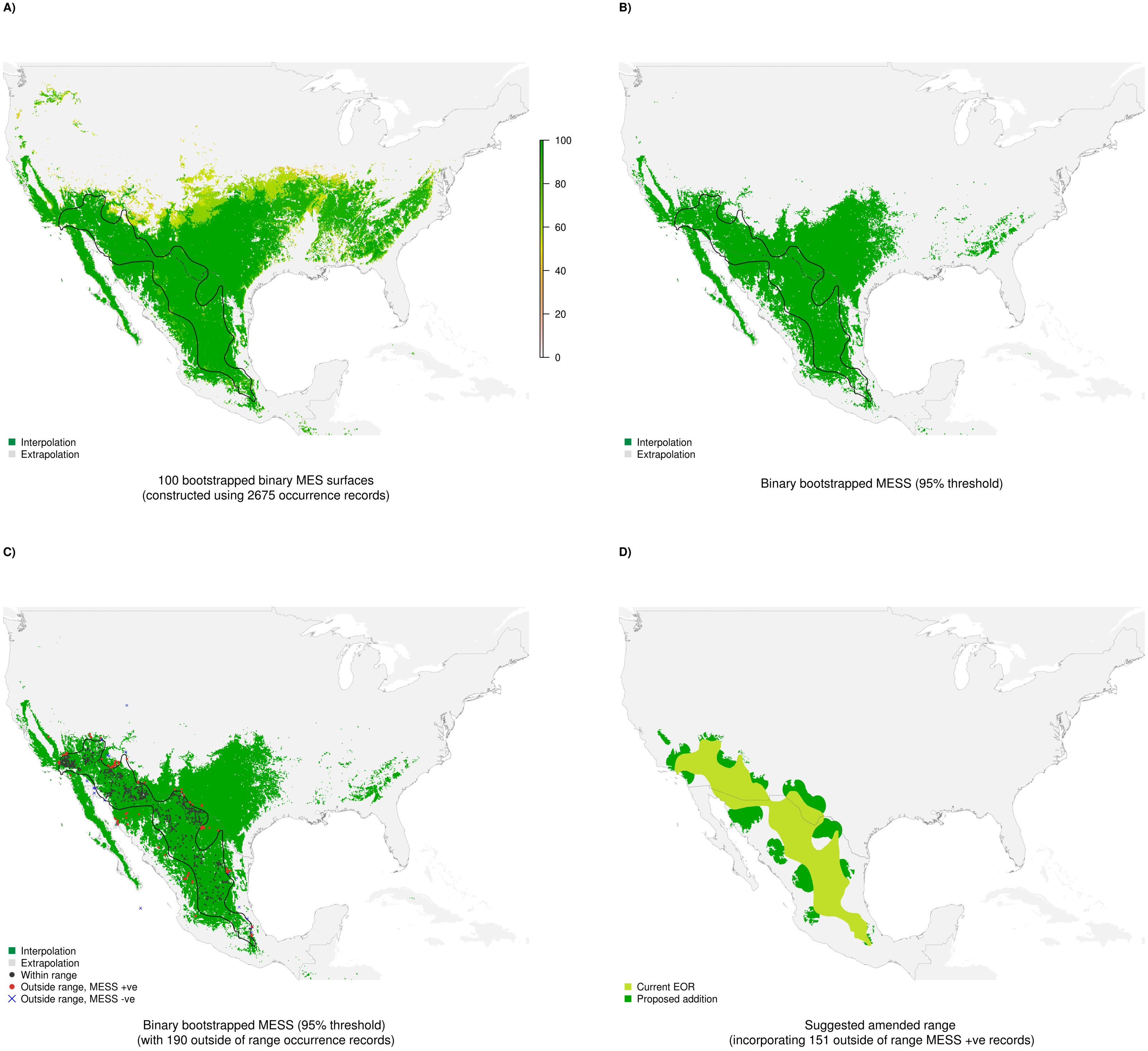

Crotalus simus

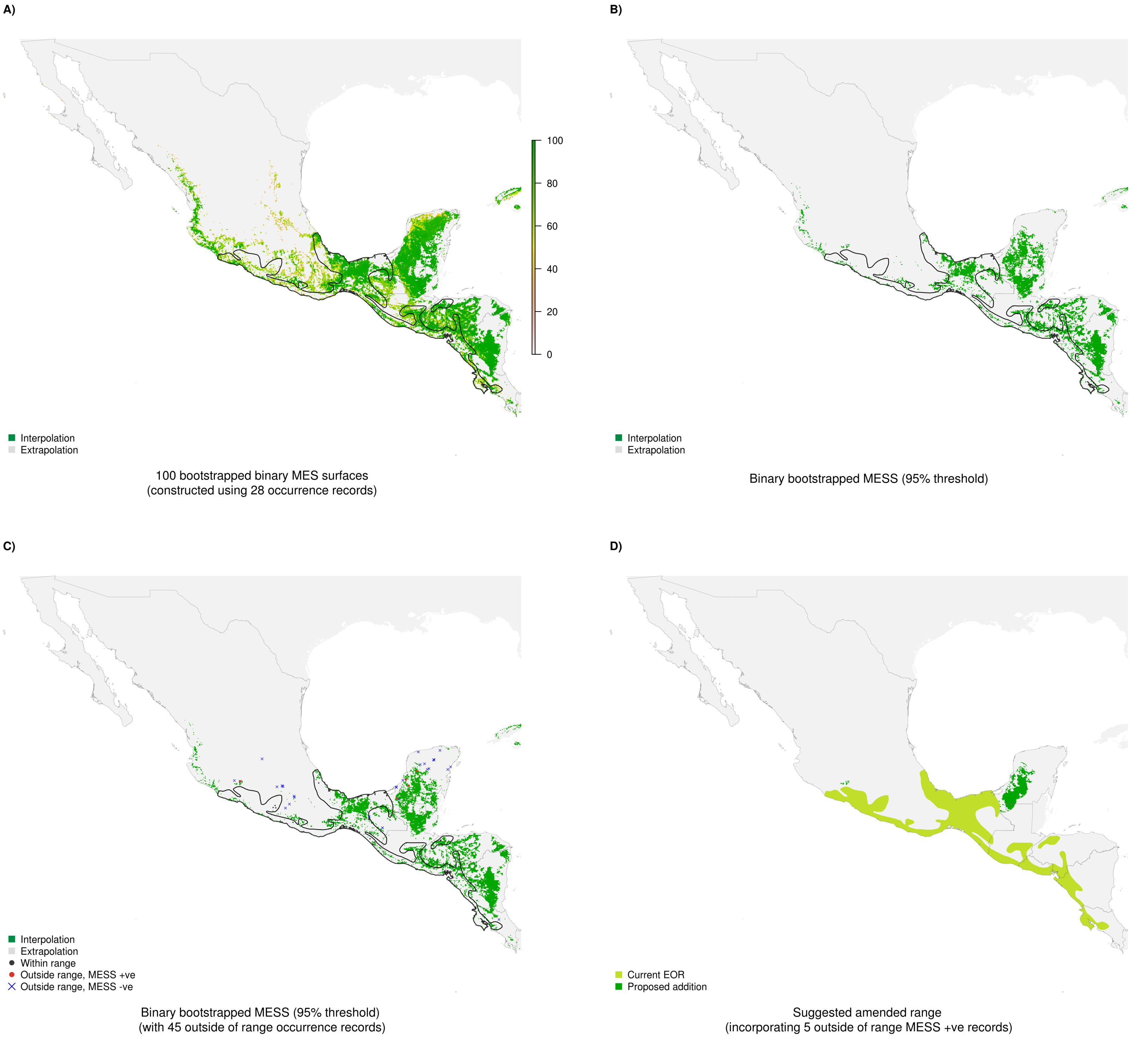

Crotalus totonacus

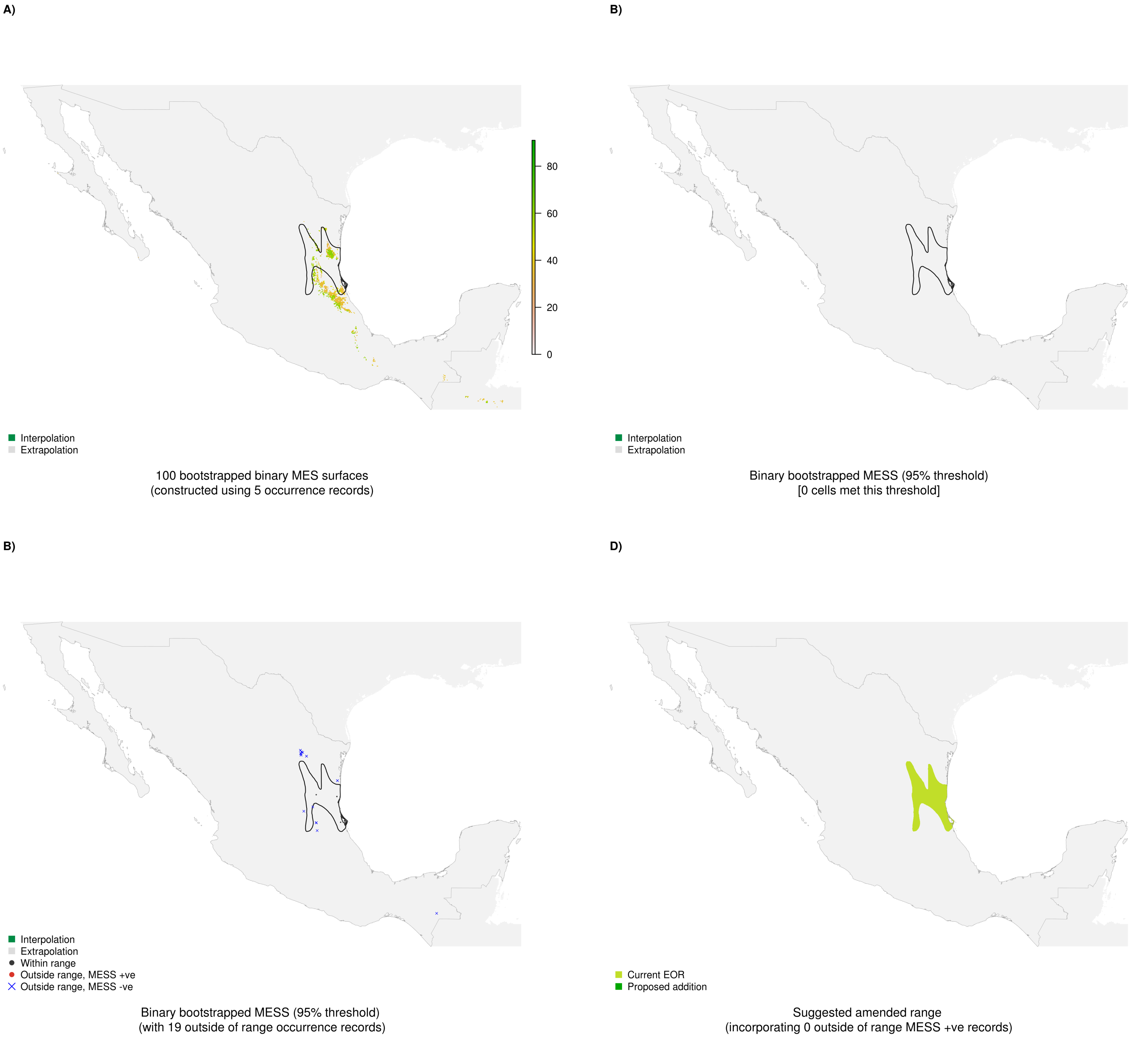

Crotalus tzabcan

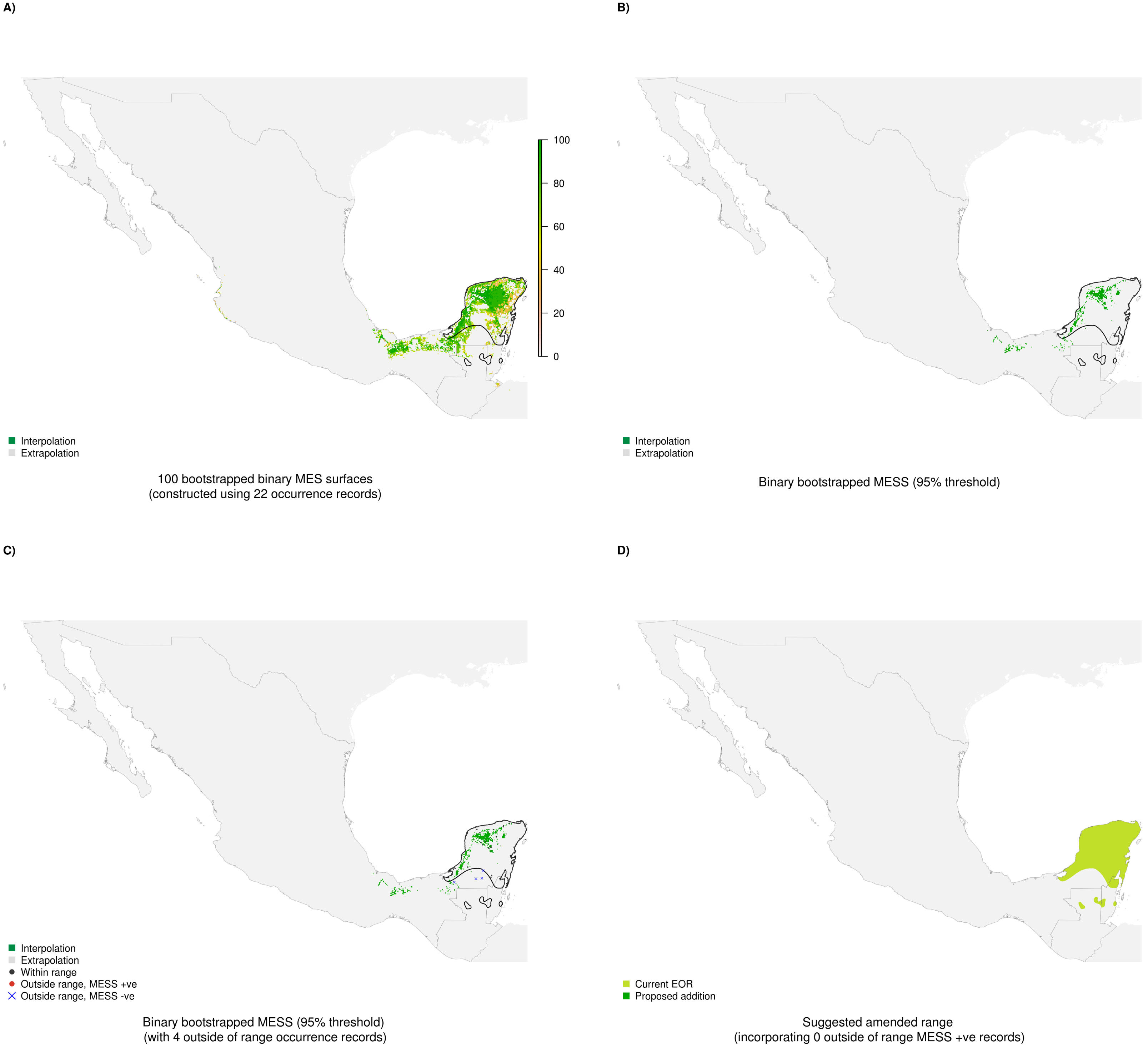

Crotalus viridis

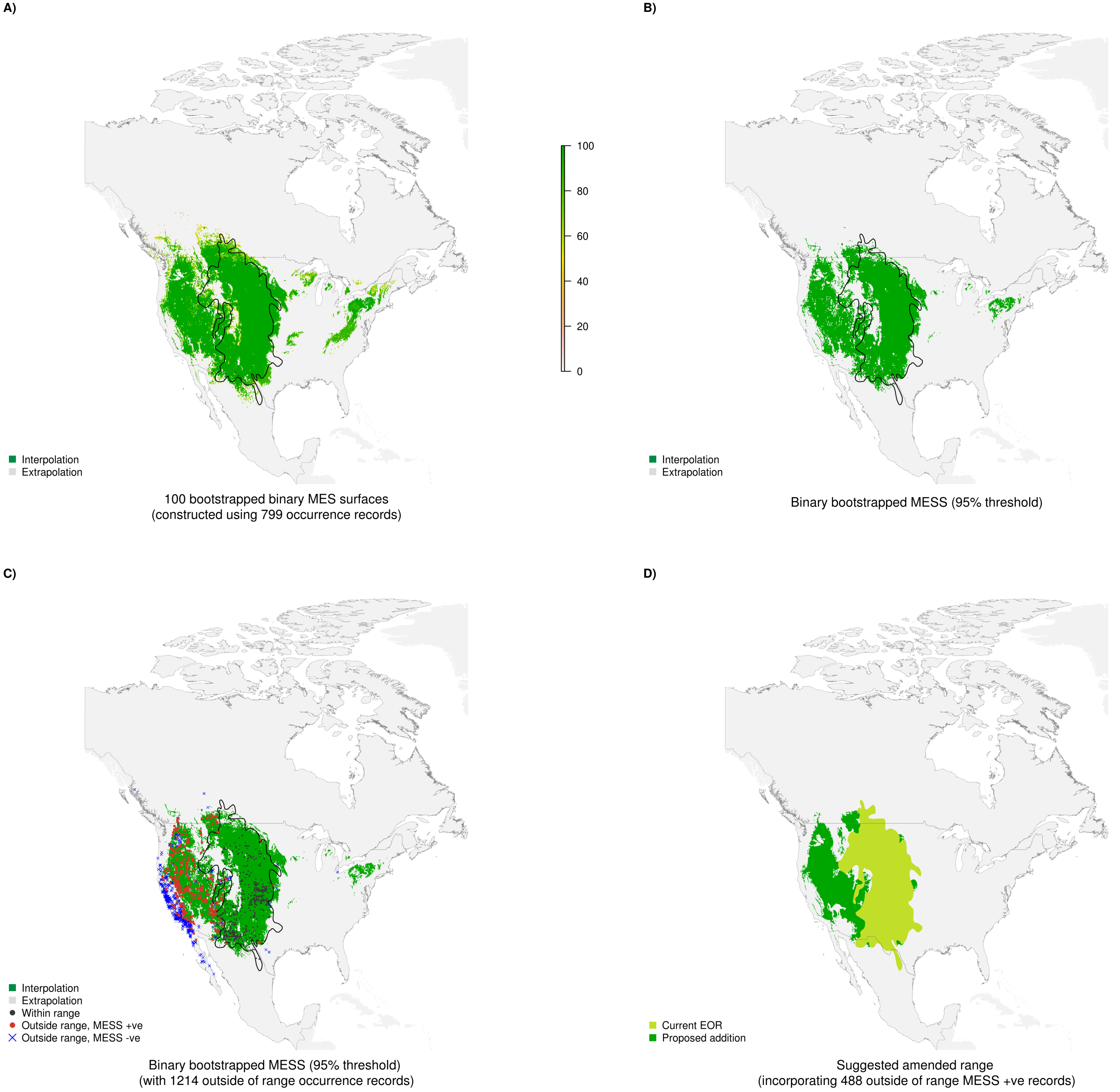

Cryptelytrops albolabris

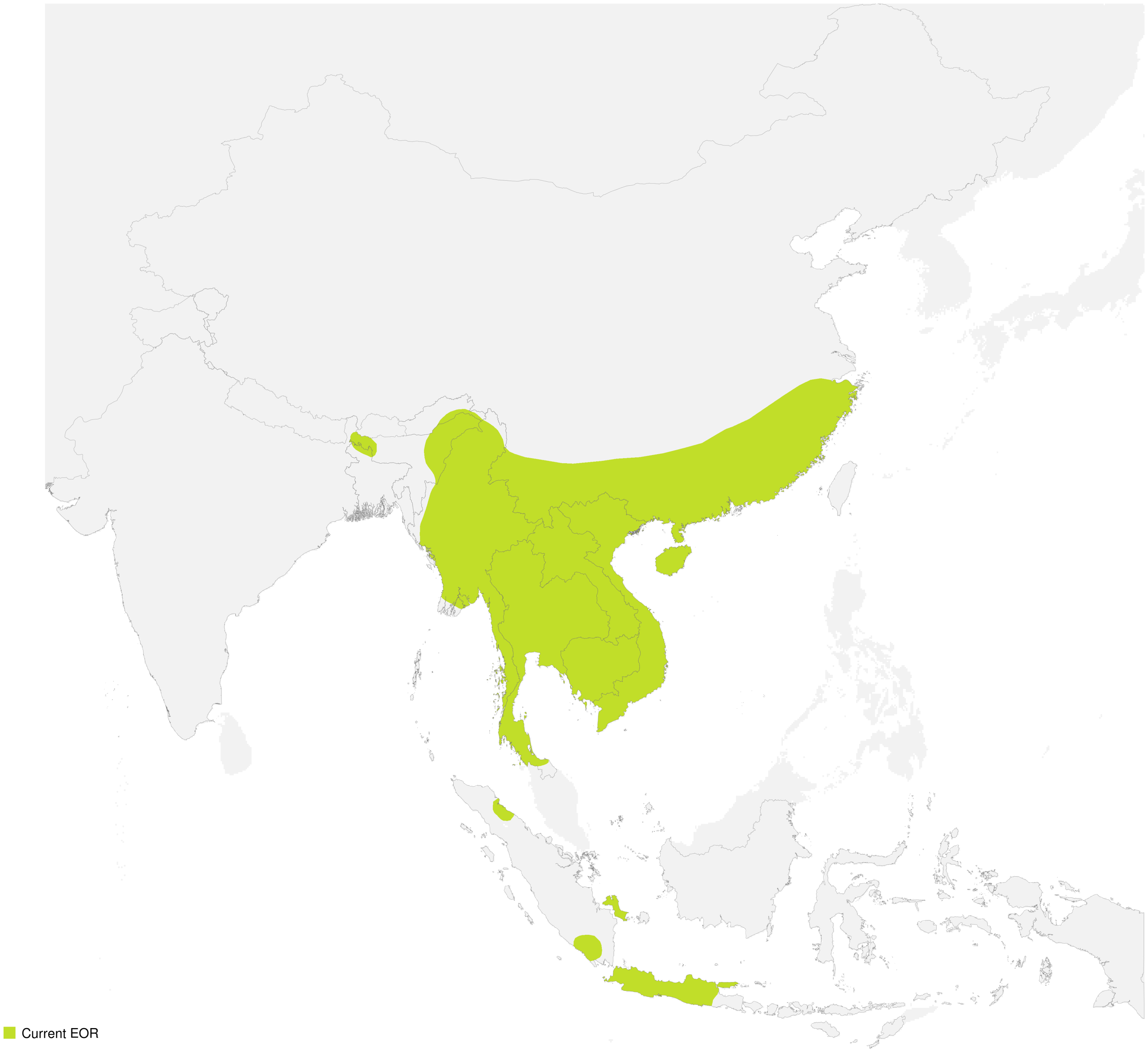

Cryptelytrops erythrurus

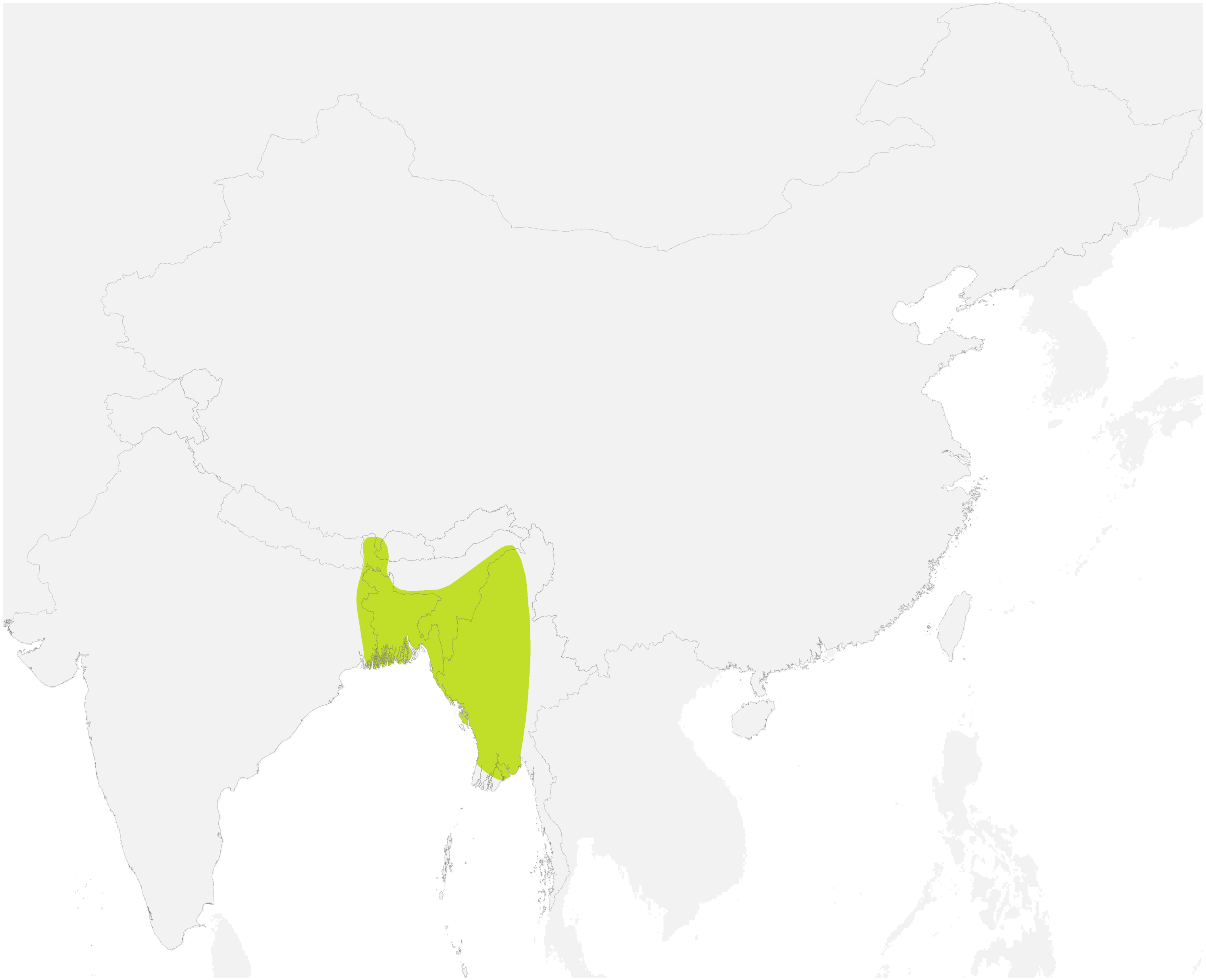

Current EOR

Cryptelytrops insularis

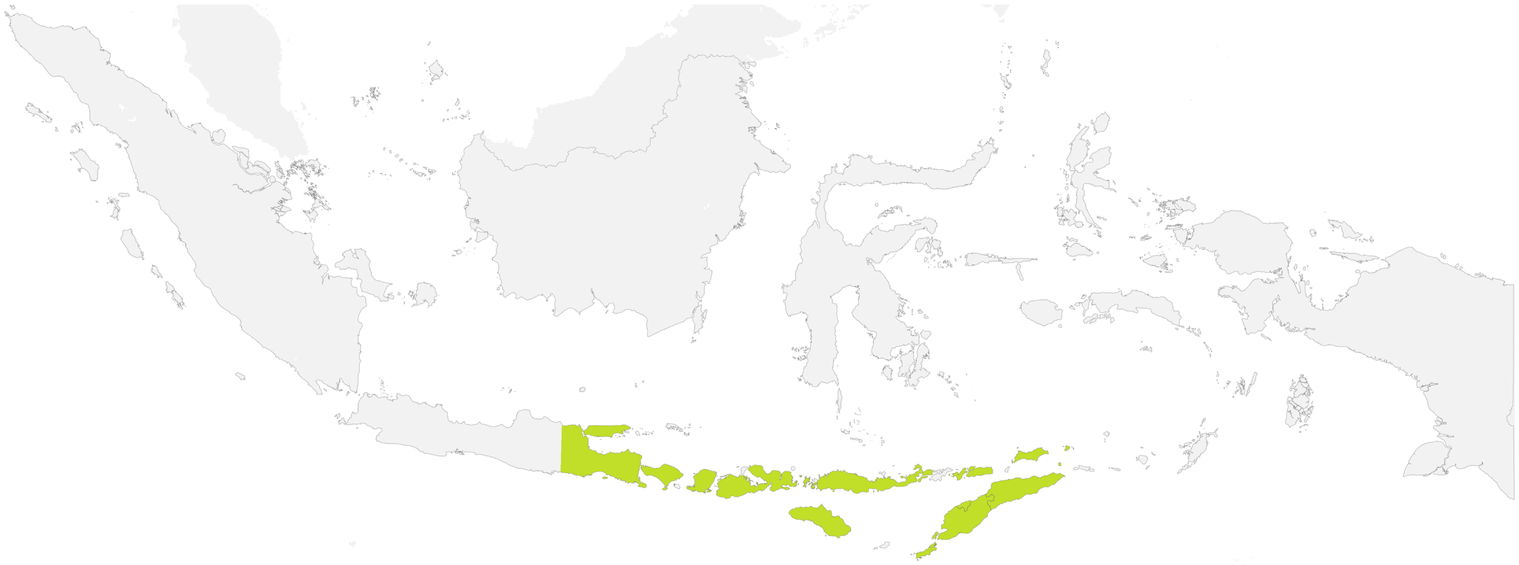

Current EOR

Cryptelytrops macrops

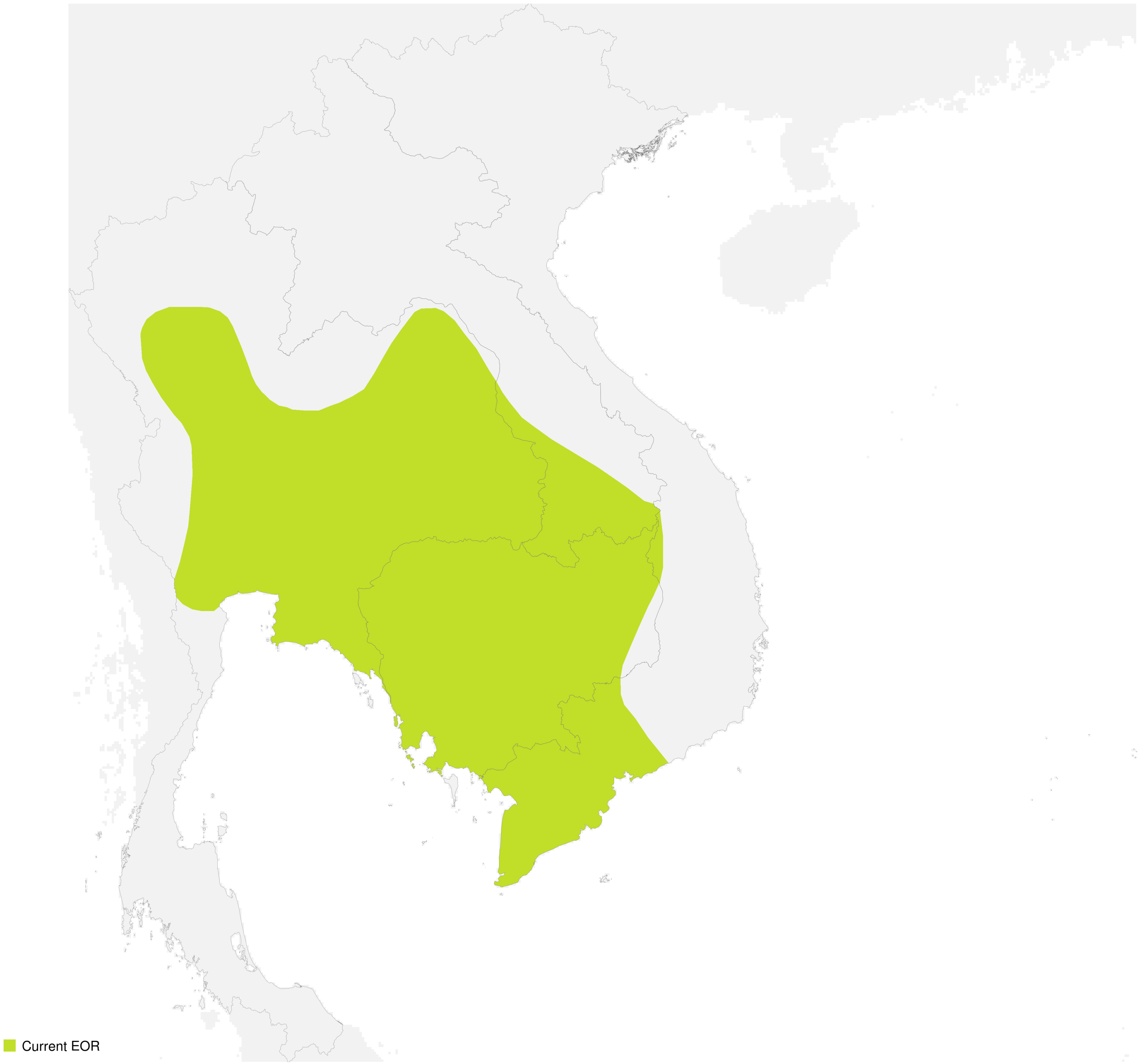

Cryptelytrops purpureomaculatus

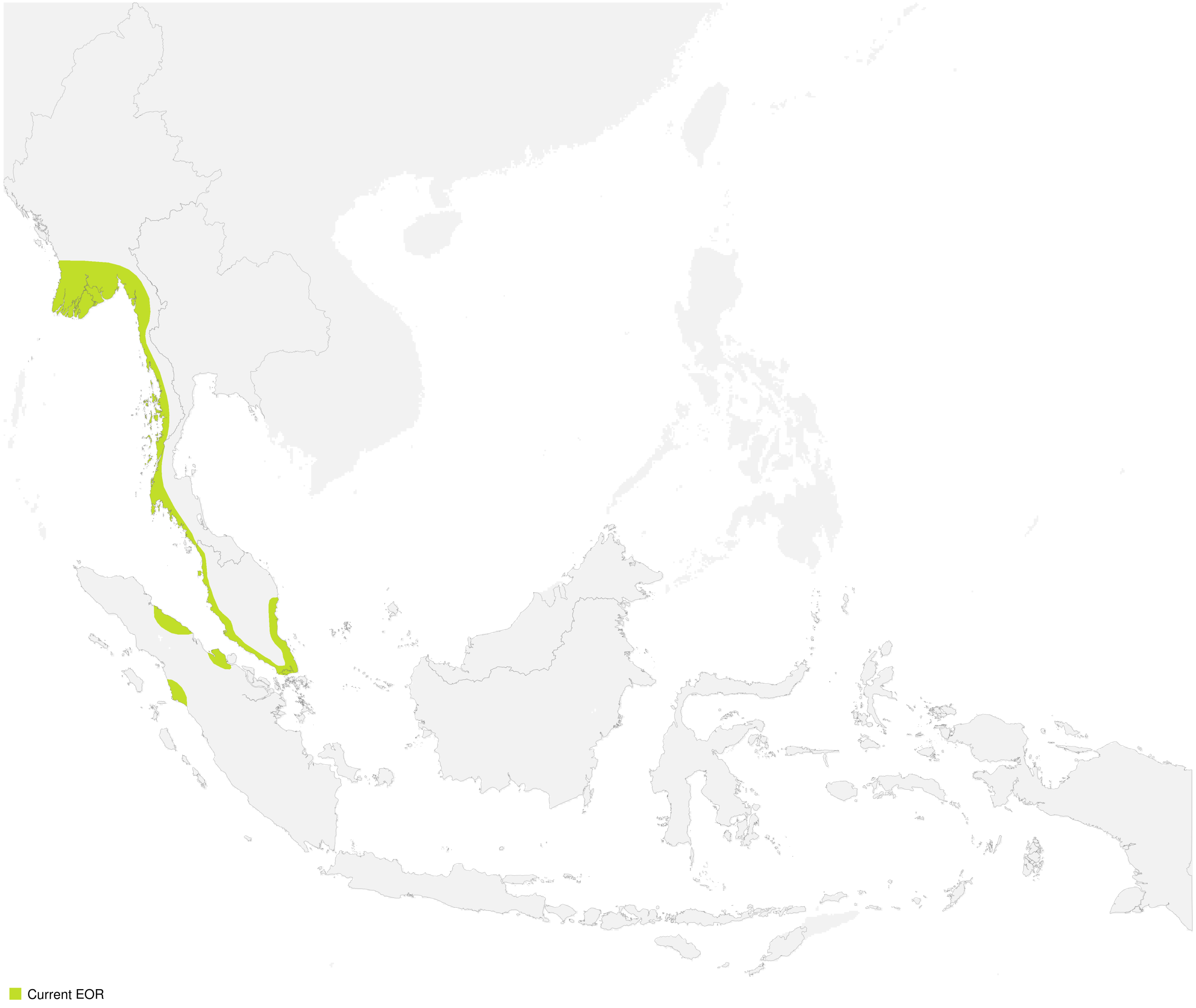

Cryptelytrops septentrionalis

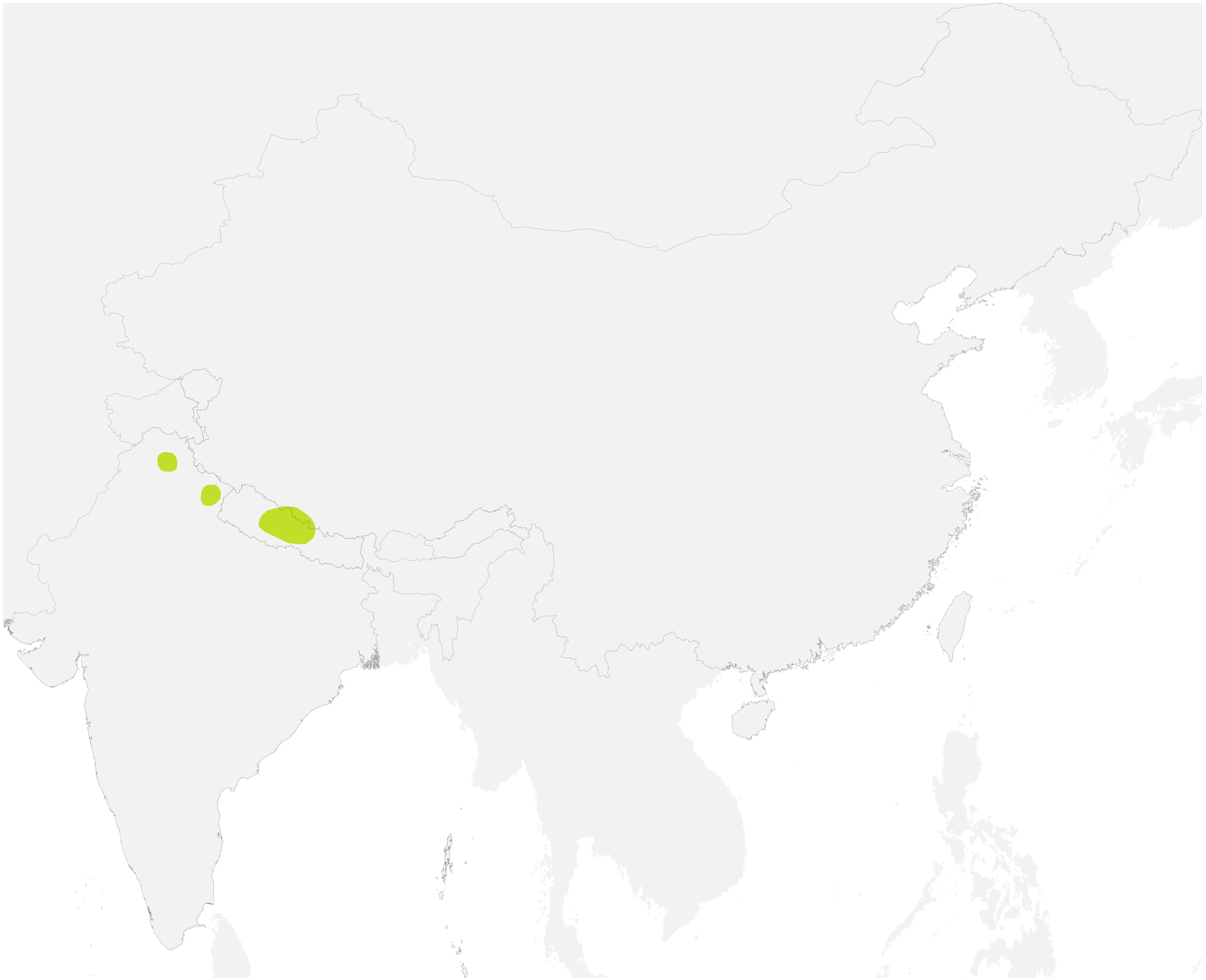

Current EOR

Daboia deserti

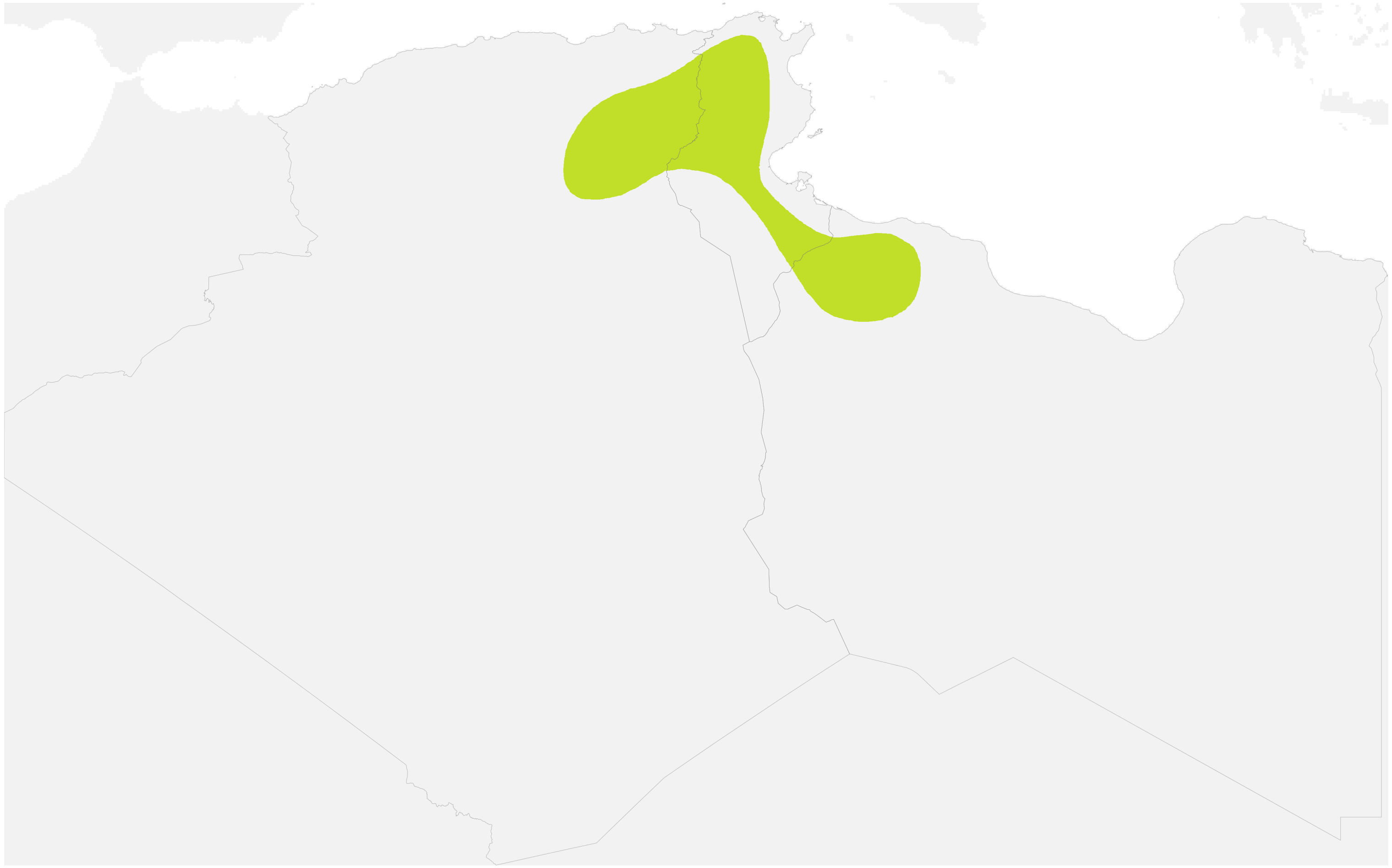

Current EOR

Daboia mauritanica

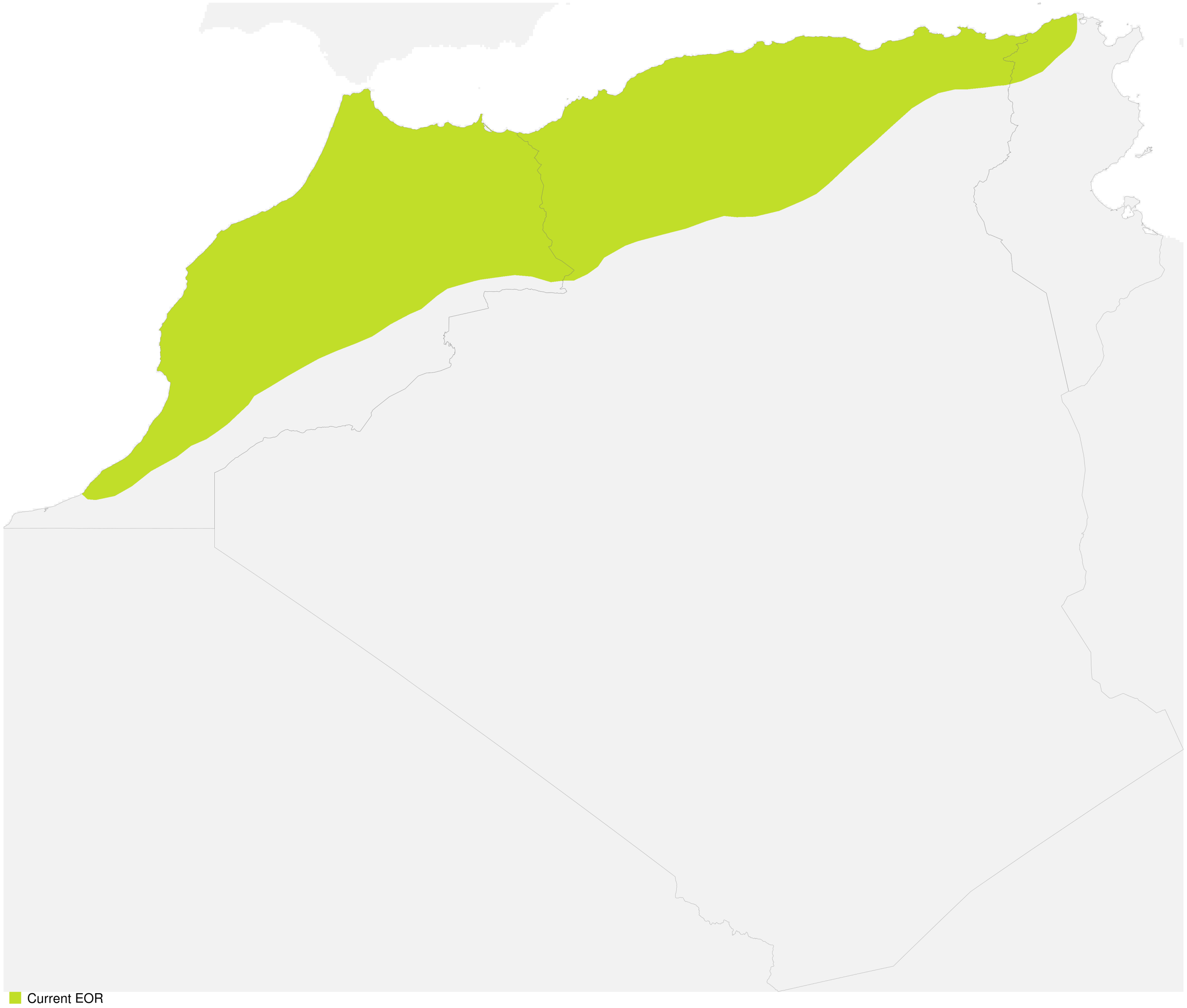

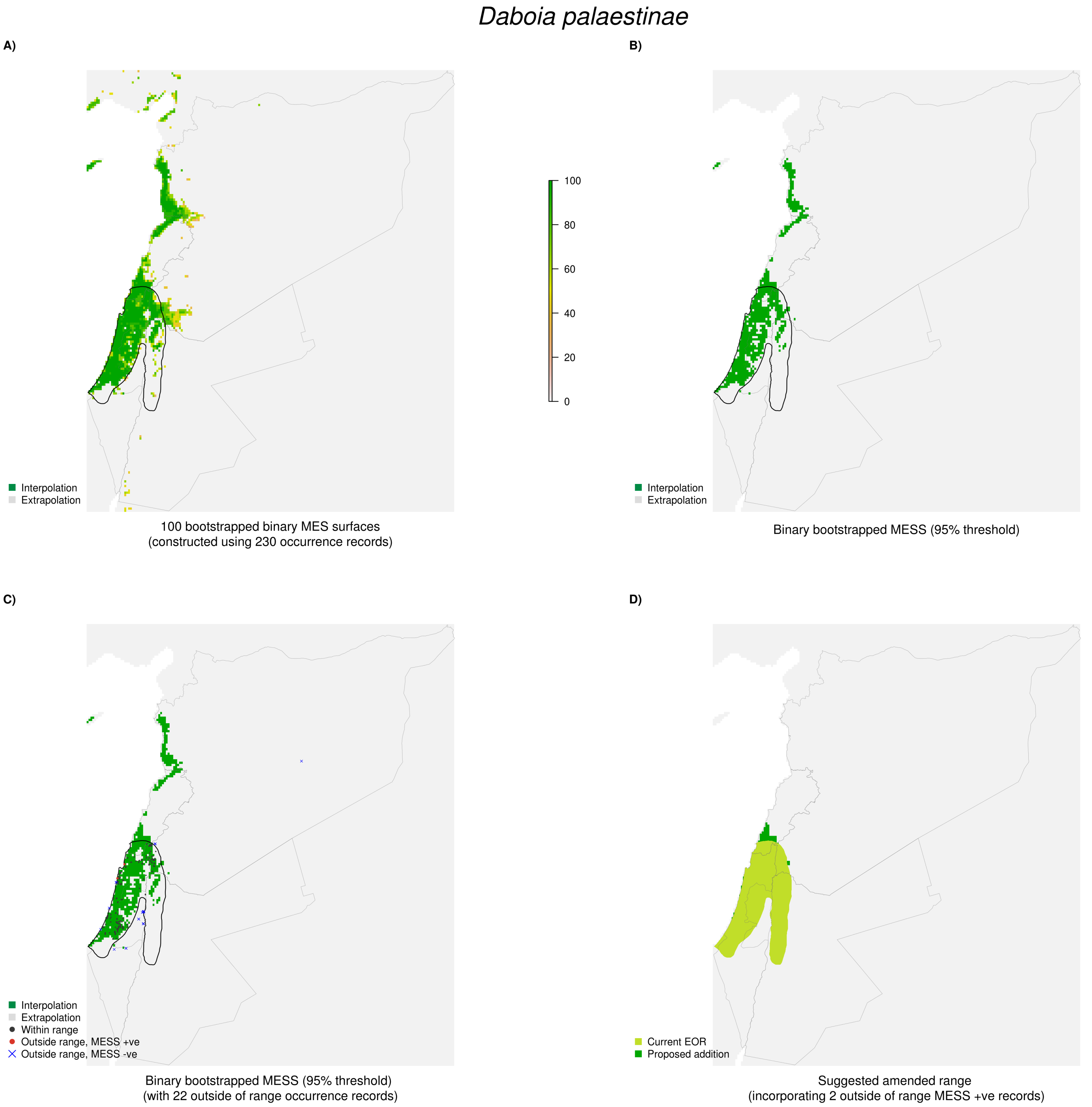

Daboia palaestinae
A)
B)
100
80
60
40
20
0
Interpolation
Extrapolation
100 bootstrapped binary MES surfaces
(constructed using 230 occurrence records)
Interpolation
Extrapolation
Binary bootstrapped MESS (95% threshold)
C)
D)
Interpolation
Extrapolation
Within range
Outside range, MESS +ve
Outside range, MESS -ve
Binary bootstrapped MESS (95% threshold)
(with 22 outside of range occurrence records)
Current EOR
Proposed addition
Suggested amended range
(incorporating 2 outside of range MESS +ve records)

Daboia russelii

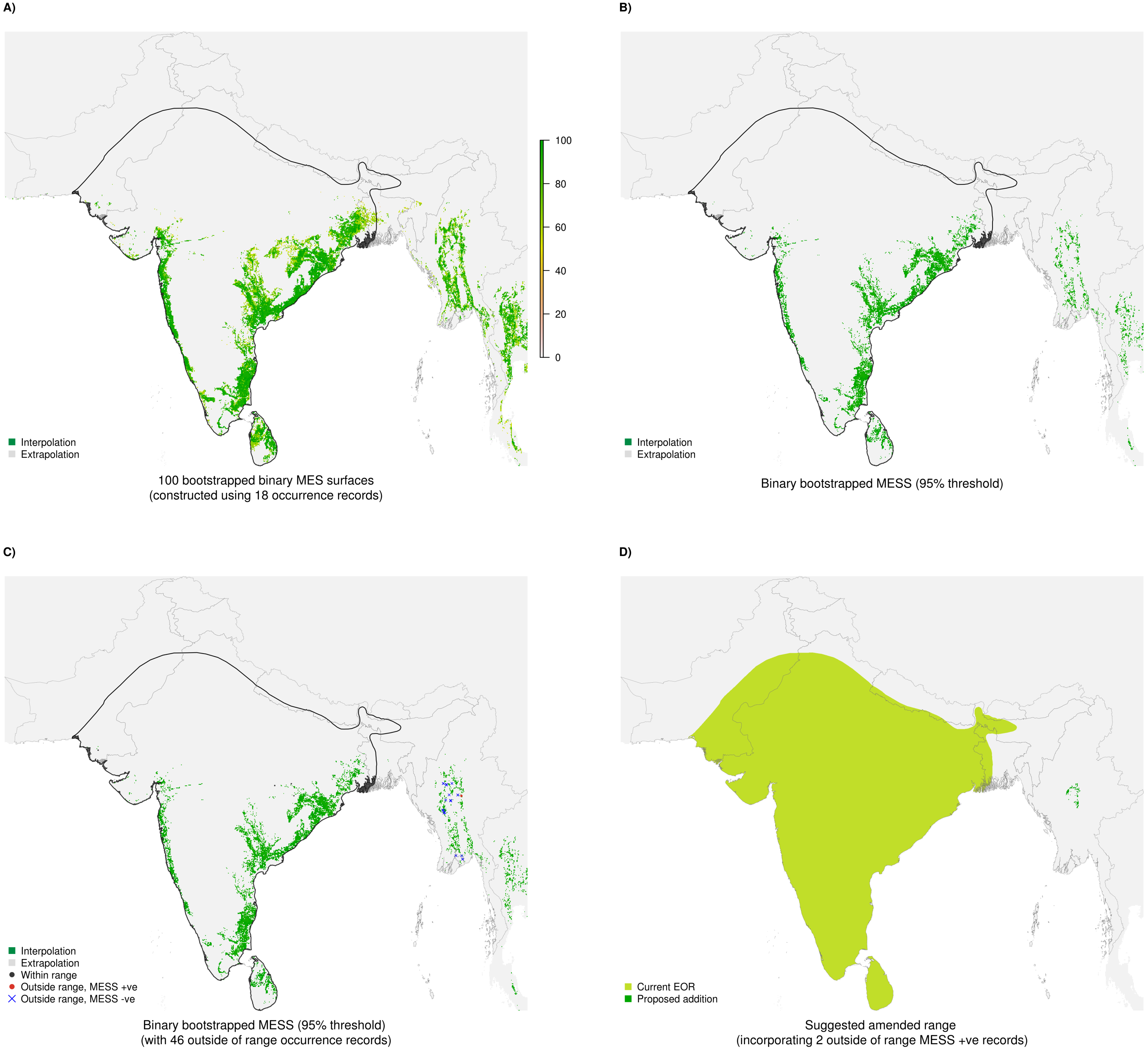

Daboia siamensis

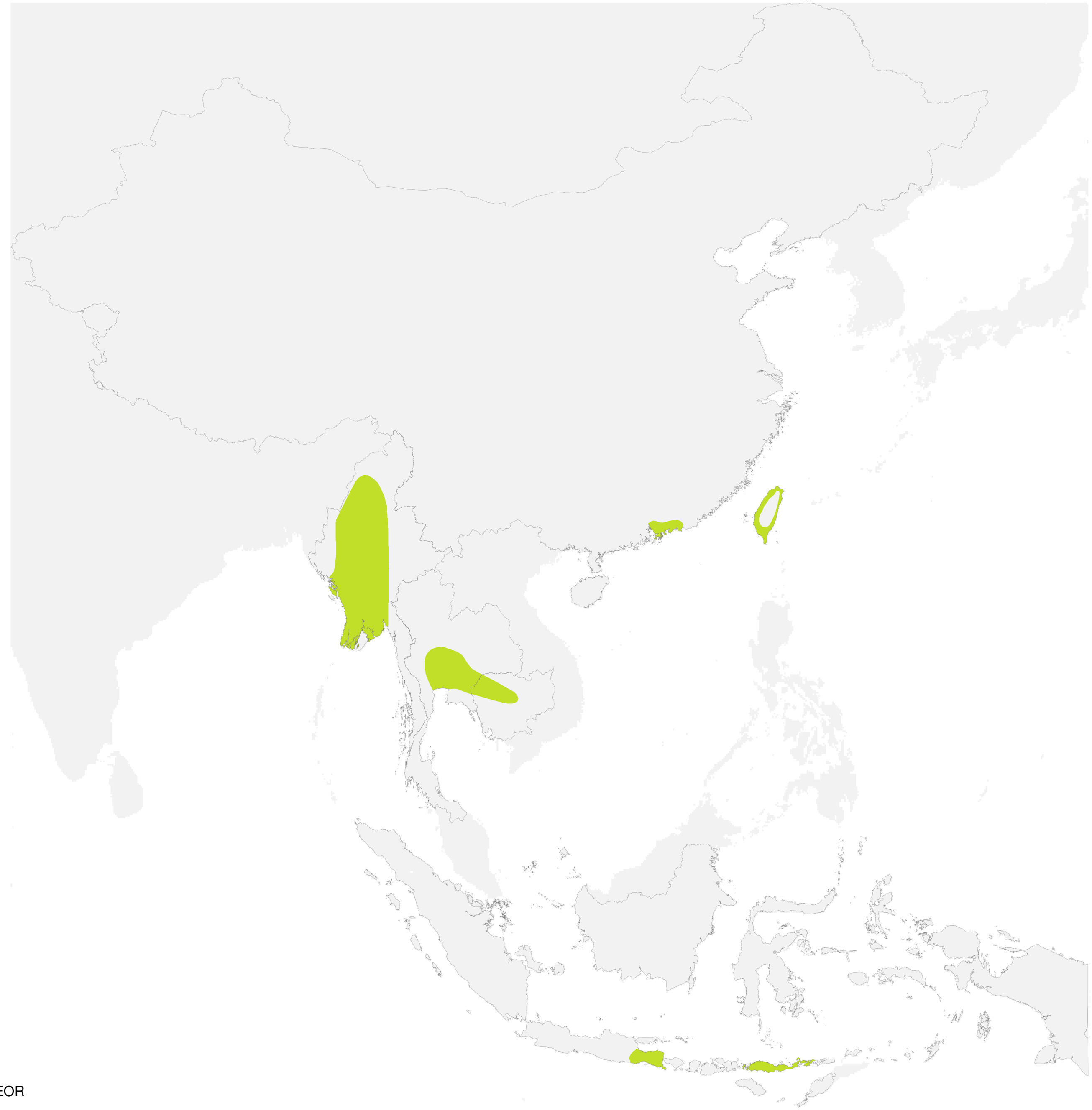

Current EOR

Deinagkistrodon acutus

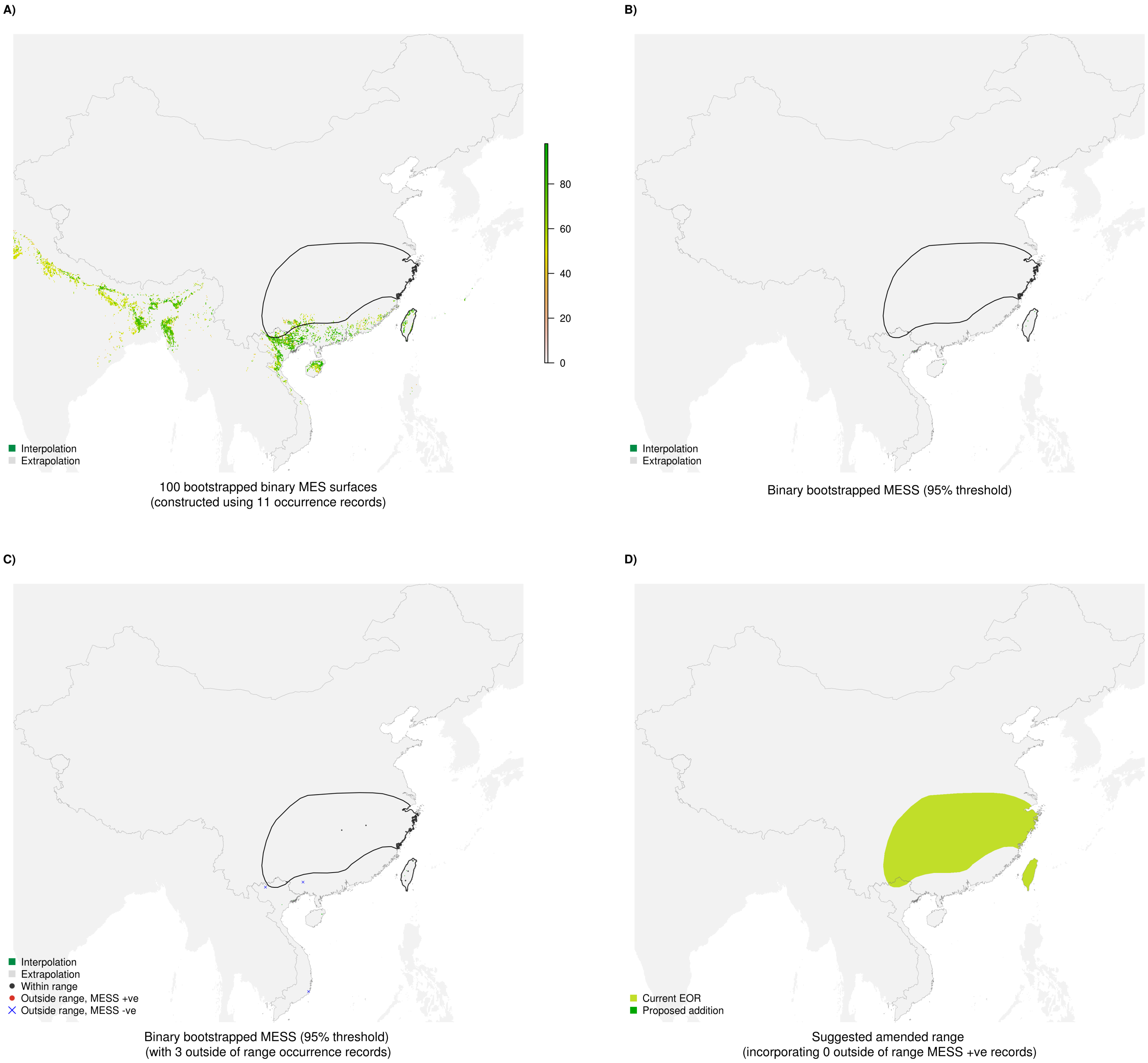

Dendroaspis angusticeps

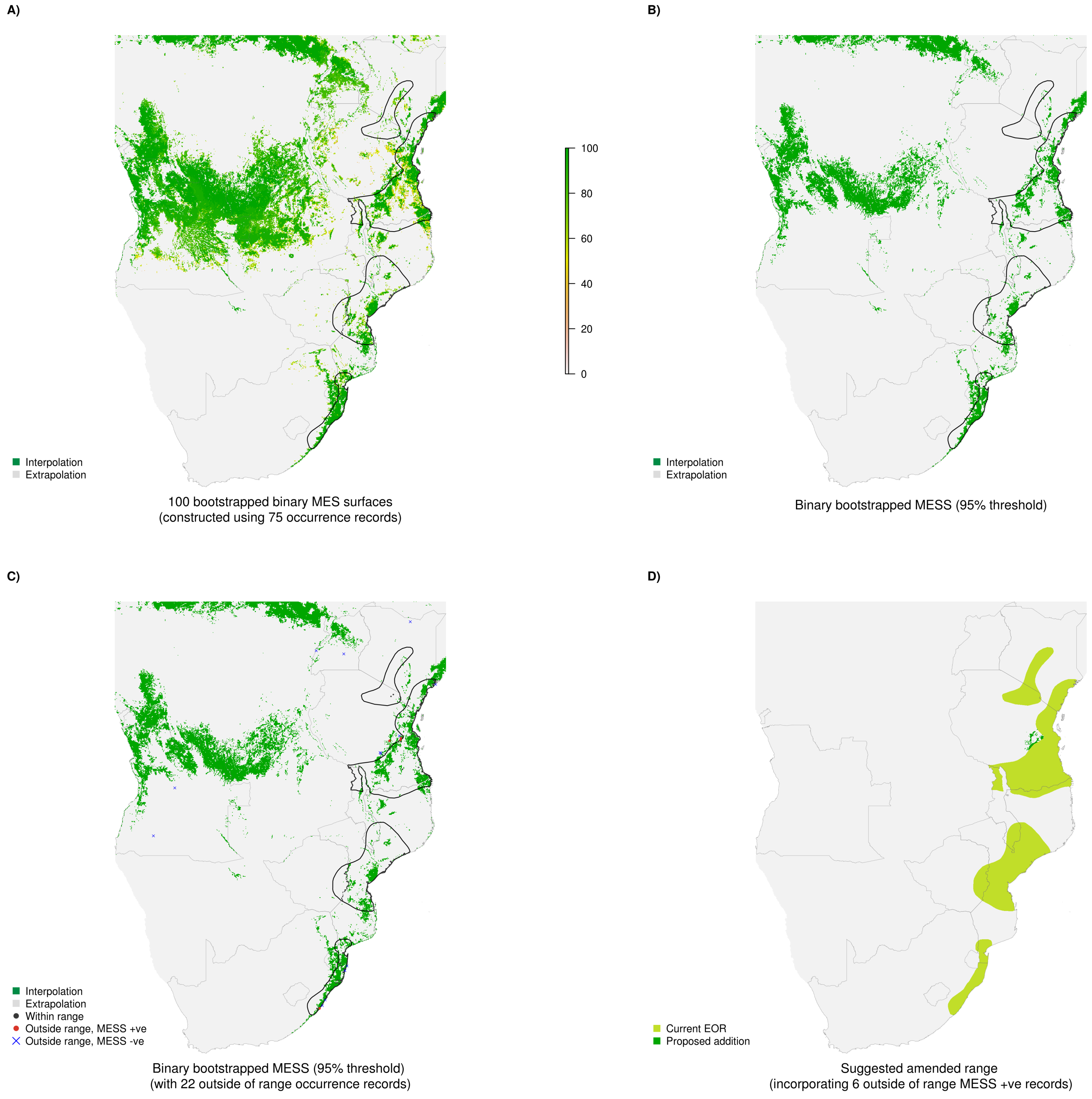

Dendroaspis jamesoni

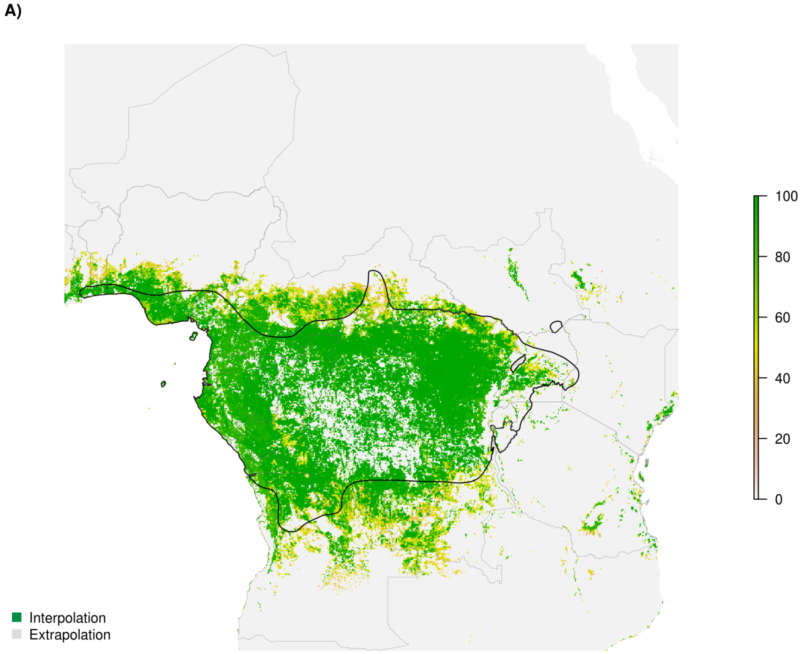

100 bootstrapped binary MES surfaces
(constructed using 99 occurrence records)

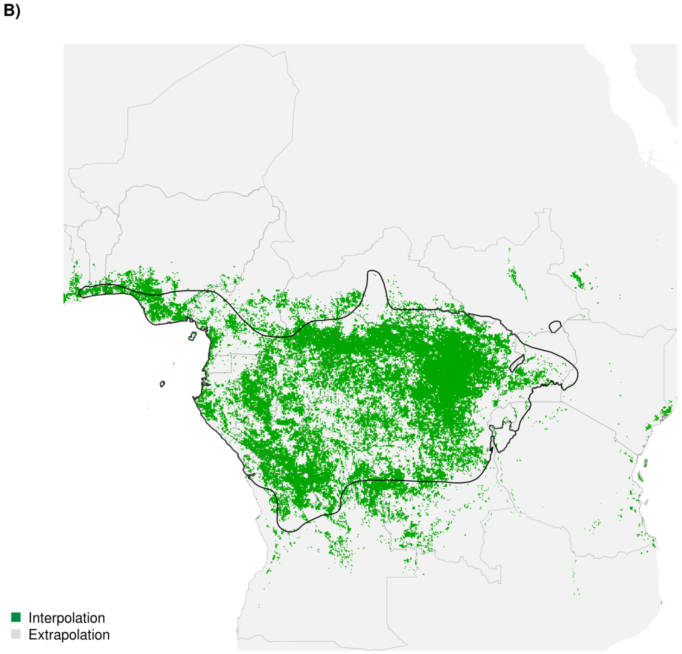

Binary bootstrapped MESS (95% threshold)

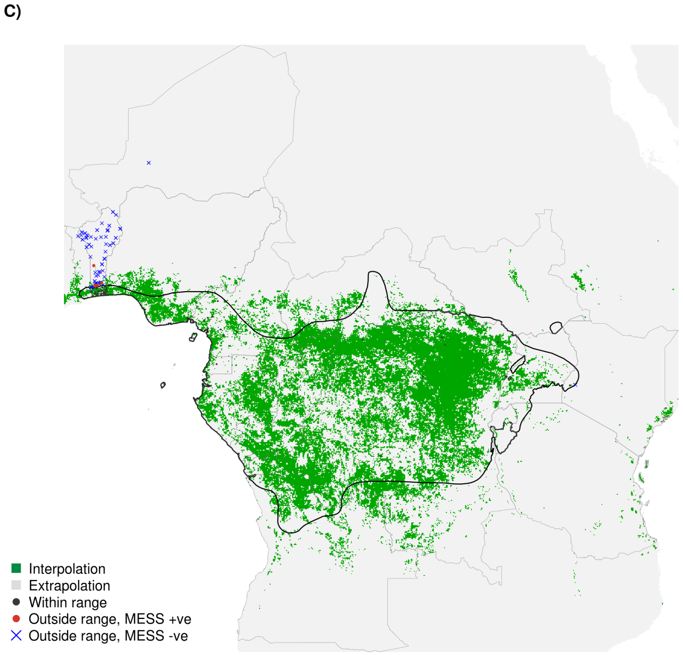

Binary bootstrapped MESS (95% threshold)
(with 81 outside of range occurrence records)

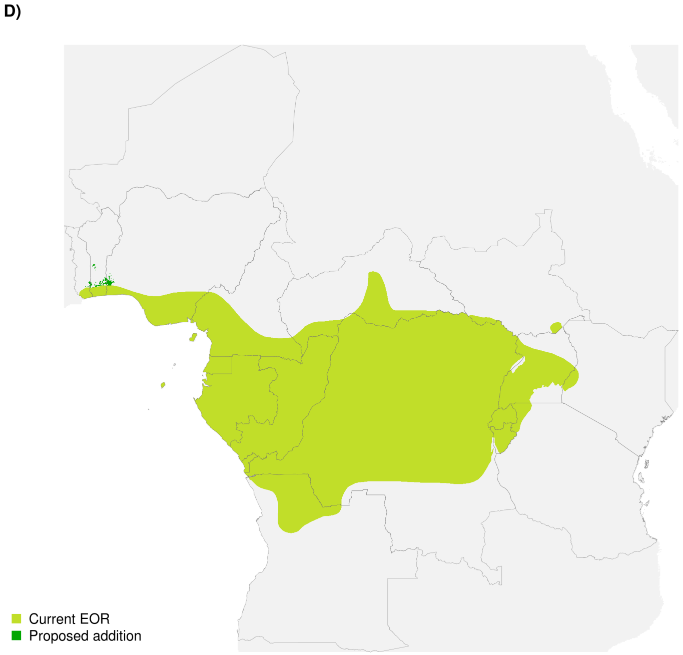

Suggested amended range
(incorporating 5 outside of range MESS +ve records)

Dendroaspis polylepis

A)

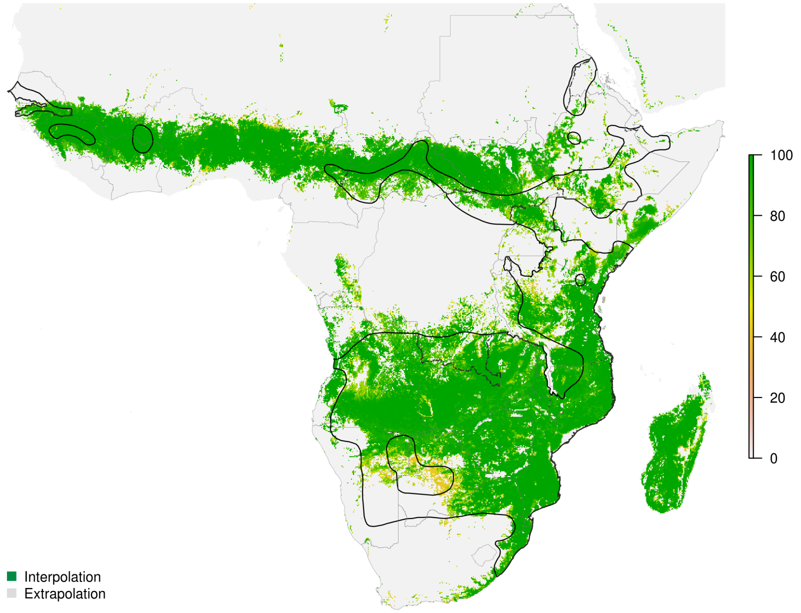

100 bootstrapped binary MES surfaces
(constructed using 139 occurrence records)

B)

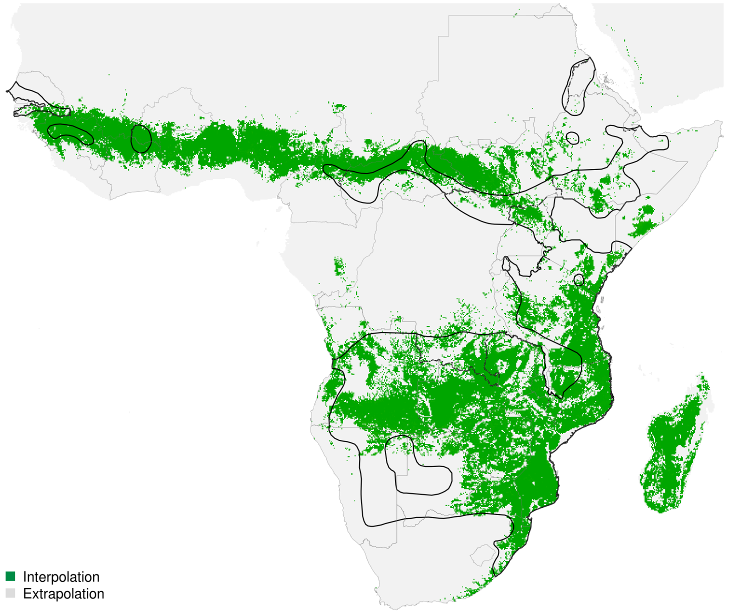

Binary bootstrapped MESS (95% threshold)

C)

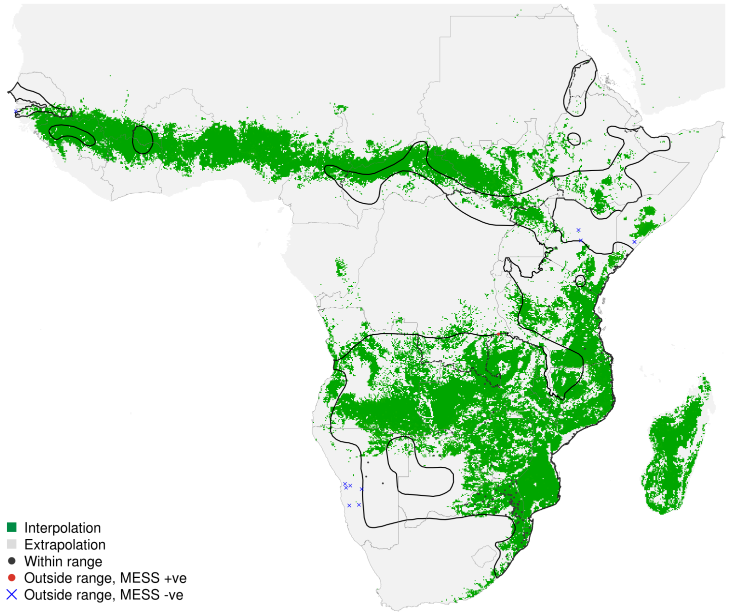

Binary bootstrapped MESS (95% threshold)
(with 13 outside of range occurrence records)

D)

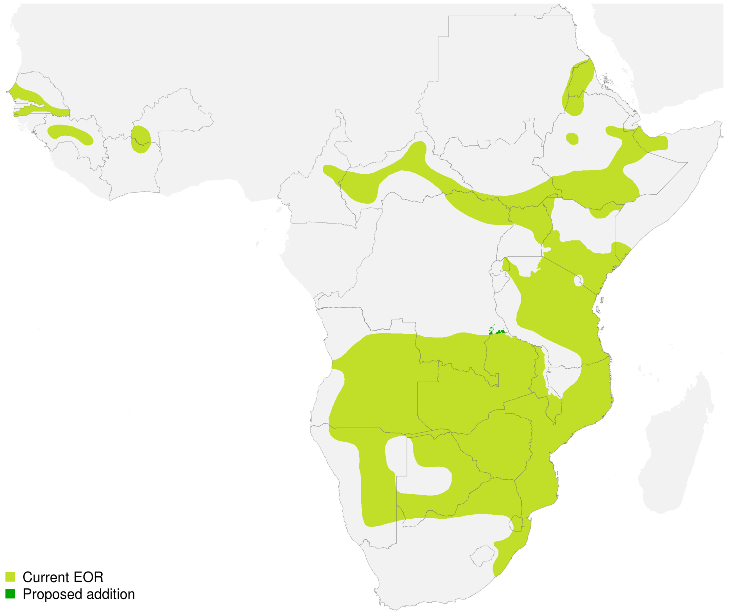

Suggested amended range
(incorporating 1 outside of range MESS +ve records)

Dendroaspis viridis

A)

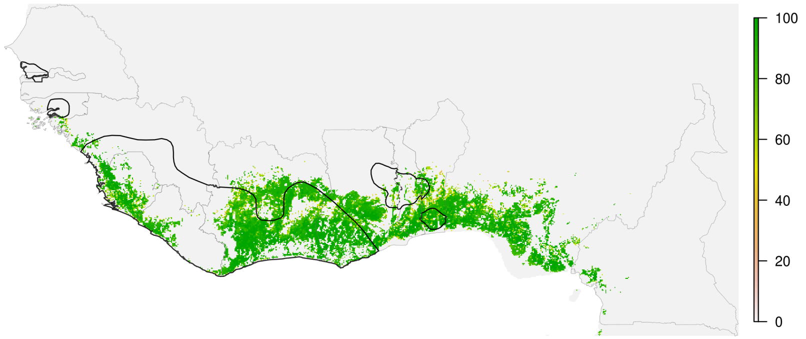

100 bootstrapped binary MES surfaces
(constructed using 59 occurrence records)

B)

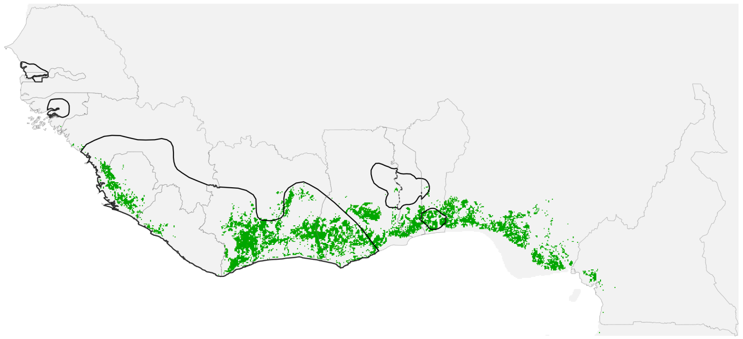

Binary bootstrapped MESS (95% threshold)

C)

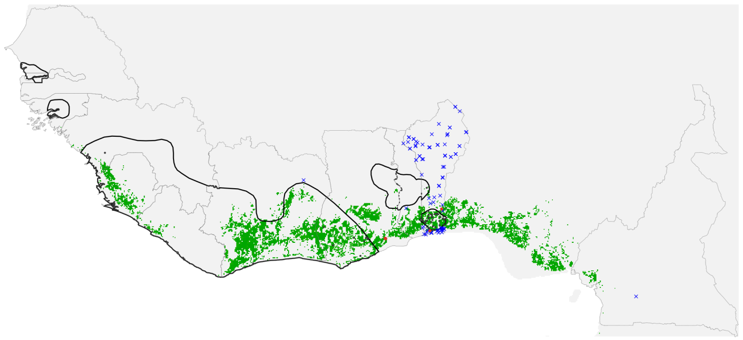

Binary bootstrapped MESS (95% threshold)
(with 94 outside of range occurrence records)

D)

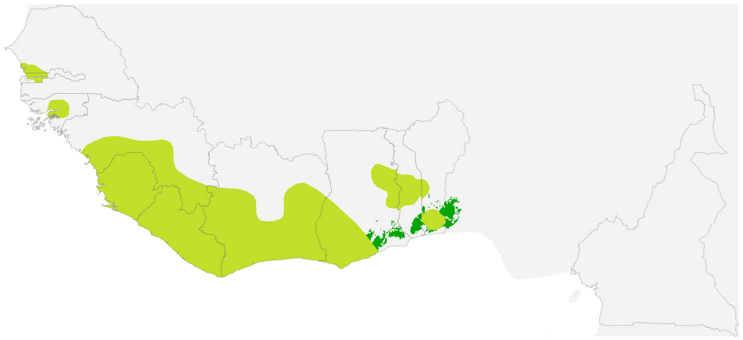

Suggested amended range
(incorporating 7 outside of range MESS +ve records)

Dispholidus typus

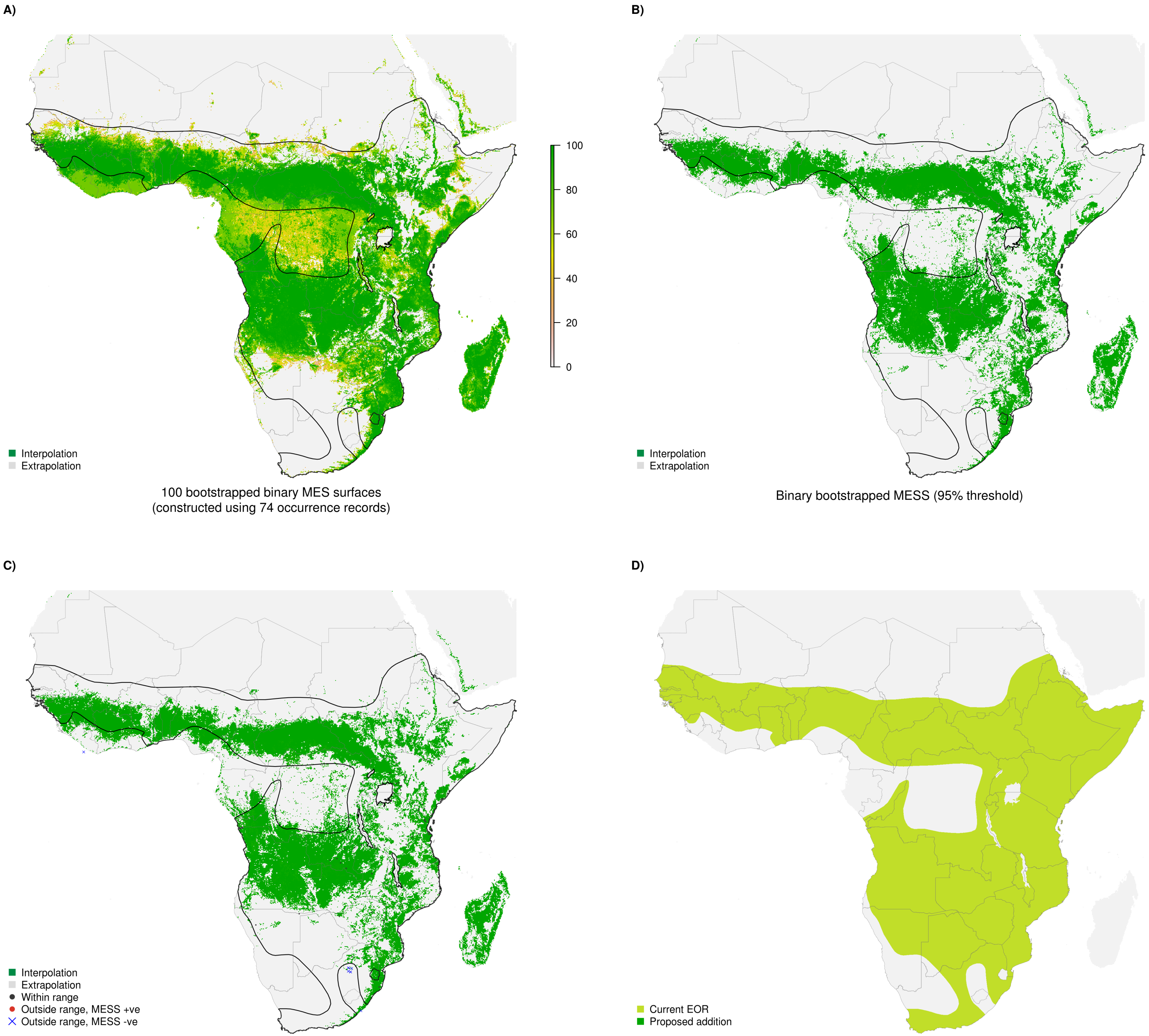

100 bootstrapped binary MES surfaces (constructed using 74 occurrence records)

Binary bootstrapped MESS (95% threshold)

Binary bootstrapped MESS (95% threshold) (with 9 outside of range occurrence records)

Suggested amended range (incorporating 0 outside of range MESS +ve records)

Echis borkini

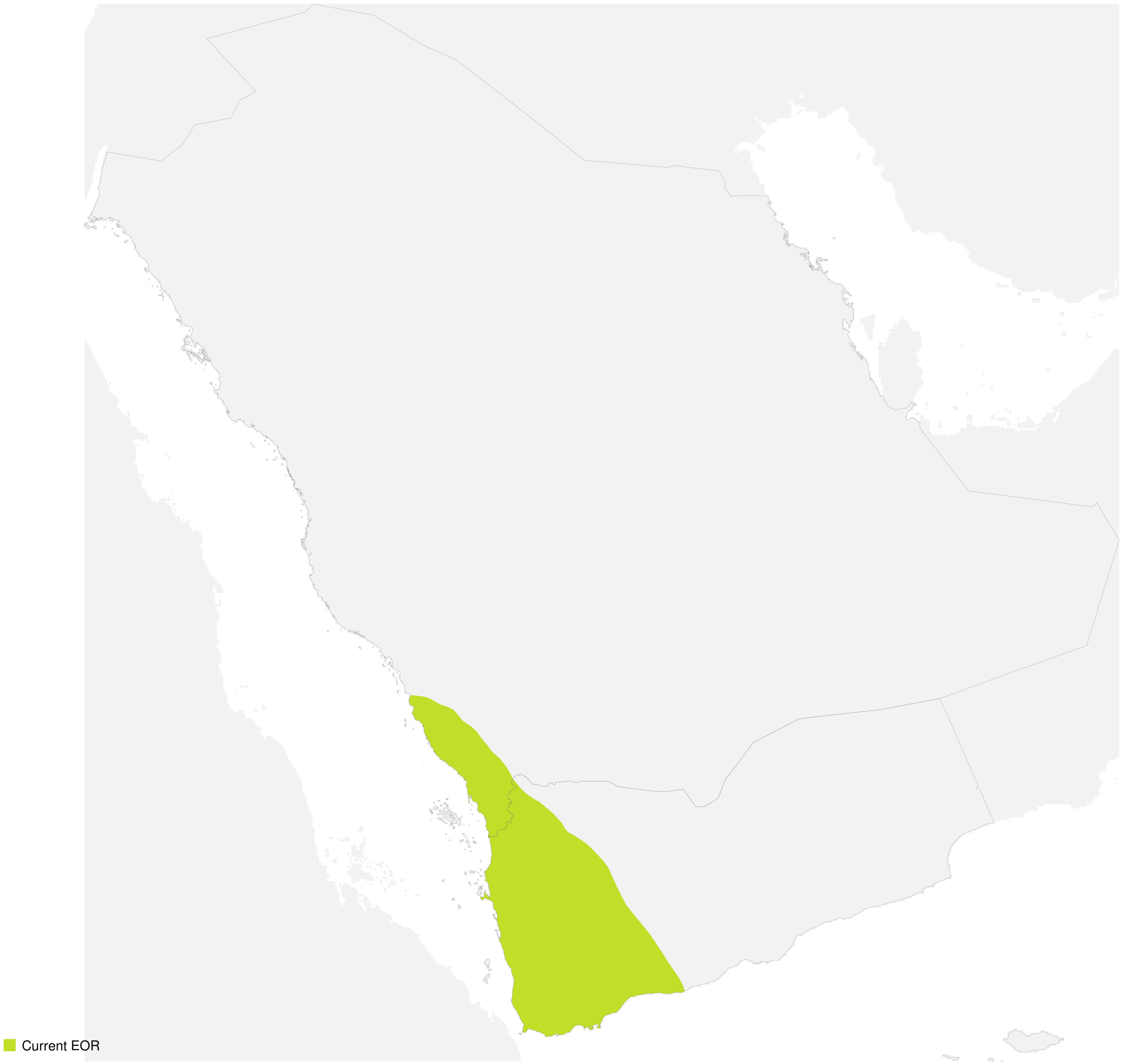

Echis carinatus

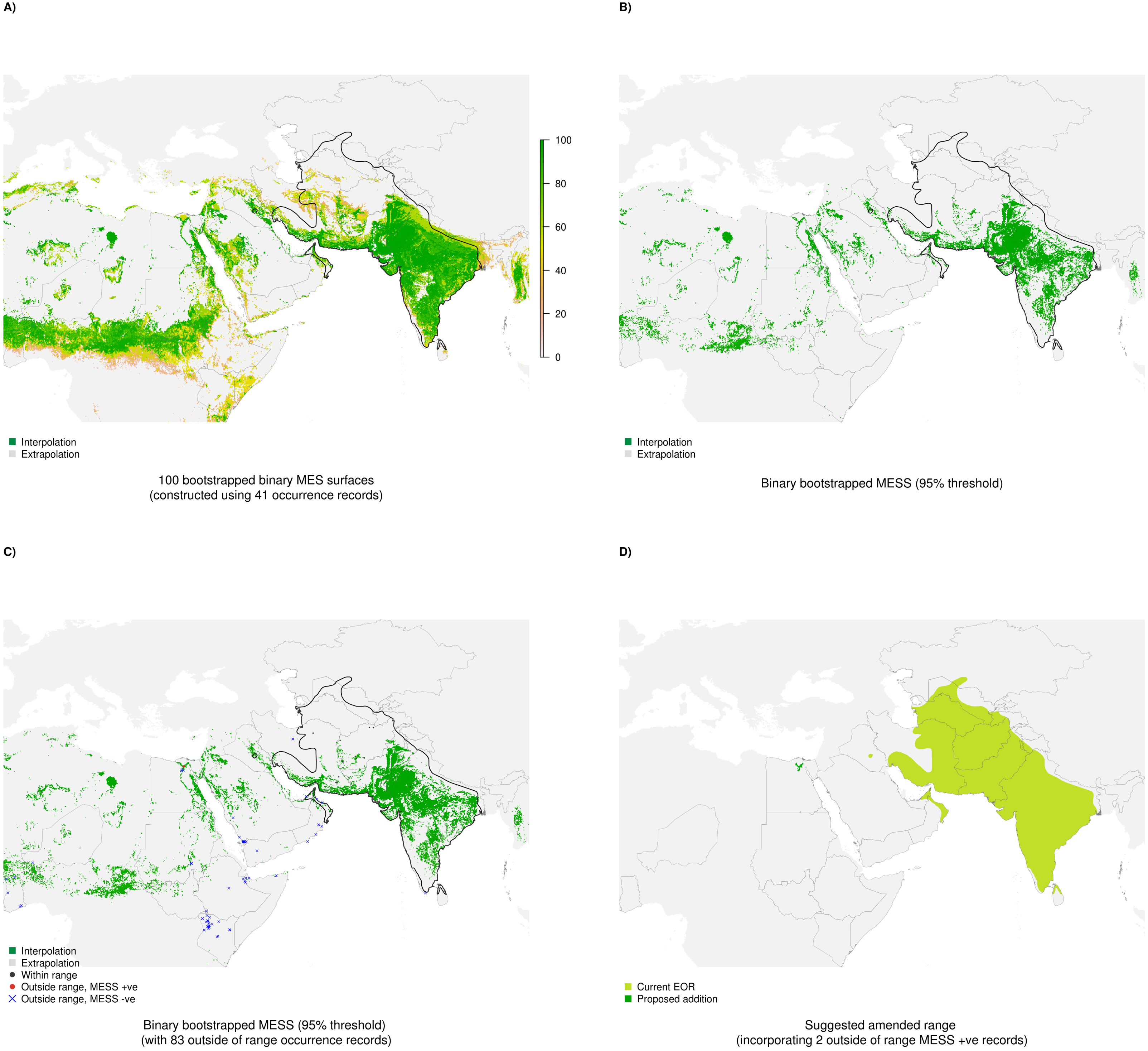

Echis coloratus

A)

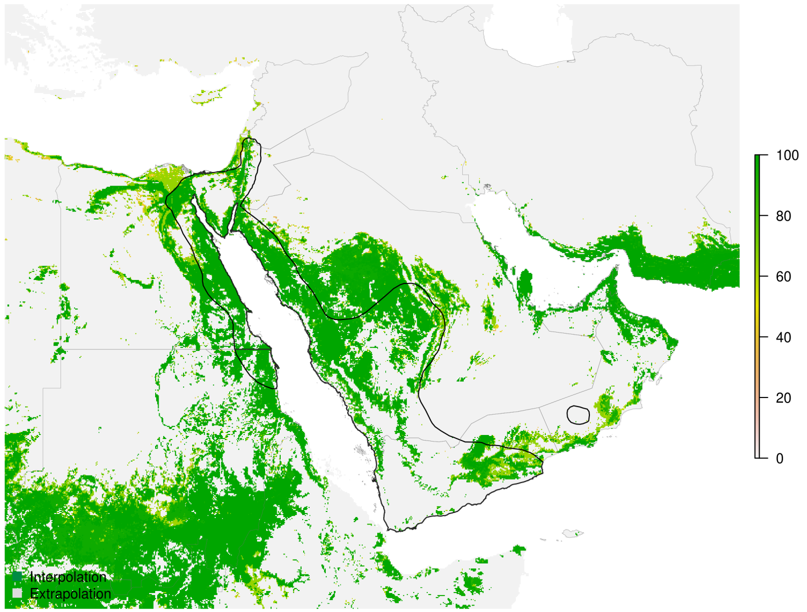

100 bootstrapped binary MES surfaces
(constructed using 123 occurrence records)

B)

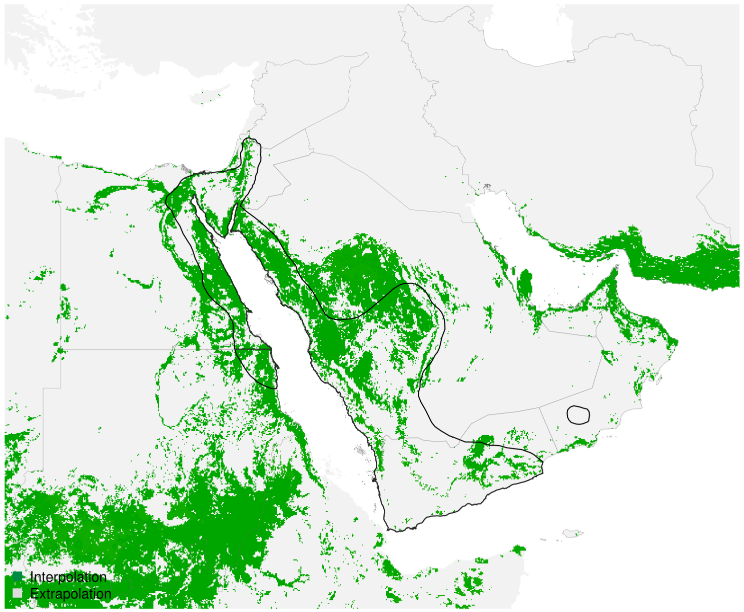

Binary bootstrapped MESS (95% threshold)

C)

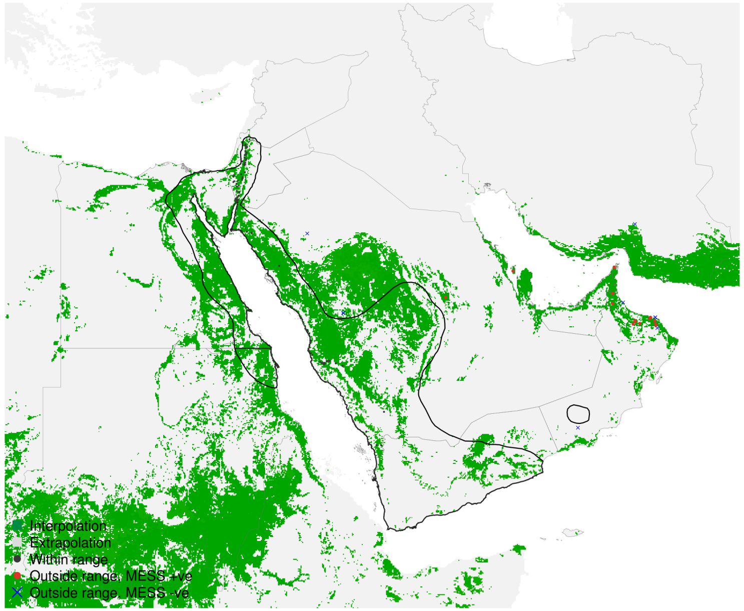

Binary bootstrapped MESS (95% threshold)
(with 25 outside of range occurrence records)

D)

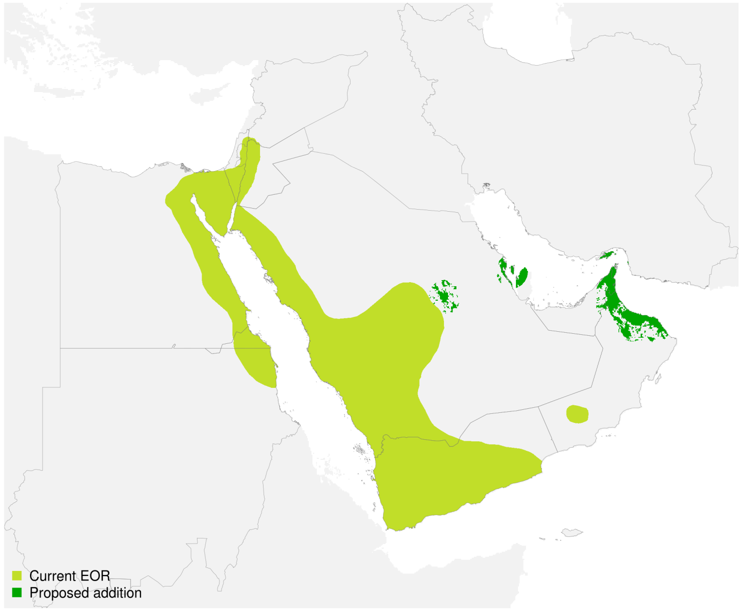

Suggested amended range
(incorporating 16 outside of range MESS +ve records)

Echis hughesi

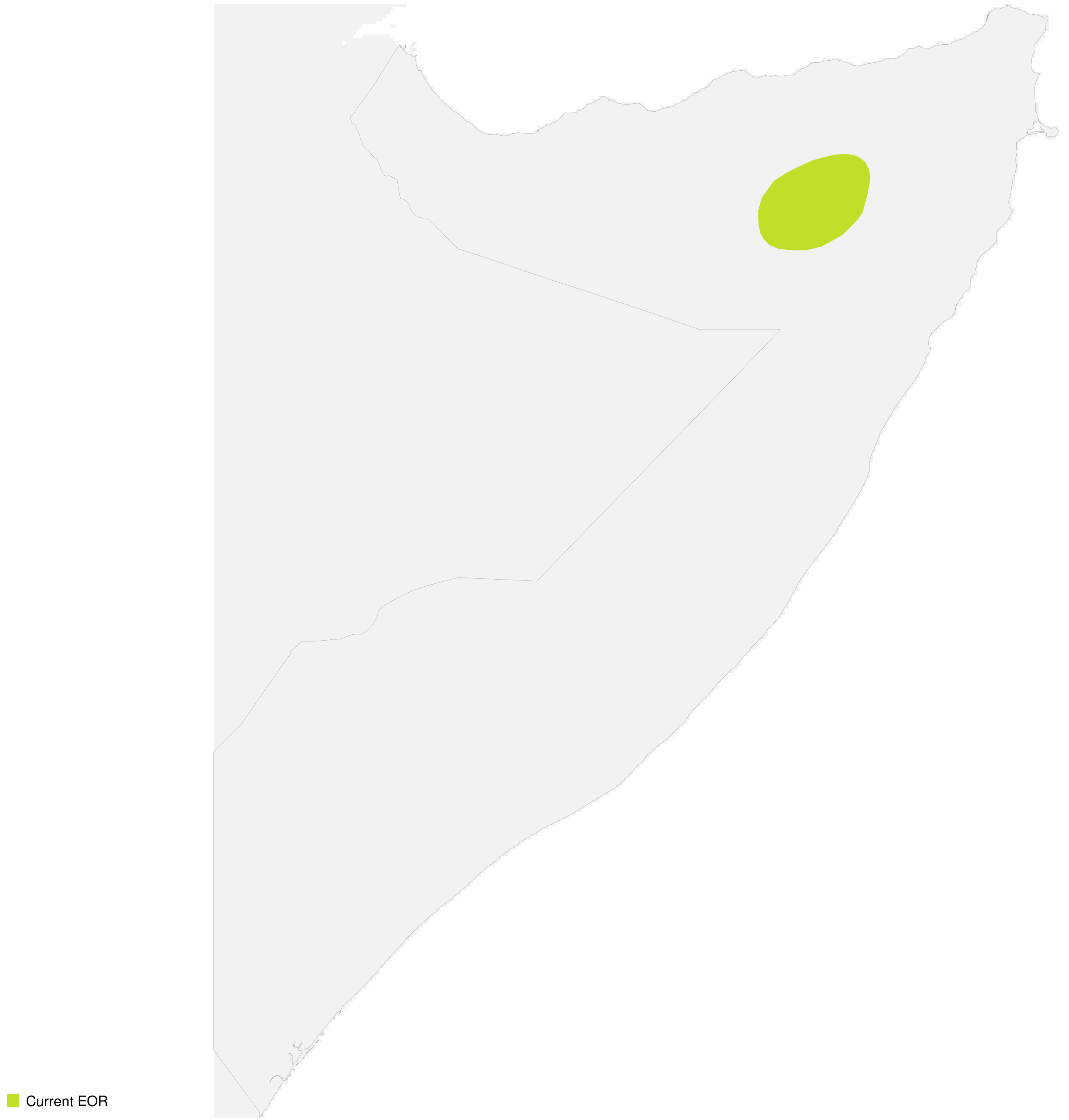

Echis jogeri

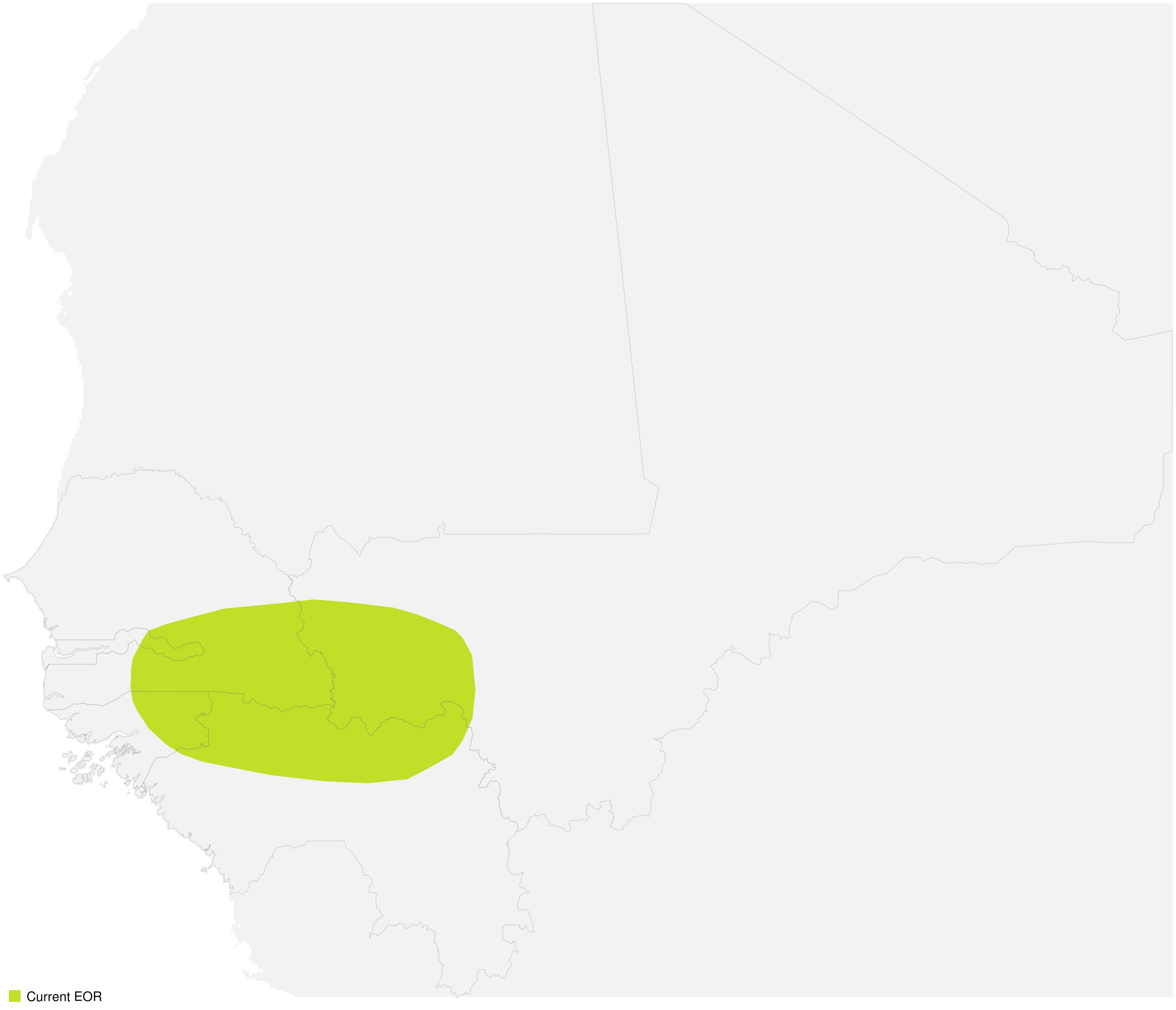

Echis khosatzkii

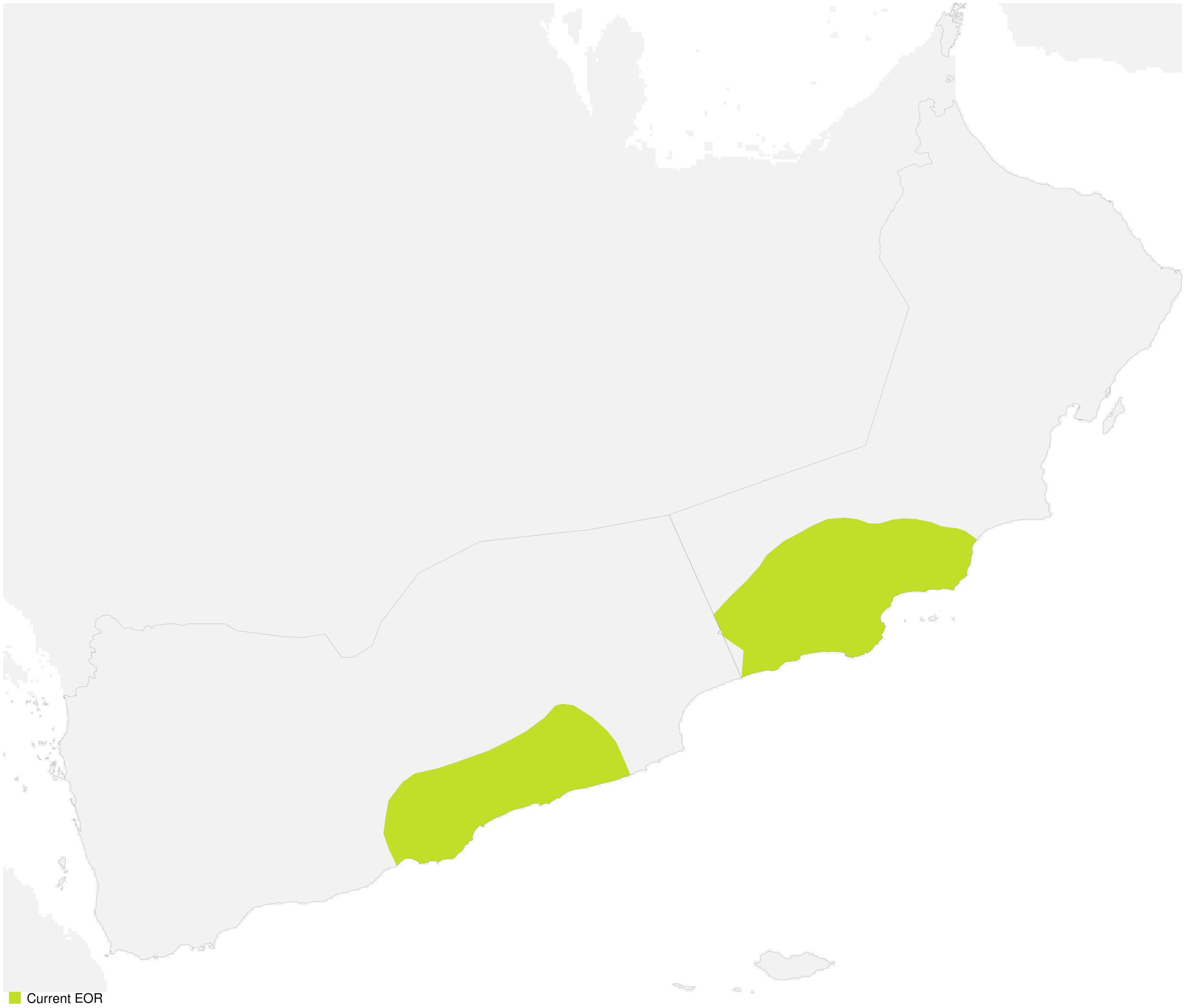

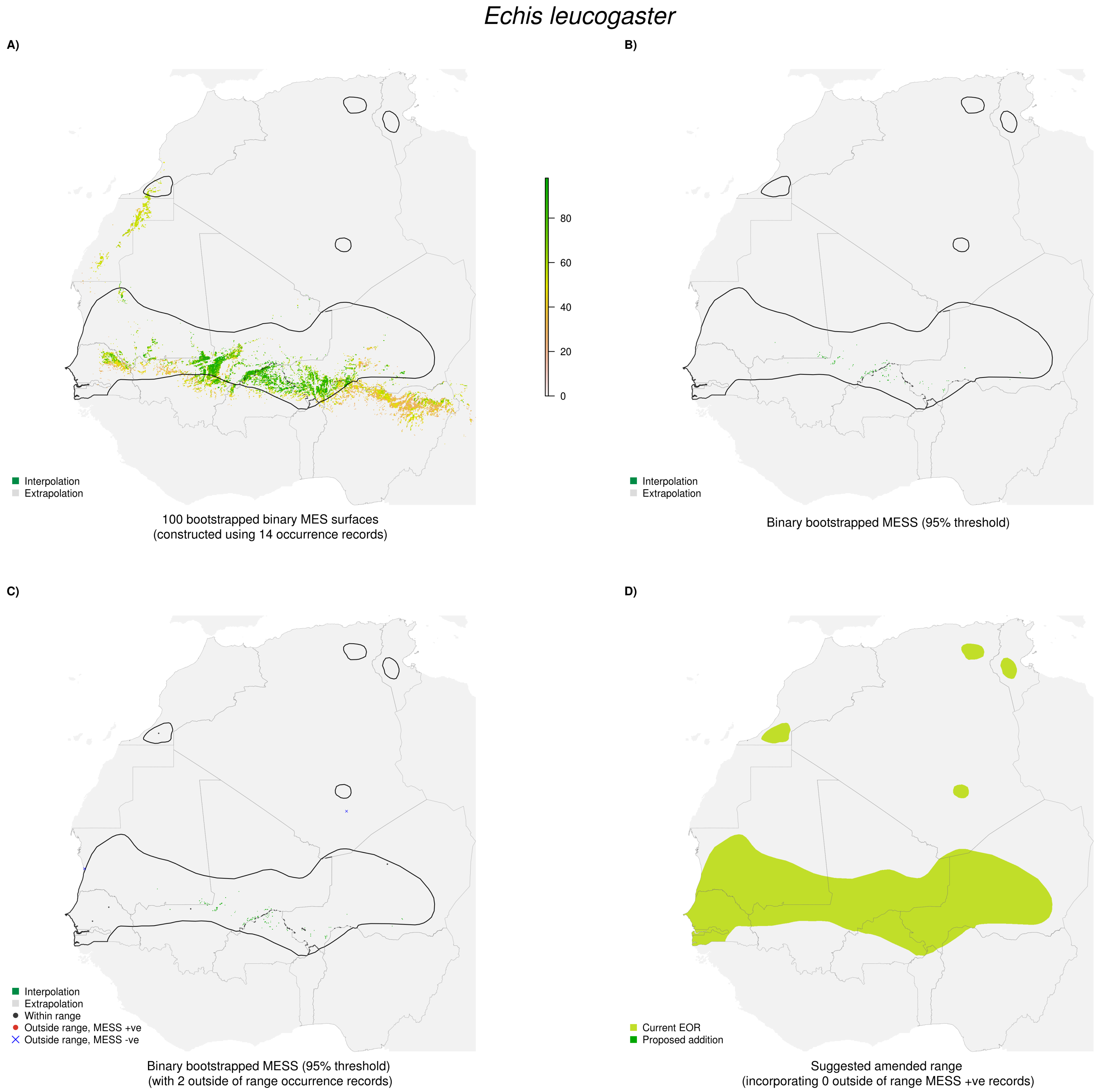
Echis leucogaster
A)
80
60
40
20
0
Interpolation
Extrapolation
100 bootstrapped binary MES surfaces
(constructed using 14 occurrence records)
B)
Interpolation
Extrapolation
Binary bootstrapped MESS (95% threshold)
C)
Interpolation
Extrapolation
Within range
Outside range, MESS +ve
Outside range, MESS -ve
Binary bootstrapped MESS (95% threshold)
(with 2 outside of range occurrence records)
D)
Current EOR
Proposed addition
Suggested amended range
(incorporating 0 outside of range MESS +ve records)

Echis megalocephalus

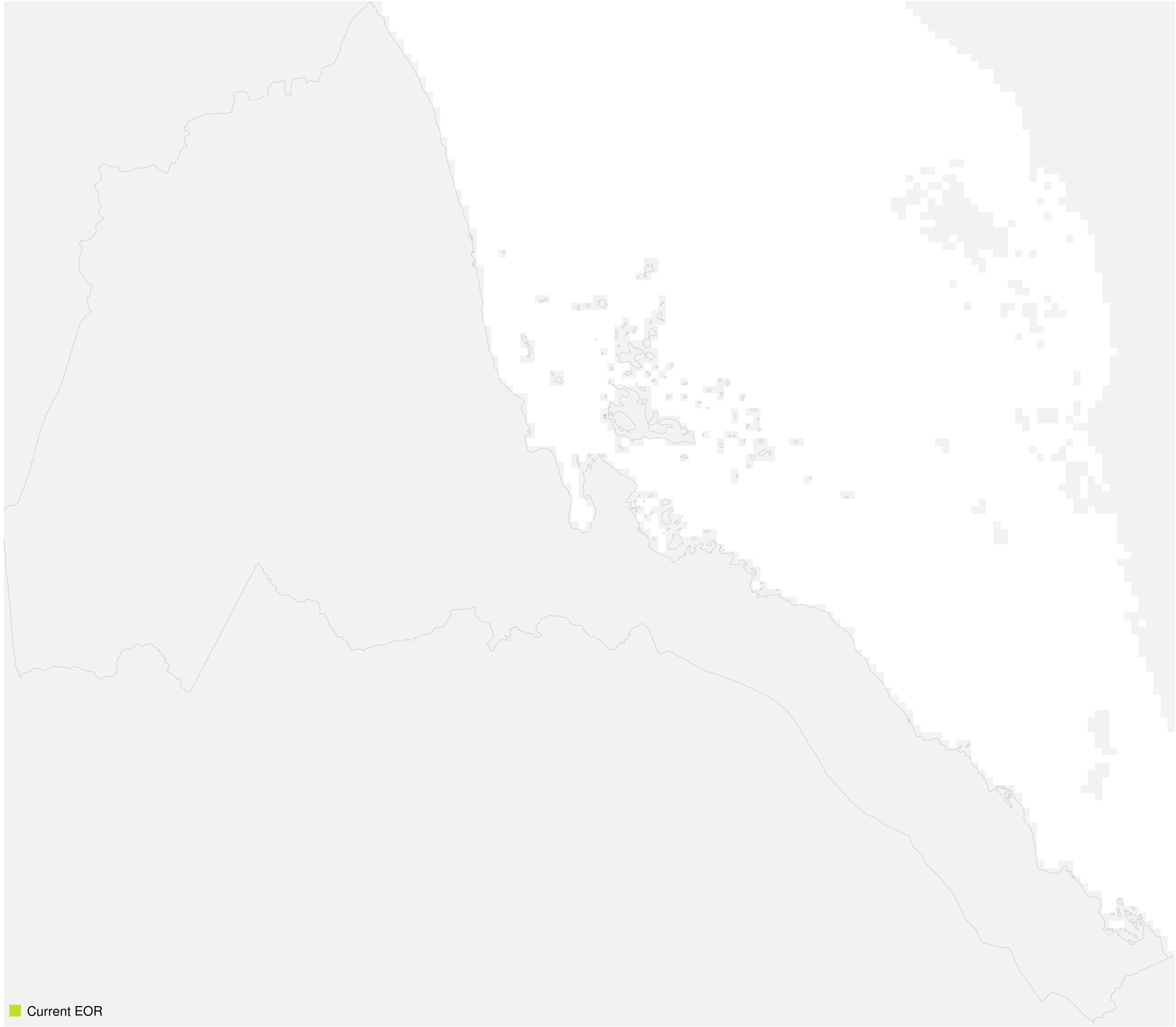

Echis ocellatus

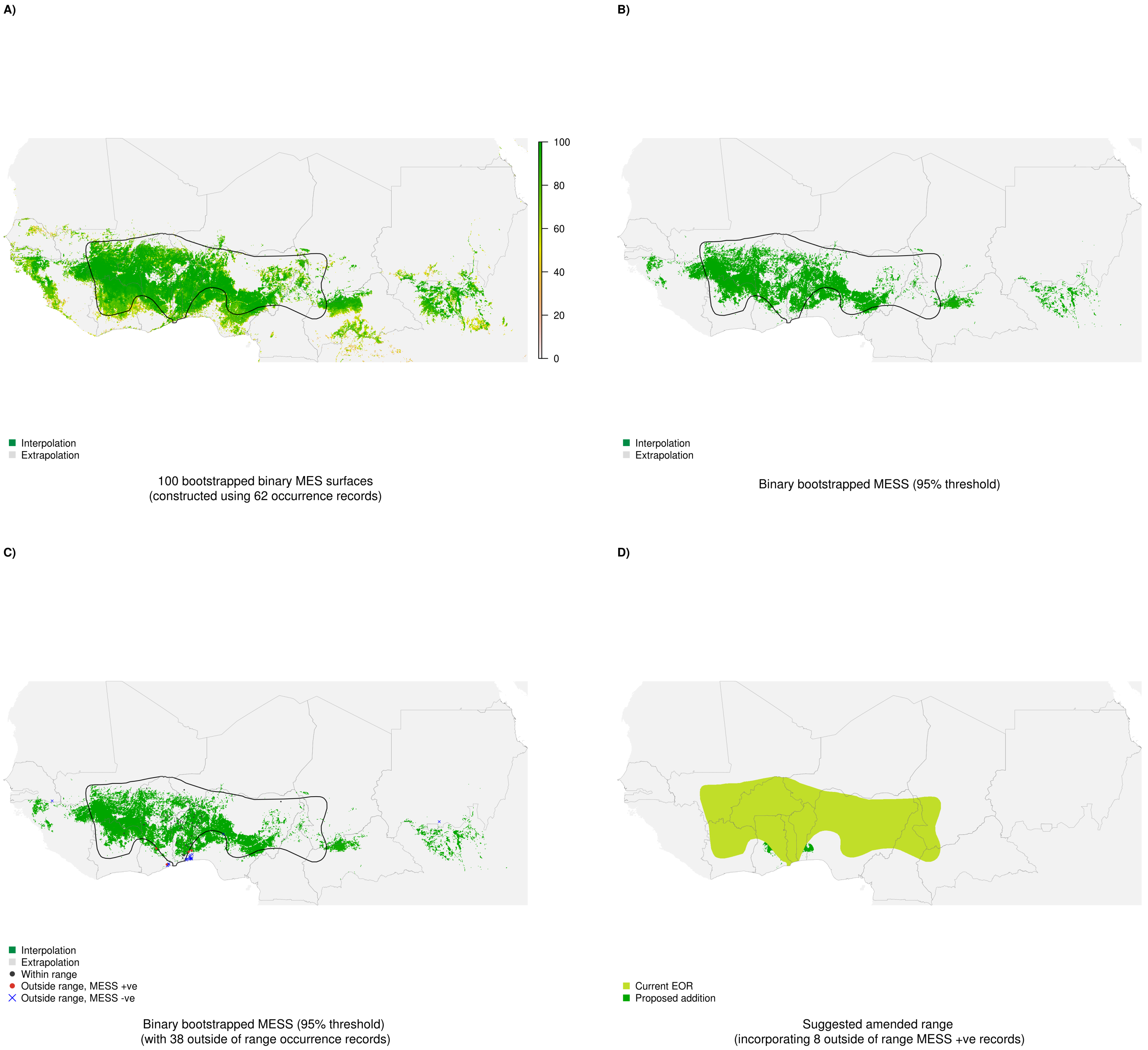

Echis omanensis

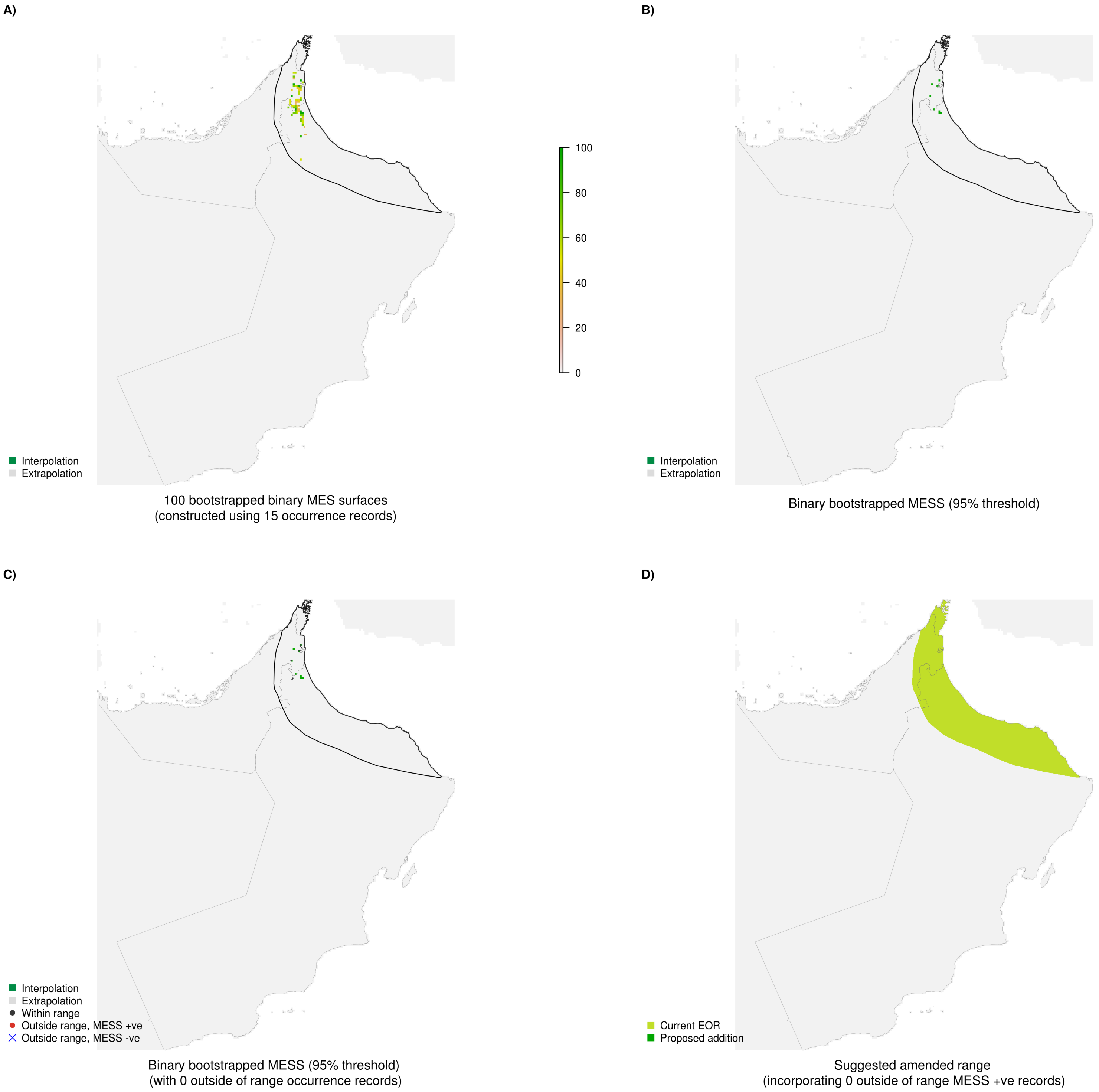

Echis pyramidum

A)

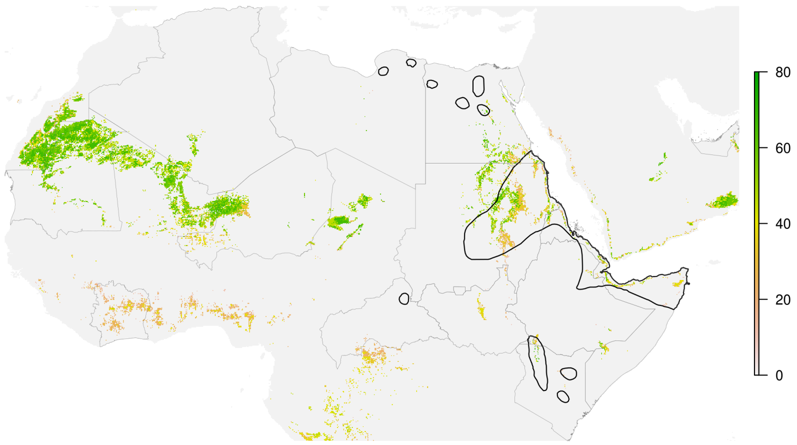

100 bootstrapped binary MES surfaces
(constructed using 6 occurrence records)

B)

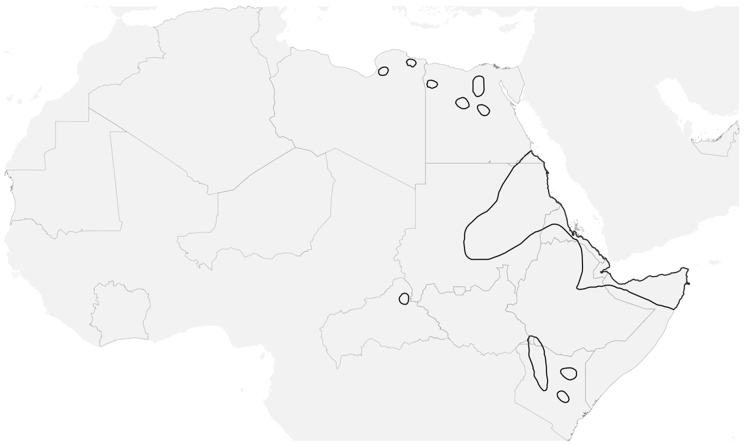

Binary bootstrapped MESS (95% threshold)
[0 cells met this threshold]

B)

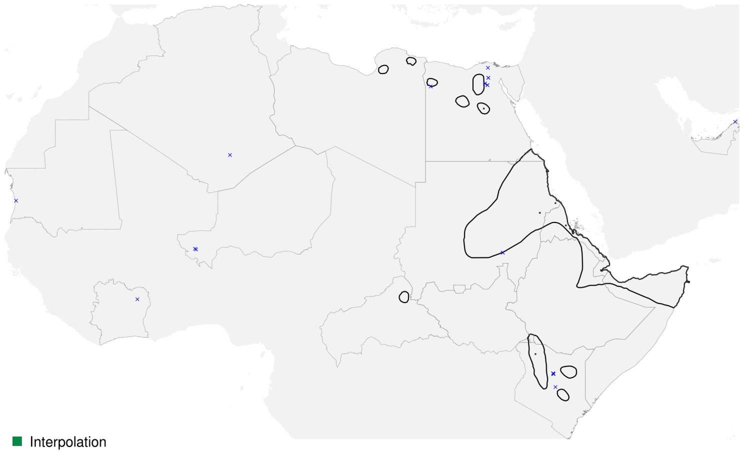

Binary bootstrapped MESS (95% threshold)
(with 23 outside of range occurrence records)

D)

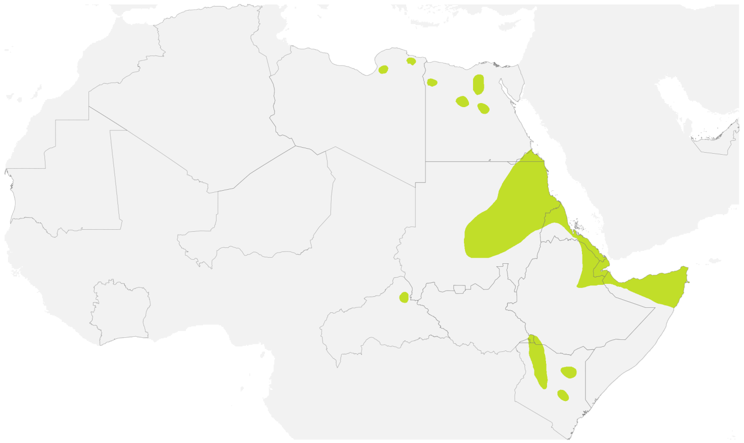

Suggested amended range
(incorporating 0 outside of range MESS +ve records)

Eristocophis macmahoni

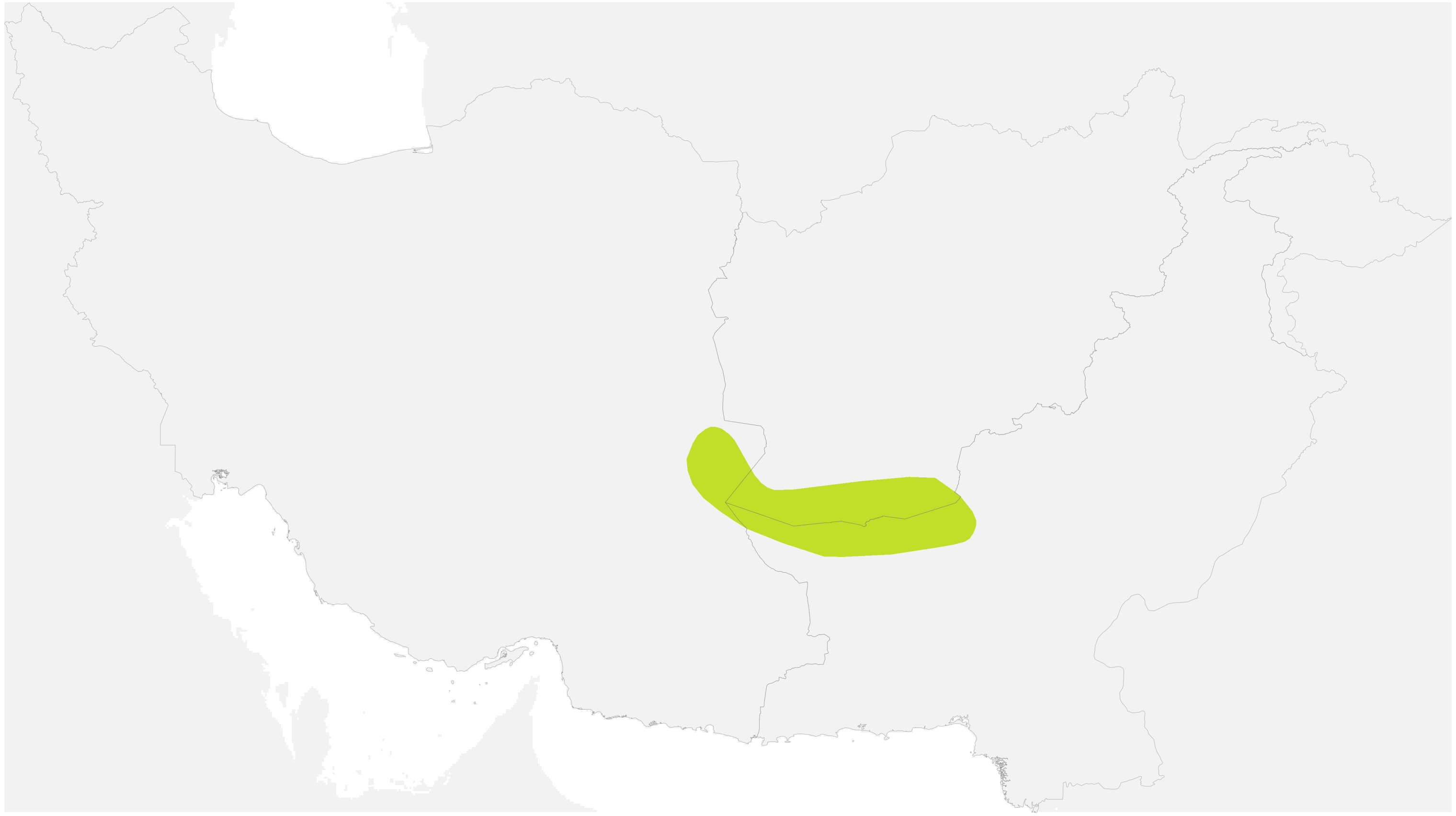

Current EOR

Gloydius blomhoffi

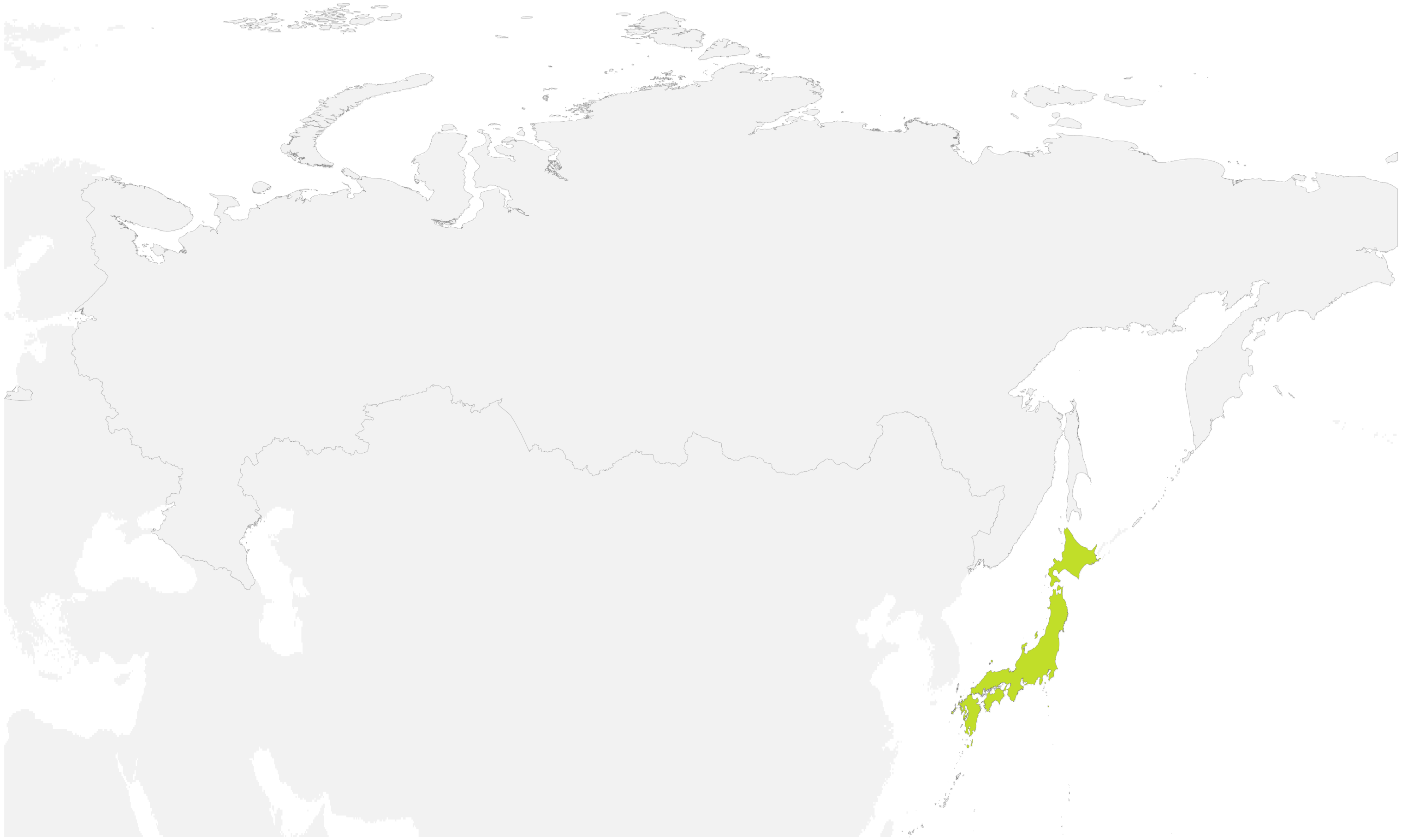

Current EOR

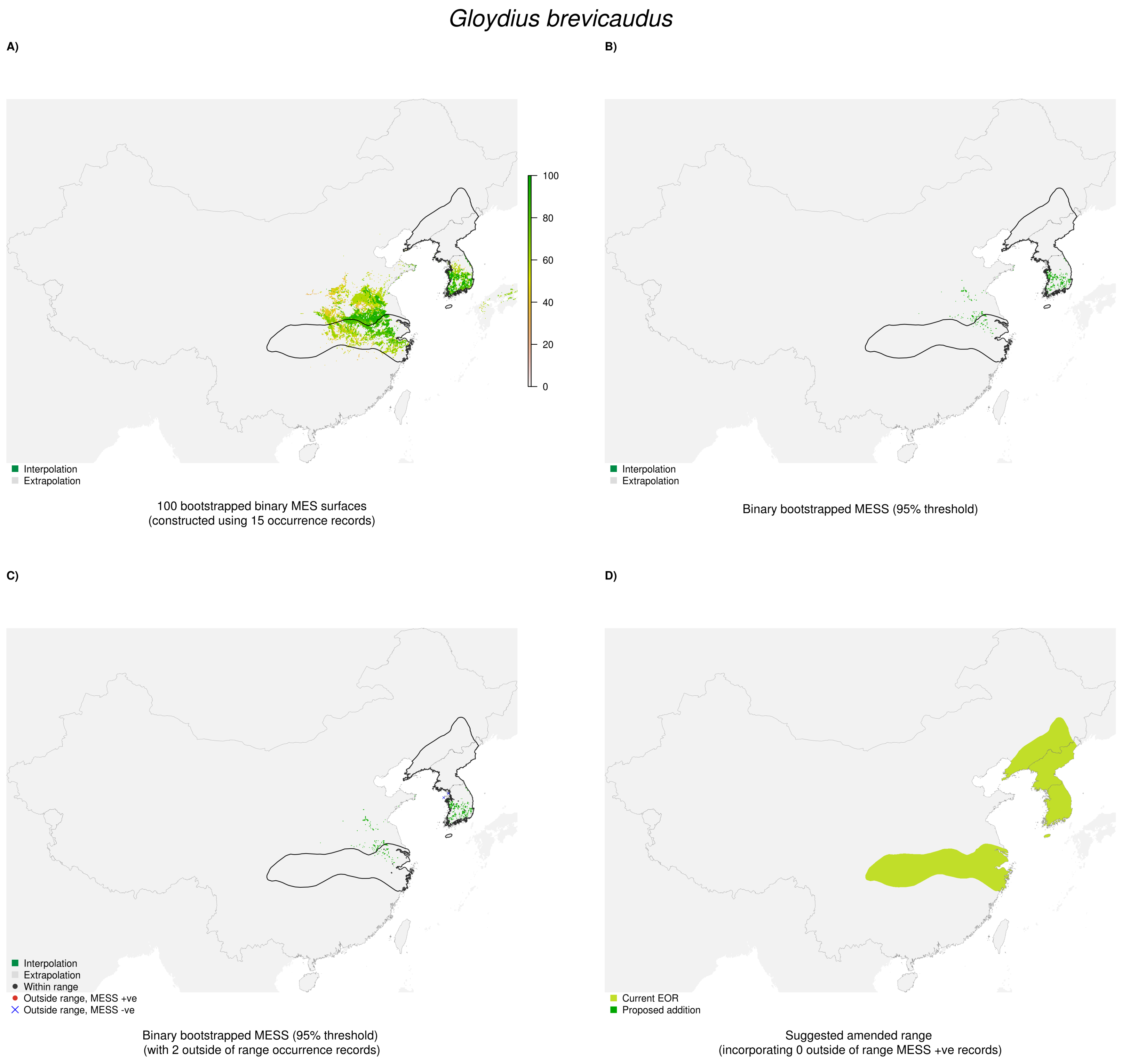
Gloydius brevicaudus
A)
100
80
60
40
20
0
Interpolation
Extrapolation
100 bootstrapped binary MES surfaces
(constructed using 15 occurrence records)
B)
Interpolation
Extrapolation
Binary bootstrapped MESS (95% threshold)
C)
Interpolation
Extrapolation
Within range
Outside range, MESS +ve
Outside range, MESS -ve
Binary bootstrapped MESS (95% threshold)
(with 2 outside of range occurrence records)
D)
Current EOR
Proposed addition
Suggested amended range
(incorporating 0 outside of range MESS +ve records)

Gloydius halys caucasicus

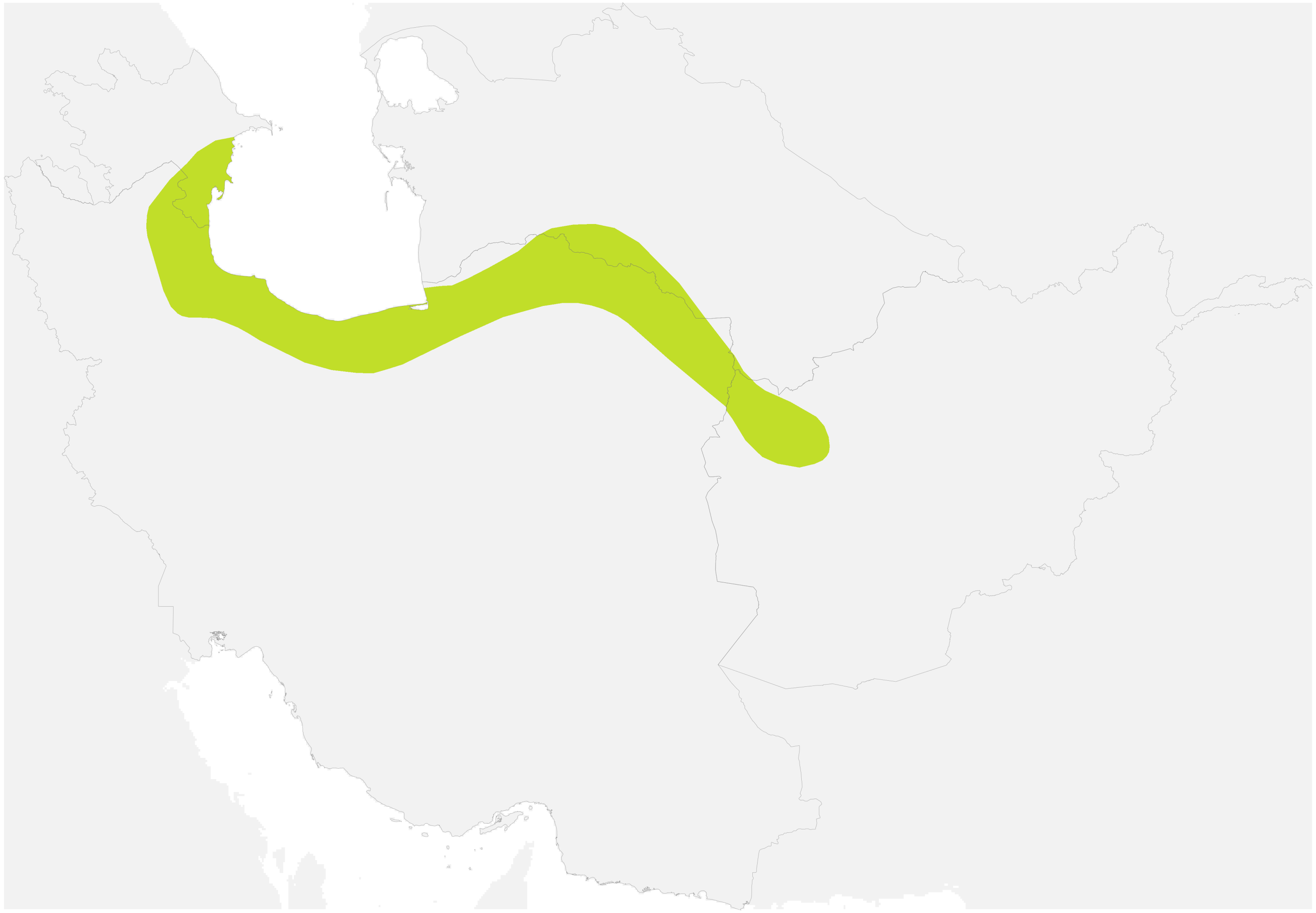

Current EOR

Gloydius halys

A)

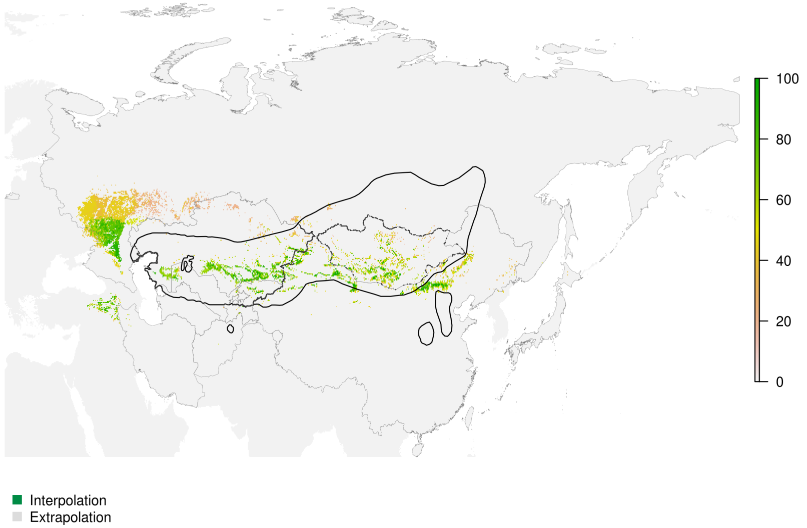

100 bootstrapped binary MES surfaces
(constructed using 15 occurrence records)

B)

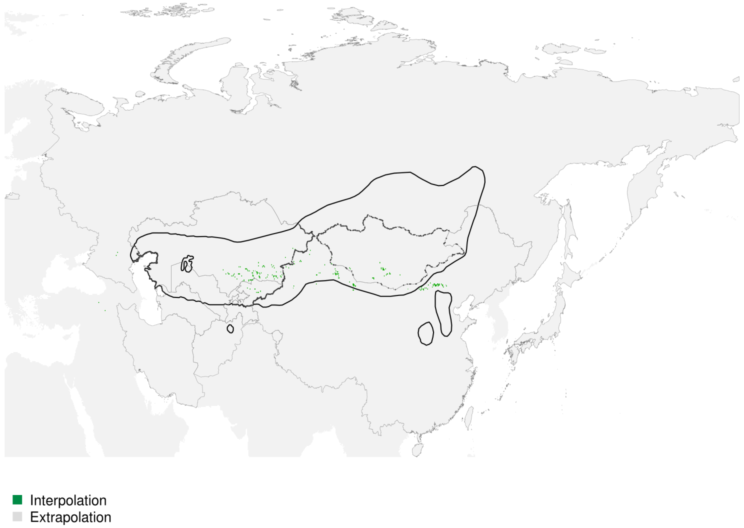

Binary bootstrapped MESS (95% threshold)

C)

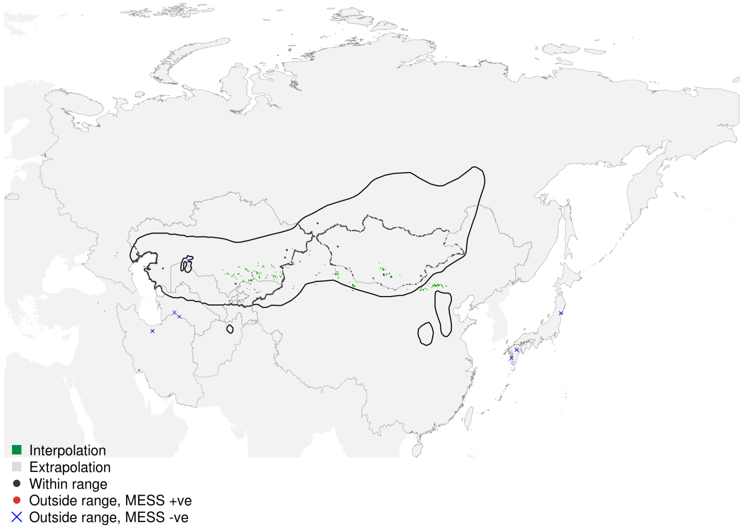

Binary bootstrapped MESS (95% threshold)
(with 12 outside of range occurrence records)

D)

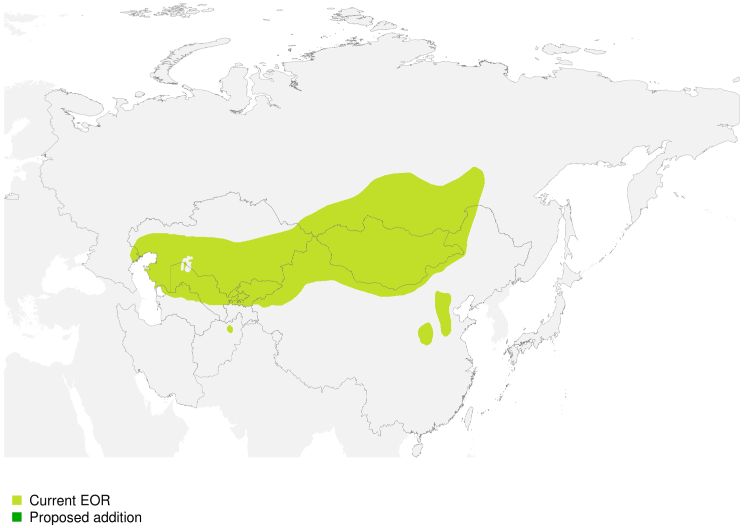

Suggested amended range
(incorporating 0 outside of range MESS +ve records)

Gloydius himalayanus

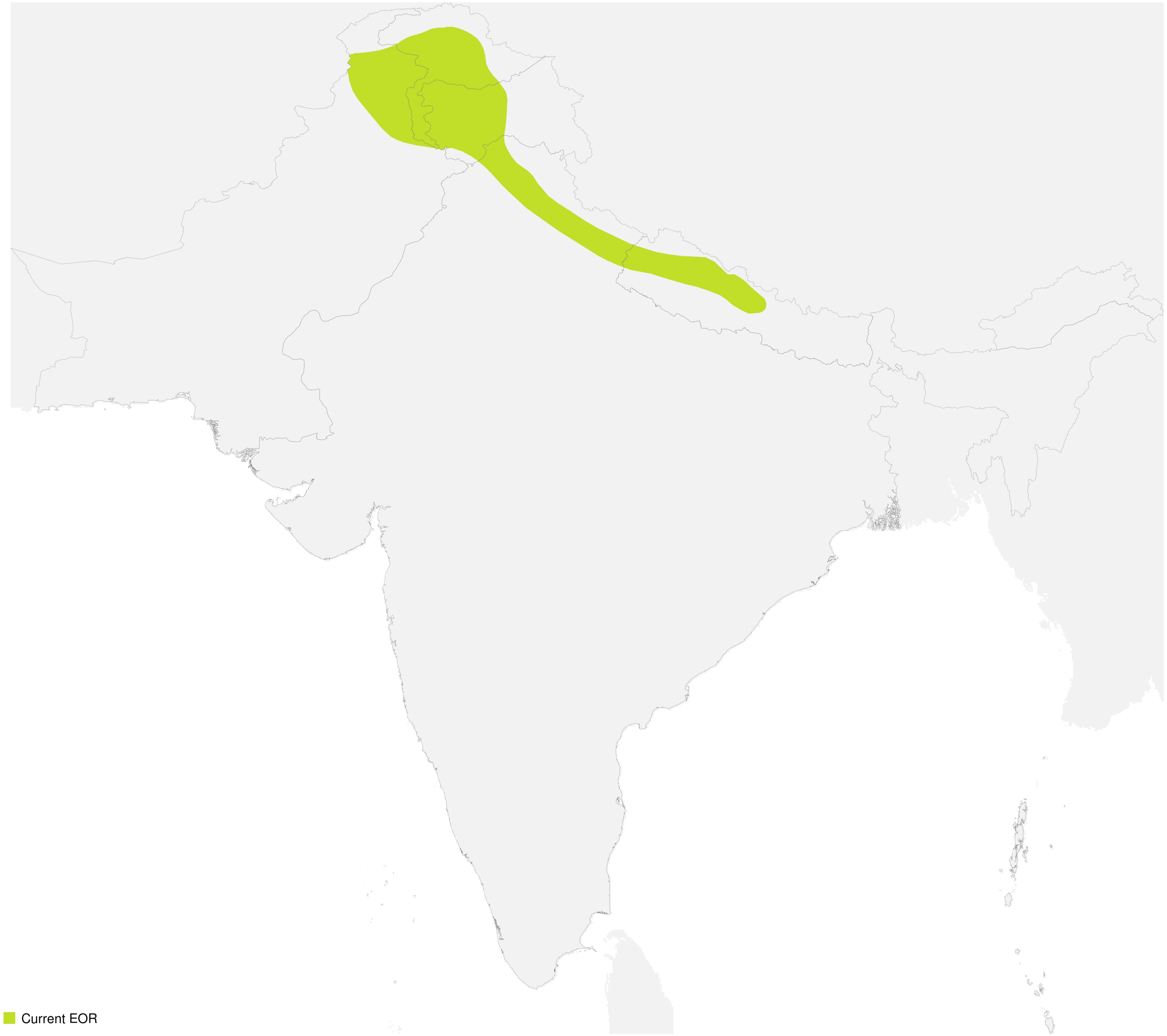

Gloydius intermedius

A)

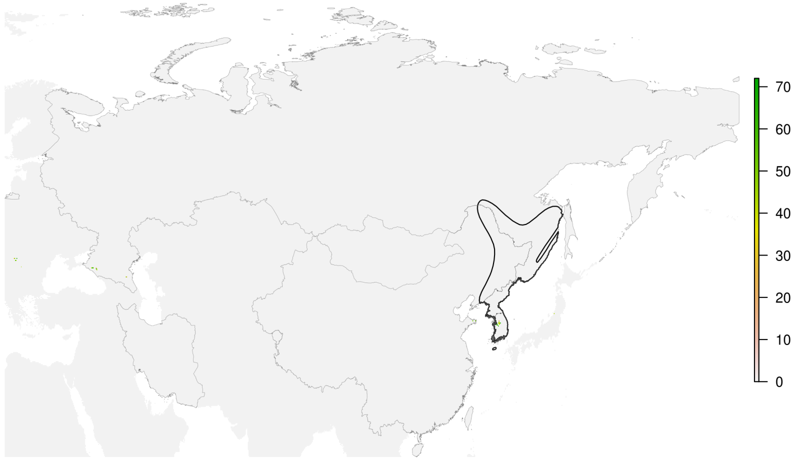

100 bootstrapped binary MES surfaces
(constructed using 6 occurrence records)

B)

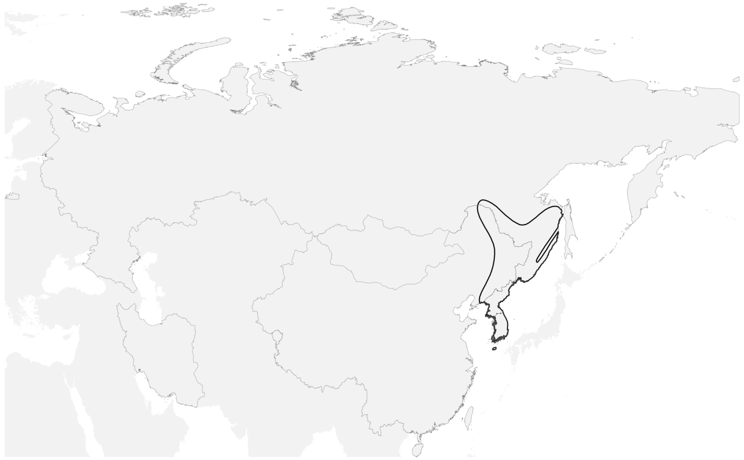

Binary bootstrapped MESS (95% threshold)
[0 cells met this threshold]

B)

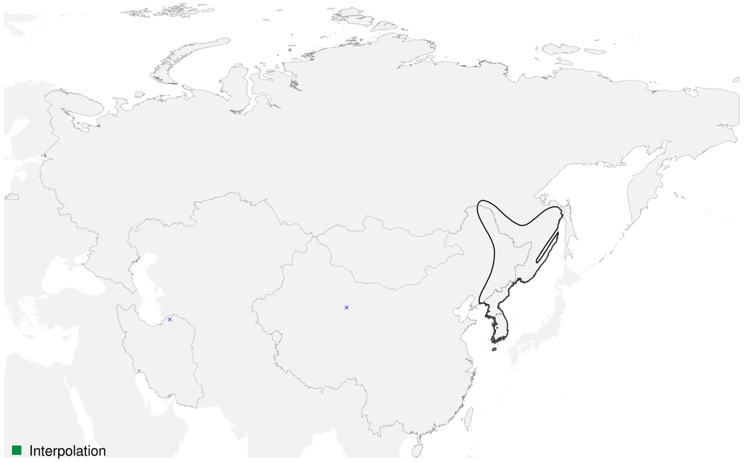

Binary bootstrapped MESS (95% threshold)
(with 2 outside of range occurrence records)

D)

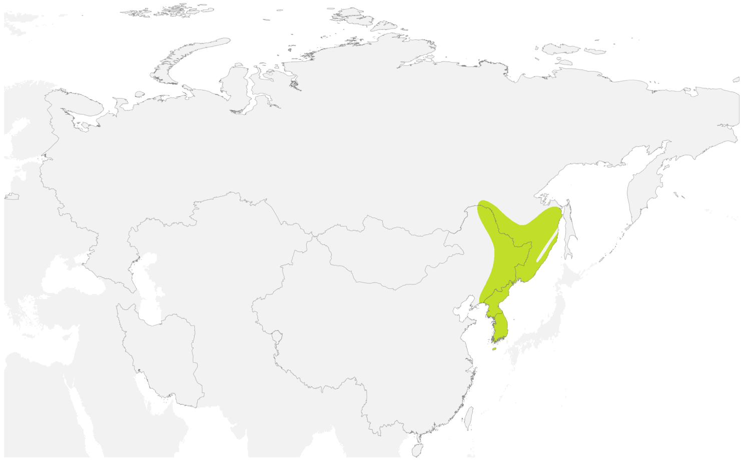

Suggested amended range
(incorporating 0 outside of range MESS +ve records)

Gloydius tsushimaensis

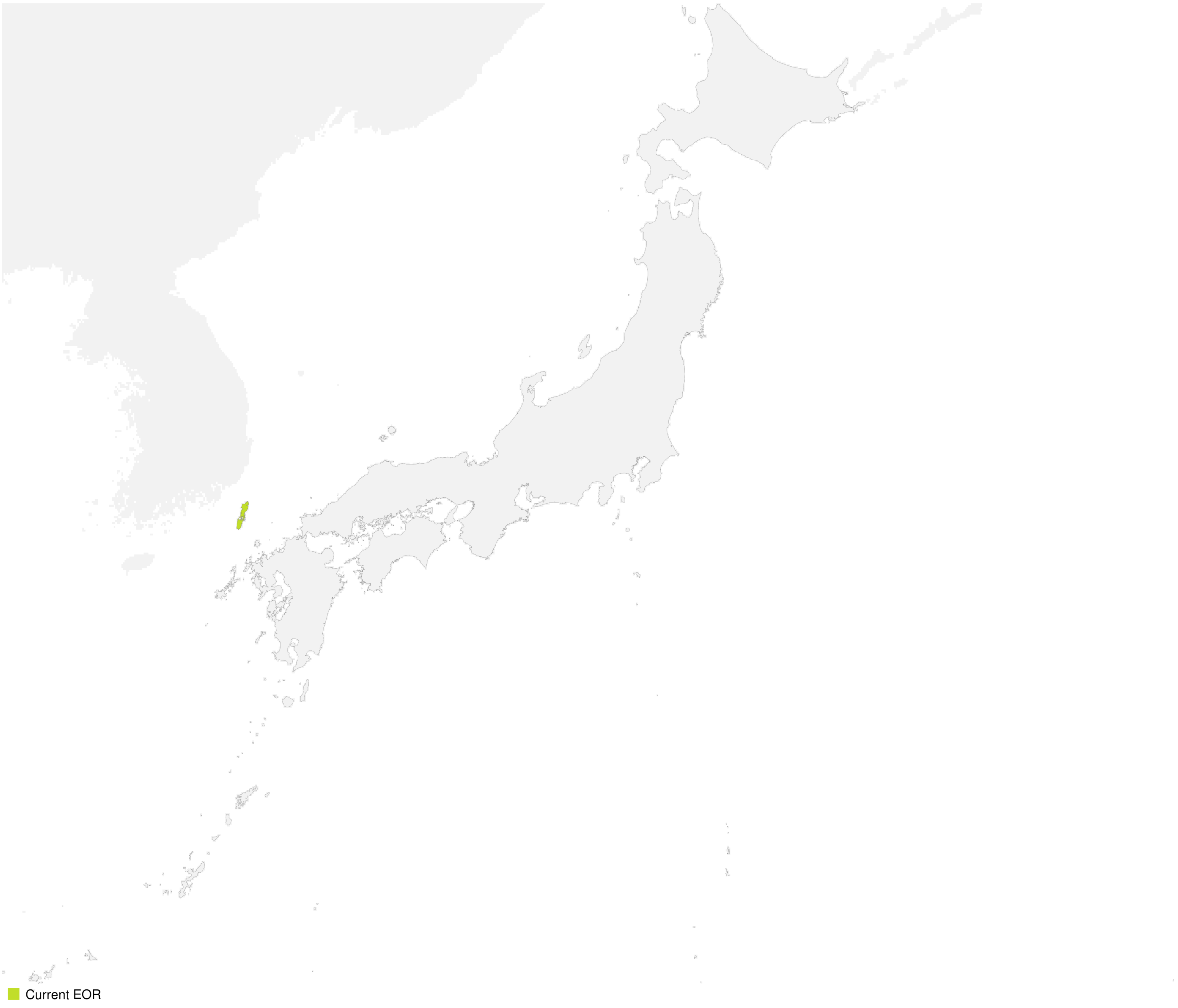

Gloydius ussuriensis

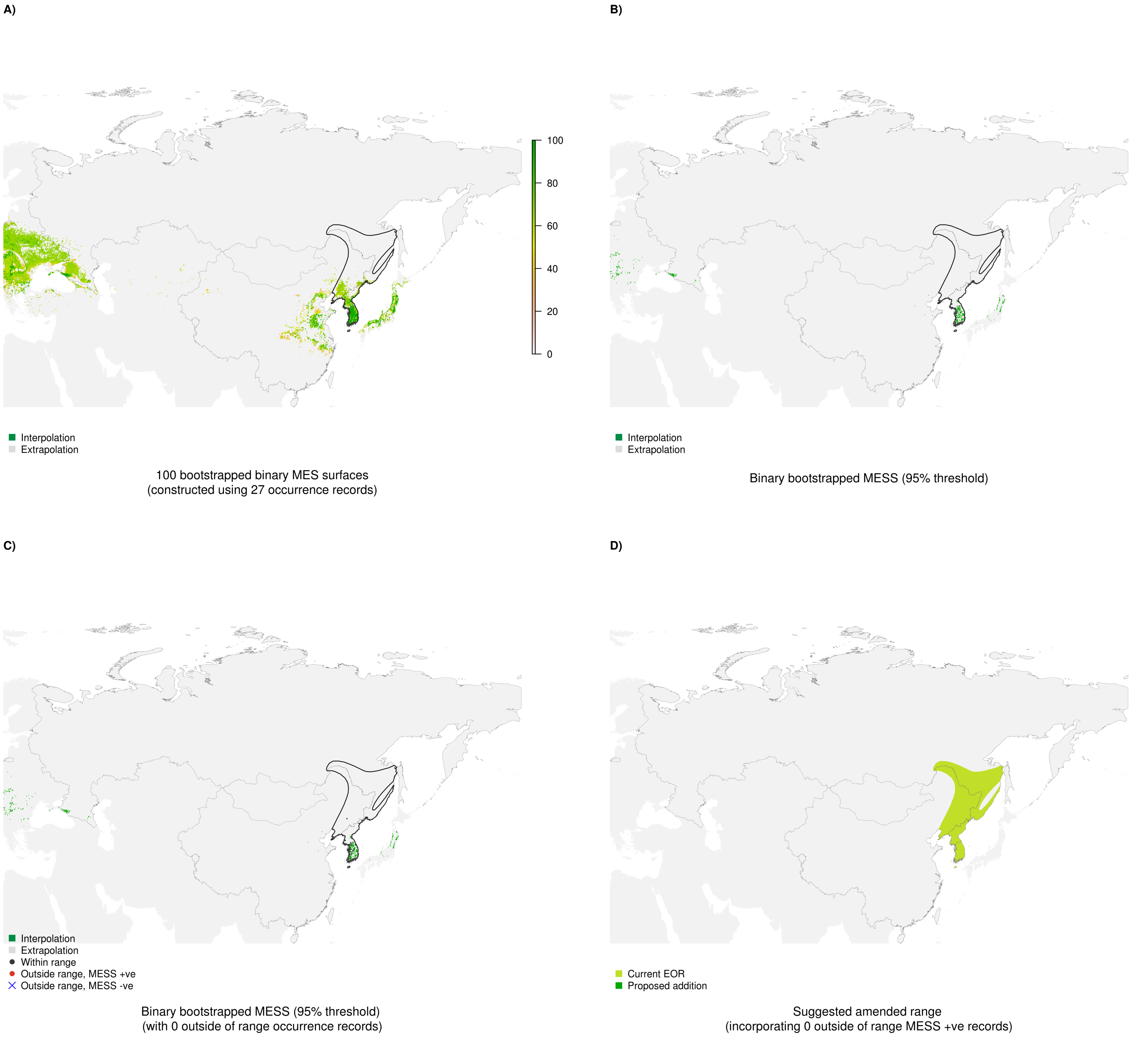

Hemachatus haemachatus

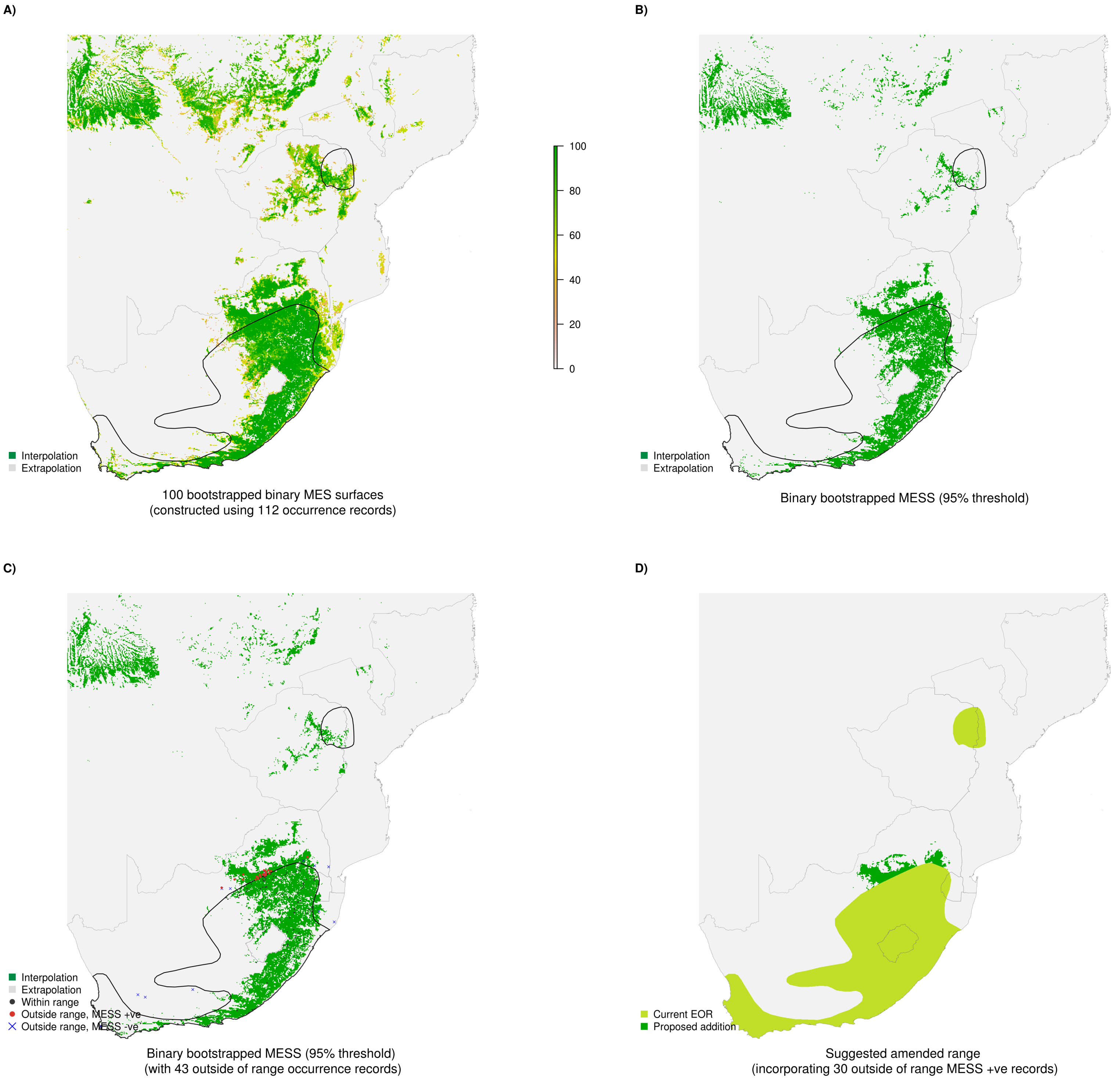

Himalayophis tibetanus

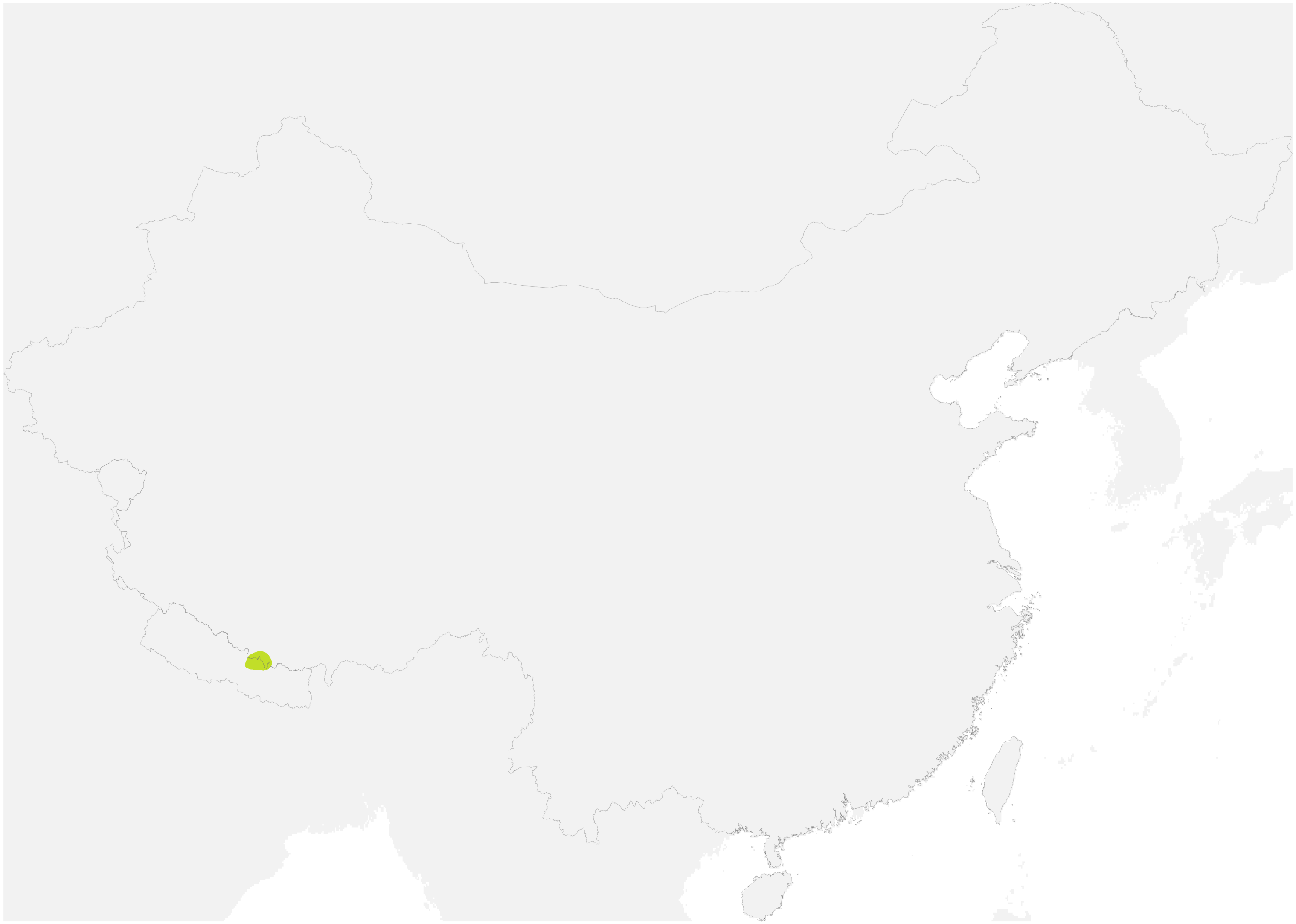

Current EOR

Hoplocephalus bitorquatus

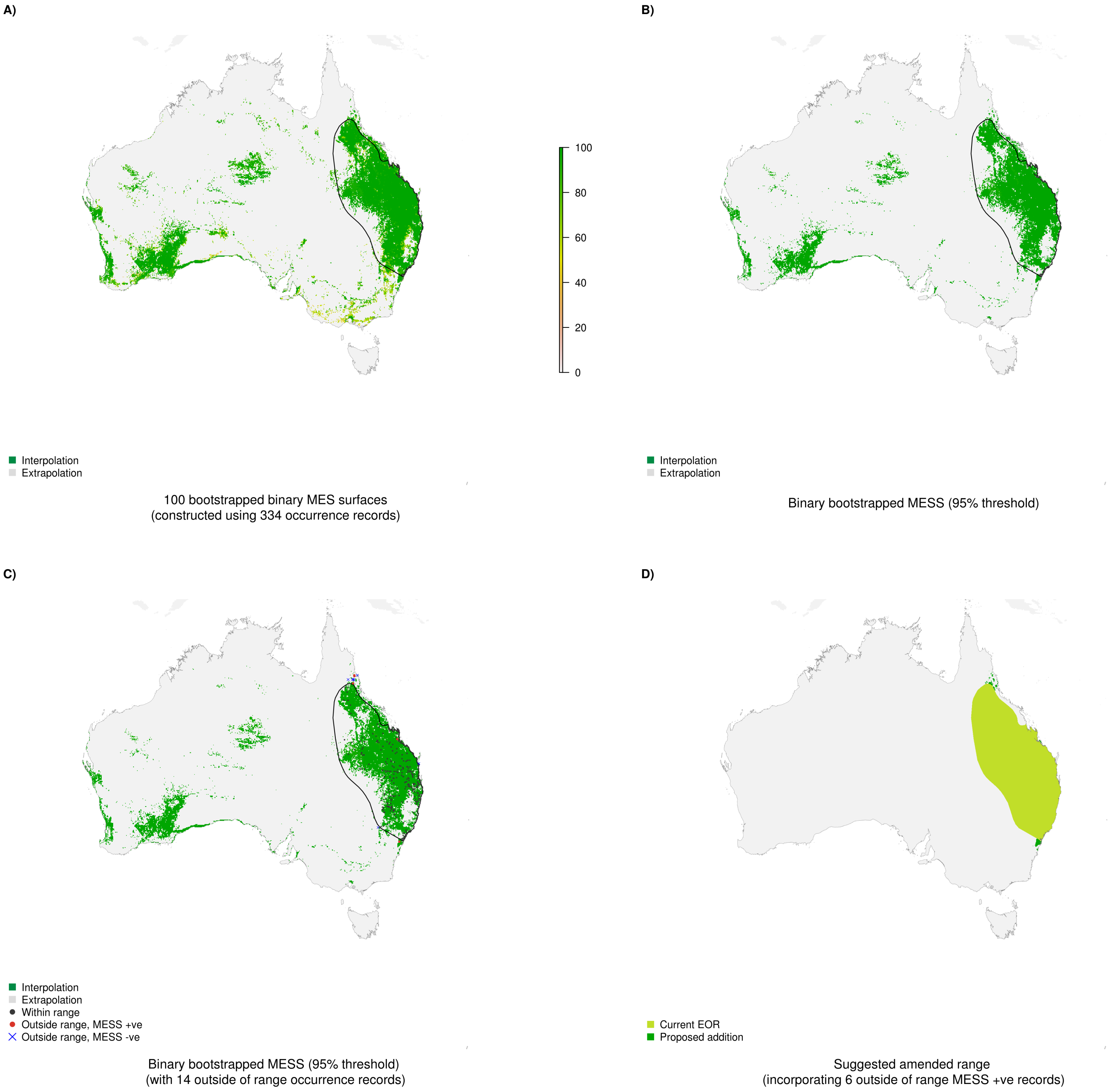

Hoplocephalus bungaroides

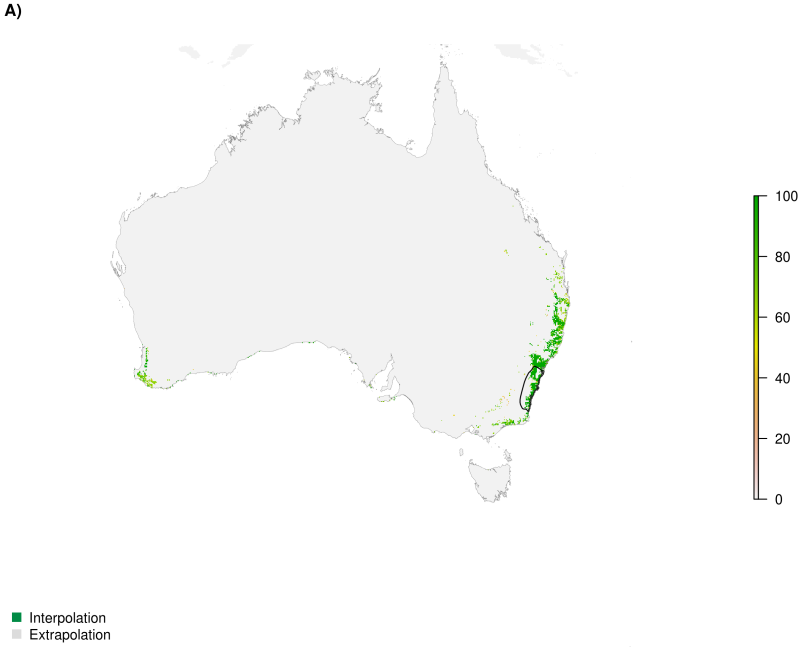

100 bootstrapped binary MES surfaces
(constructed using 104 occurrence records)

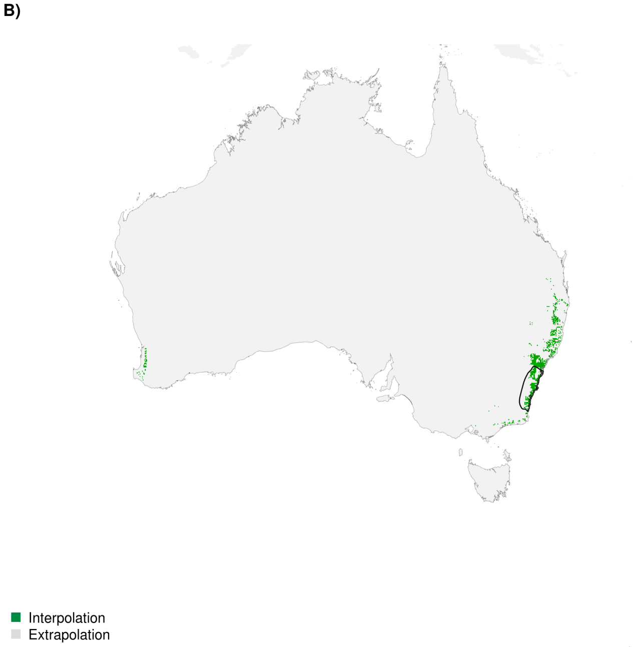

Binary bootstrapped MESS (95% threshold)

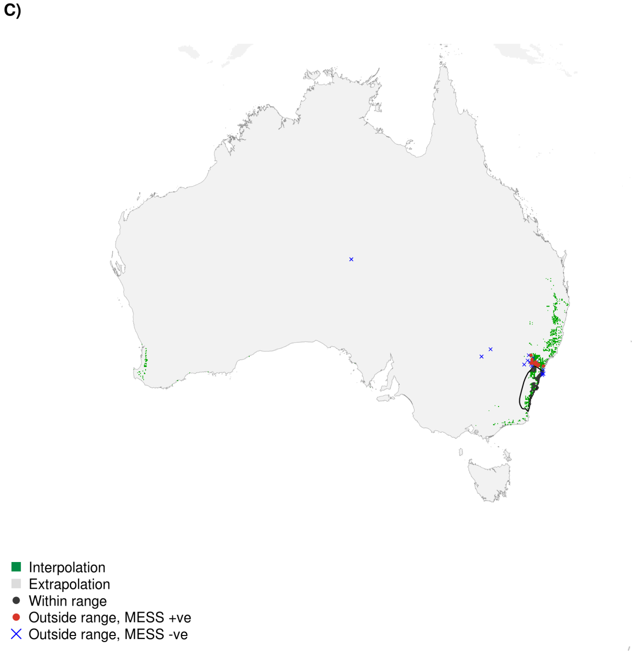

Binary bootstrapped MESS (95% threshold)
(with 39 outside of range occurrence records)

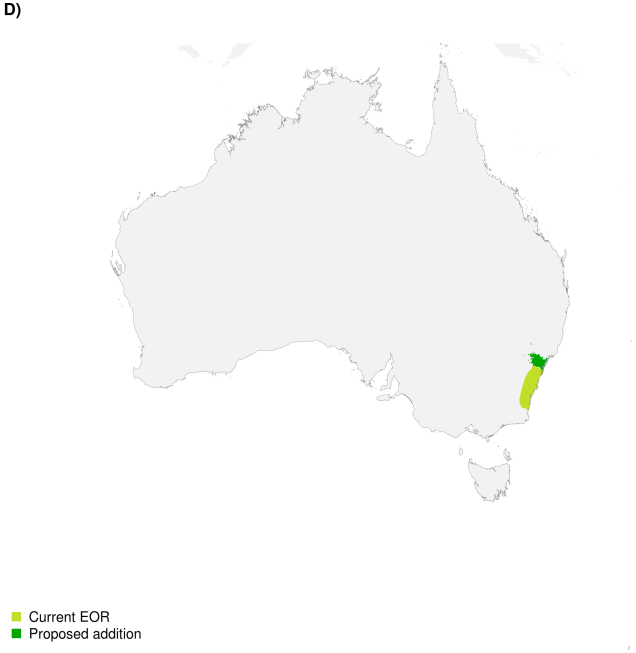

Suggested amended range
(incorporating 15 outside of range MESS +ve records)

Hoplocephalus stephensii

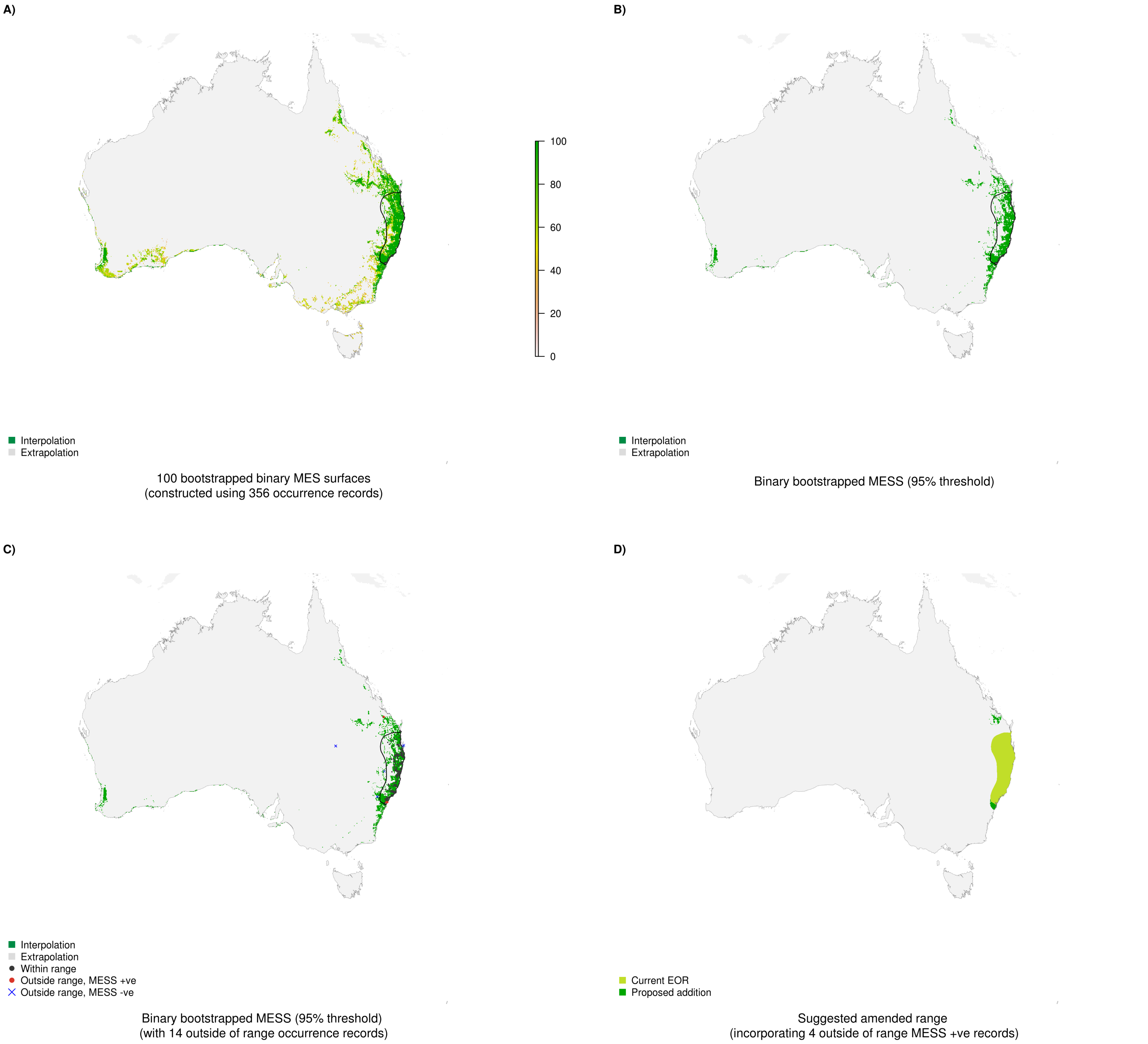

Hypnale hypnale

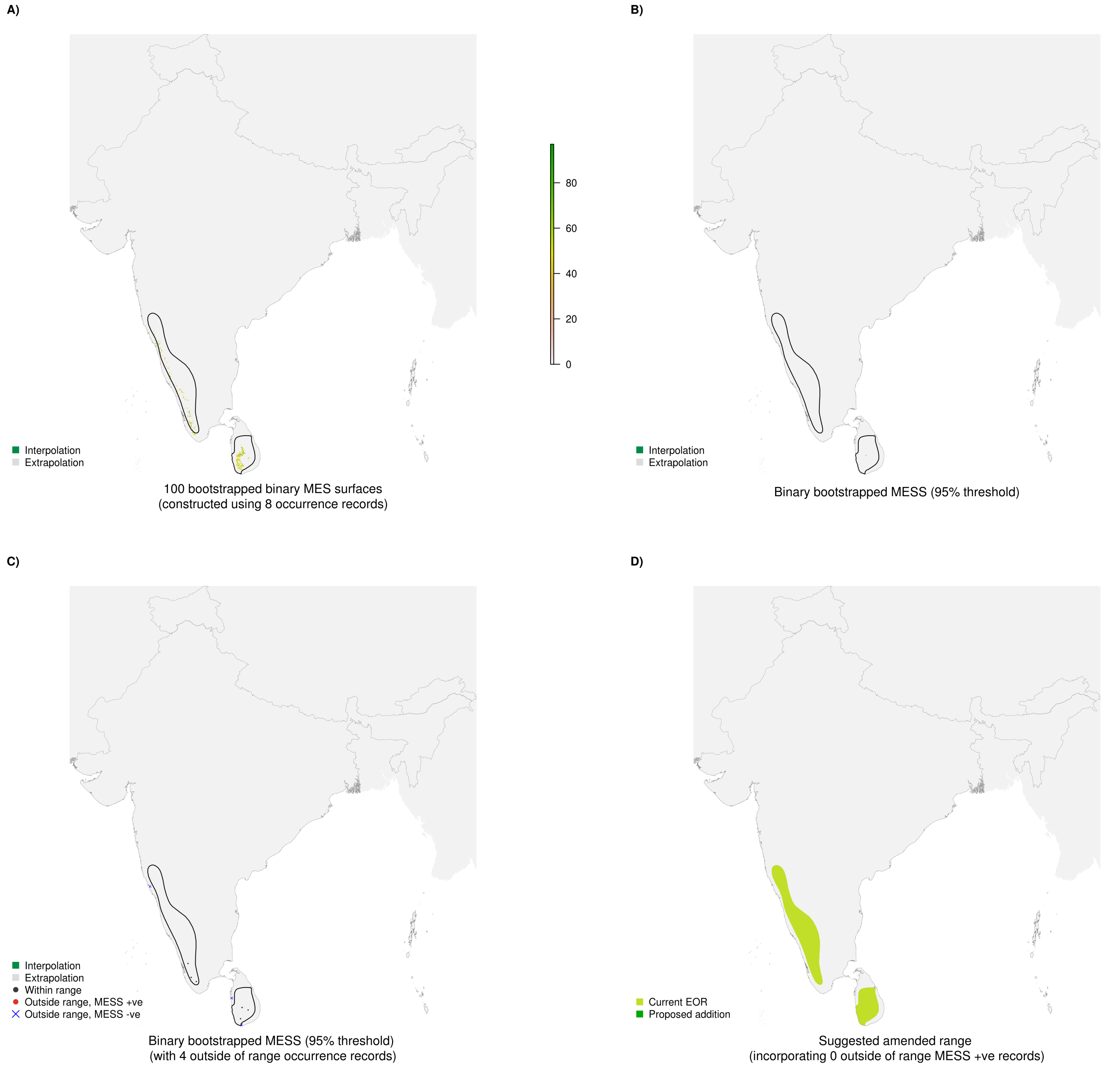

Hypnale nepa

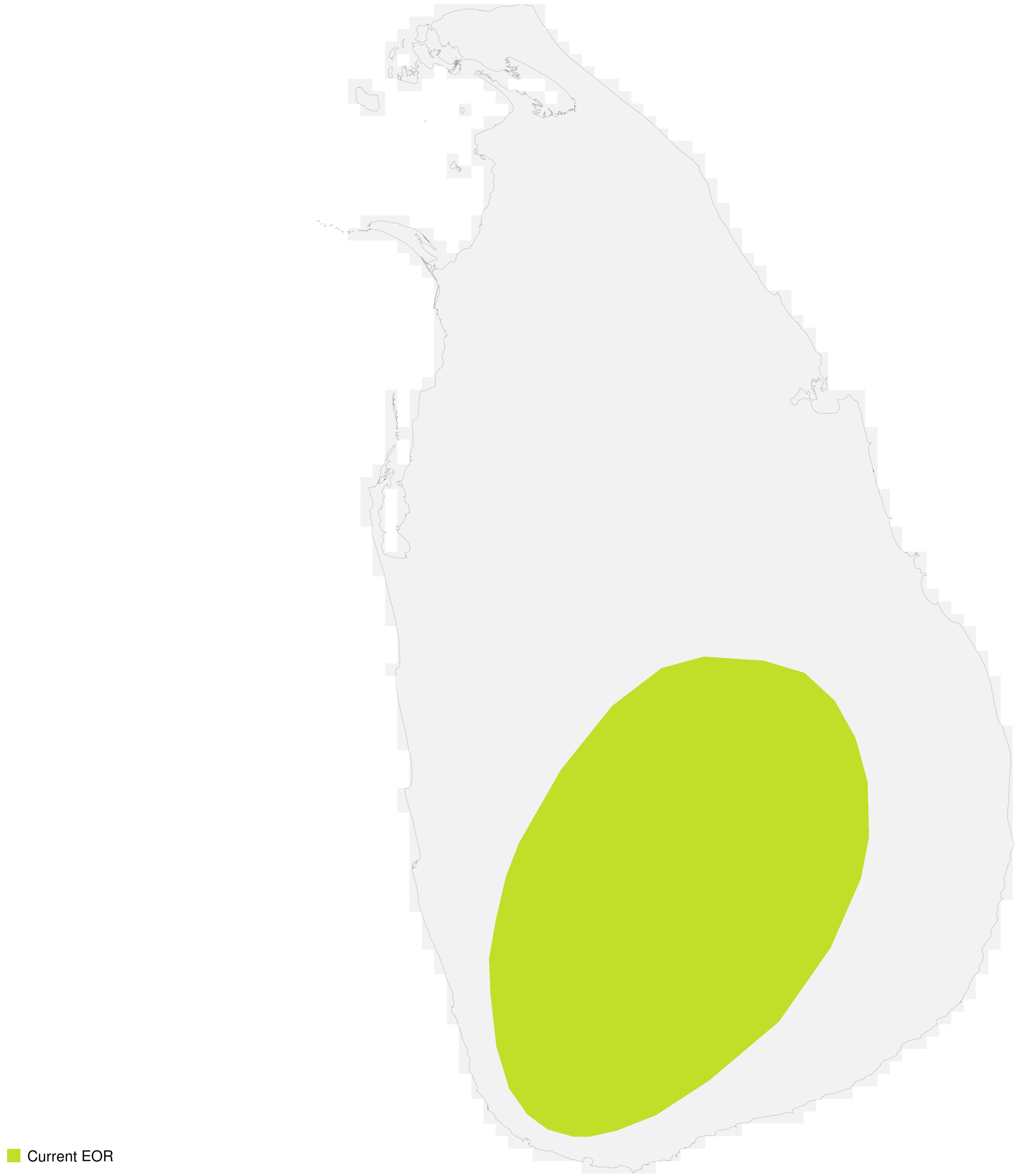

Lachesis acrochorda

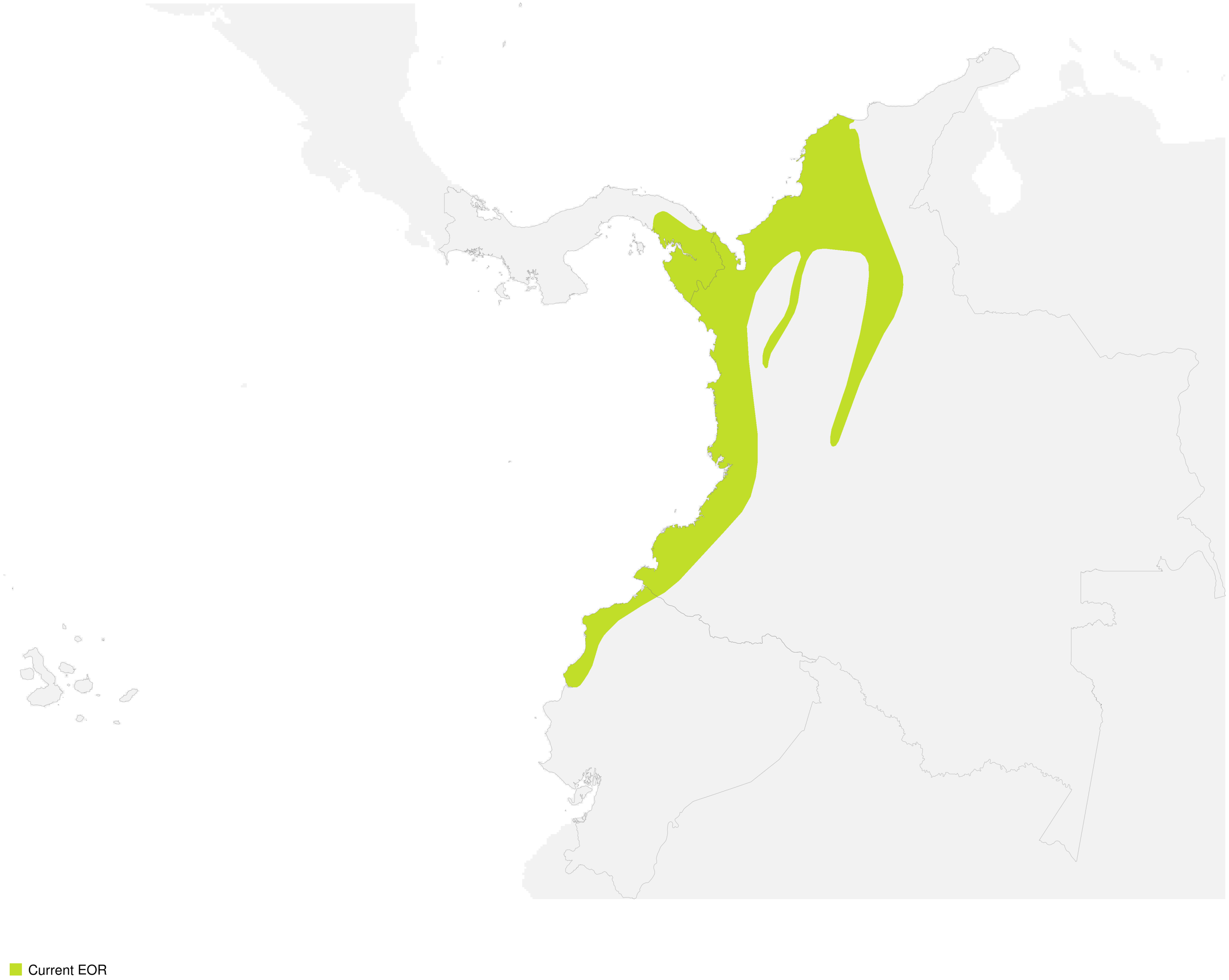

Lachesis melanocephala

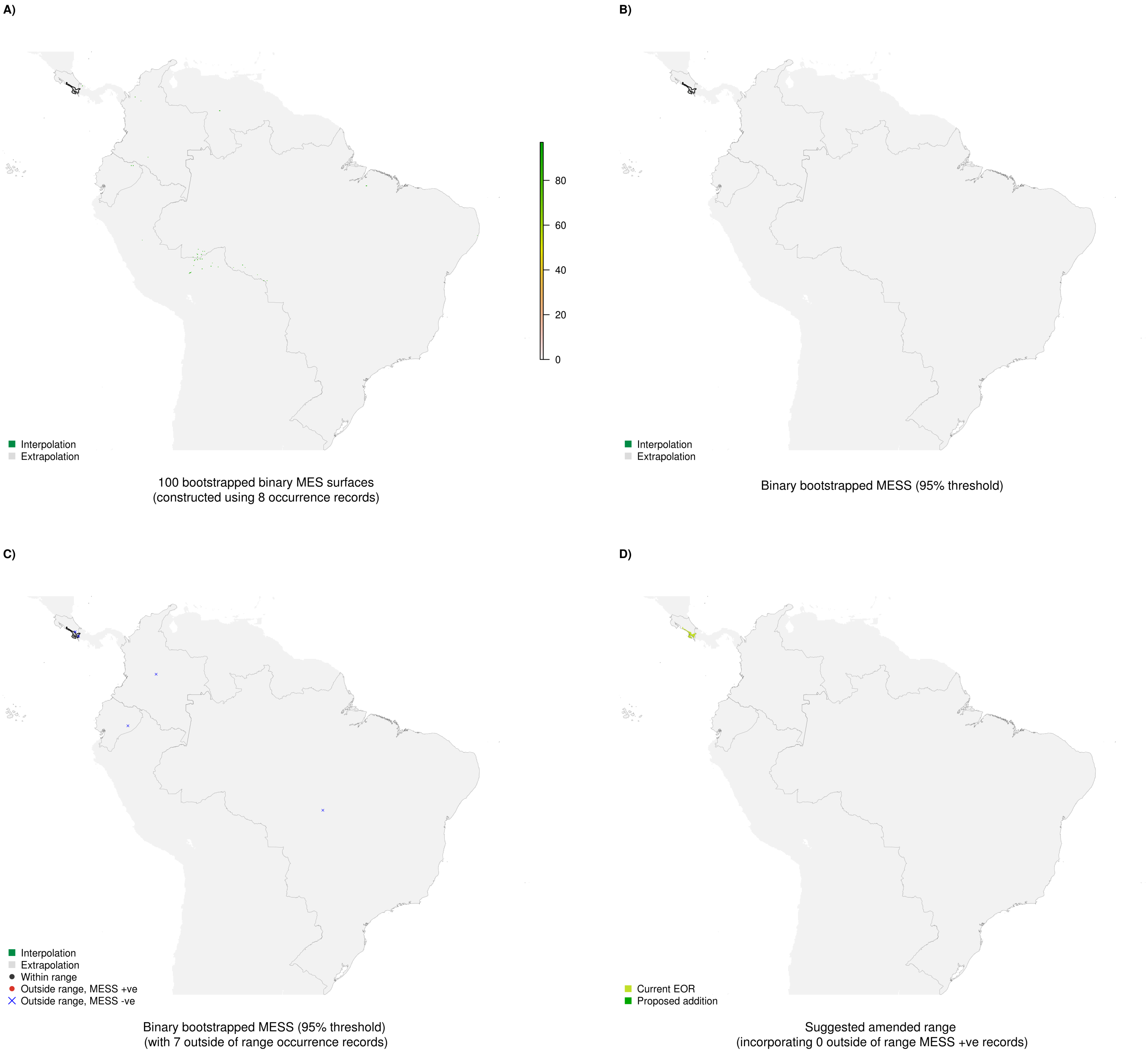

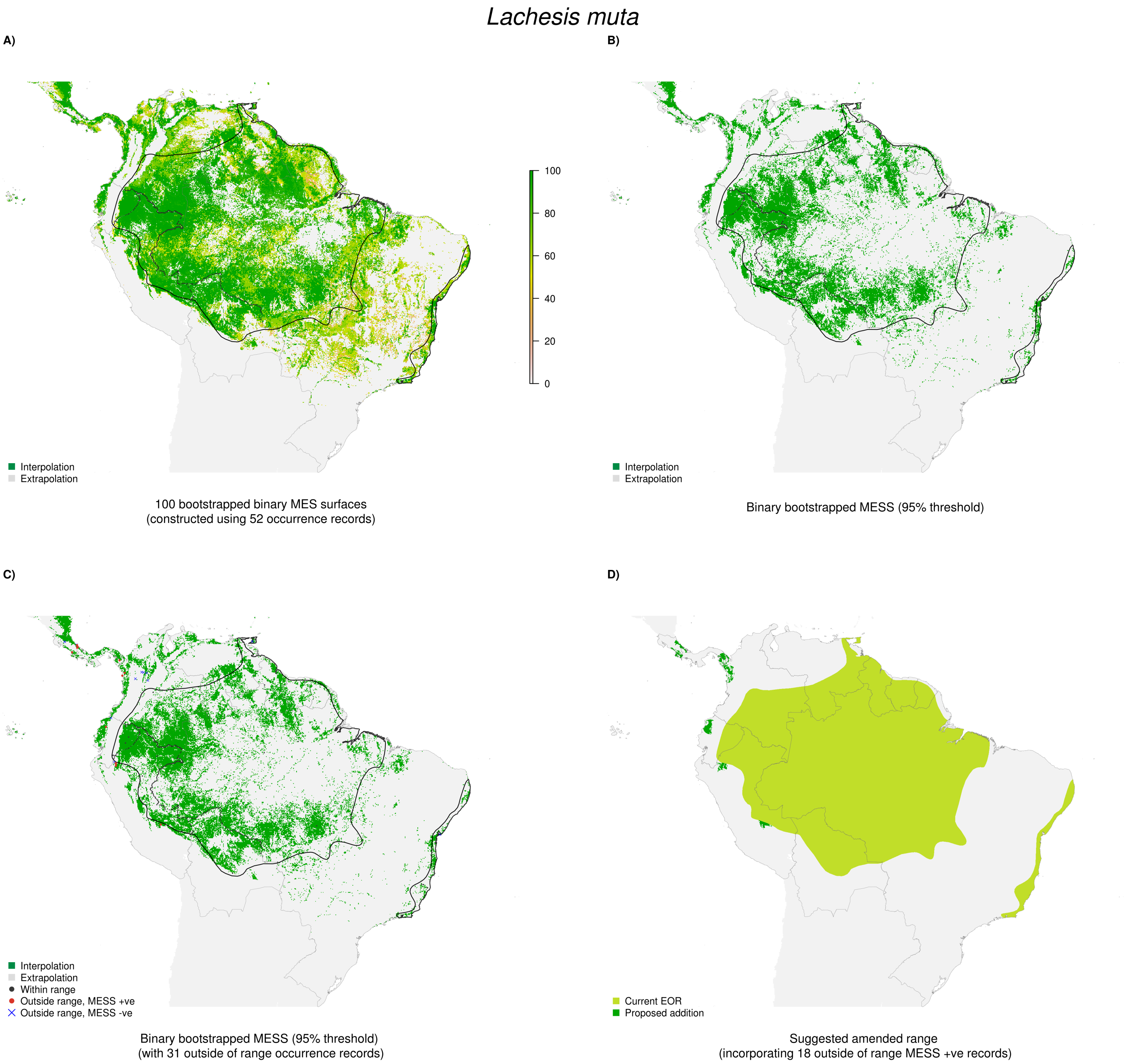

Lachesis muta
A)
100
80
60
40
20
0
Interpolation
Extrapolation
100 bootstrapped binary MES surfaces
(constructed using 52 occurrence records)
B)
Interpolation
Extrapolation
Binary bootstrapped MESS (95% threshold)
C)
Interpolation
Extrapolation
Within range
Outside range, MESS +ve
Outside range, MESS -ve
Binary bootstrapped MESS (95% threshold)
(with 31 outside of range occurrence records)
D)
Current EOR
Proposed addition
Suggested amended range
(incorporating 18 outside of range MESS +ve records)

Lachesis stenophrys

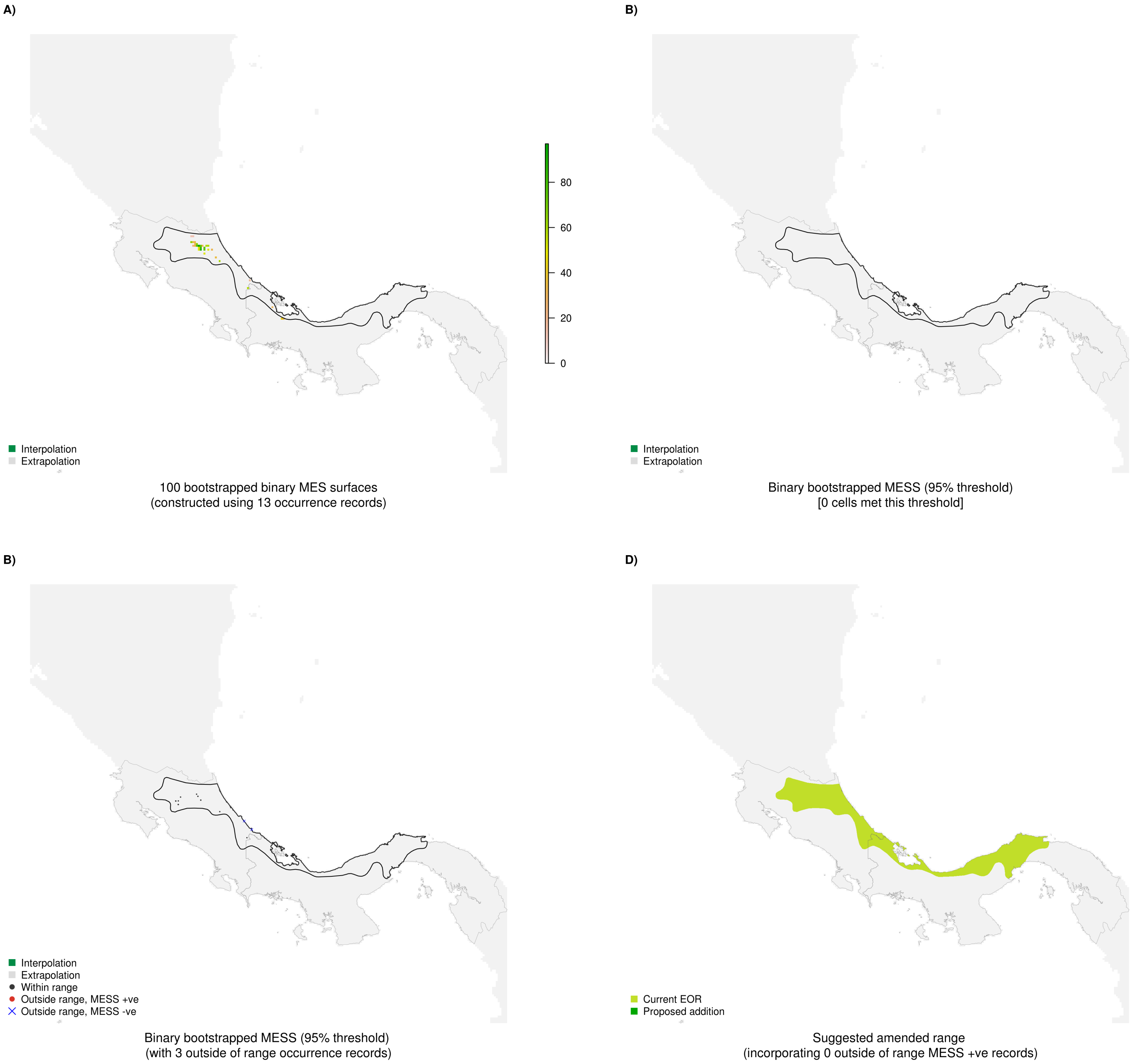

Macrovipera lebetina

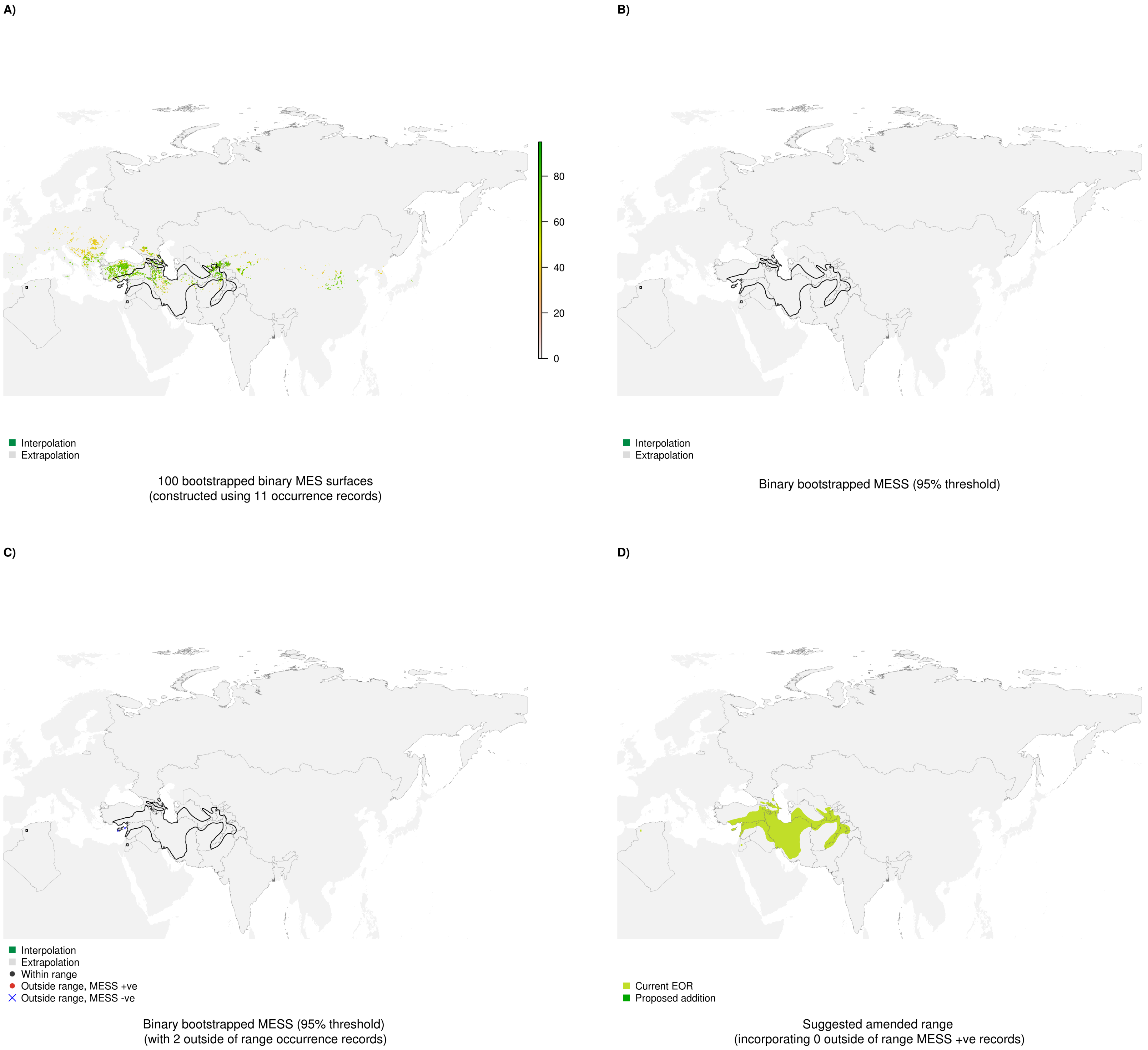

Macrovipera schweizeri

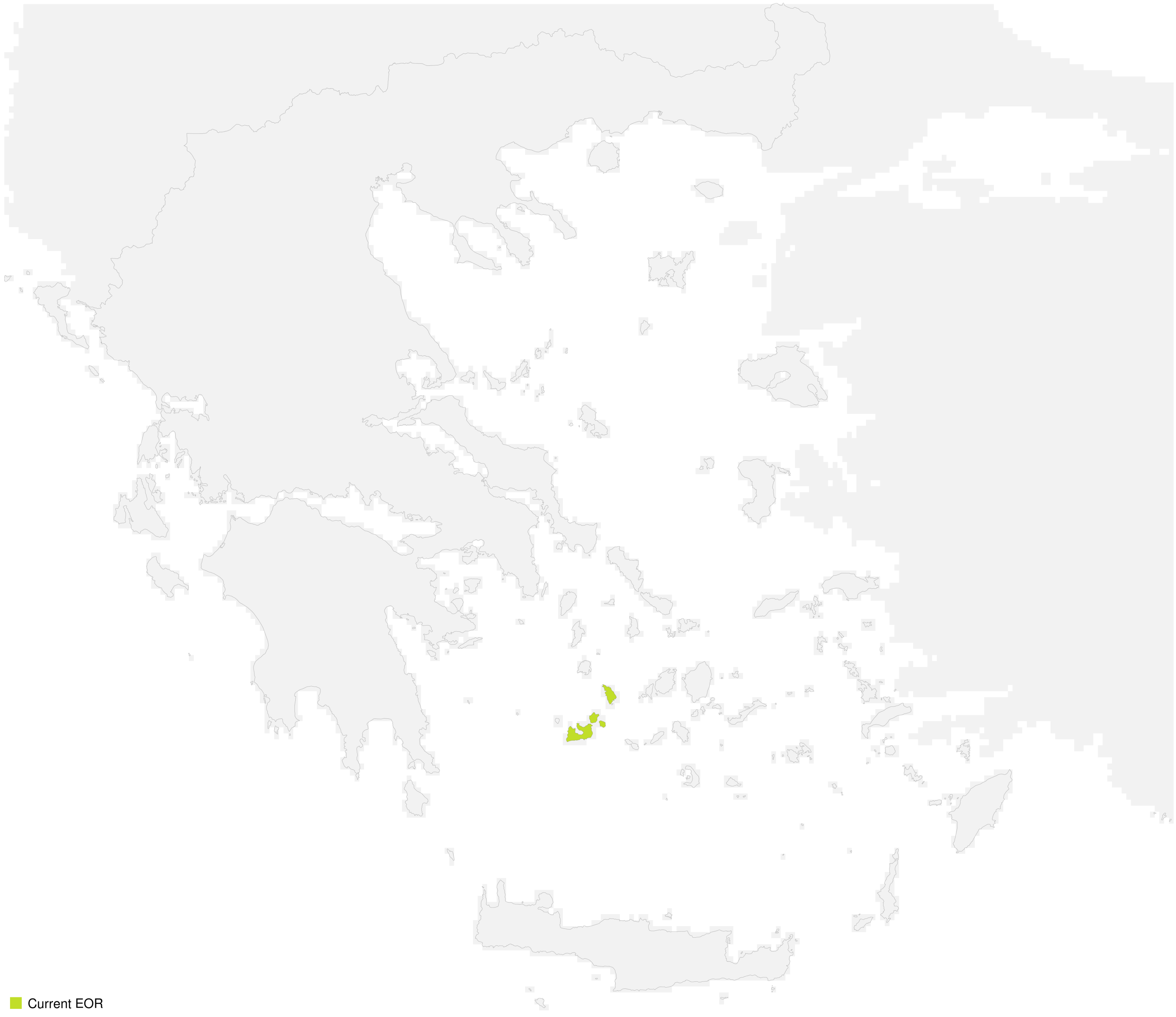

Micropechis ikaheka

A)

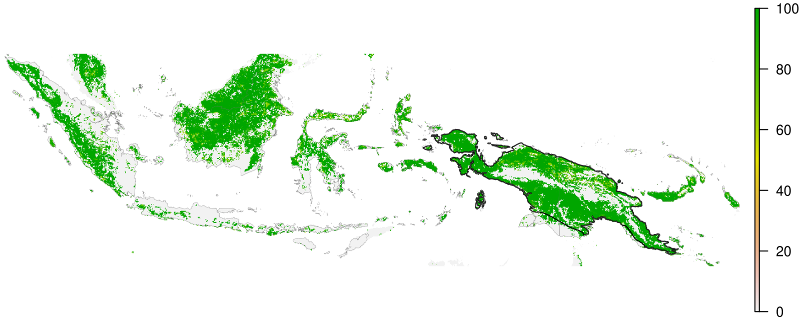

100 bootstrapped binary MES surfaces
(constructed using 95 occurrence records)

B)

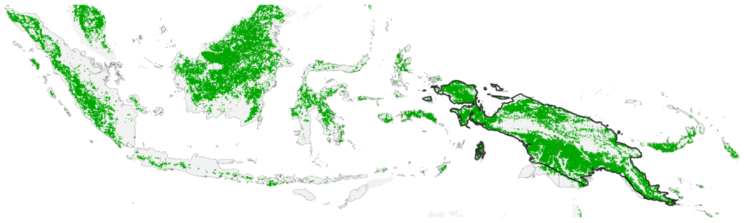

Binary bootstrapped MESS (95% threshold)

C)

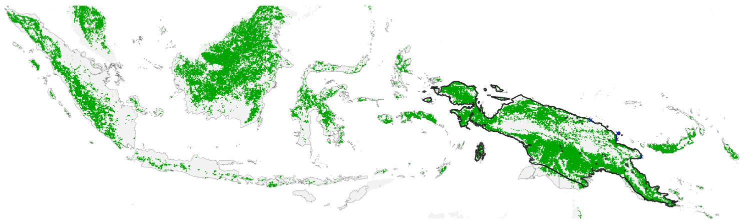

Binary bootstrapped MESS (95% threshold)
(with 6 outside of range occurrence records)

D)

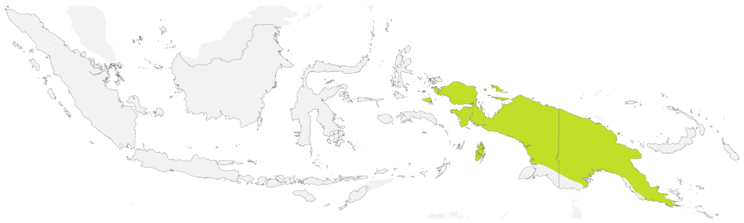

Suggested amended range
(incorporating 0 outside of range MESS +ve records)

Micruroides euryxanthus

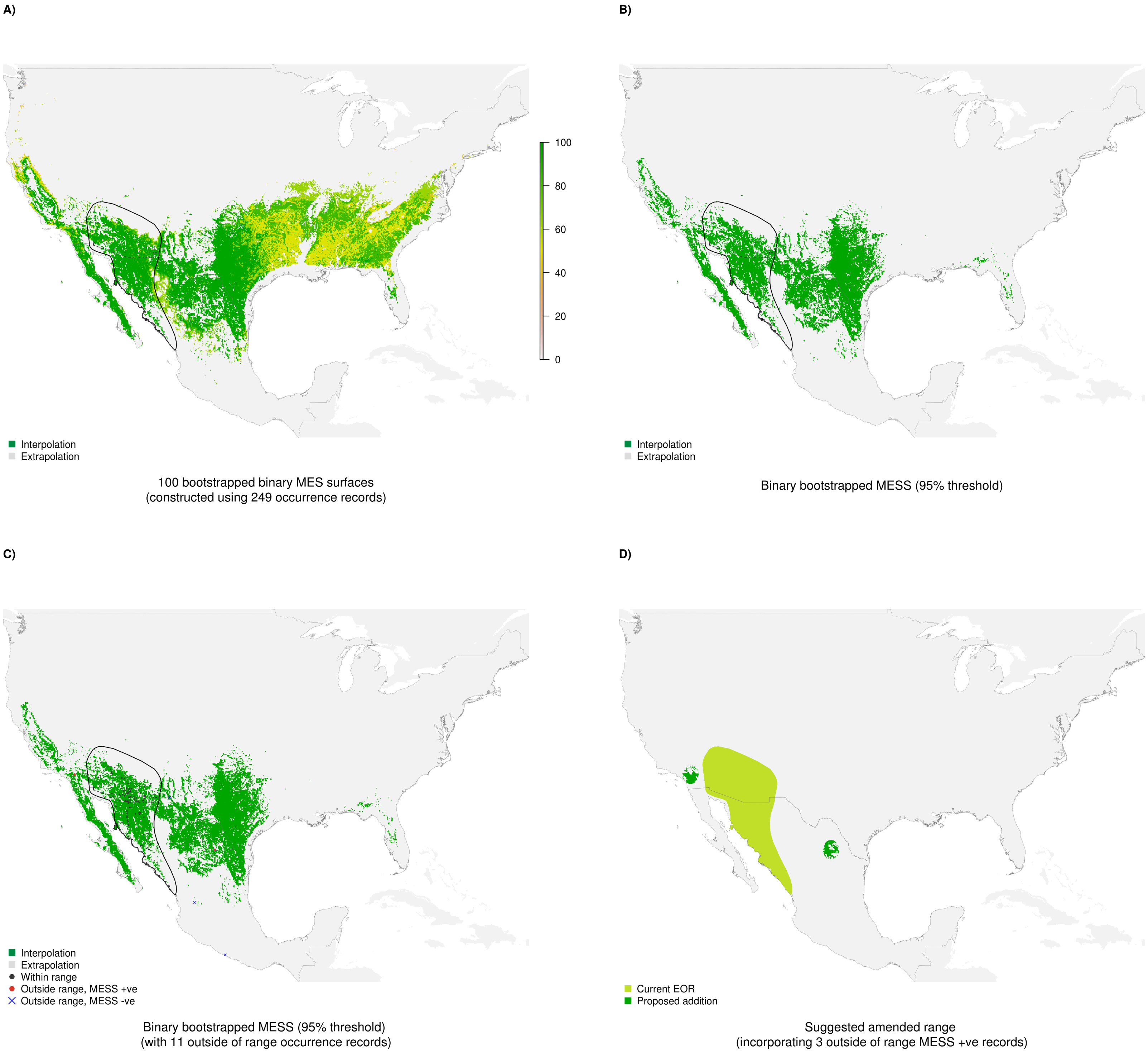

Micrurus circinalis

A)

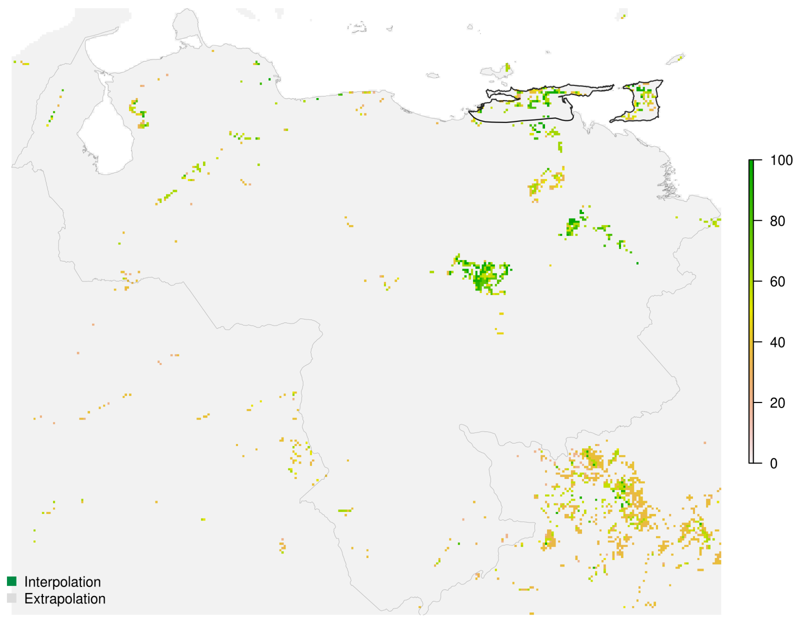

100 bootstrapped binary MES surfaces
(constructed using 10 occurrence records)

B)

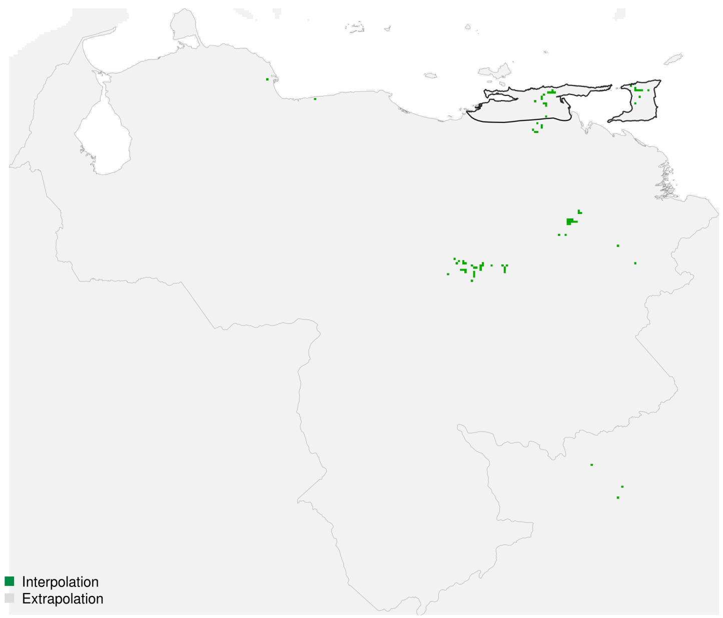

Binary bootstrapped MESS (95% threshold)

C)

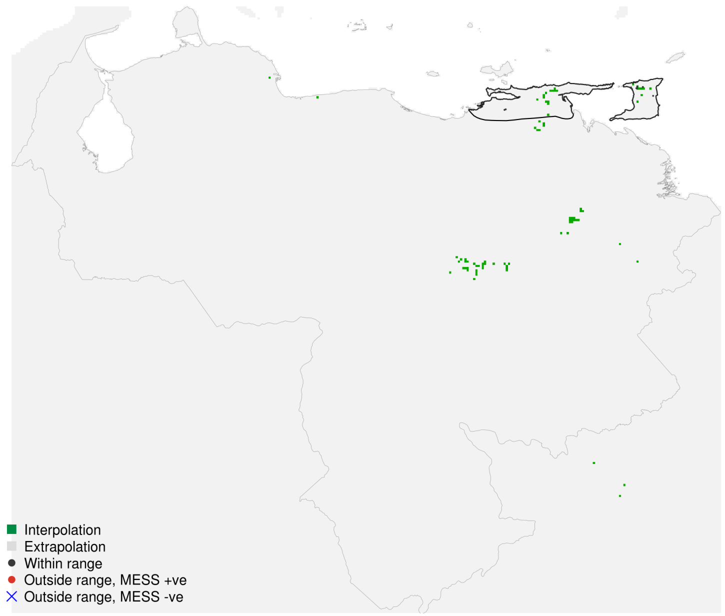

Binary bootstrapped MESS (95% threshold)
(with 0 outside of range occurrence records)

D)

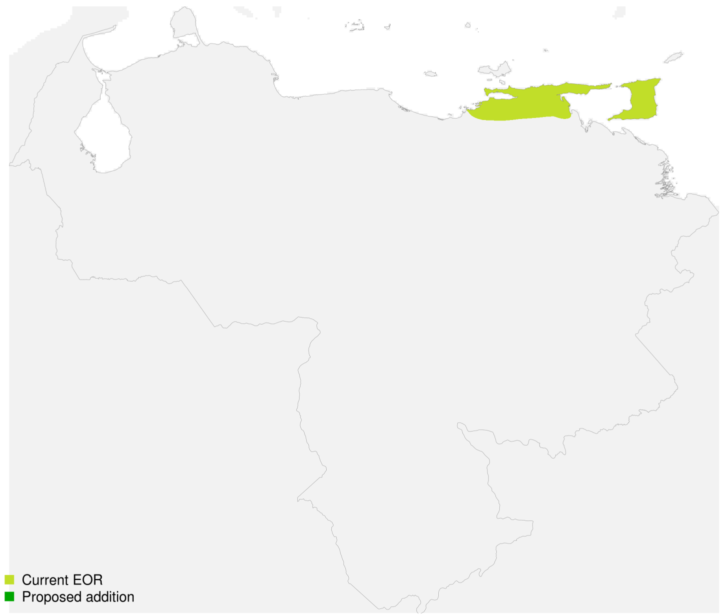

Suggested amended range
(incorporating 0 outside of range MESS +ve records)

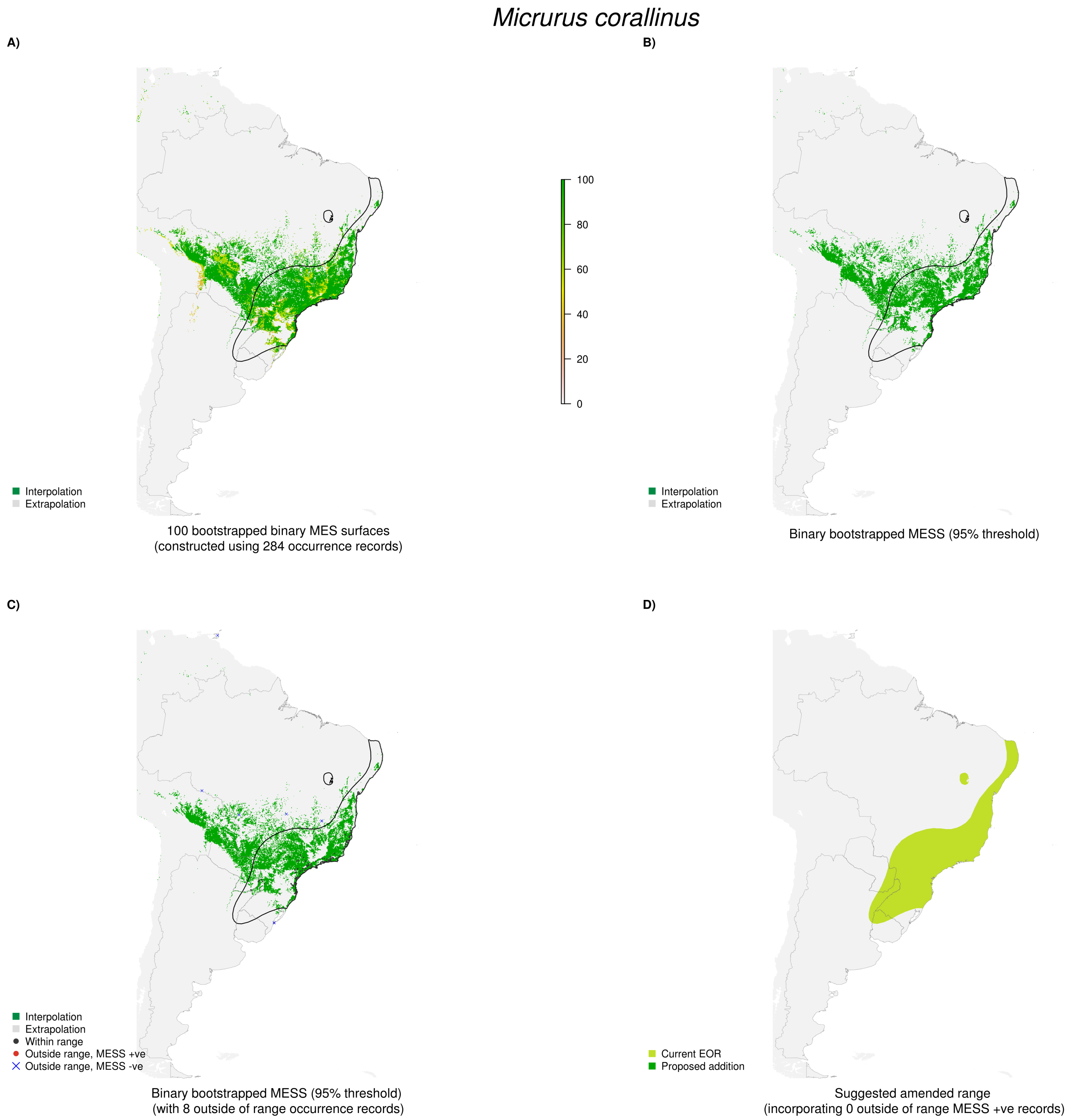
Micrurus corallinus
A)
100
80
60
40
20
0
Interpolation
Extrapolation
100 bootstrapped binary MES surfaces
(constructed using 284 occurrence records)
B)
Interpolation
Extrapolation
Binary bootstrapped MESS (95% threshold)
C)
Interpolation
Extrapolation
Within range
Outside range, MESS +ve
Outside range, MESS -ve
Binary bootstrapped MESS (95% threshold)
(with 8 outside of range occurrence records)
D)
Current EOR
Proposed addition
Suggested amended range
(incorporating 0 outside of range MESS +ve records)

Micrurus fulvius

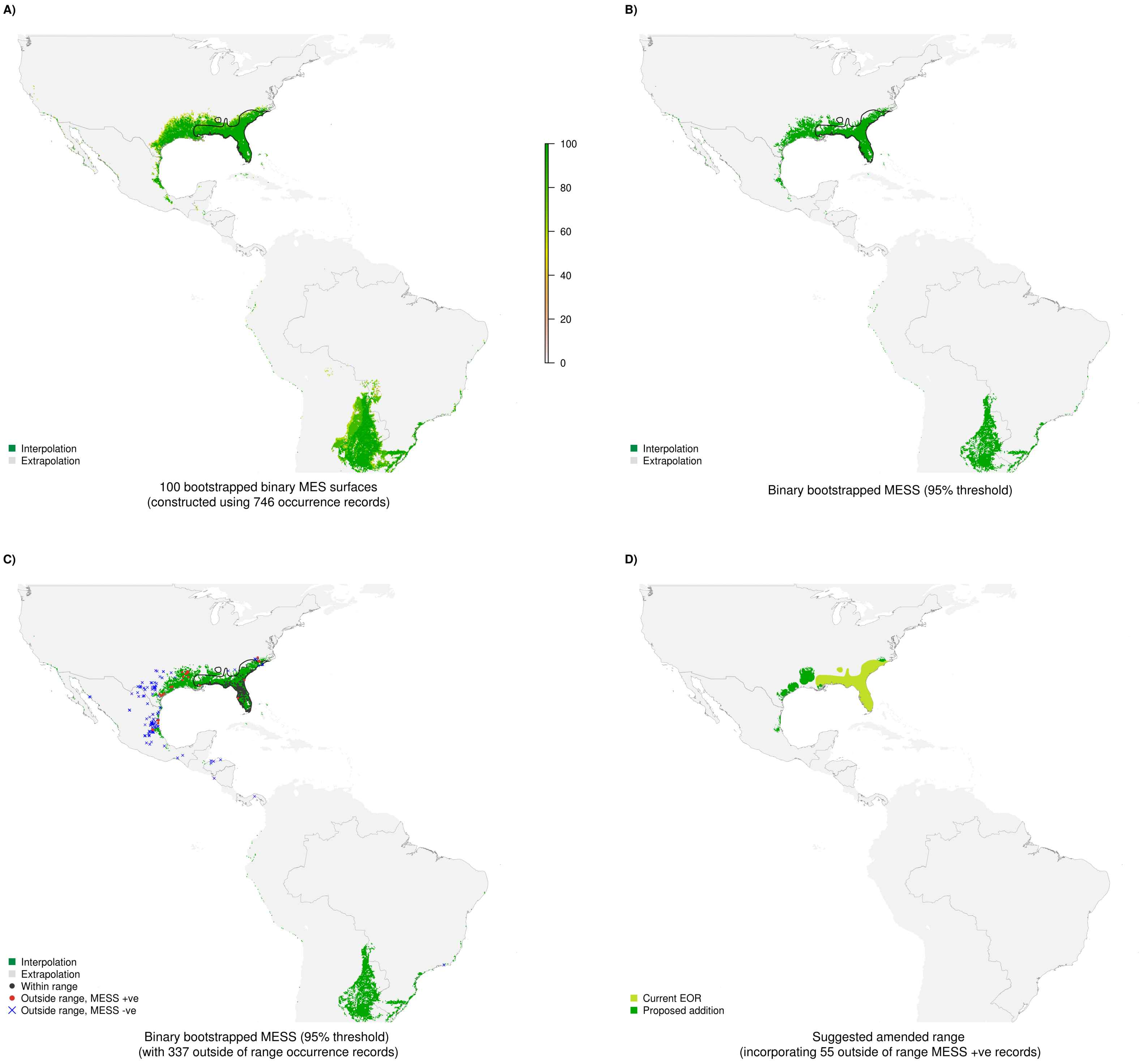

Micrurus lemniscatus

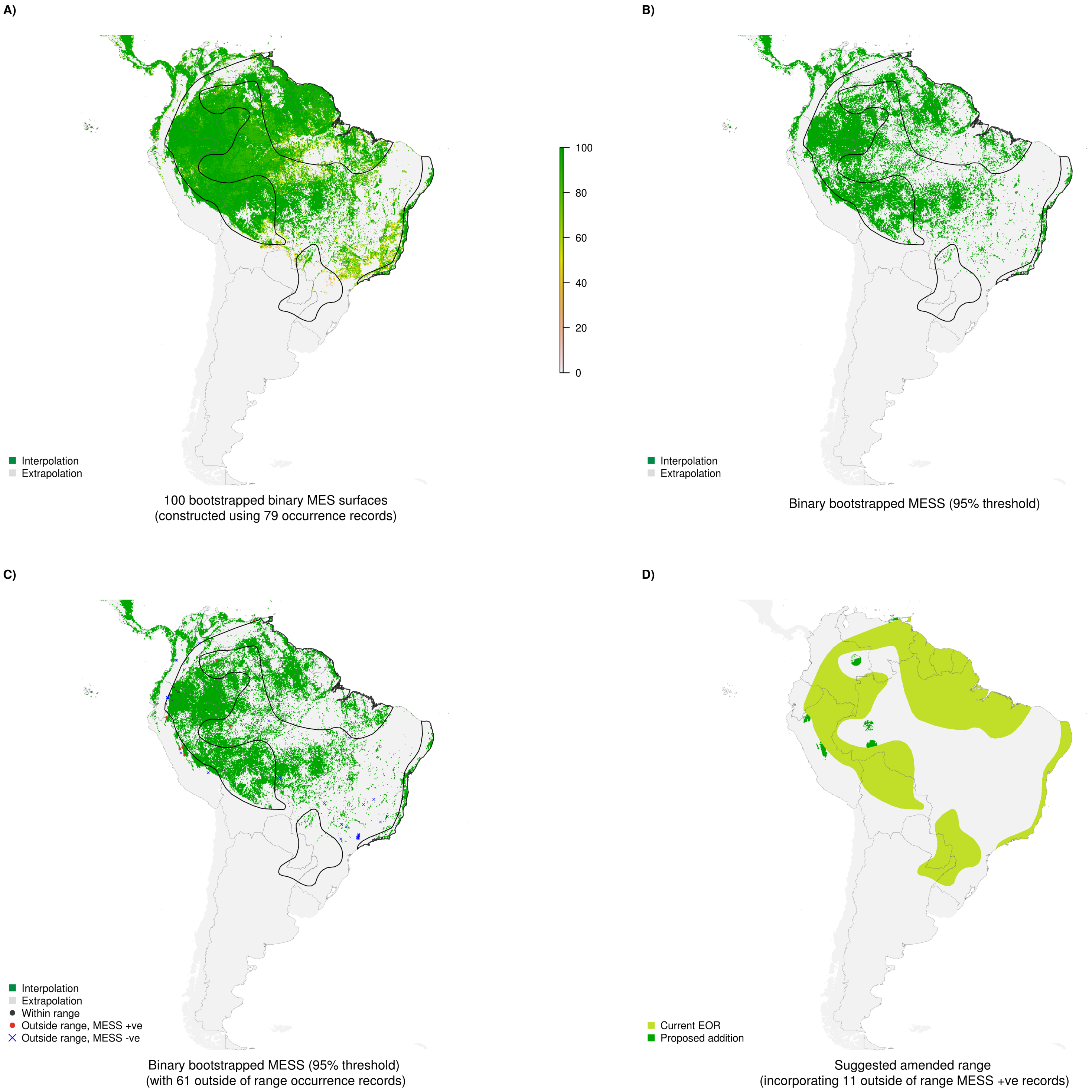

Micrurus mipartitus

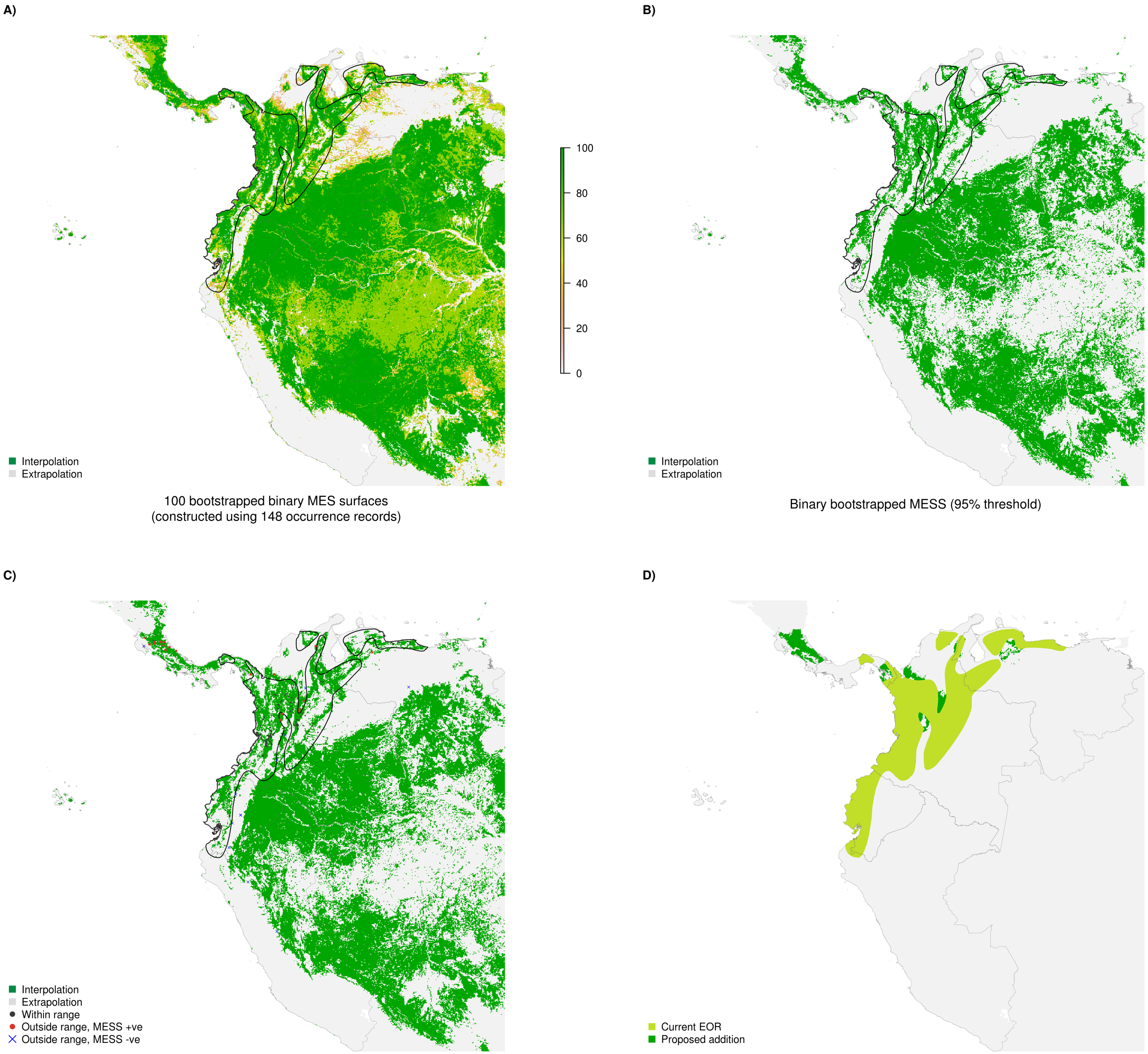

Binary bootstrapped MESS (95% threshold)
(with 62 outside of range occurrence records)

Suggested amended range
(incorporating 47 outside of range MESS +ve records)

Micrurus nigrocinctus

A)

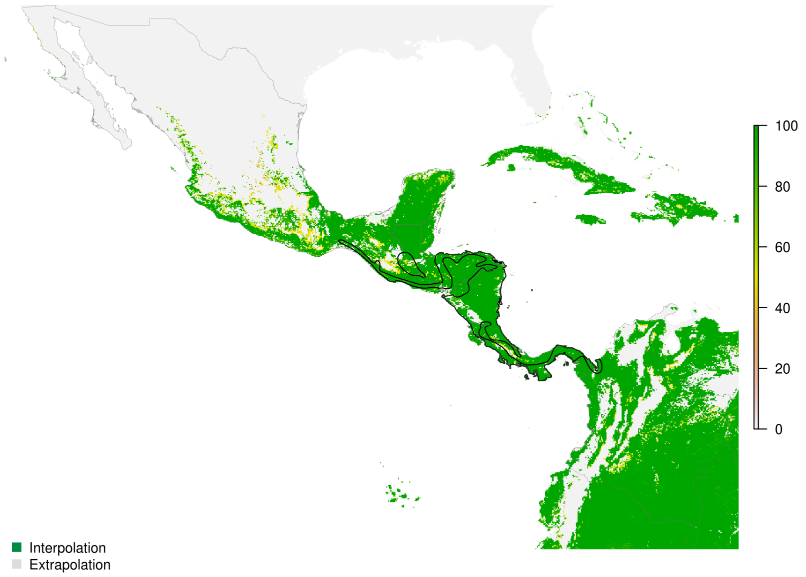

100 bootstrapped binary MES surfaces
(constructed using 223 occurrence records)

B)

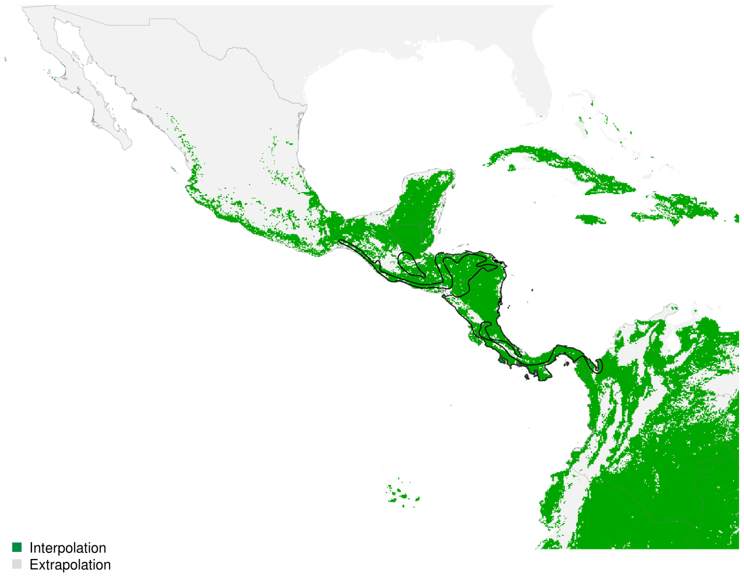

Binary bootstrapped MESS (95% threshold)

C)

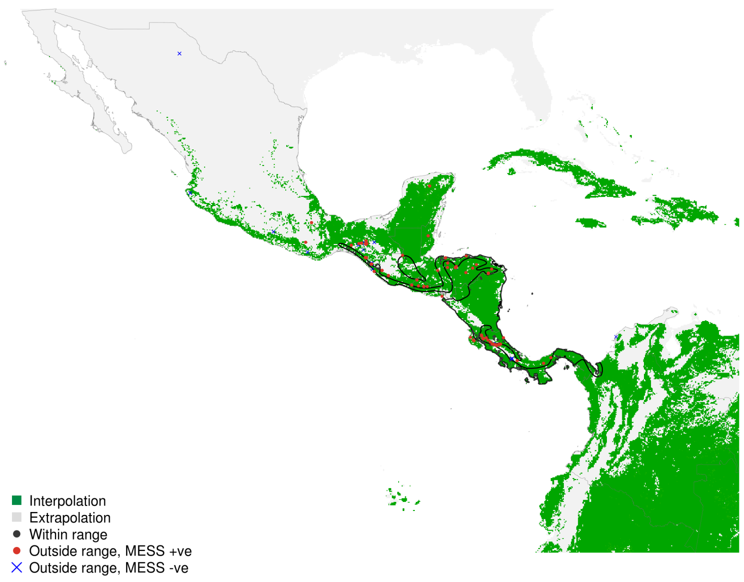

Binary bootstrapped MESS (95% threshold)
(with 200 outside of range occurrence records)

D)

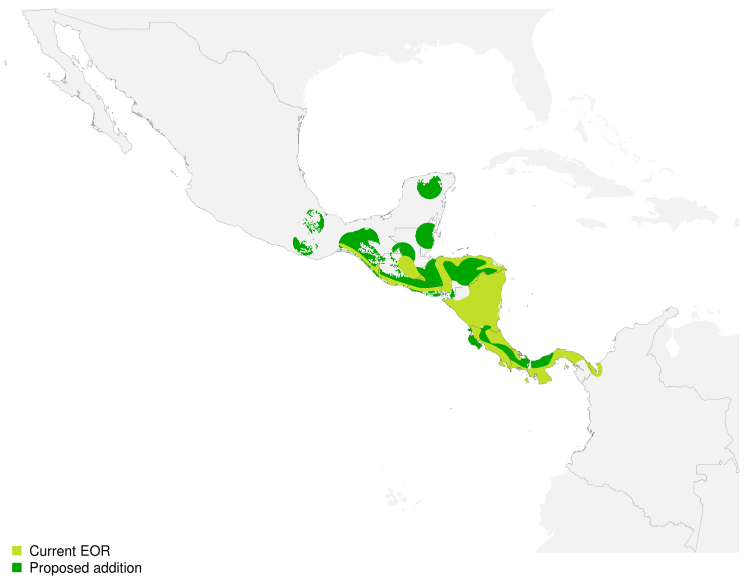

Suggested amended range
(incorporating 163 outside of range MESS +ve records)

Micrurus spixii

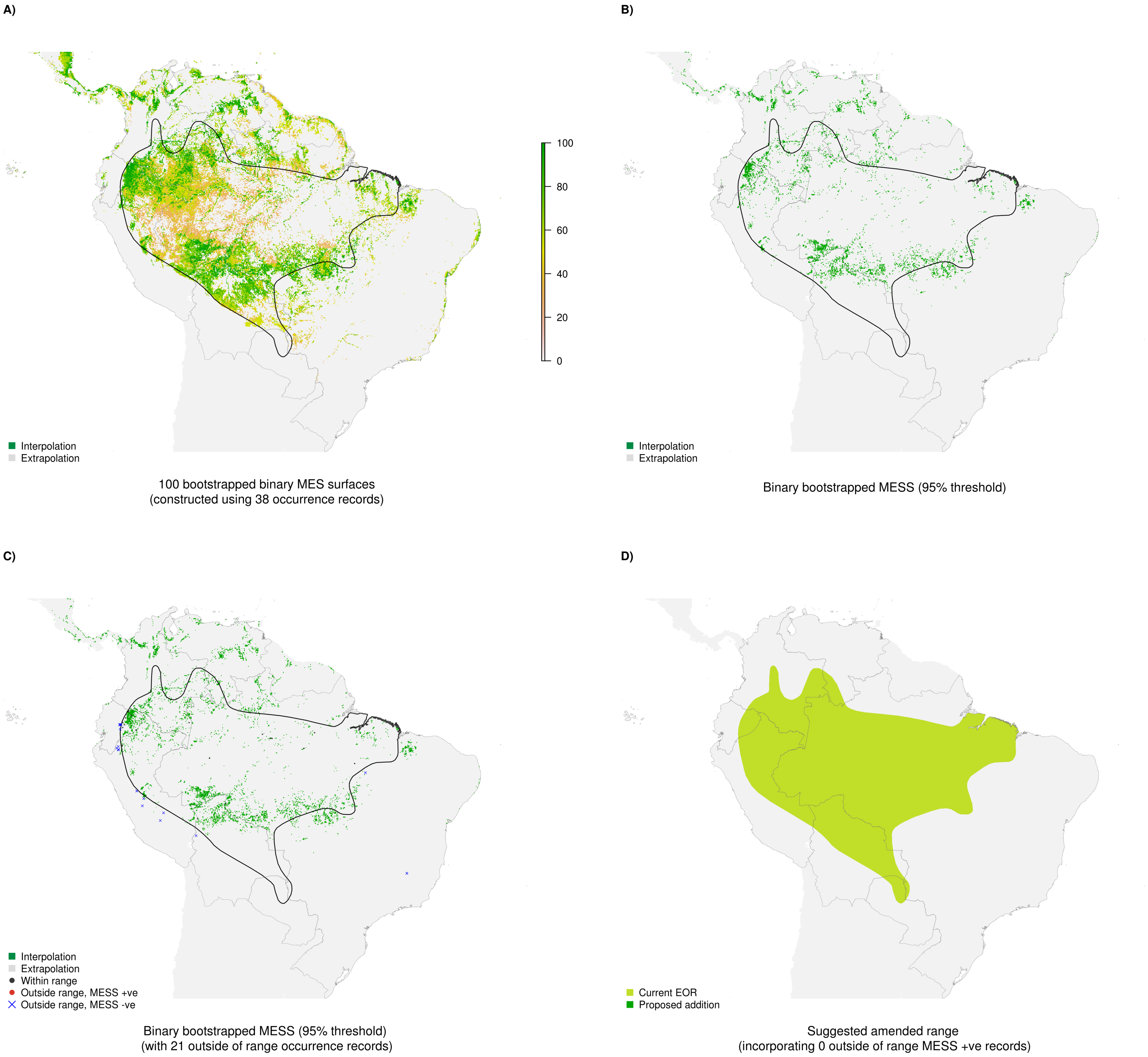

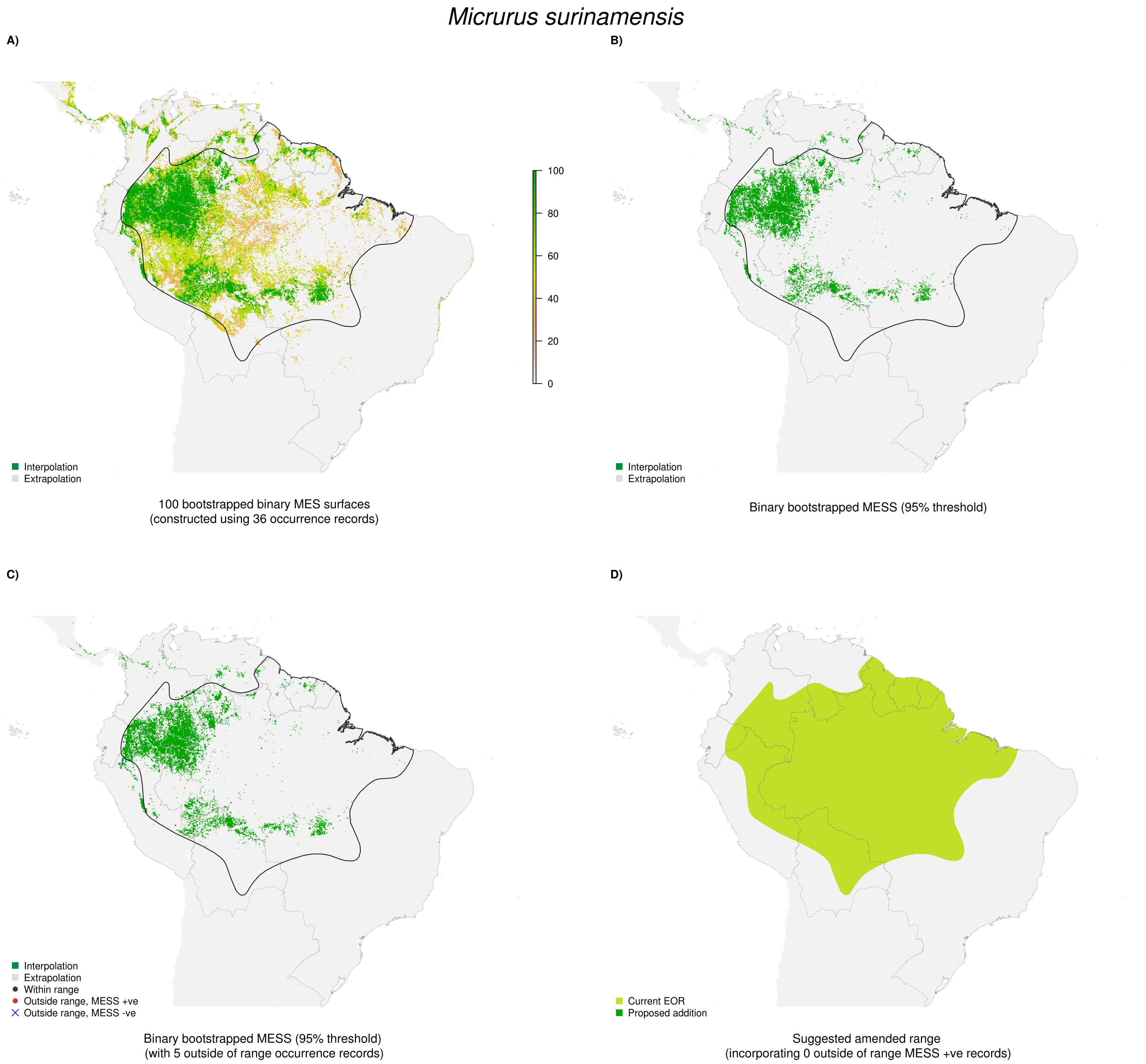
Micrurus surinamensis
A)
100
80
60
40
20
0
Interpolation
Extrapolation
100 bootstrapped binary MES surfaces
(constructed using 36 occurrence records)
B)
Interpolation
Extrapolation
Binary bootstrapped MESS (95% threshold)
C)
Interpolation
Extrapolation
Within range
Outside range, MESS +ve
Outside range, MESS -ve
Binary bootstrapped MESS (95% threshold)
(with 5 outside of range occurrence records)
D)
Current EOR
Proposed addition
Suggested amended range
(incorporating 0 outside of range MESS +ve records)

Micrurus tener

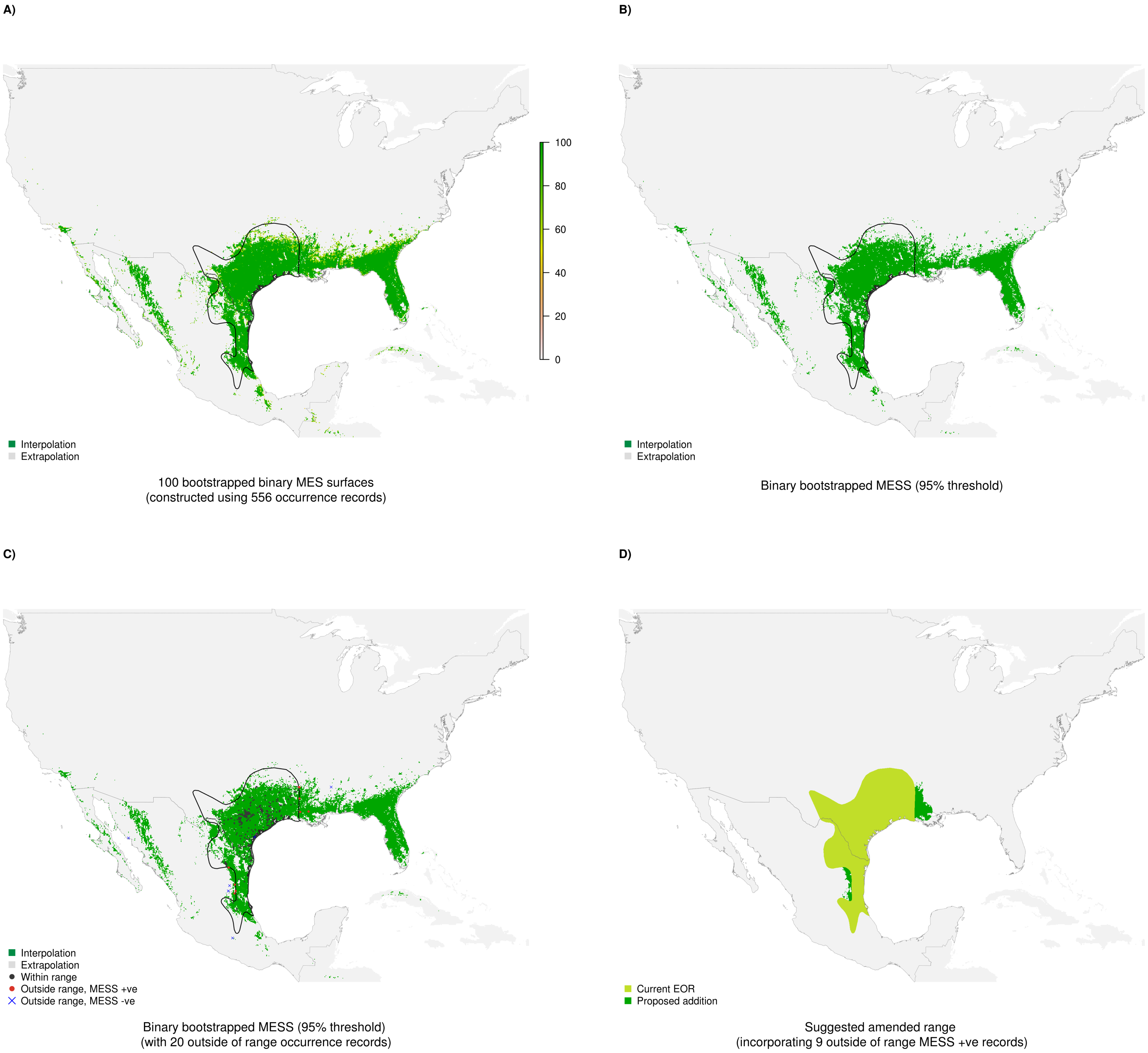

Montivipera raddei

A)

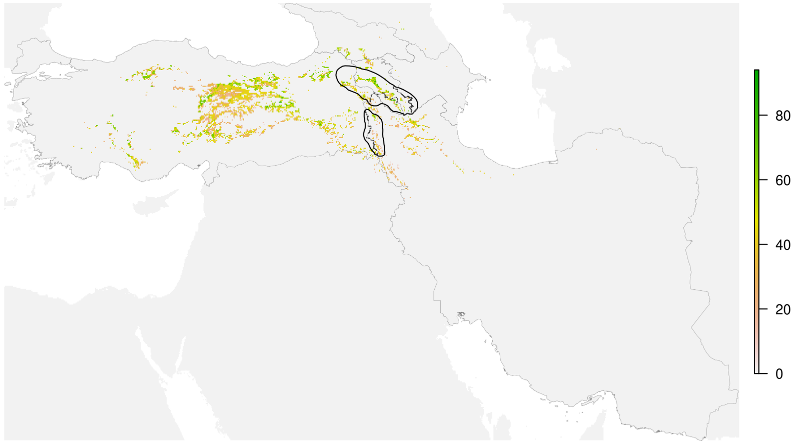

Interpolation
Extrapolation

100 bootstrapped binary MES surfaces
(constructed using 9 occurrence records)

B)

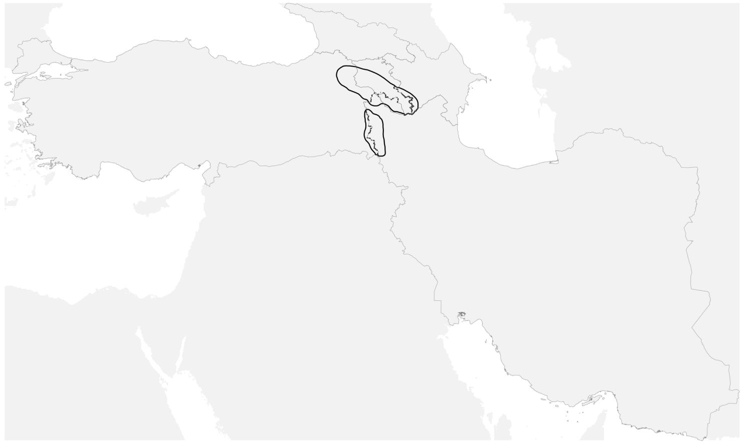

Interpolation
Extrapolation

Binary bootstrapped MESS (95% threshold)
[0 cells met this threshold]

B)

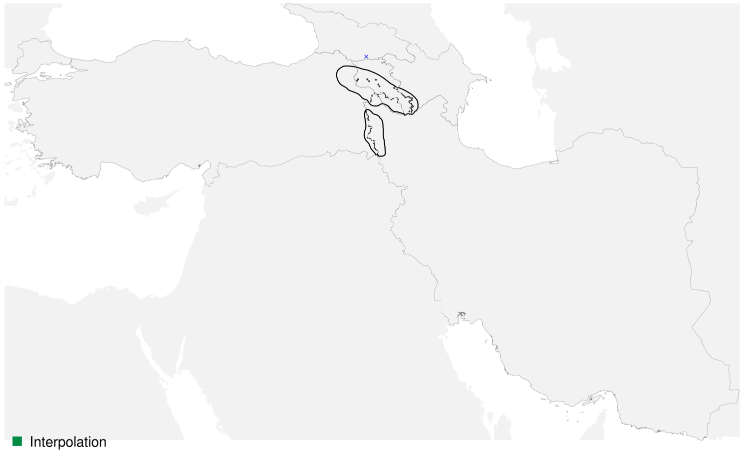

Interpolation
Extrapolation
Within range
Outside range, MESS +ve
Outside range, MESS -ve

Binary bootstrapped MESS (95% threshold)
(with 1 outside of range occurrence records)

D)

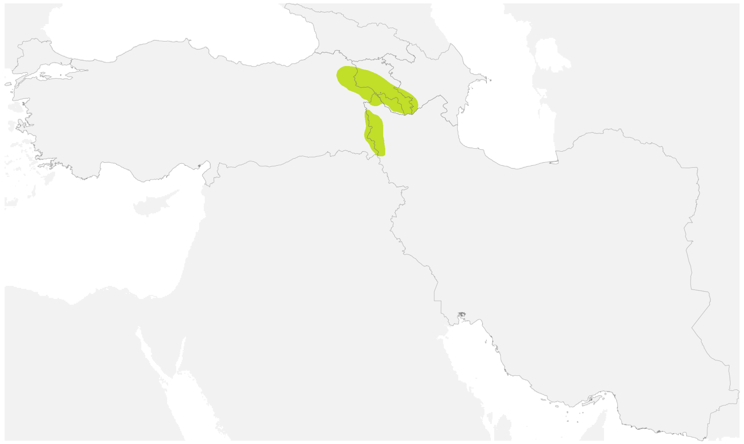

Current EOR
Proposed addition

Suggested amended range
(incorporating 0 outside of range MESS +ve records)

Montivipera xanthina

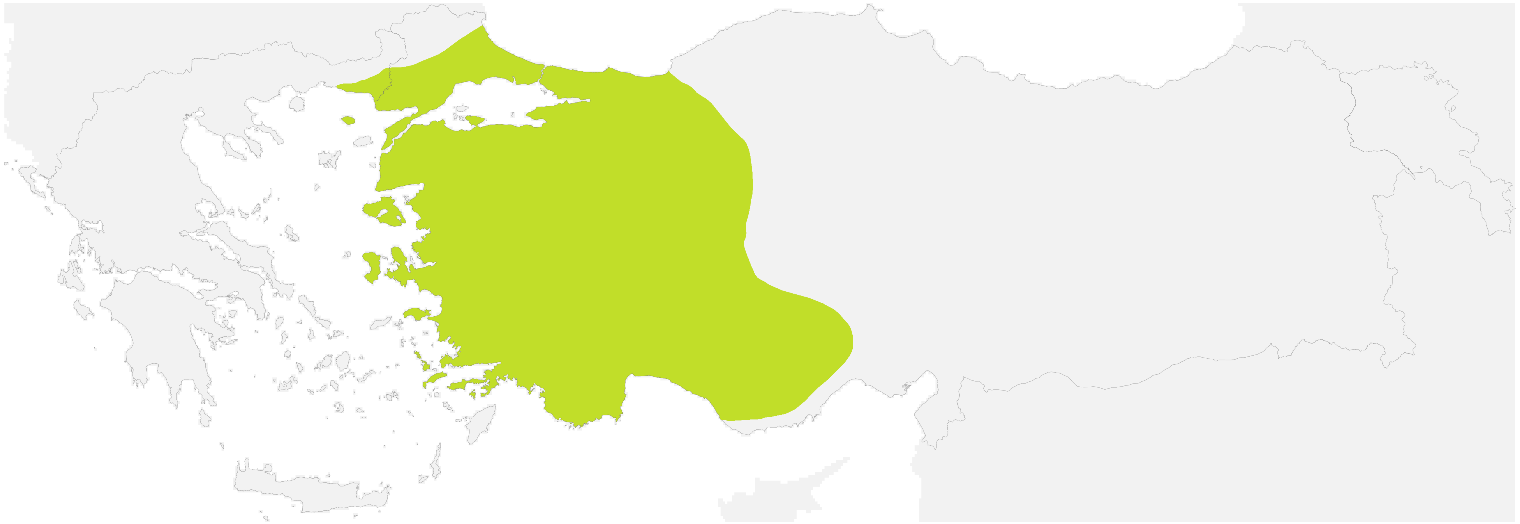

Current EOR

Naja anchietae

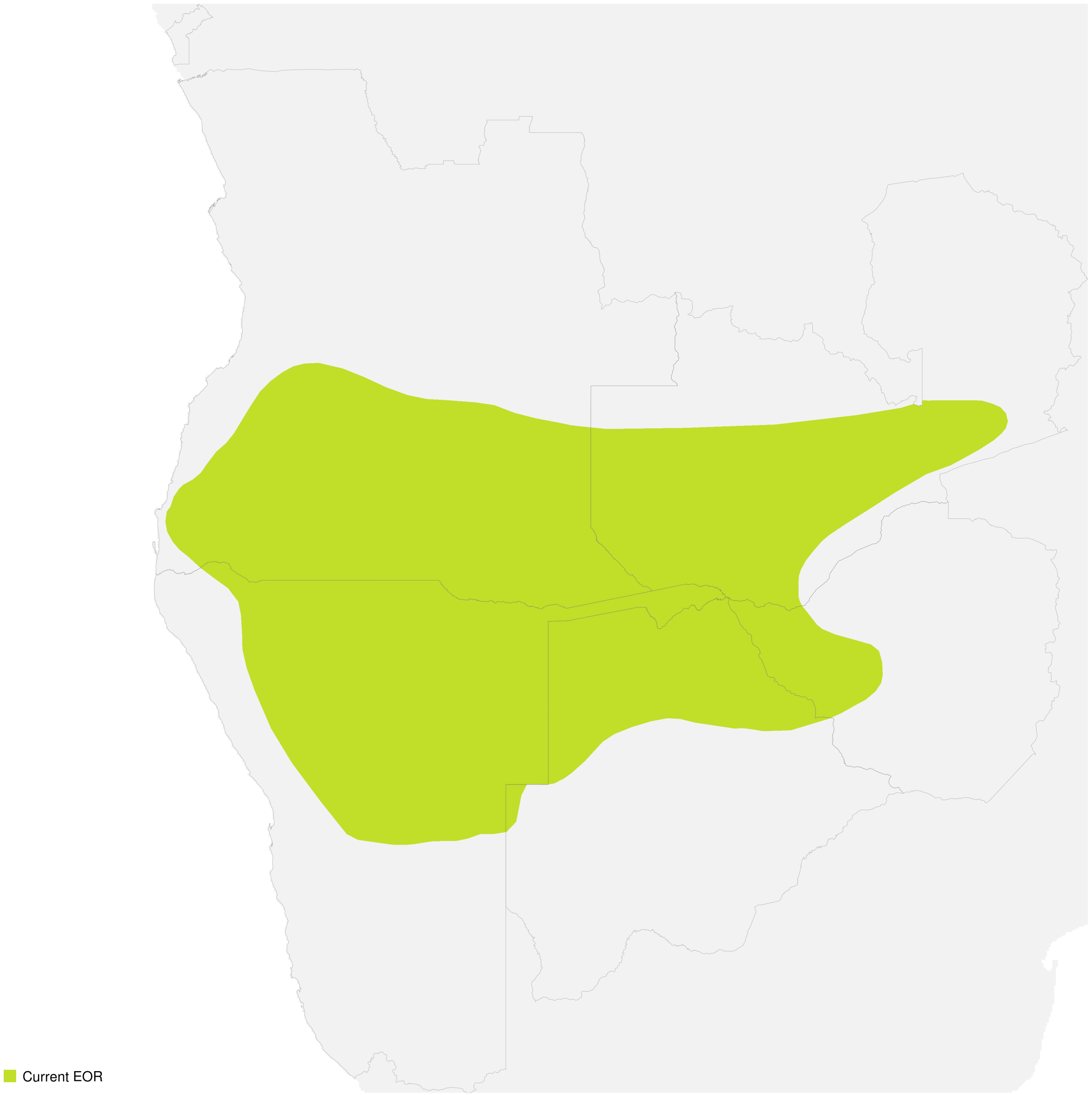

Naja annulata

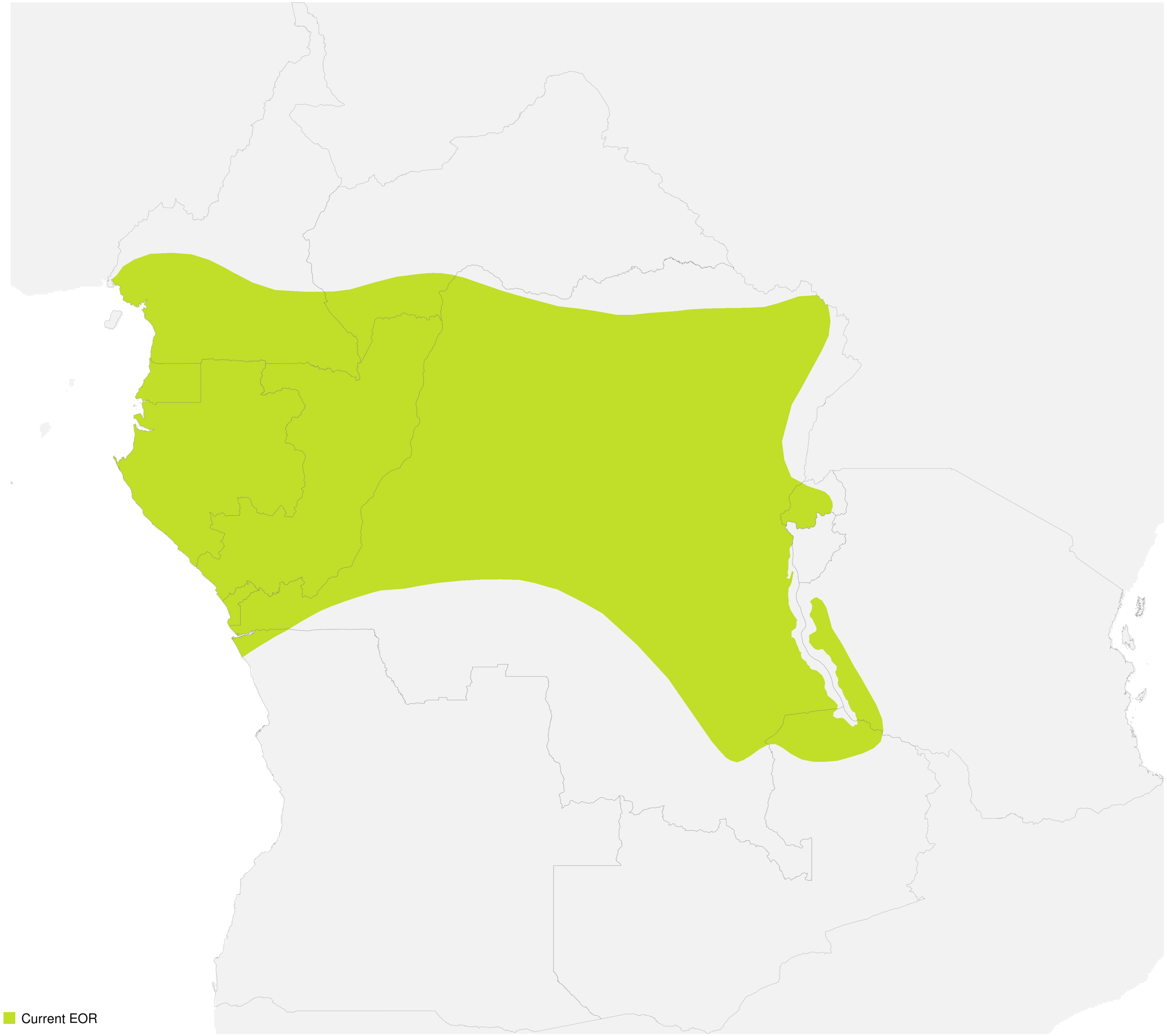

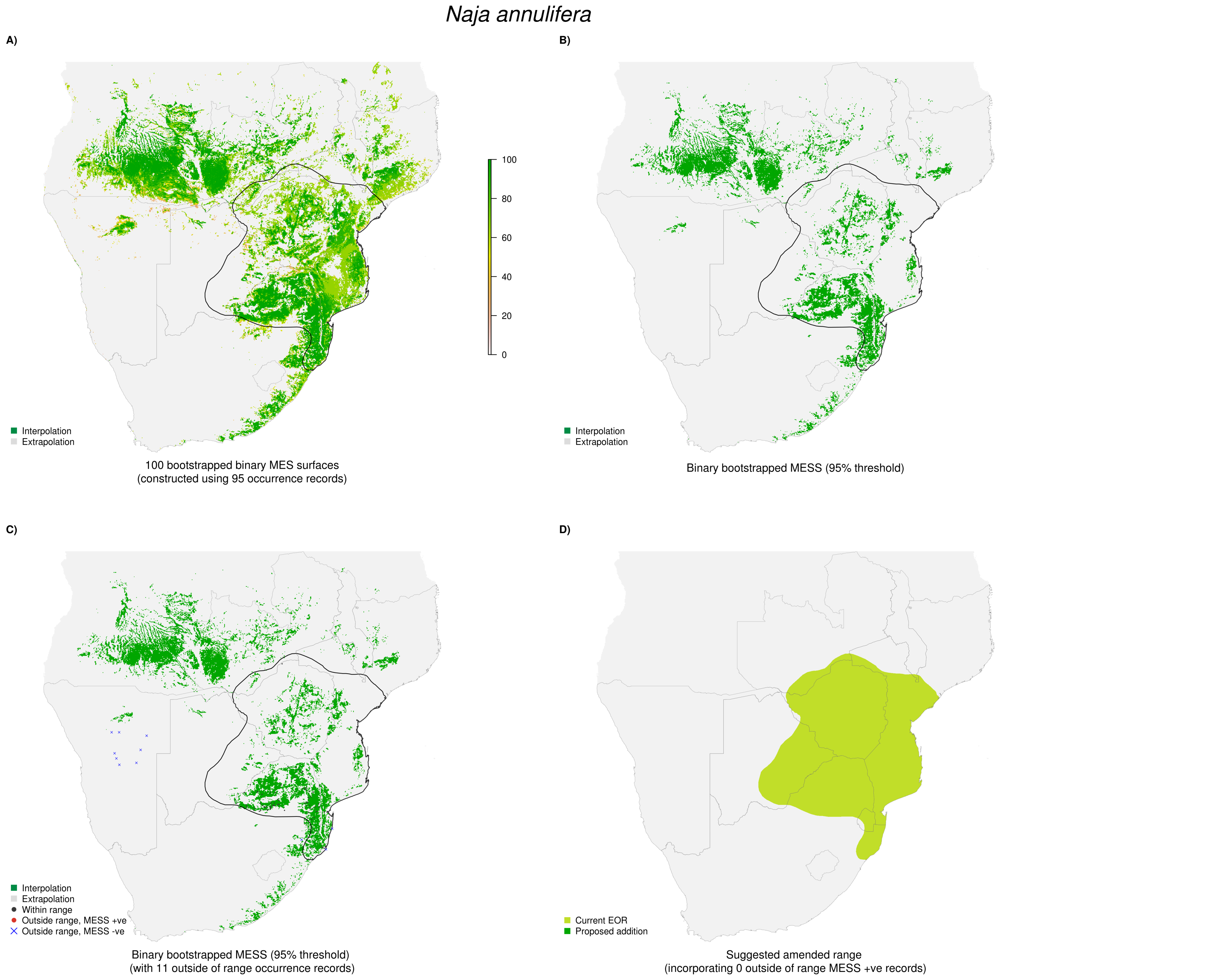
Naja annulifera
A)
100
80
60
40
20
0
Interpolation
Extrapolation
100 bootstrapped binary MES surfaces
(constructed using 95 occurrence records)
B)
Interpolation
Extrapolation
Binary bootstrapped MESS (95% threshold)
C)
Interpolation
Extrapolation
Within range
Outside range, MESS +ve
Outside range, MESS -ve
Binary bootstrapped MESS (95% threshold)
(with 11 outside of range occurrence records)
D)
Current EOR
Proposed addition
Suggested amended range
(incorporating 0 outside of range MESS +ve records)

Naja arabica

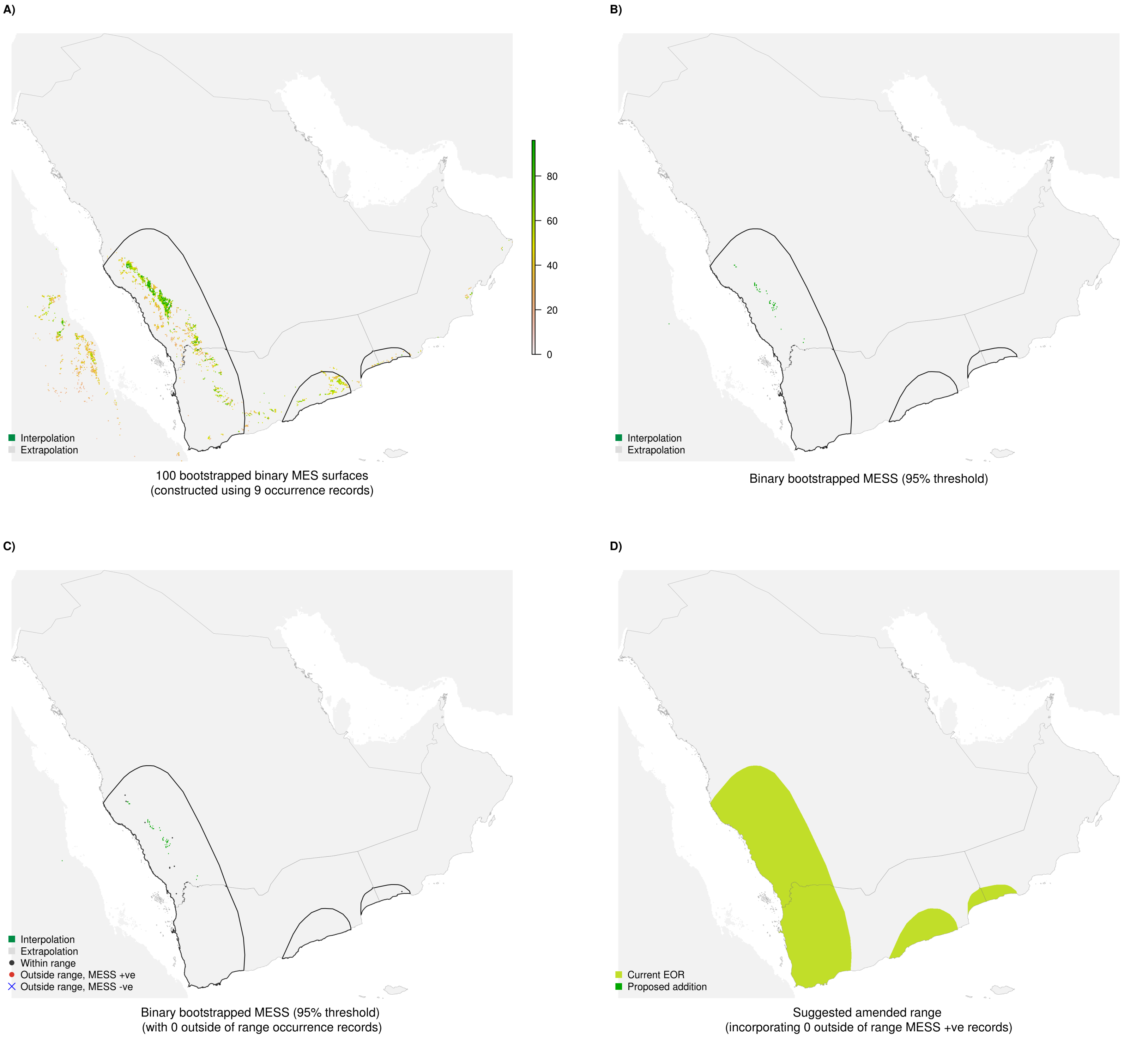

Naja ashei

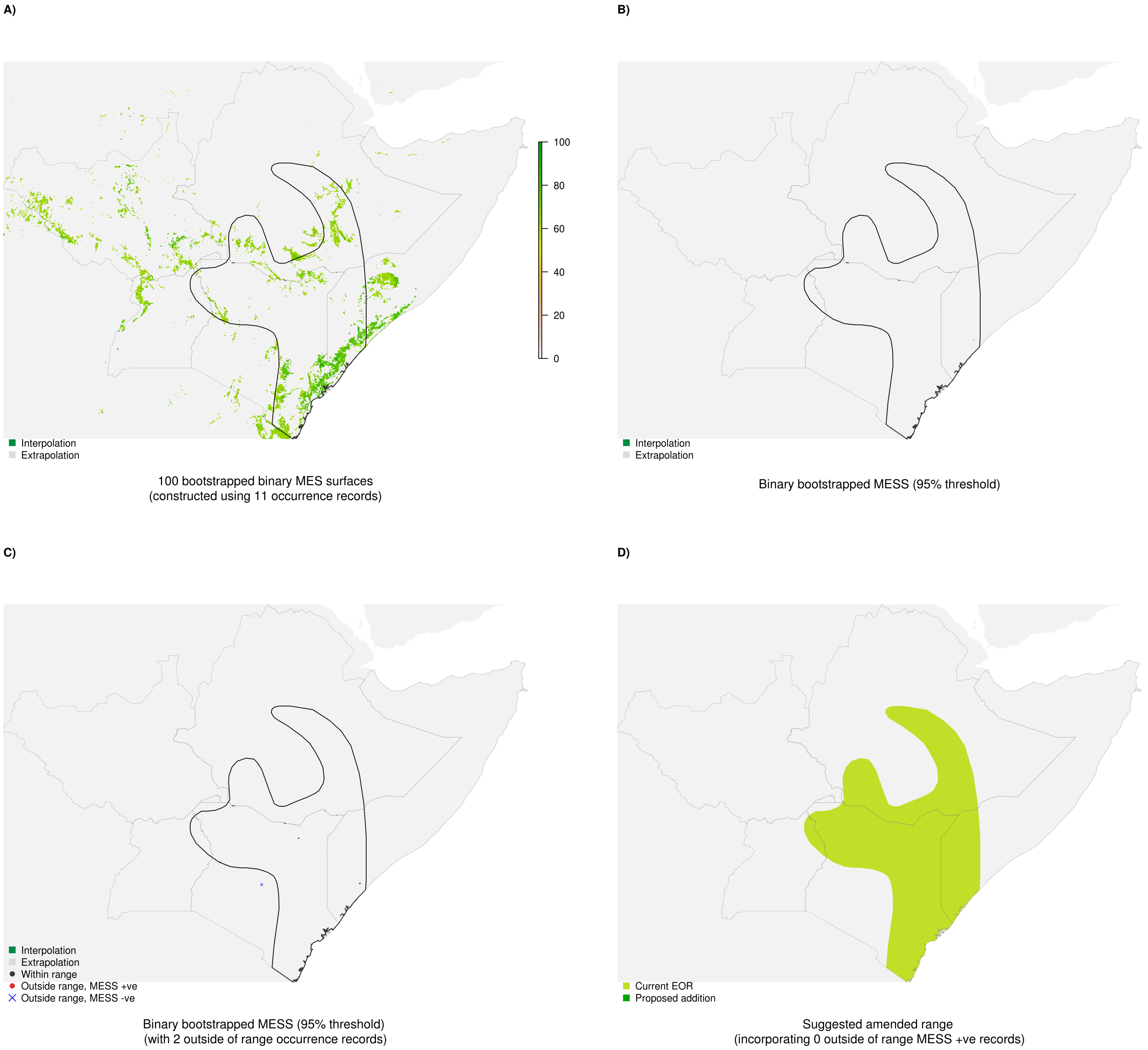
A)
100
80
60
40
20
0
Interpolation
Extrapolation
100 bootstrapped binary MES surfaces
(constructed using 11 occurrence records)
B)
Interpolation
Extrapolation
Binary bootstrapped MESS (95% threshold)
C)
Interpolation
Extrapolation
Within range
Outside range, MESS +ve
Outside range, MESS -ve
Binary bootstrapped MESS (95% threshold)
(with 2 outside of range occurrence records)
D)
Current EOR
Proposed addition
Suggested amended range
(incorporating 0 outside of range MESS +ve records)

Naja atra

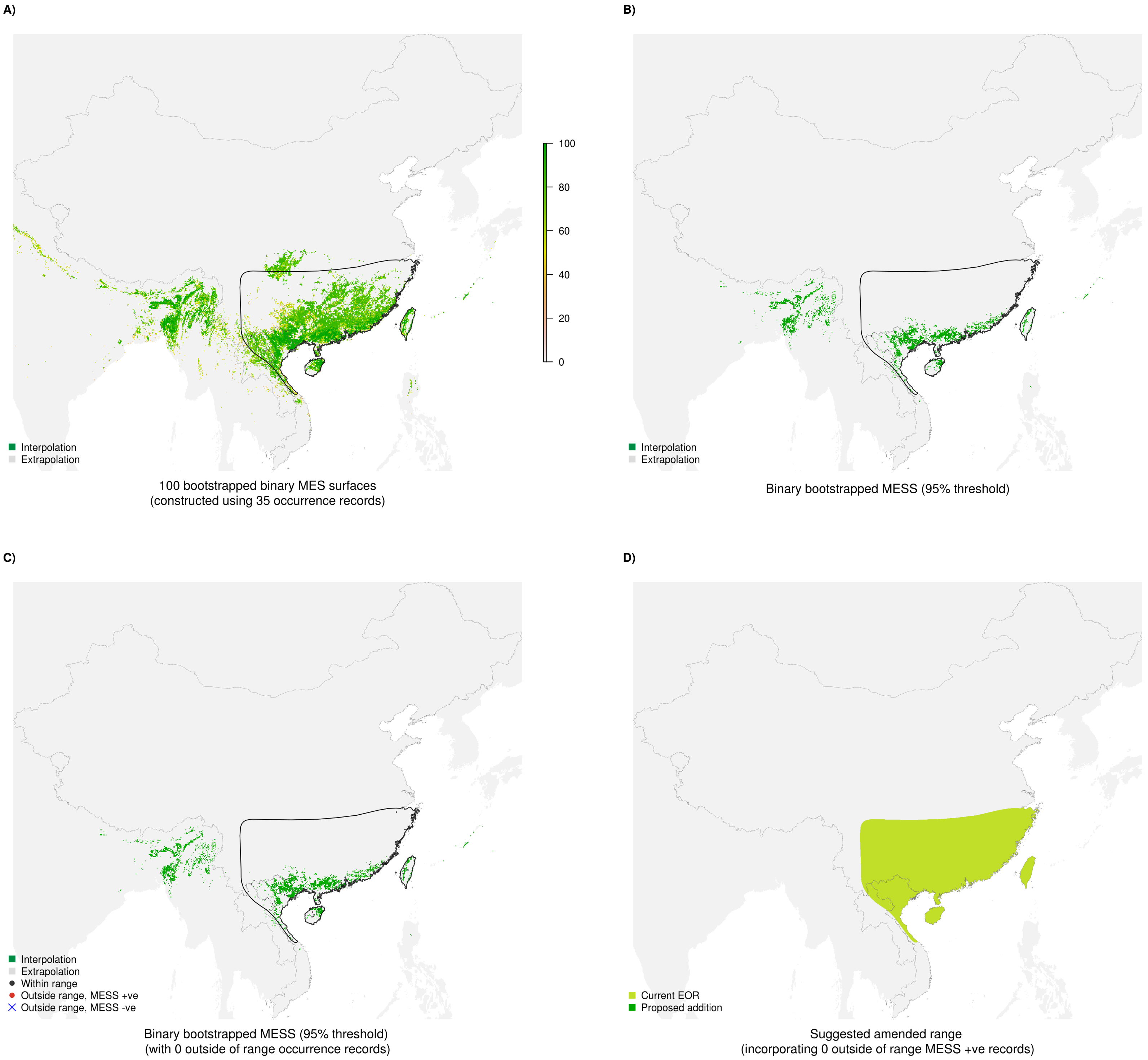
A)
100
80
60
40
20
0
Interpolation
Extrapolation
100 bootstrapped binary MES surfaces
(constructed using 35 occurrence records)
B)
Interpolation
Extrapolation
Binary bootstrapped MESS (95% threshold)
C)
Interpolation
Extrapolation
Within range
Outside range, MESS +ve
Outside range, MESS -ve
Binary bootstrapped MESS (95% threshold)
(with 0 outside of range occurrence records)
D)
Current EOR
Proposed addition
Suggested amended range
(incorporating 0 outside of range MESS +ve records)

Naja christyi

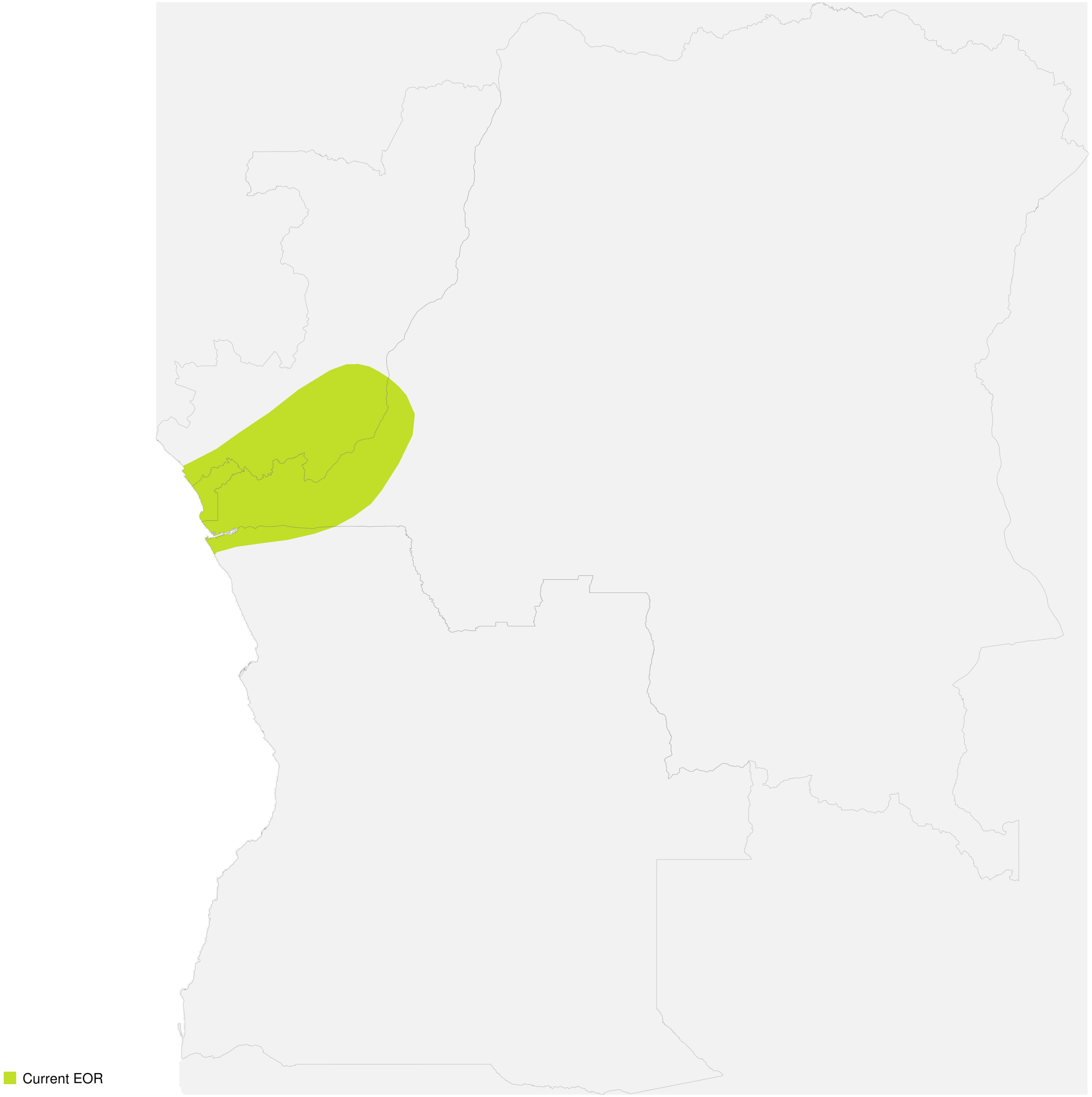

Naja haje

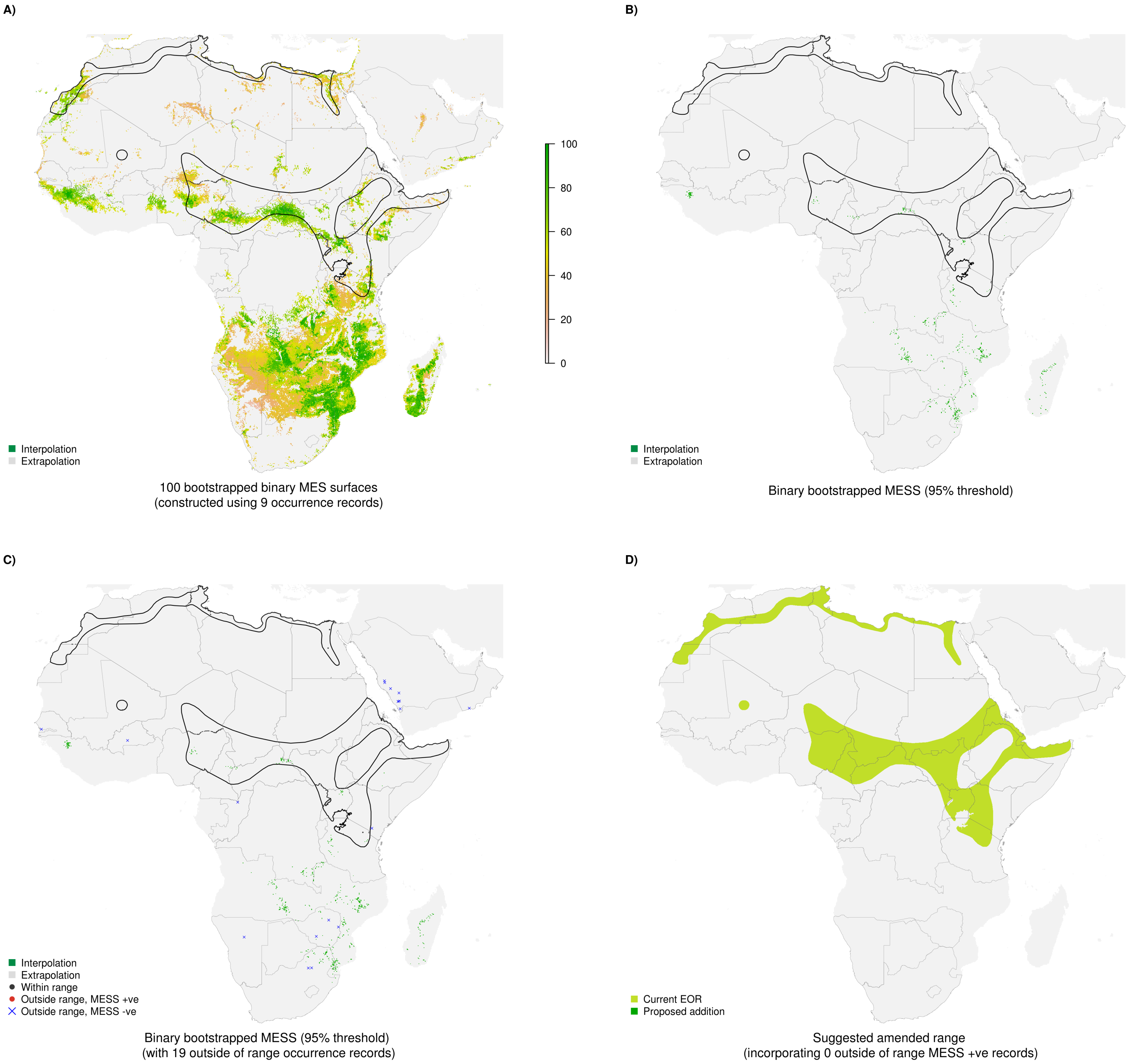
A)
100
80
60
40
20
0
Interpolation
Extrapolation
100 bootstrapped binary MES surfaces
(constructed using 9 occurrence records)
B)
Interpolation
Extrapolation
Binary bootstrapped MESS (95% threshold)
C)
Interpolation
Extrapolation
Within range
Outside range, MESS +ve
Outside range, MESS -ve
Binary bootstrapped MESS (95% threshold)
(with 19 outside of range occurrence records)
D)
Current EOR
Proposed addition
Suggested amended range
(incorporating 0 outside of range MESS +ve records)

Naja kaouthia

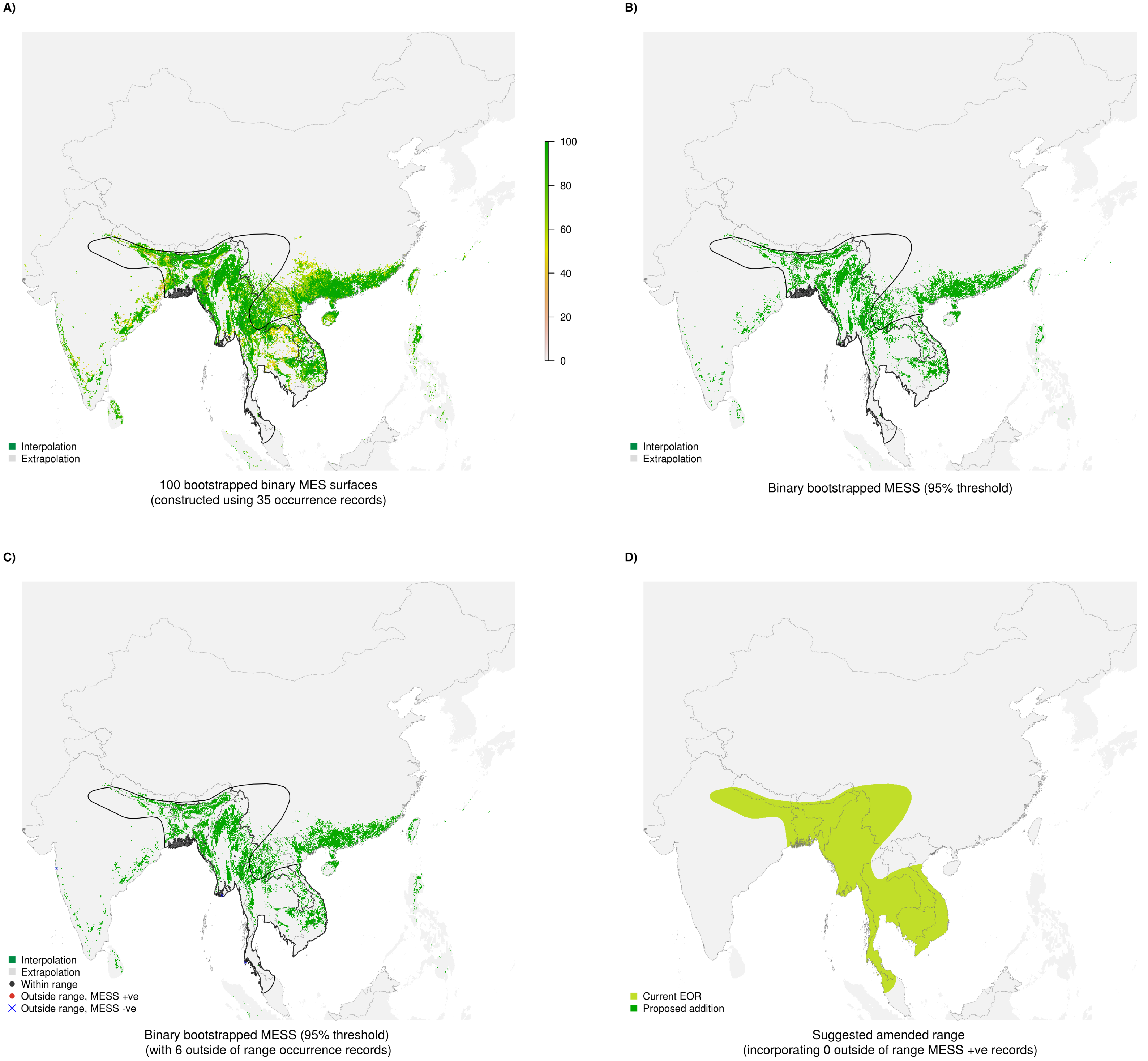

Naja katiensis

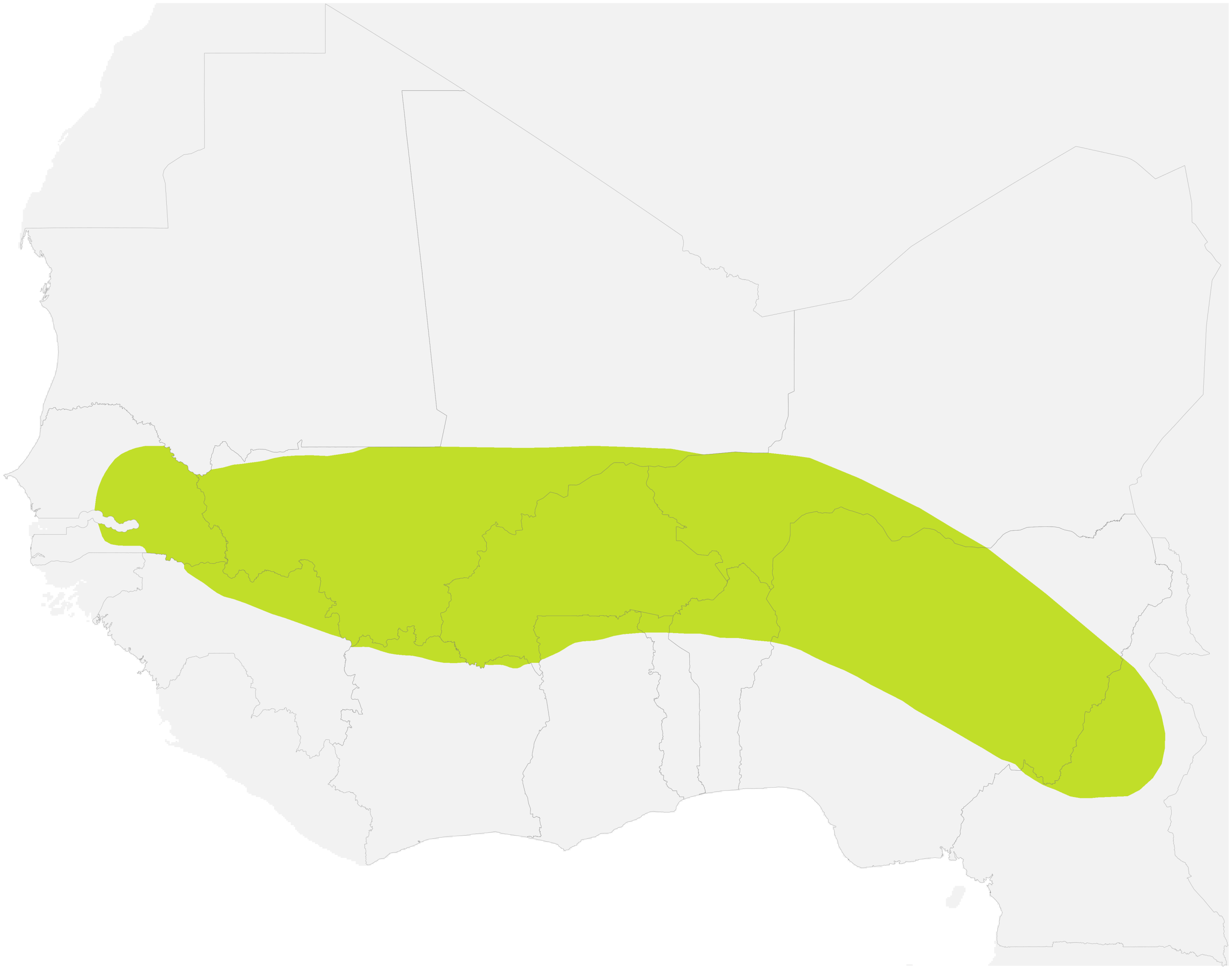

Current EOR

Naja mandalayensis

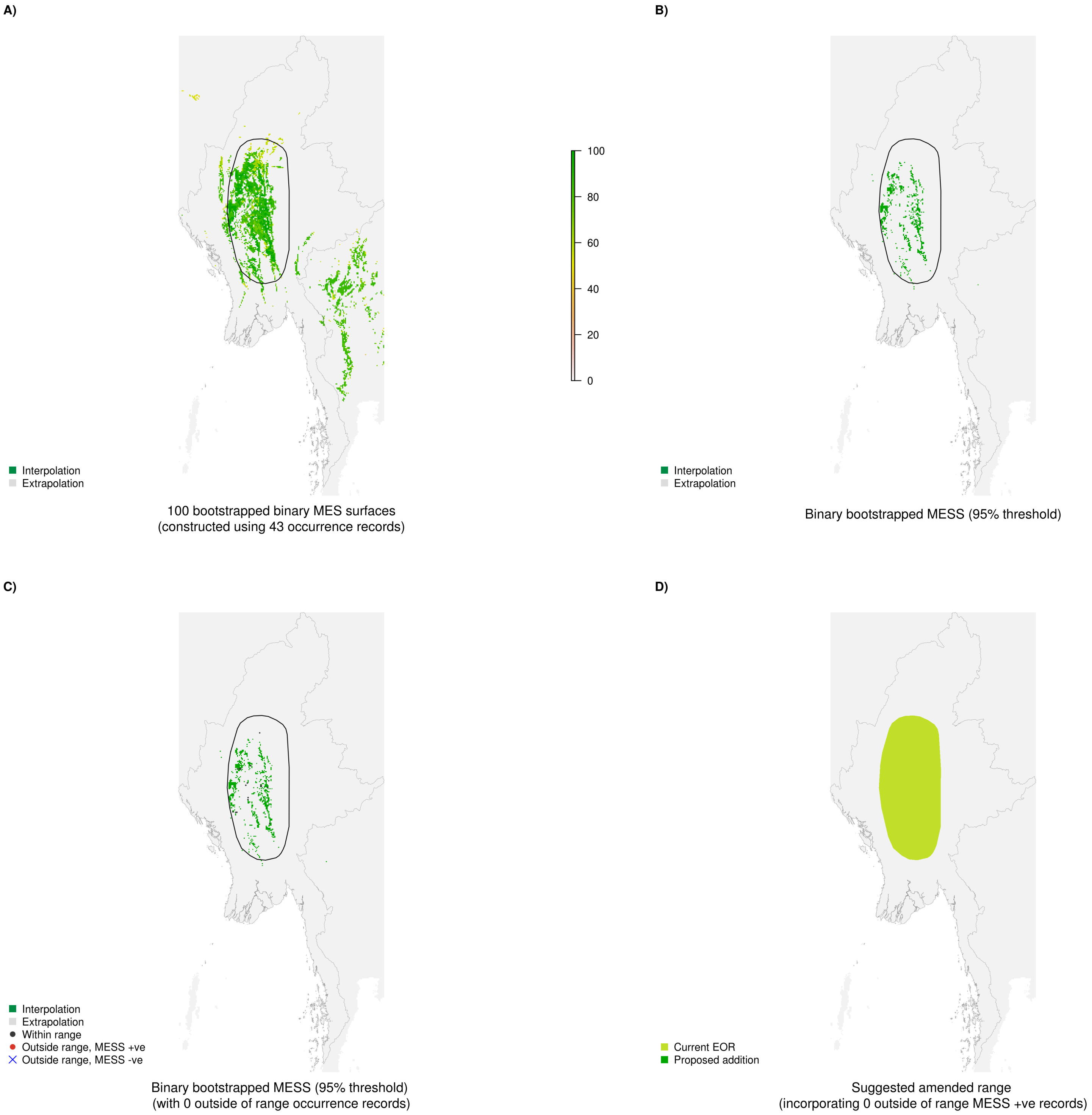

Naja melanoleuca

A)

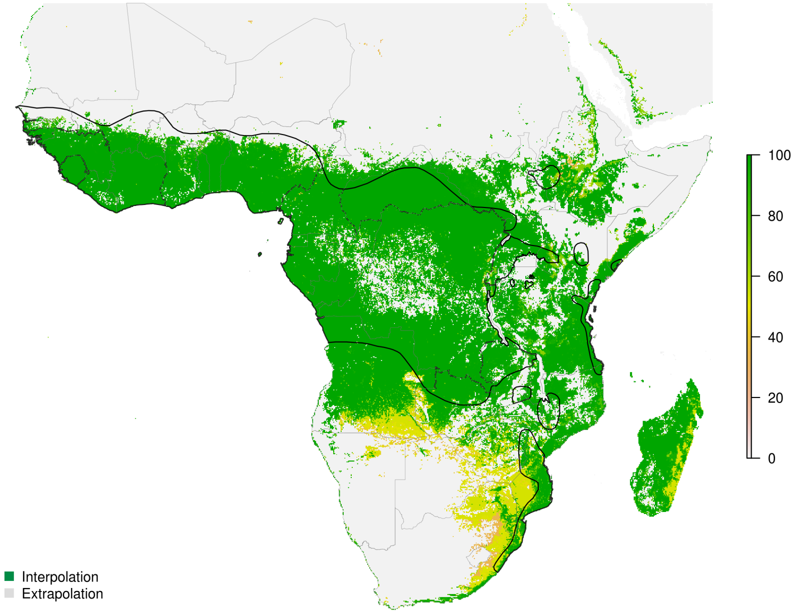

100 bootstrapped binary MES surfaces
(constructed using 355 occurrence records)

B)

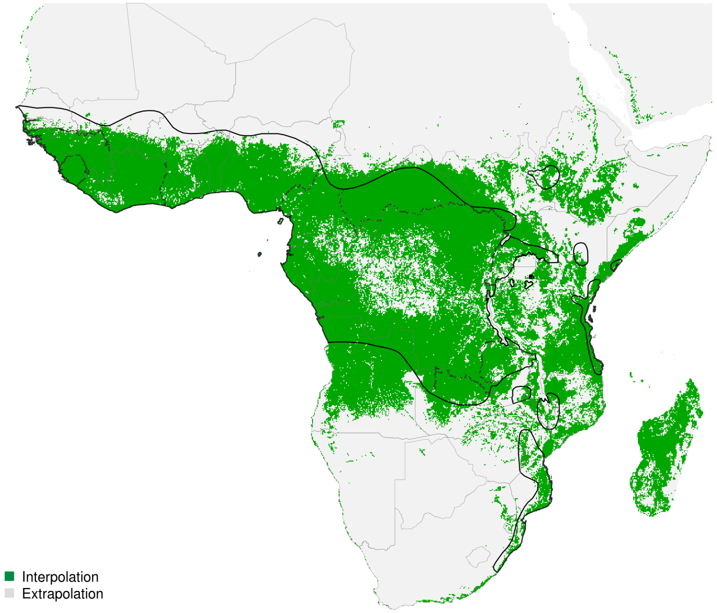

Binary bootstrapped MESS (95% threshold)

C)

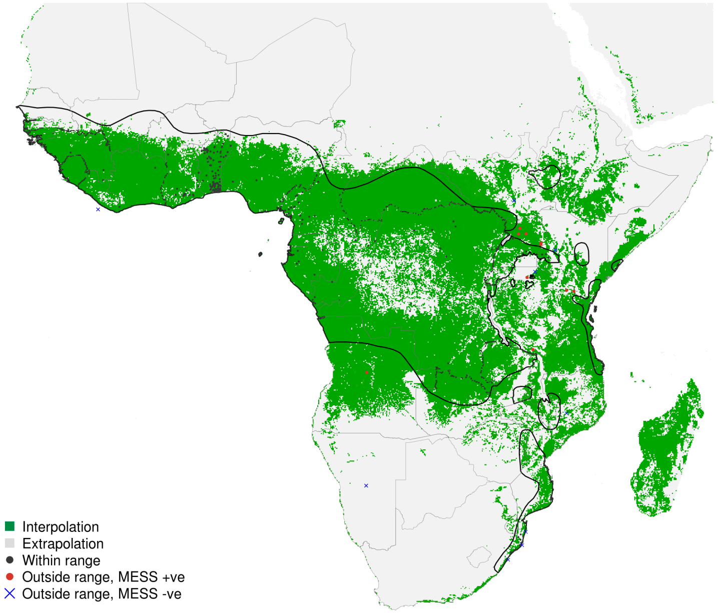

Binary bootstrapped MESS (95% threshold)
(with 30 outside of range occurrence records)

D)

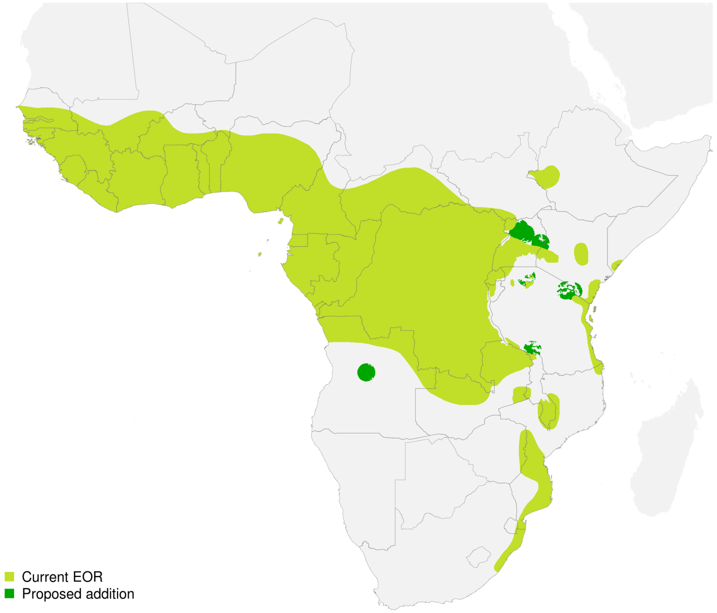

Suggested amended range
(incorporating 16 outside of range MESS +ve records)

Naja mossambica

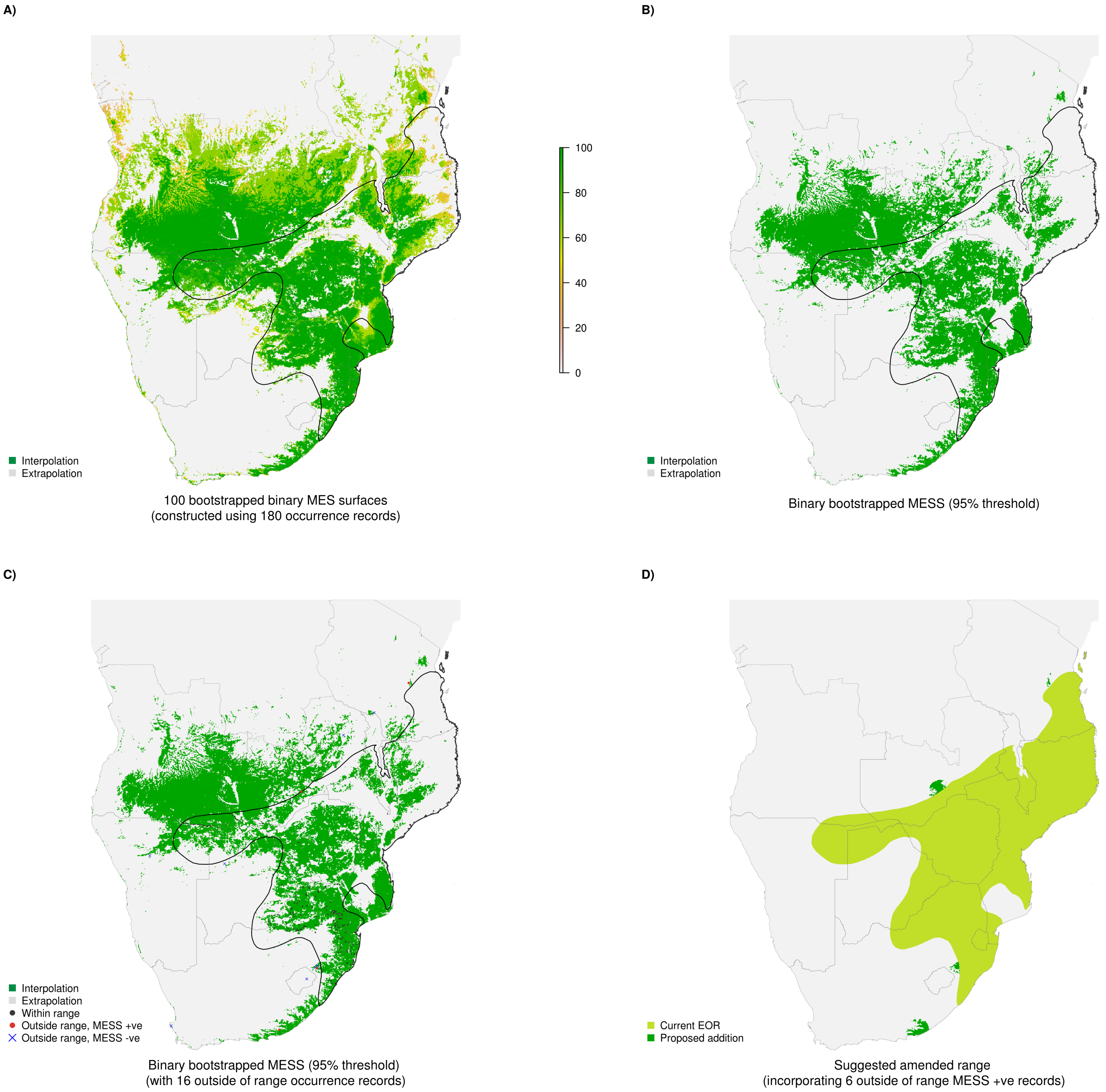

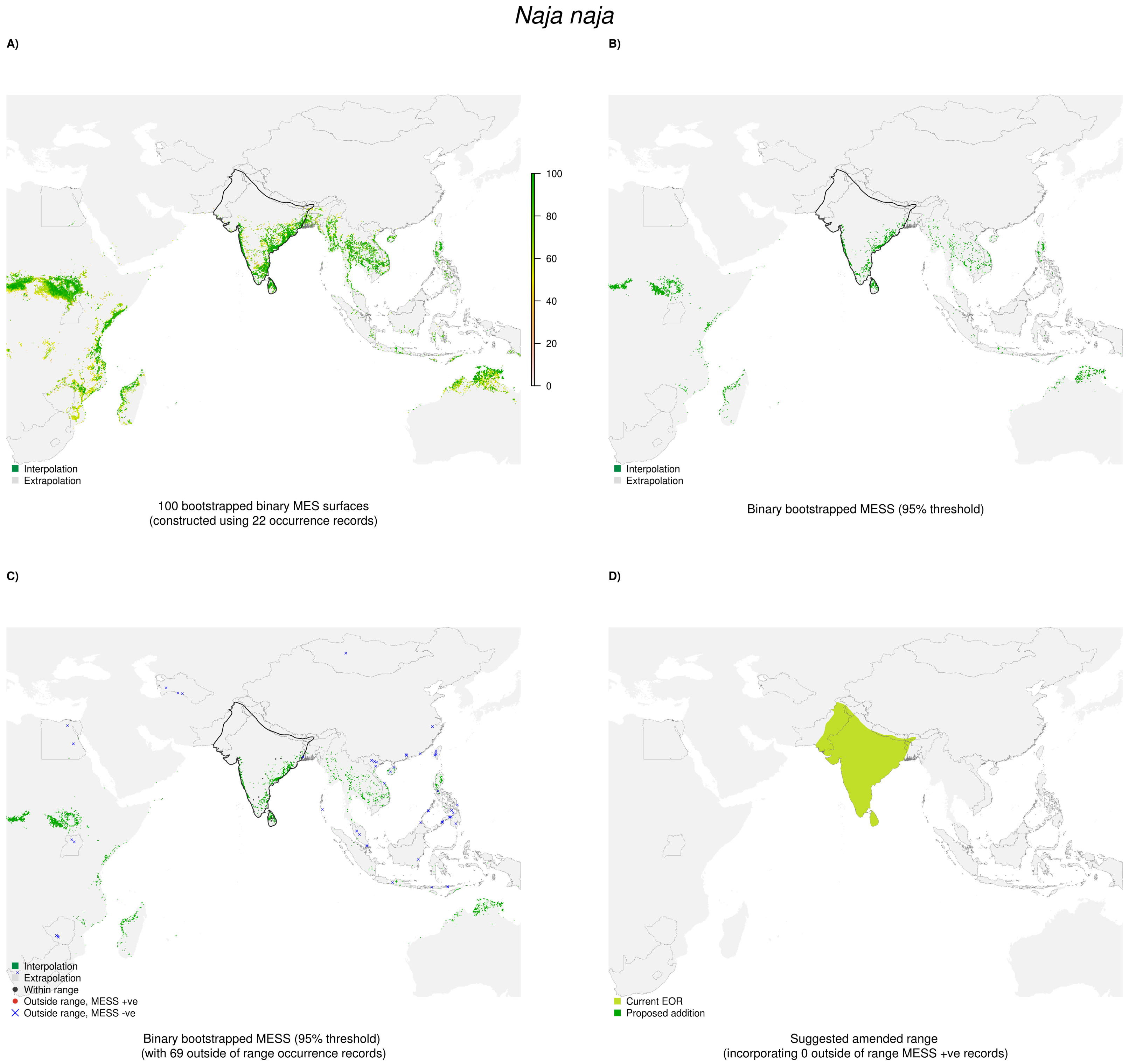
Naja naja
A)
100
80
60
40
20
0
Interpolation
Extrapolation
100 bootstrapped binary MES surfaces
(constructed using 22 occurrence records)
B)
Interpolation
Extrapolation
Binary bootstrapped MESS (95% threshold)
C)
Interpolation
Extrapolation
Within range
Outside range, MESS +ve
Outside range, MESS -ve
Binary bootstrapped MESS (95% threshold)
(with 69 outside of range occurrence records)
D)
Current EOR
Proposed addition
Suggested amended range
(incorporating 0 outside of range MESS +ve records)

Naja nigricincta

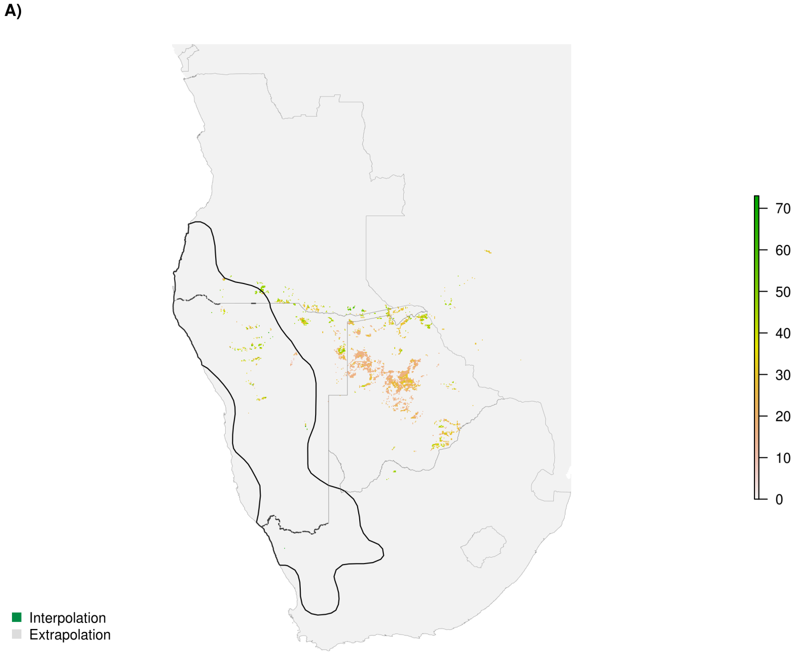

100 bootstrapped binary MES surfaces
(constructed using 5 occurrence records)

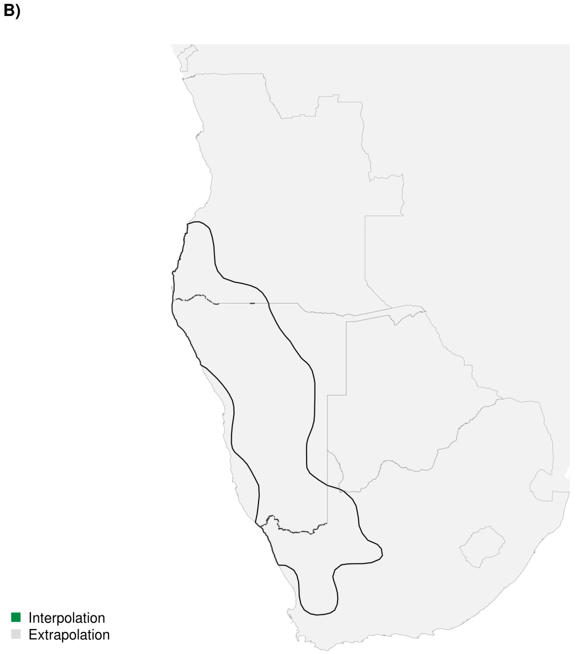

Binary bootstrapped MESS (95% threshold)
[0 cells met this threshold]

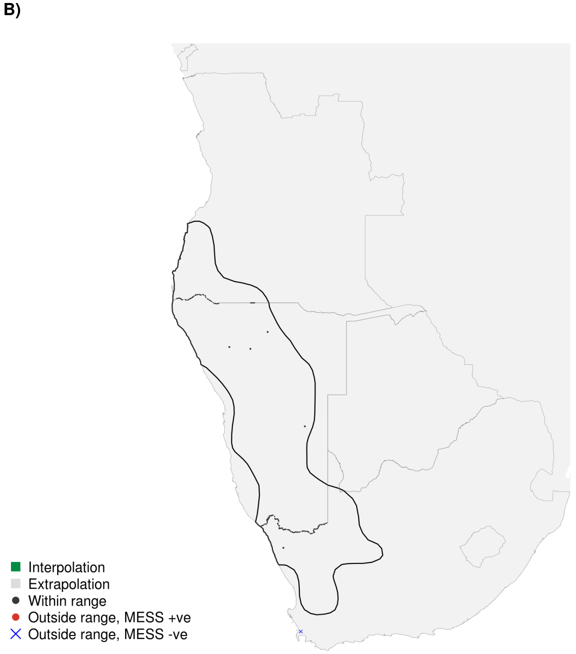

Binary bootstrapped MESS (95% threshold)
(with 1 outside of range occurrence records)

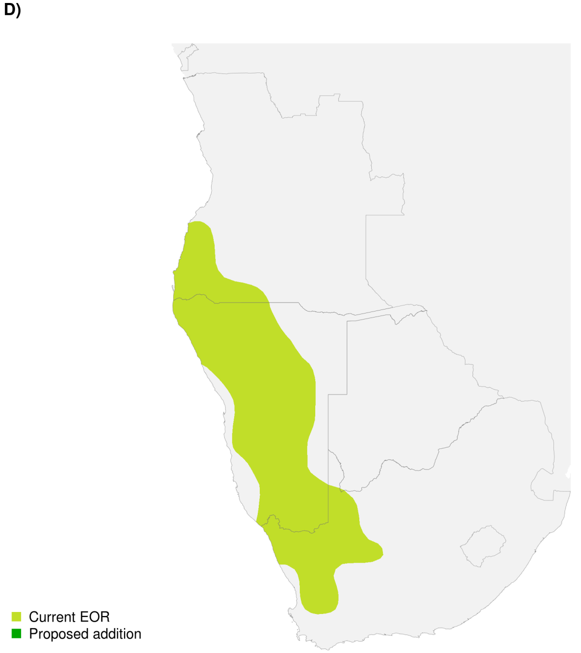

Suggested amended range
(incorporating 0 outside of range MESS +ve records)

Naja nigricollis

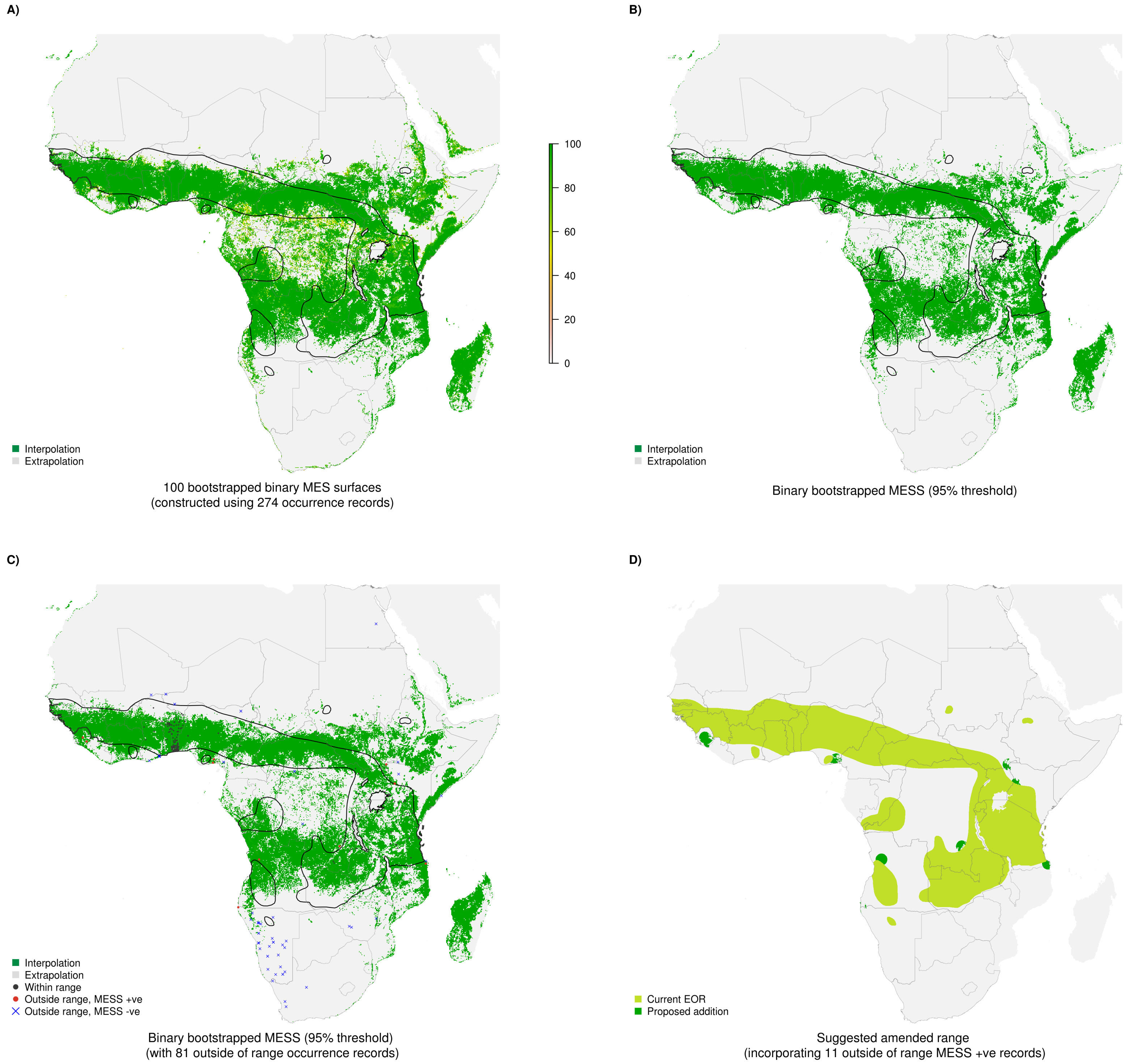

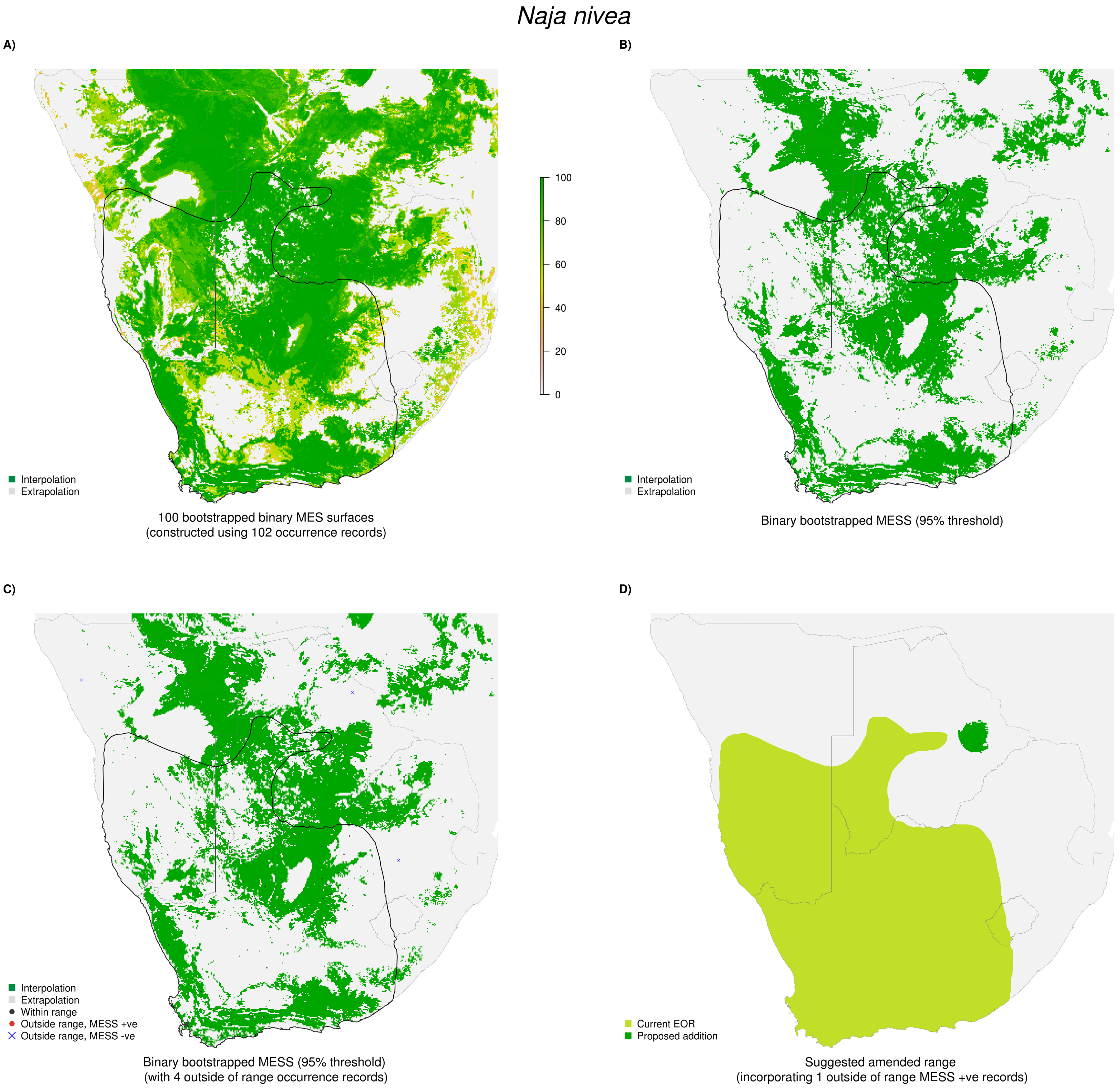
Naja nivea
A)
B)
C)
D)
100
80
60
40
20
0
Interpolation
Extrapolation
100 bootstrapped binary MES surfaces
(constructed using 102 occurrence records)
Interpolation
Extrapolation
Binary bootstrapped MESS (95% threshold)
Interpolation
Extrapolation
Within range
Outside range, MESS +ve
Outside range, MESS -ve
Binary bootstrapped MESS (95% threshold)
(with 4 outside of range occurrence records)
Current EOR
Proposed addition
Suggested amended range
(incorporating 1 outside of range MESS +ve records)

Naja nubiae

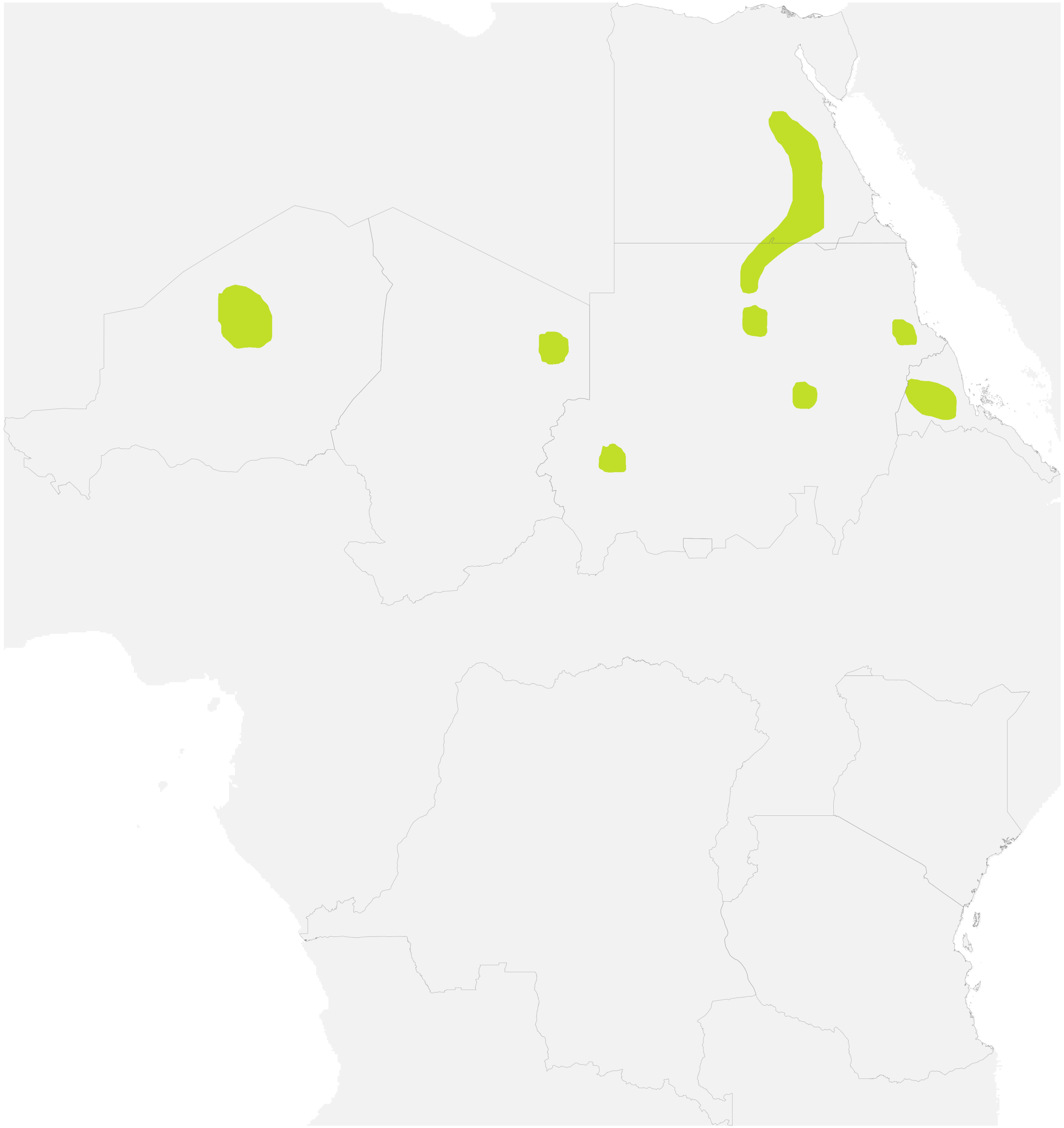

Naja oxiana

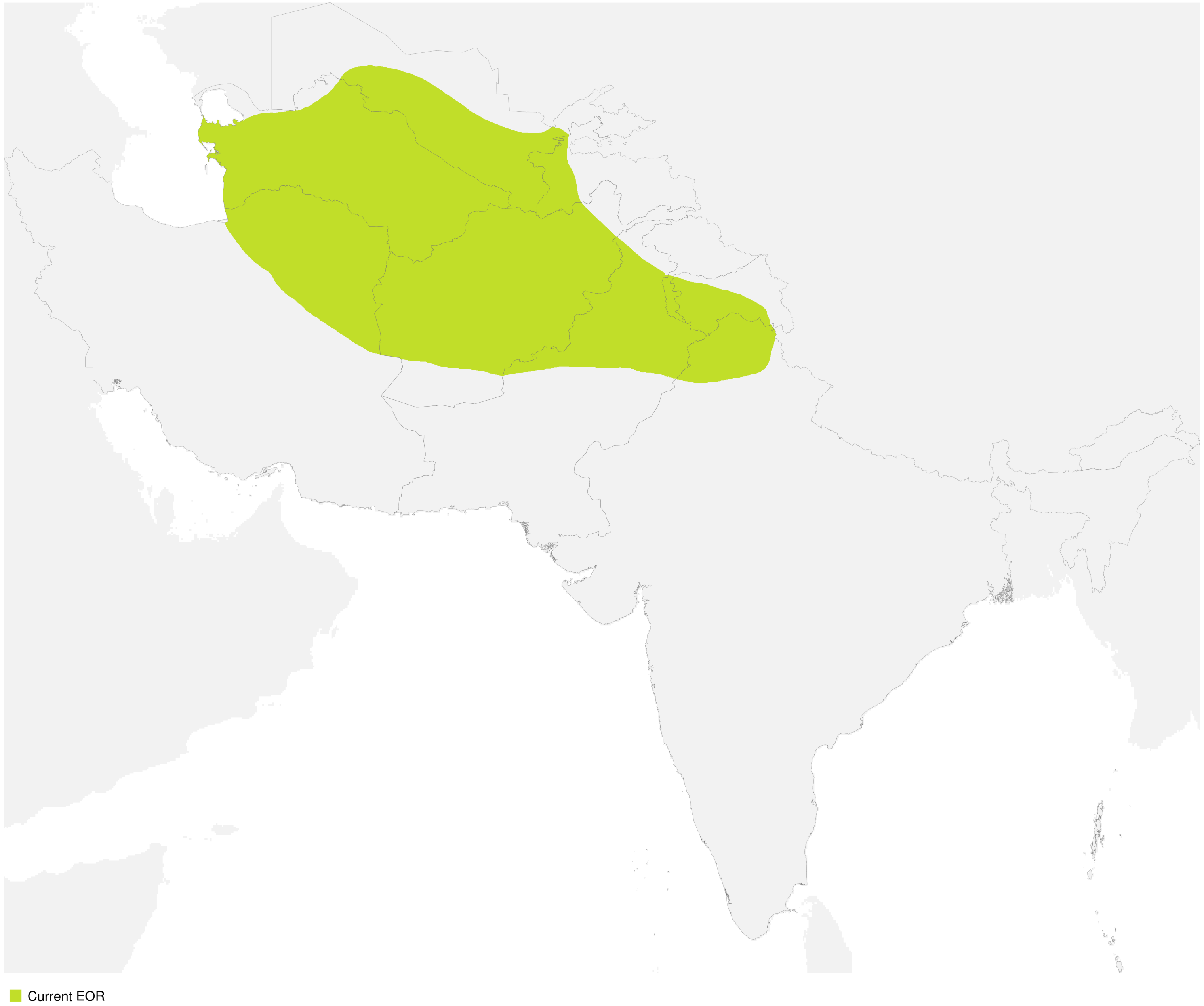

Naja pallida

A)

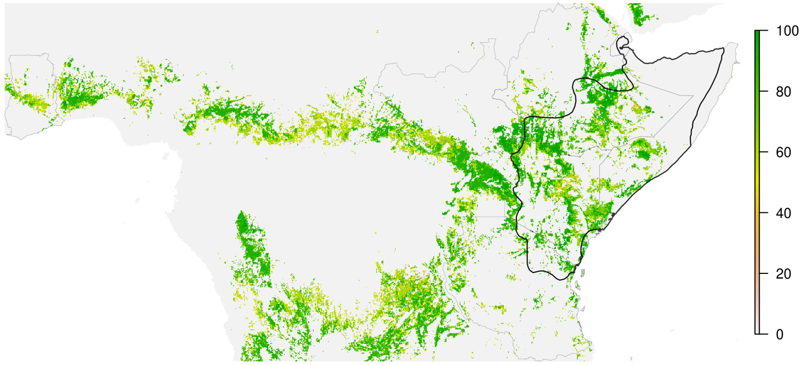

Interpolation
Extrapolation

100 bootstrapped binary MES surfaces
(constructed using 32 occurrence records)

B)

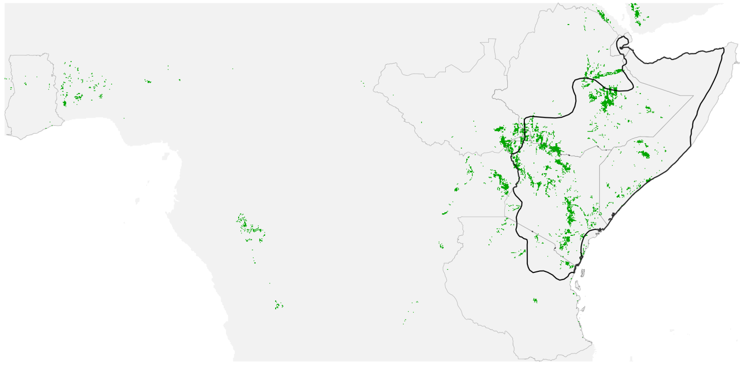

Interpolation
Extrapolation

Binary bootstrapped MESS (95% threshold)

C)

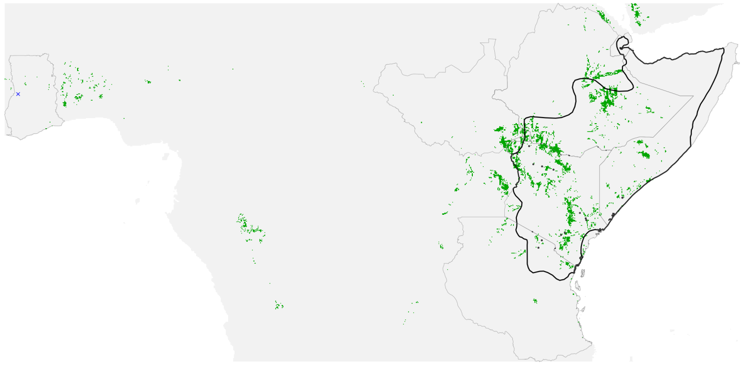

Interpolation
Extrapolation
Within range
Outside range, MESS +ve
Outside range, MESS -ve

Binary bootstrapped MESS (95% threshold)
(with 1 outside of range occurrence records)

D)

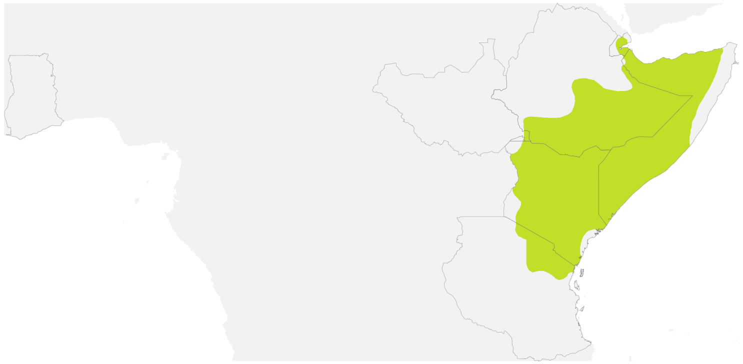

Current EOR
Proposed addition

Suggested amended range
(incorporating 0 outside of range MESS +ve records)

Naja philippinensis

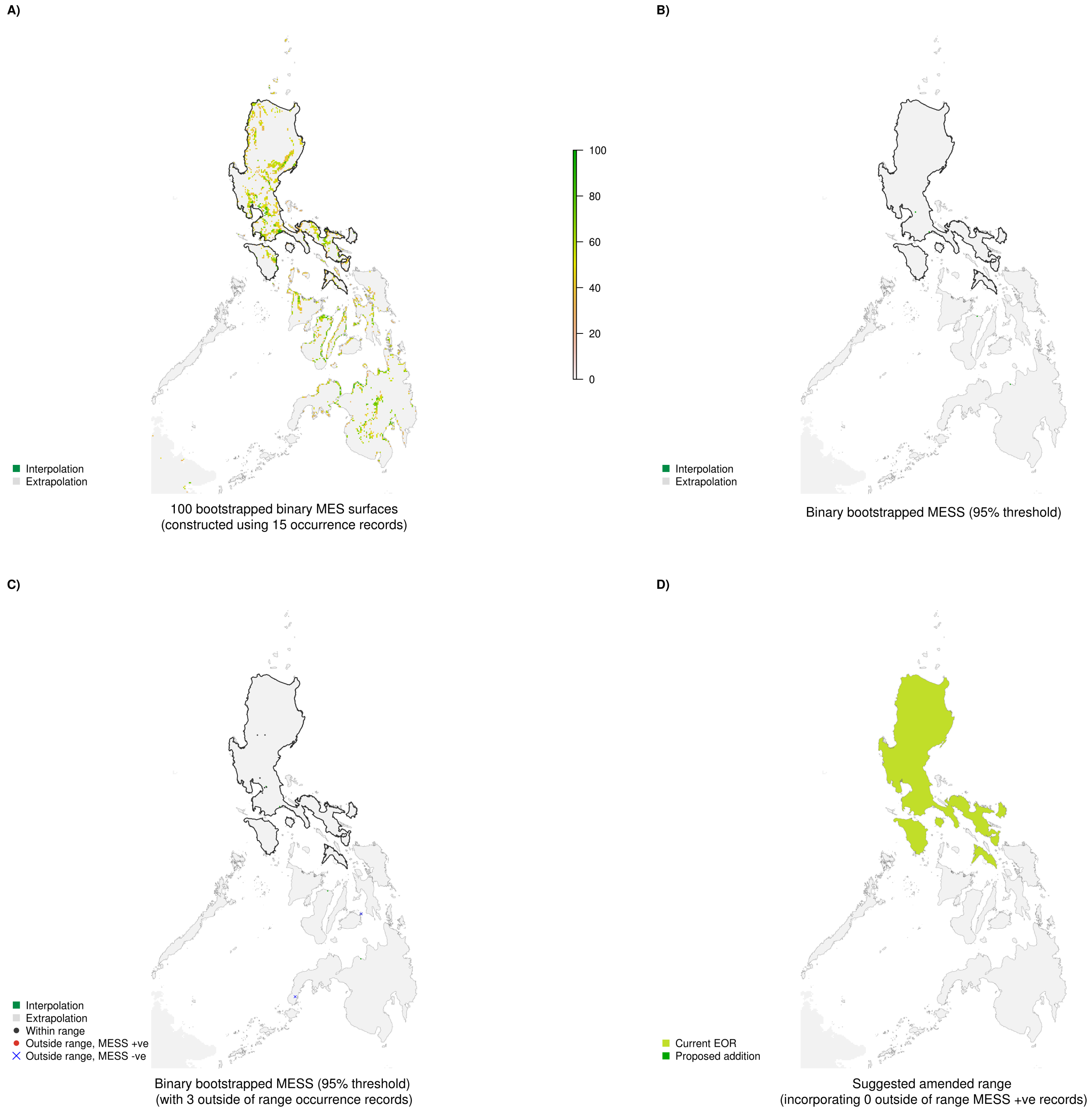

Naja sagittifera

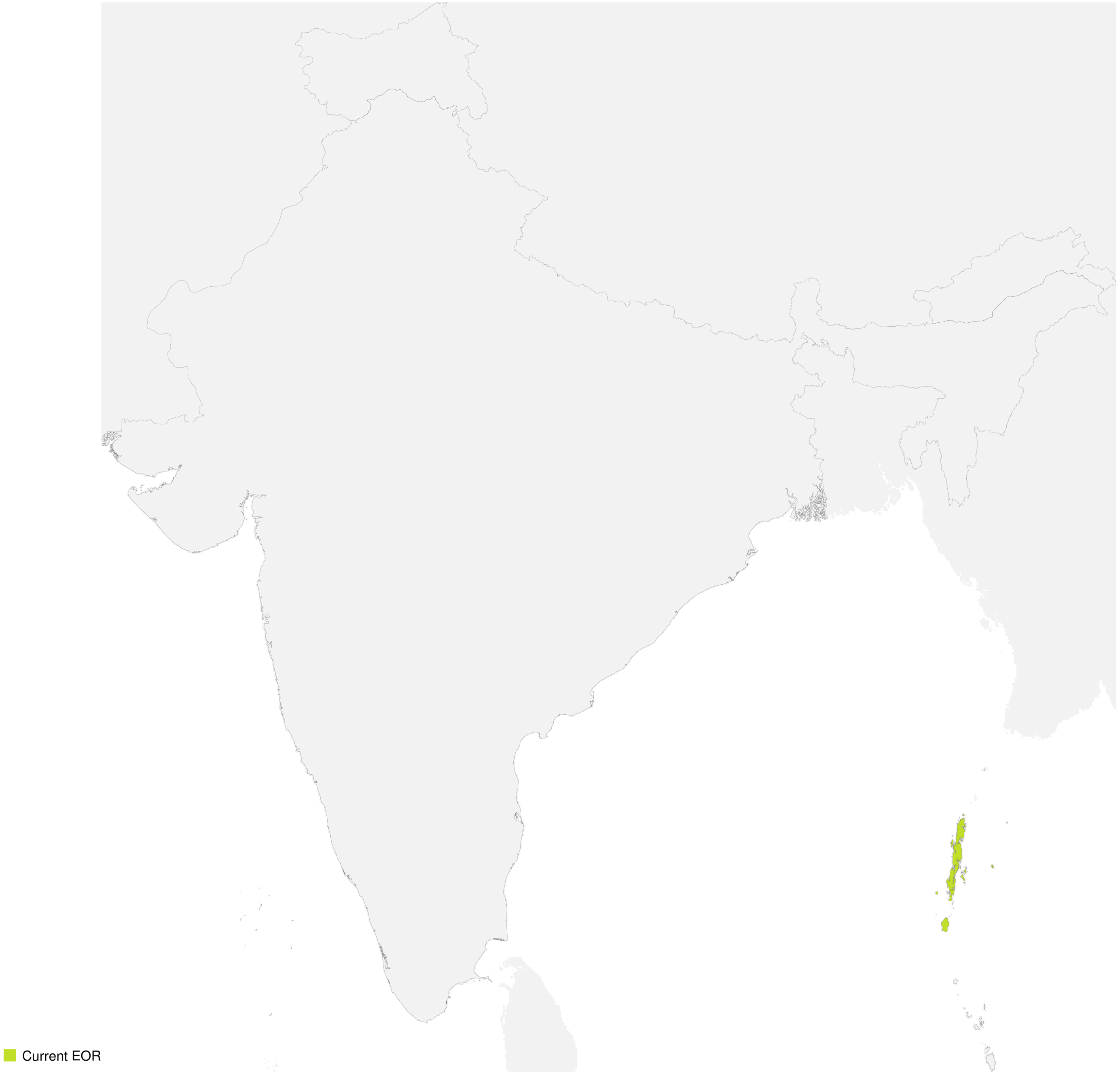

Naja samarensis

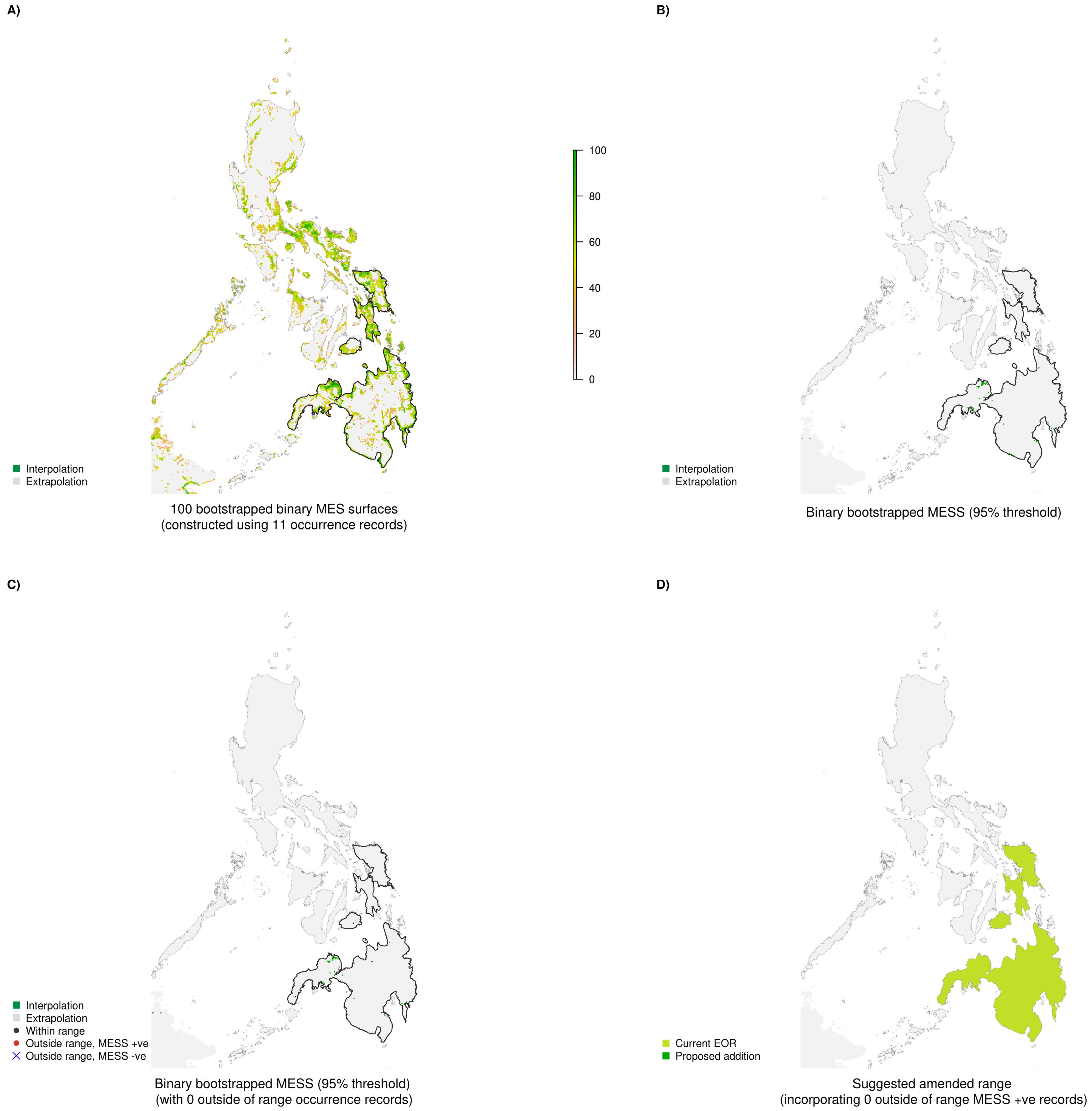

Naja senegalensis

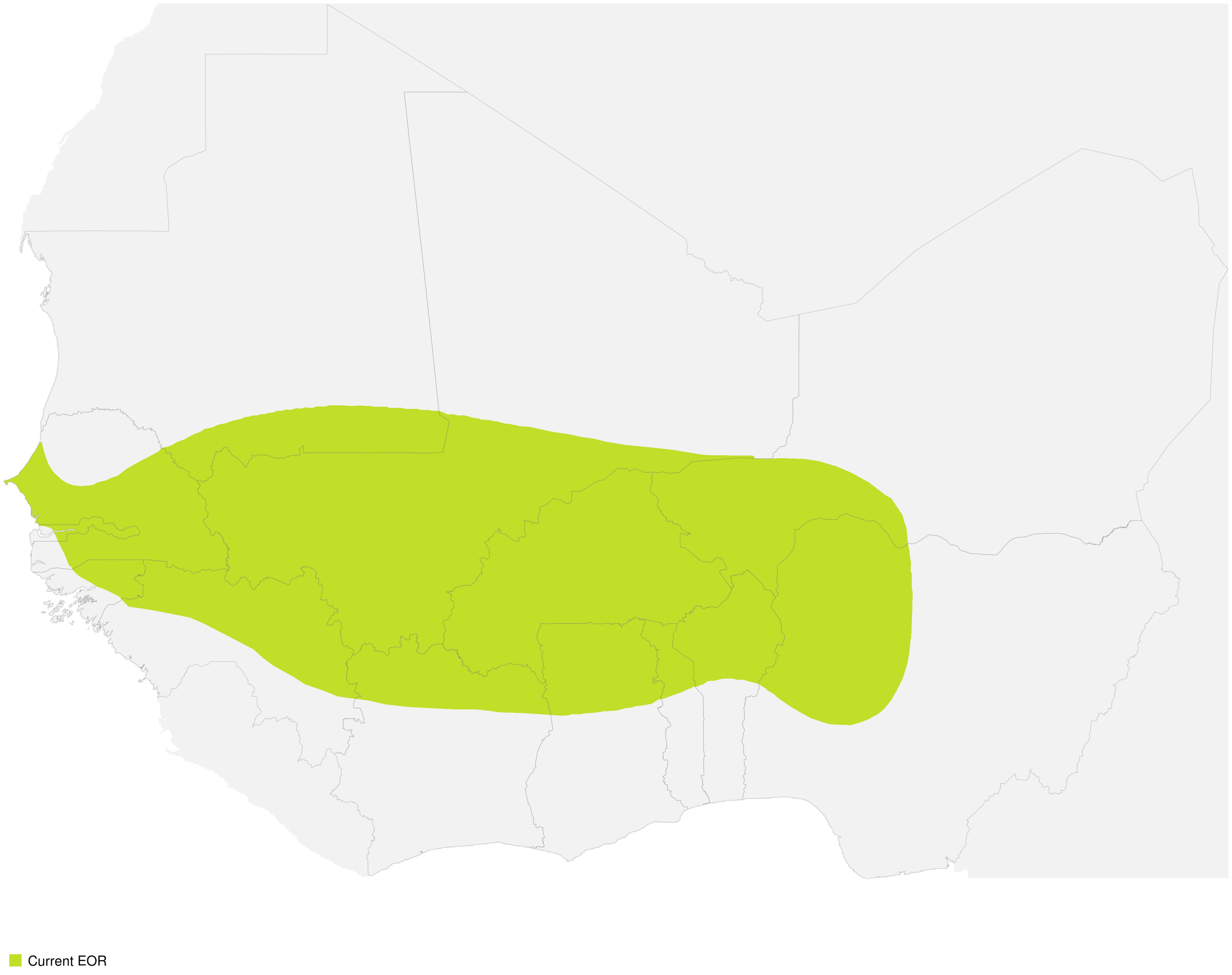

Naja siamensis

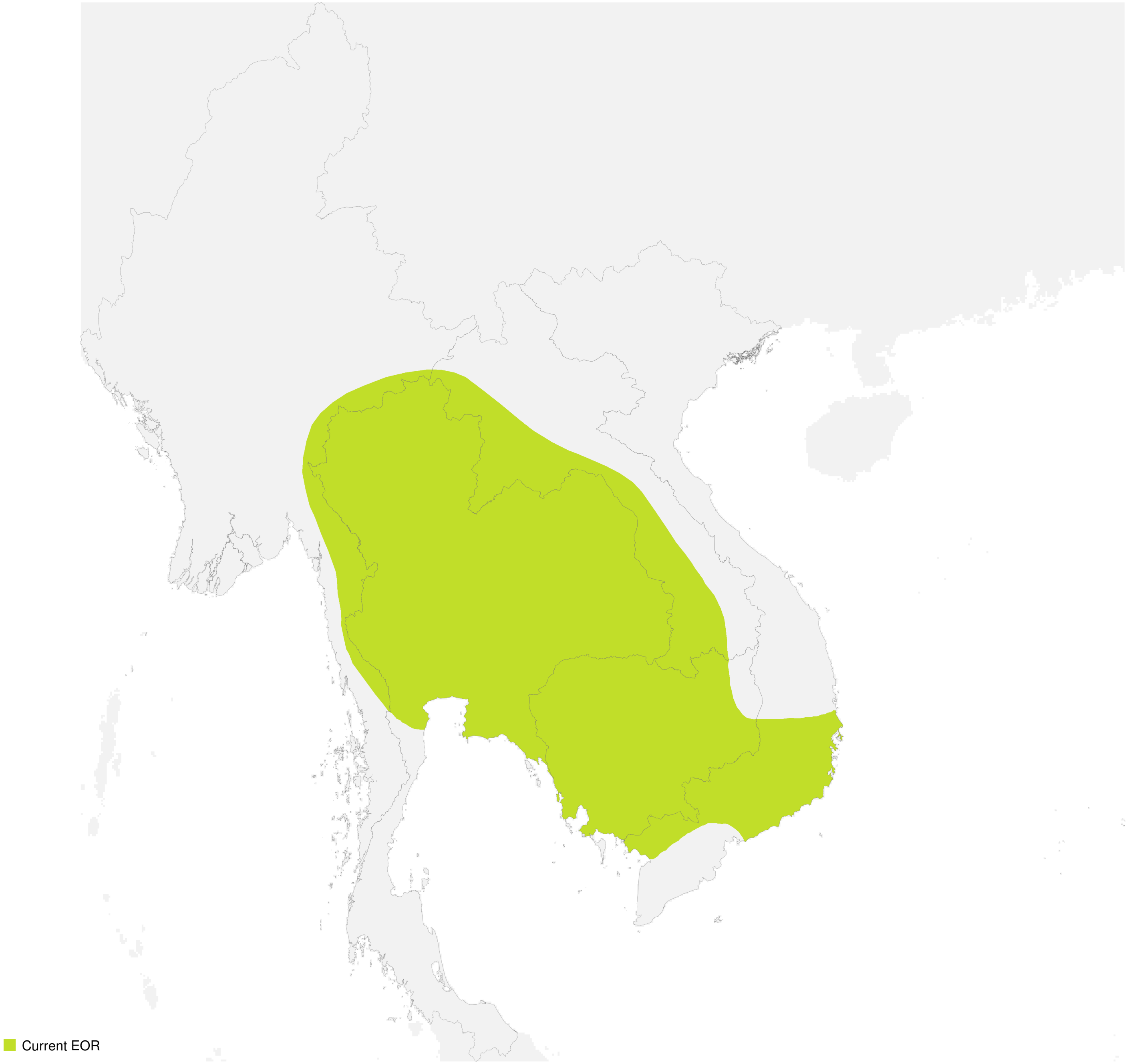

Naja sputatrix

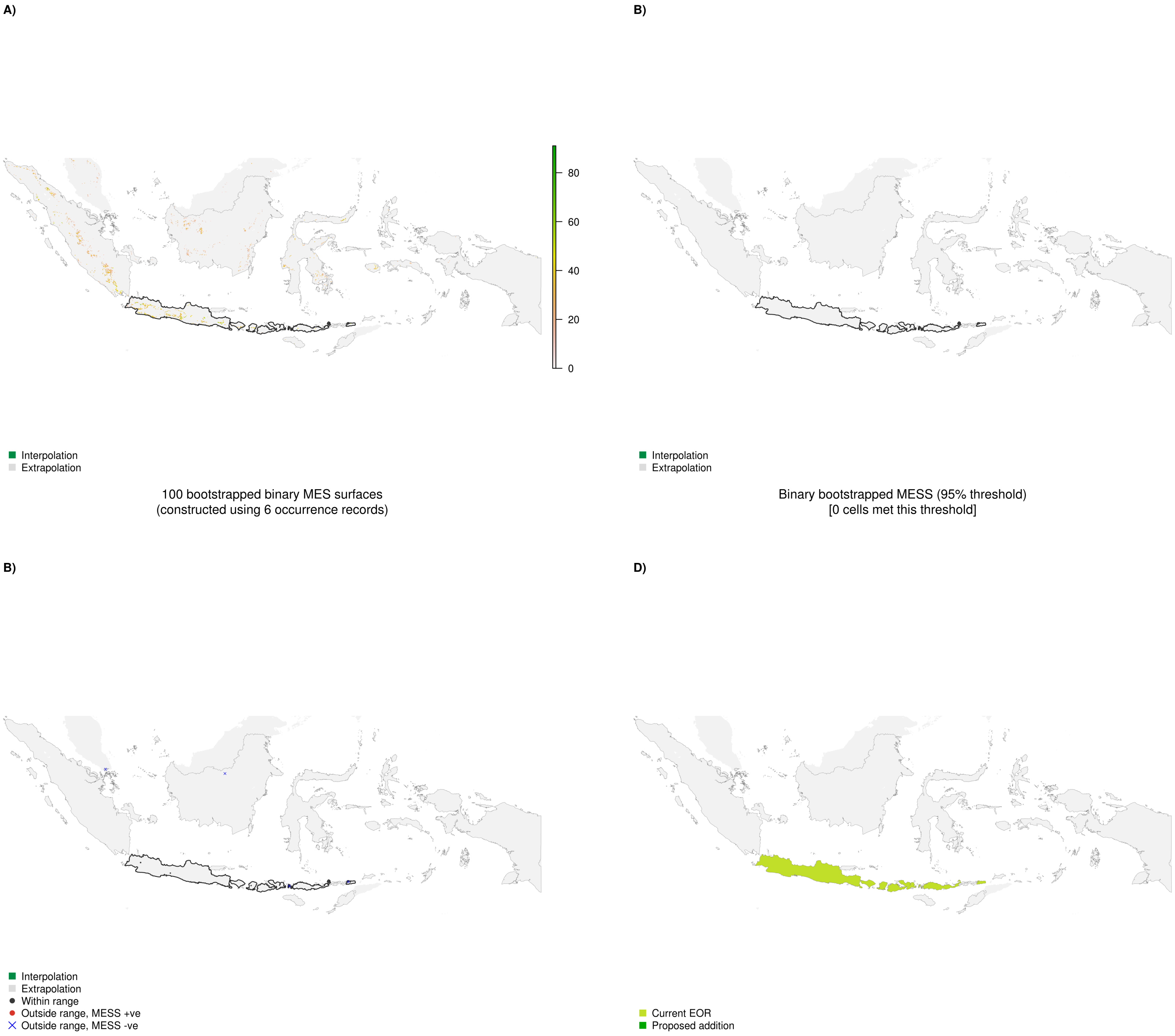

100 bootstrapped binary MES surfaces
(constructed using 6 occurrence records)

Binary bootstrapped MESS (95% threshold)
[0 cells met this threshold]

Binary bootstrapped MESS (95% threshold)
(with 5 outside of range occurrence records)

Suggested amended range
(incorporating 0 outside of range MESS +ve records)

Naja sumatrana

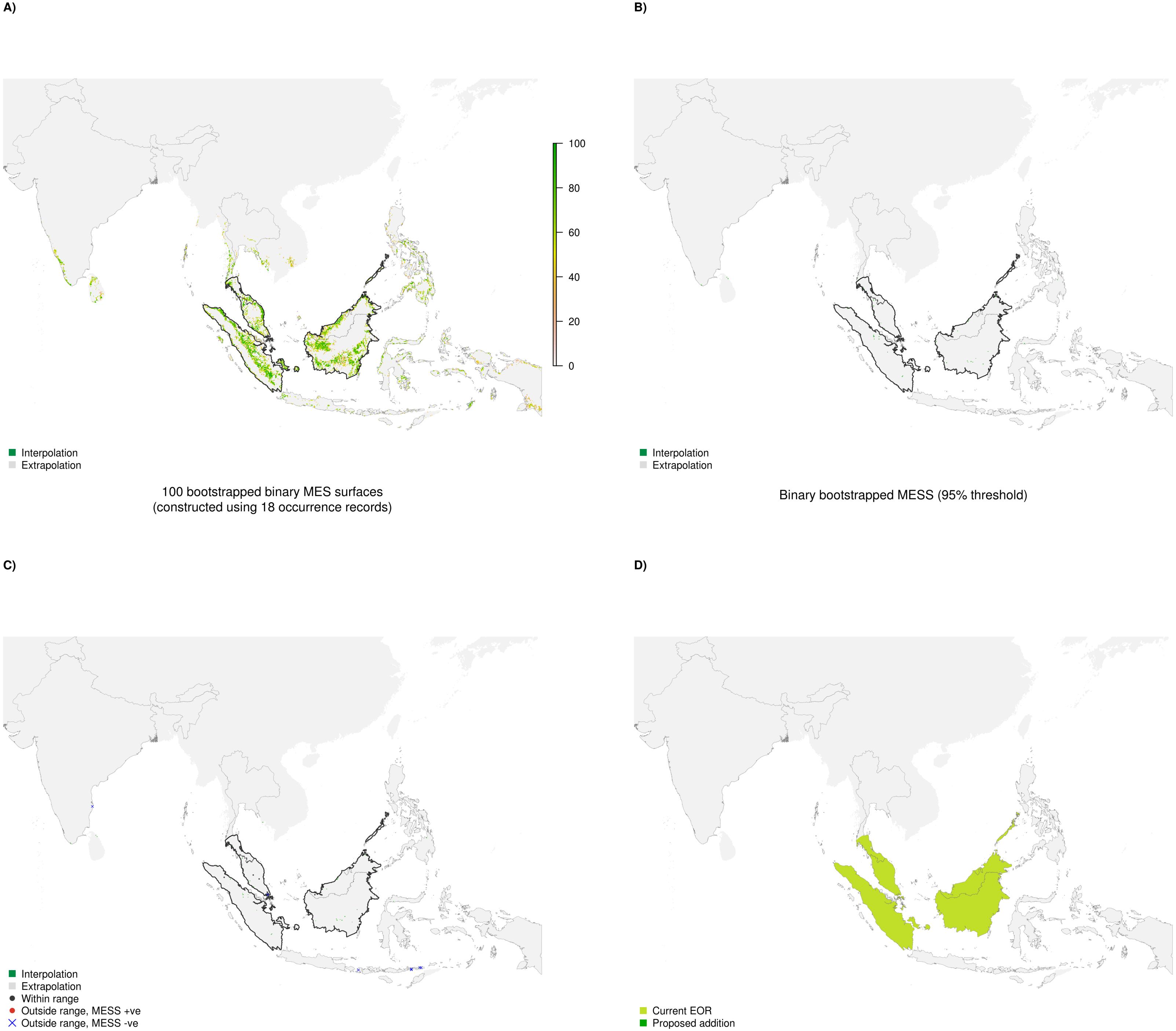

A) 100 bootstrapped binary MES surfaces (constructed using 18 occurrence records)

B) Binary bootstrapped MESS (95% threshold)

C) Binary bootstrapped MESS (95% threshold) (with 7 outside of range occurrence records)

D) Suggested amended range (incorporating 0 outside of range MESS +ve records)

Notechis scutatus

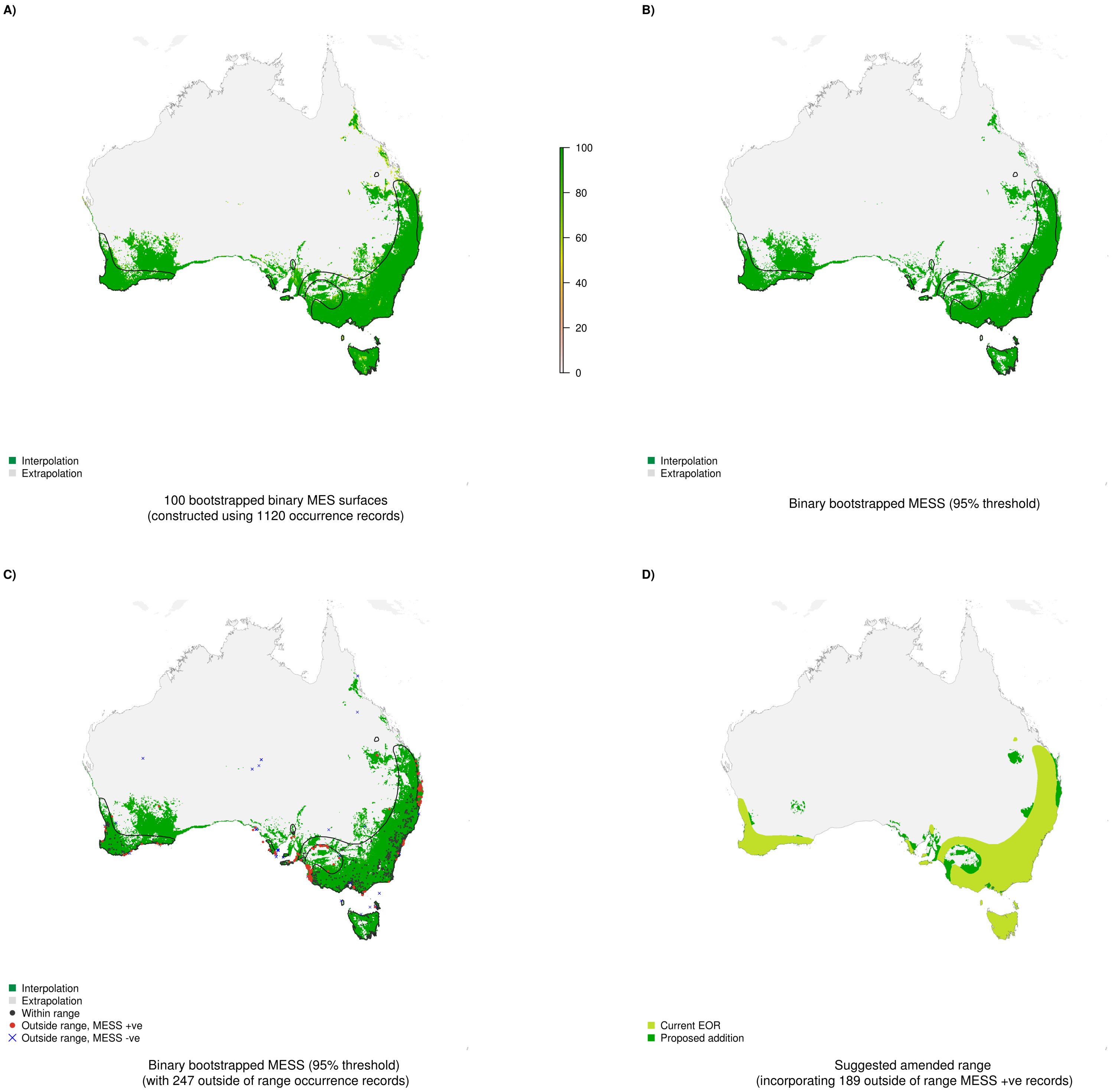

Ophiophagus hannah

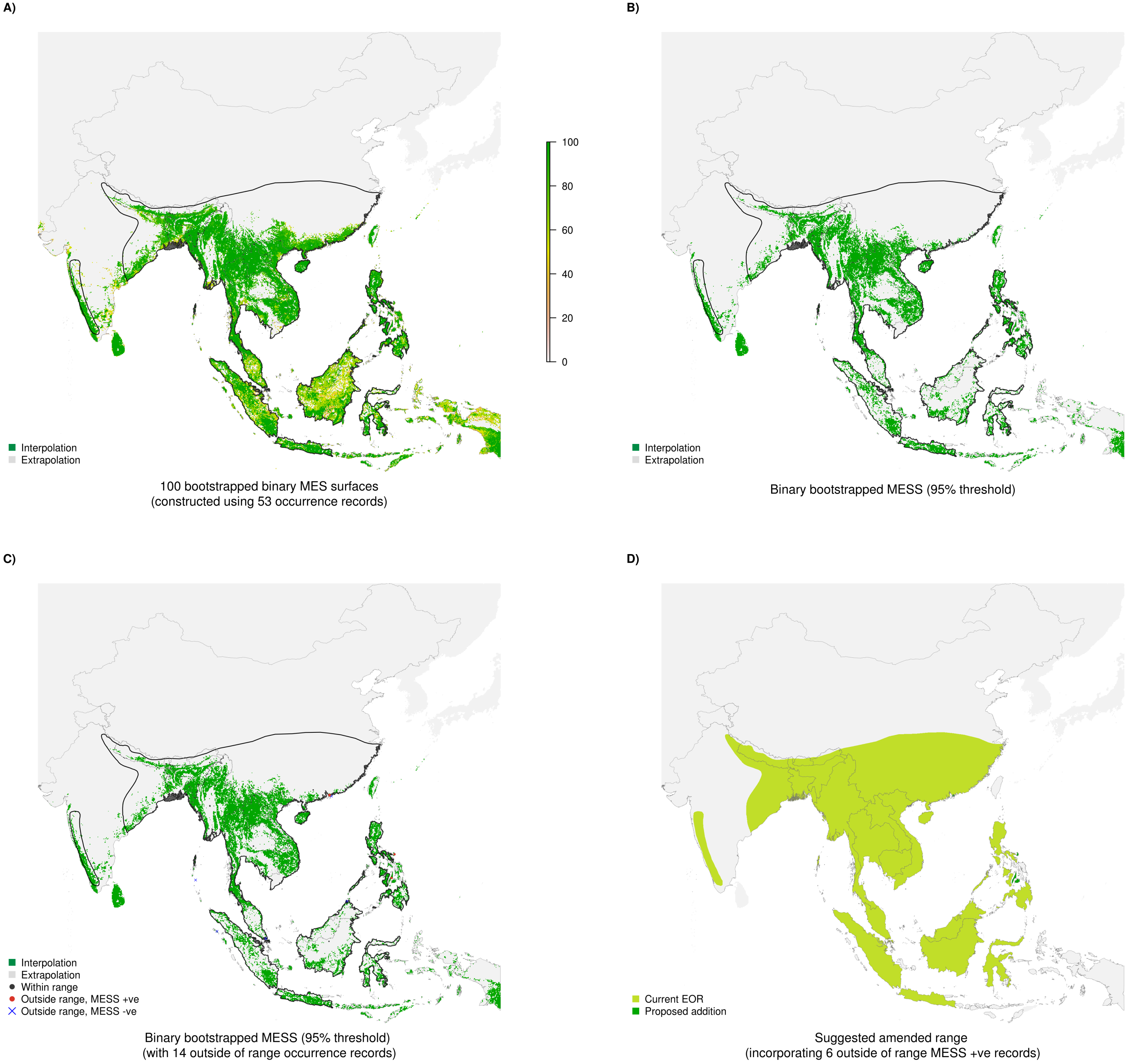

Ophryacus melanurus

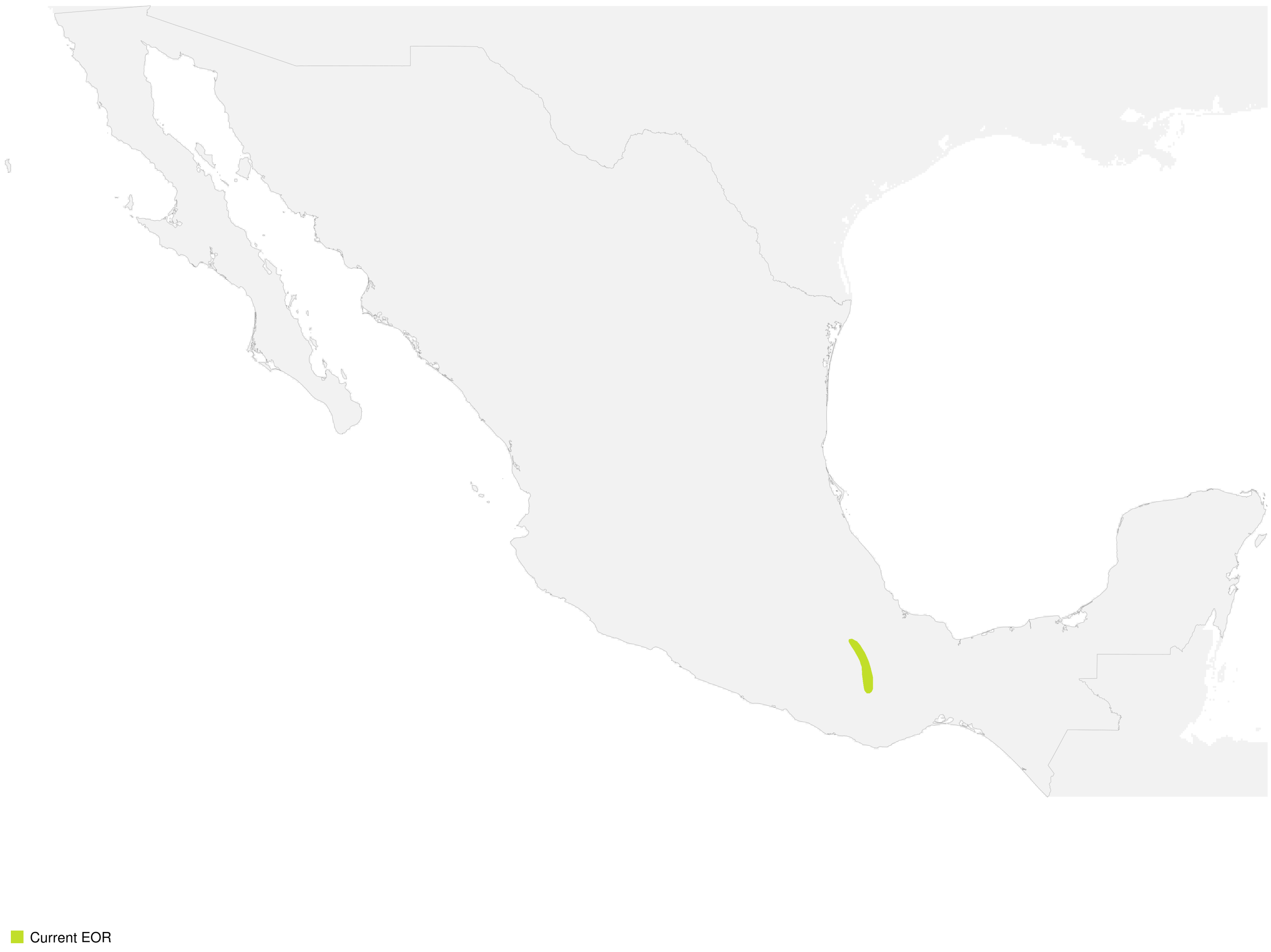

Ophryacus undulatus

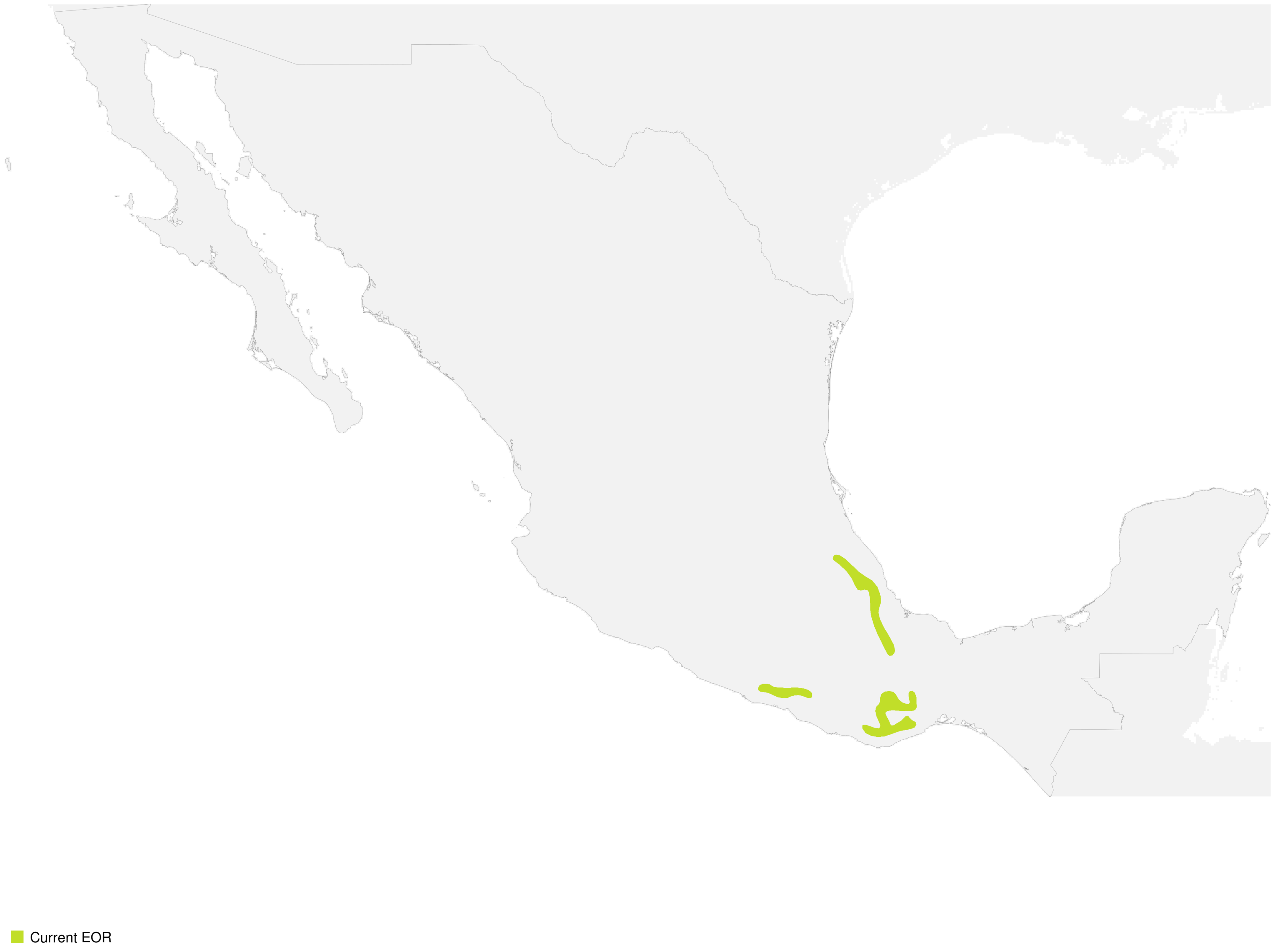

Oxyuranus microlepidotus

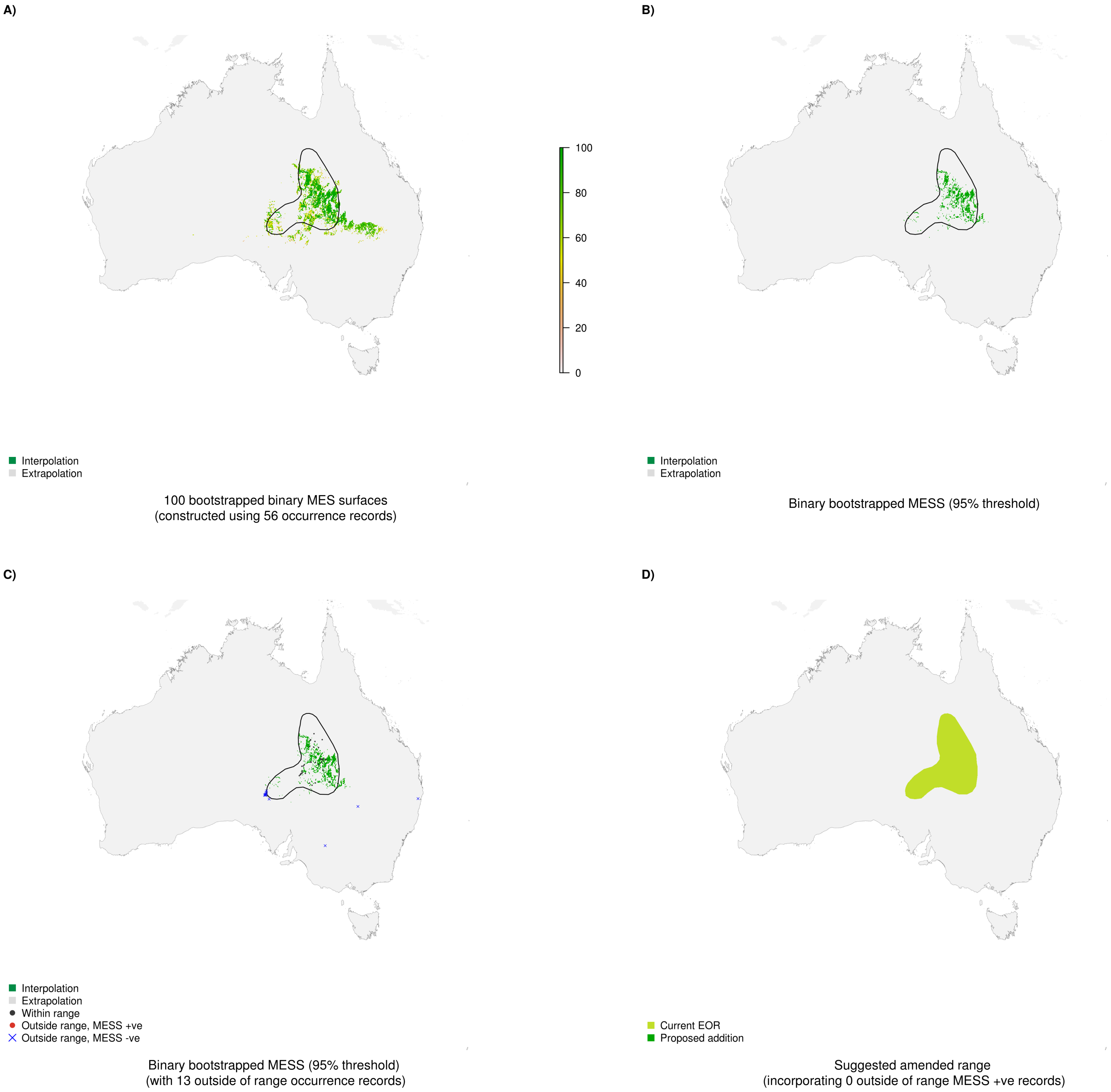

Oxyuranus scutellatus

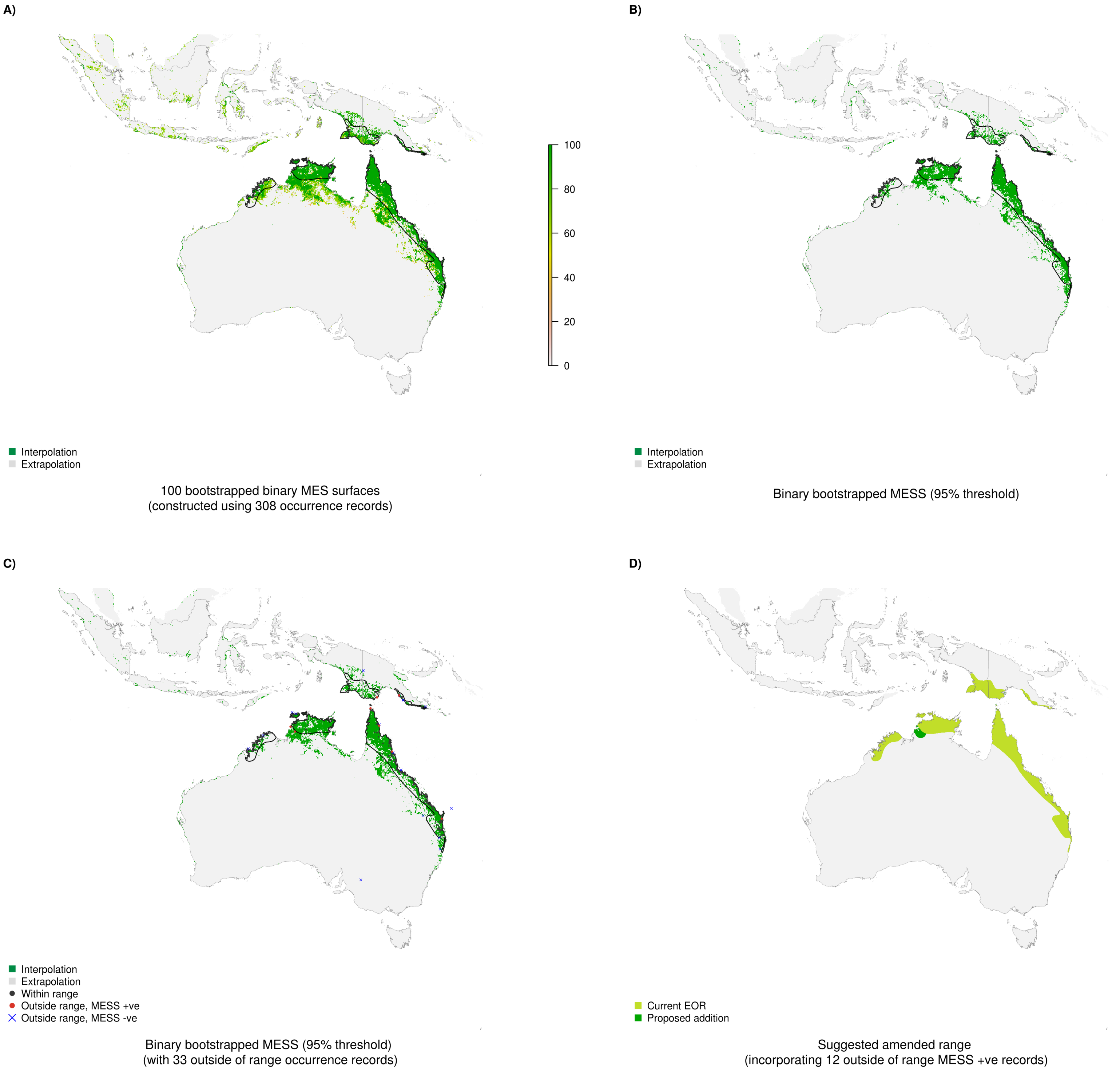

Oxyuranus temporalis

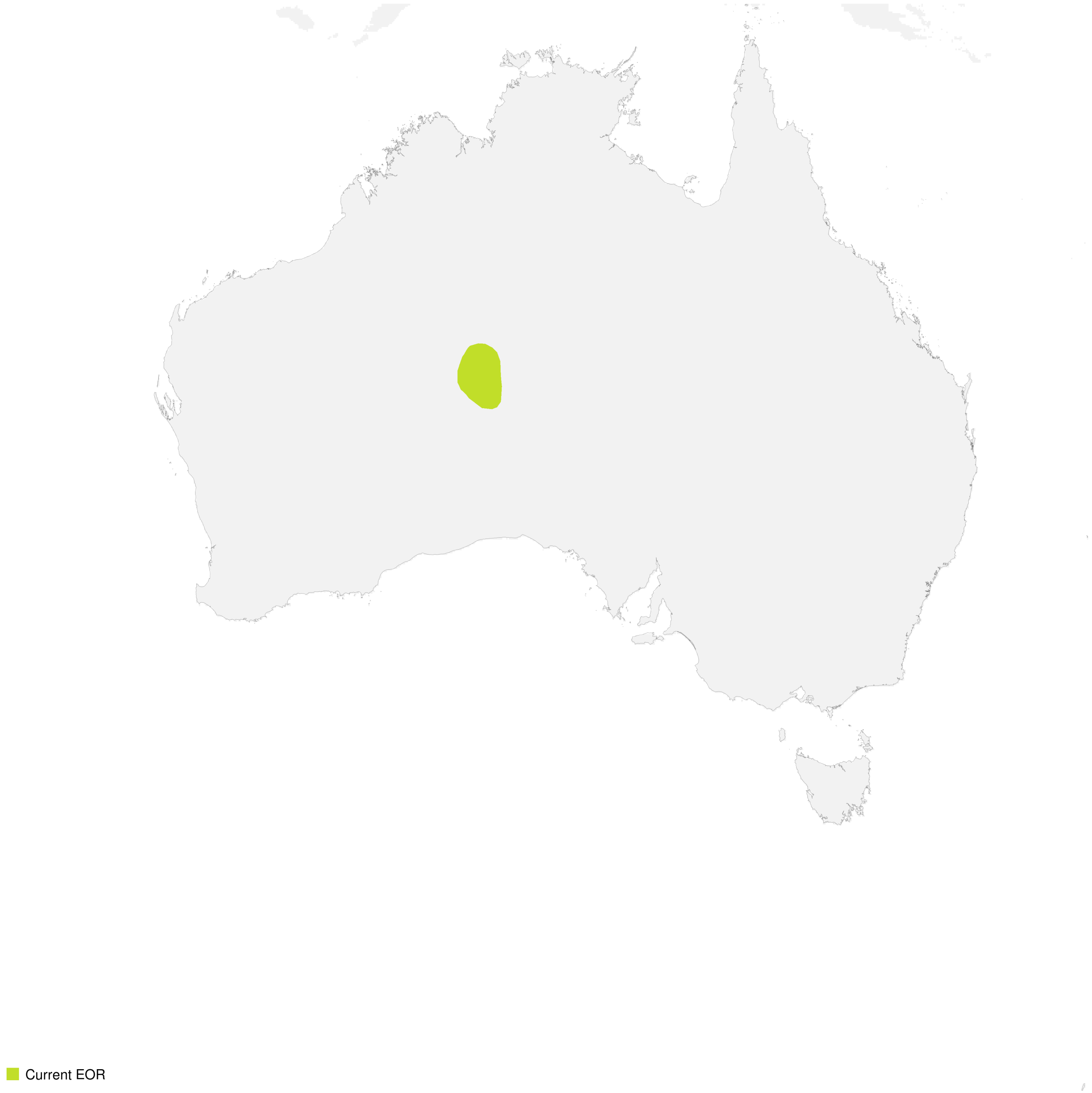

Parias flavomaculatus

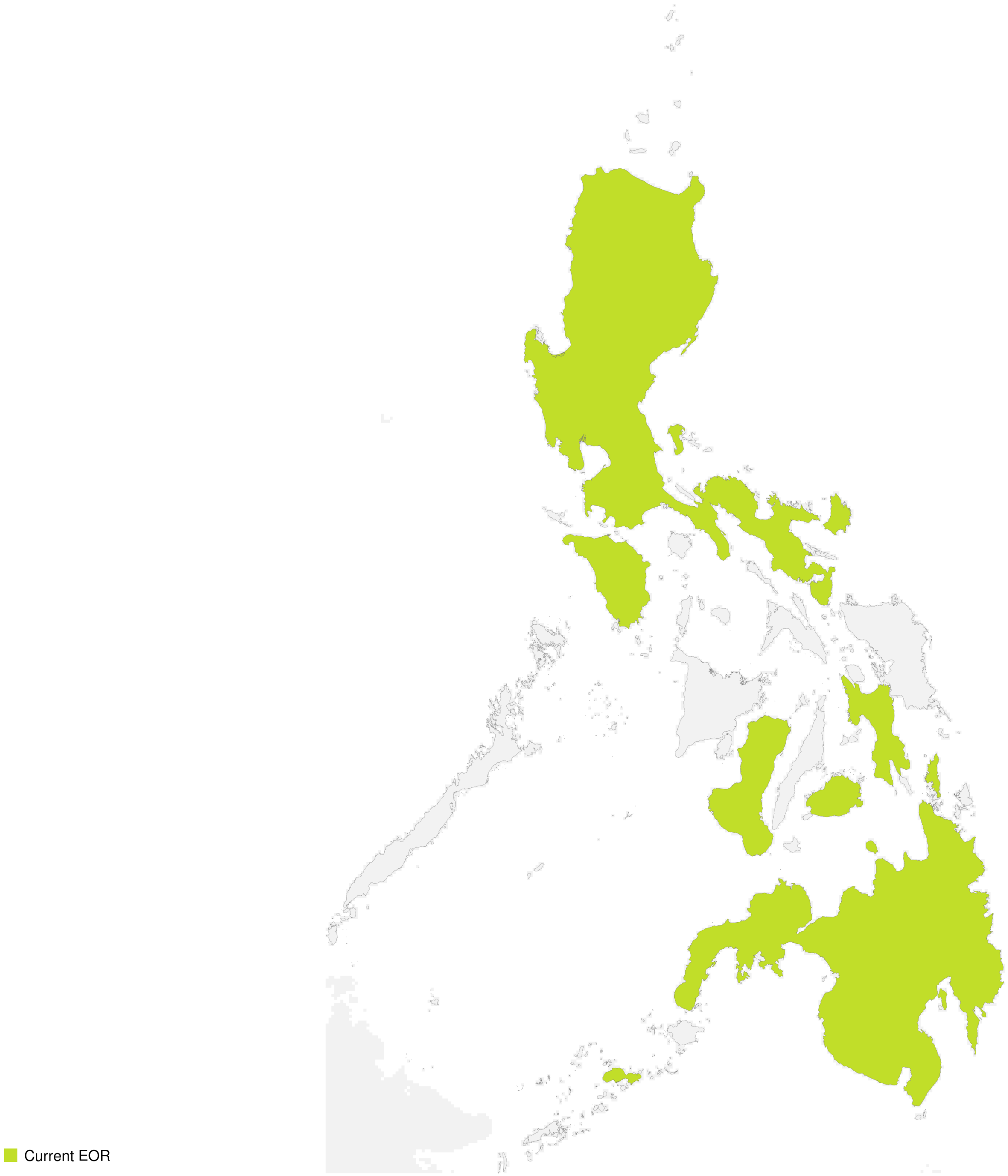

Parias sumatranus

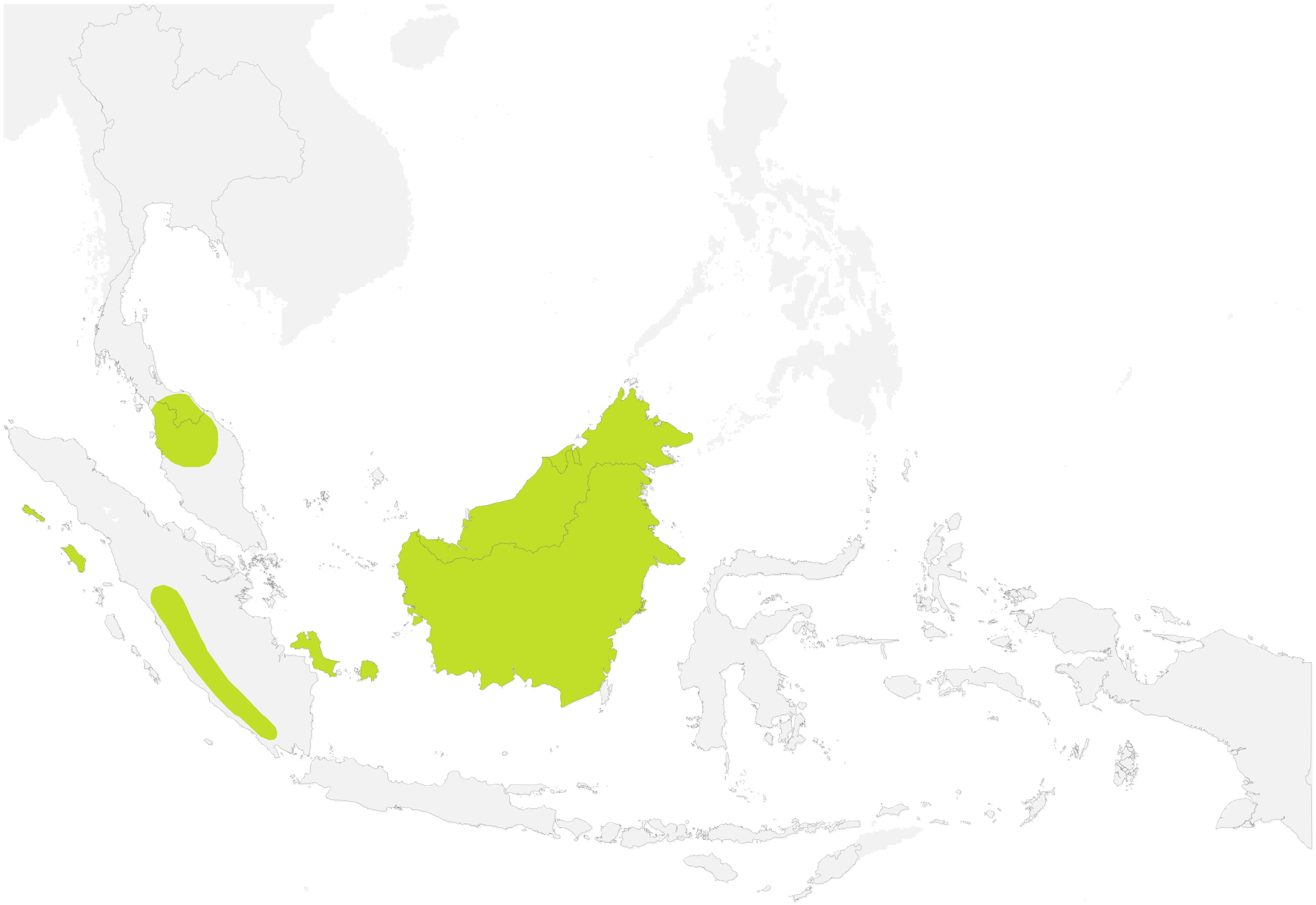

Current EOR

Porthidium arcosae

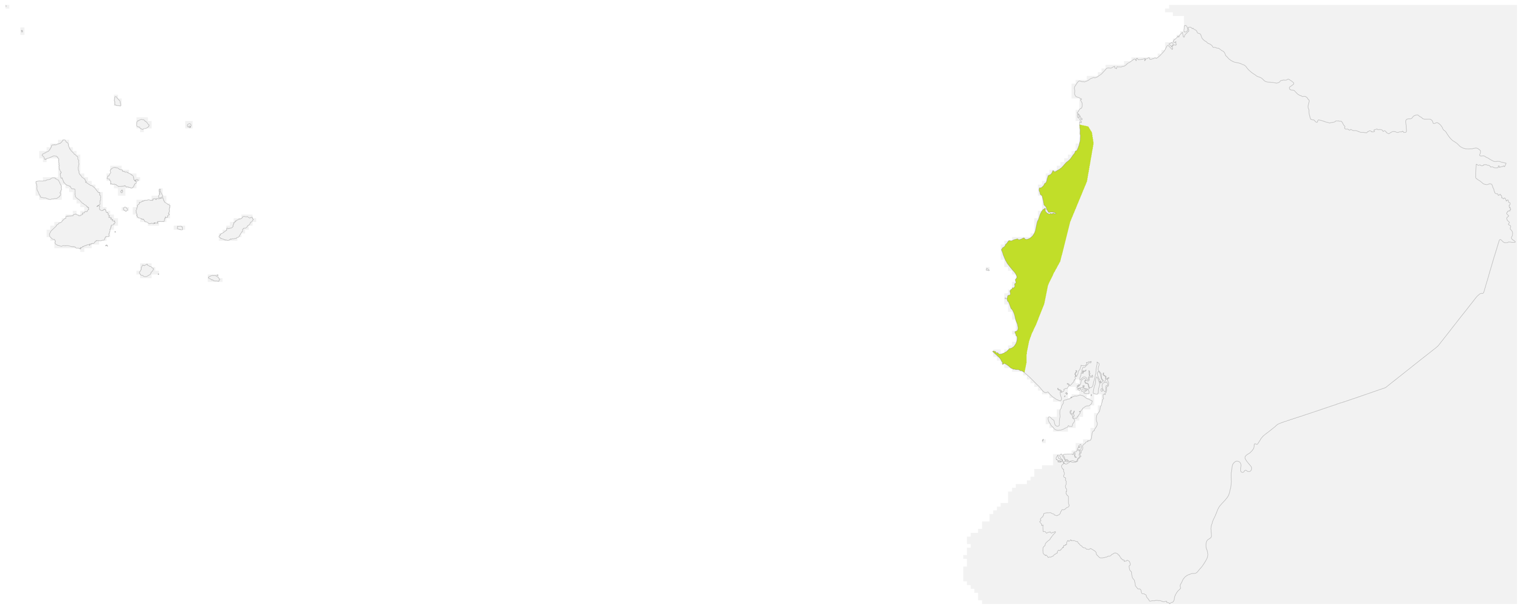

Porthidium dunni

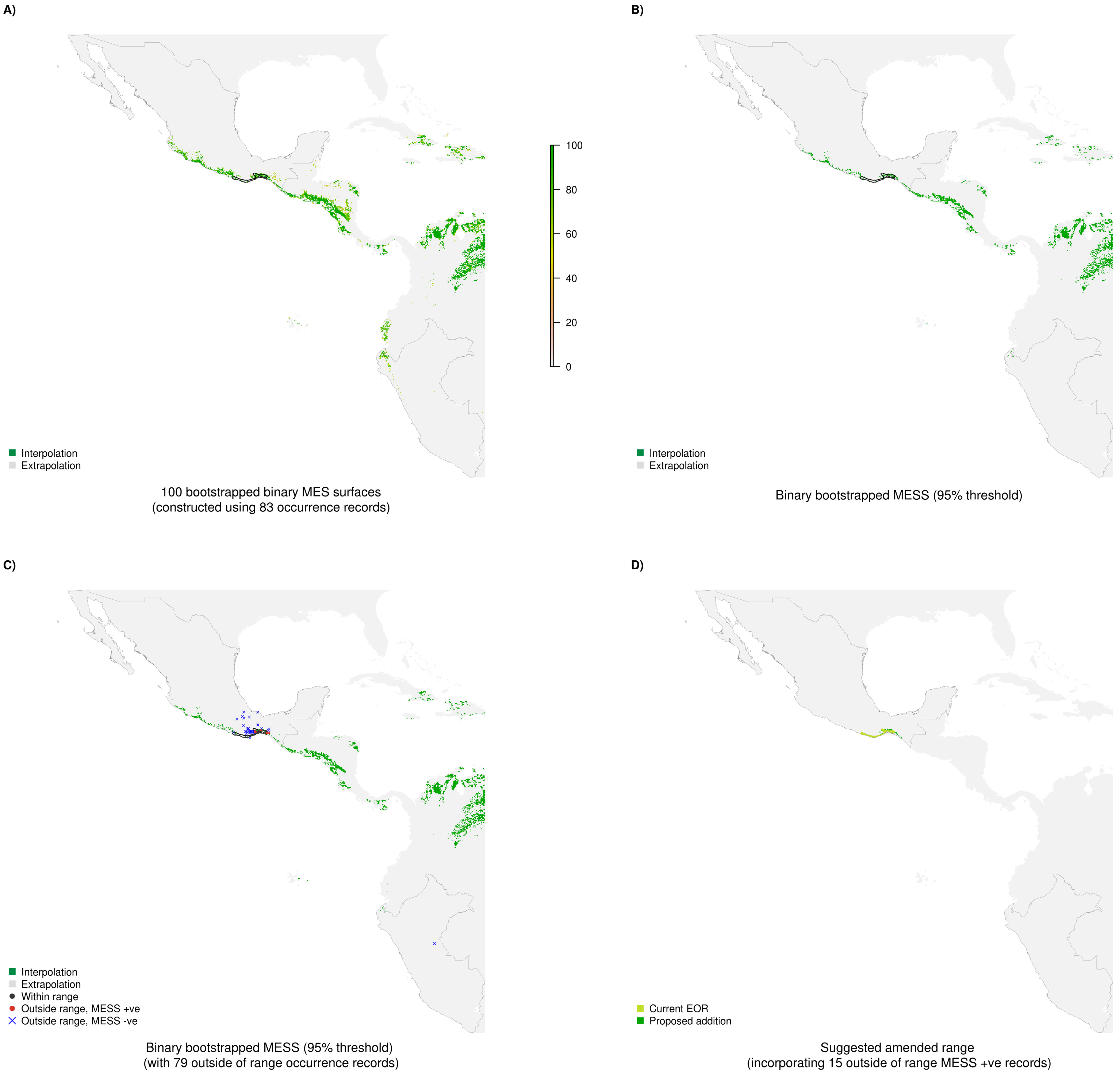

Porthidium hespere

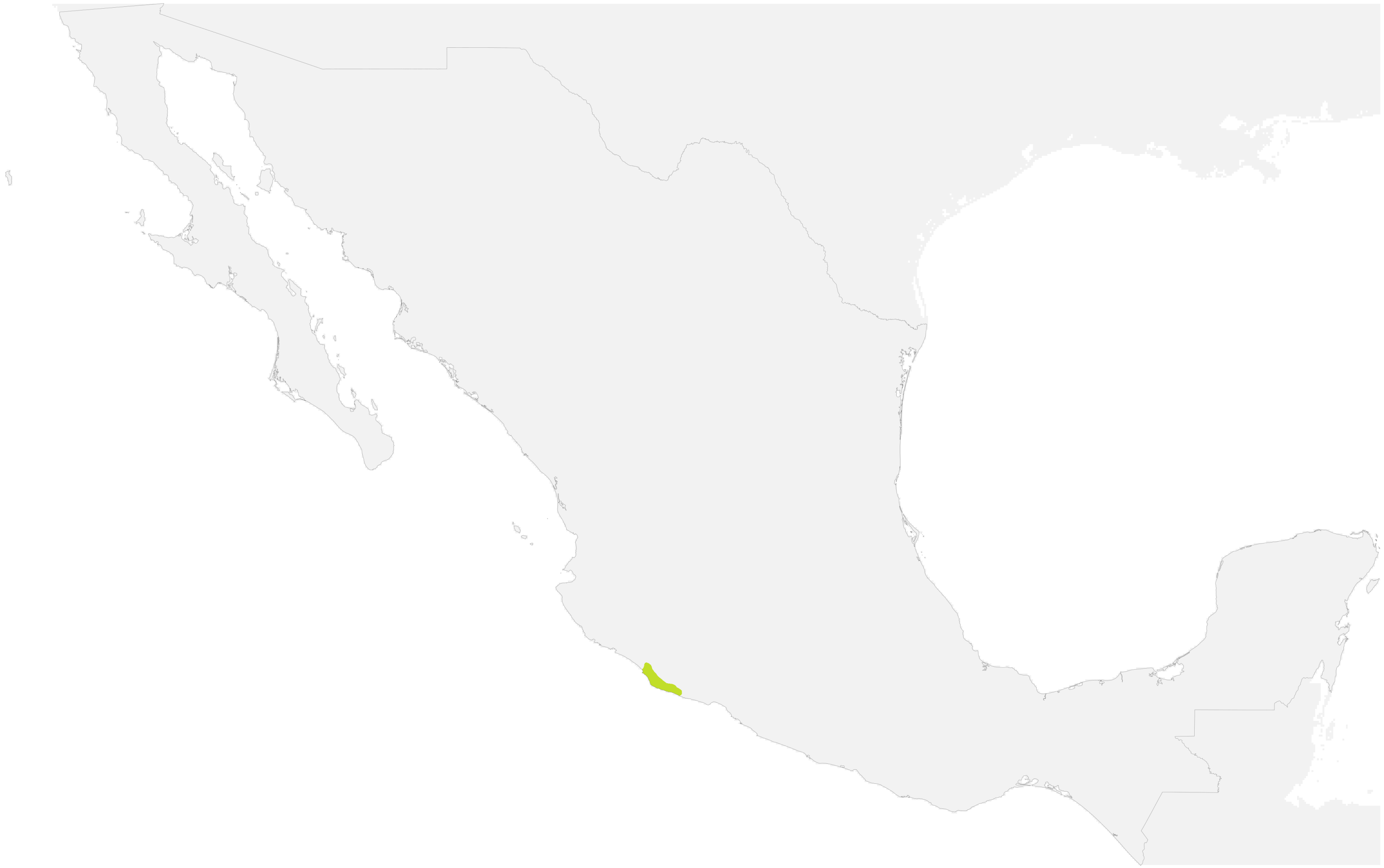

Current EOR

Porthidium lansbergii

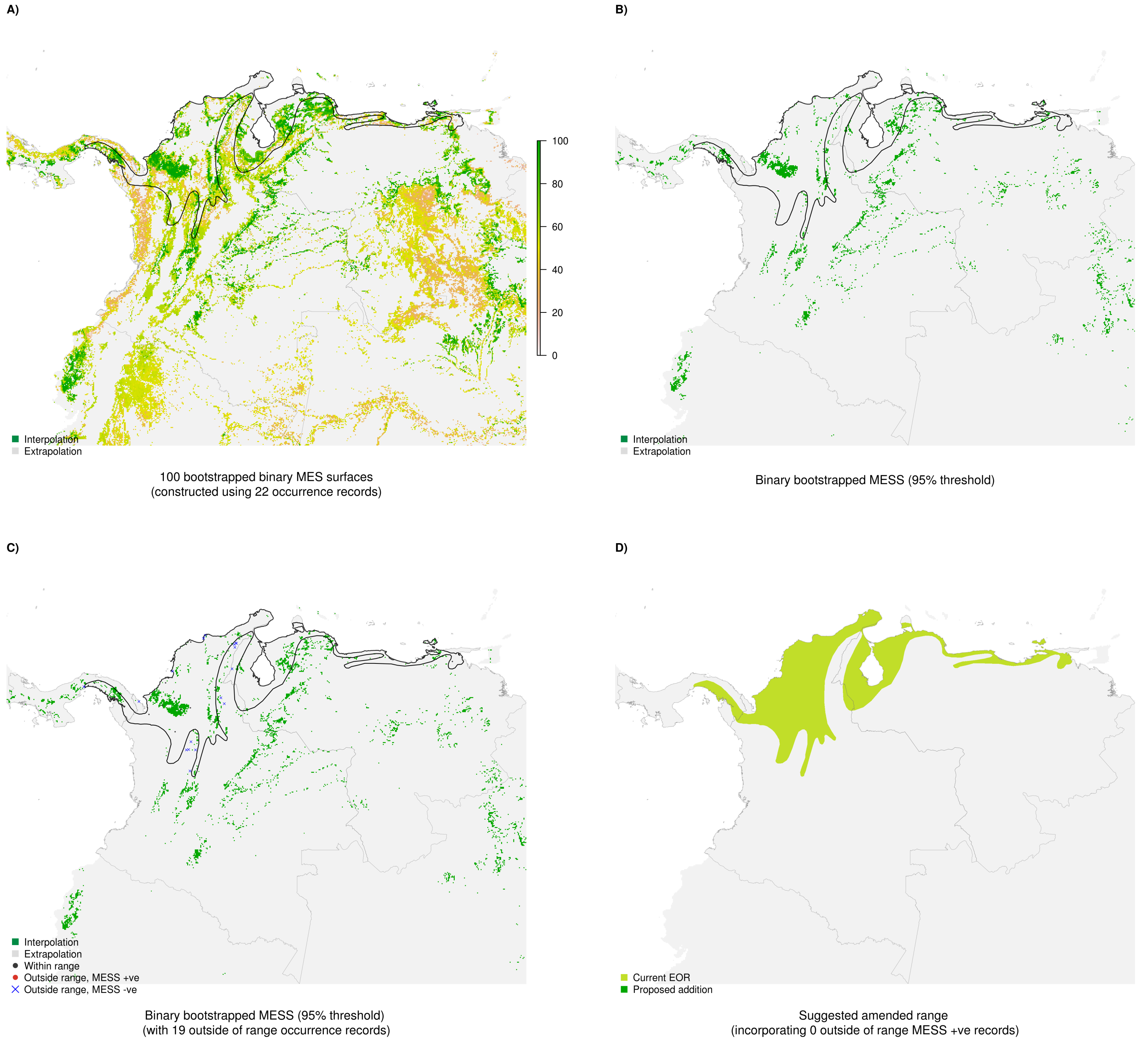

Porthidium nasutum

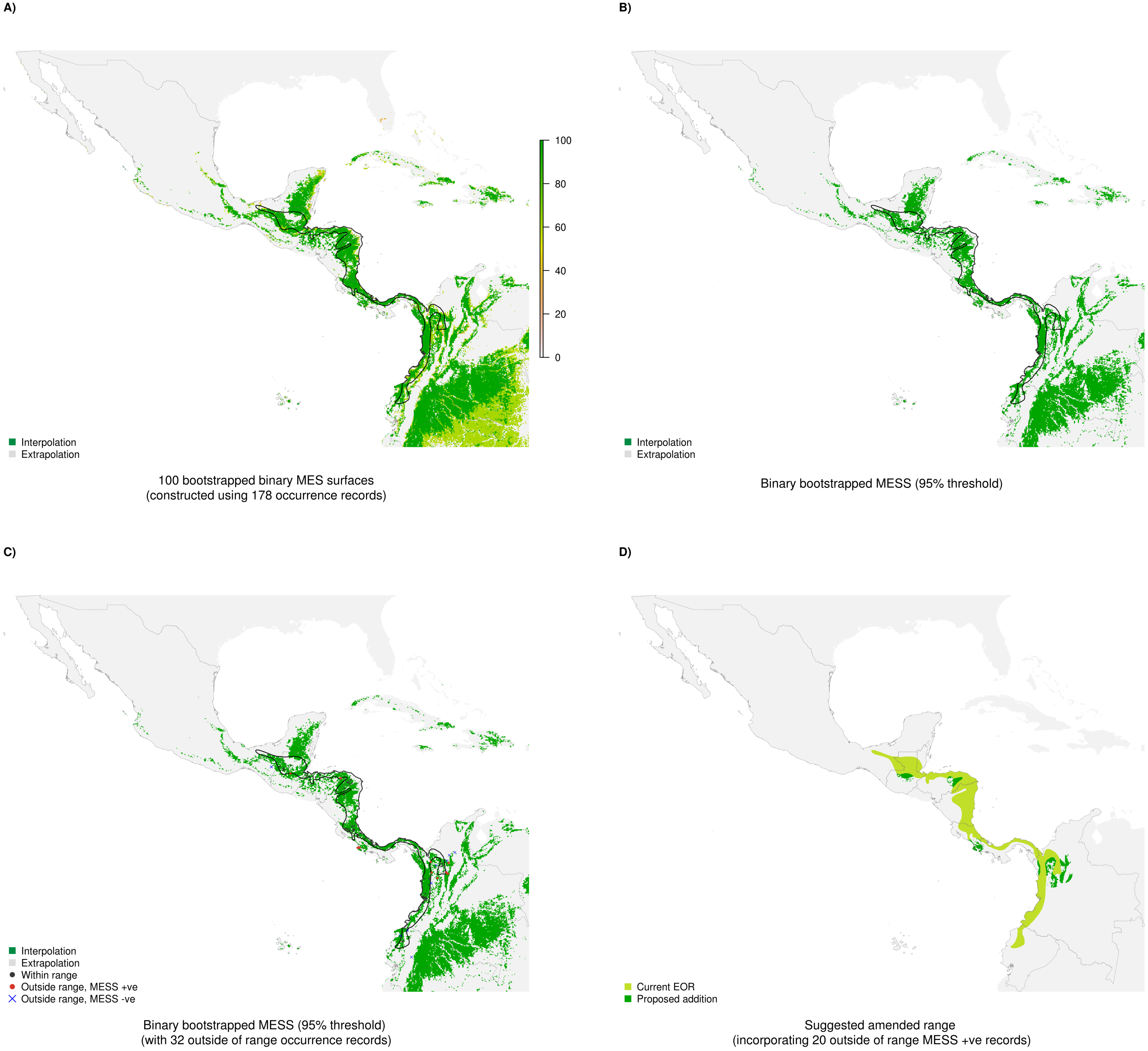

Porthidium ophryomegas

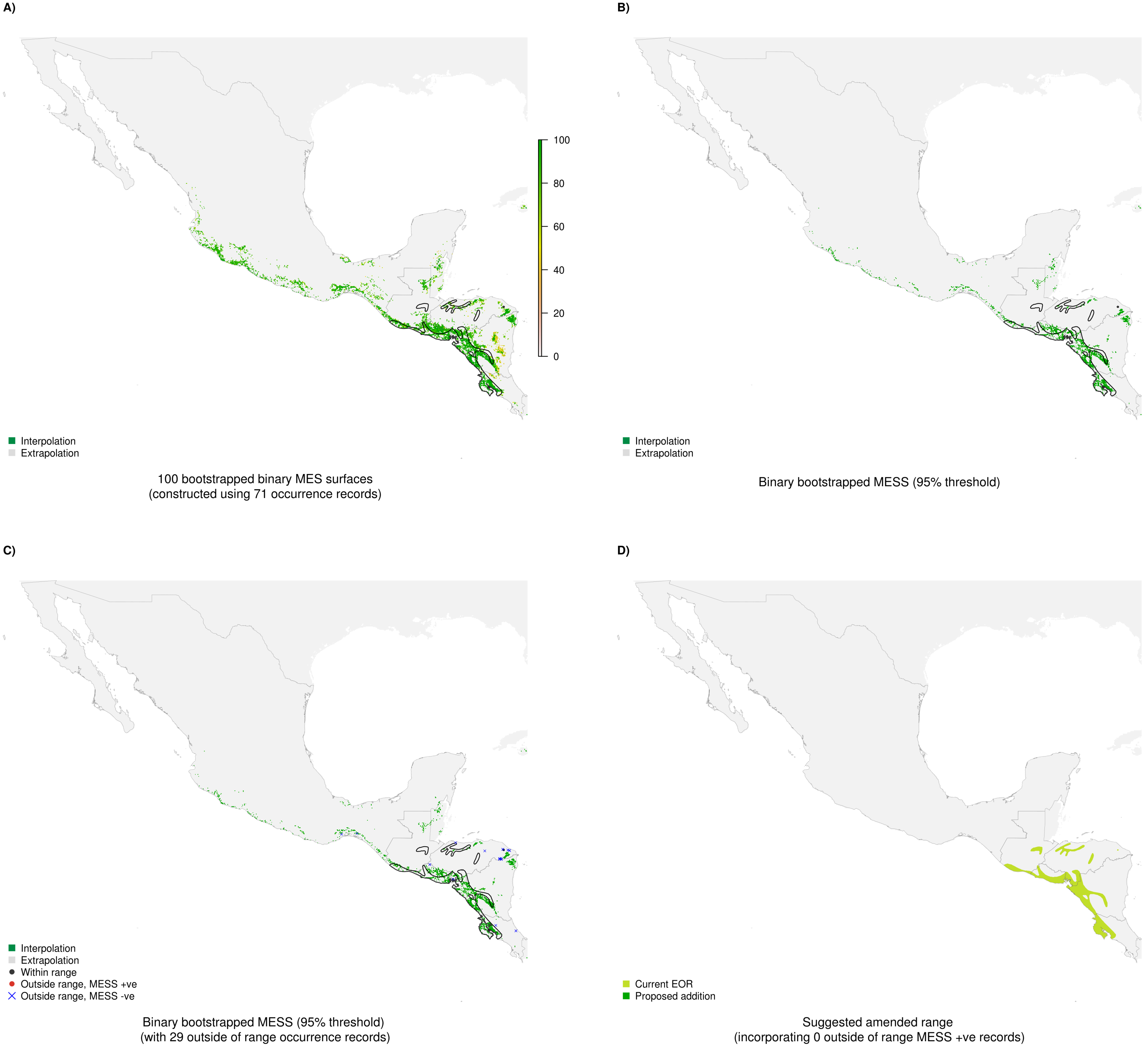

Porthidium porrasi

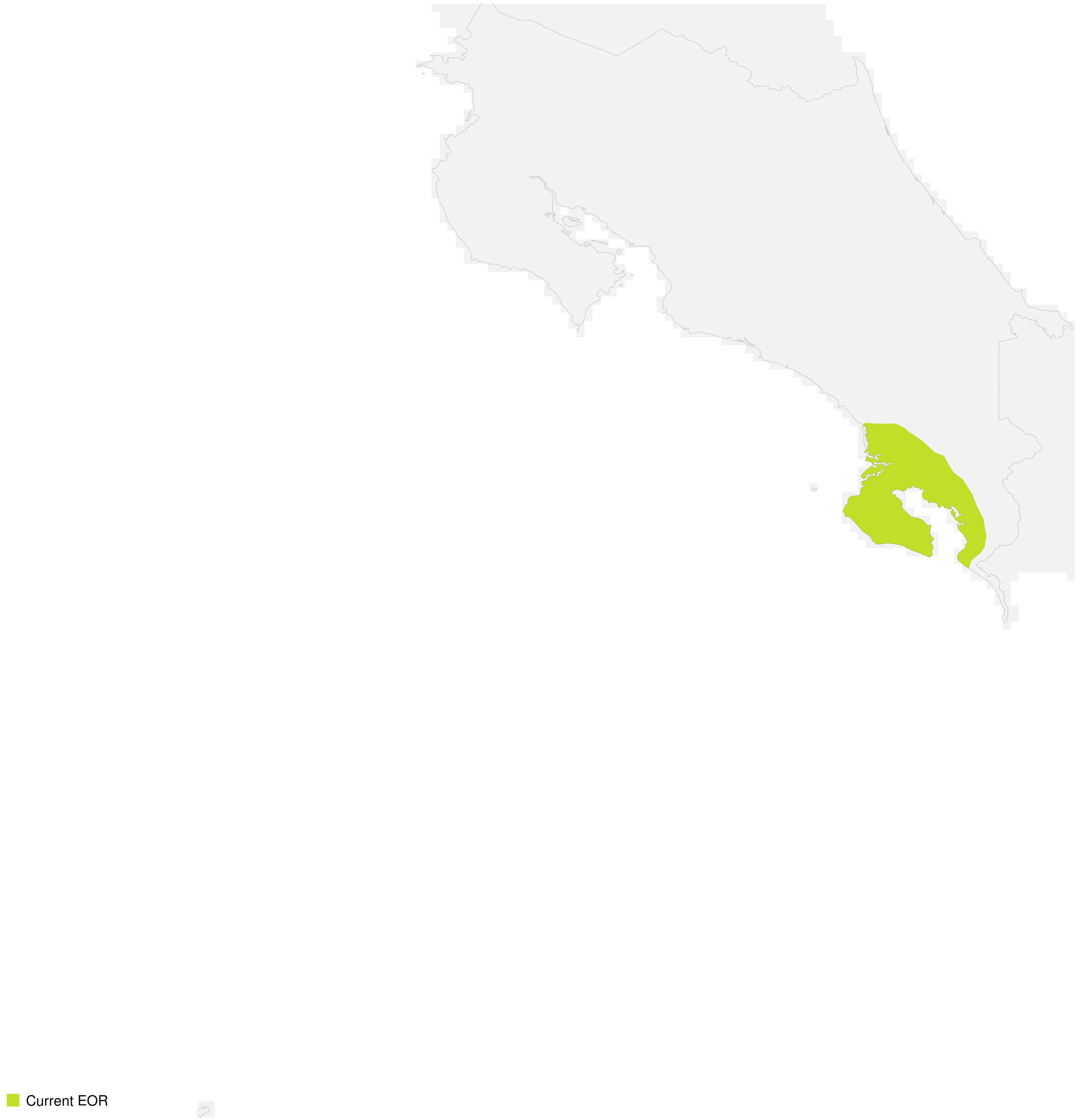

Porthidium volcanicum

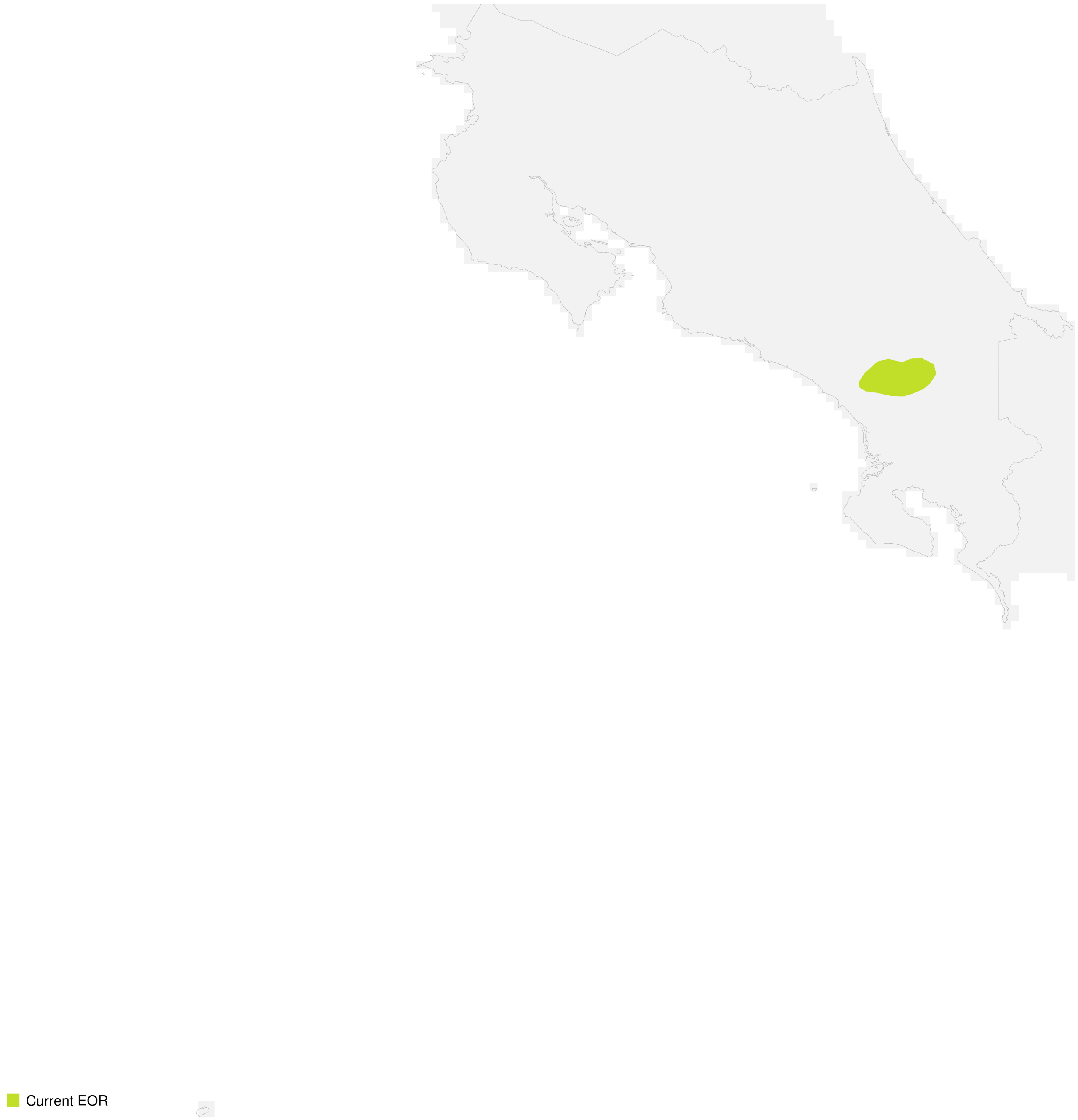

Porthidium yucatanicum

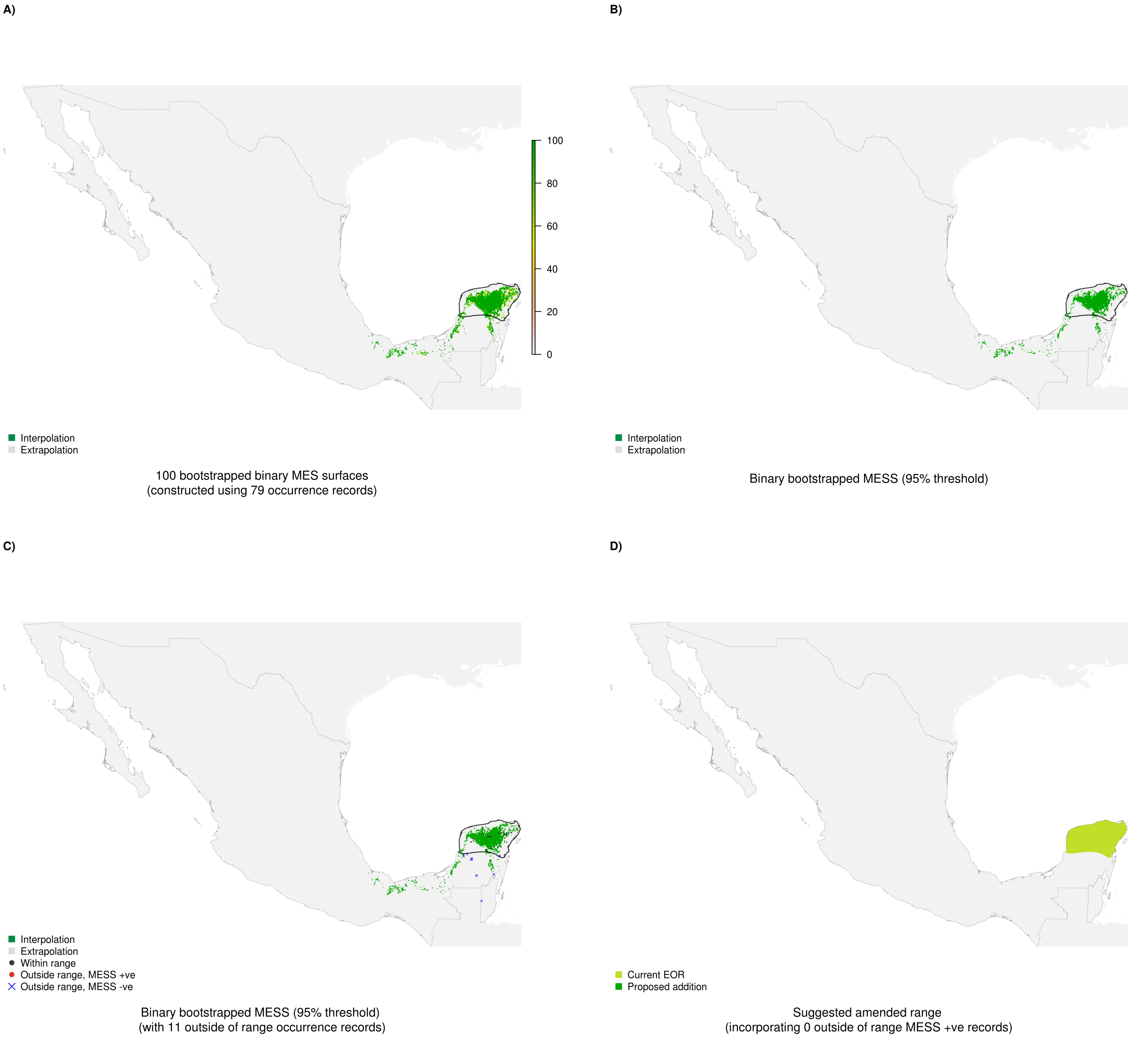

Proatheris superciliaris

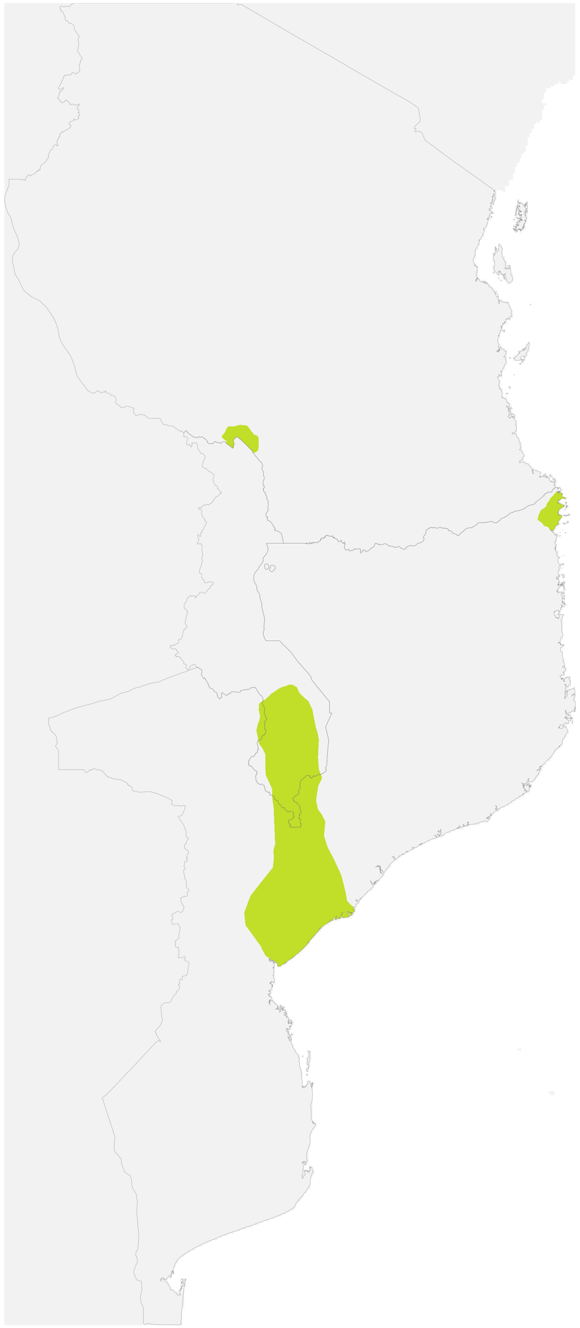

Current EOR

Protobothrops elegans

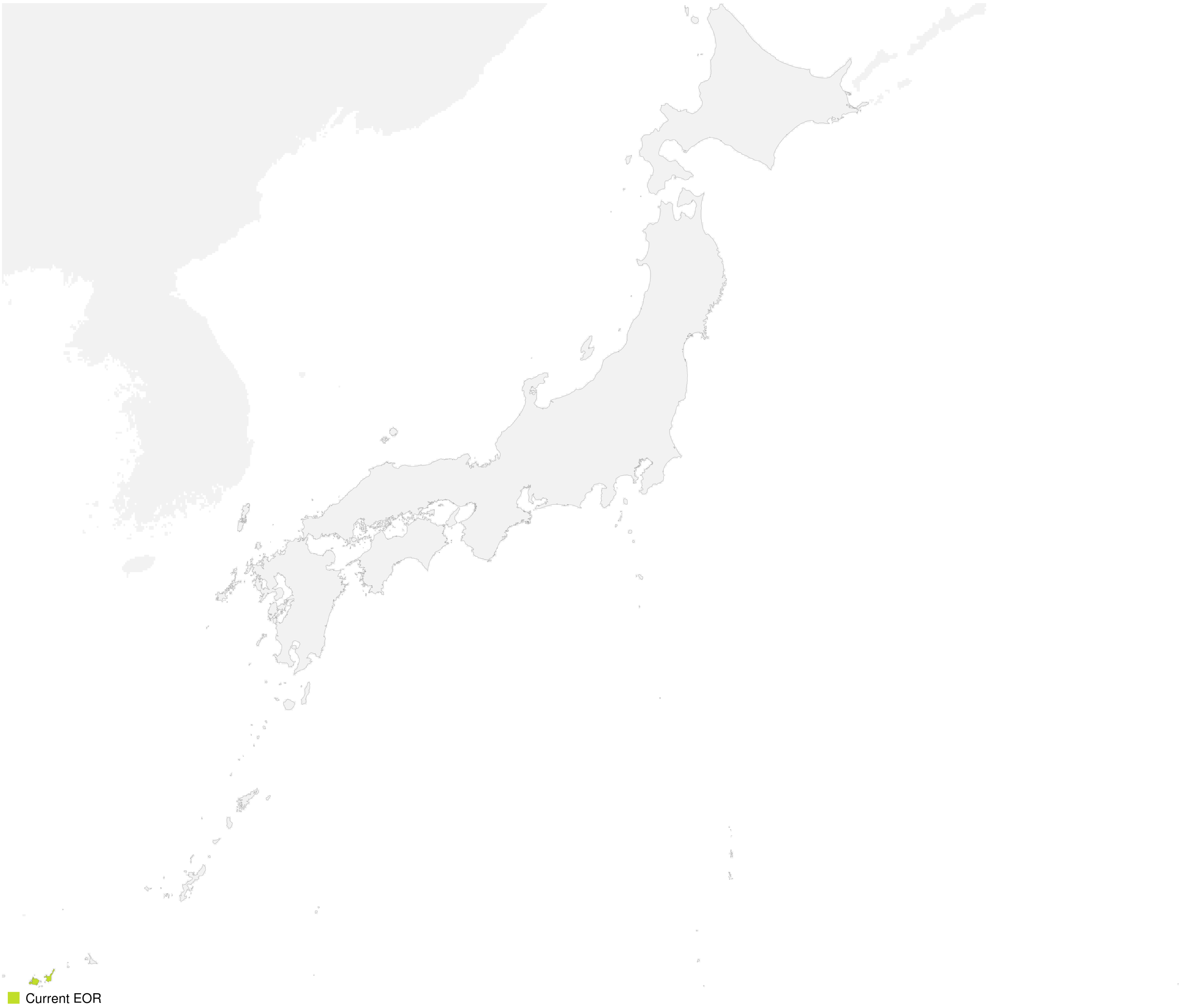

Protobothrops flavoviridis

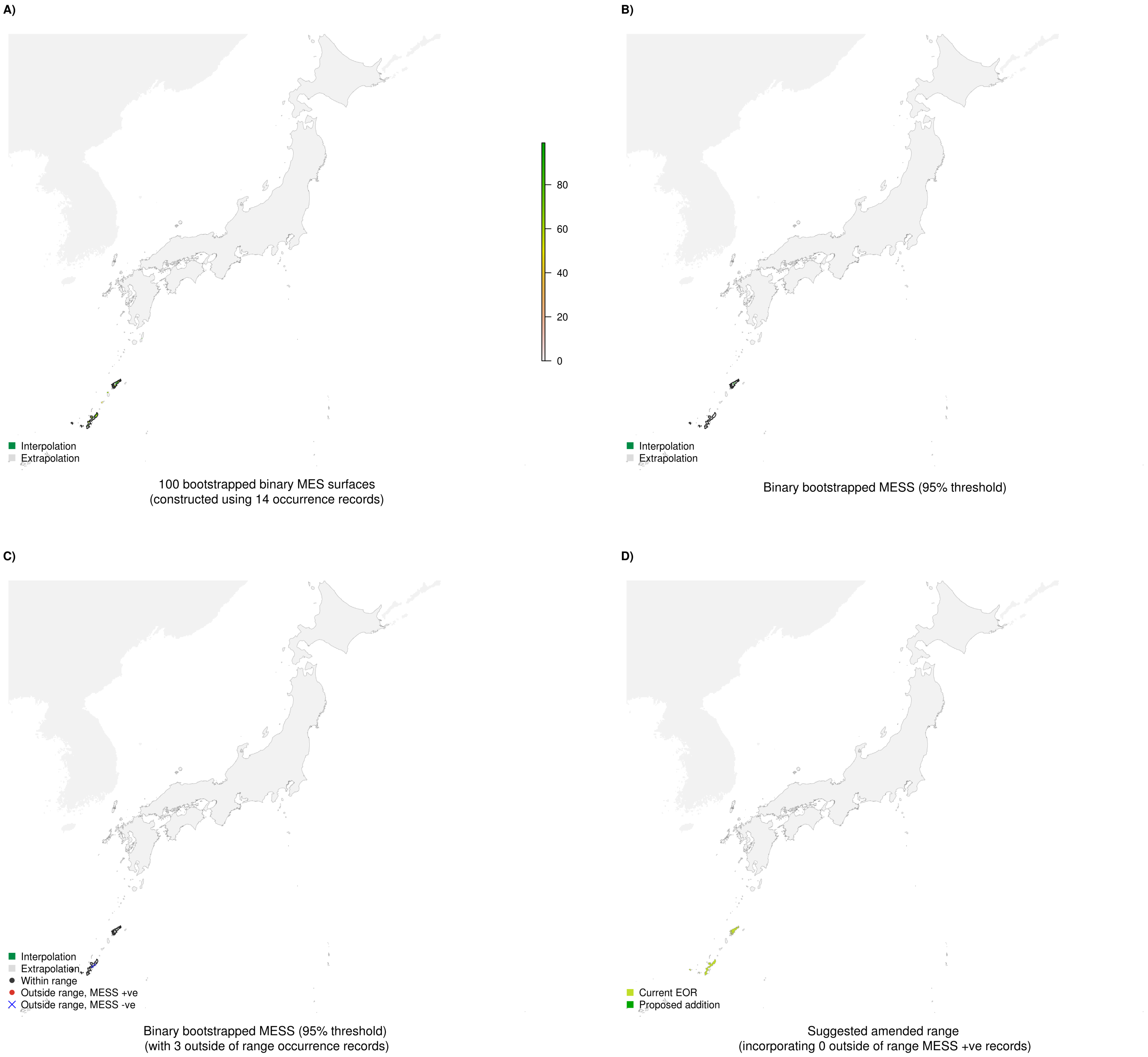

Protobothrops jerdonii

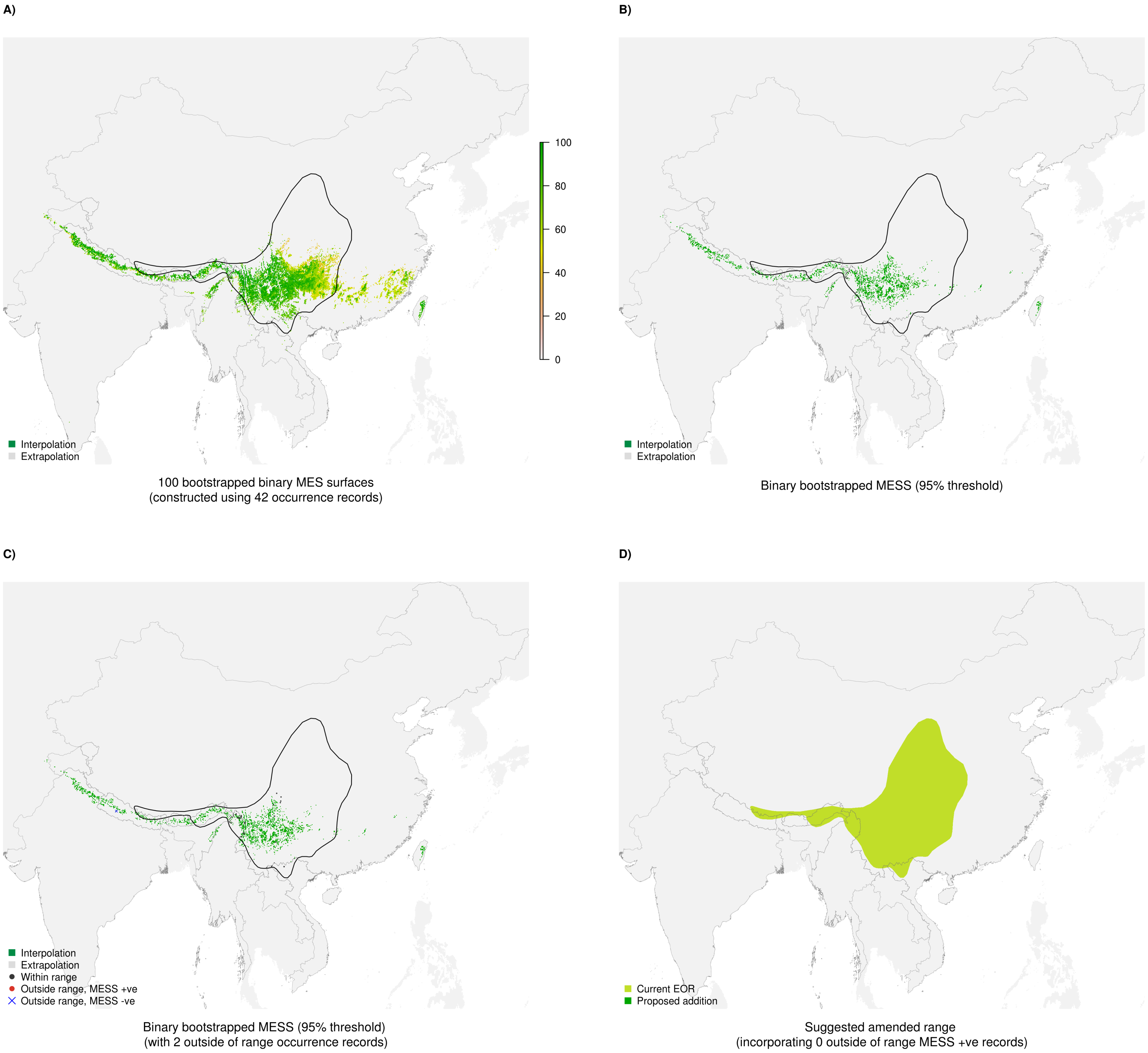

Protobothrops kaulbacki

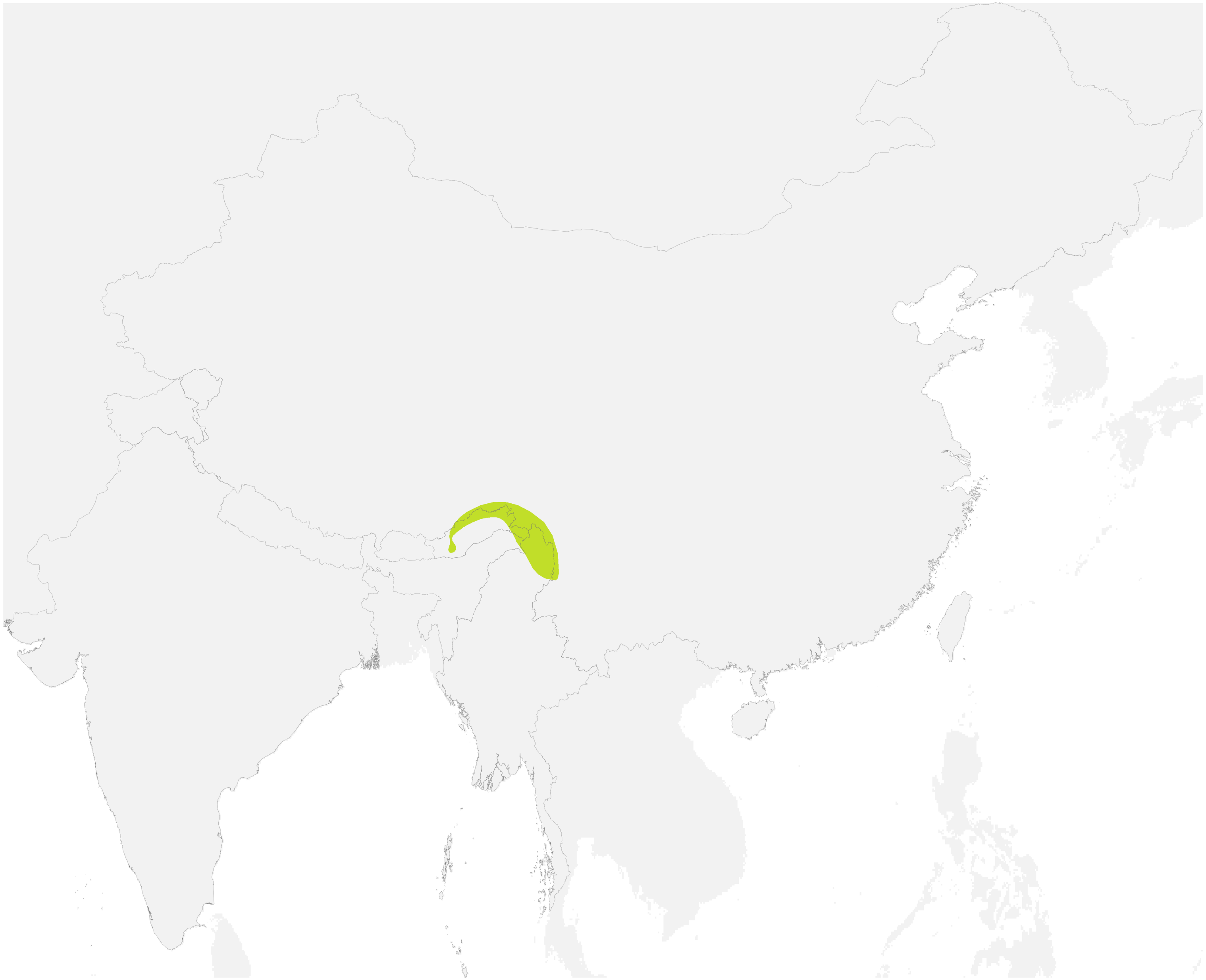

Current EOR

Protobothrops mangshanensis

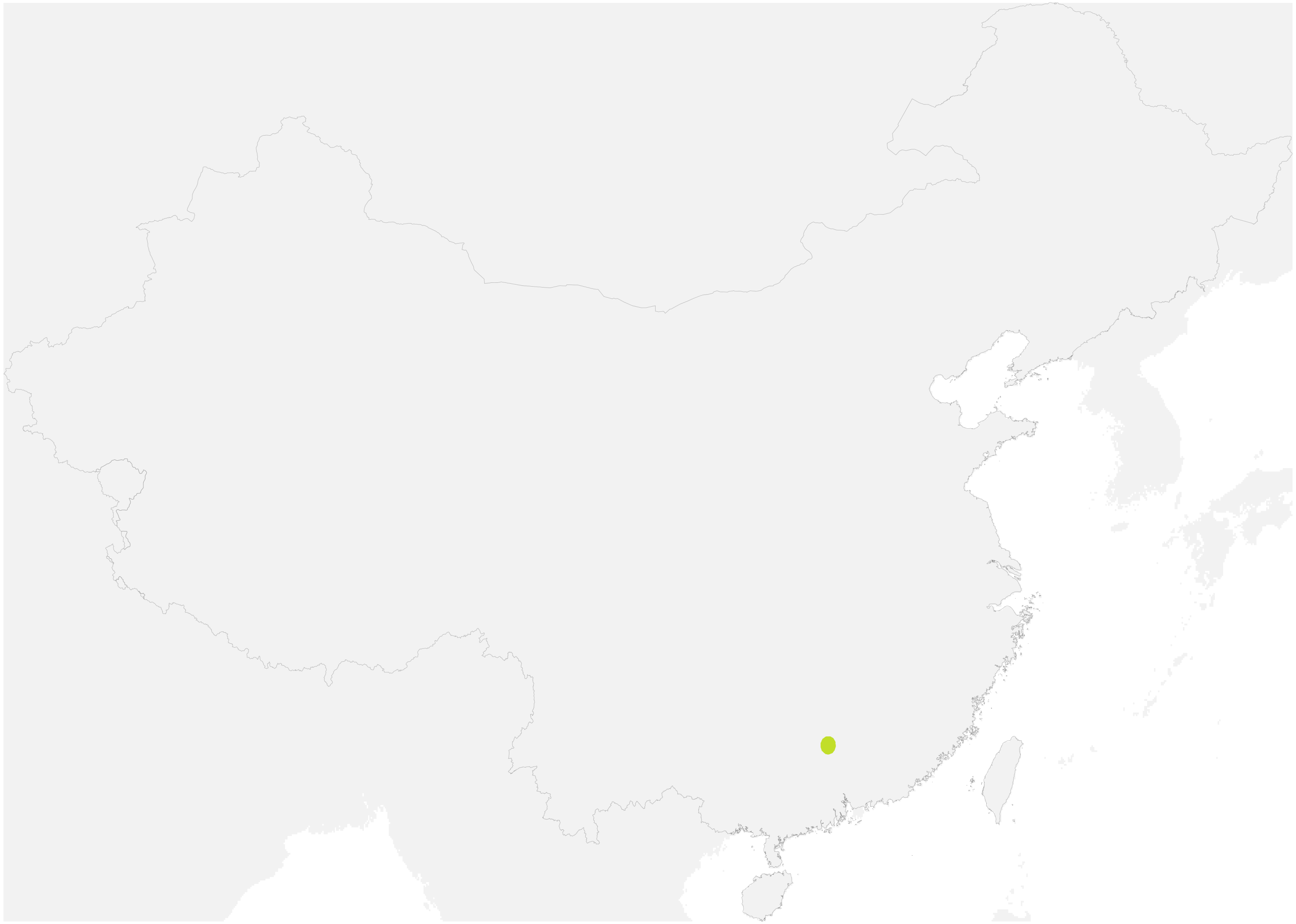

Current EOR

Protobothrops mucrosquamatus

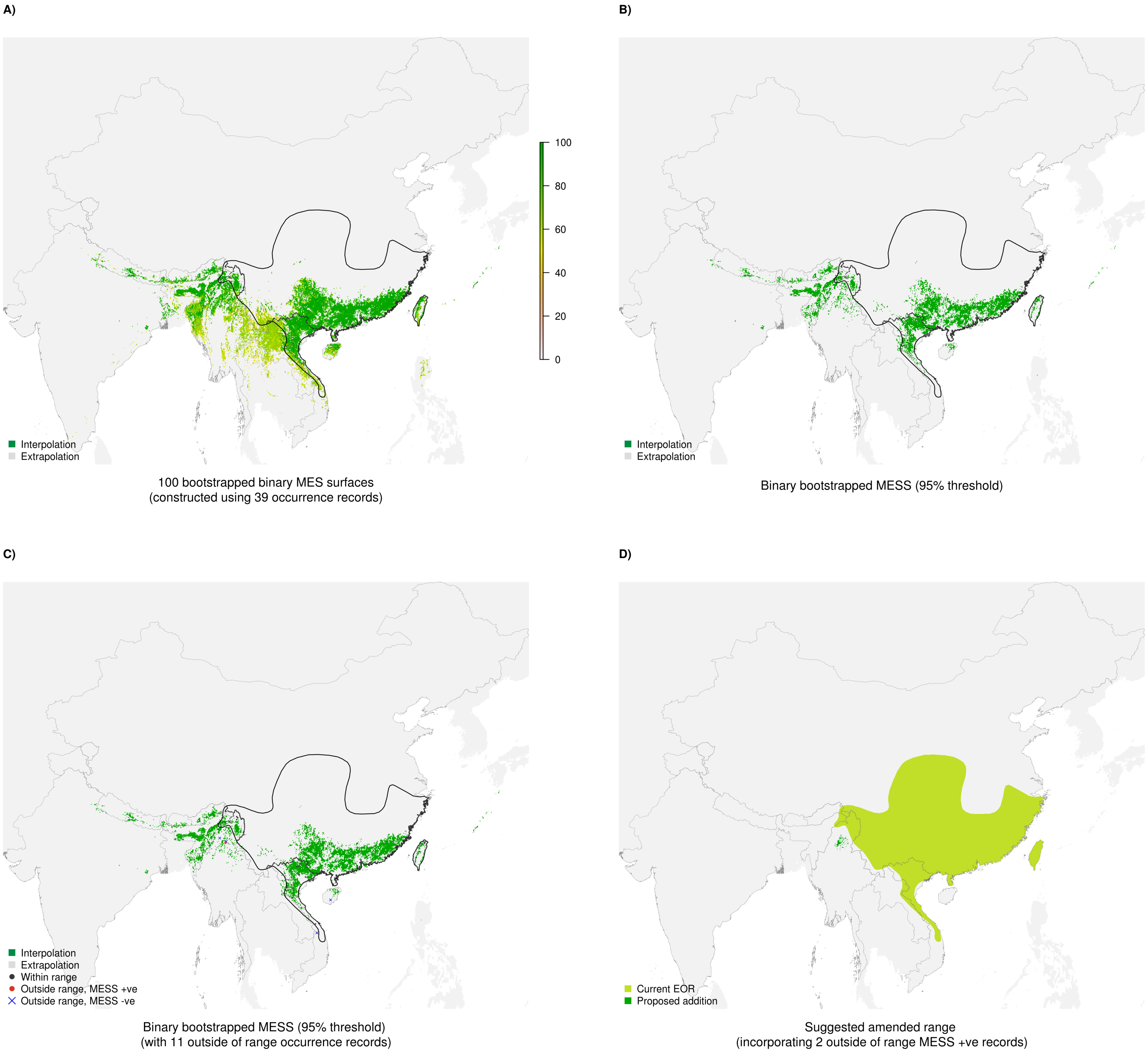

Pseudechis australis

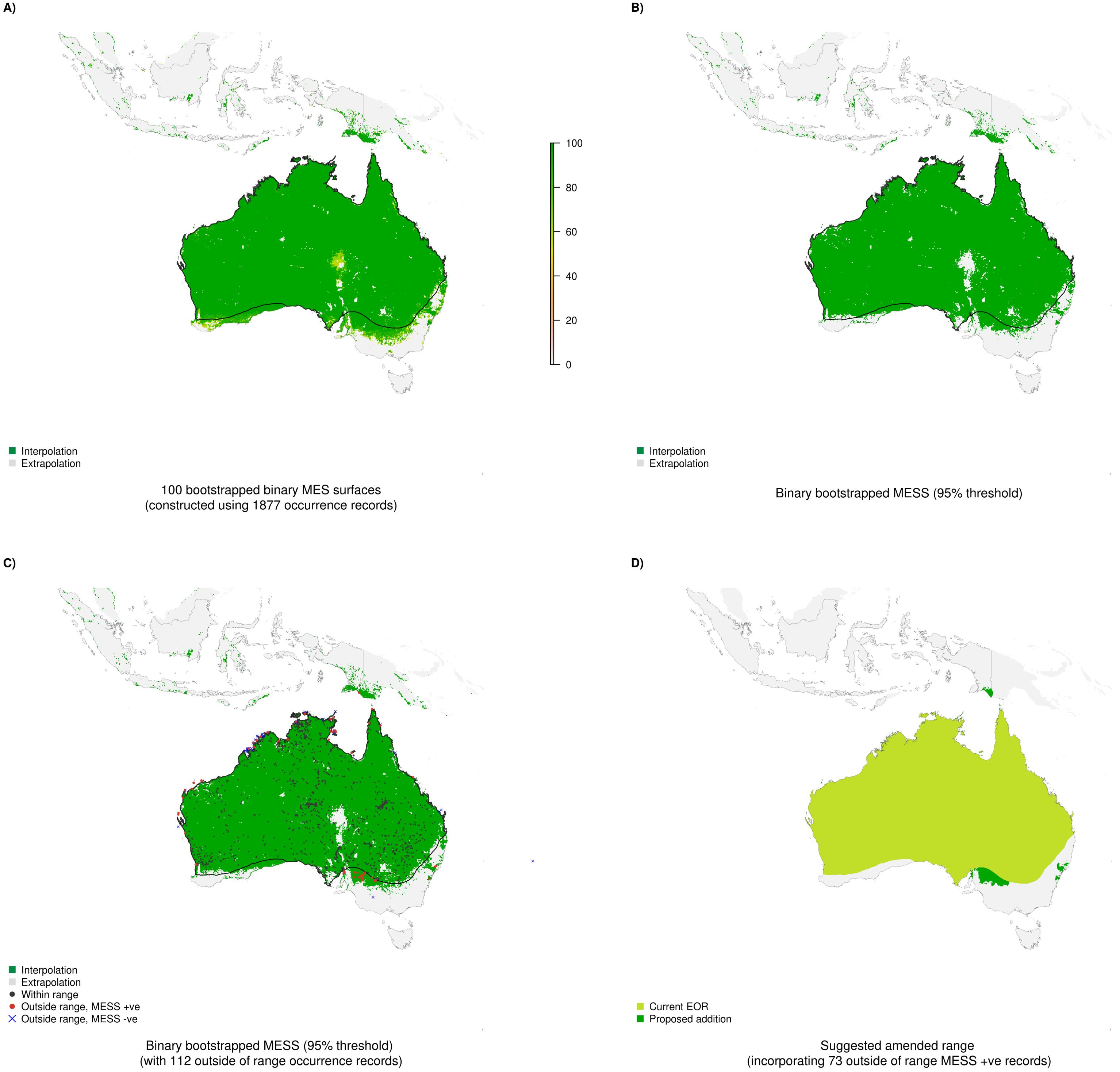

Pseudechis butleri

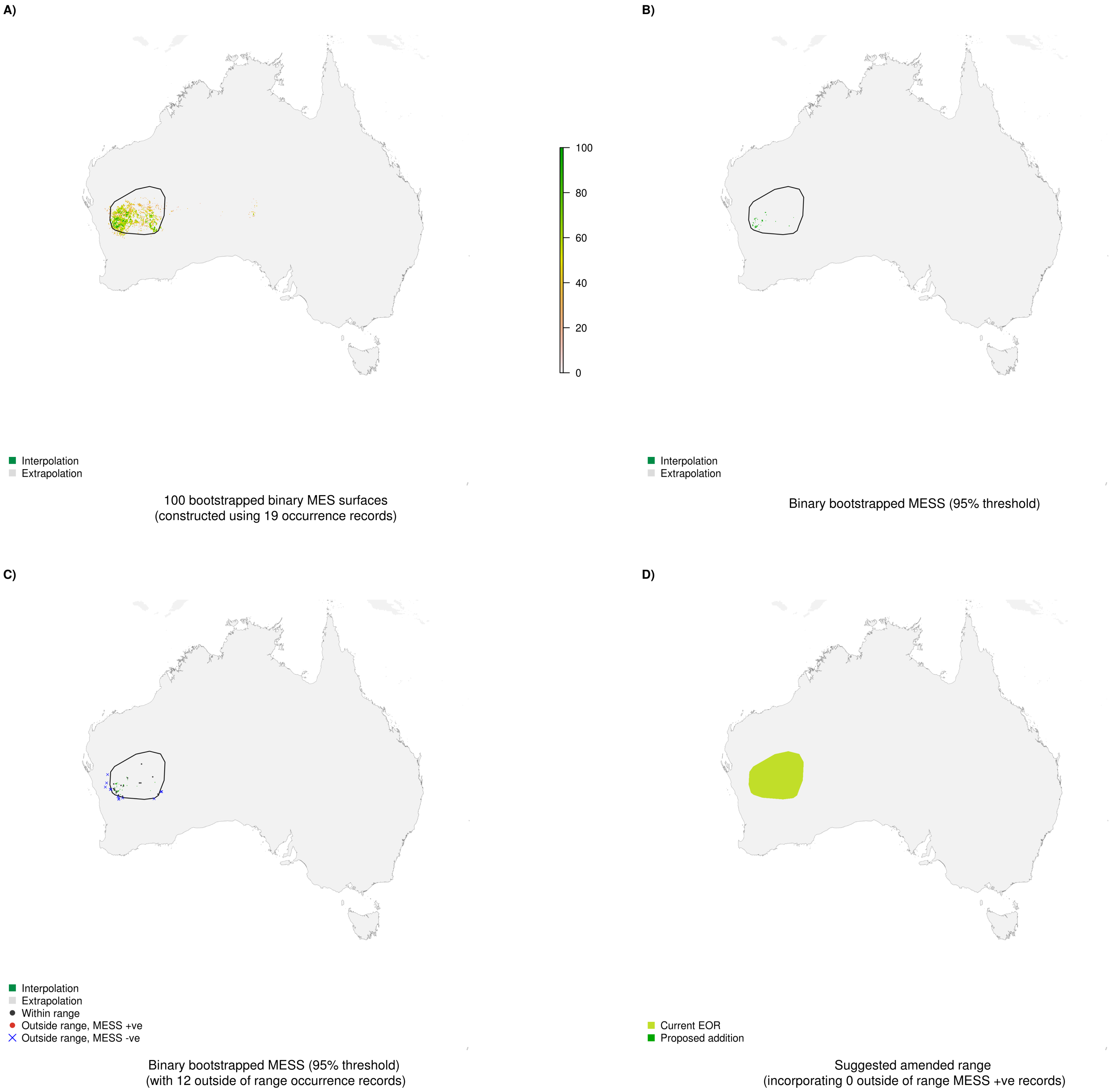

Pseudechis colletti

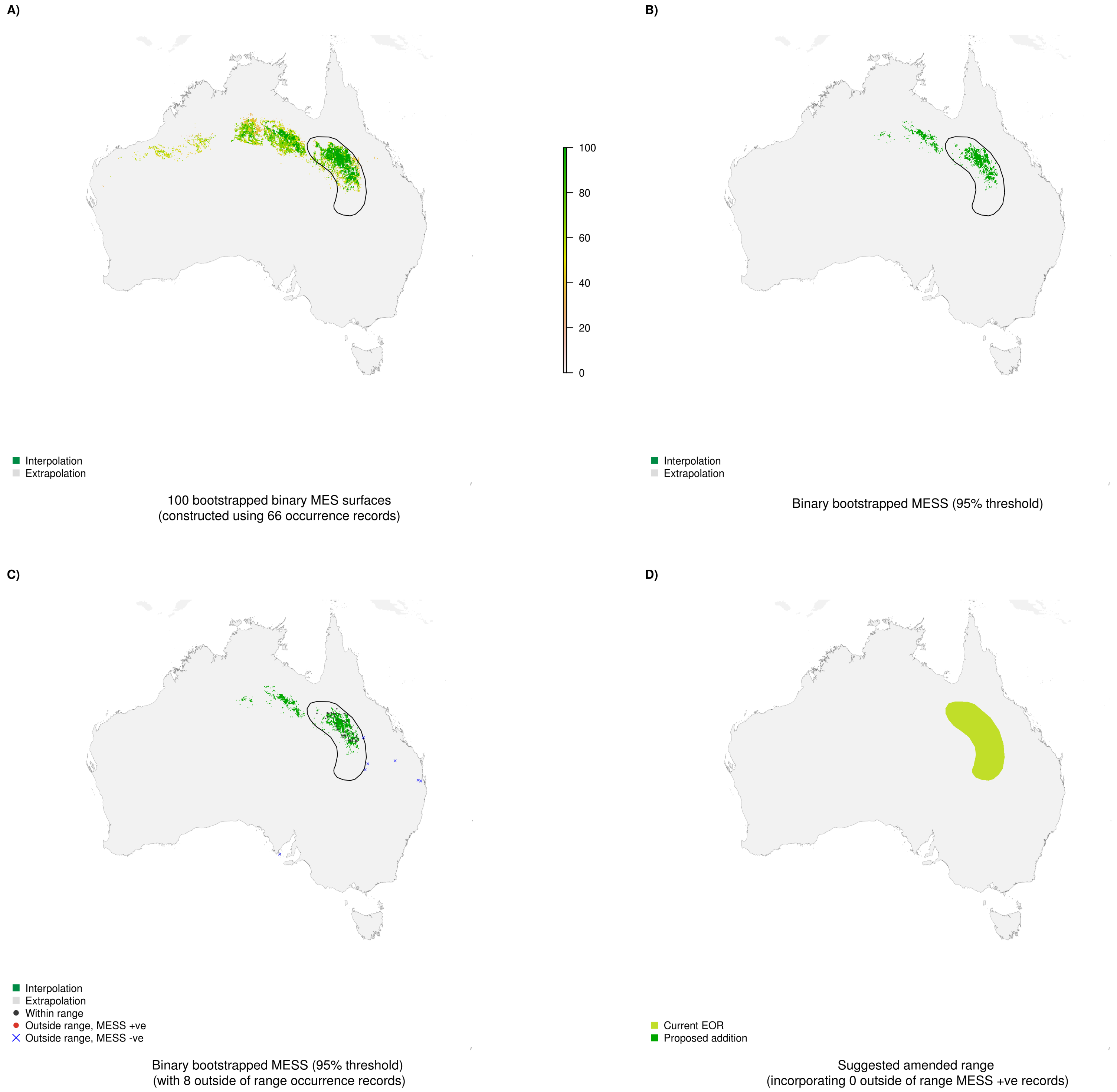

Pseudechis guttatus

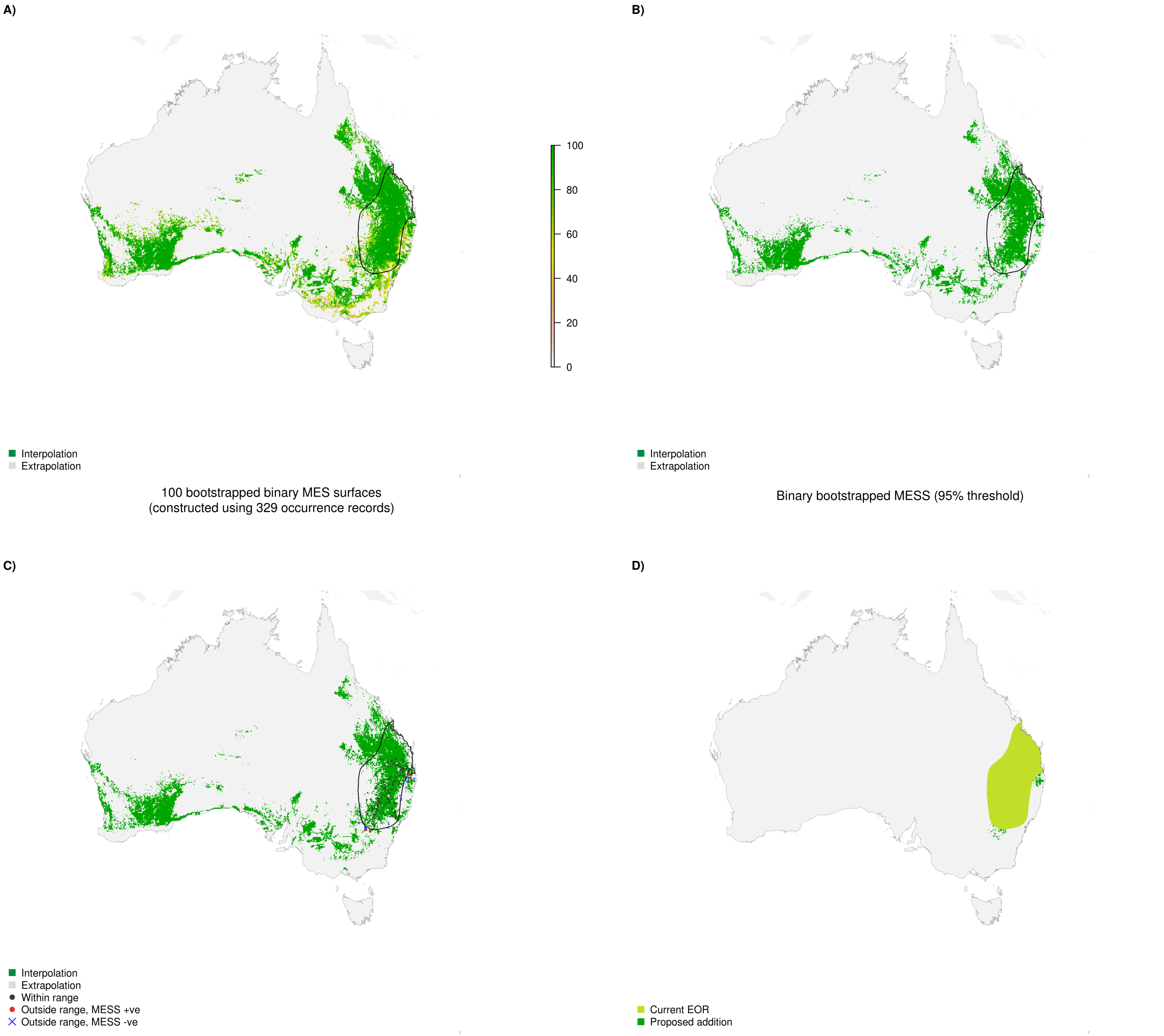

A) 100 bootstrapped binary MES surfaces (constructed using 329 occurrence records)

B) Binary bootstrapped MESS (95% threshold)

C) Binary bootstrapped MESS (95% threshold) (with 20 outside of range occurrence records)

D) Suggested amended range (incorporating 6 outside of range MESS +ve records)

Pseudechis papuanus

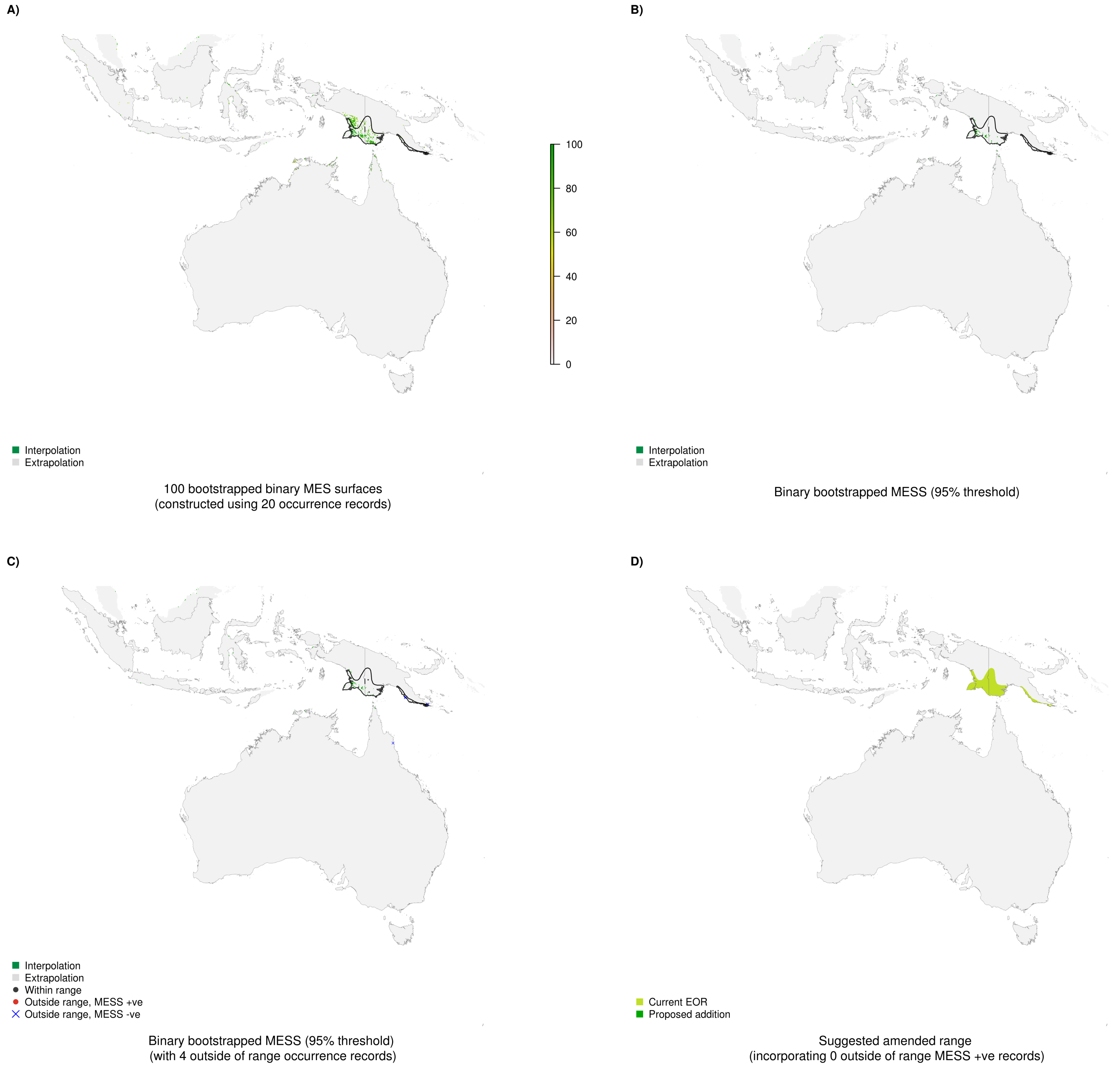

Pseudechis porphyriacus

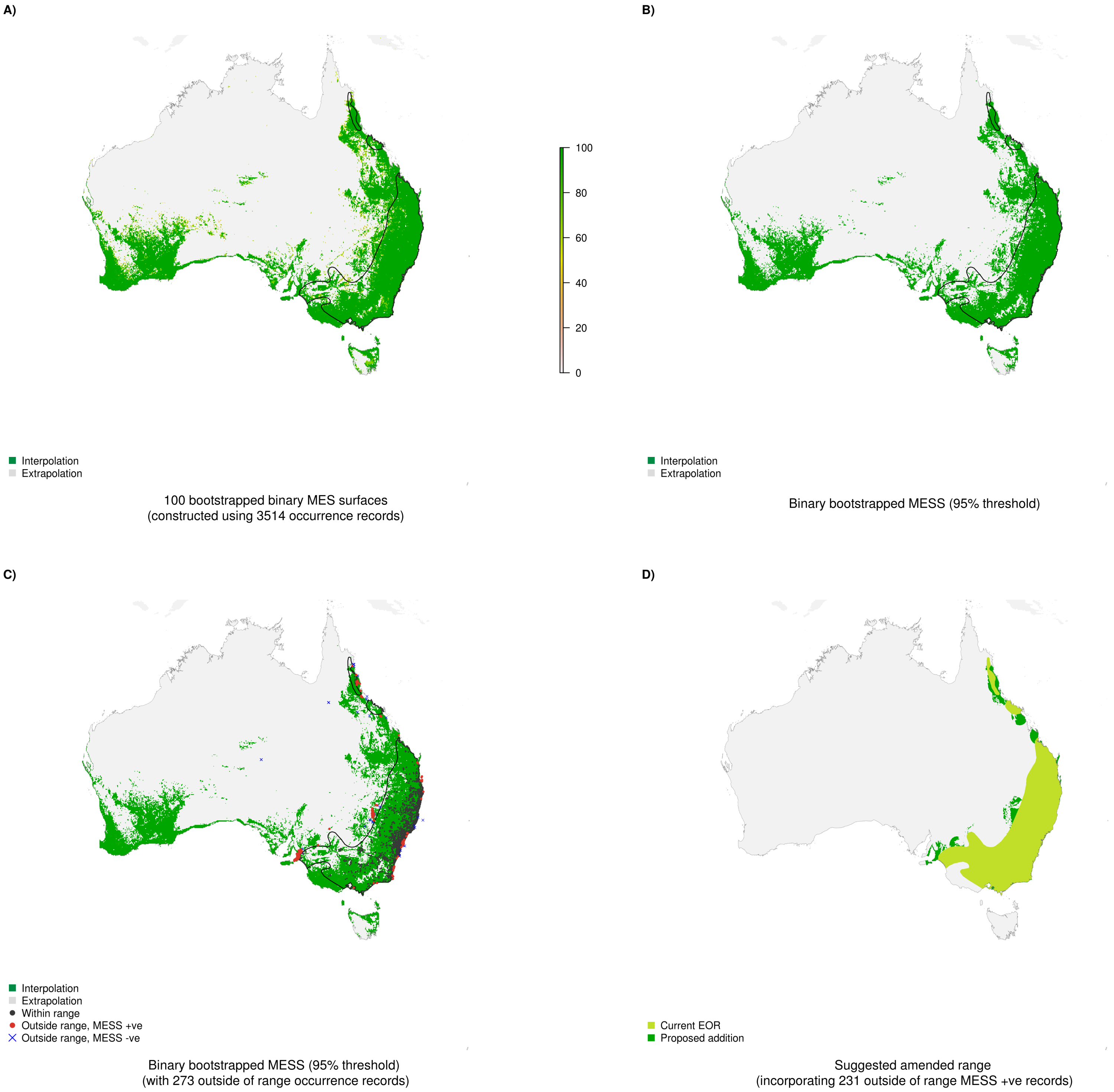

Pseudechis rossignolii

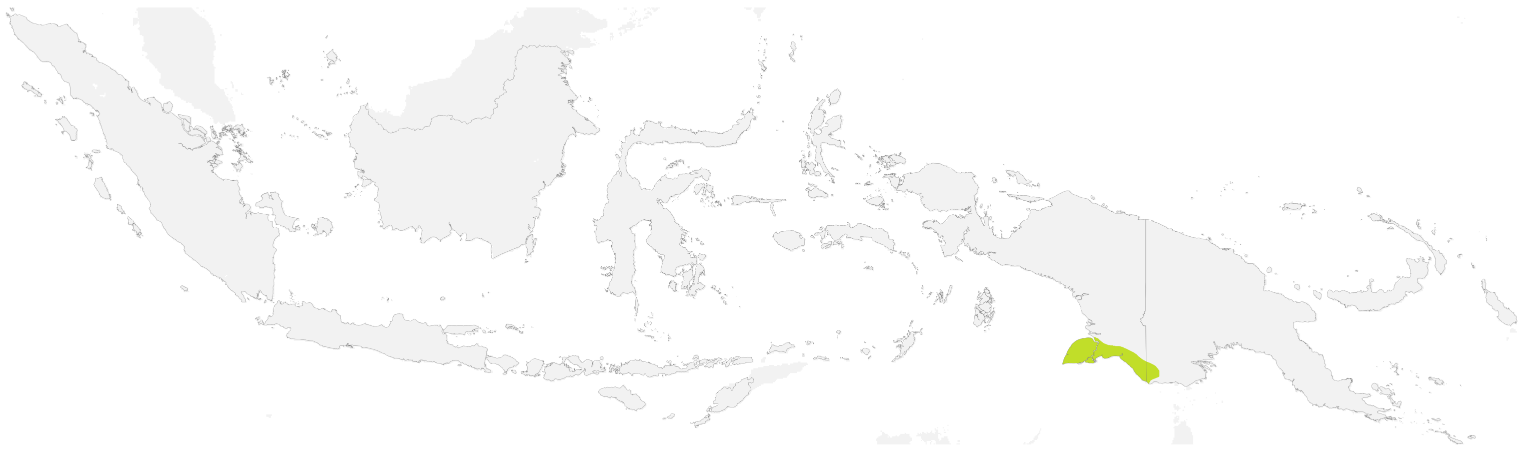

Pseudechis weigeli

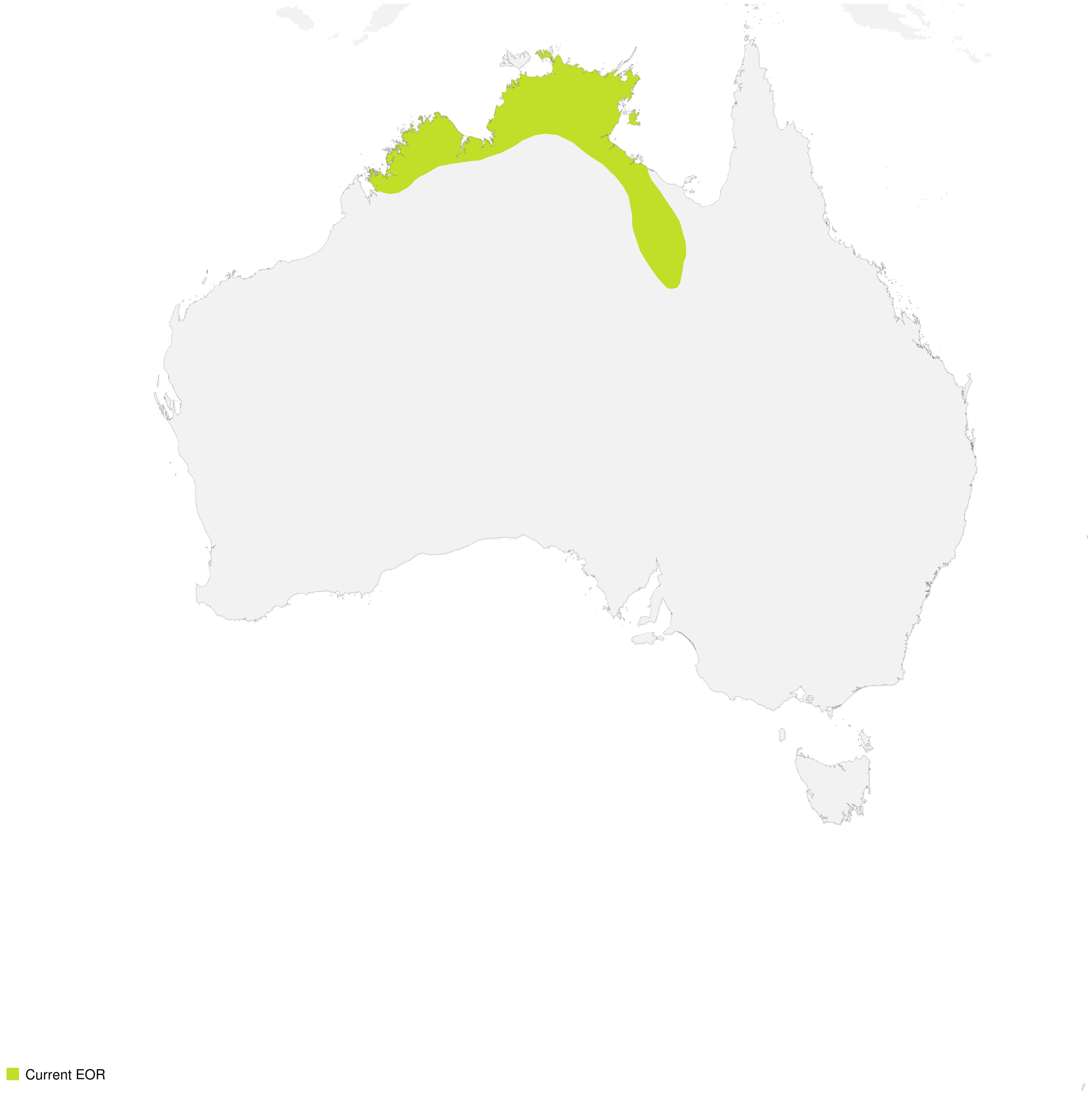

Pseudocerastes fieldi

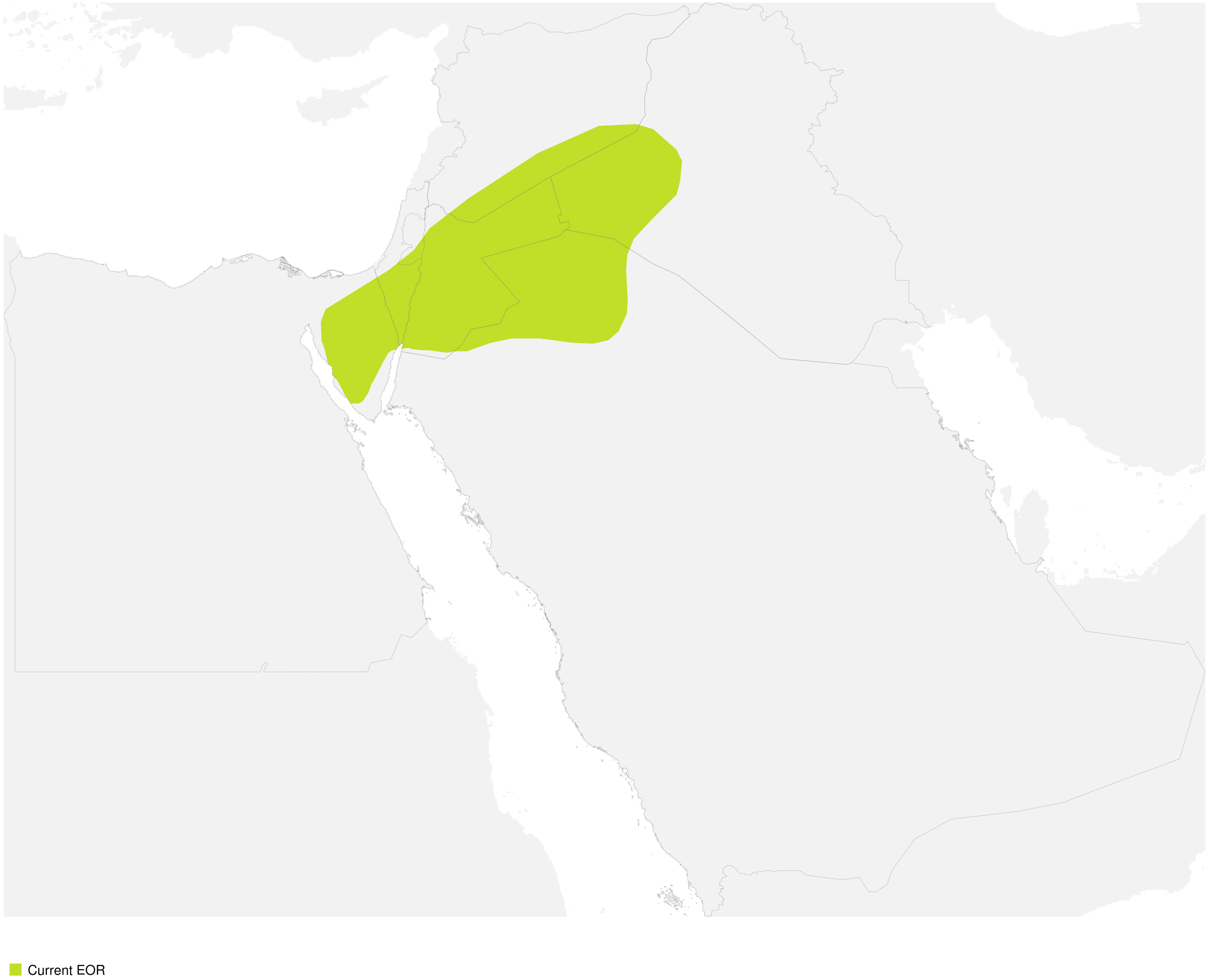

Pseudocerastes persicus

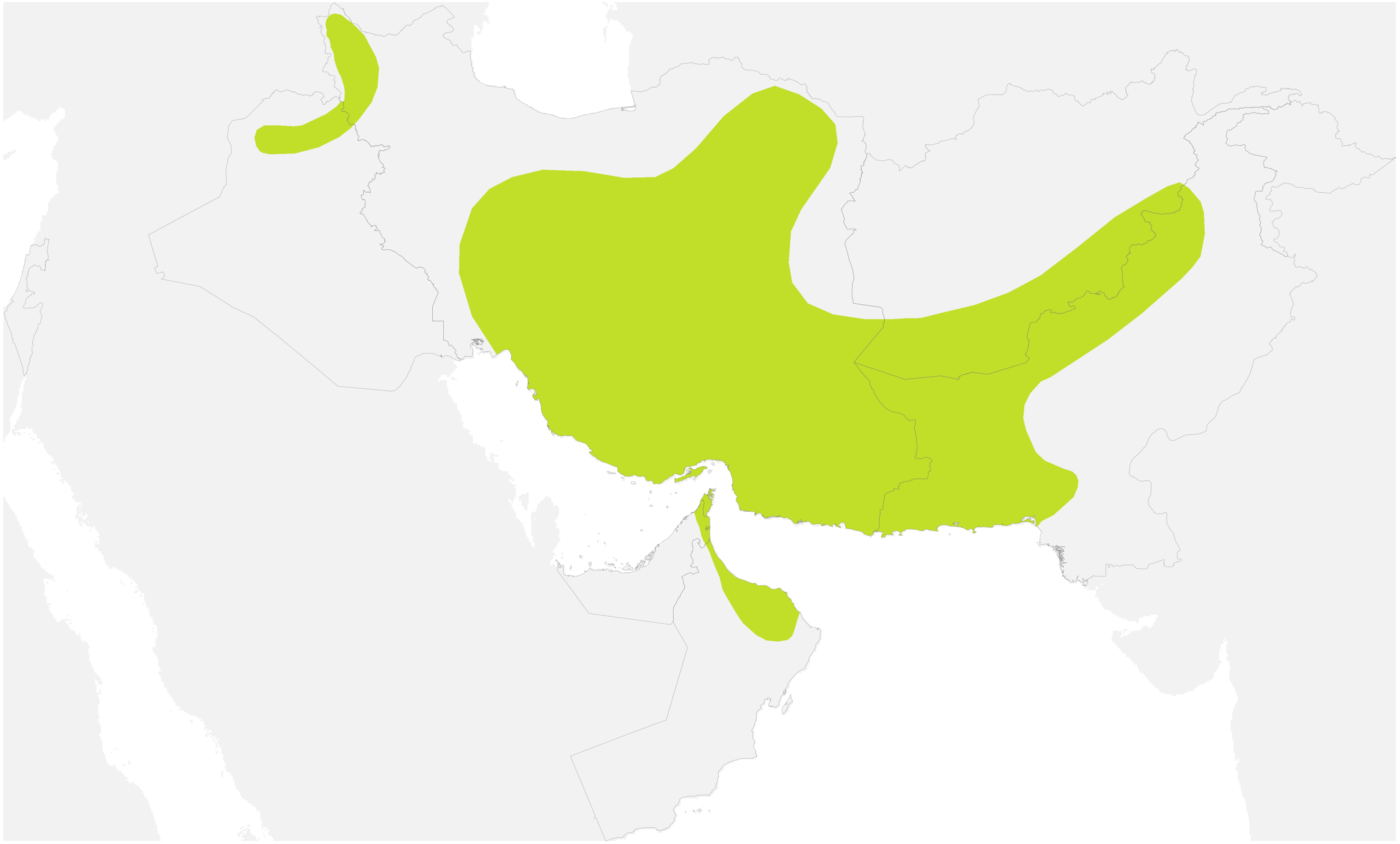

Current EOR

Pseudohaje goldii

A)

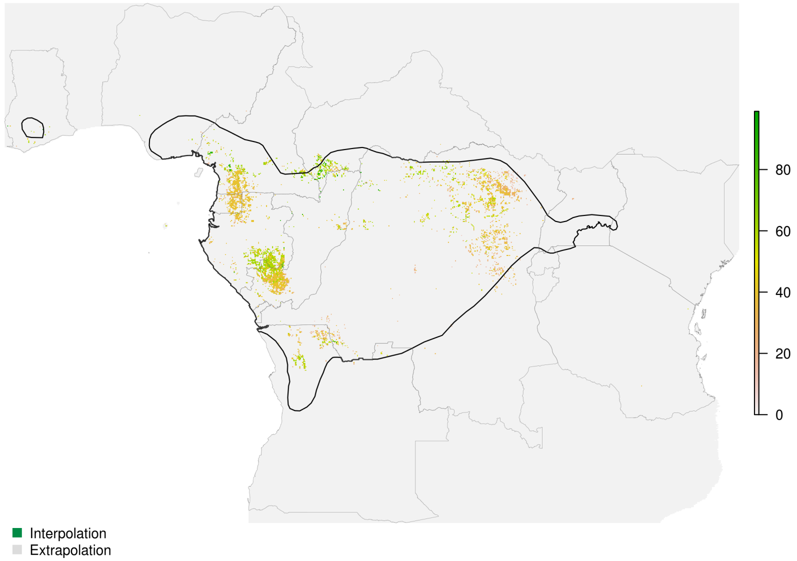

100 bootstrapped binary MES surfaces
(constructed using 11 occurrence records)

B)

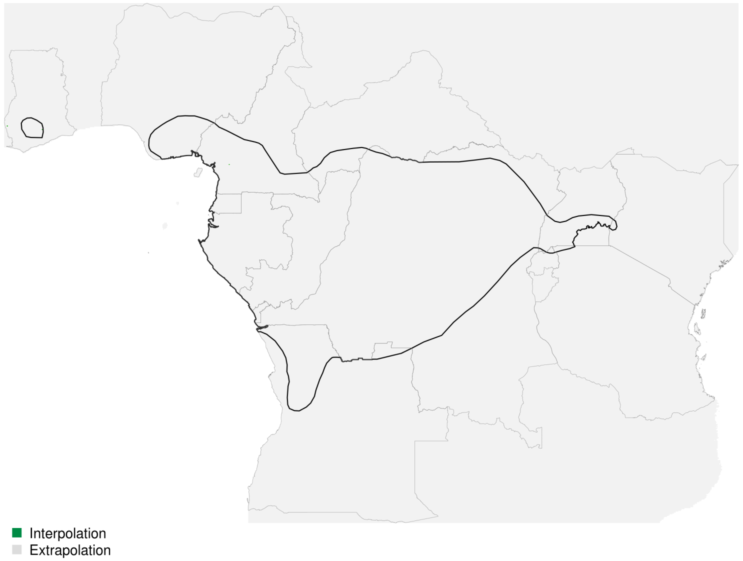

Binary bootstrapped MESS (95% threshold)

C)

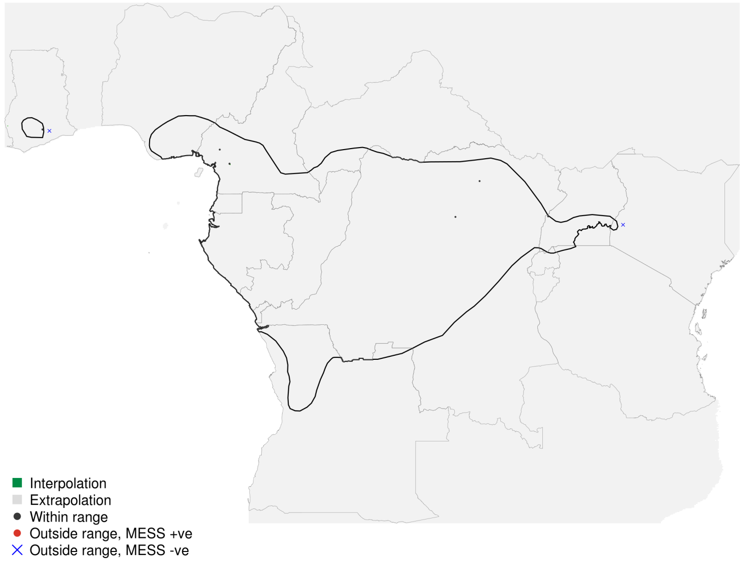

Binary bootstrapped MESS (95% threshold)
(with 2 outside of range occurrence records)

D)

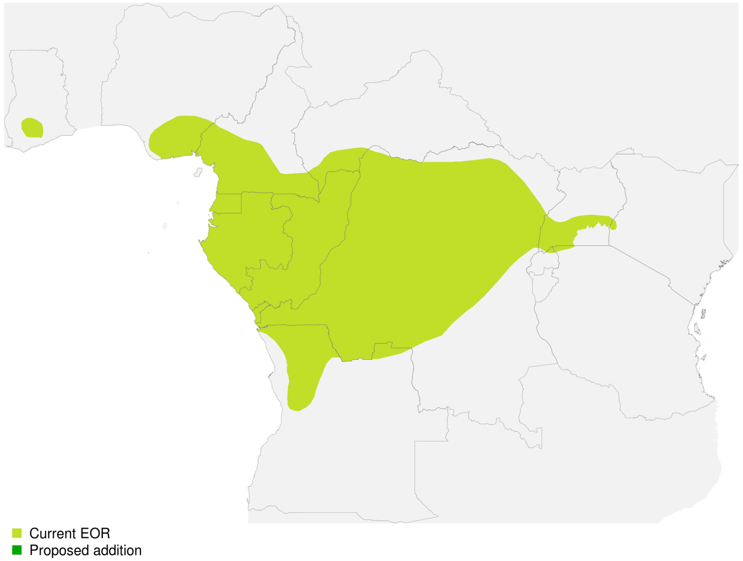

Suggested amended range
(incorporating 0 outside of range MESS +ve records)

Pseudohaje nigra

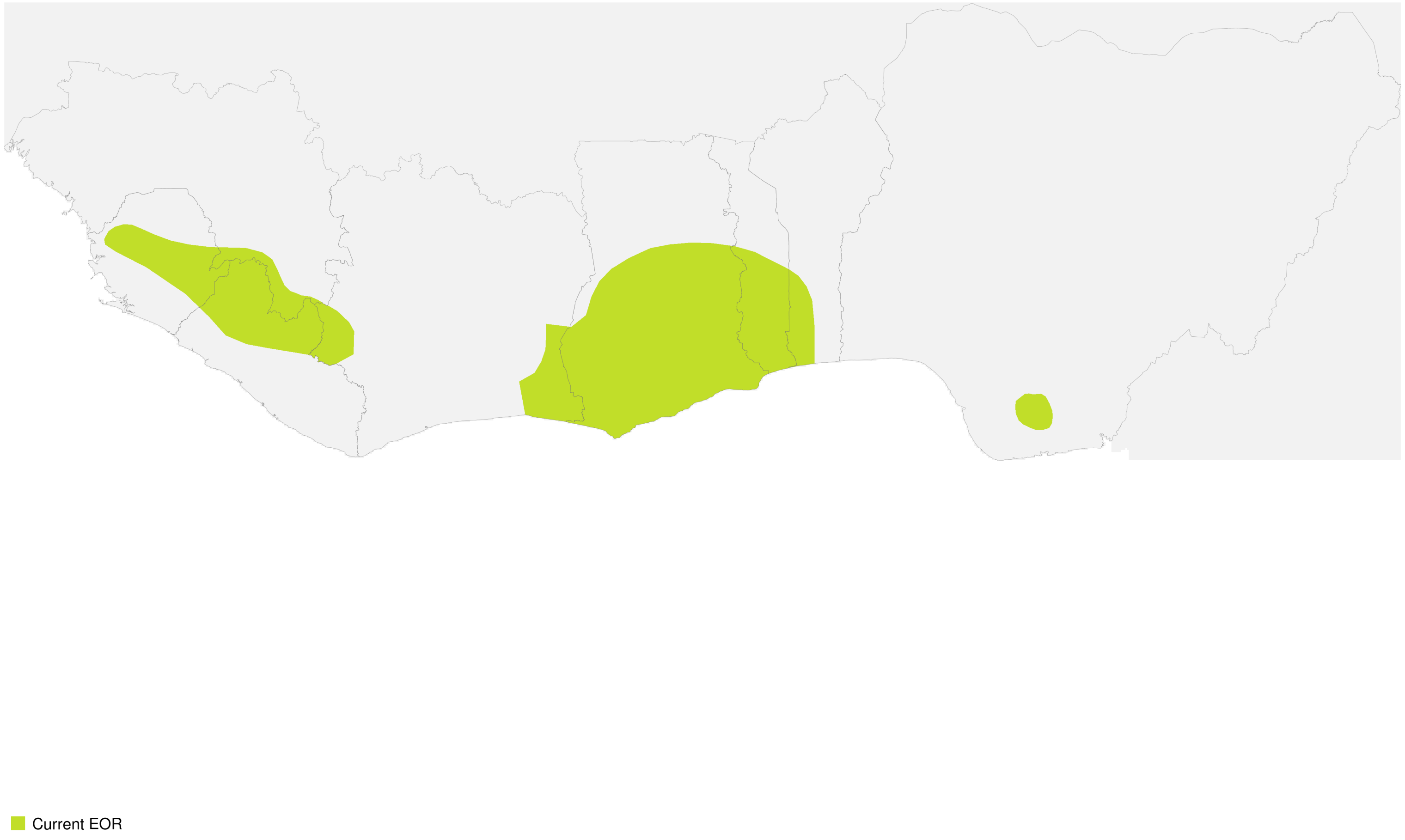

Pseudonaja affinis

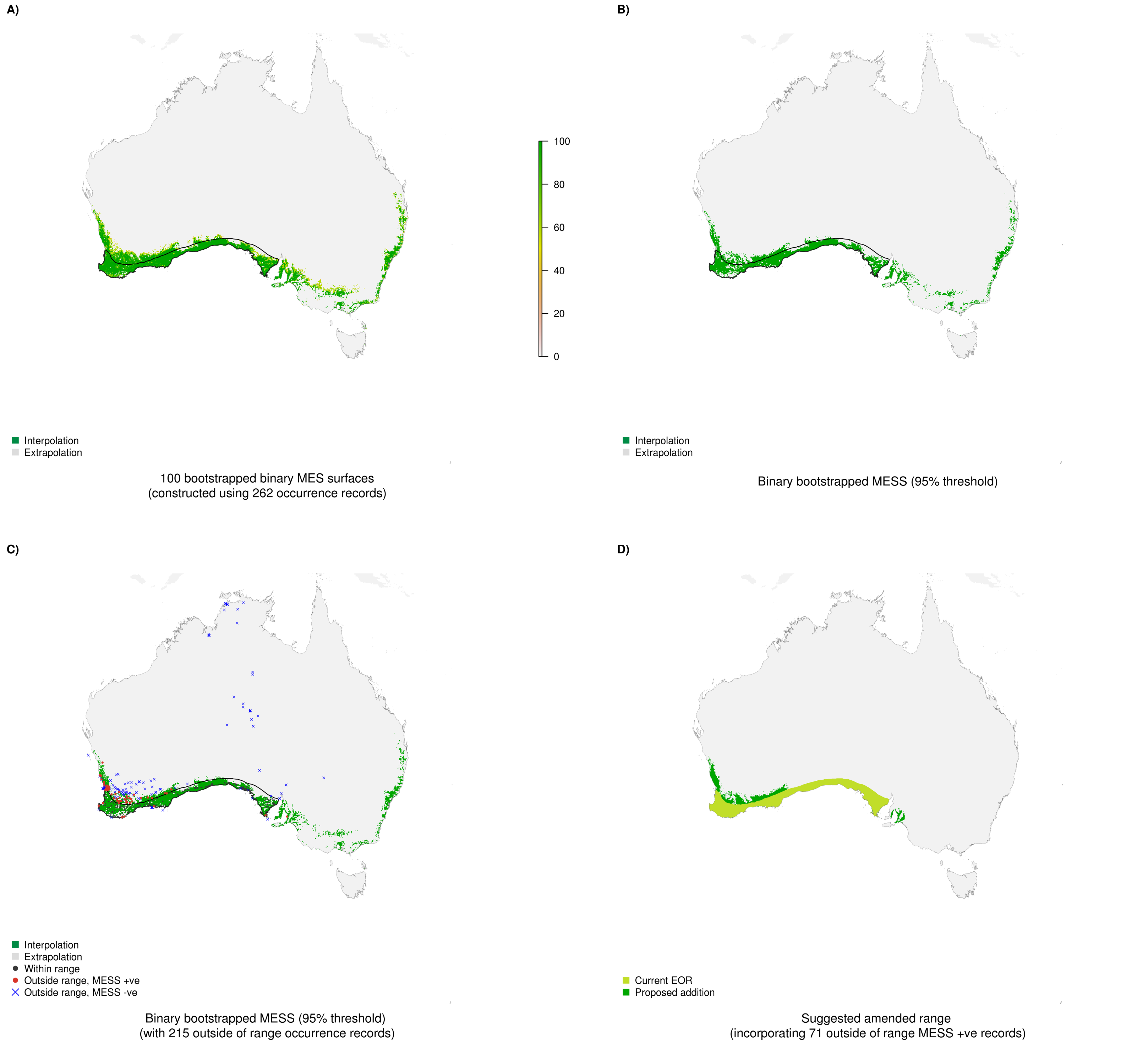

Pseudonaja aspidorhyncha

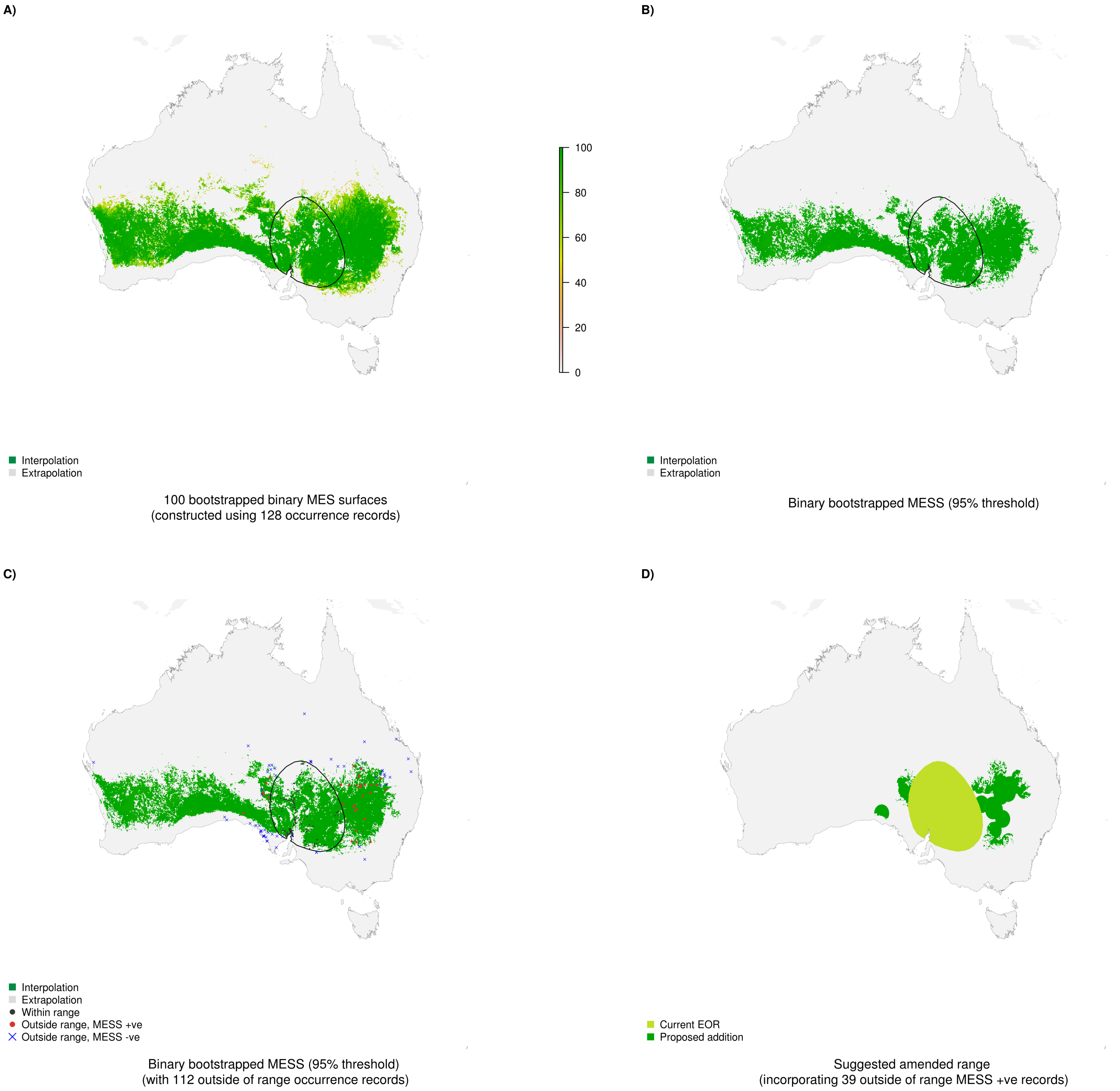

Pseudonaja guttata

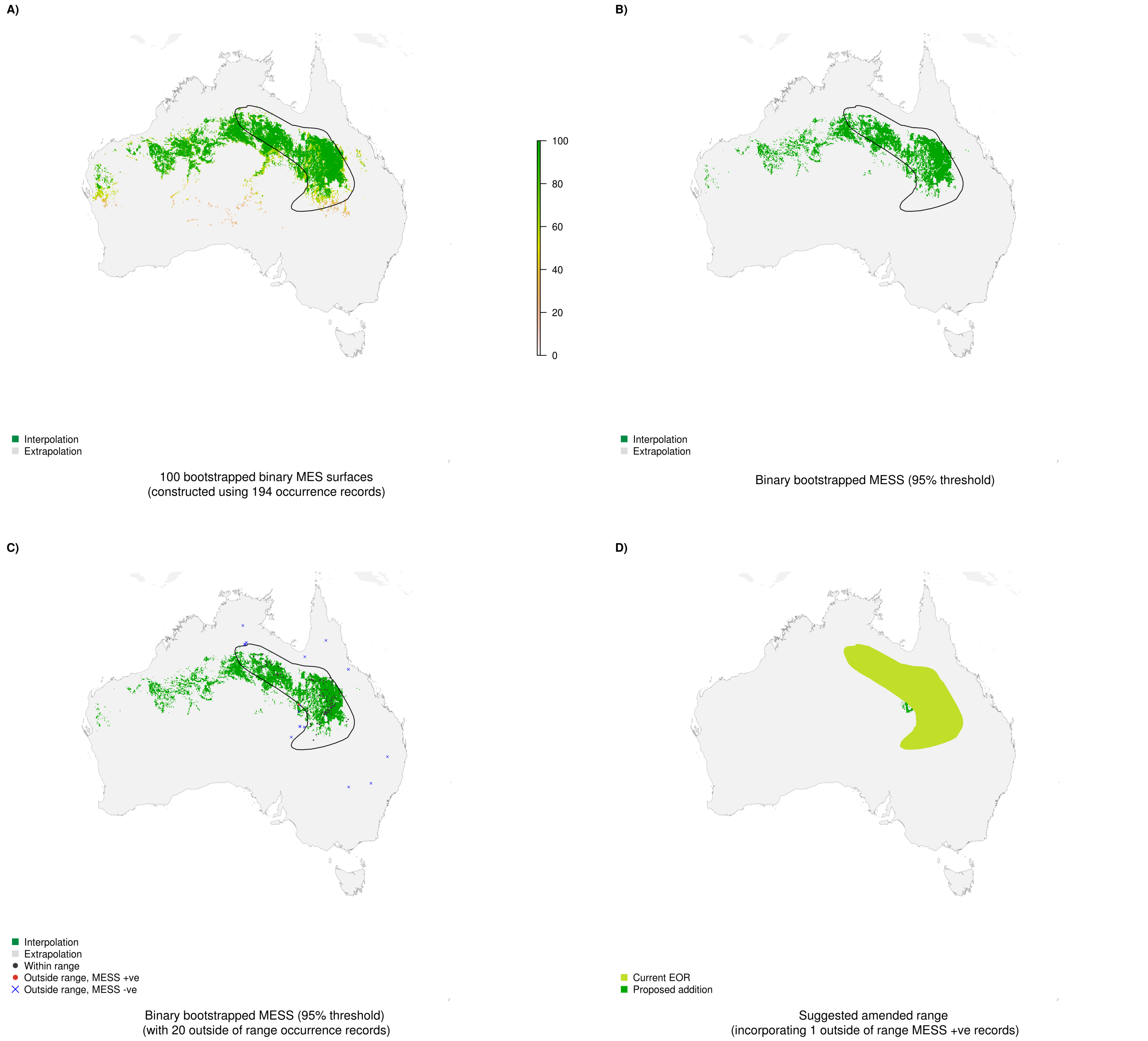

Pseudonaja inframacula

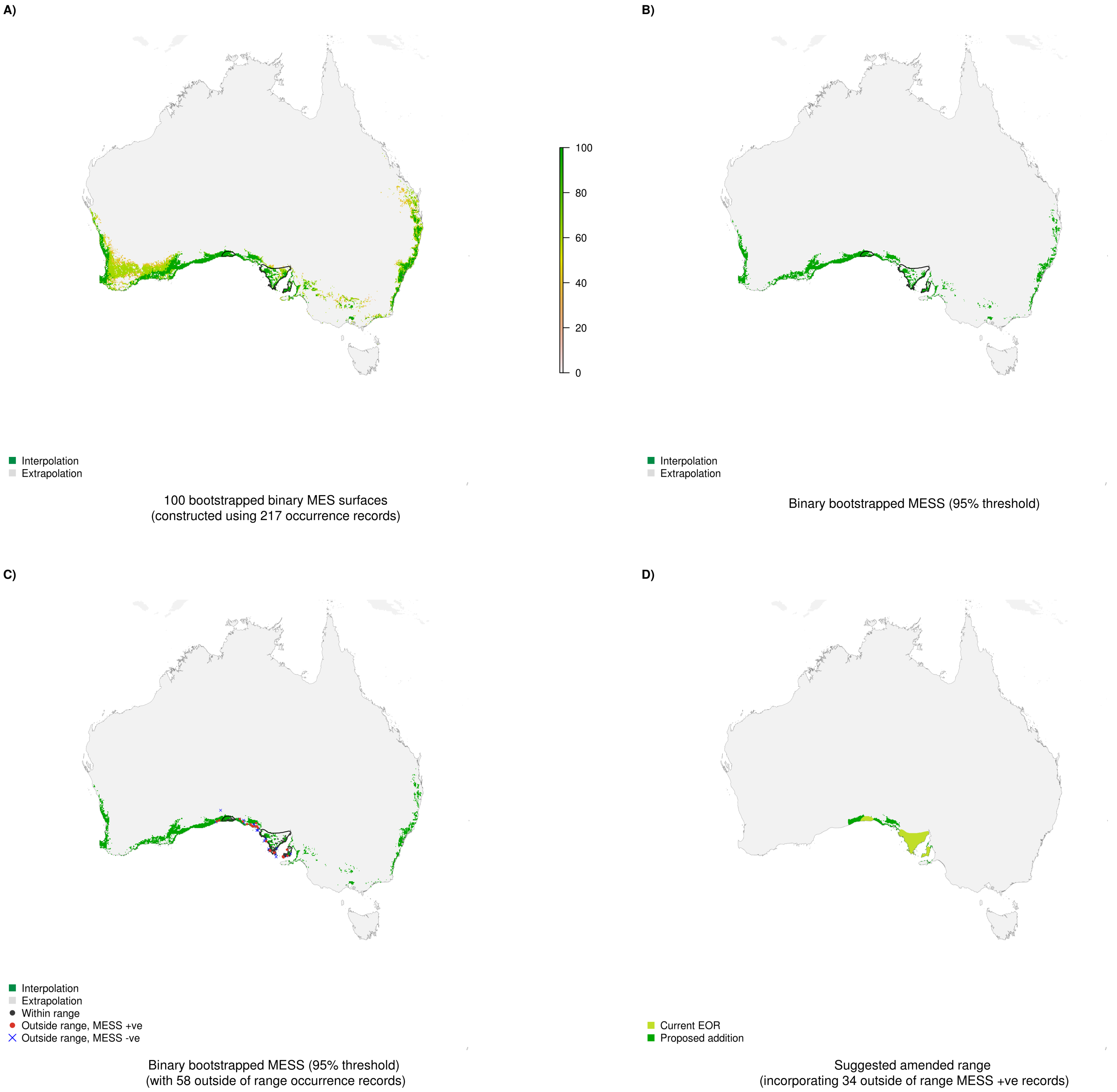

Pseudonaja ingrami

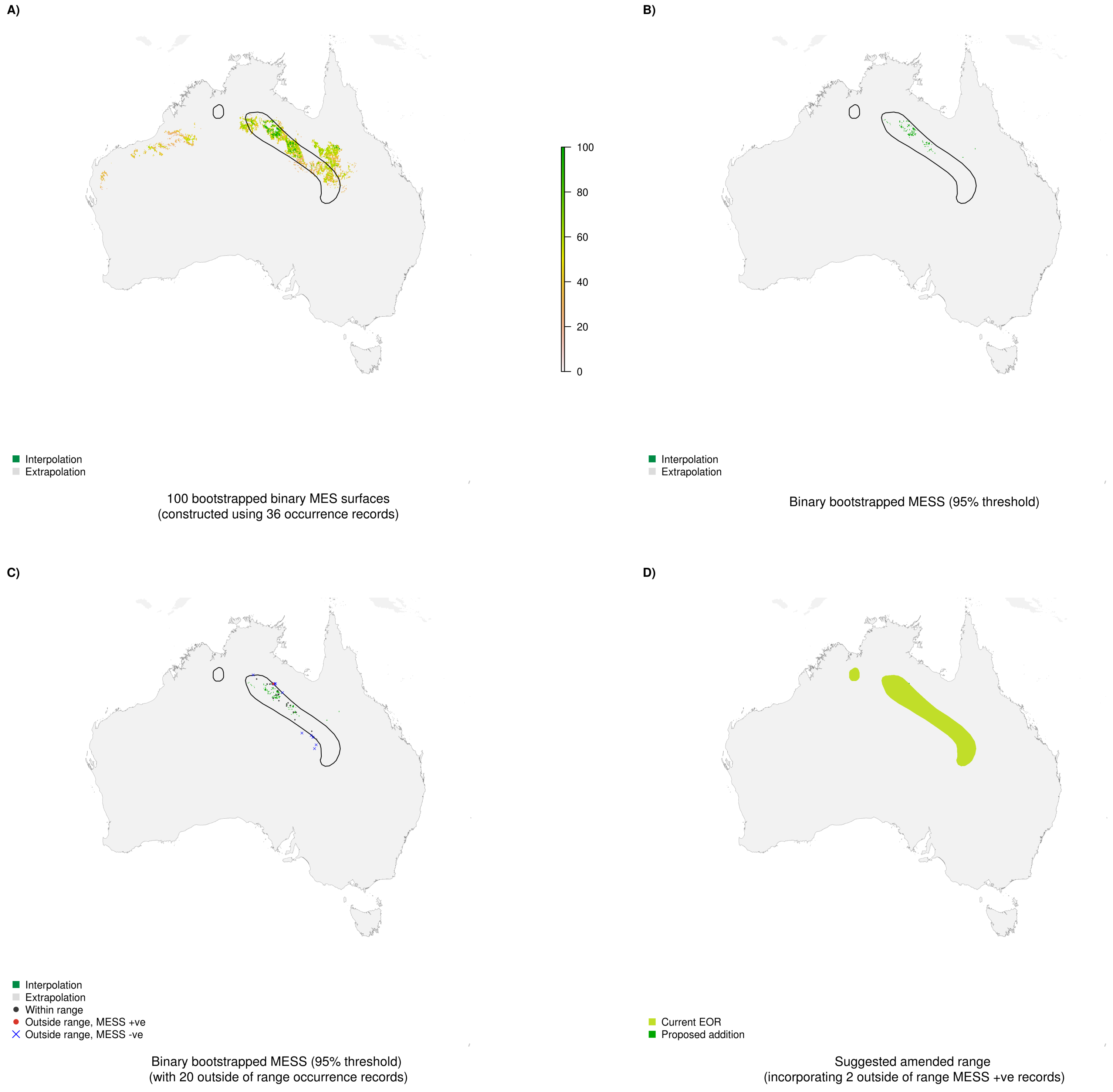

Pseudonaja mengdeni

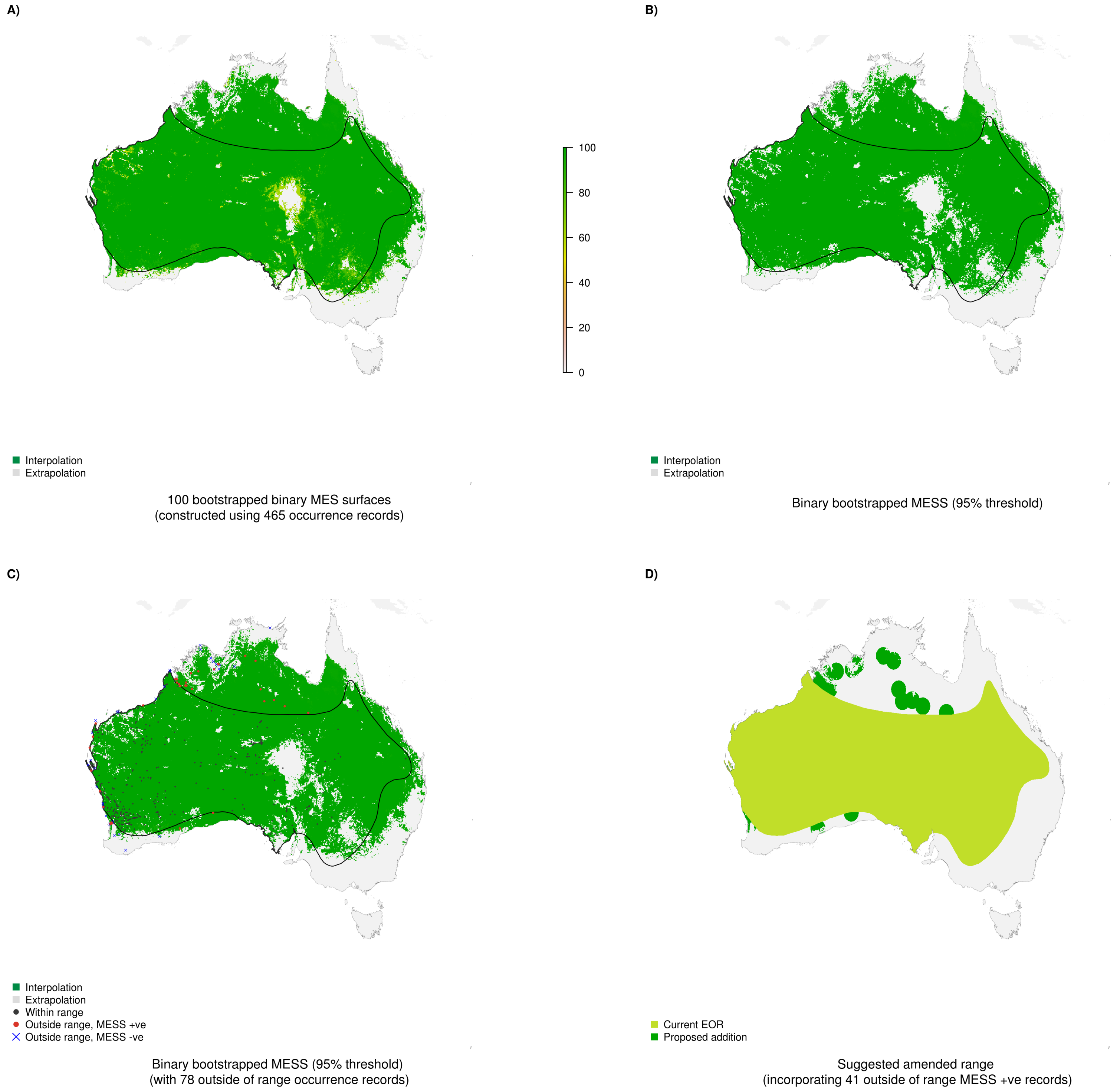

Pseudonaja nuchalis

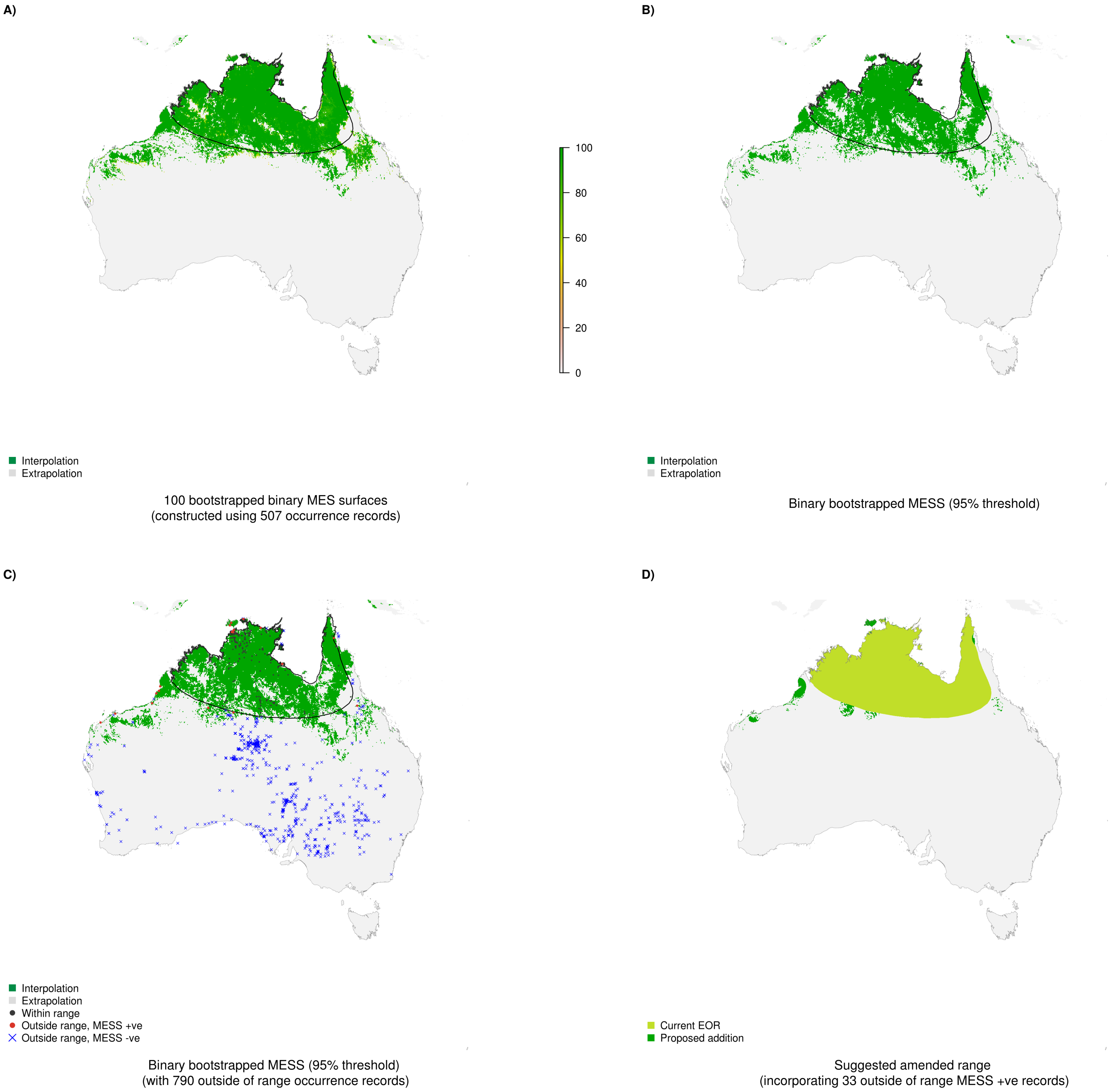

Pseudonaja tanneri

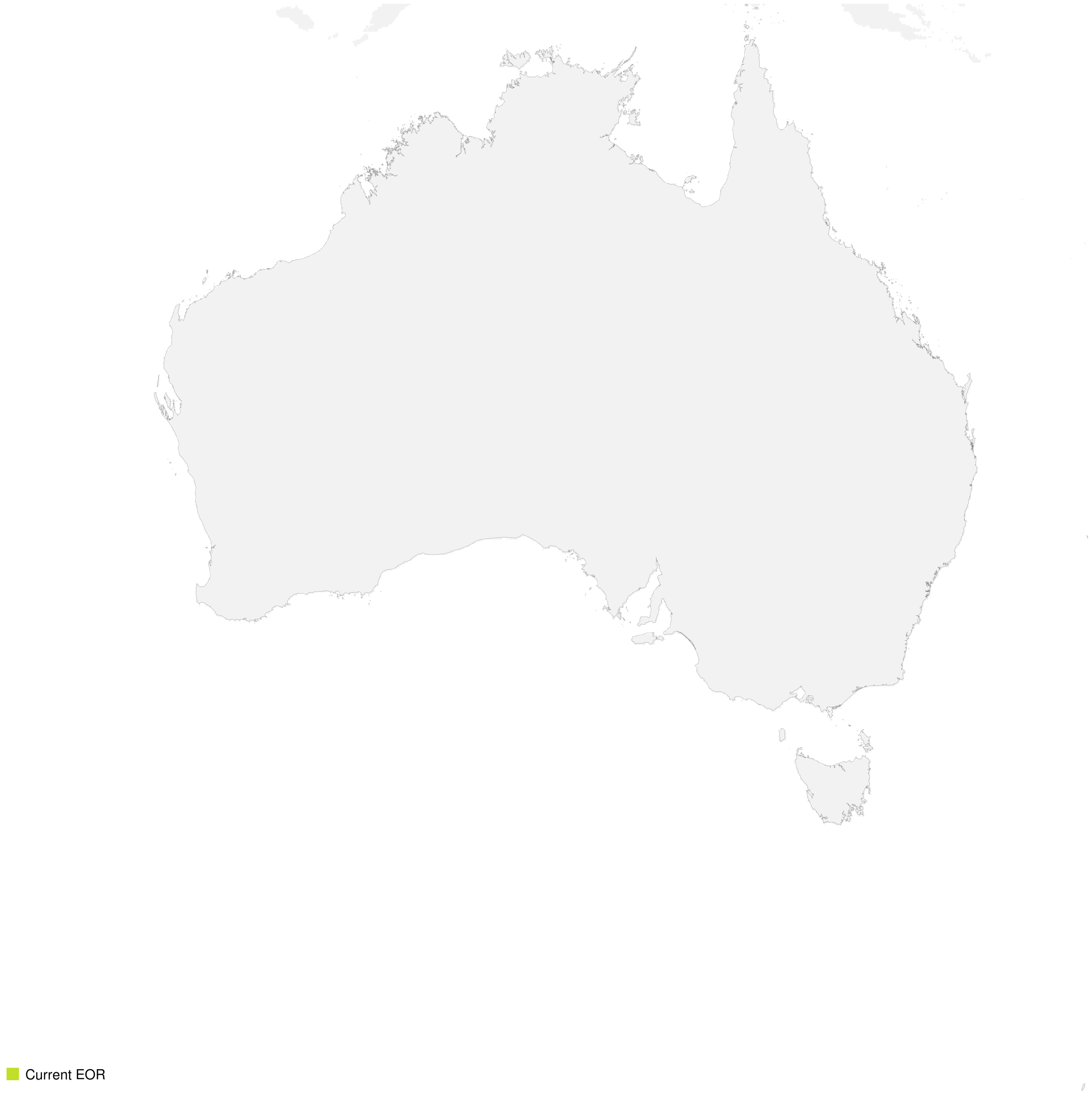

Pseudonaja textilis

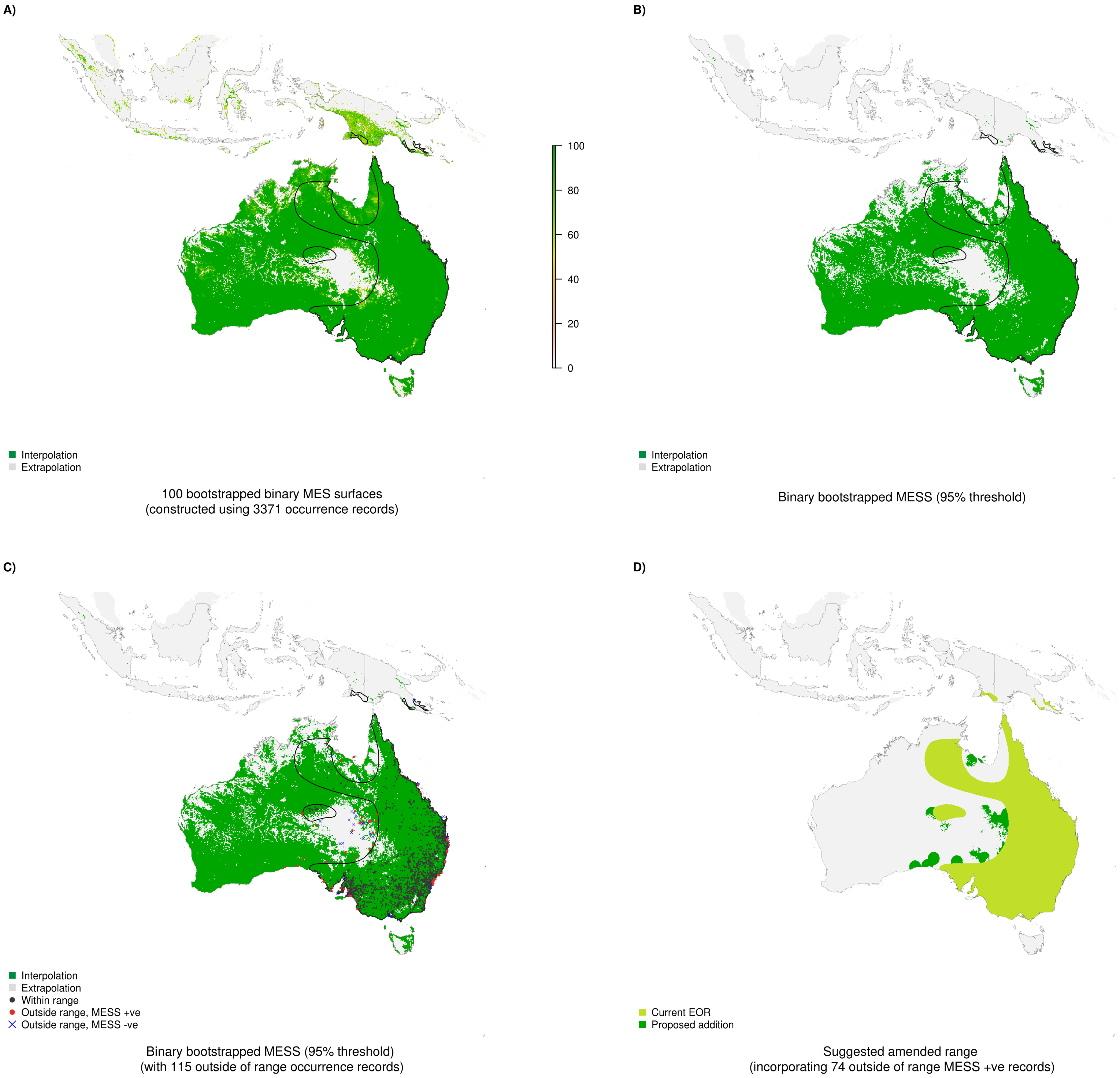

Rhabdophis tigrinus

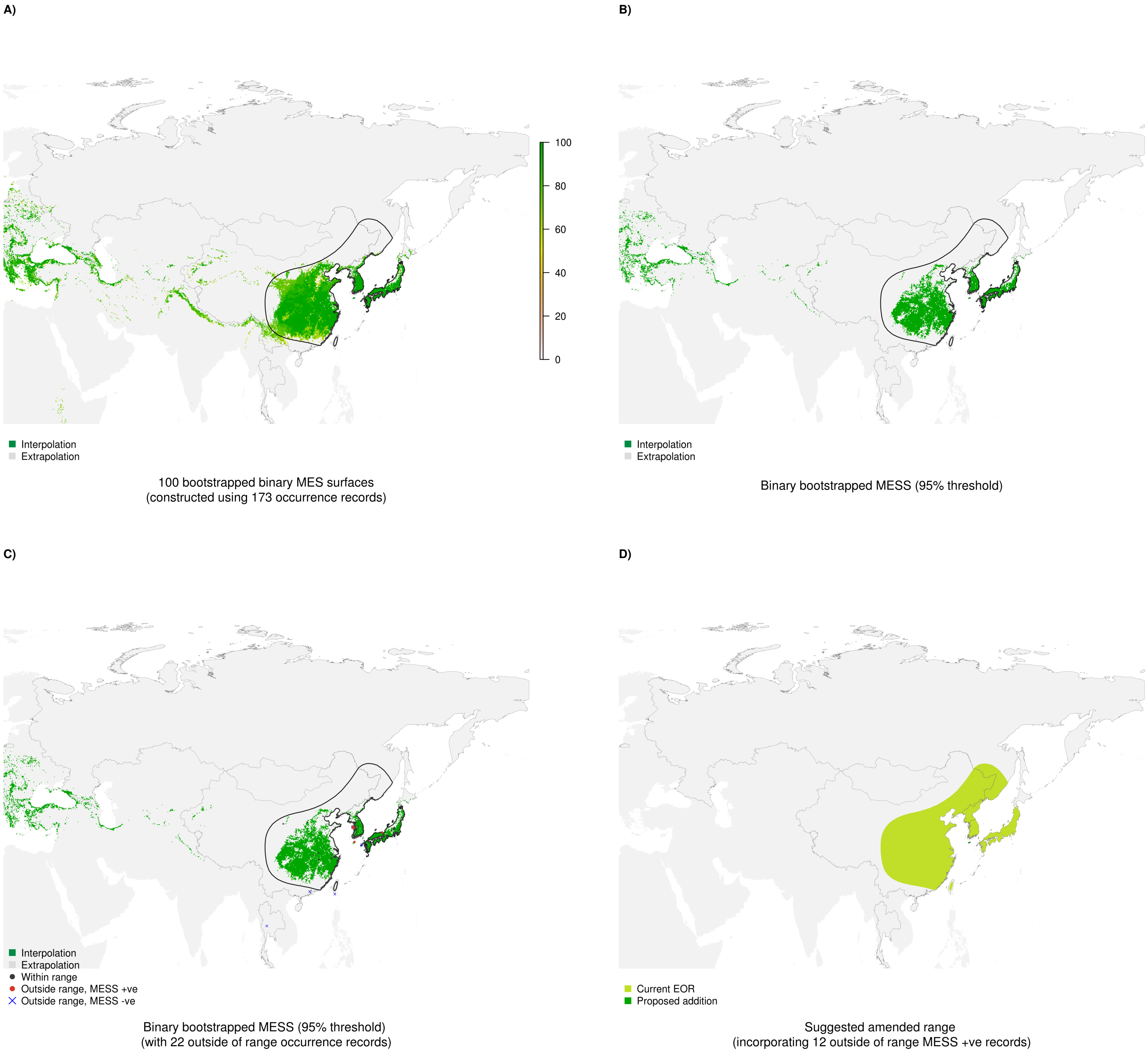

Sistrurus catenatus

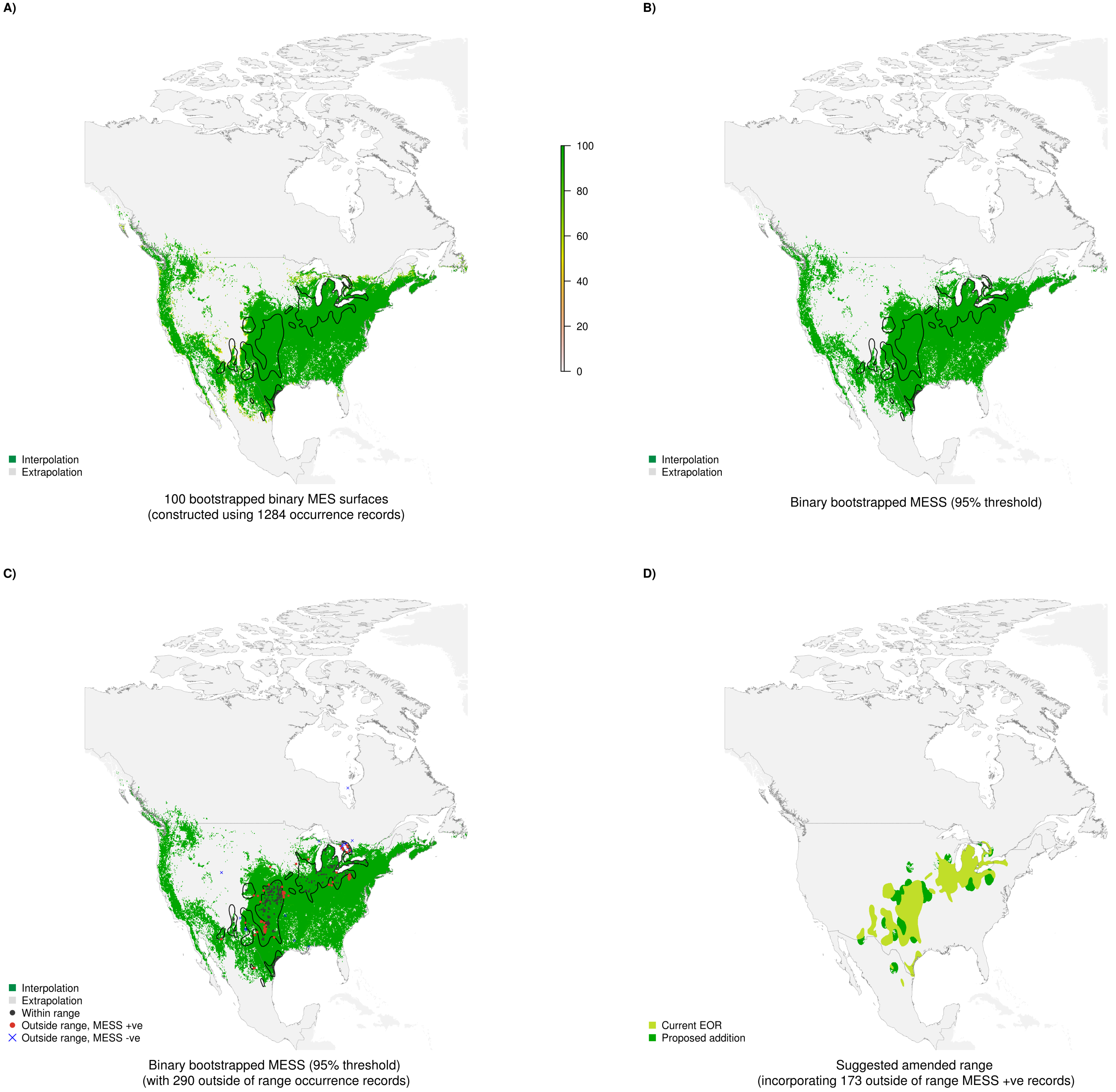

Sistrurus miliarius

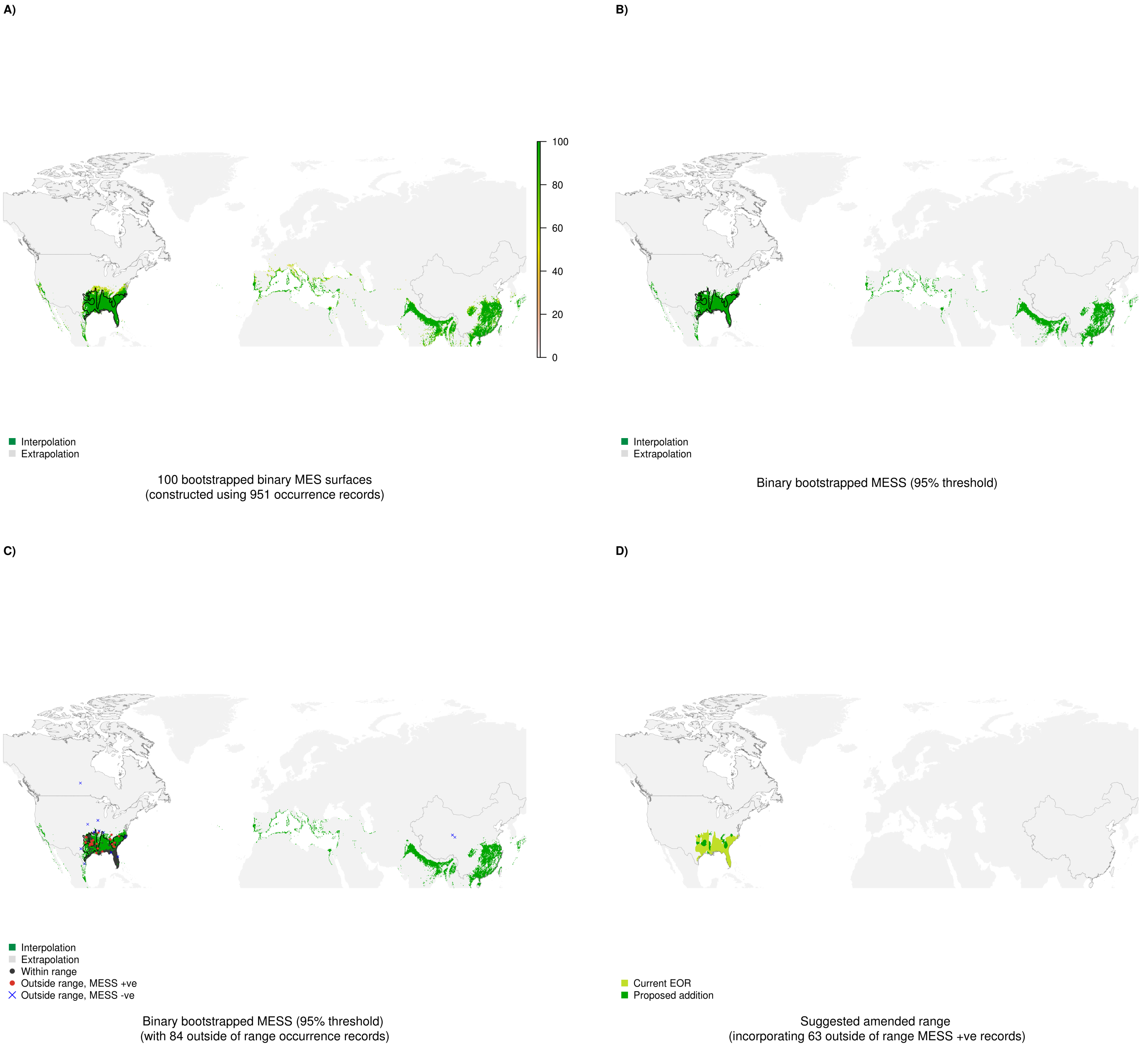

Thelotornis capensis

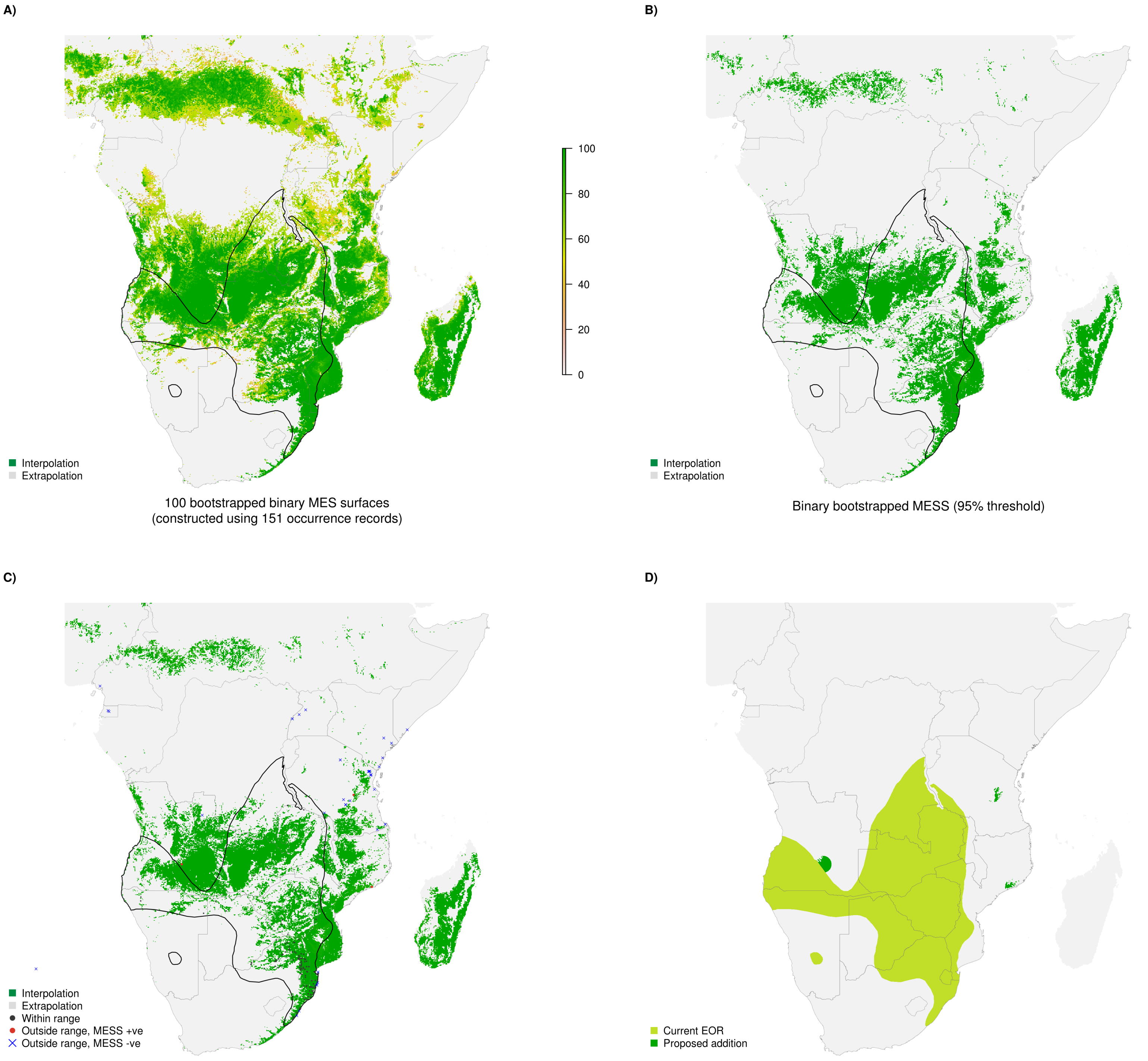

A) 100 bootstrapped binary MES surfaces (constructed using 151 occurrence records)

B) Binary bootstrapped MESS (95% threshold)

C) Binary bootstrapped MESS (95% threshold) (with 47 outside of range occurrence records)

D) Suggested amended range (incorporating 3 outside of range MESS +ve records)

Thelotornis kirtlandii

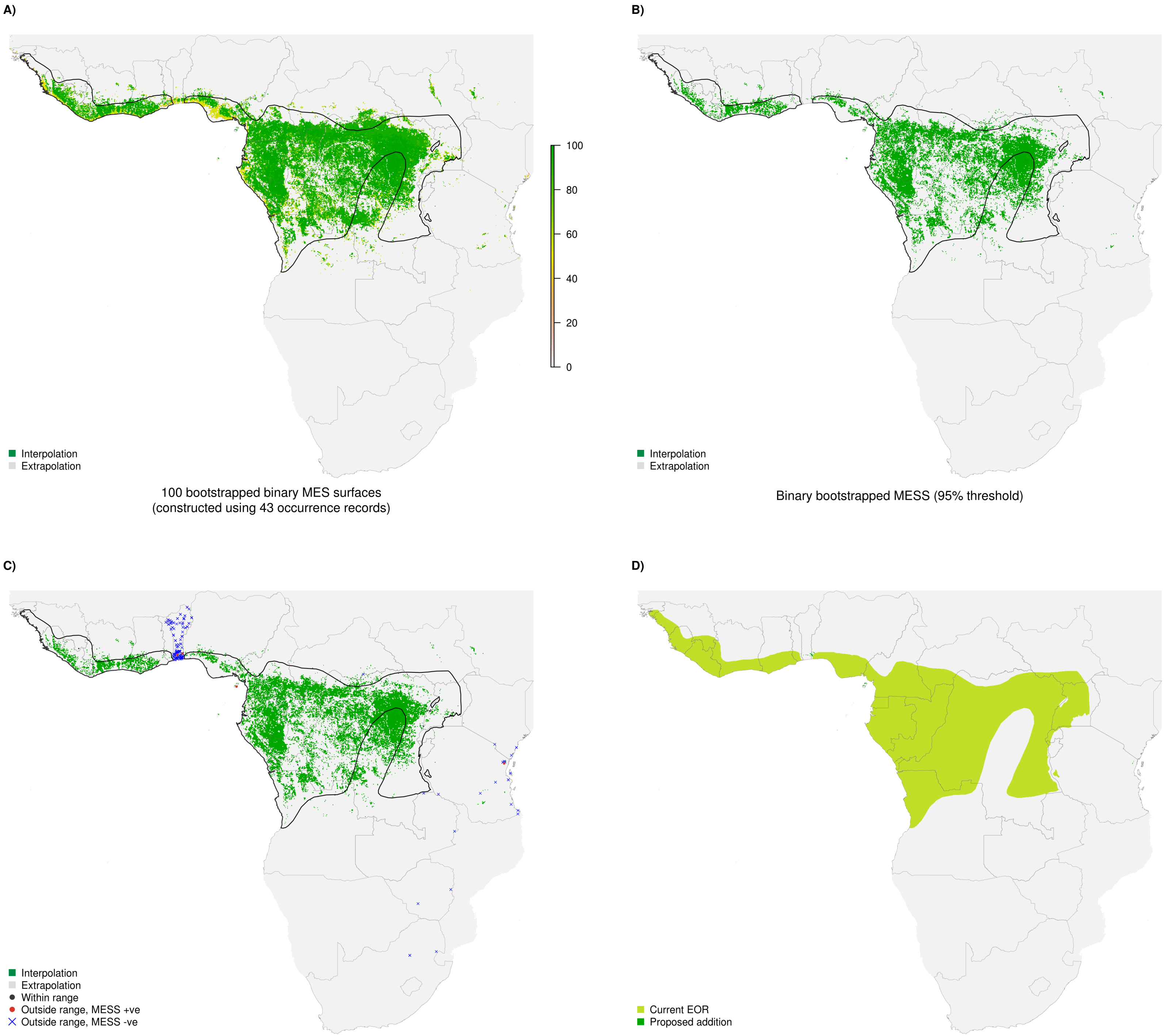

A) 100 bootstrapped binary MES surfaces (constructed using 43 occurrence records)

B) Binary bootstrapped MESS (95% threshold)

C) Binary bootstrapped MESS (95% threshold) (with 154 outside of range occurrence records)

D) Suggested amended range (incorporating 5 outside of range MESS +ve records)

Thelotornis mossambicanus

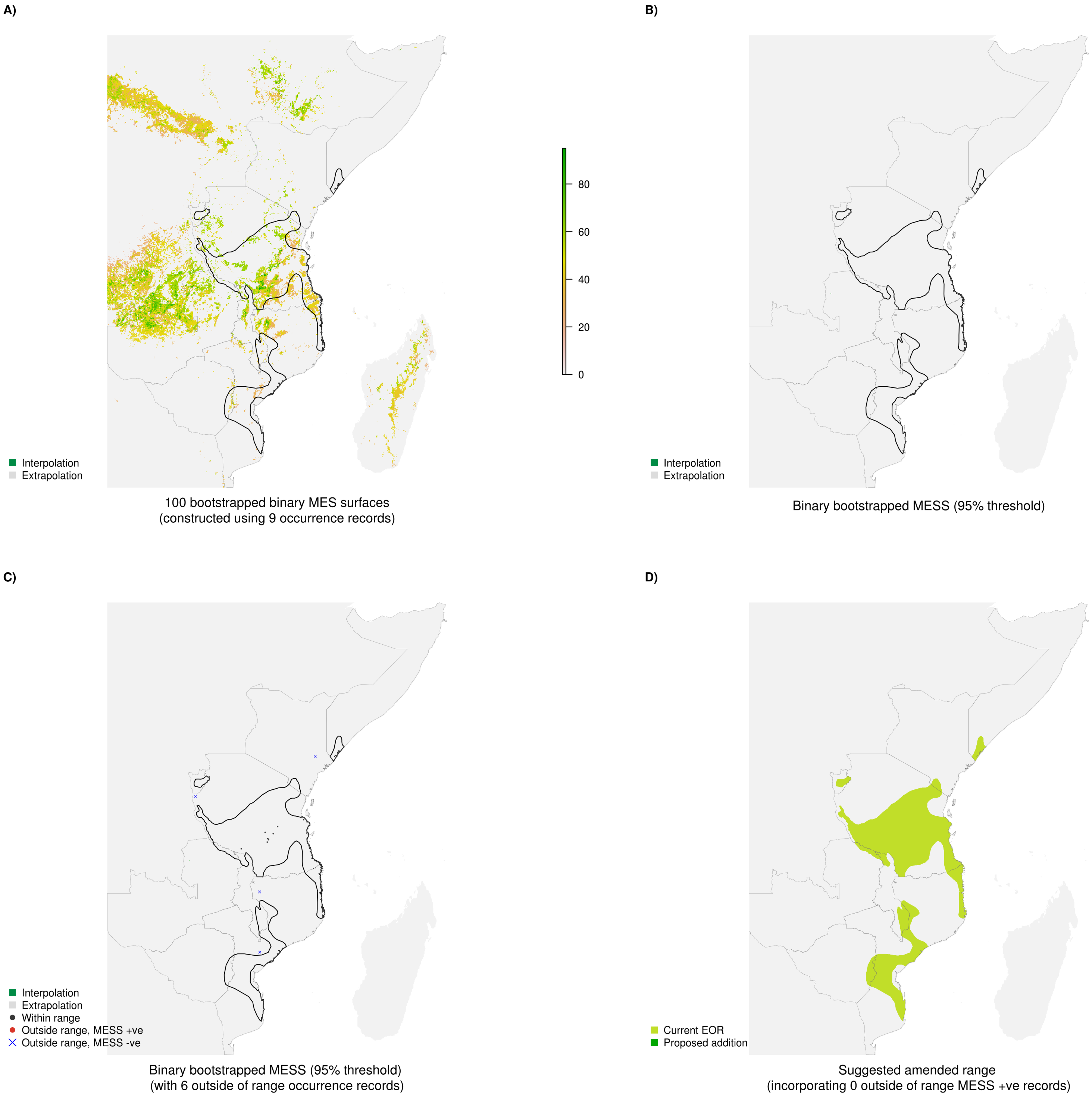

Thelotornis usambaricus

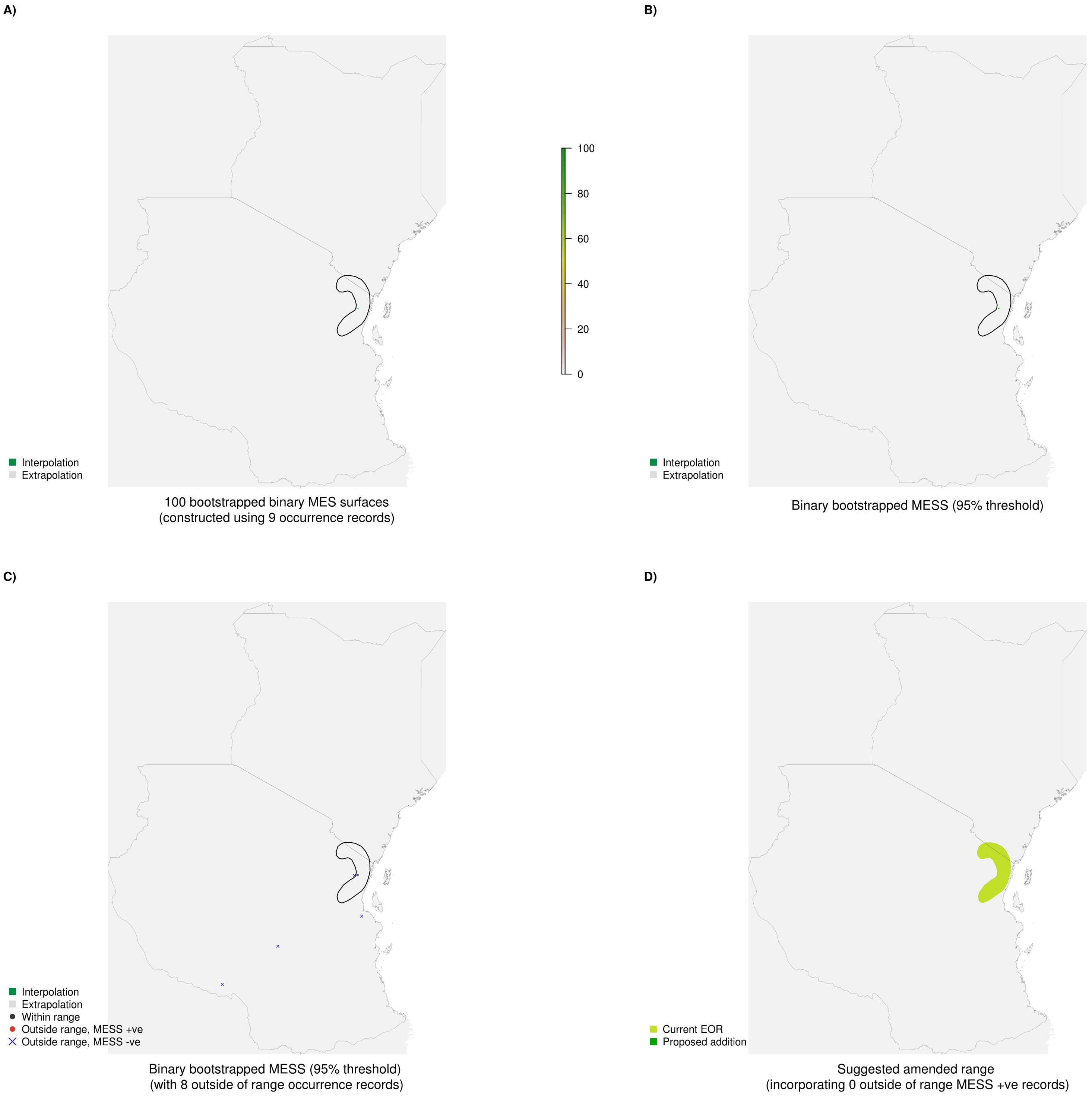

Trimeresurus gramineus

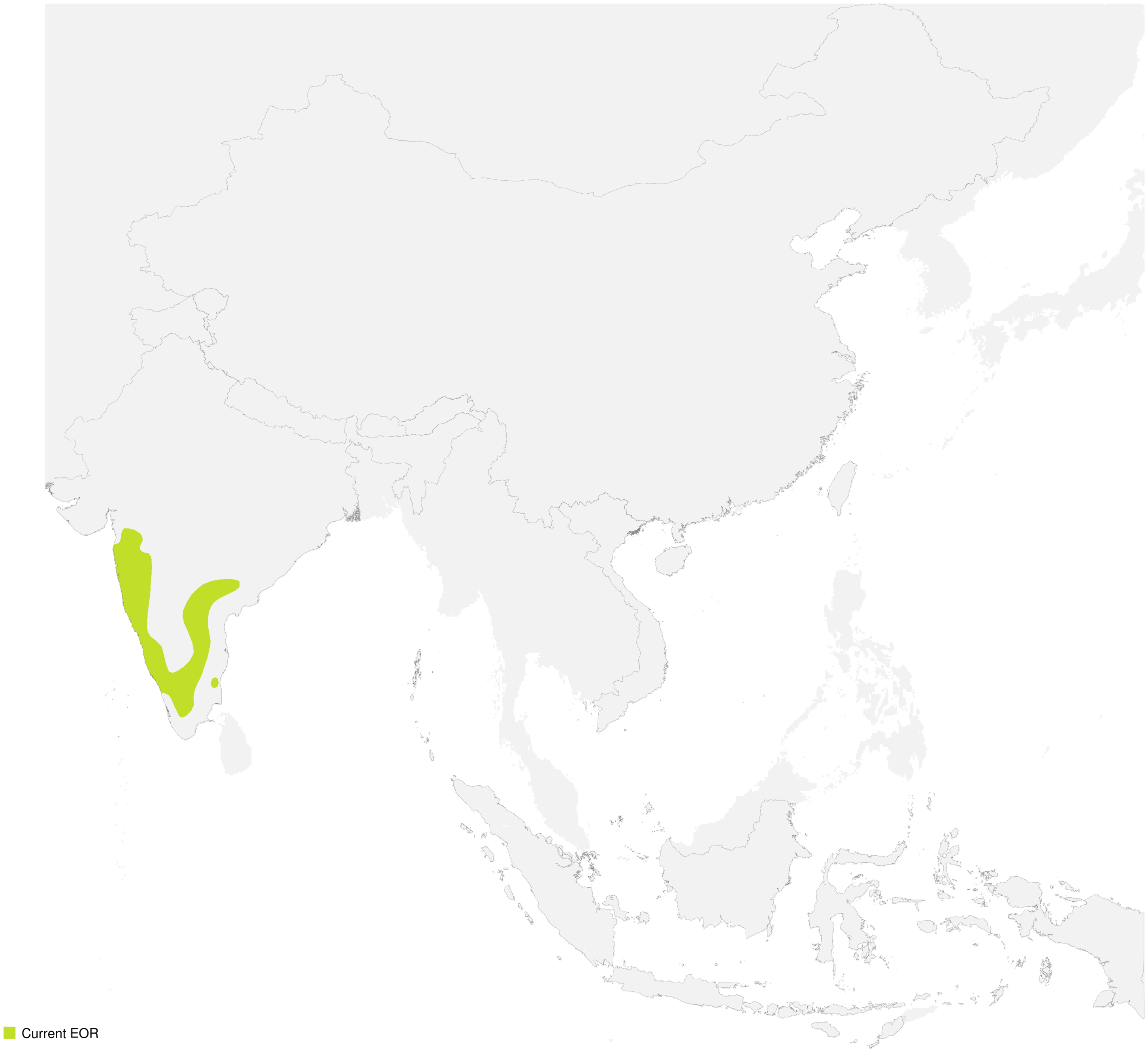

Trimeresurus hageni

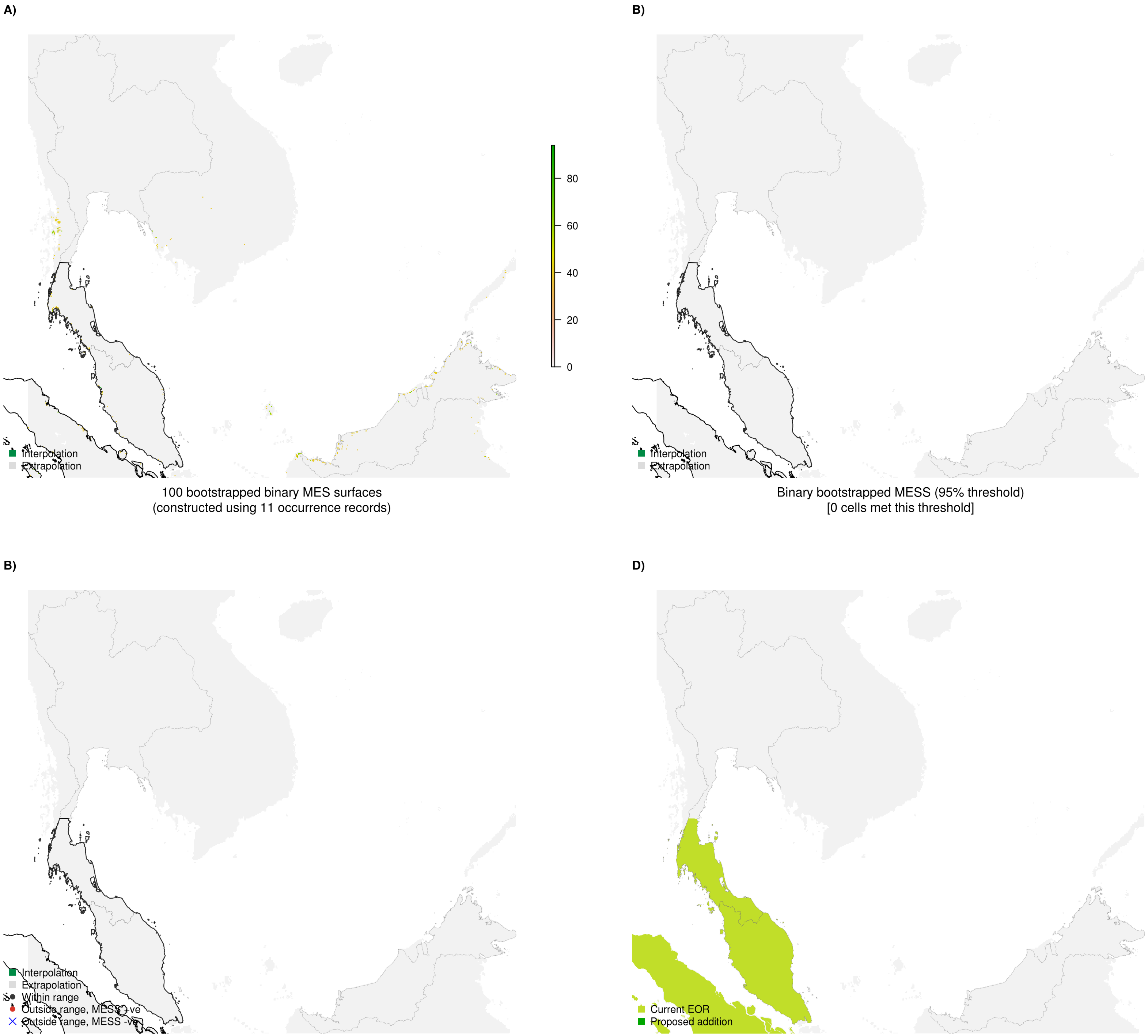

A) 100 bootstrapped binary MES surfaces (constructed using 11 occurrence records)

B) Binary bootstrapped MESS (95% threshold) [0 cells met this threshold]

B) Binary bootstrapped MESS (95% threshold) (with 1 outside of range occurrence records)

D) Suggested amended range (incorporating 0 outside of range MESS +ve records)

Trimeresurus malabaricus

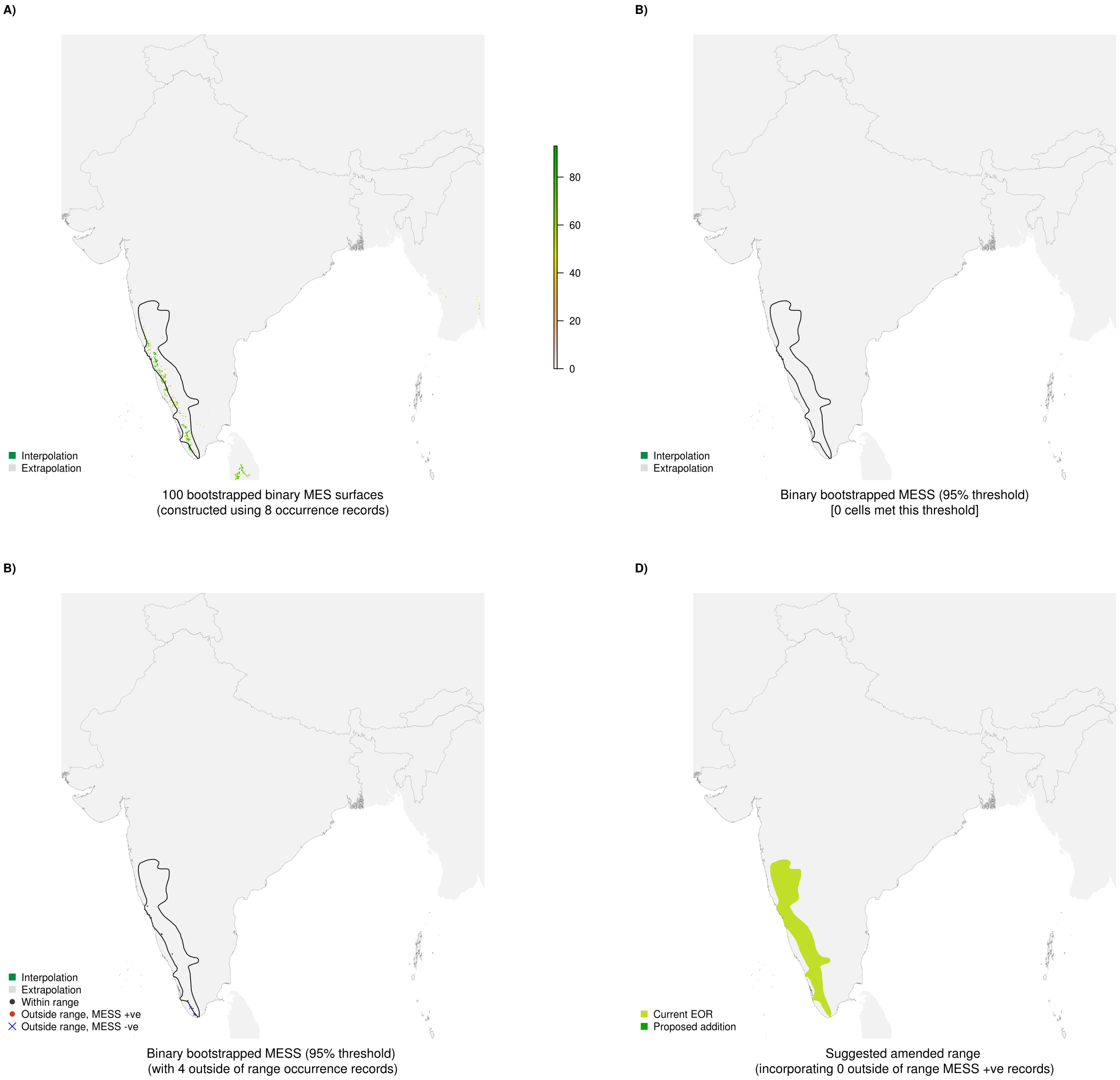

Trimeresurus trigonocephalus

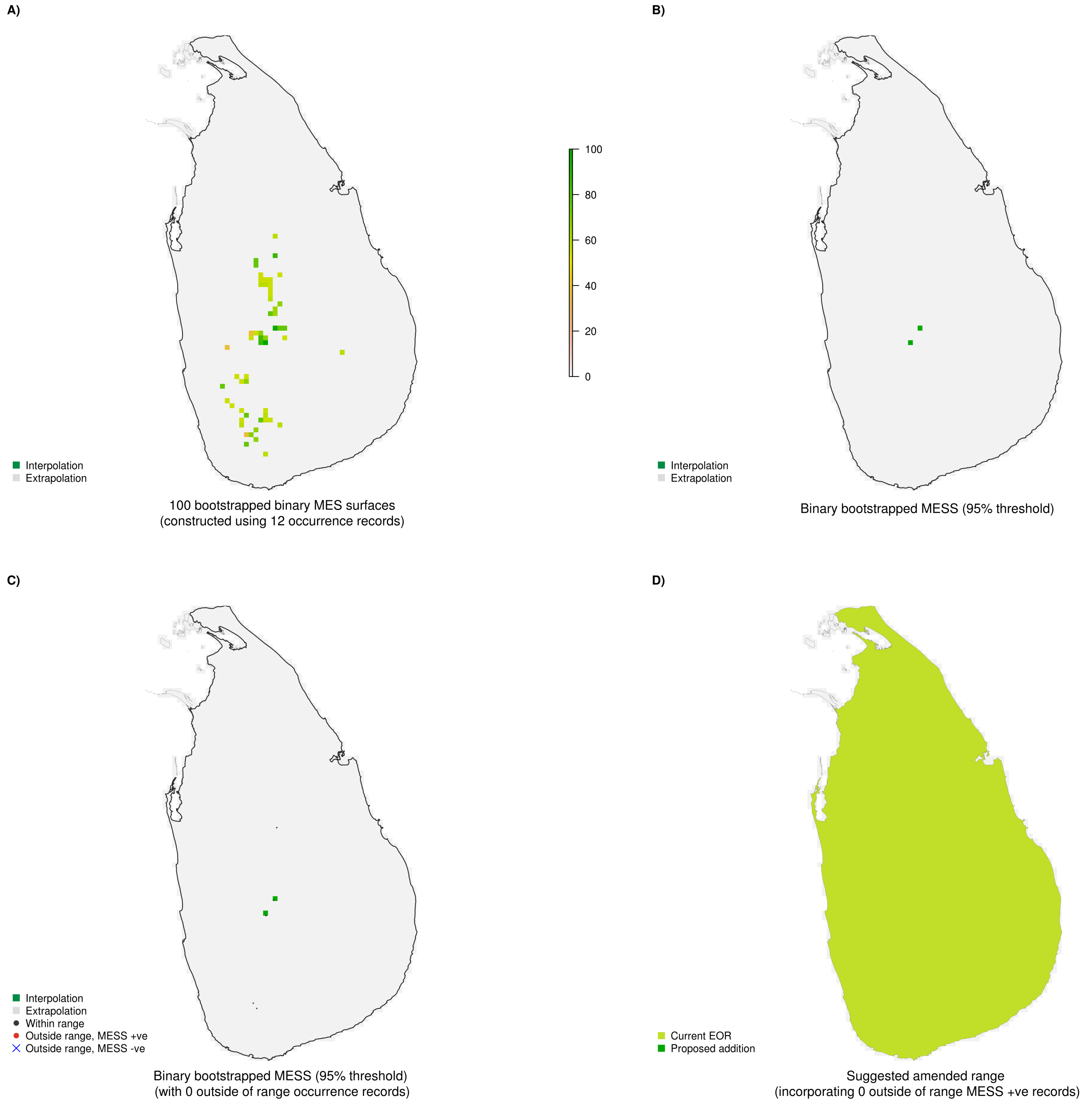

Tropidechis carinatus

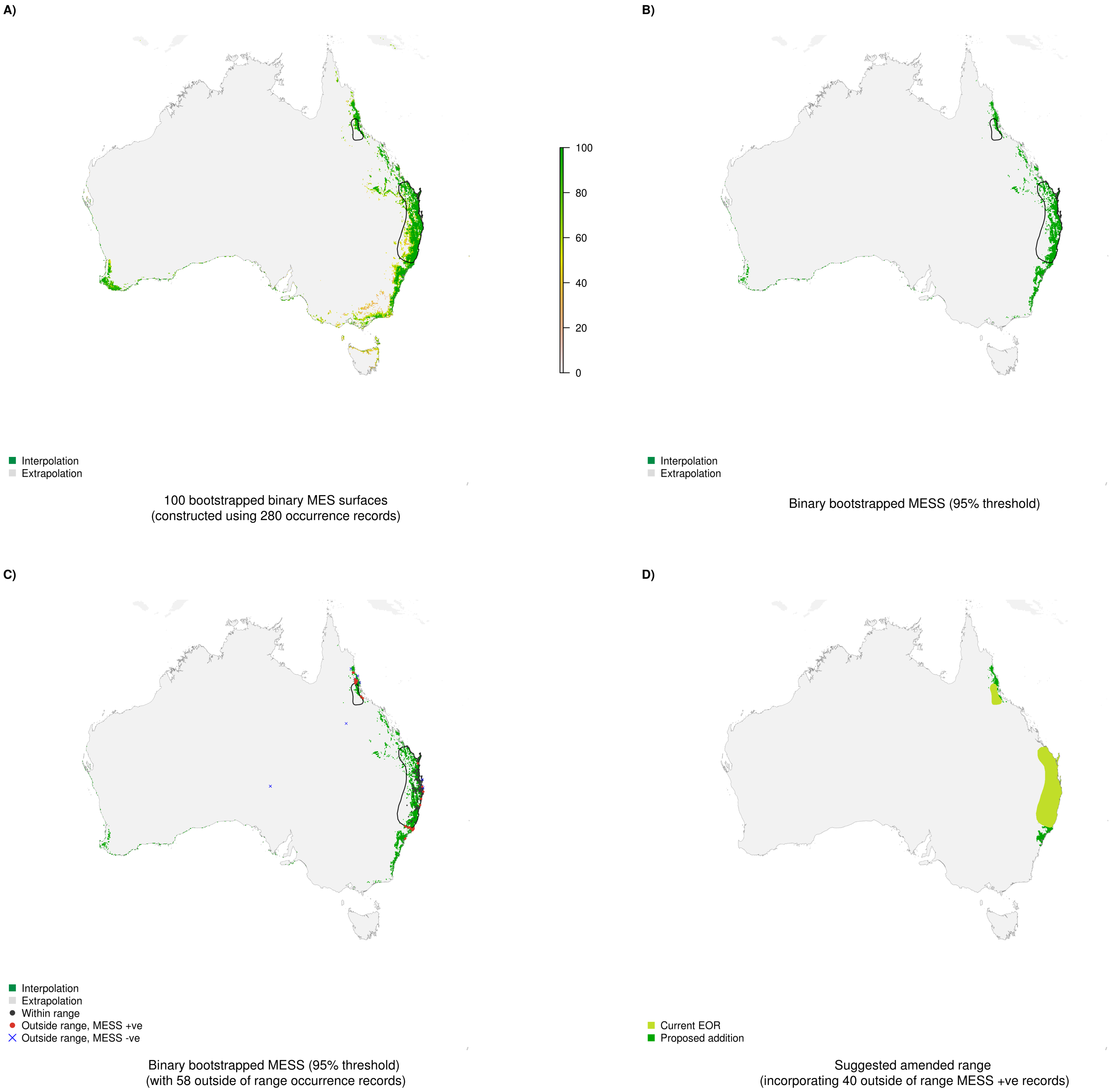

Tropidolaemus philippensis

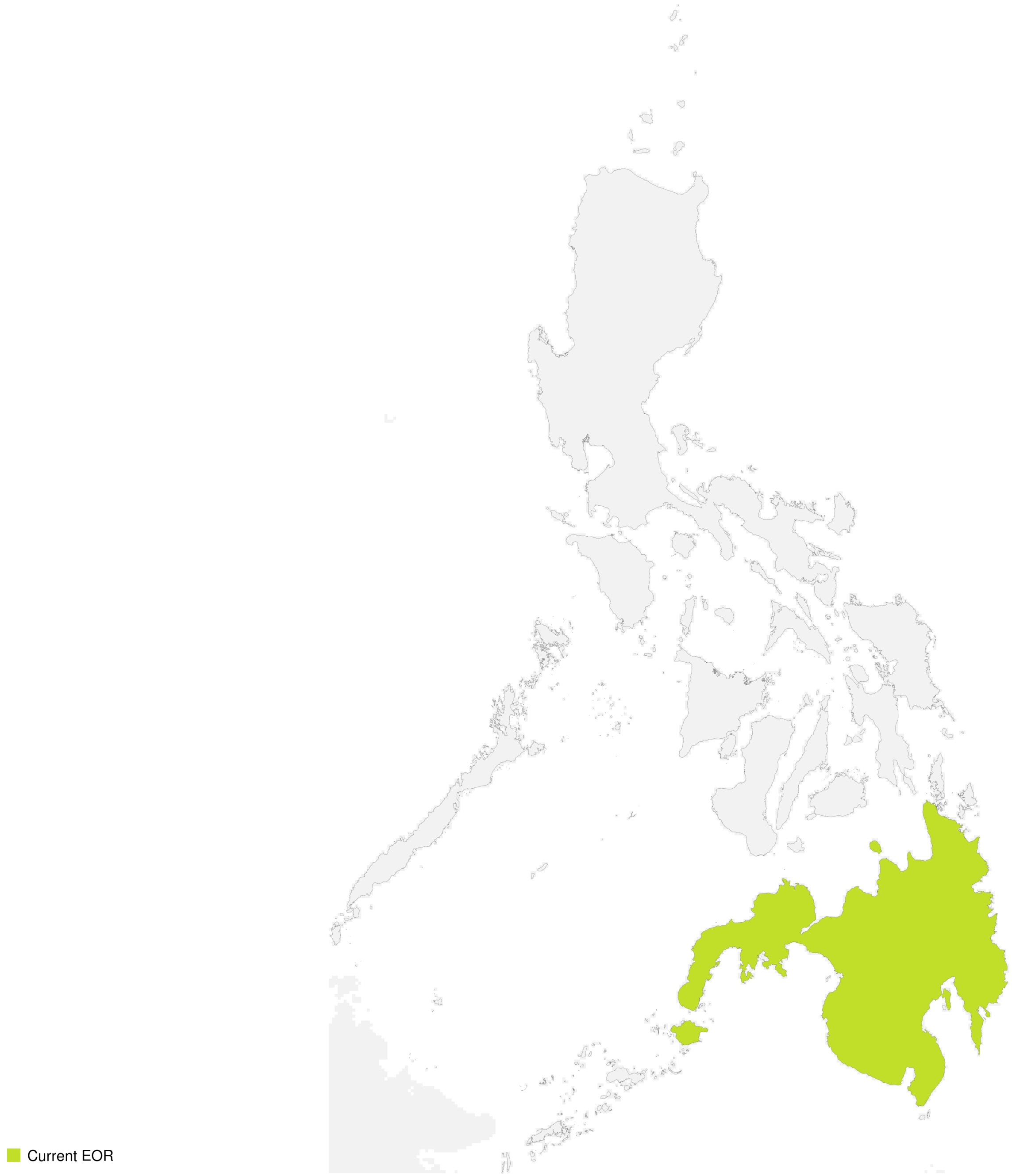

Tropidolaemus subannulatus

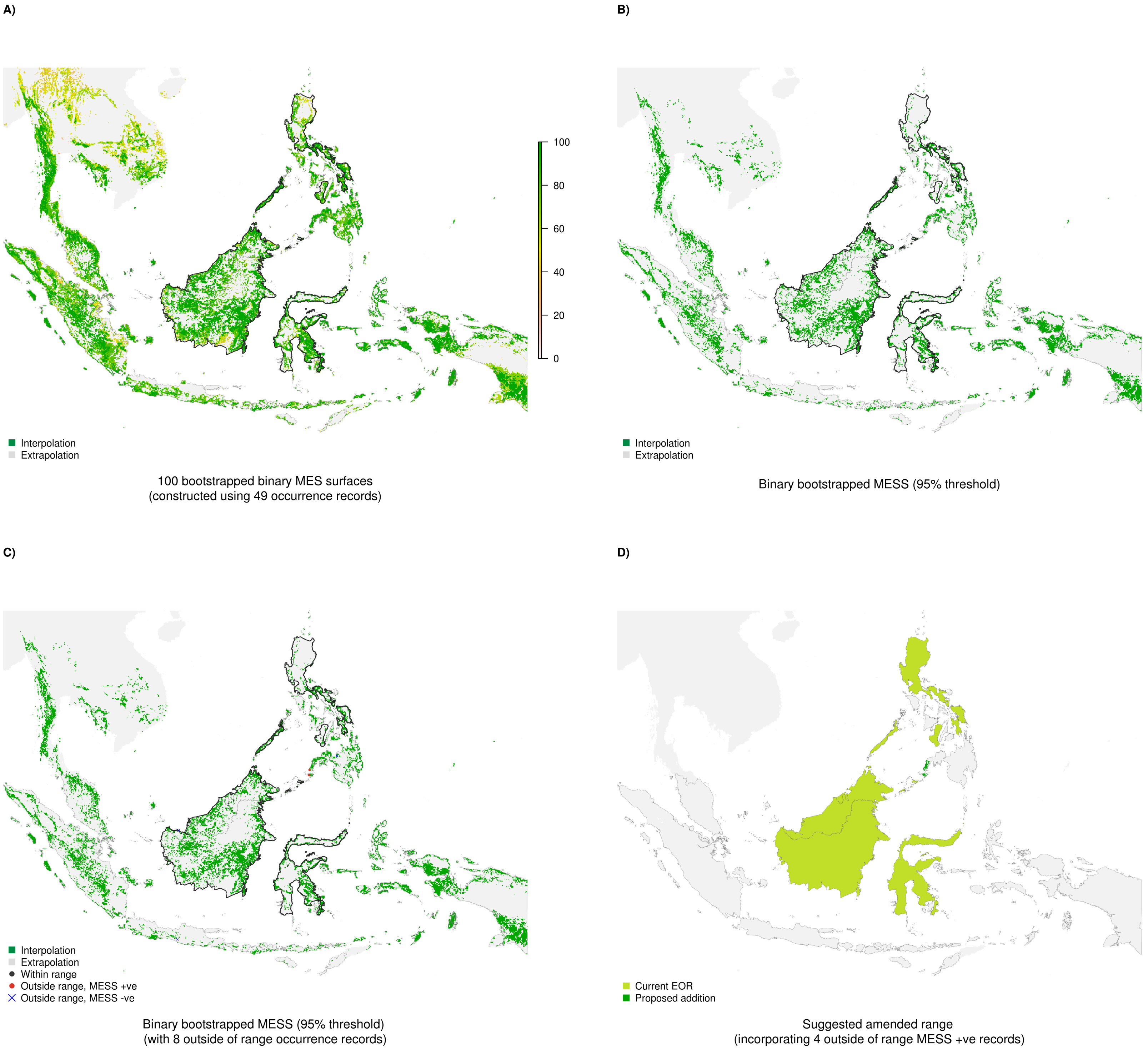

Vipera ammodytes

A)

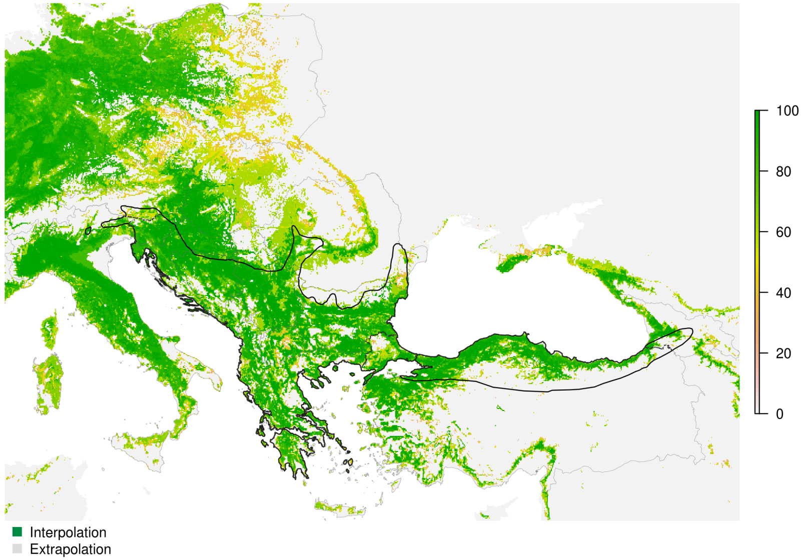

100 bootstrapped binary MES surfaces
(constructed using 32 occurrence records)

B)

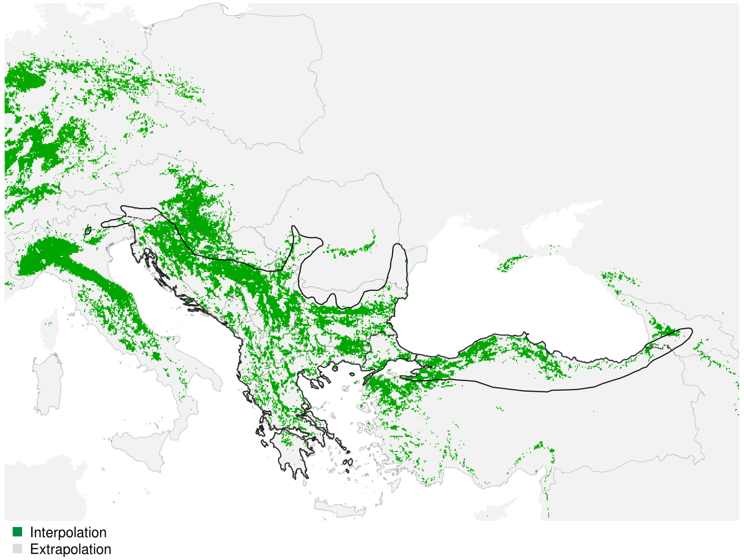

Binary bootstrapped MESS (95% threshold)

C)

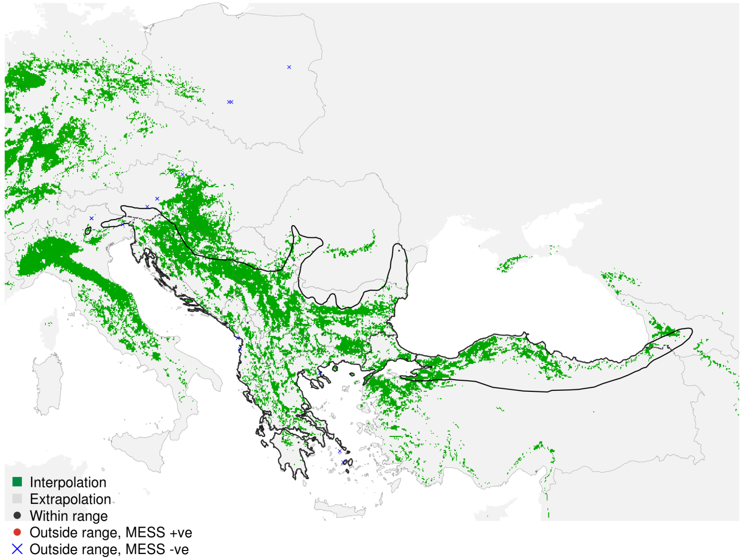

Binary bootstrapped MESS (95% threshold)
(with 17 outside of range occurrence records)

D)

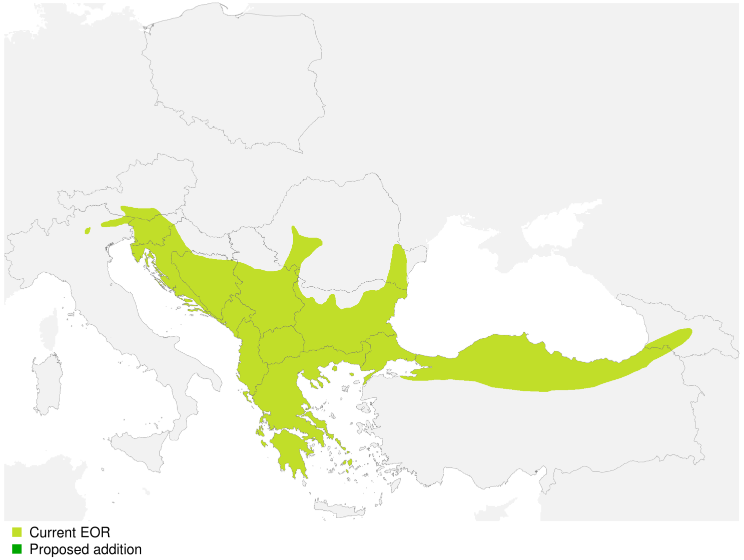

Suggested amended range
(incorporating 0 outside of range MESS +ve records)

Vipera aspis

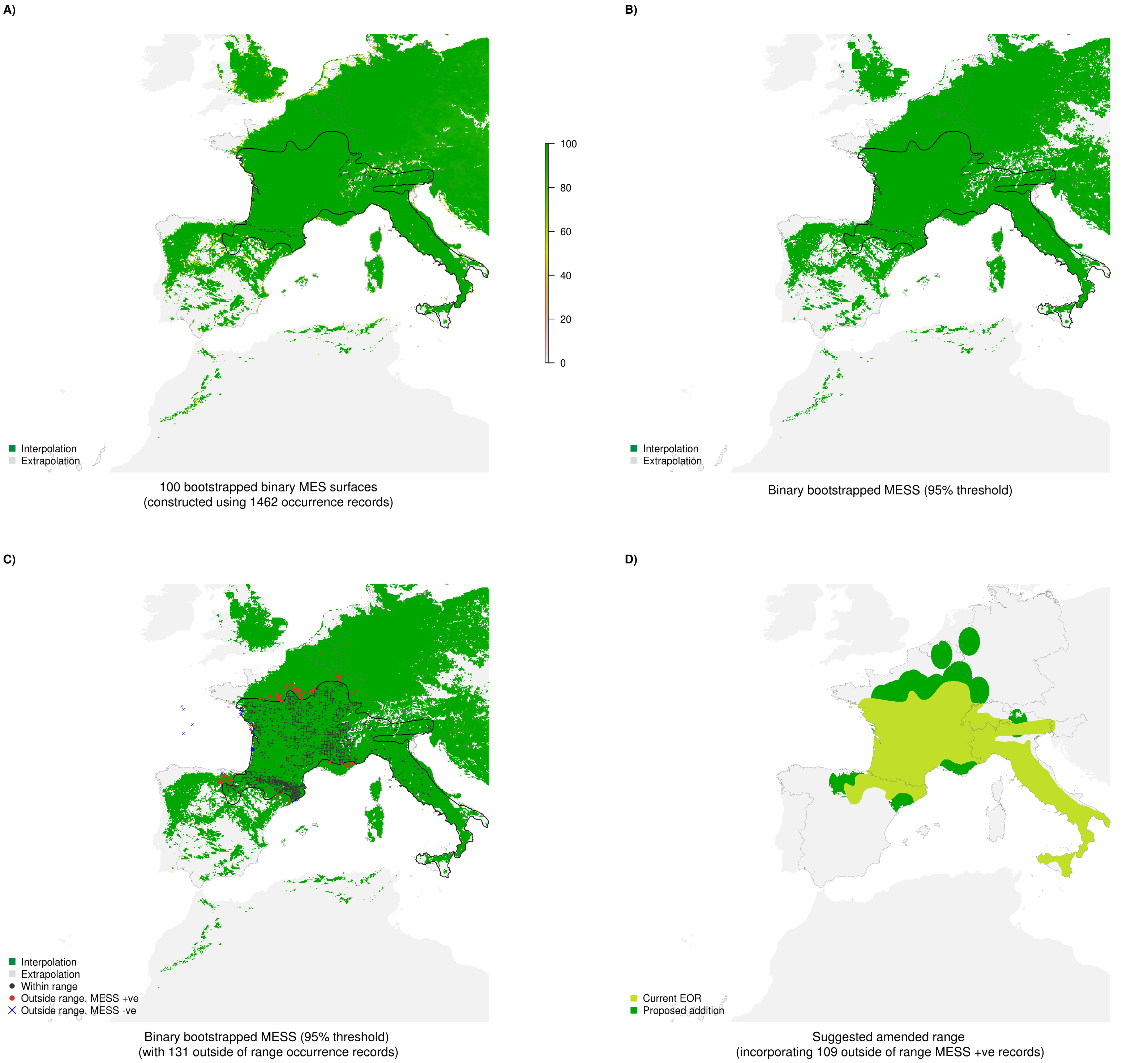

Vipera berus

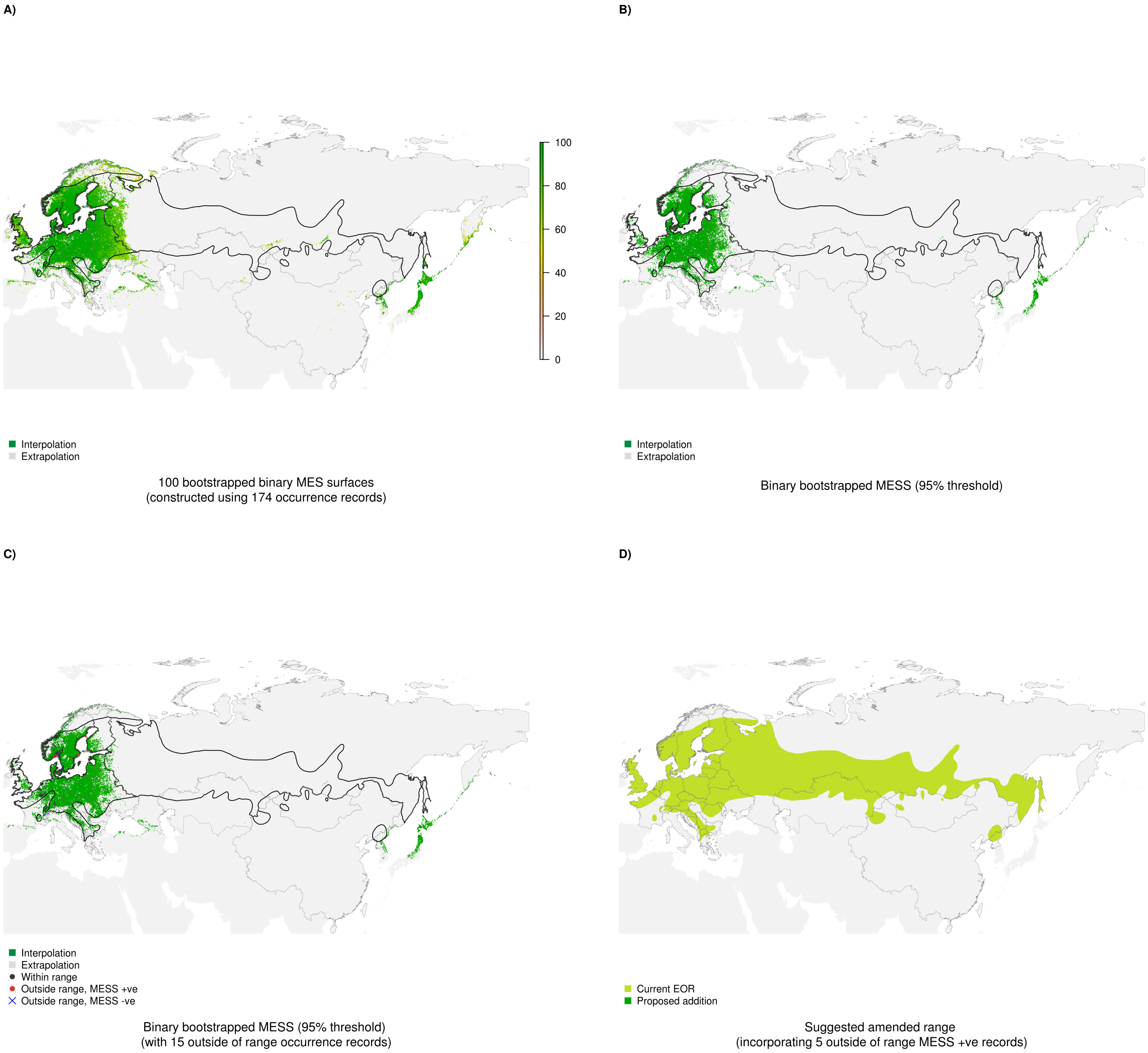

Vipera darevskii

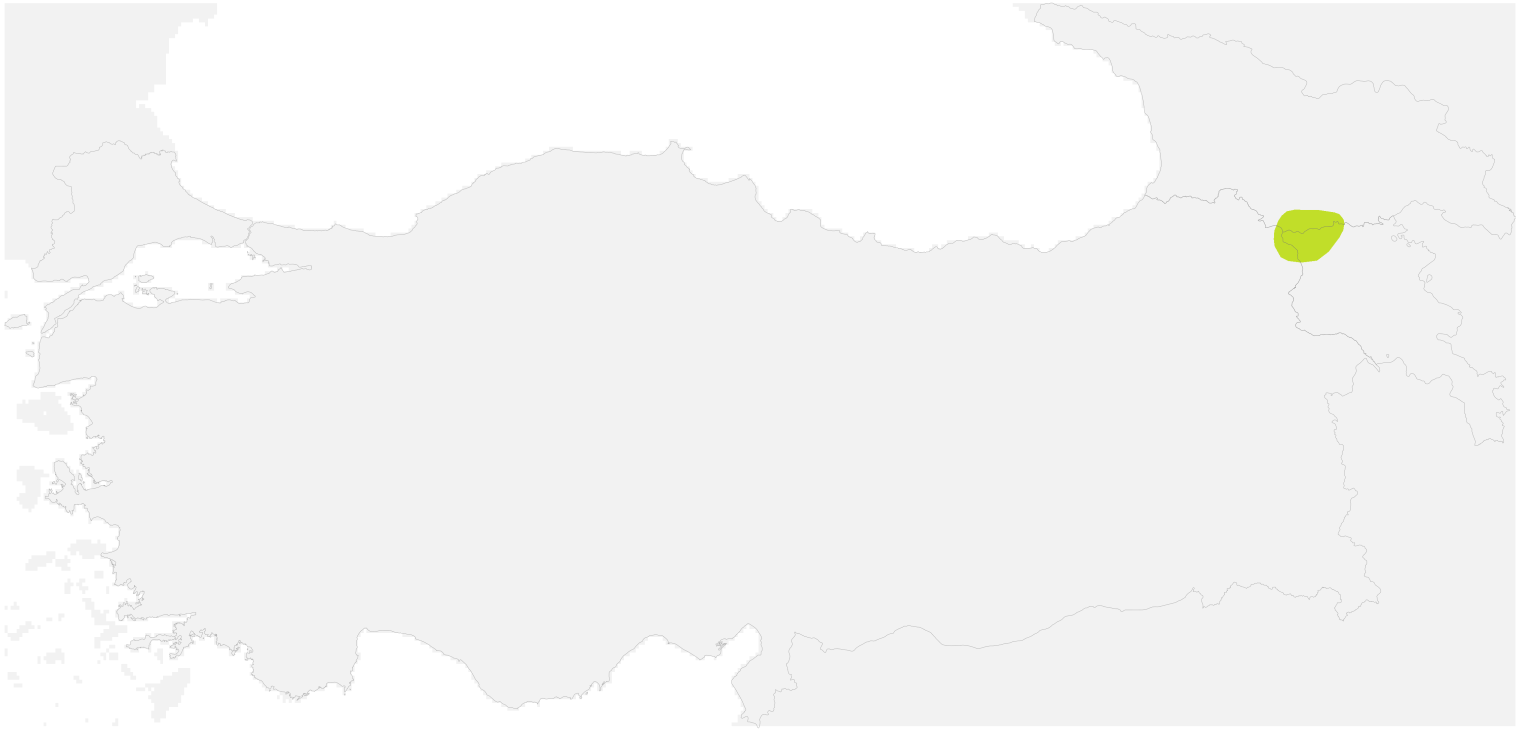

Current EOR

Vipera ebneri

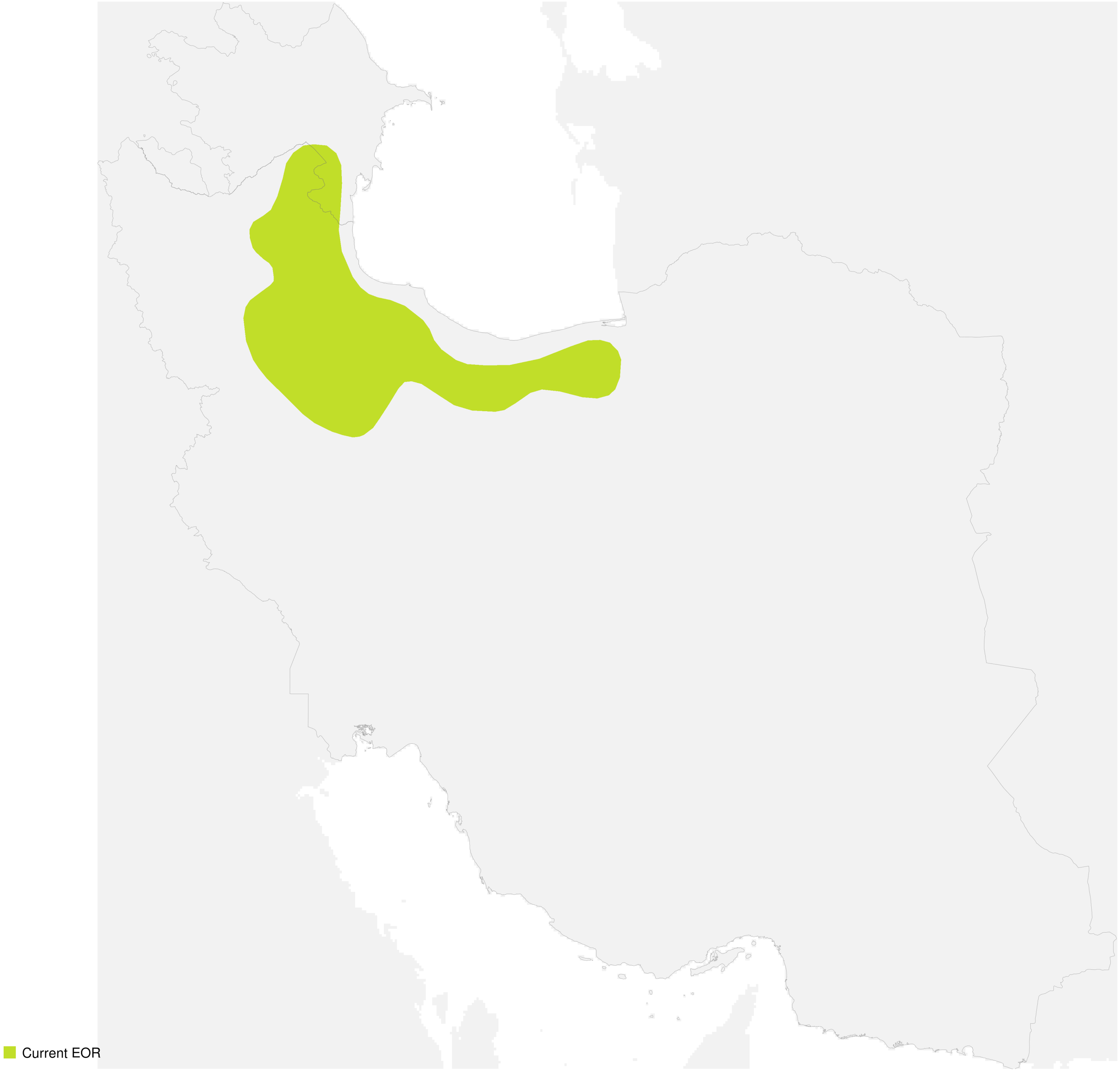

Vipera eriwanensis

A)

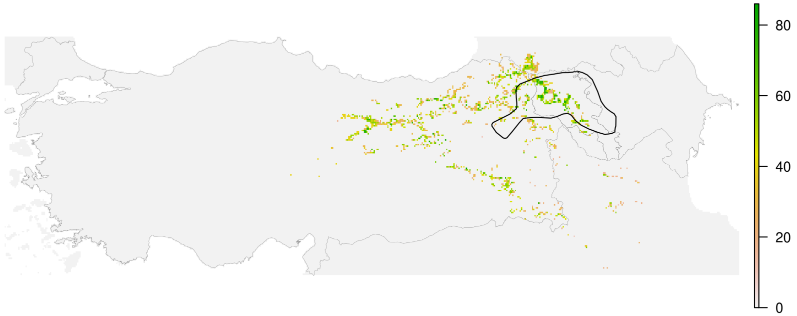

- Interpolation
- Extrapolation

100 bootstrapped binary MES surfaces
(constructed using 5 occurrence records)

B)

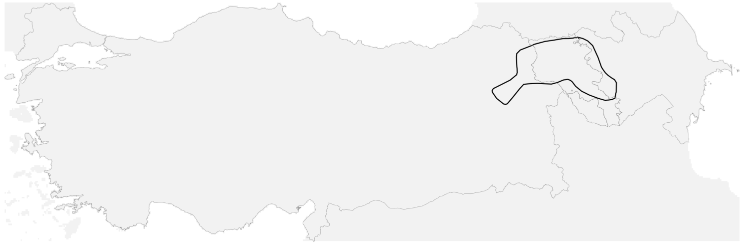

- Interpolation
- Extrapolation

Binary bootstrapped MESS (95% threshold)
[0 cells met this threshold]

B)

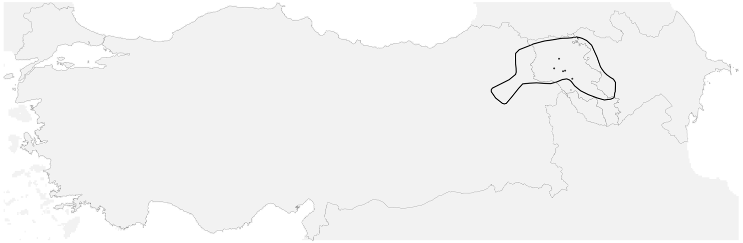

- Interpolation
- Extrapolation
- Within range
- Outside range, MESS +ve
- Outside range, MESS -ve

Binary bootstrapped MESS (95% threshold)
(with 0 outside of range occurrence records)

D)

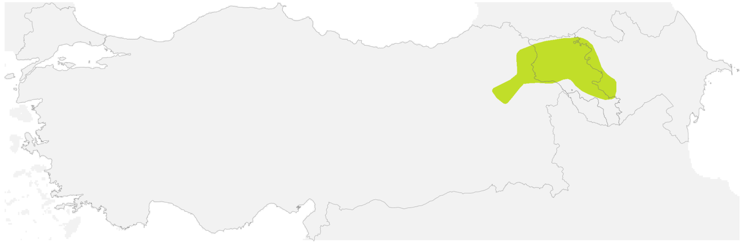

- Current EOR
- Proposed addition

Suggested amended range
(incorporating 0 outside of range MESS +ve records)

Vipera kaznakovi

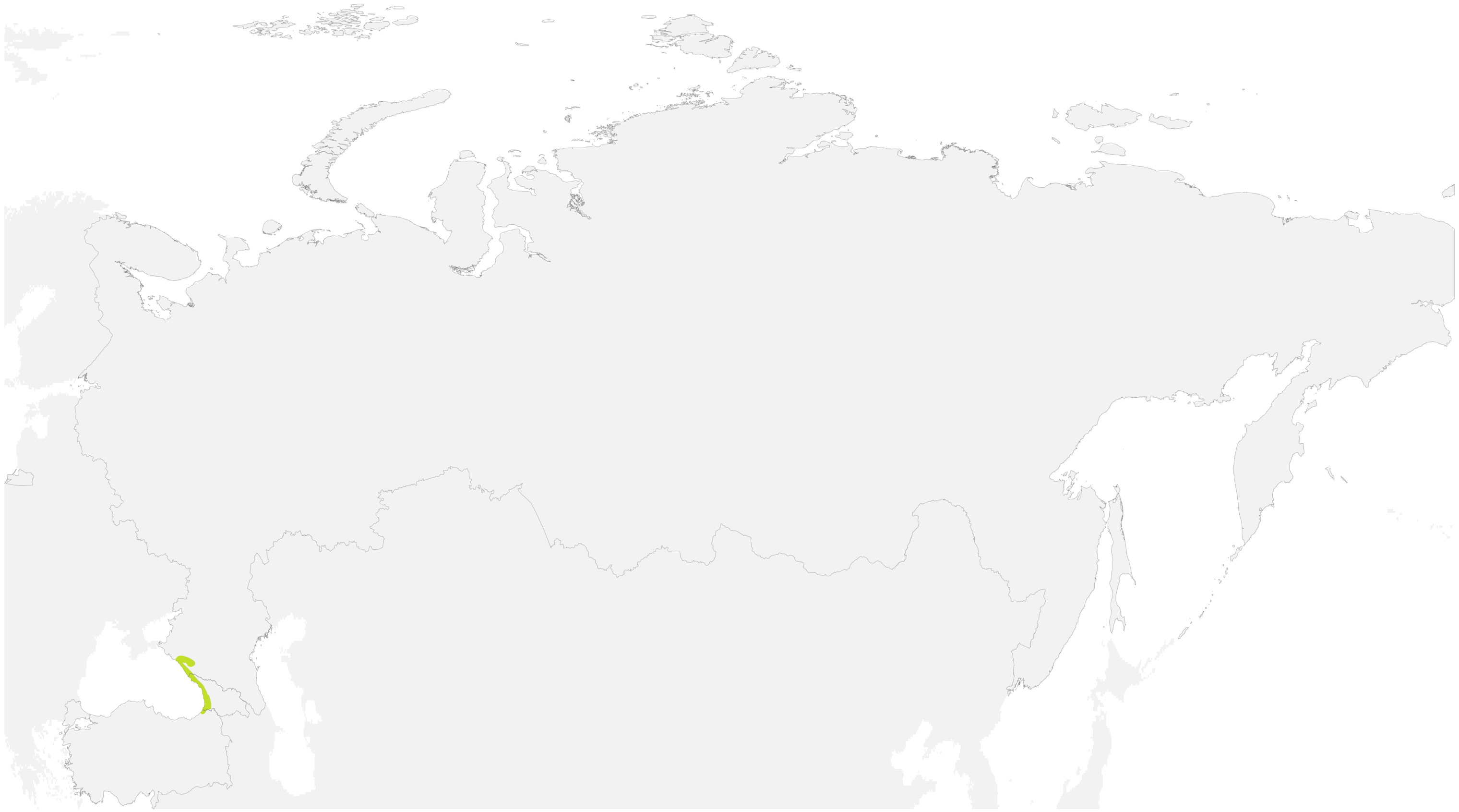

Current EOR

Vipera latastei

A)

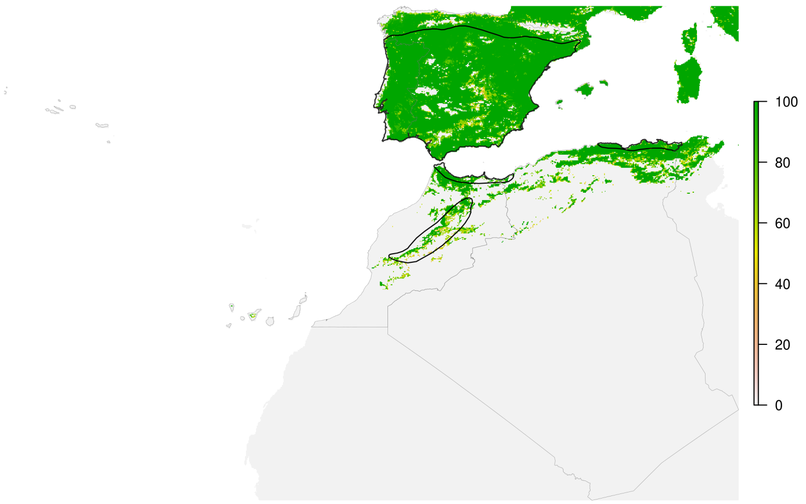

Interpolation
Extrapolation

100 bootstrapped binary MES surfaces
(constructed using 638 occurrence records)

B)

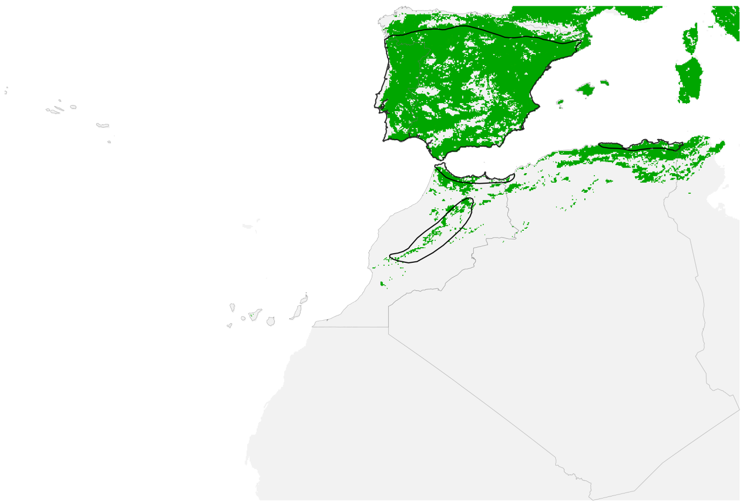

Interpolation
Extrapolation

Binary bootstrapped MESS (95% threshold)

C)

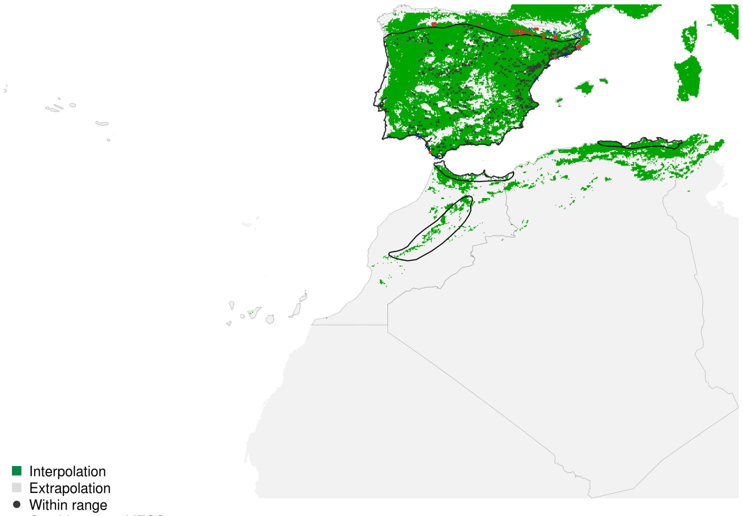

Interpolation
Extrapolation
Within range
Outside range, MESS +ve
Outside range, MESS -ve

Binary bootstrapped MESS (95% threshold)
(with 47 outside of range occurrence records)

D)

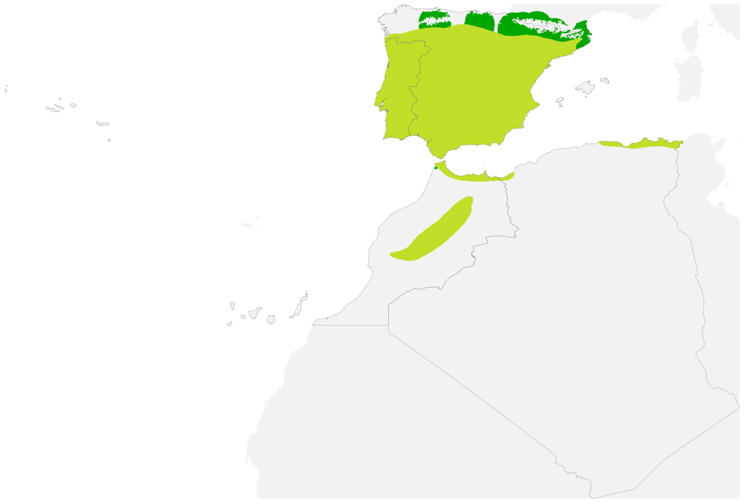

Current EOR
Proposed addition

Suggested amended range
(incorporating 29 outside of range MESS +ve records)

Vipera lotievi

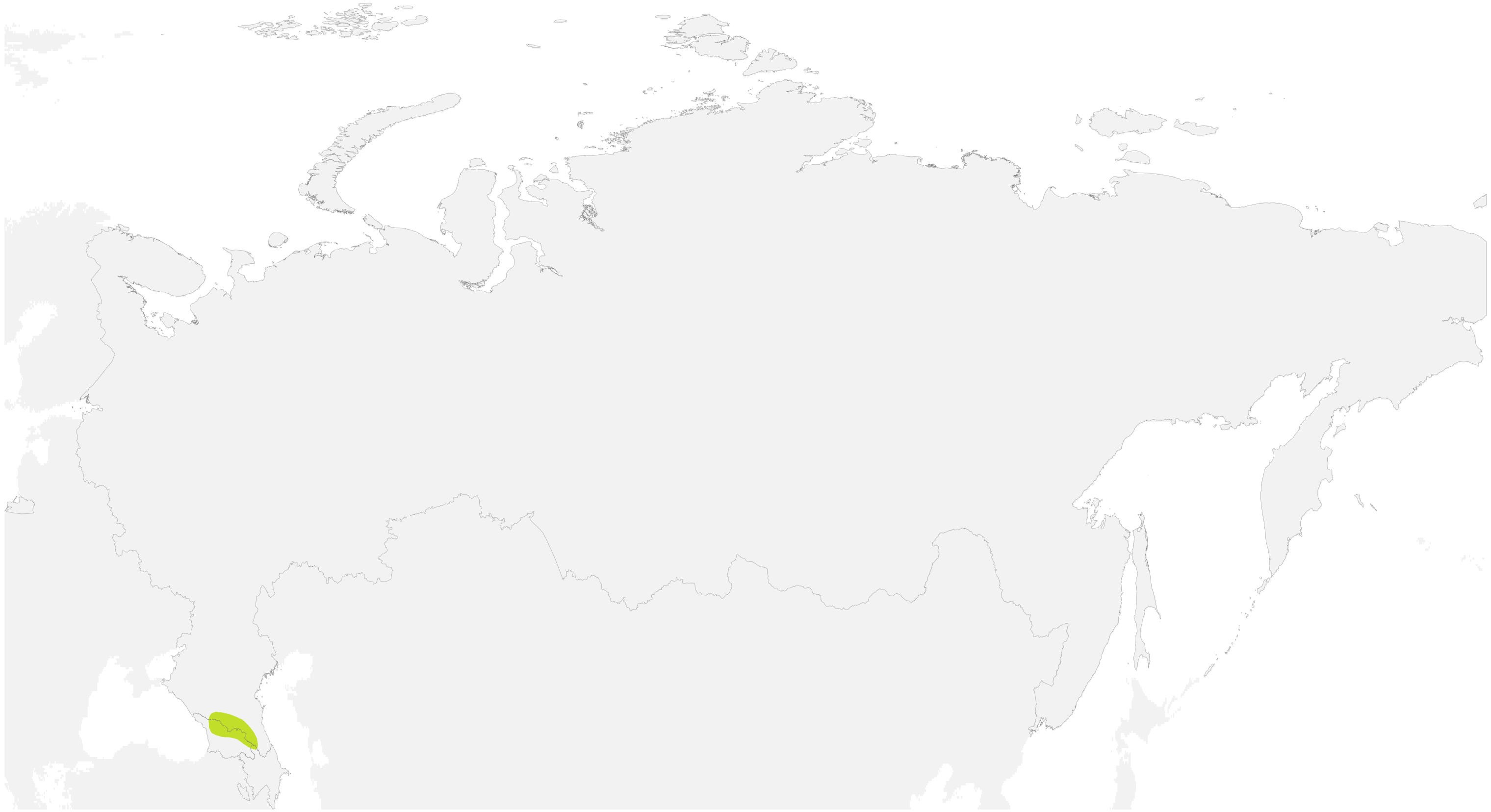

Current EOR

Vipera nikolskii

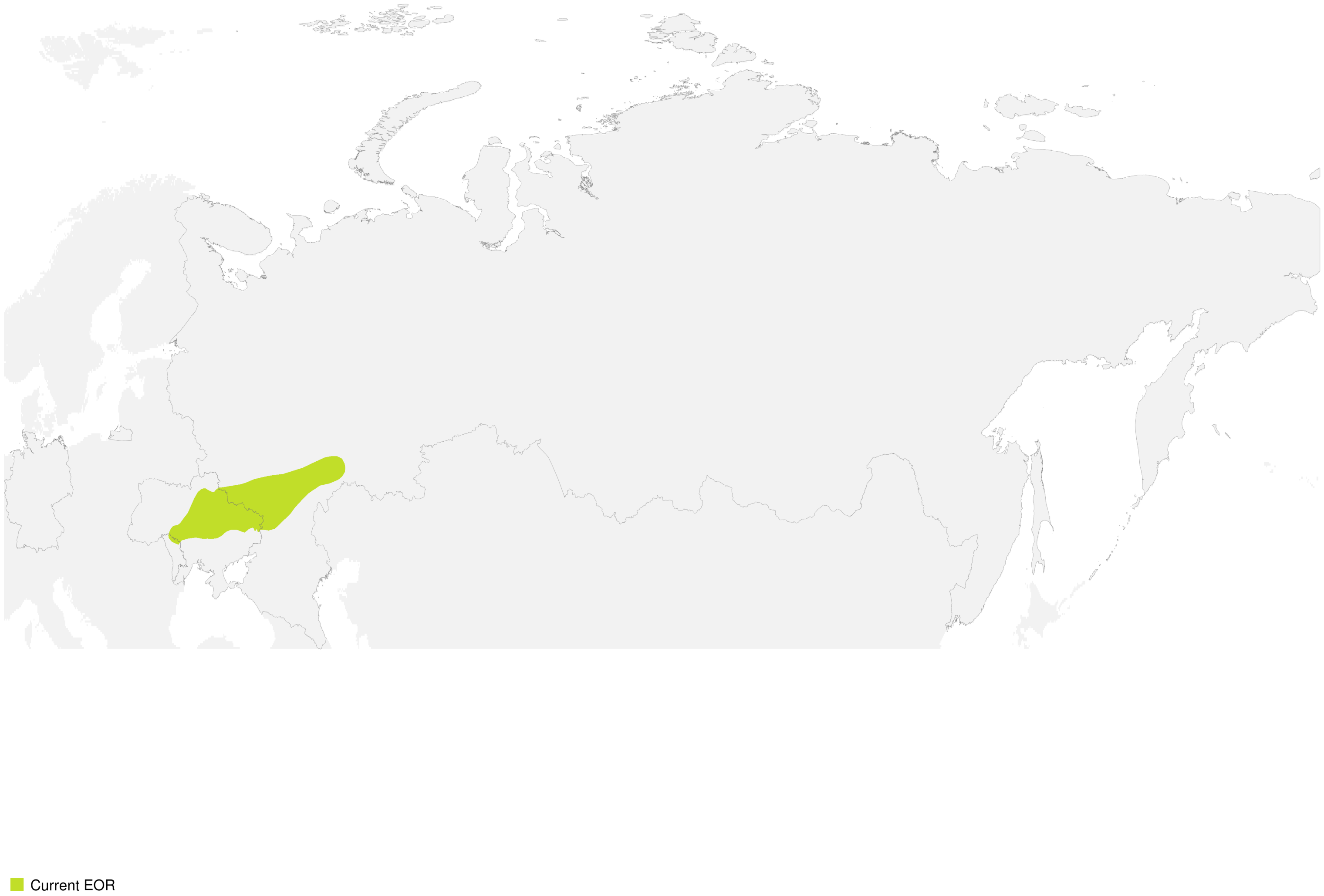

Vipera renardi

A)

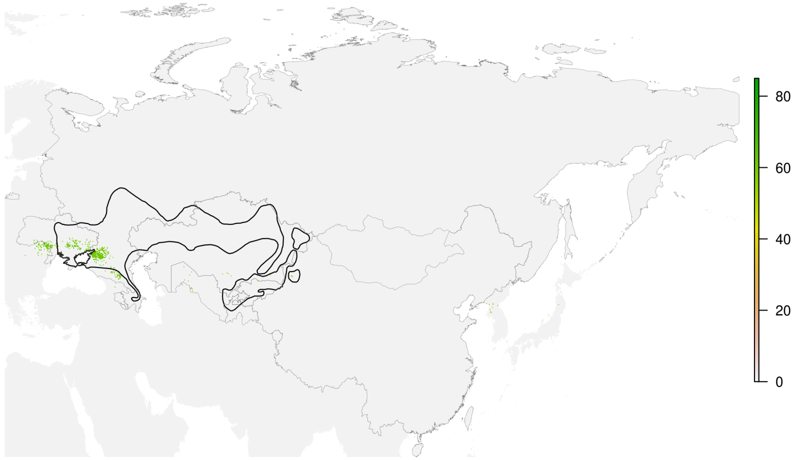

Interpolation
Extrapolation

100 bootstrapped binary MES surfaces
(constructed using 5 occurrence records)

B)

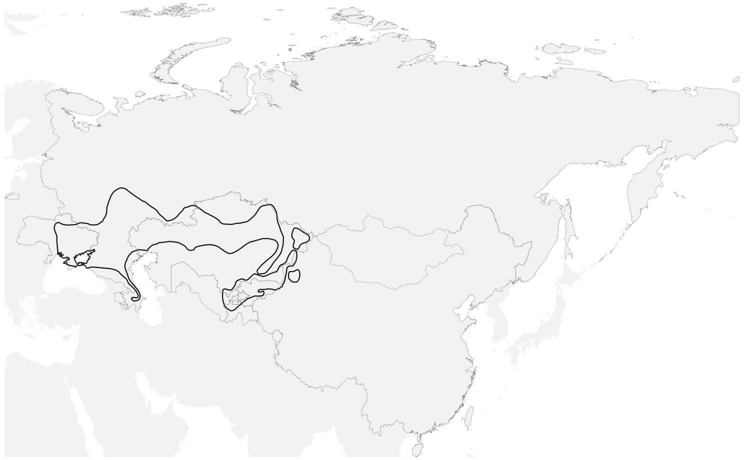

Interpolation
Extrapolation

Binary bootstrapped MESS (95% threshold)
[0 cells met this threshold]

B)

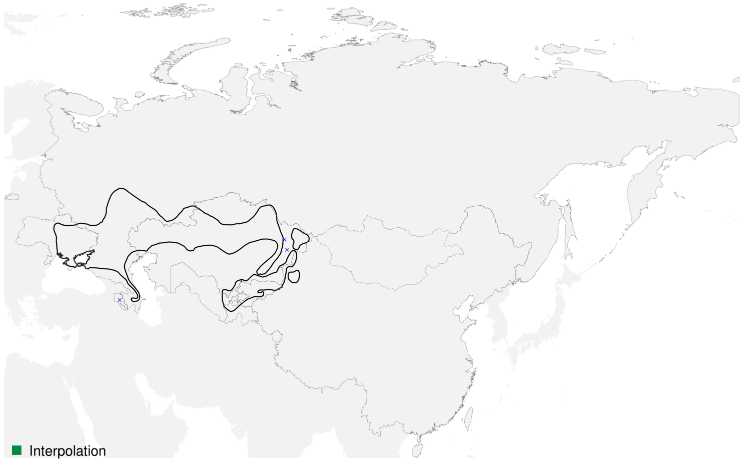

Interpolation
Extrapolation
Within range
Outside range, MESS +ve
Outside range, MESS -ve

Binary bootstrapped MESS (95% threshold)
(with 3 outside of range occurrence records)

D)

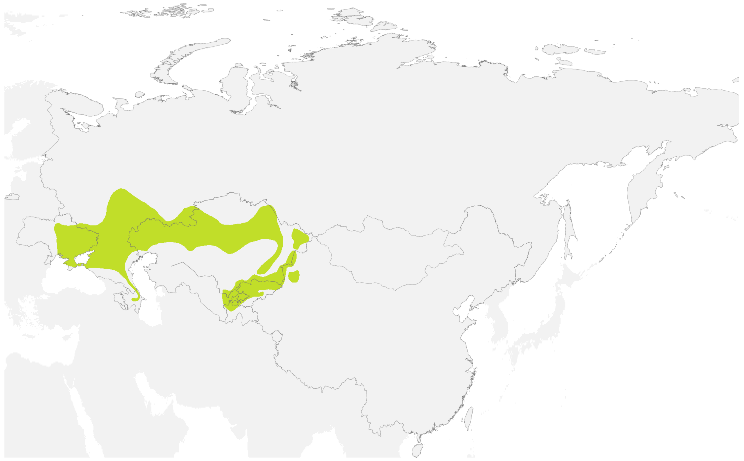

Current EOR
Proposed addition

Suggested amended range
(incorporating 0 outside of range MESS +ve records)

Vipera seoanei

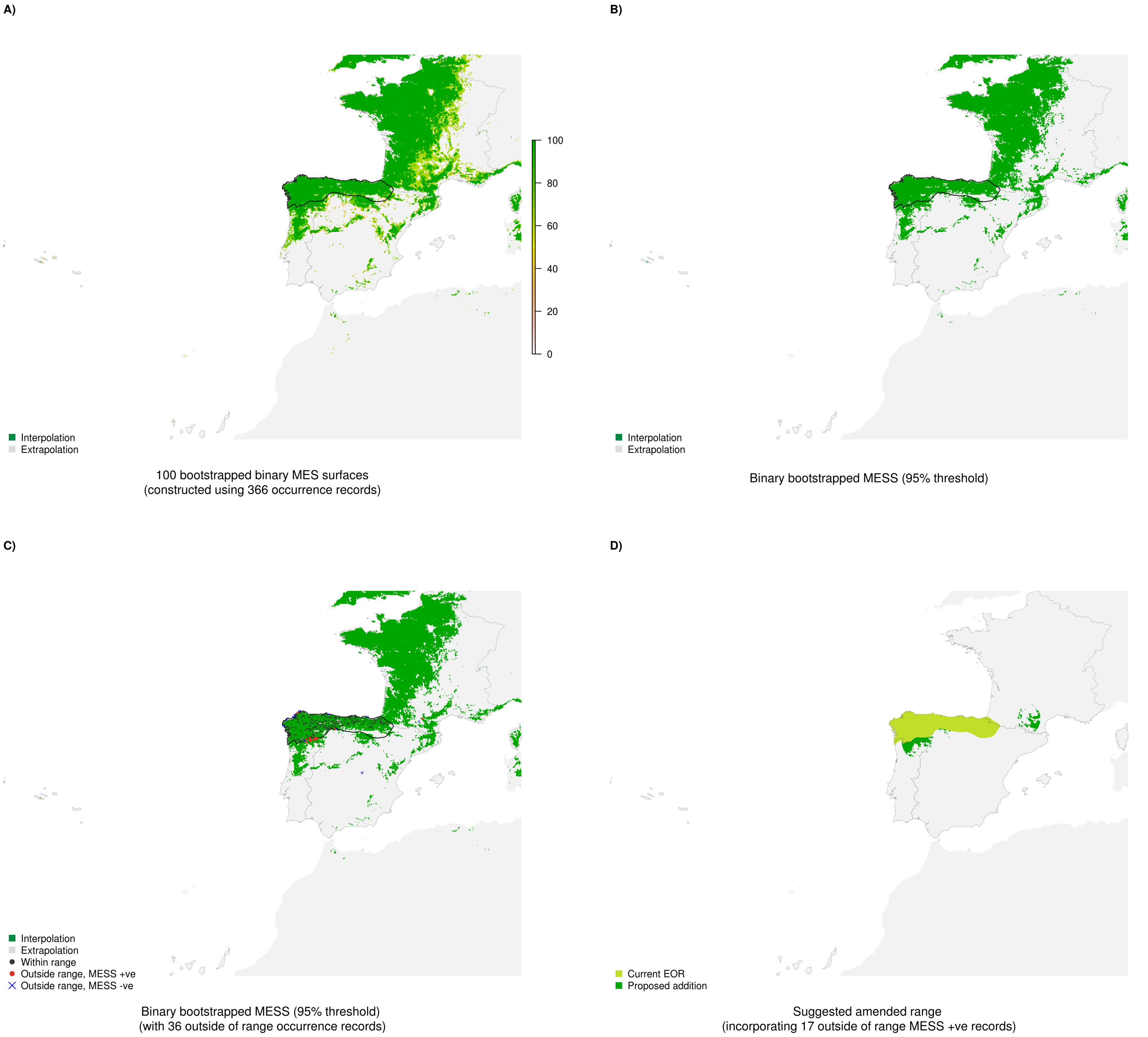

Vipera ursinii

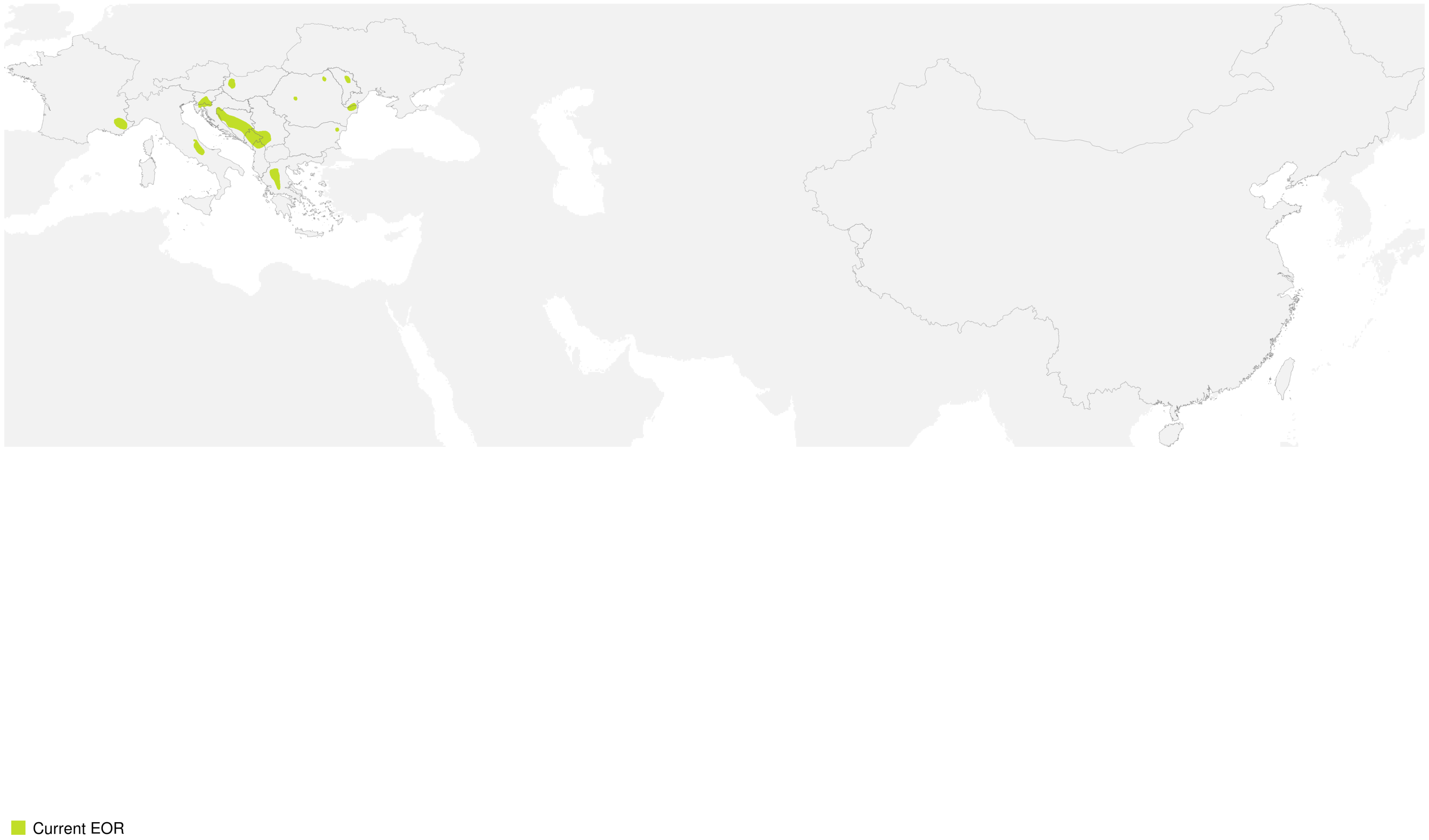

Viridovipera stejnegeri

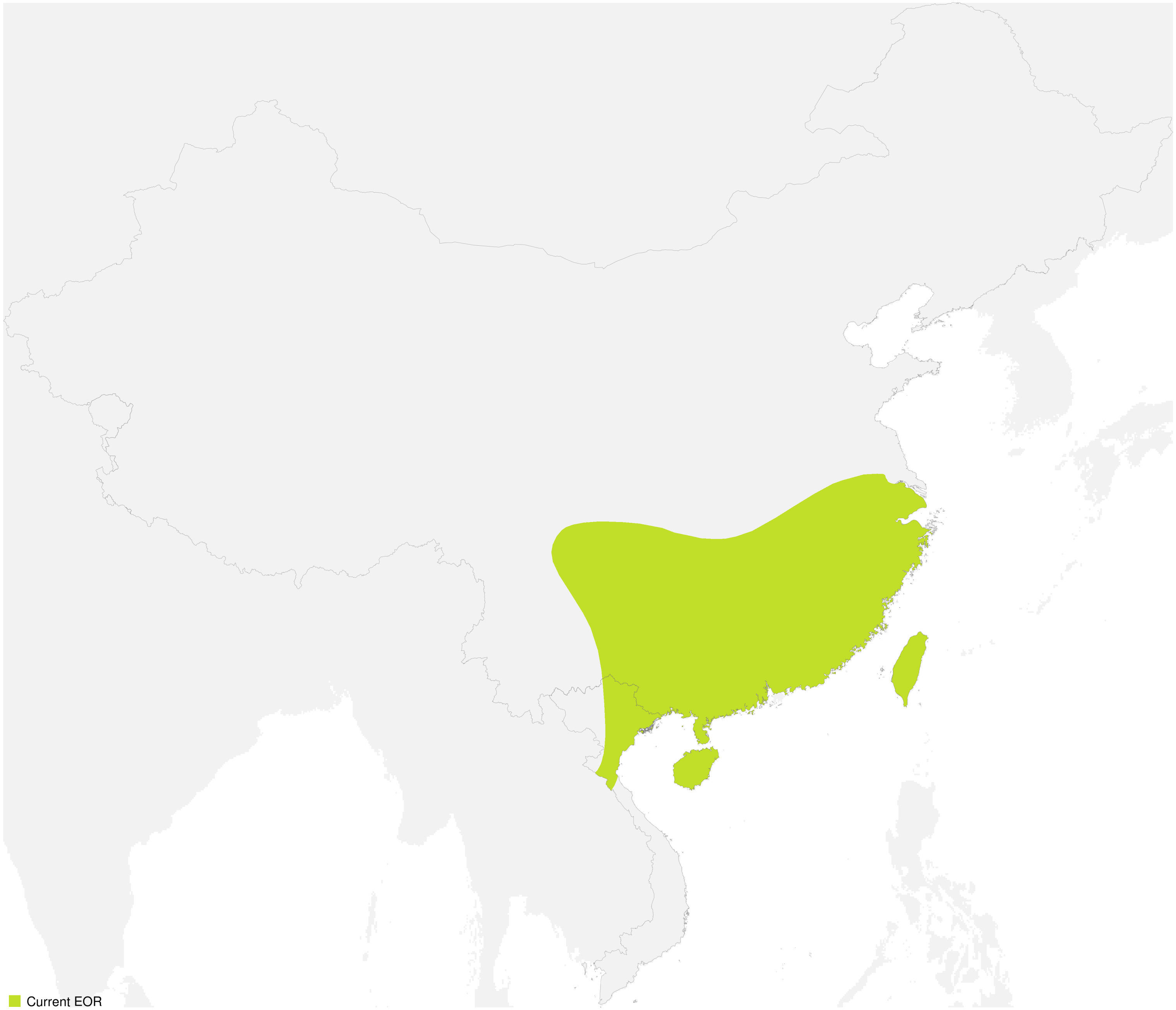

Walterinnesia aegyptia

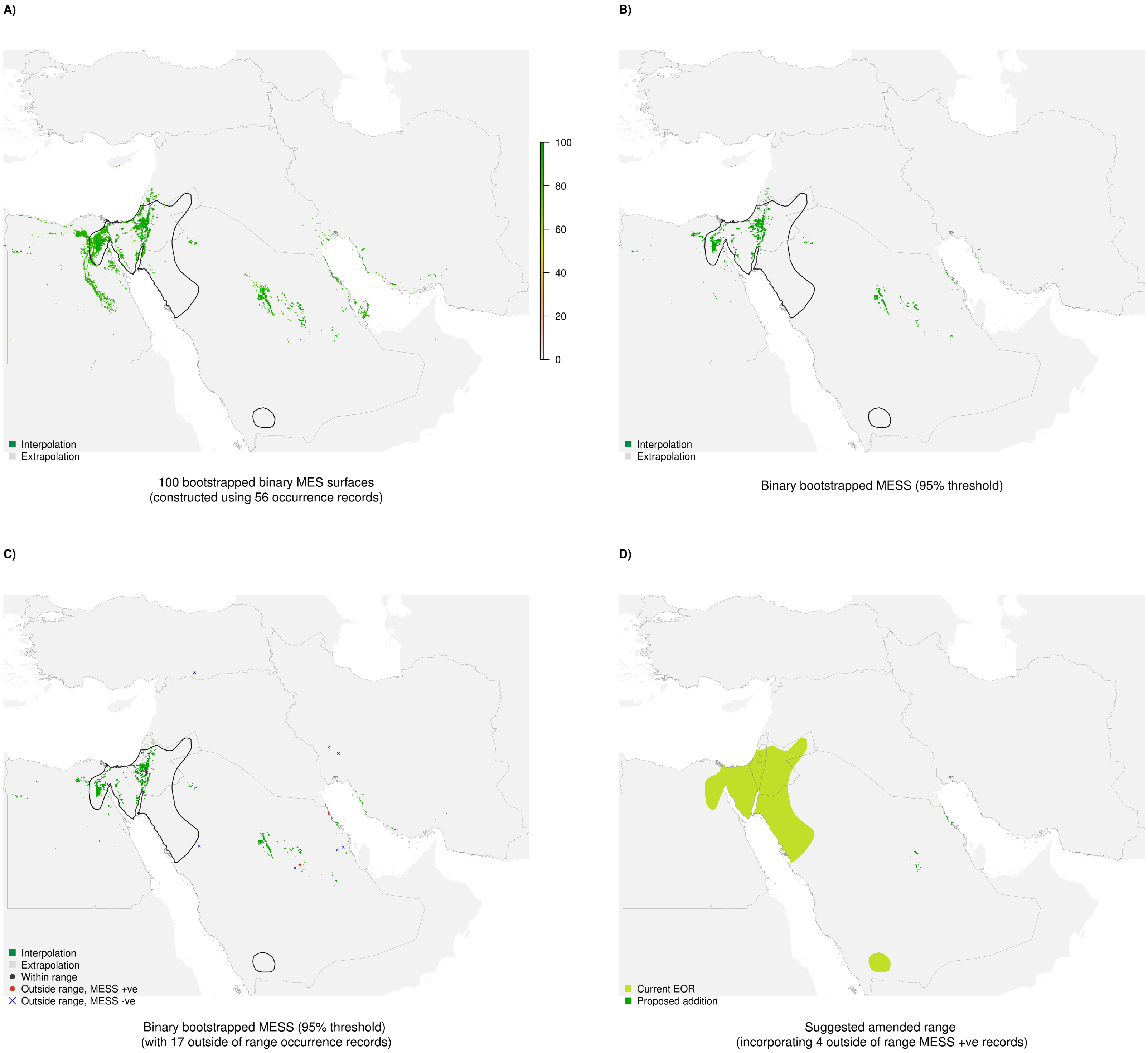

Walterinnesia morgani

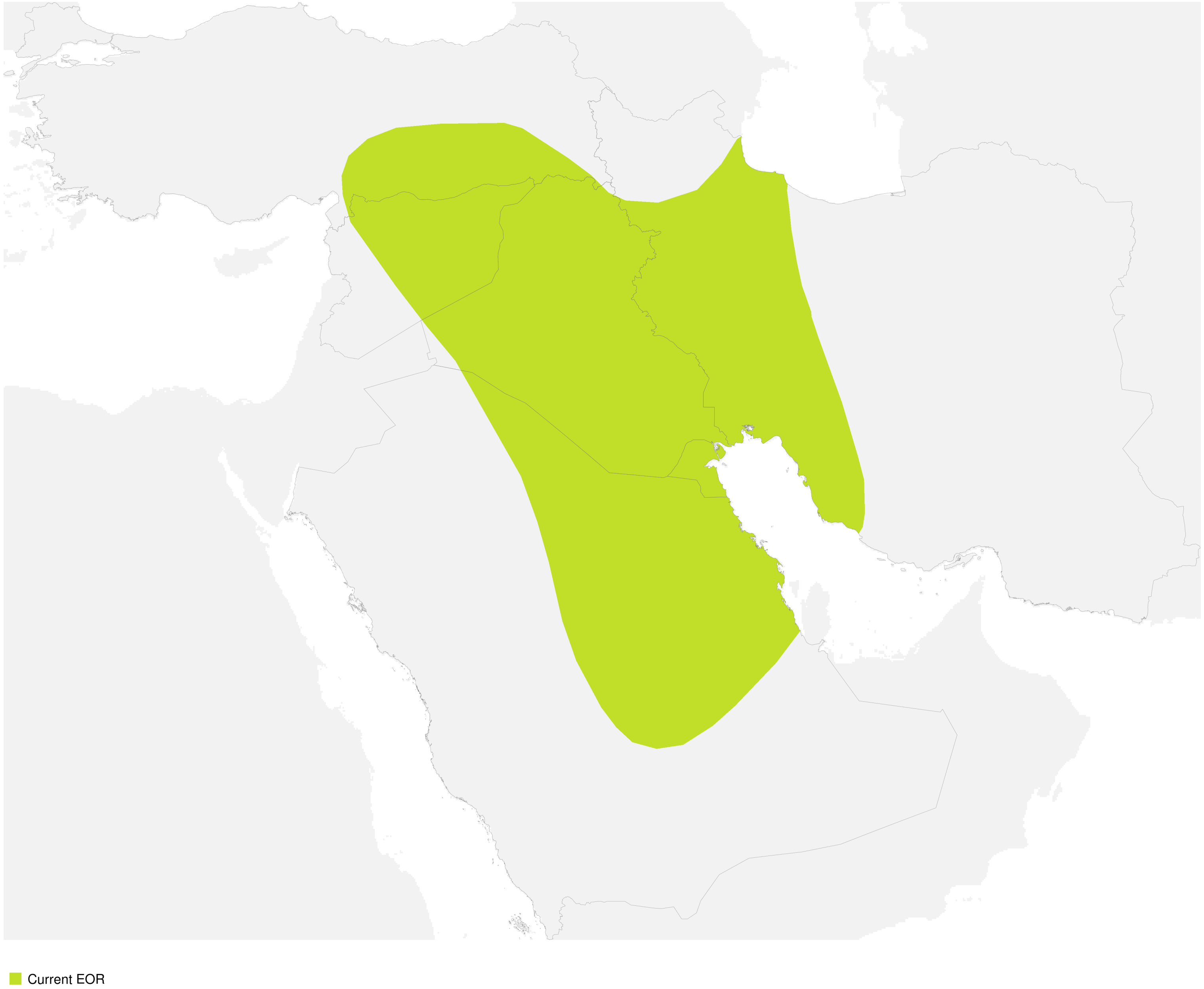

Supplement: Supplementary appendix 1 [file mmc1.pdf]
